# Supplementary material for: Elucidating the reaction mechanism of a palladium-palladium dual catalytic process through kinetic studies of proposed elementary steps
Source: Commun Chem. 2023 Mar 18;6:51. doi: 10.1038/s42004-023-00849-x (PMC10024772; doi:10.1038/s42004-023-00849-x)
Supplement: Supplementary file 2 — Supplementary Information file [file 42004_2023_849_MOESM2_ESM.pdf]

Supplementary Information for

Elucidating reaction mechanism of palladium-palladium dual catalytic process through kinetic studies of proposed elementary steps

Anže Ivančič, Janez Košmrlj, Martin Gazvoda*

University of Ljubljana, Faculty of Chemistry and Chemical Technology, Večna pot 113, 1001 Ljubljana, Slovenia

email: martin.gazvoda@fkkt.uni-lj.si

TABLE OF CONTENTS

1. SUPPLEMENTARY METHODS 1
1.1. General information 1
1.1.1. General reagent information 1
1.1.2. General experimental information 2
1.1.3. General analytical information 2
2. SUPPLEMENTARY NOTE 1 4
2.1. EXPERIMENTAL PROCEDURES AND CHARACTERIZATION DATA 4
2.1.1. Syntheses of haloalkynes 4
2.1.2. Syntheses of palladium complexes 12
2.1.3. Syntheses of 1,2-disubstituted alkynes **3** 46
2.1.4. Transmetallation reactions 54
2.1.5. Kinetic studies of the elementary steps of the mechanism 146
3. SUPPLEMENTARY REFERENCES 231

1. SUPPLEMENTARY METHODS

1.1. General information

1.1.1. General reagent information

All reagents purchased commercially (Fluorochem, Merck, Sigma Aldrich, TCI) were used without further purification unless noted otherwise.

Aryl halides, 4-iodotoluene (**1a**), 1-iodo-4-nitrobenzene (**1c**), 4-bromo-1-nitrobenzene (**1f**), 1-iodo-4-(*tert*-butyl)benzene (**1h**), (4-iodophenyl)methanol (**1i**), 1-iodo-4-(trifluoromethyl)benzene (**1k**), 4-iodobenzaldehyde (**1l**) and 4-iodoacetophenone (**1m**) were purchased from Fluorochem Ltd. 4-iodoanisole (**1b**), 4-bromotoluene (**1d**) and 4-bromoanisole (**1e**) were purchased from Sigma Aldrich, Merck KGaA. Iodobenzene (**1g**) and 4-chloro-1-iodobenzene (**1j**) were purchased from Fluka, Honeywell International Inc.

Terminal alkynes phenylacetylene (**2a**), 1-ethynyl-4-nitrobenzene (**2b**), 1-chloro-3-ethynylbenzene (**2c**), 2-ethynylpyridine (**2d**), ethynylcyclohexane (**2f**), 1-chloro-4-ethynylbenzene (**2j**), 1-bromo-4-ethynylbenzene (**2k**), 1-(trifluoromethyl)-4-ethynylbenzene (**2l**), 4-ethynylbenzaldehyde (**2m**), 4'-ethynylacetophenone (**2n**) and 2-ethynylthiophene (**2p**) were purchased from Fluorochem Ltd. 6-Chlorohex-1-yne (**2e**) and 1-ethynylcyclohexene (**2q**) were purchased from Sigma Aldrich, Merck KGaA, Darmstadt, Germany. 4-Ethynyltoluene (**2g**) and 4-ethynylanisole (**2h**) were purchased from Alfa Aesar, Thermo Fisher (Kandel) GmbH. 1-Ethynyl-(4-*tert*-butyl)benzene (**2i**) was purchased from Apollo Scientific Ltd. 4-(Trimethylsilyl)phenylacetylene (**2o**) TCI Europe N.V.

N-Iodosuccinimide, *N*-bromosuccinimide, *N*-chlorosuccinimide, triphenylphosphine, copper iodide, tetrabutylammonium chloride and triethylamine were purchased from Fluorochem Ltd. Sodium sulfate (Na_2SO_4), potassium carbonate (K_2CO_3), silver acetate, 1,3,5-trimethoxybenzene and triphenyl phosphate were purchased from Sigma Aldrich, Merck KGaA. Morpholine, NH_4Cl, NaOH, NaI and pyrrolidine were purchased from Fluka, Honeywell. Iodine was purchased from abcr GmbH. $NaHCO_3$ was purchased from Gram-mol d.o.o., Croatia. Silver nitrate ($AgNO_3$) was purchased from Alkaloid AD Skopje. Tetrakis(triphenylphosphine)palladium(0) and bis(triphenylphosphine)palladium(II) dichloride were purchased from Fluorochem Ltd. Bis(tri(*o*-tolyl)phosphine)palladium(0) was purchased from abcr.

All solvents used were HPLC grade. 1-Propanol, benzene, and *N*,*N*-dimethylformamide (DMF) were purchased from Sigma Aldrich, Merck KGaA. Hexane was purchased from Honeywell. Toluene, ethyl acetate, dichloromethane and chloroform were purchased from CARLO ERBA Reagents S.A.S. Diethyl ether and methanol were purchased from J. T. Baker, VWR International, LLC.

Benzene and toluene were distilled over a sodium wire. Dichloromethane was distilled over CaH_2. Chloroform was distilled over P_2O_5 and stored over activated molecular sieves (4 Å). Ethyl acetate and petroleum ether for column chromatography were obtained from CARLO ERBA Reagents S.A.S. and were used as received. All deuterated solvents were purchased from Euriso-top. $CDCl_3$ for transmetallation reactions was degassed and stored under argon.

Column chromatography was carried out on Fluka silica gel 60N (mesh 220-240). Thin-layer chromatography (TLC) was performed on Fluka analytical TLC plates (silica gel matrix, with fluorescent indicator, 60 Å medium pore diameter). Visualization of compounds was done by illumination with an UV lamp (254 nm or 366 nm).

1.1.2. General experimental information

All reactions were carried out in oven-dried glassware under argon atmosphere. Reactions were usually carried out in 5-, 10-, 25- or 50-mL round-bottom reaction flasks sealed with a rubber septum. Reactions under high pressure were conducted in ~21 mL Merck's Ace pressure tubes (Cat. No. Z564621) with a screw cap in a heating block. The transmetallation reactions were performed in Wilmad Precision NMR Sample Tubes.

1.1.3. General analytical information

^{1}H, ^{19}F{^{1}H}, ^{31}P{^{1}H} and ^{13}C{^{1}H} NMR spectra were recorded with a Bruker Avance NEO 600 MHz NMR (600 MHz, 565 MHz, 243 MHz and 151 MHz) instrument, Bruker Avance III 500 MHz NMR (500 MHz, 471 MHz, 202 MHz and 126 MHz) instrument at 296 or 302 K or with a Bruker Avance DPX 300 spectrometer (300 MHz, 122 MHz and 76 MHz) at 302 K. Proton spectra were referenced to residual signal of $CHCl_3$ (δ = 7.26 ppm). Carbon chemical shifts were determined relative to the central ^{13}C signal of $CDCl_3$ (77.16 ppm) and were acquired with a Bruker ^{13}C composite pulse decoupling (CPD) program. ^{31}P{^{1}H} NMR spectra were referenced to external 85% phosphoric acid (δ = 0 ppm) and were acquired with a Bruker ^{31}P composite pulse decoupling (CPD) program. ^{19}F{^{1}H} NMR spectra were referenced to external CCl_3F (δ = 0 ppm) and were acquired with a Bruker ^{19}F composite pulse decoupling (CPD) program. Chemical shifts (δ) are given in ppm. Coupling constants (*J*) are given in Hz. Multiplicities are indicated as follows: s (singlet), d (doublet), t (triplet), m (multiplet) or br (broadened).

NMR conversions were determined by ^{1}H NMR by comparing integrals of resonances of corresponding product with integrals of resonances of internal standard 1,3,5-trimethoxybenzene (δ = 3.75 ppm, δ = 6.08 ppm) or by ^{31}P NMR by comparing integrals of resonances of corresponding product with integral of resonance of triphenyl phosphate as an internal standard (δ = −17.3 ppm).

NMR kinetics experiments were performed on a Bruker Avance DPX 300 spectrometer (300 MHz for ^{1}H kinetics or 122 MHz for ^{31}P kinetics) at 302 K, recycle delay 2 s, typical sleep time 300 s, with spin rotation, on a Bruker Avance III 500 MHz NMR spectrometer (500 MHz for ^{1}H kinetics or 202 MHz for ^{31}P kinetics), recycle delay 2 s, typical sleep time 147 s, with spin rotation, and on a Bruker Avance NEO 600 MHz NMR spectrometer (600 MHz for ^{1}H kinetics), recycle delay 2 s, typical sleep time 147 s, with spin rotation.

HRMS spectra were recorded with Agilent 6224 Accurate Mass TOF LC/MS spectrometer equipped with a double orthogonal electrospray source at atmospheric pressure ionization (ESI) coupled to a HPLC instrument.

IR spectra were obtained with a Perkin-Elmer Spectrum 100, equipped with a Specac Golden Gate Diamond ATR as a solid sample support.

Melting points were determined on a Kofler micro hot stage.

2. SUPPLEMENTARY NOTE 1

2.1. EXPERIMENTAL PROCEDURES AND CHARACTERIZATION DATA

2.1.1. Syntheses of haloalkynes

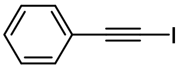

4a
98%

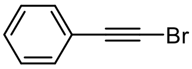

4b
82%

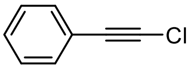

4c
62%

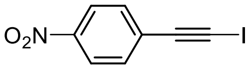

4d
24%

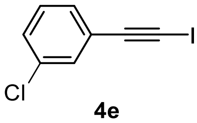

4e
44%

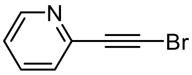

4f
69%

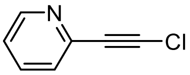

4g
23%

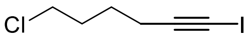

4h
52%

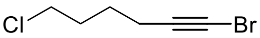

4i
86%

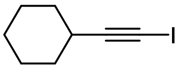

4j
93%

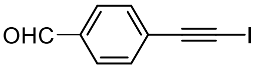

4k
82%

Syntheses of iodoalkynes

General procedure 1 (GP1) – Synthesis of 1-iodoalkynes 4 from terminal alkynes 2

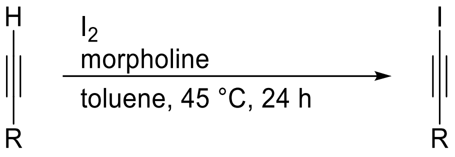

2 → **4**

1-Iodoalkynes **4** were prepared according to the modified literature procedure.[1] An oven-dried round-bottom reaction flask was fitted with a stirring bar and closed with a rubber septum, which was pierced with a needle connected by a tube to a Schlenk vacuum manifold. The flask was cooled to room temperature under argon atmosphere and charged with iodine (1.1 equiv.) and toluene (3 mL/mmol), respectively. Morpholine (3 equiv.) was slowly added to the solution and the resulting red suspension was stirred vigorously in the dark at room temperature for 30 min, followed by the addition of terminal alkyne **2** (1 equiv.). The mixture was stirred overnight in the dark at 45 °C. The reaction mixture was then cooled to room temperature and filtered.

The residue was washed with EtOAc (3 × 10 mL). The filtrate was washed with saturated aqueous NH_4Cl solution (20 mL), saturated aqueous $NaHCO_3$ solution (20 mL), water (20 mL) and saturated aqueous NaCl solution (2 × 10 mL). The organic phase was dried over anhydrous Na_2SO_4 and filtered. The filtrate was concentrated *in vacuo* using a rotary evaporator and the crude product was purified by column chromatography. The fractions containing the product were combined and the volatile components were evaporated *in vacuo* using a rotary evaporator.

Synthesis of 1-iodo-2-phenylacetylene (4a)

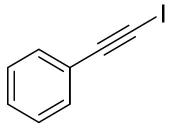

Following *GP1*. Prepared from phenylacetylene (**2a**) (1.10 mL, 10.0 mmol), toluene (30 mL), iodine (2.83 g, 11.2 mmol), morpholine (2.6 mL, 30.1 mmol). Purified by column chromatography (mobile phase petroleum ether/ethyl acetate = 20:1). Yield: 2.23 g (9.78 mmol, 98 %) of yellow oil.

^{1}H NMR (300 MHz, $CDCl_3$) δ = 7.48–7.39 (m, 2H), 7.35–7.28 (m, 3H).

Spectroscopic data are in agreement with literature data.[2]

Synthesis of 1-(iodoethynyl)-4-nitrobenzene (4d)

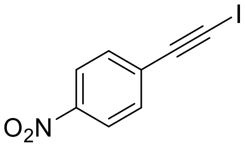

Following *GP1*. Prepared from 1-ethynyl-4-nitrobenzene (**2b**) (0.749 mg, 5.09 mmol), toluene (15 mL), iodine (1.41 g, 5.56 mmol), morpholine (1.3 mL, 15.1 mmol). Purified by column chromatography (mobile phase petroleum ether/ethyl acetate = 1:1). Yield: 339 mg (1.24 mmol, 24 %) of yellow solid.

Mp: decomposes at 160.4 °C.

^{1}H NMR (500 MHz, $CDCl_3$) δ = 8.19 (d, J = 8.9 Hz, 2H), 7.58 (d, J = 8.9 Hz, 2H).

Spectroscopic data are in agreement with literature.[3]

Synthesis of 1-chloro-3-(iodoethynyl)benzene (4e)

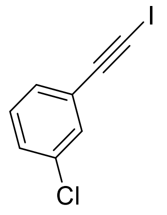

Following *GP1*. Prepared from 1-chloro-3-ethynylbenzene (**2c**) (0.615 mL, 5.00 mmol), toluene (15 mL), iodine (1.42 g, 5.59 mmol), morpholine (1.3 mL, 15.1 mmol). Purified by column chromatography (mobile phase petroleum ether/ethyl acetate = 20:1). Yield: 580 mg (2.21 mmol, 44 %) of yellowish oil.

^{1}H NMR (500 MHz, $CDCl_3$) δ = 7.42 (t, J = 1.7 Hz, 1H), 7.33–7.28 (m, 2H), 7.24 (t, J = 7.8 Hz, 1H).

Spectroscopic data are in agreement with literature.[4]

Synthesis of 6-chloro-1-iodohex-1-yne (4h)

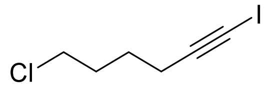

Following *GP1*. Prepared from 6-chlorohex-1-yne (**2e**) (0.370 mL, 3.05 mmol), toluene (10 mL), iodine (837 mg, 3.30 mmol), morpholine (0.777 mL, 9.01 mmol). Purified by column chromatography (mobile phase petroleum ether/ethyl acetate = 20:1). Yield: 389 mg (1.60 mmol, 52 %) of colourless oil.

IR (cm^{-1}): 2953, 1728, 1712, 1579, 1555, 1515, 1371, 1347, 1226, 1146, 1077, 857, 837, 764, 704, 651.

^{1}H NMR (500 MHz, $CDCl_3$) δ = 3.56 (t, J = 6.5 Hz, 2H), 2.41 (t, J = 7.0 Hz, 2H), 1.92–1.83 (m, 2H), 1.72–1.62 (m, 2H).

^{13}C{^{1}H} NMR (126 MHz, $CDCl_3$) δ = 93.8, 77.4, 44.6, 31.5, 25.7, 20.2.

Synthesis of (iodoethynyl)cyclohexane (4j)

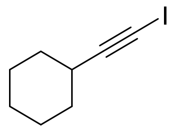

Following *GP1*. Prepared from ethynylcyclohexane (**2f**) (0.650 mL, 4.98 mmol), toluene (15 mL), iodine (1.43 g, 5.63 mmol), morpholine (1.3 mL, 15.1 mmol). Purified by column

chromatography (mobile phase petroleum ether/ethyl acetate = 20:1). Yield: 1.09 g (4.66 mmol, 93 %) of yellow oil.

^{1}H NMR (500 MHz, $CDCl_3$) δ = 2.62–2.45 (m, 1H), 1.86–1.74 (m, 2H), 1.74–1.61 (m, 2H), 1.54–1.37 (m, 3H), 1.36–1.22 (m, 3H).

Spectroscopic data are in accordance with literature.[5]

Synthesis of 4-(iodoethynyl)benzaldehyde (4k)

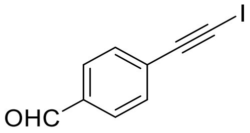

Prepared according to alternative procedure[6]. In a round bottom flask 4-ethynylbenzaldehyde (**2m**) (517 mg, 3.97 mmol) was dissolved in acetone (20 mL). To this solution *N*-iodosuccinimide (1.175 g, 5.22 mmol) and $AgNO_3$ (103 mg, 0.61 mmol) were added. Resulting mixture was stirred overnight. After 20 h the reaction mixture was concentrated *in vacuo*. Residue was purified using column chromatography (mobile phase petroleum ether/ethyl acetate = 1:1). Fractions that contained product were united and solvent was evaporated *in vacuo*. Yield: 833 mg (3.25 mmol, 82 %) of yellow powder.

Mp: decomposes at 110.6 °C.

^{1}H NMR (500 MHz, $CDCl_3$) δ = 10.01 (s, 1H), 7.83 (d, J = 8.4 Hz, 2H), 7.58 (d, J = 8.2 Hz, 2H).

Spectroscopic data are in agreement with literature.[6]

Syntheses of bromoalkynes

General procedure 2 (*GP2*) – Synthesis of 1-bromoalkynes 4 from terminal alkynes 2

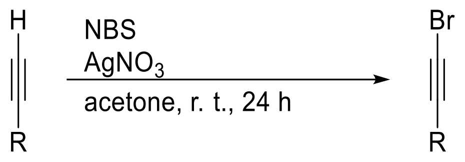

2 **4**

1-Bromoalkynes **4** were prepared according to the modified literature procedure.[7] An oven-dried round-bottom reaction flask was fitted with a stirring bar and closed with a rubber septum, which was pierced with a needle connected by a tube to a Schlenk vacuum manifold. The flask was cooled to room temperature under an argon atmosphere and charged with $AgNO_3$ (0.1 equiv.) and *N*-bromosuccinimide (1.2 equiv.), followed by the addition of acetone (5 mL). To this suspension was added the terminal alkyne **2** (1 equiv.) was added and the reaction mixture was vigorously stirred overnight at room temperature. The reaction mixture was then concentrated *in vacuo* using a rotary evaporator and purified by column chromatography. The fractions containing the product were combined and the volatile components were evaporated using a rotary evaporator.

Synthesis of (bromoethynyl)benzene (4b)

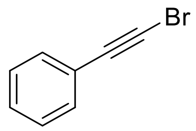

Following *GP2*. Prepared from phenylacetylene (**2a**) (0.440 mL, 4.01 mmol), acetone (20 mL), $AgNO_3$ (76 mg, 0.45 mmol), *N*-bromosuccinimide (865 mg, 4.86 mmol). Purified by column chromatography (mobile phase petroleum ether/ethyl acetate = 20:1). Yield: 594 mg (3.28 mmol, 82 %) of yellow oil.

^{1}H NMR (500 MHz, $CDCl_3$) δ = 7.45 (dd, *J* = 8.1, 1.6 Hz, 2H), 7.37–7.28 (m, 3H).

Spectroscopic data are in agreement with literature.[8]

Synthesis of 2-(bromoethynyl)pyridine (4f)

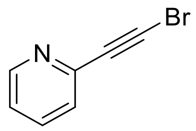

Following *GP2*. Prepared from 2-ethynylpyridine (**2d**) (0.505 mL, 5.00 mmol), acetone (25 mL), $AgNO_3$ (82 mg, 0.48 mmol), *N*-bromosuccinimide (1.08 g, 6.07 mmol). Purified by column chromatography (mobile phase petroleum ether/ethyl acetate = 5:1). Yield: 631 mg (3.47 mmol, 69 %) of brownish oil.

^{1}H NMR (500 MHz, $CDCl_3$) δ = 8.57 (ddd, J = 4.9, 1.7, 1.1 Hz, 1H), 7.66 (td, J = 7.7, 1.8 Hz, 1H), 7.43 (dt, J = 7.9, 1.0 Hz, 1H), 7.27 (ddd, J = 7.6, 4.9, 1.1 Hz, 1H).

Spectroscopic data are in agreement with literature.[9]

Synthesis of 1-bromo-6-chlorohexyne (4i)

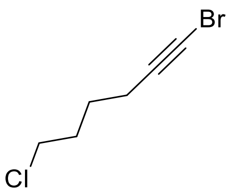

Following *GP2*. Prepared from 6-chloro-1-hexyne (**2e**) (1.212 mL, 10.0 mmol), acetone (50 mL), $AgNO_3$ (180 mg, 1.06 mmol), *N*-bromosuccinimide (2.16 g, 12.1 mmol). Purified by column chromatography (mobile phase petroleum ether/ethyl acetate = 2:1). Yield: 1.688 g (8.63 mmol, 86 %) of yellow oil.

^{1}H NMR (500 MHz, $CDCl_3$) δ = 3.56 (t, J = 6.5 Hz, 2H), 2.27 (t, J = 6.9 Hz, 2H), 1.93–1.84 (m, 2H), 1.73–1.63 (m, 2H).

Spectroscopic data are in agreement with literature.[10]

Syntheses of chloroalkynes

General procedure 3 (*GP3*) – Synthesis of 1-chloroalkynes 4 from terminal alkynes 2

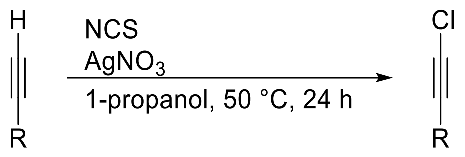

1-Chloroalkynes **4** were prepared according to the modified literature procedure.[11] An oven-dried Ace reaction tube was fitted with a stirring bar, cooled under argon to room temperature and was charged with $AgNO_3$ (1 equiv.), K_2CO_3 (1 equiv.) and *N*-chlorosuccinimide (2 equiv.). Then 1-propanol (20 mL) and the terminal alkyne **2** (1 equiv.) were added, respectively. Suspension was stirred vigorously at 50 °C for 2 h. The reaction mixture was then cooled to 0 °C, diluted with Et_2O (10 mL) and filtered and the residue was washed with Et_2O (3 × 10 mL). Filtrate was collected and washed with saturated aqueous NaCl solution (3 × 10 mL). The organic phase was dried over anhydrous Na_2SO_4, filtered and concentrated *in vacuo* using a rotary evaporator. The crude product was purified by column chromatography. The fractions containing the product were combined and volatile components were evaporated using rotary evaporator.

Synthesis of (chloroethynyl)benzene (4c)

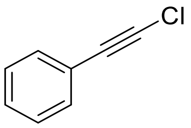

Following *GP3*. Prepared from phenylacetylene (**2a**) (1.10 mL, 10.0 mmol), 1-propanol (18 mL), $AgNO_3$ (1.85 mg, 10.9 mmol), K_2CO_3 (1.67 g, 12.1 mmol), *N*-chlorosuccinimide (2.69 g, 20.1 mmol). Purified by column chromatography (mobile phase petroleum ether). Yield: 847 mg (6.20 mmol, 62 %) of colorless oil.

^{1}H NMR (500 MHz, $CDCl_3$) δ = 7.44 (dd, J = 8.0, 1.8 Hz, 2H), 7.36–7.28 (m, 3H).

Spectroscopic data are in agreement with literature.[12]

Synthesis of 2-(chloroethynyl)pyridine (4g)

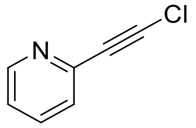

Following *GP3*. Prepared from 2-ethynylpyridine (**2d**) (0.510 mL, 5.05 mmol), 1-propanol (8 mL), $AgNO_3$ (1.06 g, 6.25 mmol), K_2CO_3 (776 mg, 12.1 mmol), *N*-chlorosuccinimide (1.69 g, 12.6 mmol). Purified by column chromatography (mobile phase dichloromethane/methanol = 50:1). Yield: 163 mg (1.18 mmol, 23 %) of yellow oil.

^{1}H NMR (500 MHz, $CDCl_3$) δ = 8.57 (ddd, J = 4.9, 1.7, 0.7 Hz, 1H), 7.65 (dt, J = 7.8, 1.8 Hz, 1H), 7.43 (dt, J = 7.8, 1.0 Hz, 1H), 7.25 (ddd, J = 7.6, 4.9, 1.2 Hz, 1H).

Spectroscopic data are in agreement with literature.[13]

2.1.2. Syntheses of palladium complexes

Syntheses of bis(triphenylphosphine)palladium(II) alkynylides halides 5

General procedure 4 (*GP4*) – Synthesis of bis(triphenylphosphine)palladium(II) acetylide halides 5 from haloacetylenes 4

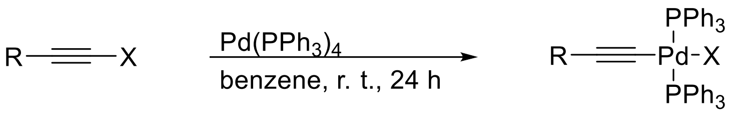

4 **5**

Bis(triphenylphosphine)palladium(II) acetylide halides **5** were prepared according to the modified literature procedure.[14] An oven-dried round-bottom reaction flask was fitted with a stirring bar and closed with a rubber septum, which was pierced with a needle connected by a tube to a Schlenk vacuum manifold. The flask was cooled to room temperature under argon atmosphere. The flask was charged with haloacetylene **4** (1.2 equiv.) by quickly opening the septum and flushing with argon. Benzene (10 mL/mmol) was added with a syringe by piercing the septum, and then tetrakis(triphenylphosphine)palladium (1 equiv.) was added by quickly opening the septum and flushing with argon. The reaction mixture was purged with argon for 5 min. Argon was applied by the needle connected to a Schlenk vacuum manifold. The reaction mixture was stirred overnight in the dark at room temperature. The reaction mixture was then concentrated *in vacuo* using a rotary evaporator. The oily residue was suspended in MeOH (20 mL) using an ultrasonic bath (1 min). The suspension was filtered and the solid residue was washed with MeOH (10 mL), Et_2O (10 mL) and dried to give the desired product.

Compounds **5** were generally not stable for extended periods of time: they were stable for about 1 hour in $CDCl_3$ solution, and for a week in a freezer (–20 °C) under an argon atmosphere. Therefore, compounds **5** were not further purified but were characterized as obtained by the procedure described above. The compounds were mostly in >85% purity as judged by NMR (see corresponding ^{1}H and $^{31}P\{^{1}H\}$ NMR spectra in the 'Copies of NMR spectra').

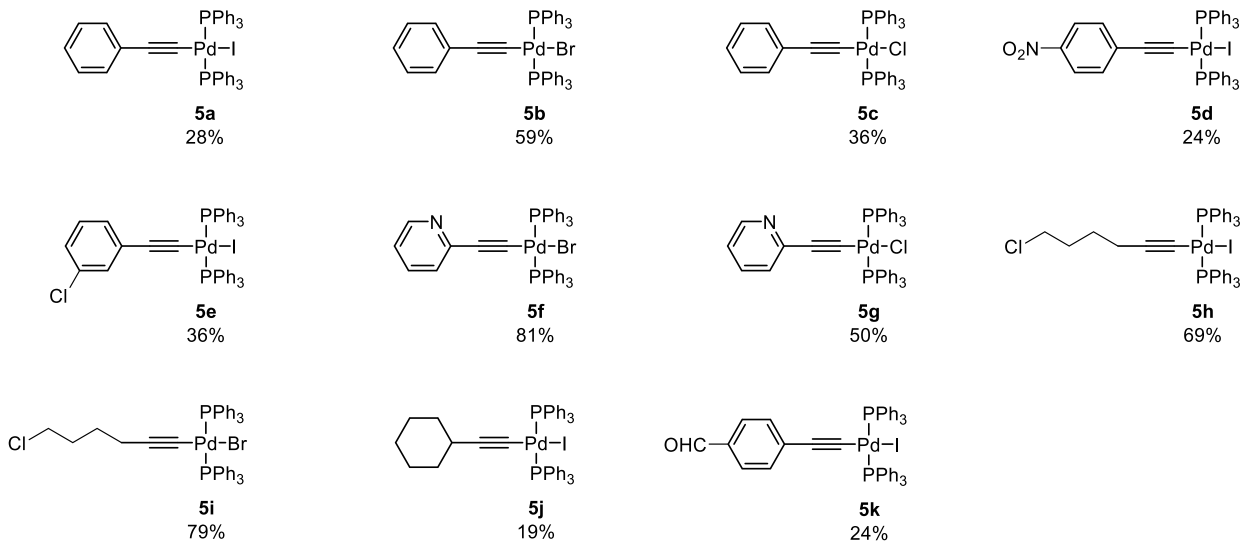

Synthesis of bis(triphenylphosphine)palladium(II) (phenyl)ethyn-1-ide iodide (5a)

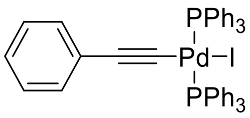

Following *GP4*. Prepared from tetrakis(triphenylphosphine)palladium (577 mg, 0.50 mmol), (iodoethynyl)benzene (**4a**) (182 mg, 0.80 mmol), benzene (5 mL). Yield: 120 mg (0.140 mmol, 28 %) of yellow powder.

Mp: decomposes at 153.6 °C.

^{1}H NMR (500 MHz, $CDCl_3$) δ = 7.83–7.73 (m, 12H), 7.43–7.32 (m, 18H), 6.93–6.87 (m, 1H), 6.87–6.81 (m, 2H), 6.11–6.04 (m, 2H).

^{31}P{^{1}H} NMR (202 MHz, $CDCl_3$) δ = +22.8 (s).

Spectroscopic data are in agreement with literature.[15]

Synthesis of bis(triphenylphosphine)palladium(II) (phenyl)ethyn-1-ide bromide (5b)

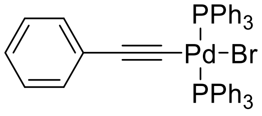

Following *GP4*. Prepared from tetrakis(triphenylphosphine)palladium (631 mg, 0.55 mmol), 1-bromo-ethynylbenzene (**4b**) (148 mg, 0.82 mmol), benzene (7 mL). Yield: 262 mg (0.323 mmol, 59 %) of yellow powder.

Mp: 169.3–170.1 °C.

IR (cm^{-1}): 3052, 2118, 1595, 1571, 1481, 1434, 1092, 752, 741, 691.

^{1}H NMR (500 MHz, $CDCl_3$) δ = 7.80–7.74 (m, 12H), 7.44–7.33 (m, 18H), 6.93–6.89 (m, 1H), 6.88–6.83 (m, 2H), 6.10 (d, J = 7.1 Hz, 2H).

^{13}C{^{1}H} NMR (126 MHz, $CDCl_3$) δ = 135.2 (t, J = 6.3 Hz), 131.7 (t, J = 25.1 Hz), 130.7, 130.3, 128.1 (t, J = 5.3 Hz), 127.4, 127.2, 125.3, 111.0 (t, J = 6.8 Hz), 98.9 (t, J = 14.2 Hz).

^{31}P{^{1}H} NMR (202 MHz, $CDCl_3$) δ = +24.2 (s).

HRMS: calculated for $C_{44}H_{35}P_2Pd$ $[M\text{-}Br]^+$ 731.1249, found 731.1250.

Synthesis of bis(triphenylphosphine)palladium(II) (phenyl)ethyn-1-ide chloride (5c)

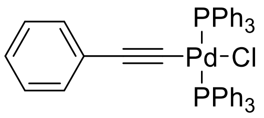

Following *GP4*. Prepared from tetrakis(triphenylphosphine)palladium (586 mg, 0.51 mmol), 1-chloro-4-ethynylbenzene (**4c**) (142 mg, 1.04 mmol), benzene (7 mL). Yield: 142 mg (0.185 mmol, 36 %) of white powder.

Mp: decomposes at 142.5 °C.

^{1}H NMR (500 MHz, $CDCl_3$) δ = 7.80–7.73 (m, 12H), 7.45–7.34 (m, 18H), 6.94–6.83 (m, 3H), 6.11 (d, J = 7.2 Hz, 2H).

^{31}P{^{1}H} NMR (122 MHz, $CDCl_3$) δ = +24.5 (s).

Spectroscopic data are in agreement with literature.[12]

Synthesis of bis(triphenylphosphine)palladium(II) (4-nitrophenyl)ethyn-1-ide iodide (5d)

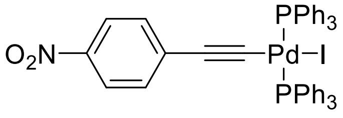

Following *GP4*. Prepared from tetrakis(triphenylphosphine)palladium (572 mg, 0.495 mmol), 1-(iodoethynyl)-4-nitrobenzene (**4d**) (169 mg, 0.619 mmol), benzene (8 mL). Yield: 108 mg (0.119 mmol, 24 %) of yellow powder.

Mp: 148.8–151.5 °C.

IR (cm^{-1}): 3051, 2119, 1584, 1507, 1478, 1433, 1338, 1171, 1094, 997, 852, 741, 689, 633.

^{1}H NMR (500 MHz, $CDCl_3$) δ = 7.80–7.71 (m, 14H), 7.45–7.34 (m, 18H), 6.13 (d, J = 8.8 Hz, 2H).

^{13}C{^{1}H} NMR (126 MHz, $CDCl_3$) δ = 144.8, 135.1 (t, J = 6.4 Hz), 132.4 (t, J = 25.6 Hz), 130.5, 128.1 (t, J = 5.5 Hz), 122.8, 114.0 (t, J = 13.8 Hz), 108.5 (t, J = 6.5 Hz).

^{31}P{^{1}H} NMR (202 MHz, $CDCl_3$) δ = +22.7 (s).

HRMS: calculated for $C_{44}H_{34}NO_2P_2Pd$ [M-I]$^+$ 776.1100, found 776.1101.

Synthesis of bis(triphenylphosphine)palladium(II) (3-chlorophenyl)ethyn-1-ide iodide (5e)

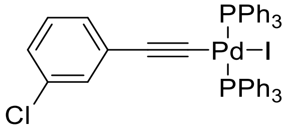

Following *GP4*. Prepared from tetrakis(triphenylphosphine)palladium (921 mg, 0.797 mmol), 1-chloro-3-(iodoethynyl)benzene (**4e**) (330 mg, 1.26 mmol), benzene (10 mL). Yield: 254 mg (0.284 mmol, 36 %) of yellow powder.

Mp: 170.7–172.3 °C.

IR (cm^{-1}): 3074, 3052, 2120, 2074, 1586, 1480, 1467, 1433, 1099, 868, 780, 741, 705, 689, 618.

^{1}H NMR (500 MHz, $CDCl_3$) δ = 7.79–7.73 (m, 12H), 7.44–7.34 (m, 18H), 6,87 (dt, J = 8.1, 0.9 Hz, 1H), 6.76 (t, J = 7.9 Hz, 1H), 5.95 (dd, J = 7.7, 1.1 Hz, 1H), 5.92 (d, J = 1.6 Hz, 1H).

^{13}C{^{1}H} NMR (126 MHz, $CDCl_3$) δ = 135.1 (t, J = 6.3 Hz), 132.9, 132.6 (t, J = 25.4 Hz), 130.8, 130.4, 128.8, 128.6, 128.3, 128.0 (t, J = 5.4 Hz), 125.4, 108.1 (t, J = 7.2 Hz), 104.9 (t, J = 13.8 Hz).

^{31}P{^{1}H} NMR (202 MHz, $CDCl_3$) δ = +22.8 (s).

HRMS: calculated for $C_{44}H_{34}ClP_2Pd$ [M-I]$^+$ 767.0863, found 767.0855.

Synthesis of bis(triphenylphosphine)palladium(II) (2-pyridyl)ethyn-1-ide bromide (5f)

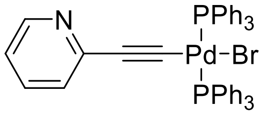

Following *GP4*. Prepared from tetrakis(triphenylphosphine)palladium (575 mg, 0.50 mmol), (2-bromoethynyl)pyridine (**4f**) (139 mg, 0.76 mmol), benzene (6 mL). Yield: 329 mg (0.405 mmol, 81 %) of grey powder.

Mp: decomposes at 171.0 °C.

IR (cm^{-1}): 2114, 1583, 1478, 1459, 1432, 1421, 1310, 1239, 1183, 1146, 1094, 1027, 997, 785, 751, 743, 692.

^{1}H NMR (500 MHz, $CDCl_3$) δ = 8.20 (d, J = 4.3 Hz, 1H), 7.82–7.73 (m, 12H), 7.39–7.31 (m, 18H), 7.14 (dt, J = 7.7, 1.6 Hz, 1H), 6.80 (dd, J = 6.6, 5.2 Hz, 1H), 6.07 (d, J = 7.9 Hz, 1H).

$^{13}C\{^{1}H\}$ NMR (126 MHz, $CDCl_3$) δ = 148.7, 146.0, 135.2 (t, J = 6.2 Hz), 134.6, 131.4 (t, J = 25.5 Hz), 130.4, 128.1 (t, J = 5.5 Hz), 125.8, 119.9, 110.9 (t, J = 7.0 Hz), 104.6 (t, J = 14.1 Hz).

$^{31}P\{^{1}H\}$ NMR (202 MHz, $CDCl_3$) δ = +23.6 (s).

HRMS: calculated for $C_{43}H_{34}NP_2Pd$ $[M-Br]^+$ 732.1201, found 732.1205.

Synthesis of bis(triphenylphosphine)palladium(II) 2-(2-pyridyl)ethyn-1-ide chloride (5g)

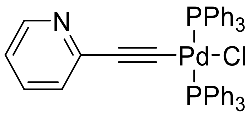

Following *GP4*. Prepared from tetrakis(triphenylphosphine)palladium (550 mg, 0.476 mmol), (2-chloroethynyl)pyridine (**4g**) (97 mg, 0.705 mmol), benzene (7 mL). Yield: 184 mg (0.24 mmol, 50 %) of grey powder.

Mp: decomposes at 142.1 °C.

^{1}H NMR (500 MHz, $CDCl_3$) δ = 8.22 (d, J = 3.9 Hz, 1H), 7.81–7.72 (m, 12H), 7.41–7.29 (m, 18H), 7.15 (t, J = 7.3 Hz, 1H), 6.81 (t, J = 5.4 Hz, 1H), 6.08 (d, J = 7.9 Hz, 1H).

$^{31}P\{^{1}H\}$ NMR (202 MHz, $CDCl_3$) δ = +23.9 (s).

Spectroscopic data are in agreement with literature.[16]

Synthesis of bis(triphenylphosphine)palladium(II) 6-chlorohexyn-1-ide iodide (5h)

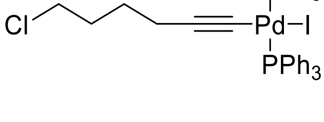

Following *GP4*. Prepared from tetrakis(triphenylphosphine)palladium (1.137 g, 0.984 mmol), 6-chloro-1-iodohex-1-yne (**4h**) (318 mg, 1.31 mmol), benzene (10 mL). Yield: 597 mg (0.683 mmol, 69 %) of orange powder.

Mp: 129.8–131.3 °C.

IR (cm^{-1}): 3057, 1480, 1434, 1095, 998, 740, 703, 690, 647.

^{1}H NMR (500 MHz, $CDCl_3$) δ = 7.79–7.70 (m, 12H), 7.40–7.32 (m, 18H), 3.05 (t, J = 6.7 Hz, 2H), 1.36 (tt, J = 6.7, 2.0 Hz, 2H), 1.13–1.03 (m, 2H), 0.78–0.68 (m, 2H).

^{13}C{^{1}H} NMR (126 MHz, $CDCl_3$) δ = 135.2 (t, J = 6.3 Hz), 133.0 (t, J = 25.3 Hz), 130.2, 127.8 (t, J = 5.4 Hz), 109.0 (t, J = 7.2 Hz), 87.0 (t, J = 13.2 Hz), 45.0, 31.2, 25.9, 20.1.

^{31}P{^{1}H} NMR (202 MHz, $CDCl_3$) δ = +22.8 (s).

HRMS: calculated for $C_{42}H_{38}ClP_2Pd$ $[M-I]^+$ 745.1172, found 745.1174.

Synthesis of bis(triphenylphosphine)palladium(II) 6-chloro-1-hexynide bromide (5i)

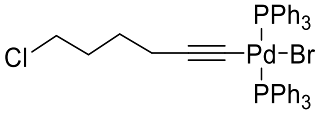

Following *GP4*. Prepared from tetrakis(triphenylphosphine)palladium (580 mg, 0.502 mmol), 1-bromo-6-chlorohexyne (**4i**) (161 mg, 0.824 mmol), benzene (5 mL). Yield: 328 mg (0.397 mmol, 79 %) of grey powder.

Mp: decomposes at 137.0 °C.

IR (cm^{-1}): 3047, 1479, 1435, 1298, 1186, 1095, 1027, 998, 744, 705, 691, 638.

^{1}H NMR (500 MHz, $CDCl_3$) δ = 7.77–7.71 (m, 12H), 7.41–7.36 (m, 18H), 3.07 (t, J = 6.8 Hz, 2H), 1.42 (tt, J = 6.7, 1.9 Hz, 2H), 1.17–1.09 (m, 2H), 0.80–0.71 (m, 2H).

^{13}C{^{1}H} NMR (126 MHz, $CDCl_3$) δ = 135.2 (t, J = 6.4 Hz), 131.9 (t, J = 24.5 Hz), 130.2, 127.9 (t, J = 5.4 Hz), 110.0 (t, J = 7.3 Hz), 83.8 (t, J = 14.3 Hz), 45.0, 31.3, 26.0, 20.2.

^{31}P{^{1}H} NMR (202 MHz, $CDCl_3$) δ = +24.0 (s).

HRMS: calculated for $C_{42}H_{38}ClP_2Pd$ $[M-Br]^+$ 747.1176, found 747.1162.

Synthesis of bis(triphenylphosphine)palladium(II) (cyclohexyl)ethyn-1-ide iodide (5j)

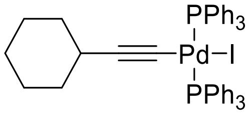

Following *GP4*. Prepared from tetrakis(triphenylphosphine)palladium (574 mg, 0.497 mmol), (iodoethynyl)cyclohexane (**4j**) (175 mg, 0.748 mmol), benzene (7 mL). Yield: 80 mg (0.093 mmol, 19 %) of beige powder.

Mp: 149.5–150.5 °C.

IR (cm^{-1}): 3052, 2923, 2848, 1572, 1480, 1094, 1027, 997, 740, 688, 617.

^{1}H NMR (500 MHz, $CDCl_3$) δ = 7.80–7.72 (m, 12H), 7.41–7.32 (m, 18H), 1.53–1.46 (br, 1H), 1.17–0.98 (br, 3H), 0.90–0.79 (m, 3H), 0.79–0.69 (m, 2H), 0.46–0.34 (m, 2H).

$^{13}C\{^{1}H\}$ NMR (126 MHz, $CDCl_3$) δ = 135.3 (t, J = 6.2 Hz), 133.2 (t, J = 25.3 Hz), 130.1, 127.8 (t, J = 5.4 Hz), 115.1 (t, J = 7.1 Hz), 83.4 (t, J = 13.6 Hz), 32.4, 31.1, 26.1, 24.5.

$^{31}P\{^{1}H\}$ NMR (202 MHz, $CDCl_3$) δ = +23.1 (s).

HRMS: calculated for $C_{44}H_{41}P_2Pd$ $[M-I]^+$ 737.1718, found 737.1724.

Synthesis of bis(triphenylphosphine)palladium(II) (4-formylphenyl)ethyn-1-ide iodide (5k)

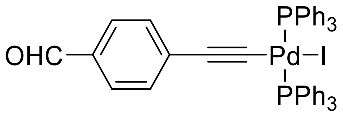

Following *GP4*. Prepared from tetrakis(triphenylphosphine)palladium (578 mg, 0.500 mmol), 1-(iodoethynyl)-4-benzaldehyde (**4j**) (194 mg, 0.758 mmol), benzene (5 mL). Yield: 106 mg (0.119 mmol, 24 %) of yellow powder.

Mp: decomposes at 133.1 °C.

IR (cm^{-1}): 2117, 1700, 1593, 1557, 1480, 1433, 1301, 1206, 1159, 1096, 1029, 998, 828, 742, 704, 690, 613.

^{1}H NMR (500 MHz, $CDCl_3$) δ = 9.77 (s, 1H), 7.80–7.72 (m, 12H), 7.44–7.33 (m, 20H), 6.18 (d, J = 8.2 Hz, 2H).

$^{13}C\{^{1}H\}$ NMR (126 MHz, $CDCl_3$) δ = 191.7, 135.1 (t, J = 6.3 Hz), 133.9, 133.1, 132.5 (t, J = 25.4 Hz), 131.0, 130.4, 128.9, 128.1 (t, J = 5.5 Hz), 122.8, 111.1 (t, J = 13.7 Hz), 109.3 (t, J = 6.7 Hz).

$^{31}P\{^{1}H\}$ NMR (202 MHz, $CDCl_3$) δ = +22.7 (s).

HRMS: calculated for $C_{45}H_{35}OP_2Pd$ $[M-I]^+$ 759.1198, found 759.1209.

Syntheses of bis(triphenylphosphine)palladium(II) bisalkynylides 6

General procedure 5 (*GP5*) – Synthesis of bis(triphenylphosphine)palladium(II) bisacetylides 6 from palladium monoacetylides 5

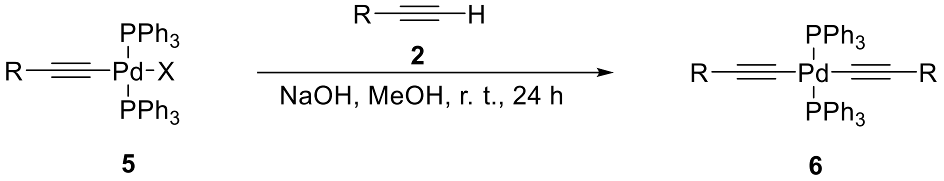

Palladium bisacetylides **6** were prepared according to the modified literature procedure.[17] An oven-dried round-bottom reaction flask was fitted with a stirring bar and closed with a rubber septum, which was pierced with a needle connected by a tube to a Schlenk vacuum manifold. The flask was allowed to cool to room temperature under argon atmosphere. The flask was charged with bis(triphenylphosphine)palladium(II) acetylide halide **5** (1 equiv.) by rapidly opening the septum and flushing with argon. A 0.2 M solution of NaOH in methanol (40 mL/mmol) and alkyne **2** (20 equiv.) were added under argon atmosphere. The reaction mixture was purged with argon for 5 min. Argon was applied by the needle connected with a tube to a Schlenk vacuum manifold. The mixture was stirred overnight in the dark at room temperature under argon atmosphere. The mixture was then filtered and the solid residue was washed with water (20 mL), methanol (20 mL) and diethyl ether (20 mL). The solid residue was dried to give the desired product in pure form.

General procedure 6 (*GP6*) – Synthesis of bis(triphenylphosphine)palladium(II) bisacetylides 6 from bis(triphenylphosphine)palladium(II) dichloride

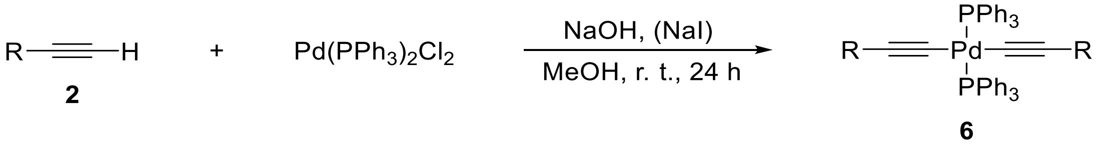

The procedure described below for the preparation of **6** is similar to the literature protocol for the synthesis of palladium bisacetylides with the crucial difference that copper is not used here as a cocatalyst.[18] An oven-dried round-bottom reaction flask was fitted with a stirring bar and closed with a rubber septum, which was pierced with a needle connected by a tube to a Schlenk vacuum manifold. The flask was allowed to cool to room temperature under argon atmospehere. The flask was charged with bis(triphenylphosphine)palladium(II) dichloride (1 equiv.) by rapidly opening the septum and flushing with argon. A 0.5 M solution of NaOH in methanol (30 mL/mmol) and alkyne **2** (20 equiv.) were added under argon atmosphere. The reaction mixture was purged with argon for 5 minutes. The argon was supplied through a needle connected to a Schlenk vacuum manifold. The mixture was stirred overnight in the dark at room temperature. The mixture was then filtered and the solid residue was washed with water (20 mL), methanol (20 mL) and diethyl ether (20 mL). The residue was further dried to afford the desired product in pure form.

For the syntheses of **6f** and **6k**, sodium iodide (4 equiv.) was added to the reaction mixture.

Method A (mimicking regeneration of **B** from **D**)

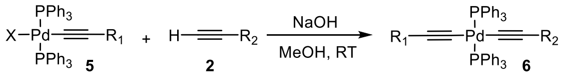

Method B (mimicking formation of **B** from Pd[II])

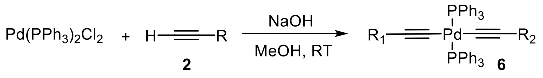

(a) Synthesized palladium bisacetylides *trans*-Pd(PPh$_3$)$_2$(CCAr)$_2$ 6:

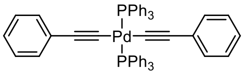

6a

Method A: from **5a**, 80% Method B: 87%
Method A: from **5b**, 95%
Method A: from **5c**, 68%

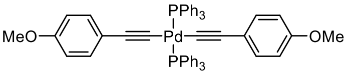

6b

Method B: 53%

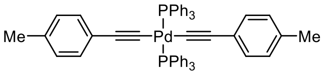

6c

Method B: 50%

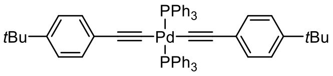

6d

Method B: 35%

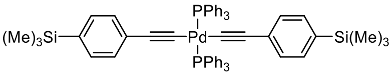

6e

Method B: 27%

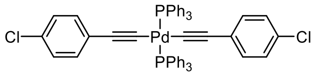

6f

Method B: 87%

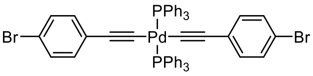

6g

Method B: 40%

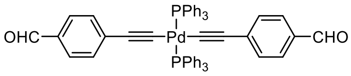

6h

Method A: from **5k**, 73% Method B: 77%

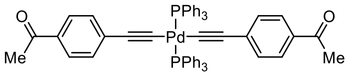

6i

Method B: 91%

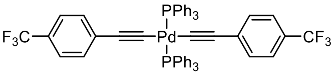

6j

Method B: 77%

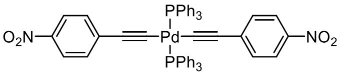

6k

Method A: from **5d**, 69% Method B: 84%

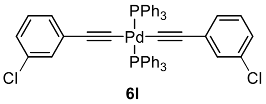

6l

Method A: from **5e**, 28%

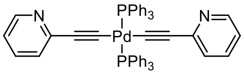

6m

Method A: from **5f**, 75% Method B: 50%
Method A: from **5g**, 76%

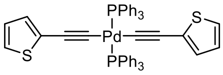

6n

Method B: 47%

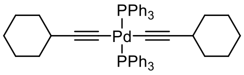

6o

Method A: from **5j**, 93% Method B: 68%

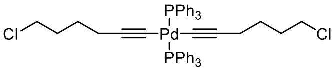

6p

Method A: from **5h**, 38% Method B: 42%
Method A: from **5i**, 77%

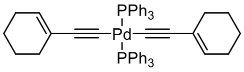

6q

Method B: 81%

(b) Synthesized palladium bisacetylides *trans*-Pd(PPh$_3$)$_2$(CCAr^1)(CCAr^2) 6 with different alkyne subsituents:

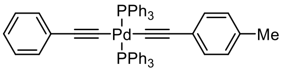

6r

Method A: from **5a**, 86%

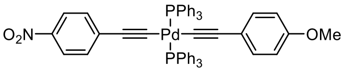

6s

Method A: from **5d**, 93%

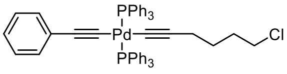

6t

Method A: from **5a**, 76%

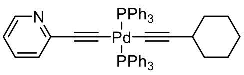

6u

Method A: from **5j**, 81%

Synthesis of bis(triphenylphosphine)palladium(II) bis(phenylethynide) (6a)

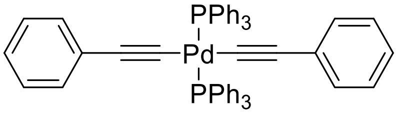

Following *GP5*. Prepared from bis(triphenylphosphine)palladium(II) (phenyl)ethyn-1-ide iodide (**5a**) (86 mg, 0.10 mmol), phenylacetylene (**2a**) (0.220 mL, 2.00 mmol), 0.2 M NaOH solution in methanol (4 mL). Yield: 67 mg (0.080 mmol, 80 %) of grey powder.

Following *GP5*. Prepared from bis(triphenylphosphine)palladium(II) phenylethynide bromide (**5b**) (170 mg, 0.21 mmol), phenylacetylene (**2a**) (0.220 mL, 2.00 mmol), 0.2 M NaOH solution in methanol (8 mL). Yield: 166 mg (0.199 mmol, 95 %) of grey powder.

Following *GP5*. Prepared from bis(triphenylphosphine)palladium(II) phenylethynide chloride (**5c**) (54 mg, 0.070 mmol), phenylacetylene (**2a**) (0.077 mL, 0.70 mmol), 0.2 M NaOH solution in methanol (2 mL). Yield: 40 mg (0.048 mmol, 68 %) of grey powder.

Following *GP6*. Prepared from bis(triphenylphosphine)palladium(II) dichloride (347 mg, 0.495 mmol), phenylacetylene (**2a**) (0.550 mL, 5.00 mmol), 0.5 M NaOH solution in methanol (15 mL). Yield: 359 mg (0.43 mmol, 87 %) of grey powder.

Mp: 137.6–139.3 °C.

^{1}H NMR (500 MHz, $CDCl_3$) δ = 7.90–7.78 (m, 12H), 7.44–7.37 (m, 6H), 7.37–7.29 (m, 12H), 6.95–6.88 (m, 6H), 6.35–6.29 (m, 4H).

^{31}P{^{1}H} NMR (202 MHz, $CDCl_3$) δ = +25.9 (s).

Spectroscopic data are in agreement with literature.[19]

Synthesis of bis(triphenylphosphine)palladium(II) bis((4-methoxyphenyl)ethyn-1-ide) (6b)

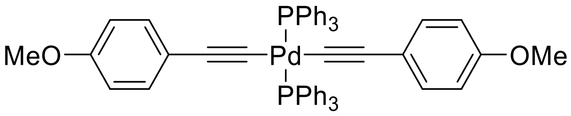

Following *GP6*. Prepared from bis(triphenylphosphine)palladium(II) dichloride (141 mg, 0.201 mmol), 4-ethynylanisole (**2h**) (0.520 mL, 4.00 mmol), 0.5 M NaOH solution in methanol (5 mL). Yield: 96 mg (0.107 mmol, 53 %) of white powder.

Mp: decomposes at 142.1 °C.

IR (cm^{-1}): 3056, 2833, 2105, 1565, 1504, 1480, 1435, 1282, 1242, 1176, 1160, 1097, 1027, 998, 932, 915, 831, 805, 744, 721, 704, 690, 643, 618.

^{1}H NMR (500 MHz, $CDCl_3$) δ = 7.85–7.78 (m, 12H), 7.42–7.37 (m, 6H), 7.37–7.31 (m, 12H), 6.46 (d, J = 8.9 Hz, 4H), 6.23 (d, J = 8.9 Hz, 4H), 3.67 (s, 6H).

^{13}C{^{1}H} NMR (126 MHz, $CDCl_3$) δ = 157.0, 135.0 (t, J = 6.4 Hz), 132.7 (t, J = 24.6 Hz), 131.9, 129.9, 127.8 (t, J = 5.4 Hz), 120.8, 112.7, 55.1. 2 carbon resonances are missing.

^{31}P{^{1}H} NMR (202 MHz, $CDCl_3$) δ = +25.9 (s).

HRMS: calculated for $C_{45}H_{37}OP_2Pd$ [M-(4-ethynylanisole)]$^+$ 761.1354, found 761.1362.

Synthesis of bis(triphenylphosphine)palladium(II) bis((4-methylphenyl)ethyn-1-ide) (6c)

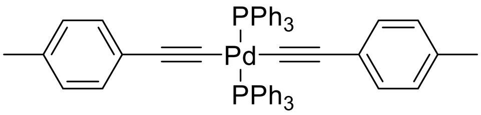

Following *GP6*. Prepared from bis(triphenylphosphine)palladium(II) dichloride (140 mg, 0.199 mmol), 4-ethynyltoluene (**2g**) (0.253 mL, 2.00 mmol), 0.5 M NaOH solution in methanol (6 mL). Yield: 86 mg (0.100 mmol, 50 %) of white powder.

Mp: 136.6–138.4 °C.

IR (cm^{-1}): 2102, 1572, 1504, 1480, 1434, 1212, 1183, 1097, 1027, 999, 817, 742, 703, 689.

^{1}H NMR (500 MHz, $CDCl_3$) δ = 7.86–7.77 (m, 12H), 7.42–7.36 (m, 6H), 7.36–7.30 (m, 12H), 6.72 (d, J = 7.8 Hz, 4H), 6.21 (d, J = 8.0 Hz, 4H), 2.17 (s, 6H).

^{13}C{^{1}H} NMR (126 MHz, $CDCl_3$) δ = 135.2 (t, J = 6.3 Hz), 134.4, 132.7 (t, J = 24.7 Hz), 130.8, 130.0, 128.0 (t, J = 5.4 Hz), 125.3, 114.8 (t, J = 4.0 Hz), 112.4 (t, J = 16.8 Hz), 21.3.

^{31}P{^{1}H} NMR (202 MHz, $CDCl_3$) δ = +25.9 (s).

HRMS: calculated for $C_{45}H_{37}P_2Pd$ [M-(4-ethynyltoluene)]$^+$ 745.1405, found 745.1410.

Synthesis of bis(triphenylphosphine)palladium(II) bis((4-tert-butylphenyl)ethyn-1-ide) (6d)

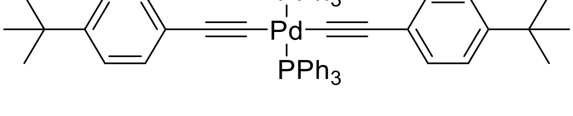

Following *GP6*. Prepared from bis(triphenylphosphine)palladium(II) dichloride (143 mg, 0.204 mmol), 1-ethynyl-(4-*tert*-butyl)benzene (**2i**) (0.360 mL, 2.00 mmol), 0.5 M NaOH solution in methanol (6 mL). Yield: 68 mg (0.072 mmol, 35 %) of white powder.

Mp: 155.4–157.4 °C.

IR (cm^{-1}): 3052, 2958, 2109, 1500, 1478, 1433, 1362, 1267, 1184, 1095, 1027, 999, 838, 797, 742, 705, 690.

^{1}H NMR (500 MHz, $CDCl_3$) δ = 7.86–7.77 (m, 12H), 7.43–7.38 (m, 6H), 7.37–7.30 (m, 12H), 6.93 (d, J = 8.6 Hz, 4H), 6.23 (d, J = 8.6 Hz, 4H), 1.19 (s, 18H).

^{13}C{^{1}H} NMR (126 MHz, $CDCl_3$) δ = 147.6, 135.2 (t, J = 6.4 Hz), 133.0 (t, J = 24.5 Hz), 132.8, 130.0, 128.0 (t, J = 5.5 Hz), 125.4, 124.2, 114.7 (t, J = 4.2 Hz), 112.3 (t, J = 16.7 Hz), 34.5, 31.4.

^{31}P{^{1}H} NMR (202 MHz, $CDCl_3$) δ = +25.9 (s).

HRMS: calculated for $C_{48}H_{43}P_2Pd$ [M-(1-ethynyl-(4-*tert*-butyl)benzene)]$^+$ 787.1875, found 787.1886.

Synthesis of bis(triphenylphosphine)palladium(II) bis((4-(trimethylsilyl)phenyl)ethyn-1-ide) (6e)

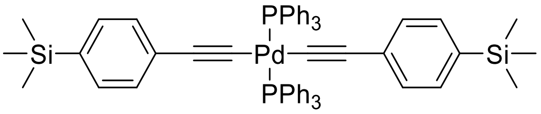

Following *GP6*. Prepared from bis(triphenylphosphine)palladium(II) dichloride (348 mg, 0.496 mmol), 4-(trimethylsilyl)phenylacetylene (**2o**) (0.387 mL, 2.00 mmol), 0.5 M NaOH solution in methanol (6 mL). Yield: 131 mg (0.134 mmol, 27 %) of white powder.

Mp: decomposes at 154.7 °C.

IR (cm^{-1}): 2952, 2113, 1590, 1479, 1434, 1248, 1183, 1107, 1095, 1027, 999, 839, 824, 743, 704, 691, 646.

^{1}H NMR (500 MHz, $CDCl_3$) δ = 7.85–7.75 (m, 12H), 7.44–7.38 (m, 6H), 7.38–7.31 (m, 12H), 7.07 (d, J = 7.8 Hz, 4H), 6.28 (d, J = 7.7 Hz, 4H), 0.16 (s, 18H).

^{13}C{^{1}H} NMR (126 MHz, $CDCl_3$) δ = 136.3, 135.1 (t, J = 6.5 Hz), 132.7 (t, J = 24.5 Hz), 132.3, 130.13, 130.09, 128.7, 128.0 (t, J = 5.5 Hz), 115.0 (t, J = 4.1 Hz), 114.5 (t, J = 16.7 Hz), -1.0.

^{31}P{^{1}H} NMR (202 MHz, $CDCl_3$) δ = +25.9 (s).

HRMS: calculated for $C_{47}H_{43}P_2PdSi$ [M-(4-(trimethylsilyl)phenylacetylene)]$^+$ 803.1644, found 803.1650.

Synthesis of bis(triphenylphosphine)palladium(II) bis((4-chlorophenyl)ethyn-1-ide) (6f)

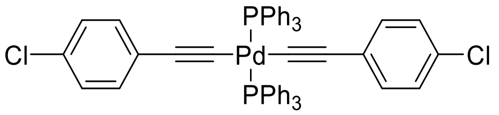

Following *GP6*. Prepared from bis(triphenylphosphine)palladium(II) dichloride (141.5 mg, 0.202 mmol), sodium iodide (131 mg, 0.874 mmol), 1-chloro-4-ethynylbenzene (**2j**) (138 mg, 1.01 mmol), 0.5 M NaOH solution in methanol (4 mL). Yield: 159 mg (0.176 mmol, 87 %) of grey powder.

Mp: 139.7–141.1 °C.

IR (cm^{-1}): 3053, 2106, 1484, 1437, 1207, 1096, 1011, 823, 751, 739, 707, 690, 640.

^{1}H NMR (500 MHz, $CDCl_3$) δ = 7.83–7.73 (m, 12H), 7.44–7.38 (m, 6H), 7.37–7.31 (m, 12H) 6.87 (d, J = 8.5 Hz, 4H), 6.20 (d, J = 8.5 Hz, 4H).

^{13}C{^{1}H} NMR (126 MHz, $CDCl_3$) δ = 135.1 (t, J = 6.4 Hz), 132.4 (t, J = 24.7 Hz), 132.0, 130.5, 130.2, 128.1 (t, J = 5.0 Hz), 127.5, 126.5, 115.4 (t, J = 16.6 Hz), 113.7 (t, J = 3.9 Hz).

^{31}P{^{1}H} NMR (202 MHz, $CDCl_3$) δ = +26.1 (s).

HRMS: calculated for $C_{44}H_{34}ClP_2Pd$ [M-(1-chloro-4-ethynylbenzene)]$^{+}$ 765.0857, found 765.0859.

Synthesis of bis(triphenylphosphine)palladium(II) bis((4-bromophenyl)ethyn-1-ide) (6g)

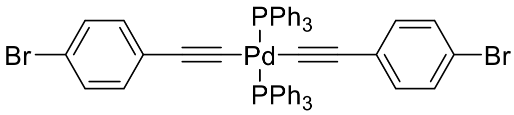

Following *GP6*. Prepared from bis(triphenylphosphine)palladium(II) dichloride (353 mg, 0.503 mmol), 1-bromo-4-ethynylbenzene (**2k**) (899 mg, 4.97 mmol), 0.5 M NaOH solution in methanol (15 mL). Yield: 197 mg (0.199 mmol, 40 %) of orange powder.

Mp: decomposes at 163.1 °C.

IR (cm^{-1}): 3053, 2111, 1480, 1434, 1206, 1094, 1069, 1027, 1008, 820, 740, 705, 687, 607.

^{1}H NMR (500 MHz, $CDCl_3$) δ = 7.82–7.72 (m, 12H), 7.44–7.38 (m, 6H), 7.37–7.31 (m, 12H), 7.02 (d, J = 8.4 Hz, 4H), 6.14 (d, J = 8.4 Hz, 4H).

^{13}C{^{1}H} NMR (126 MHz, $CDCl_3$) δ = 135.1 (t, J = 6.4 Hz), 132.42 (t, J = 24.8 Hz), 132.37, 130.4, 130.2, 128.1 (t, J = 5.4 Hz), 127.0, 118.6, 115.8 (t, J = 16.4 Hz), 113.8 (t, J = 4.1 Hz).

^{31}P{^{1}H} NMR (202 MHz, $CDCl_3$) δ = +26.1 (s).

HRMS: calculated for $C_{44}H_{34}BrP_2Pd$ [M-(1-bromo-4-ethynylbenzene)]$^+$ 811.0333, found 811.0353.

Synthesis of bis(triphenylphosphine)palladium(II) bis((4-formylphenyl)ethyn-1-ide) (6h)

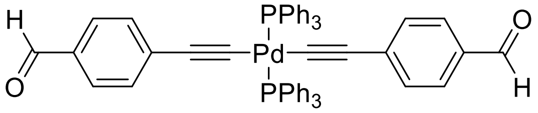

Following *GP5*. Prepared from bis(triphenylphosphine)palladium(II) (4-formlyphenyl)ethyn-1-ide iodide (**5k**) (57 mg, 0.063 mmol), 4-ethynylbenzaldehyde (**2m**) (163 mg, 1.25 mmol), 0.2 M NaOH solution in methanol (3 mL). Yield: 40.5 mg (0.046 mmol, 73 %) of orange powder.

Following *GP6*. Prepared from bis(triphenylphosphine)palladium(II) dichloride (280 mg, 0.399 mmol), 4-ethynylbenzaldehyde (**2m**) (522 mg, 4.01 mmol), 0.5 M NaOH solution in methanol (10 mL). Yield: 275 mg (0.309 mmol, 77 %) of orange powder.

Mp: decomposes at 140.5 °C.

IR (cm^{-1}): 3047, 2729, 2106, 1688, 1591, 1556,1481, 1435, 1385, 1301, 1208, 1162, 1096, 1027, 998, 829, 753, 741, 707, 689, 609.

^{1}H NMR (500 MHz, $CDCl_3$) δ = 9.80 (s, 1H), 7.81–7.75 (m, 12H), 7.46–7.39 (m, 10H), 7.38–7.33 (m, 12H), 6.40 (d, J = 8.2 Hz, 4H).

^{13}C{^{1}H} NMR (126 MHz, $CDCl_3$) δ = 191.8, 135.0 (t, J = 6.4 Hz), 134.6, 132.9, 132.1 (t, J = 25.3 Hz), 131.1, 130.4, 129.0, 128.2 (t, J = 5.4 Hz), 122.6 (t, J = 16.8 Hz), 115.1 (t, J = 4.0 Hz).

^{31}P{^{1}H} NMR (202 MHz, $CDCl_3$) δ = +26.2 (s).

HRMS: calculated for $C_{45}H_{35}OP_2Pd$ [M-(4-ethynylbenzaldehyde)]$^+$ 759.1198, found 759.1208.

Synthesis of bis(triphenylphosphine)palladium(II) bis((4-acetylphenyl)ethyn-1-ide) (6i)

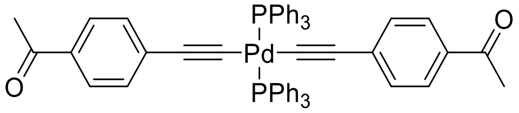

Following *GP6*. Prepared from bis(triphenylphosphine)palladium(II) dichloride (144 mg, 0.205 mmol), 4'-ethynylacetophenone (**2n**) (288 mg, 2.00 mmol), 0.5 M NaOH solution in methanol (6 mL). Yield: 171 mg (0.186 mmol, 91 %) of white powder.

Mp: decomposes at 163.4 °C.

IR (cm^{-1}): 2105, 1672, 1589, 1548, 1481, 1434, 1403, 1355, 1301, 1282, 1266, 1214, 1174, 1097, 1027, 998, 951, 851, 829, 757, 745, 706, 694.

^{1}H NMR (500 MHz, $CDCl_3$) δ = 7.82–7.75 (m, 12H), 7.53 (d, J = 8.4 Hz, 4H), 7.45–7.39 (m, 6H), 7.38–7.32 (m, 12H), 6.35 (d, J = 8.4 Hz, 4H), 2.47 (s, 6H).

$^{13}C\{^{1}H\}$ NMR (126 MHz, $CDCl_3$) δ = 197.7, 135.1 (t, J = 6.3 Hz), 133.4, 133.2, 132.2 (t, J = 25.2 Hz), 130.8, 130.4, 128.1 (t, J = 5.2 Hz), 127.6, 120.9, 114.9, 26.5.

$^{31}P\{^{1}H\}$ NMR (202 MHz, $CDCl_3$) δ = +26.2 (s).

HRMS: calculated for $C_{46}H_{37}OP_2Pd$ [M-(4'-ethynylacetophenone)]$^+$ 773.1354, found 773.1363.

Synthesis of bis(triphenylphosphine)palladium(II) bis((4-(trifluoromethyl)phenyl)ethyn-1-ide) (6j)

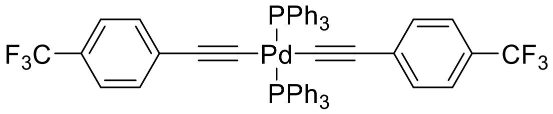

Following *GP6*. Prepared from bis(triphenylphosphine)palladium(II) dichloride (349 mg, 0.498 mmol), 1-(trifluoromethyl)-4-ethynylbenzene (**2l**) (0.816 mL, 5.00 mmol), 0.5 M NaOH solution in methanol (10 mL). Yield: 374 mg (0.386 mmol, 77 %) of yellow powder.

Mp: 129.8–130.5 °C.

IR (cm^{-1}): 3044, 2105, 1606, 1481, 1435, 1319, 1213, 1179, 1159, 1110, 1099, 1062, 1015, 999, 839, 139, 707, 689, 604.

^{1}H NMR (500 MHz, $CDCl_3$) δ = 7.84–7.76 (m, 12H), 7.45–7.40 (m, 6H), 7.40–7.32 (m, 12H), 7.16 (d, J = 8.3 Hz, 4H), 6.36 (d, J = 8.3 Hz, 4H).

$^{13}C\{^{1}H\}$ NMR (126 MHz, $CDCl_3$) δ = 135.0 (t, J = 6.4 Hz), 132.3 (t, J = 25.0 Hz), 131.6, 130.8, 130.3, 128.1 (t, J = 5.1 Hz), 126.5 (q, J = 31.9 Hz), 124.6 (q, J = 271.8 Hz), 124.2 (q, J = 3.8 Hz), 123.5, 118.3 (t, J = 16.4 Hz), 113.9 (t, J = 3.6 Hz).

$^{31}P\{^{1}H\}$ NMR (202 MHz, $CDCl_3$) δ = +26.1 (s).

^{19}F NMR (470 MHz, $CDCl_3$) δ = –62.3 (s).

HRMS: calculated for $C_{45}H_{34}F_3P_2Pd$ [M-(1-(trifluoromethyl)-4-ethynylbenzene)]$^+$ 799.1123, found 799.1128.

Synthesis of bis(triphenylphosphine)palladium(II) bis((4-nitrophenyl)ethyn-1-ide) (6k)

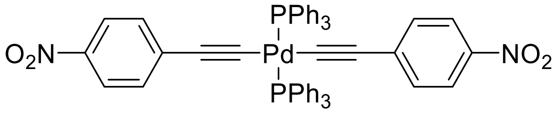

Following *GP5*. Prepared from bis(triphenylphosphine)palladium(II) (4-nitrophenyl)ethyn-1-ide iodide (**5d**) (68 mg, 0.075 mmol), 1-nitro-4-ethynylbenzene (**2b**) (110 mg, 0.75 mmol), 0.2 M NaOH solution in methanol (2 mL). Yield: 48 mg (0.052 mmol, 69 %) of yellow powder.

Following *GP6*. Prepared from bis(triphenylphosphine)palladium(II) dichloride (145 mg, 0.207 mmol), sodium iodide (138 mg, 0.920 mmol), 1-ethynyl-4-nitrobenzene (**2b**) (154 mg, 1.05 mmol), 0.5 M NaOH solution in methanol (4 mL). Yield: 161 mg (0.174 mmol, 84 %) of yellow powder.

Mp: decomposes at 152.4 °C.

IR (cm^{-1}): 3047, 2107, 1586, 1509, 1484, 1435, 1336, 1215, 1107, 1096, 998, 852, 751, 741, 707, 689, 626.

^{1}H NMR (500 MHz, $CDCl_3$) δ = 7.87–7.69 (m, 16H), 7.49–7.40 (m, 6H), 7.40–7.32 (m, 12H), 6.34 (d, J = 8.5 Hz, 4H).

^{13}C{^{1}H} NMR (126 MHz, $CDCl_3$) δ = 144.6, 135.0 (t, J = 6.3 Hz), 132.0 (t, J = 24.9 Hz), 131.0, 130.6, 128.2 (t, J = 5.1 Hz), 124.9 (t, J = 16.4 Hz), 122.9, 114.4 (t, J = 4.1 Hz).

^{31}P{^{1}H} NMR (202 MHz, $CDCl_3$) δ = +26.4 (s).

HRMS: calculated for $C_{44}H_{34}NO_2P_2Pd$ [M-(1-nitro-4-ethynylbenzene)]$^+$ 776.1100, found 776.1107.

Synthesis of bis(triphenylphosphine)palladium(II) bis((3-chlorophenyl)ethyn-1-ide) (6l)

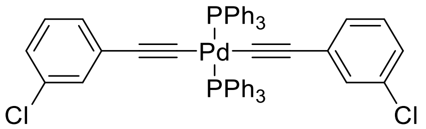

Following *GP5*. Prepared from bis(triphenylphosphine)palladium(II) (3-chlorophenyl)ethyn-1-ide iodide (**5e**) (142 mg, 0.159 mmol), 1-chloro-3-ethynylbenzene (**2c**) (0.400 mL, 3.25 mmol), 0.2 M NaOH solution in methanol (5 mL). Yield: 40 mg (0.044 mmol, 28 %) of yellow powder.

Mp: 115.9–116.9 °C.

IR (cm^{-1}): 3057, 2110, 1586, 1470, 1432, 1091, 860, 778, 741, 703, 683, 617.

^{1}H NMR (300 MHz, $CDCl_3$) δ = 7.84–7.75 (m, 12H), 7.47–7.32 (m, 18H), 6.89 (ddd, J = 8.0, 2.1, 1.5 Hz, 2H), 6.83 (t, J = 7.7 Hz, 2H), 6.21 (dt, J = 7.4, 1.5 Hz, 2H), 6.18 (t, J = 1.6 Hz, 2H).

^{13}C{^{1}H} NMR (76 MHz, $CDCl_3$) δ = 135.1 (t, J = 6.3 Hz), 133.0, 132.4 (t, J = 24.9 Hz), 131.0, 130.3, 129.7, 128.7, 128.4, 128.1 (t, J = 5.3 Hz), 125.1, 116.3 (t, J = 16.7 Hz), 113.6 (t, J = 4.0 Hz).

^{31}P{^{1}H} NMR (122 MHz, $CDCl_3$) δ = +26.2 (s).

HRMS: calculated for $C_{44}H_{34}ClP_2Pd$ [M-(1-chloro-3-ethynylbenzene)]$^+$ 765.0858, found 765.0859.

Synthesis of bis(triphenylphosphine)palladium(II) bis((2-pyridyl)ethyn-1-ide) (6m)

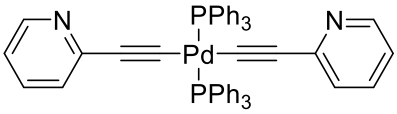

Following *GP5*. Prepared from bis(triphenylphosphine)palladium(II) (2-pyridyl)ethyn-1-ide bromide (**5f**) (82 mg, 0.101 mmol), 2-ethynylpyridine (**2d**) (0.202 mL, 2.00 mmol), 0.2 M NaOH solution in methanol (4 mL). Yield: 62 mg (0.074 mmol, 75 %) of beige powder.

Following *GP5*. Prepared from bis(triphenylphosphine)palladium(II) (2-pyridyl)ethyn-1-ide chloride (**5g**) (232 mg, 0.301 mmol), 2-ethynylpyridine (**2d**) (0.300 mL, 2.97 mmol), 0.2 M NaOH solution in methanol (8 mL). Yield: 191 mg (0.229 mmol, 76 %) of beige powder.

Following *GP6*. Prepared from bis(triphenylphosphine)palladium(II) dichloride (140 mg, 0.199 mmol), 2-ethynylpyridine (**2d**) (0.202 mL, 2.00 mmol), 0.5 M NaOH solution in methanol (6 mL). Yield: 86 mg (0.100 mmol, 50 %) of beige powder.

Mp: 123.1–128.8 °C.

IR (cm^{-1}): 3045, 2110, 1580, 1554, 1479, 1457, 1432, 1421, 1237, 1183, 1147, 1092, 1056, 998, 987, 780, 747, 691, 630.

^1H NMR (500 MHz, $CDCl_3$) δ = 8.23 (dd, J = 4.9, 1.0 Hz, 2H), 7.85–7.76 (m, 12H), 7.34–7.27 (m, 18H), 7.15 (dt, J = 7.7, 1.8 Hz, 2H), 6.79 (ddd, J = 4.8, 2.5, 1.1 Hz, 2H), 6.13 (d, J = 7.9 Hz, 2H).

$^{13}C\{^1H\}$ NMR (126 MHz, $CDCl_3$) δ = 148.7, 146.6, 135.2 (t, J = 6.4 Hz), 134.6, 132.2 (t, J = 24.9 Hz), 130.2, 128.0 (t, J = 5.2 Hz), 125.9, 119.6, 117.8 (t, J = 16.4 Hz), 115.5 (t, J = 3.8 Hz).

$^{31}P\{^1H\}$ NMR (202 MHz, $CDCl_3$) δ = +25.1 (s).

HRMS: calculated for $C_{50}H_{40}N_2P_2Pd$ $[M+2H]^{2+}$ 418.0845, found 418.0850.

Synthesis of bis(triphenylphosphine)palladium(II) bis((2-thiophenyl)ethyn-1-ide) (6n)

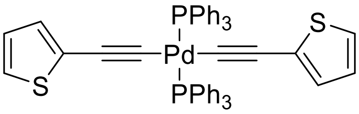

Following *GP6*. Prepared from bis(triphenylphosphine)palladium(II) dichloride (353 mg, 0.502 mmol), 2-ethynylthiophene (**2p**) (0.498 mL, 5.24 mmol), 0.5 M NaOH solution in methanol (15 mL). Yield: 201 mg (0.238 mmol, 47 %) of brown powder.

Mp: 161.3–165.4 °C.

IR (cm^{-1}): 3070, 3052, 2103, 1510, 1478, 1433, 1133, 1096, 847, 821, 745, 688.

^{1}H NMR (500 MHz, $CDCl_3$) δ = 7.87–7.70 (m, 12H), 7.46–7.32 (m, 18H), 6.76 (dd, J = 5.1, 0.8 Hz, 2H), 6.61 (dd, J = 5.1, 3.6 Hz, 2H), 6.02 (d, J = 3.5 Hz, 2H).

^{13}C{^{1}H} NMR (126 MHz, $CDCl_3$) δ = 135.0 (t, J = 6.4 Hz), 132.4 (t, J = 25.0 Hz), 130.2, 129.0, 128.1 (t, J = 5.4 Hz), 127.9, 125.9, 122.8, 119.0 (t, J = 16.6 Hz), 106.5.

^{31}P{^{1}H} NMR (202 MHz, $CDCl_3$) δ = +25.4 (s).

HRMS: calculated for $C_{42}H_{33}P_2PdS$ [M-(2-ethynylthiophene)]$^+$ 735.0801, found 735.0750.

Synthesis of bis(triphenylphosphine)palladium(II) bis((cyclohexyl)ethyn-1-ide) (6o)

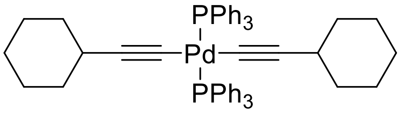

Following *GP5*. Prepared from bis(triphenylphosphine)palladium(II) (cyclohexyl)ethyn-1-ide iodide (**5j**) (175 mg, 0.202 mmol), ethynylcyclohexane (**2f**) (0.270 mL, 2.07 mmol), 0.2 M NaOH solution in methanol (8 mL). Yield: 158 mg (0.187 mmol, 93 %) of white powder.

Following *GP6*. Prepared from bis(triphenylphosphine)palladium(II) dichloride (140 mg, 0.199 mmol), ethynylcyclohexane (**2f**) (0.261 mL, 2.00 mmol), 0.5 M NaOH solution in methanol (6 mL). Yield: 114 mg (0.135 mmol, 68 %) of white powder.

Mp: 157.2–160.8 °C.

IR (cm^{-1}): 3053, 2106, 1484, 1437, 1207, 1096, 1011, 823, 751, 739, 707, 690, 640.

^{1}H NMR (500 MHz, $CDCl_3$) δ = 7.80–7.74 (m, 12H), 7.39–7.34 (m, 6H), 7.34–7.28 (m, 12H), 1.74–1.63 (br, 2H), 1.29–1.15 (m, 6H), 1.09–1.00 (m, 4H), 0.97–0.79 (m, 6H), 0.67–0.57 (m, 4H).

^{13}C{^{1}H} NMR (126 MHz, $CDCl_3$) δ = 135.4 (t, J = 6.5 Hz), 133.4 (t, J = 24.3 Hz), 129.7, 127.6 (t, J = 5.4 Hz), 119.5 (t, J = 4.2 Hz), 96.7 (t, J = 16.3 Hz), 33.3, 31.8, 26.3, 25.0.

^{31}P{^{1}H} NMR (202 MHz, $CDCl_3$) δ = +25.4 (s).

HRMS: calculated for $C_{44}H_{41}P_2Pd$ [M-(ethynylcyclohexane)]$^+$ 733.1734, found 733.1722.

Synthesis of bis(triphenylphosphine)palladium(II) bis(6-chlorohexyn-1-ide) (6p)

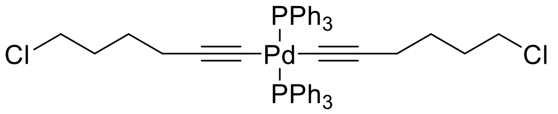

Following *GP5*. Prepared from bis(triphenylphosphine)palladium(II) (6-chloro-hexyn-1-ide) iodide (**5h**) (108 mg, 0.12 mmol), 6-chlorohex-1-yne (**2e**) (0.170 mL, 1.40 mmol), 0.2 M NaOH solution in methanol (5 mL). Yield: 40 mg (0.046 mmol, 38 %) of white powder.

Following *GP5*. Prepared from bis(triphenylphosphine)palladium(II) (6-chloro-hexyn-1-ide) bromide (**5i**) (84 mg, 0.102 mmol), 6-chlorohex-1-yne (**2e**) (0.242 mL, 2.00 mmol), 0.2 M NaOH solution in methanol (4 mL). Yield: 68 mg (0.079 mmol, 77 %) of white powder.

Following *GP6*. Prepared from bis(triphenylphosphine)palladium(II) dichloride (140 mg, 0.199 mmol), 6-chlorohex-1-yne (**2e**) (0.241 mL, 1.99 mmol), 0.5 M NaOH solution in methanol (6 mL). Yield: 72 mg (0.084 mmol, 42 %) of white powder.

Mp: 105.4–108.8 °C.

IR (cm^{-1}): 3047, 2122, 1480, 1432, 1314, 1298, 1186, 1094, 1027, 998, 748, 693, 638.

^{1}H NMR (500 MHz, $CDCl_3$) δ = 7.81–7.71 (m, 12H), 7.42–7.30 (m, 18H), 3.13 (t, J = 6.8 Hz, 4H), 1.55 (t, J = 6.6, 4H), 1.23 (quint, J = 7.4, 4H), 0.90 (quint, J = 7.4 Hz, 4H).

^{13}C{^{1}H} NMR (126 MHz, $CDCl_3$) δ = 135.3 (t, J = 6.4 Hz), 133.1 (t, J = 24.3 Hz), 129.9, 127.7 (t, J = 5.4 Hz), 113.3 (t, J = 4.1 Hz), 98.9 (t, J = 17.2 Hz), 45.2, 31.4, 26.5, 20.5.

^{31}P{^{1}H} NMR (202 MHz, $CDCl_3$) δ = +25.8 (s).

HRMS: calculated for $C_{42}H_{38}ClP_2Pd$ [M-(6-chlorohex-1-yne)]$^+$ 741.1188, found 743.1173.

Synthesis of bis(triphenylphosphine)palladium(II) bis((cyclohex-1-enyl)ethyn-1-ide) (6q)

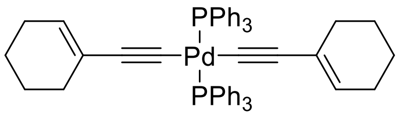

Following *GP6*. Prepared from bis(triphenylphosphine)palladium(II) dichloride (142 mg, 0.202 mmol), 1-ethynylcyclohexene (**2q**) (0.253 mL, 2.00 mmol), 0.5 M NaOH solution in methanol (6 mL). Yield: 138 mg (0.164 mmol, 81 %) of white powder.

Mp: 142.1–143.7 °C.

IR (cm^{-1}): 3052, 2927, 2831, 2211, 2178, 2094, 1959, 1479, 1433, 1092, 913, 836, 741, 703, 686, 618.

^{1}H NMR (500 MHz, $CDCl_3$) δ = 7.80–7.70 (m, 12H), 7.40–7.29 (m, 18H), 4.83 (quint, J = 1.7 Hz, 2H), 1.80–1.72 (m, 4H), 1.34–1.17 (m, 4H).

$^{13}C\{^{1}H\}$ NMR (126 MHz, $CDCl_3$) δ = 135.2 (t, J = 6.4 Hz), 133.1 (t, J = 24.5 Hz), 129.8, 127.8 (t, J = 5.1 Hz), 124.4, 117.0 (t, J = 4.1 Hz), 108.6 (t, J = 17.2 Hz), 29.5, 25.4, 22.6, 22.0.

$^{31}P\{^{1}H\}$ NMR (202 MHz, $CDCl_3$) δ = +25.3 (s).

HRMS: calculated for $C_{42}H_{38}ClP_2Pd$ [M-(1-ethynylcyclohexene)]$^+$ 731.1578, found 731.1563.

Synthesis of bis(triphenylphosphine)palladium(II) (4-methylphenyl)ethyn-1-ide (phenyl)ethyn-1-ide (6r)

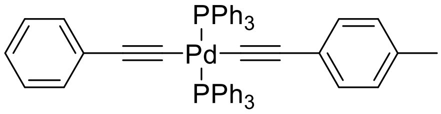

Following *GP5*. Prepared from bis(triphenylphosphine)palladium(II) (phenyl)ethyn-1-ide iodide (**5a**) (87 mg, 0.101 mmol), 4-ethynyltoluene (**2g**) (0.127 mL, 1.00 mmol), 0.2 M NaOH solution in methanol (4 mL). Yield: 74 mg (0.087 mmol, 86 %) of white powder.

Mp: decomposes 122.7 °C.

IR (cm^{-1}): 3046, 2104, 1594, 1572, 1505, 1481, 1435, 1206, 1187, 1095, 1027, 998, 814, 750, 740, 705, 687.

^{1}H NMR (500 MHz, $CDCl_3$) δ = 7.86–7.77 (m, 12H), 7.43–7.37 (m, 6H), 7.37–7.30 (m, 12H), 6.95–6.88 (m, 3H), 6.73 (d, J = 7.9 Hz, 2H), 6.34–6.29 (m, 2H), 6.22 (d, J = 8.0 Hz, 2H), 2.18 (s, 3H).

$^{13}C\{^{1}H\}$ NMR (126 MHz, $CDCl_3$) δ = 135.1 (t, J = 6.4 Hz), 134.5, 132.7 (t, J = 24.6 Hz), 130.9, 130.8, 130.1, 128.0 (t, J = 5.4 Hz), 125.2, 124.8, 114.88, 114.84, 113.9 (t, J = 16.8 Hz), 112.3 (t, J = 16.9 Hz), 21.3.

$^{31}P\{^{1}H\}$ NMR (202 MHz, $CDCl_3$) δ = +25.9 (s).

HRMS: calculated for $C_{44}H_{35}P_2Pd$ [M-((4-tolyl)acetylene)]$^+$ 731.1249, found 731.1243.

Synthesis of bis(triphenylphosphine)palladium(II) (4-methoxyphenyl)ethyn-1-ide (4-nitrophenyl)ethyn-1-ide (6s)

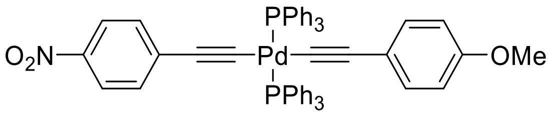

Following *GP5*. Prepared from bis(triphenylphosphine)palladium(II) (4-nitrophenyl)ethyn-1-ide iodide (**5d**) (279 mg, 0.309 mmol), 4-ethynylanisole (**2h**) (0.401 mL, 3.09 mmol), 0.2 M NaOH solution in methanol (9 mL). Yield: 260 mg (0.286 mmol, 93 %) of brownish yellow powder.

Mp: decomposes 123.7 °C.

IR (cm^{-1}): 3055, 2099, 1583, 1504, 1480, 1434, 1332, 1282, 1240, 1207, 1160, 1096, 1027, 998, 855, 833, 750, 741, 704, 689, 626.

^{1}H NMR (500 MHz, $CDCl_3$) δ = 7.83–7.75 (m, 14H), 7.44–7.39 (m, 6H), 7.39–7.33 (m, 12H), 6.47 (d, J = 8.7 Hz, 2H), 6.33 (d, J = 8.8 Hz, 2H), 6.24 (d, J = 8.7 Hz, 2H), 3.68 (s, 3H).

^{13}C{^{1}H} NMR (126 MHz, $CDCl_3$) δ = 157.3, 144.4, 135.1 (t, J = 6.5 Hz), 132.3 (t, J = 24.9 Hz), 132.0, 131.1, 130.3, 128.1 (t, J = 5.5 Hz), 127.3 (t, J = 16.5 Hz), 122.9, 120.6, 115.1 (t, J = 4.2 Hz), 113.9 (t, J = 4.0 Hz), 112.9, 112.8, 109.2 (t, J = 16.4 Hz), 55.2.

^{31}P{^{1}H} NMR (202 MHz, $CDCl_3$) δ = +26.1 (s).

HRMS: calculated for $C_{44}H_{34}NO_2P_2Pd$ [M-((4-ethynyl)anisole)]$^+$ 776.1100, found 776.1105.

Synthesis of bis(triphenylphosphine)palladium(II) (6-chlorobutyl)ethyn-1-ide (phenyl)ethyn-1-ide (6t)

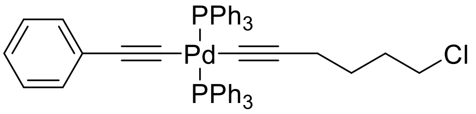

Following *GP5*. Prepared from bis(triphenylphosphine)palladium(II) (phenyl)ethyn-1-ide iodide (**5a**) (115 mg, 0.134 mmol), 6-chloro-1-hexyne (**2e**) (0.163 mL, 1.34 mmol), 0.2 M NaOH solution in methanol (5 mL). Yield: 86 mg (0.101 mmol, 76 %) of brownish-grey powder.

Mp: 105.8–107.7 °C.

IR (cm^{-1}): 3052, 2106, 1593, 1572, 1481, 1433, 1093, 1028, 742, 704, 687.

^{1}H NMR (500 MHz, $CDCl_3$) δ = 7.86–7.71 (m, 12H), 7.46–7.29 (m, 18H), 6.96–6.85 (m, 3H), 6.35–6.26 (m, 2H), 3.15 (t, J = 6.7 Hz, 2H), 1.59 (t, J = 6.7 Hz, 2H), 1.32–1.20 (m, 2H), 0.99–0.86 (m, 2H).

^{13}C{^{1}H} NMR (126 MHz, $CDCl_3$) δ = 135.2 (t, J = 6.6 Hz), 132.9 (t, J = 24.8 Hz), 130.9, 130.0, 128.3, 127.9 (t, J = 5.4 Hz), 127.1, 124.7, 124.7, 114.6, 114.5, 113.8 (t, J = 4.5 Hz), 98.1 (t, J = 17.0 Hz), 45.2, 31.4, 26.5, 20.5.

^{31}P{^{1}H} NMR (202 MHz, $CDCl_3$) δ = +25.9 (s).

HRMS: calculated for $C_{44}H_{35}P_2Pd$ [M-(6-chlorohexyne)]$^+$ 731.1247, found 731.1243.

Synthesis of bis(triphenylphosphine)palladium(II) (2-pyridyl)ethyn-1-ide (cyclohexyl)ethyn-1-ide (6u)

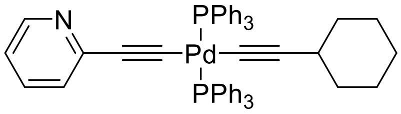

Following *GP5*. Prepared from bis(triphenylphosphine)palladium(II) (cyclohexyl)ethyn-1-ide iodide (**5j**) (62 mg, 0.072 mmol), 2-ethynylpyridine (**2d**) (0.073 mL, 0.723 mmol), 0.2 M NaOH solution in methanol (3 mL). Yield: 49 mg (0.058 mmol, 81 %) of white powder.

Mp: decomposes at 145.0 °C.

IR (cm^{-1}): 3050, 2921, 2848, 2112, 1579, 1554, 1480, 1458, 1434, 1419, 1236, 1093, 1028, 776, 743, 704, 688.

^{1}H NMR (500 MHz, $CDCl_3$) δ = 8.25 (d, *J* = 4.1 Hz, 1H), 7.86–7.71 (m, 12H), 7.41–7.27 (m, 18H), 7.18 (dt, *J* = 7.7, 1.8 Hz, 1H), 6.79 (ddd, *J* = 7.4, 5.1, 0.8 Hz, 1H), 6.25 (d, *J* = 7.9 Hz, 1H), 1.70 (s, 1H), 1.23–1.10 (m, 3H), 1.06–0.96 (m, 2H), 0.95–0.87 (m, 1H), 0.87–0.75 (m, 2H), 0.64–0.51 (m, 2H).

^{13}C{^{1}H} NMR (126 MHz, $CDCl_3$) δ = 148.7, 146.7, 135.3 (t, *J* = 6.4 Hz), 134.6, 132.7 (t, *J* = 24.6 Hz), 129.9, 127.8 (t, *J* = 5.3 Hz), 125.7, 121.4, 120.8, 119.3, 114.4 (t, *J* = 3.7 Hz), 94.0 (t, *J* = 16.4 Hz), 33.0, 31.4, 26.3, 24.7.

^{31}P{^{1}H} NMR (202 MHz, $CDCl_3$) δ = +25.4 (s).

HRMS: calculated for $C_{51}H_{45}NP_2Pd$ $[M]^+$ 840.2096, found 840.2143.

Syntheses of bis(triphenylphosphine)palladium(II) aryl halides 7

General procedure 7 (*GP7*) – Synthesis of bis(triphenylphosphine)palladium(II) aryl iodides 7 from aryl iodides 1

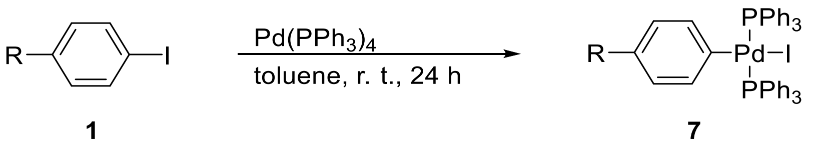

Bis(triphenylphosphine)palladium(II) aryl iodides **7** were prepared according to the modified literature procedure.[20] An oven-dired round-bottom reaction flask was fitted with a stirring bar and closed with a rubber septum, which was pierced with a needle connected by a tube to a Schlenk vacuum manifold. The flask was cooled to room temperature under argon atmosphere. The flask was charged with tetrakis(triphenylphosphine)palladium(0) (1 equiv.) by quickly opening the septum and flushing with argon. Toluene (20 mL/mmol) was added with a syringe by piercing the septum followed by addition of aryl iodide 1 (2 equiv.) that was added by rapidly opening the septum and flushing with argon. The reaction mixture was purged with argon for 5 min. Argon was applied by the needle connected to a Schlenk vacuum manifold. The mixture was stirred overnight in the dark at room temperature under argon atmosphere. The mixture was then filtered and the solid residue was washed with methanol (20 mL) and diethyl ether (20 mL) to give the desired product.

General procedure 8 (*GP8*) – Synthesis of bis(triphenylphosphine)palladium(II) aryl bromides 7 from aryl bromides 1

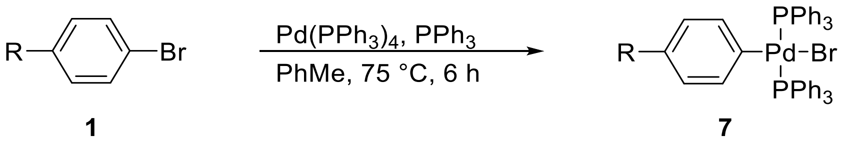

Bis(triphenylphosphine)palladium(II) bromides **7** were prepared according to the modified literature procedure.[21] An oven-dried Ace reaction tube was fitted with a stirring bar and cooled to room temperature under argon atmosphere. The tube was charged with tetrakis(triphenylphosphine)palladium(0) (1 equiv.) by quickly opening the cap and flushing with argon. Toluene (20 mL/mmol), aryl bromide 1 (10 equiv.) and triphenylphosphine (2 equiv.) were added by rapidly opening the tube and flushing with argon. The reaction mixture was purged with argon for 5 min. The argon was supplied through a needle connected to a Schlenk vacuum manifold. The reaction mixture was heated to 75 °C and stirred under argon atmosphere. After 6 hours, the mixture was cooled to room temperature and filtered. The solid residue was washed with methanol (20 mL) and diethyl ether (20 mL) to give the desired product.

General procedure 9 (*GP9*) – Synthesis of bis(triphenylphosphine)palladium(II) aryl chlorides 7 from bis(triphenylphosphine)palladium(II) aryl iodides 7

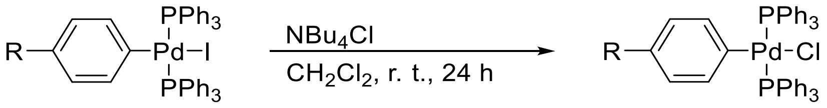

7 **7**

Bis(triphenylphosphine)palladium(II) chlorides **7** were prepared according to the modified literature procedure.[22] An oven-dried round-bottom reaction flask was fitted with a stirring bar and closed with a rubber septum, which was pierced with a needle connected by a tube to a Schlenk vacuum manifold. The flask was cooled to room temperature under an argon atmosphere and charged with bis(triphenylphosphine)palladium(II) aryl iodide **7** (1 equiv.) by rapidly opening the septum and flushing with argon. Dichloromethane (10 mL/mmol) was added with a syringe by piercing the septum under argon atmosphere. The solution was cooled to 0 °C on an ice bath. A solution of tetrabutylammonium chloride (10 equiv.) in dichloromethane (10 mL/mmol) was then slowly added with a syringe by piercing the septum. The reaction was allowed to warm to room temperature and stirred overnight under an argon atmosphere. The solvent was evaporated with the aid of rotary evaporator and the crude product was suspended in methanol (10 ml) and filtered. The solid residue was washed with diethyl ether (20 mL) to give the desired product.

(a) Synthesized palladium addition complexes 7:

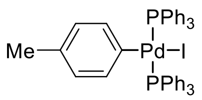

7a (55%)

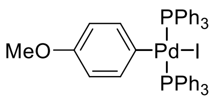

7b (64%)

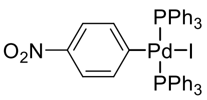

7c (46%)

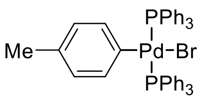

7d (61%)

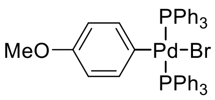

7e (46%)

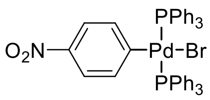

7f (51%)

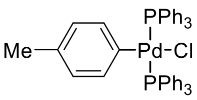

7g
from **7a**: 77%

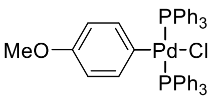

7h
from **7b**: 94%

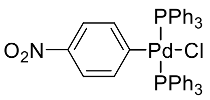

7i
from **7c**: 96%

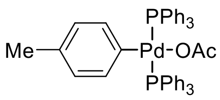

7j
from **7g**: 49%

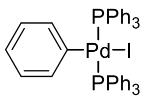

7k (64%)

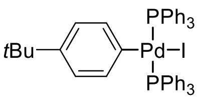

7l (72%)

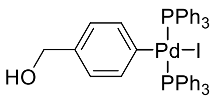

7m (35%)

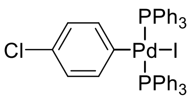

7n (72%)

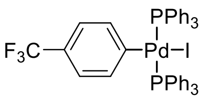

7o (46%)

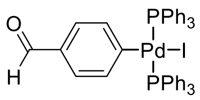

7p (57%)

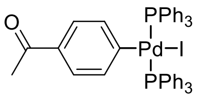

7q (63%)

(b) Other synthesized palladium complexes:

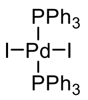

96%

Synthesis of bis(triphenylphosphine)palladium(II) (4-methylphenyl)ide iodide (7a)

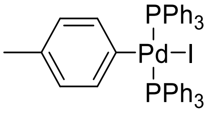

Following *GP7*. Prepared from tetrakis(triphenylphosphine)palladium(0) (591 mg, 0.51 mmol), 4-iodotoluene (**1a**) (233 mg, 1.07 mmol), toluene (10 mL). Yield: 239 mg (0.28 mmol, 55 %) of white powder.

Mp: 166.4–166.7 °C.

^{1}H NMR (500 MHz, $CDCl_3$) δ = 7.53–7.47 (m, 12H), 7.37–7.28 (m, 6H), 7.25–7.19 (m, 12H), 6.41 (dt, J = 8.1, 2.1 Hz, 2H), 6.07 (d, J = 7.4 Hz, 2H), 1.91 (s, 3H).

^{31}P{^{1}H} NMR (202 MHz, $CDCl_3$) δ = +22.5 (s).

Spectroscopic data are in agreement with literature.[23]

Synthesis of bis(triphenylphosphine)palladium(II) (4-methoxyphenyl)ide iodide (7b)

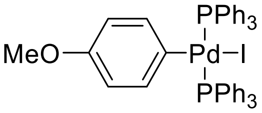

Following *GP7*. Prepared from tetrakis(triphenylphosphine)palladium(0) (573 mg, 0.50 mmol), 4-iodoanisole (**1b**) (246 mg, 1.05 mmol), toluene (10 mL). Yield: 280 mg (0.32 mmol, 64 %) of white powder.

Mp: decomposes at 136.1 °C.

^{1}H NMR (500 MHz, $CDCl_3$) δ = 7.55–7.47 (m, 12H), 7.36–7.29 (m, 6H), 7.28–7.22 (m, 12H), 6.40 (dt, J = 8.6, 1.9 Hz, 2H), 5.92 (d, J = 8.5 Hz, 2H), 3.94 (s, 3H).

^{31}P{^{1}H} NMR (202 MHz, $CDCl_3$) δ = +22.8 (s).

Spectroscopic data are in agreement with literature.[23]

Synthesis of bis(triphenylphosphine)palladium(II) (4-nitrophenyl)ide iodide (7c)

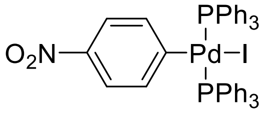

Following *GP7*. Prepared from tetrakis(triphenylphosphine)palladium(0) (589 mg, 0.51 mmol), 1-iodo-4-nitrobenzene (**1c**) (244 mg, 0.98 mmol), toluene (10 mL). Yield: 199 mg (0.23 mmol, 46 %) of white powder.

Mp: decomposes at 174.6 °C.

^{1}H NMR (500 MHz, $CDCl_3$) δ = 7.57–7.50 (m, 12H), 7.37–7.32 (m, 6H), 7.29–7.23 (m, 12H), 7.01 (d, J = 8.5 Hz, 2H), 6.84 (dt, J = 8.6, 1.6 Hz, 2H).

^{31}P{^{1}H} NMR (202 MHz, $CDCl_3$) δ = +22.5 (s).

Spectroscopic data are in agreement with literature.[23]

Synthesis of bis(triphenylphosphine)palladium(II) (4-methylphenyl)ide bromide (7d)

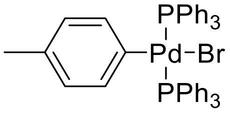

Following *GP8*. Prepared from 4-bromotoluene (**1d**) (617 mg, 3.61 mmol), triphenylphosphine (193 mg, 0.736 mmol), tetrakis(triphenylphosphine)palladium(0) (421 mg, 0.364 mmol), toluene (6 mL). Yield: 178 mg (0.222 mmol, 61 %) of grey powder.

Mp: 167.0–168.3 °C.

^{1}H NMR (500 MHz, $CDCl_3$) δ = 7.54–7.45 (m, 12H), 7.35–7.29 (m, 6H), 7.26–7.20 (m, 12H), 6.42 (dt, J = 8.0, 2.0 Hz, 2H), 6.07 (d, J = 7.6 Hz, 2H), 1.92 (s, 3H).

^{31}P{^{1}H} NMR (202 MHz, $CDCl_3$) δ = +23.1 (s).

Spectroscopic data are in agreement with literature.[24]

Synthesis of bis(triphenylphosphine)palladium(II) (4-methoxyphenyl)ide bromide (7e)

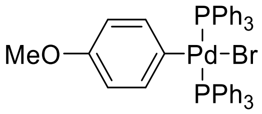

Following *GP8*. Prepared from 4-bromoanisole (**1e**) (0.305 mL, 2.44 mmol), triphenylphosphine (140 mg, 0.53 mmol), tetrakis(triphenylphosphine)palladium(0) (400 mg, 0.35 mmol), toluene (3 mL). Yield: 132 mg (0.161 mmol, 46 %) of yellow powder.

Mp: decomposes at 162.4 °C.

^{1}H NMR (500 MHz, $CDCl_3$) δ = 7.55–7.47 (m, 12H), 7.35–7.30 (m, 6H), 7.28–7.22 (m, 12H), 6.41 (d, J = 8.3 Hz, 2H), 5.92 (d, J = 8.3 Hz, 2H), 3.50 (s, 3H).

^{31}P{^{1}H} NMR (202 MHz, $CDCl_3$) δ = +23.4 (s).

Spectroscopic data are in agreement with literature.[24]

Synthesis of bis(triphenylphosphine)palladium(II) (4-nitrophenyl)ide bromide (7f)

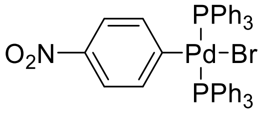

Following *GP8*. Prepared from 4-bromo-1-nitrobenzene (**1f**) (493 mg, 2.44 mmol), triphenylphosphine (140 mg, 0.53 mmol), tetrakis(triphenylphosphine)palladium(0) (400 mg, 0.35 mmol), toluene (3 mL). Yield: 148 mg (0.178 mmol, 51 %) of yellow powder.

Mp: decomposes at 198.4 °C.

^{1}H NMR (500 MHz, $CDCl_3$) δ = 7.59–7.50 (m, 12H), 7.39–7.32 (m, 6H), 7.30–7.22 (m, 12H), 7.00 (d, J = 8.5 Hz, 2H), 6.86 (dt, J = 8.8, 1.5 Hz, 2H).

^{31}P{^{1}H} NMR (202 MHz, $CDCl_3$) δ = +23.5 (s).

Spectroscopic data are in agreement with literature.[24]

Synthesis of bis(triphenylphosphine)palladium(II) (4-methylphenyl)ide chloride (7g)

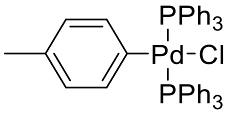

Following *GP9*. Prepared from bis(triphenylphosphine)palladium(II) (4-methylphenyl)ide iodide (**7a**) (256 mg, 0.302 mmol), tetrabutylammonium chloride (851 mg, 3.06 mmol), dichloromethane (20 mL). Yield: 176 mg (0.232 mmol, 77 %) of grey powder.

Mp: 183.4–186.7 °C.

^{1}H NMR (500 MHz, $CDCl_3$) δ = 7.54–7.44 (m, 12H), 7.36–7.29 (m, 6H), 7.26–7.20 (m, 12H), 6.42 (dt, J = 8.0, 1.9 Hz, 2H), 6.08 (d, J = 7.6 Hz, 2H), 1.93 (s, 3H).

$^{31}P\{^{1}H\}$ NMR (202 MHz, $CDCl_3$) δ = +23.1 (s).

Spectroscopic data are in agreement with literature.[22]

Synthesis of bis(triphenylphosphine)palladium(II) (4-methoxyphenyl)ide chloride (7h)

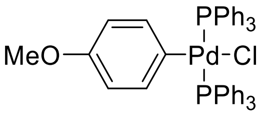

Following *GP9*. Prepared from bis(triphenylphosphine)palladium(II) (4-methoxyphenyl)ide iodide (**7b**) (190 mg, 0.22 mmol), tetrabutylammonium chloride (466 mg, 1.68 mmol), dichloromethane (5 mL). Yield: 159 mg (0.206 mmol, 94 %) of white powder.

Mp: decomposes at 202 °C.

^{1}H NMR (500 MHz, $CDCl_3$) δ = 7.55–7.47 (m, 12H), 7.36–7.30 (m, 6H), 7.29–7.21 (m, 12H), 6.42 (dt, *J* = 8.6, 1.7 Hz, 2H), 5.94 (d, *J* = 8.5 Hz, 2H), 3.51 (s, 3H).

$^{31}P\{^{1}H\}$ NMR (202 MHz, $CDCl_3$) δ = +23.4 (s).

Spectroscopic data are in agreement with literature.[22]

Synthesis of bis(triphenylphosphine)palladium(II) (4-nitrophenyl)ide chloride (7i)

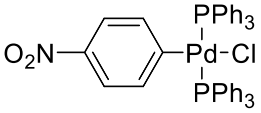

Following *GP9*. Prepared from bis(triphenylphosphine)palladium(II) (4-nitrophenyl)ide iodide (**7c**) (264 mg, 0.300 mmol), tetrabutylammonium chloride (864 mg, 3.11 mmol), dichloromethane (6 mL). Yield: 228 mg (0.254 mmol, 96 %) of grey powder.

Mp: decomposes at 211 °C.

^{1}H NMR (500 MHz, $CDCl_3$) δ = 7.57–7.48 (m, 12H), 7.36–7.30 (m, 6H), 7.26–7.20 (m, 12H), 6.88 (dt, *J* = 8.2, 1.7 Hz, 2H), 6.66 (d, *J* = 8.1 Hz, 2H).

$^{31}P\{^{1}H\}$ NMR (202 MHz, $CDCl_3$) δ = +23.6 (s).

Spectroscopic data are in agreement with literature.[22]

Synthesis of bis(triphenylphosphine)palladium(II) (4-methylphenyl)ide acetate (7j)

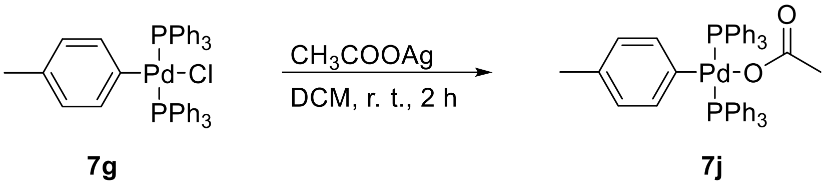

An oven-dried round-bottom reaction flask was fitted with a stirring bar and closed with a rubber septum, which was pierced with a needle connected by a tube to a Schlenk vacuum manifold. The flask was cooled to room temperature under an argon atmosphere and charged with bis(triphenylphospine)palladium (4-methylphenyl)ide chloride (**7g**) (454 mg, 0.60 mmol) by rapidly opening the septum. Dichloromethane (20 mL) was added with a syringe by piercing the septum under argon atmosphere. Silver acetate (135 mg, 0.81 mmol) was added to this solution by rapidly opening the septum and purging with argon. The resulting suspension was stirred vigorously for 2 h and filtered through a pad of celite. The filtrate was collected and the solvent was removed using a rotary evaporator to give a white solid. The crude product was crystallized from a mixture of chloroform and hexane (V/V = 1/3, 40 mL). Yield: 227 mg (0.291 mmol, 49 %) of white crystals.

Mp: 141.6–145.7 °C.

IR (cm^{-1}): 3051, 1599, 1580, 1479, 1432, 1374, 1324, 1184, 1096, 798, 742, 690, 664.

^{1}H NMR (500 MHz, $CDCl_3$) δ = 7.47–7.36 (m, 12H), 7.36–7.30 (m, 6H), 7.29–7.20 (m, 12H), 6.39 (d, J = 7.6 Hz, 2H), 6.18 (d, J = 7.5 Hz, 2H), 2.02 (s, 3H), 0.94 (s, 3H).

$^{13}C\{^{1}H\}$ NMR (126 MHz, $CDCl_3$) δ = 176.1, 140.4, 137.6 (t, J = 4.0 Hz), 134.7 (t, J = 6.1 Hz), 131.2, 130.7 (t, J = 21.9 Hz), 129.8, 128.1, 128.0 (t, J = 4.8 Hz), 23.7, 20.5.

$^{31}P\{^{1}H\}$ NMR (202 MHz, $CDCl_3$) δ = +20.0 (s).

HRMS: calculated for $C_{43}H_{37}P_2Pd$ [M-acetate]$^+$ 721.1405, found 721.1403.

Synthesis of bis(triphenylphosphine)palladium(II) phenylide iodide (7k)

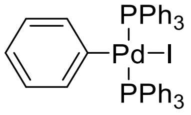

Following *GP7*. Prepared from tetrakis(triphenylphosphine)palladium(0) (580 mg, 0.50 mmol), iodobenzene (**1g**) (0.112 mL, 1.00 mmol), toluene (10 mL). Yield: 267 mg (0.32 mmol, 64 %) of white powder.

Mp: decomposes at 170.8 °C.

^{1}H NMR (500 MHz, $CDCl_3$) δ = 7.54–7.45 (m, 12H), 7.35–7.29 (m, 6H), 7.26–7.21 (m, 12H), 6.60 (d, J = 6.8 Hz, 2H), 6.33 (t, J = 7.2 Hz, 1H), 6.21 (t, J = 7.3 Hz, 2H).

$^{31}P\{^{1}H\}$ NMR (202 MHz, $CDCl_3$) δ = +22.9 (s).

Spectroscopic data are in agreement with literature.[25]

Synthesis of bis(triphenylphosphine)palladium(II) (4-*tert*-butylphenyl)ide iodide (7l)

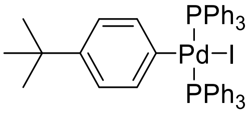

Following *GP7*. Prepared from tetrakis(triphenylphosphine)palladium(0) (590 mg, 0.51 mmol), 1-iodo-4-(*tert*-butyl)benzene (**1h**) (0.177 mL, 1.00 mmol), toluene (10 mL). Yield: 326 mg (0.37 mmol, 72 %) of white powder.

Mp: decomposes at 141.9 °C.

IR (cm^{-1}): 3055, 2947, 1574, 1479, 1433, 1156, 1159, 1095, 1028, 1007, 811, 743, 690.

^{1}H NMR (500 MHz, $CDCl_3$) δ = 7.54–7.47 (m, 12H), 7.35–7.28 (m, 6H), 7.26–7.20 (m, 12H), 6.49 (dt, J = 8.4, 2.1 Hz, 2H), 6.27 (d, J = 8.2 Hz, 2H), 1.06 (s, 9H).

$^{13}C\{^{1}H\}$ NMR (126 MHz, $CDCl_3$) δ = 154.5, 144.6, 135.0 (t, J = 6.0 Hz), 132.4 (t, J = 22.9 Hz), 129.81, 129.76, 127.9 (t, J = 5.3 Hz), 125.2, 33.8, 31.7.

$^{31}P\{^{1}H\}$ NMR (202 MHz, $CDCl_3$) δ = +22.8 (s).

HRMS: calculated for $C_{46}H_{43}P_2Pd$ $[M-I]^+$ 763.1876, found 763.1875.

Synthesis of bis(triphenylphosphine)palladium(II) (4-(hydroxymethyl)phenyl)ide iodide (7m)

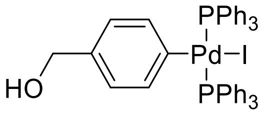

Following *GP7*. Prepared from tetrakis(triphenylphosphine)palladium(0) (578 mg, 0.50 mmol), (4-iodophenyl)methanol (**1i**) (240 mg, 1.03 mmol), toluene (10 mL). Yield: 150 mg (0.17 mmol, 35 %) of white powder.

Mp: decomposes at 147.3 °C.

IR (cm^{-1}): 3506, 3047,1582, 1571, 1478, 1432, 1382, 1180, 1093, 1051, 1027, 1010, 999, 812, 781, 749, 739, 687, 618.

^{1}H NMR (500 MHz, $CDCl_3$) δ = 7.58–7.47 (m, 12H), 7.35–7.29 (m, 6H), 7.27–7.21 (m, 12H), 6.62 (dt, J = 8.1, 2.0 Hz, 2H), 6.23 (d, J = 7.8 Hz, 2H), 4.19 (d, J = 5.8 Hz, 2H), 0.91 (t, J = 5.8 Hz, 1H).

$^{13}C\{^{1}H\}$ NMR (126 MHz, $CDCl_3$) δ = 159.7, 136.1 (t, J = 5.1 Hz), 135.1 (t, J = 6.5 Hz), 134.6, 132.2 (t, J = 23.3 Hz), 129.9, 127.9 (t, J = 5.4 Hz), 127.1, 65.7.

$^{31}P\{^{1}H\}$ NMR (202 MHz, $CDCl_3$) δ = + 23.0 (s).

HRMS: calculated for $C_{43}H_{37}OP_2Pd$ $[M-I]^+$ 737.1354, found 737.1349.

Synthesis of bis(triphenylphosphine)palladium(II) (4-chlorophenyl)ide iodide (7n)

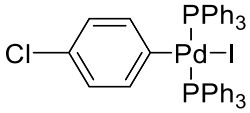

Following *GP7*. Prepared from tetrakis(triphenylphosphine)palladium(0) (583 mg, 0.50 mmol), 4-chloro-1-iodobenzene (**1j**) (247 mg, 1.04 mmol), toluene (10 mL). Yield: 314 mg (0.36 mmol, 72 %) of white powder.

Mp: 178.8–179.6 °C.

IR (cm^{-1}): 3048, 1586, 1571, 1479, 1464, 1433, 1182, 1094, 1086, 1045, 1026, 1007, 843, 793, 750, 740, 702, 689, 618.

^{1}H NMR (500 MHz, $CDCl_3$) δ = 7.56–7.48 (m, 12H), 7.39–7.32 (m, 6H), 7.30–7.22 (m, 12H), 6.48 (dt, J = 8.4, 1.8 Hz, 2H), 6.21 (d, J = 8.2 Hz, 2H).

$^{13}C\{^{1}H\}$ NMR (126 MHz, $CDCl_3$) δ = 156.9 (t, J = 2.9 Hz), 136.6 (t, J = 5.1 Hz), 135.0 (t, J = 6.3 Hz), 131.9 (t, J = 23.1 Hz), 130.0, 128.0 (t, J = 5.3 Hz), 127.6.

$^{31}P\{^{1}H\}$ NMR (202 MHz, $CDCl_3$) δ = +22.7 (s).

HRMS: calculated for $C_{42}H_{34}ClP_2Pd$ $[M-I]^+$ 741.0859, found 741.0860.

Synthesis of bis(triphenylphosphine)palladium(II) (4-(trifluoromethyl)phenyl)ide iodide (7o)

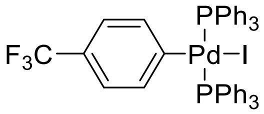

Following *GP7*. Prepared from tetrakis(triphenylphosphine)palladium(0) (576 mg, 0.50 mmol), 1-iodo-4-(trifluoromethyl)benzene (**1k**) (313 mg, 1.15 mmol), toluene (10 mL). Yield: 207 mg (0.23 mmol, 46 %) of white powder.

Mp: decomposes at 174.0 °C.

^{1}H NMR (500 MHz, $CDCl_3$) δ = 7.57–7.47 (m, 12H), 7.38–7.30 (m, 6H), 7.29–7.21 (m, 12H), 6.72 (d, J = 7.8 Hz, 2H), 6.40 (d, J = 7.8 Hz, 2H).

$^{31}P\{^{1}H\}$ NMR (202 MHz, $CDCl_3$) δ = +23.0 (s).

^{19}F NMR (470 MHz, $CDCl_3$) δ = −62.1 (s).

Spectroscopic data in agreement with literature.[23]

Synthesis of bis(triphenylphosphine)palladium(II) (4-formylphenyl)ide iodide (7p)

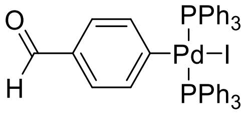

Following *GP7*. Prepared from tetrakis(triphenylphosphine)palladium(0) (483 mg, 0.42 mmol), 4-iodobenzaldehyde (**1l**) (235 mg, 1.01 mmol), toluene (10 mL). Yield: 209 mg (0.24 mmol, 57 %) of white powder.

Mp: 172.1–173.8 °C.

IR (cm^{-1}): 3047, 1677, 1654, 1568, 1547, 1478, 1432, 1378, 1296, 1211, 1182, 1161, 1094, 1046, 1027, 998, 829, 804, 741, 689.

^{1}H NMR (500 MHz, $CDCl_3$) δ = 9.51 (s, 1H), 7.57–7.48 (m, 12H), 7.36–7.30 (m, 6H), 7.26–7.20 (m, 12H), 6.88 (dt, J = 8.2, 1.7 Hz, 2H), 6.66 (d, J = 8.1 Hz, 2H).

$^{13}C\{^{1}H\}$ NMR (126 MHz, $CDCl_3$) δ = 192.8, 176.7 (t, J = 2.9 Hz), 136.5 (t, J = 4.7 Hz), 134.9 (t, J = 6.4 Hz), 131.7 (t, J = 23.7 Hz), 131.3, 130.2, 128.0 (t, J = 5.5 Hz), 127.7.

$^{31}P\{^{1}H\}$ NMR (202 MHz, $CDCl_3$) δ = +22.8 (s).

HRMS: calculated for $C_{43}H_{35}OP_2Pd$ $[M-I]^+$ 731.1214, found 731.1206.

Synthesis of bis(triphenylphosphine)palladium(II) (4-acetylphenyl)ide iodide (7q)

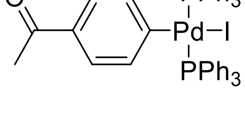

Following *GP7*. Prepared from tetrakis(triphenylphosphine)palladium(0) (584 mg, 0.51 mmol), 4-iodoacetophenone (**1m**) (247 mg, 1.00 mmol), toluene (10 mL). Yield: 277 mg (0.32 mmol, 63 %) of white powder.

Mp: decomposes at 159.9 °C.

IR (cm^{-1}): 3048, 3023, 1670, 1567, 1540, 1480, 1434, 1383, 1355, 1308, 1274, 1181, 1158, 1092, 1045, 1027, 1008, 998, 957, 833, 807, 744, 691, 667, 617.

^{1}H NMR (500 MHz, $CDCl_3$) δ = 7.56–7.49 (m, 12H), 7.36–7.29 (m, 6H), 7.26–7.20 (m, 12H), 6.76 (s, 4H), 2.28 (s, 3H).

^{13}C{^{1}H} NMR (126 MHz, $CDCl_3$) δ = 198.7, 172.7 (t, J = 3.2 Hz), 136.0 (t, J = 5.1 Hz), 135.0 (t, J = 6.3 Hz), 131.8 (t, J = 23.5 Hz), 130.1, 128.0 (t, J = 5.3 Hz), 126.6, 26.4.

^{31}P{^{1}H} NMR (202 MHz, $CDCl_3$) δ = +22.7 (s).

HRMS: calculated for $C_{44}H_{37}OP_2Pd$ $[M-I]^+$ 749.1354, found 749.1357.

Synthesis of bis(triphenylphosphine)palladium(II) iodide

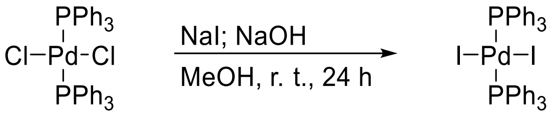

Bis(triphenylphosphine)palladium(II) iodide was prepared following the modified literature procedure.[26] An oven-dried round-bottom reaction flask was fitted with a stirring bar and closed with a rubber septum, which was pierced with a needle connected by a tube to a Schlenk vacuum manifold. The flask was allowed to cool to room temperature under argon atmosphere. The flask was charged with bis(triphenylphosphine)palladium(II) dichloride (366 mg, 0.52 mmol) by rapidly opening the septum and flushing with argon. A 0.5 M solution of NaOH in methanol (10 mL) and sodium iodide (758 mg, 5.06 mmol) were added by rapidly opening the septum and flushing with argon. The reaction mixture was purged with argon for 5 min. Argon was applied by the needle connected to a Schlenk vacuum manifold. The mixture was stirred overnight in the dark at room temperature. The mixture was then filtered and the solid residue was washed with water (20 mL), methanol (20 mL) and diethyl ether (20 mL). The residue was air-dried to give the desired product in pure form.

Mp: 276–279 °C.

^{1}H NMR (500 MHz, $CDCl_3$) δ = 7.74–7.65 (m, 12H), 7.42–7.33 (m, 18H).

^{31}P{^{1}H} NMR (202 MHz, $CDCl_3$) δ = +12.8 (s).

Spectroscopic data in agreement with literature.[26]

2.1.3. Syntheses of 1,2-disubstituted alkynes 3

General procedure 10 (*GP10*) – Synthesis of 1,2-disubstituted alkynes

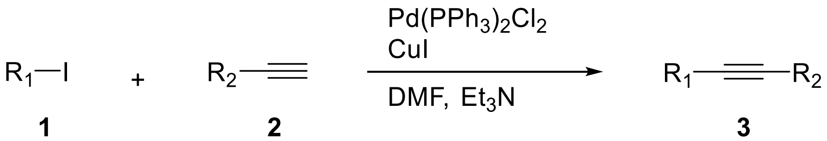

1 **2** **3**

An oven-dried round-bottom reaction flask was fitted with a stirring bar and closed with a rubber septum, which was pierced with a needle connected by a tube to a Schlenk vacuum manifold. The flask was cooled to room temperature under argon atmosphere. The flask was charged with aryl iodide **1** (1 equiv.) and alkyne **2** (1.2 equiv.) by rapidly opening the septum and flushing with argon. DMF (2 mL/mmol) was added with a syringe by piercing the septum. To this solution triethylamine (7 equiv.), bis(triphenylphosphine)palladium dichloride (0.05 equiv.) and copper(I) iodide (0.10 equiv.) were added by rapidly opening the septum and flushing with argon. Solution was stirred overnight at the room temperature under argon atmosphere. The reaction mixture was extracted with diethyl ether (2 × 20 mL). Combined organic layers were washed with water (2 × 30 mL) and brine (2 × 30 mL). The organic phase was dried over anhydrous Na_2SO_4, filtered and concentrated *in vacuo* using a rotary evaporator. The crude product was purified by column chromatography and the fractions containing the product were combined and the volatile components were evaporated *in vacuo* using a rotary evaporator.

Synthesis of 1-methoxy-4-(phenylethynyl)benzene (3a)

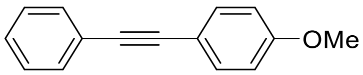

Following *GP10*. Prepared from bis(triphenylphosphine)palladium(II) dichloride (39 mg, 0.056 mmol), copper(I) iodide (25 mg, 0.13 mmol), 4-iodoanisole (**1b**) (242 mg, 1.03 mmol), phenylacetylene (**2a**) (0.132 mL, 1.20 mmol), triethylamine (0.976 mL, 7.00 mmol), DMF (2 mL). Yield: 154 mg (0.74 mmol, 72 %) of white powder.

^{1}H NMR (500 MHz, $CDCl_3$) δ = 7.55–7.44 (m, 4H), 7.38–7.29 (m, 3H), 6.88 (d, J = 8.8 Hz, 2H), 3.83 (s, 3H).

Spectroscopic data are in agreement with literature.[27]

Synthesis of 2-(4-tolylethynyl)pyridine (3b)

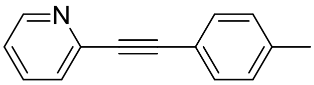

Following *GP10*. Prepared from bis(triphenylphosphine)palladium(II) dichloride (36 mg, 0.051 mmol), copper(I) iodide (20 mg, 0.10 mmol), 4-iodotoluene (**1a**) (216 mg, 0.99 mmol), 2-ethynylpyridine (**2d**) (0.121 mL, 1.20 mmol), triethylamine (0.976 mL, 7.00 mmol), DMF (2 mL). Yield: 153 mg (0.79 mmol, 80 %) of orange solid.

^{1}H NMR (500 MHz, $CDCl_3$) δ = 8.60 (d, J = 4.3 Hz, 1H), 7.65 (dt, J = 7.8, 1.8 Hz, 1H), 7.53–7.45 (m, 3H), 7.21 (ddd, J = 7.6, 4.9, 1.0 Hz, 1H), 7.16 (d, J = 7.9 Hz, 2H), 2.36 (s, 3H).

Spectroscopic data are in agreement with literature.[28]

Synthesis of 1-methyl-4-((4-nitrophenyl)ethynyl)benzene (3c)

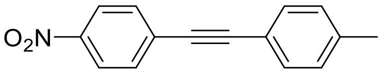

Following *GP10*. Prepared from bis(triphenylphosphine)palladium(II) dichloride (40 mg, 0.057 mmol), copper(I) iodide (21 mg, 0.11 mmol), 1-iodo-4-nitrobenzene (**1c**) (243 mg, 0.98 mmol), 4-ethynyltoluene (**2g**) (0.152 mL, 1.20 mmol), triethylamine (0.976 mL, 7.00 mmol), DMF (2 mL). Yield: 49 mg (0.21 mmol, 21 %) of yellow solid.

^{1}H NMR (500 MHz, $CDCl_3$) δ = 8.21 (d, J = 8.9 Hz, 2H), 7.65 (d, J = 9.0 Hz, 2H), 7.45 (d, J = 8.1 Hz, 2H), 7.20 (d, J = 7.9 Hz, 2H), 2.39 (s, 3H).

Spectroscopic data are in agreement with literature.[29]

Synthesis of 1-(6-chlorohex-1-yn-1-yl)-4-methylbenzene (3d)

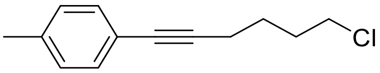

Following *GP10*. Prepared from bis(triphenylphosphine)palladium(II) dichloride (36 mg, 0.051 mmol), copper(I) iodide (21 mg, 0.11 mmol), 4-iodotoluene (**1a**) (214 mg, 0.98 mmol), 6-chlorohex-1-yne (**2e**) (0.145 mL, 1.20 mmol), triethylamine (0.976 mL, 7.00 mmol), DMF (2 mL). Yield: 176 mg (0.85 mmol, 87 %) of yellow oil.

^{1}H NMR (500 MHz, $CDCl_3$) δ = 7.31 (d, J = 7.9 Hz, 2H), 7.11 (d, J = 7.7 Hz, 2H), 3.61 (t, J = 6.6 Hz, 2H), 2.47 (t, J = 7.0 Hz, 2H), 2.35 (s, 3H), 2.01–1.93 (m, 2H), 1.81–1.73 (m, 2H).

Spectroscopic data in agreement with literature.[30]

Synthesis of 1-methyl-4-(phenylethynyl)benzene (3e)

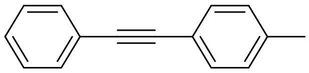

Following *GP10*. Prepared from bis(triphenylphosphine)palladium(II) dichloride (37 mg, 0.053 mmol), copper(I) iodide (23 mg, 0.12 mmol), 4-iodotoluene (**1a**) (218 mg, 1.00 mmol), phenylacetylene (**2a**) (0.132 mL, 1.20 mmol), triethylamine (0.976 mL, 7.00 mmol), DMF (2 mL). Yield: 170 mg (0.88 mmol, 88 %) of white powder.

^{1}H NMR (300 MHz, $CDCl_3$) δ = 7.57–7.49 (m, 2H), 7.43 (d, J = 8.1 Hz, 2H), 7.37–7.30 (m, 3H), 7.16 (d, J = 8.1 Hz, 2H), 2.37 (s, 3H).

Spectroscopic data in agreement with literature.[27]

Synthesis of 1-(*tert*-butyl)-4-(phenylethynyl)benzene (3f)

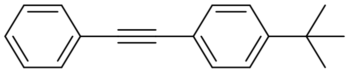

Following *GP10*. Prepared from bis(triphenylphosphine)palladium(II) dichloride (35 mg, 0.050 mmol), copper(I) iodide (21 mg, 0.11 mmol), 4-*tert*-butyliodobenzene (**1h**) (0.178 mL, 1.00 mmol), phenylacetylene (**2a**) (0.132 mL, 1.20 mmol), triethylamine (0.976 mL, 7.00 mmol), DMF (2 mL). Yield: 233 mg (0.99 mmol, 99 %) of yellow oil.

^{1}H NMR (500 MHz, $CDCl_3$) δ = 7.57–7.54 (m, 2H), 7.50 (dt, J = 8.5, 1.9 Hz, 2H), 7.42–7.34 (m, 5H), 1.35 (s, 9H).

Spectroscopic data are in accordance with literature.[31]

Synthesis of 1-(phenylethynyl)-4-(trifluoromethyl)benzene (3g)

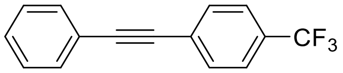

Following *GP10*. Prepared from bis(triphenylphosphine)palladium(II) dichloride (38 mg, 0.054 mmol), copper(I) iodide (25 mg, 0.13 mmol), 4-iodobenzotrifluoride (**1k**) (283 mg, 1.04 mmol), phenylacetylene (**2a**) (0.132 mL, 1.20 mmol), triethylamine (0.976 mL, 7.00 mmol), DMF (2 mL). Yield: 179 mg (0.73 mmol, 70 %) of white solid.

^{1}H NMR (500 MHz, $CDCl_3$) δ = 7.62 (dd, J = 14.6, 8.6 Hz, 4H), 7.58–7.53 (m, 2H), 7.41–7.35 (m, 3H).

^{19}F{^{1}H} NMR (471 MHz, $CDCl_3$) δ = −62.8 (s)

Spectroscopic data are in agreement with literature.[32]

Synthesis of (4-(phenylethynyl)phenyl)methanol (3h)

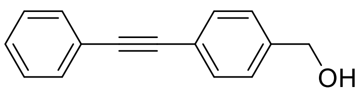

Following *GP10*. Prepared from bis(triphenylphosphine)palladium(II) dichloride (37 mg, 0.053 mmol), copper(I) iodide (23 mg, 0.12 mmol), 4-iodobenzyl alcohol (**1i**) (249 mg, 1.06 mmol), phenylacetylene (**2a**) (0.132 mL, 1.20 mmol), triethylamine (0.976 mL, 7.00 mmol), DMF (2 mL). Yield: 79 mg (0.38 mmol, 36 %) of orange solid.

^{1}H NMR (500 MHz, $CDCl_3$) δ = 7.58–7.49 (m, 4H), 7.42–7.31 (m, 5H), 4.72 (d, J = 6.0 Hz, 2H), 1.67 (t, J = 6.0 Hz, 1H).

Spectroscopic data are in agreement with literature.[33]

Synthesis of 1-(4-(phenylethynyl)phenyl)ethan-1-one (3i)

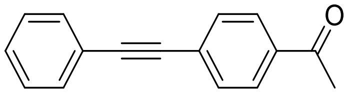

Following *GP10*. Prepared from bis(triphenylphosphine)palladium(II) dichloride (35 mg, 0.050 mmol), copper(I) iodide (22 mg, 0.12 mmol), 4-iodobenzene (**1g**) (0.122 mL, 1.00 mmol), 4-acetylphenylacetylene (**2n**) (175 mg, 1.21 mmol), triethylamine (0.976 mL, 7.00 mmol), DMF (2 mL). Yield: 129 mg (0.59 mmol, 59 %) of beige solid.

^{1}H NMR (500 MHz, $CDCl_3$) δ = 7.95 (dt, J = 8.5, 1.7 Hz, 2H), 7.61 (dt, J = 8.5, 1.7 Hz, 2H), 7.58–7.53 (m, 2H), 7.40–7.35 (m, 3H).

Spectroscopic data in agreement with literature.[32]

Synthesis of 1-nitro-4-(phenylethynyl)benzene (3j)

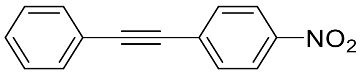

Following *GP10*. Prepared from bis(triphenylphosphine)palladium(II) dichloride (39 mg, 0.056 mmol), copper(I) iodide (25 mg, 0.13 mmol), 1-iodo-4-nitrobenzene (**1c**) (246 mg, 0.99 mmol), phenylacetylene (**2a**) (0.132 mL, 1.20 mmol), triethylamine (0.976 mL, 7.00 mmol), DMF (2 mL). Yield: 123 mg (0.55 mmol, 56 %) of yellow solid.

^{1}H NMR (500 MHz, $CDCl_3$) δ = 8.23 (dt, J = 8.9, 2.1 Hz, 2H), 7.67 (dt, J = 8.9, 2.1 Hz, 2H), 7.59–7.53 (m, 2H), 7.42–7.36 (m, 3H).

Spectroscopic data are in agreement with literature.[32]

Synthesis of 1-(*tert*-butyl)-4-(4-tolylethynyl)benzene (3k)

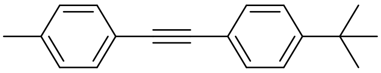

Following *GP10*. Prepared from bis(triphenylphosphine)palladium(II) dichloride (37 mg, 0.053 mmol), copper(I) iodide (24 mg, 0.13 mmol), 4-iodotoluene (**1a**) (220 mg, 1.01 mmol), 1-ethynyl-(4-*tert*-butyl)benzene (**2i**) (0.213 mL, 1.18 mmol), triethylamine (0.976 mL, 7.00 mmol), DMF (2 mL). Yield: 230 mg (0.93 mmol, 92 %) of yellow solid.

^{1}H NMR (500 MHz, $CDCl_3$) δ = 7.51–7.41 (m, 4H), 7.36 (dt, J = 8.6, 1.9 Hz, 2H), 7.14 (d, J = 7.9 Hz, 2H), 2.36 (s, 3H), 1.33 (s, 9H).

Spectroscopic data are in agreement with literature.[34]

Synthesis of trimethyl(4-(4-tolylethynyl)phenyl)silane (3l)

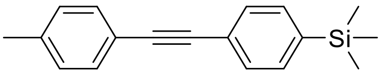

Following *GP10*. Prepared from bis(triphenylphosphine)palladium(II) dichloride (36 mg, 0.051 mmol), copper(I) iodide (21 mg, 0.11 mmol), 4-iodotoluene (**1a**) (217 mg, 1.00 mmol), (4-ethynylphenyl)trimethylsilane (**2o**) (0.166 mL, 0.86 mmol), triethylamine (0.976 mL, 7.00 mmol), DMF (2 mL). Yield: 132 mg (0.50 mmol, 58 %) of orange solid.

^{1}H NMR (500 MHz, $CDCl_3$) δ = 7.49 (s, 4H), 7.43 (d, J = 8.1 Hz, 2H), 7.16 (d, J = 8.0 Hz, 2H), 2.37 (s, 3H), 0.27 (s, 9H).

Spectroscopic data are in agreement with literature.[35]

Synthesis of 1-chloro-4-(4-tolylethynyl)benzene (3m)

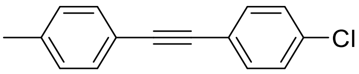

Following *GP10*. Prepared from bis(triphenylphosphine)palladium(II) dichloride (37 mg, 0.053 mmol), copper(I) iodide (21 mg, 0.11 mmol), 1-chloro-4-iodobenzene (**1j**) (236 mg, 0.99 mmol), 4-ethynyltoluene (**2g**) (0.152 mL, 1.20 mmol), triethylamine (0.976 mL, 7.00 mmol), DMF (2 mL). Yield: 219 mg (0.93 mmol, 97 %) of white solid.

^{1}H NMR (500 MHz, $CDCl_3$) δ = 7.43 (tt, J = 8.7, 2.1 Hz, 4H), 7.31 (dt, J = 8.7, 2.1 Hz, 2H), 7.16 (d, J = 7.9 Hz, 2H), 2.37 (s, 3H).

Spectroscopic data are in agreement with literature.[33]

Synthesis of 1-bromo-4-(4-tolylethynyl)benzene (3n)

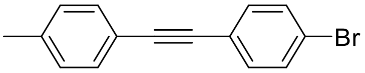

Following *GP10*. Prepared from bis(triphenylphosphine)palladium(II) dichloride (37 mg, 0.053 mmol), copper(I) iodide (23 mg, 0.12 mmol), 4-iodotoluene (**1a**) (216 mg, 0.99 mmol), 1-bromo-4-ethynylbenzene (**2k**) (223 mg, 1.23 mmol), triethylamine (0.976 mL, 7.00 mmol), DMF (2 mL). Yield: 126 mg (0.46 mmol, 46 %) of yellow solid.

^{1}H NMR (500 MHz, $CDCl_3$) δ = 7.47 (dt, J = 8.6, 2.1 Hz, 2H), 7.42 (d, J = 8.1 Hz, 2H), 7.38 (dt, J = 8.6, 2.1 Hz, 2H), 7.16 (d, J = 8.1 Hz, 2H), 2.37 (s, 3H).

Spectroscopic data are in agreement with literature.[32]

Synthesis of 1-methyl-4-((4-(trifluoromethyl)phenyl)ethynyl)benzene (3o)

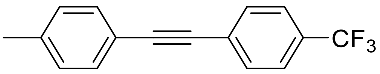

Following *GP10*. Prepared from bis(triphenylphosphine)palladium(II) dichloride (35 mg, 0.050 mmol), copper(I) iodide (26 mg, 0.14 mmol), 4-iodobenzotrifluoride (**1k**) (294 mg, 1.08 mmol), 4-ethynyltoluene (**2g**) (0.152 mL, 1.20 mmol), triethylamine (0.976 mL, 7.00 mmol), DMF (2 mL). Yield: 167 mg (0.64 mmol, 59 %) of white solid.

^{1}H NMR (500 MHz, $CDCl_3$) δ = 7.43 (tt, J = 8.7, 2.2 Hz, 4H), 7.31 (dt, J = 8.7, 2.2 Hz, 2H), 7.16 (d, J = 7.9 Hz, 2H), 2.37 (s, 3H).

^{19}F{^{1}H} NMR (470 MHz, $CDCl_3$) δ = -62.7 (s).

Spectroscopic data are in agreement with literature.[36]

Synthesis of 4-(4-tolylethynyl)benzaldehyde (3p)

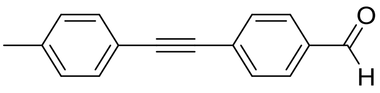

Following *GP10*. Prepared from bis(triphenylphosphine)palladium(II) dichloride (37 mg, 0.053 mmol), copper(I) iodide (22 mg, 0.11 mmol), 4-iodotoluene (**1a**) (216 mg, 0.99 mmol), 4-ethynylbenzaldehyde (**2m**) (157 mg, 1.21 mmol), triethylamine (0.976 mL, 7.00 mmol), DMF (2 mL). Yield: 102 mg (0.46 mmol, 47 %) of yellow solid.

^{1}H NMR (500 MHz, $CDCl_3$) δ = 10.02 (s, 1H), 7.86 (d, J = 8.4 Hz, 2H), 7.66 (d, J = 8.2 Hz, 2H), 7.45 (d, J = 8.2 Hz, 2H), 7.19 (d, J = 8.2 Hz, 2H), 2.39 (s, 3H).

Spectroscopic data are in agreement with literature.[37]

Synthesis of 1-methoxy-4-(4-tolylethynyl)benzene (3q)

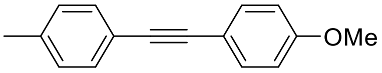

Following *GP10*. Prepared from bis(triphenylphosphine)palladium(II) dichloride (36 mg, 0.051 mmol), copper(I) iodide (20 mg, 0.11 mmol), 4-iodotoluene (**1a**) (218 mg, 1.00 mmol), 4-ethynylanisole (**2h**) (0.156 mL, 1.20 mmol), triethylamine (0.976 mL, 7.00 mmol), DMF (2 mL). Yield: 173 mg (0.778 mmol, 78 %) of white solid.

^{1}H NMR (500 MHz, $CDCl_3$) δ = 7.43 (d, J = 8.8 Hz, 2H), 7.41 (d, J = 8.1 Hz, 2H), 7.14 (d, J = 8.1 Hz, 2H), 6.87 (d, J = 8.8 Hz, 2H), 3.83 (s, 3H), 2.36 (s, 3H).

Spectroscopic data are in agreement with literature.[37]

Synthesis of 1-nitro-4-((4-(trifluoromethyl)phenyl)ethynyl)benzene (3r)

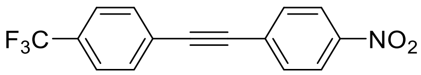

Following *GP10*. Prepared from bis(triphenylphosphine)palladium(II) dichloride (38 mg, 0.054 mmol), copper(I) iodide (20 mg, 0.11 mmol), 4-iodobenzotrifluoride (**1k**) (276 mg, 1.01 mmol), 1-ethynyl-4-nitrobenzene (**2b**) (176 mg, 1.20 mmol), triethylamine (0.976 mL, 7.00 mmol), DMF (2 mL). Yield: 75 mg (0.258 mmol, 26 %) of yellow solid.

^{1}H NMR (500 MHz, $CDCl_3$) δ = 8.25 (dt, J = 8.9, 2.1 Hz, 2H), 7.70 (dt, J = 8.9, 2.1 Hz, 2H), 7.68–7.63 (m, 4H).

^{19}F{^{1}H} NMR (470 MHz, $CDCl_3$) δ = −62.9 (s).

Spectroscopic data are in agreement with literature.[38]

Synthesis of 1-methoxy-4-((4-(trifluoromethyl)phenyl)ethynyl)benzene (3s)

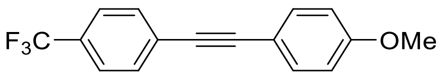

Following *GP10*. Prepared from bis(triphenylphosphine)palladium(II) dichloride (37 mg, 0.053 mmol), copper(I) iodide (20 mg, 0.11 mmol), 4-iodobenzotrifluoride (**1k**) (278 mg, 1.02 mmol), 4-ethynylanisole (**2h**) (0.156 mL, 1.20 mmol), triethylamine (0.976 mL, 7.00 mmol), DMF (2 mL). Yield: 134 mg (0.485 mmol, 49 %) of white solid.

^{1}H NMR (500 MHz, $CDCl_3$) δ = 7.63–7.57 (m, 4H), 7.49 (d, J = 8.9 Hz, 2H), 6.90 (d, J = 8.9 Hz, 2H), 3.84 (s, 3H).

^{19}F{^{1}H} NMR (470 MHz, $CDCl_3$) δ = −62.7 (s).

Spectroscopic data are in agreement with literature.[39]

2.1.4. Transmetallation reactions

The transmetallation reactions of Figure 6 were performed in a sealed NMR tube at 298 K. The conversions to the corresponding products were determined by ^{1}H NMR by comparing the integrals of the characteristic resonances of the starting compounds and the desired product.

General procedure 11 (*GP11*) – Transmetallation reactions between palladium oxidative addition complexes 7 and palladium bisacetylides 6.

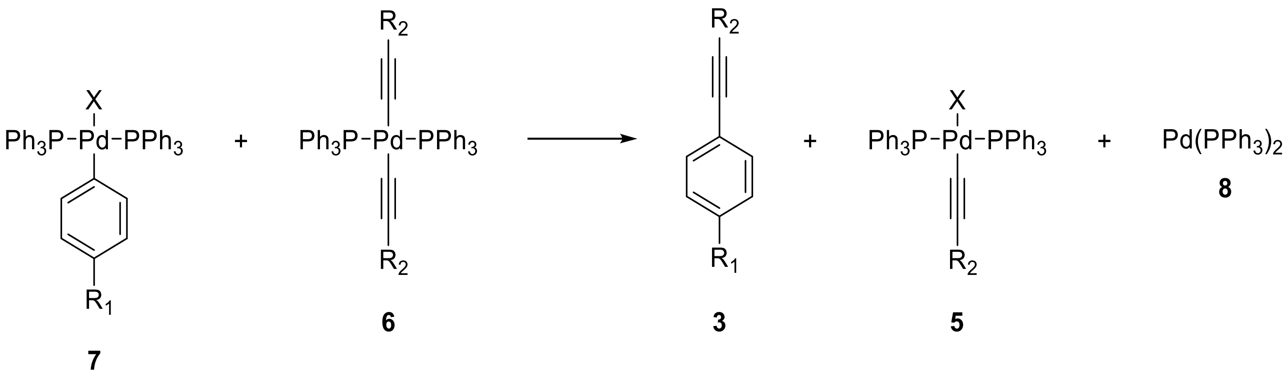

A solution of bis(triphenylphosphine)palladium aryl halide **7** (1 equiv., 0.01 M) in $CDCl_3$ was prepared in a vial under argon atmosphere. Bis(triphenylphosphine)palladium bisacetylide **6** (1 equiv.) was added to this solution in one portion. The reaction mixture was sonicated for 0.5 min and transferred to an NMR tube, purged with argon and sealed. NMR spectra were recorded in 2.5 min intervals.

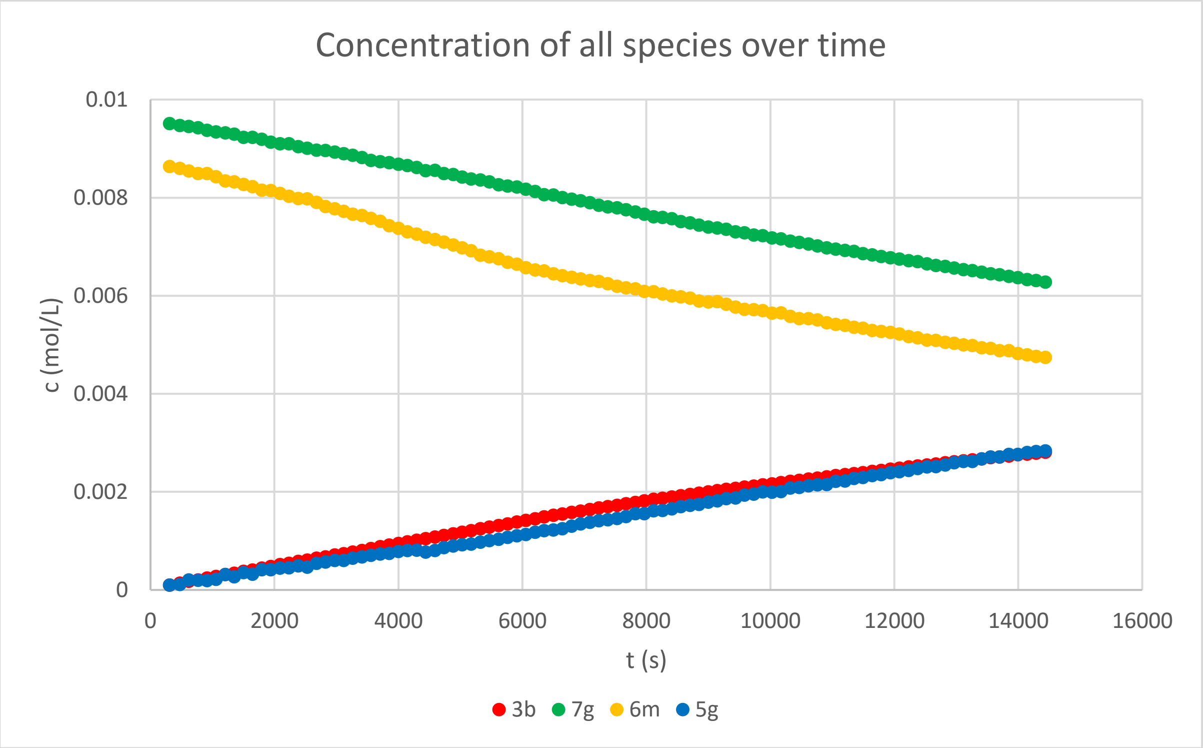

Figure 6a. Plot of concentration over time for species **3b**, **5g**, **6m** and **7g**.

Transmetallation of 7b and 6a

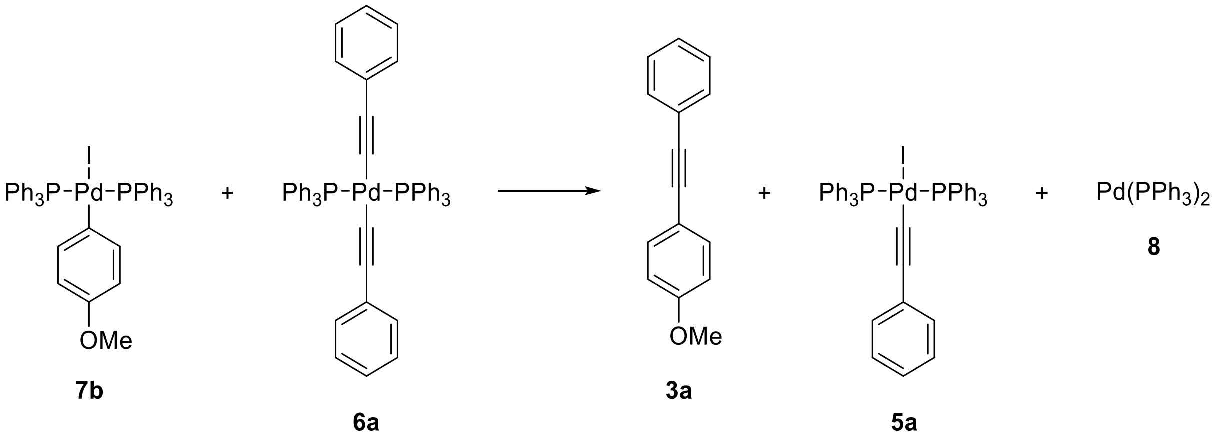

Following *GP11* using bis(triphenylphosphine)palladium(II) (4-methoxyphenyl)ide iodide (**7b**) (6.0 mg, 6.9 µmol), bis(triphenylphosphine)palladium(II) bis(phenylethynide) (**6a**) (5.9 mg, 7.1 µmol), $CDCl_3$ (0.700 mL).

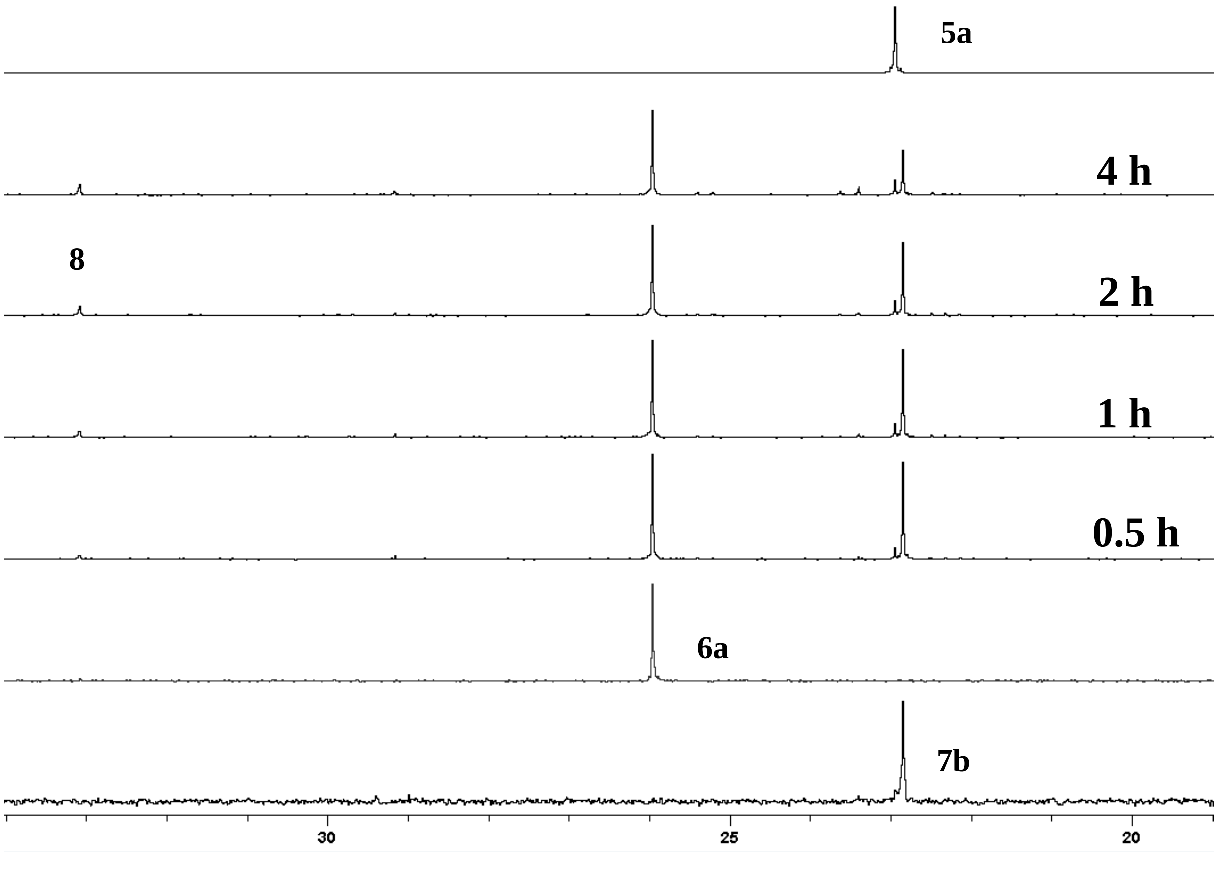

Supplementary Figure 1. Stack of $^{31}P\{^1H\}$ NMR spectra for transmetallation of **7b** and **6a**.

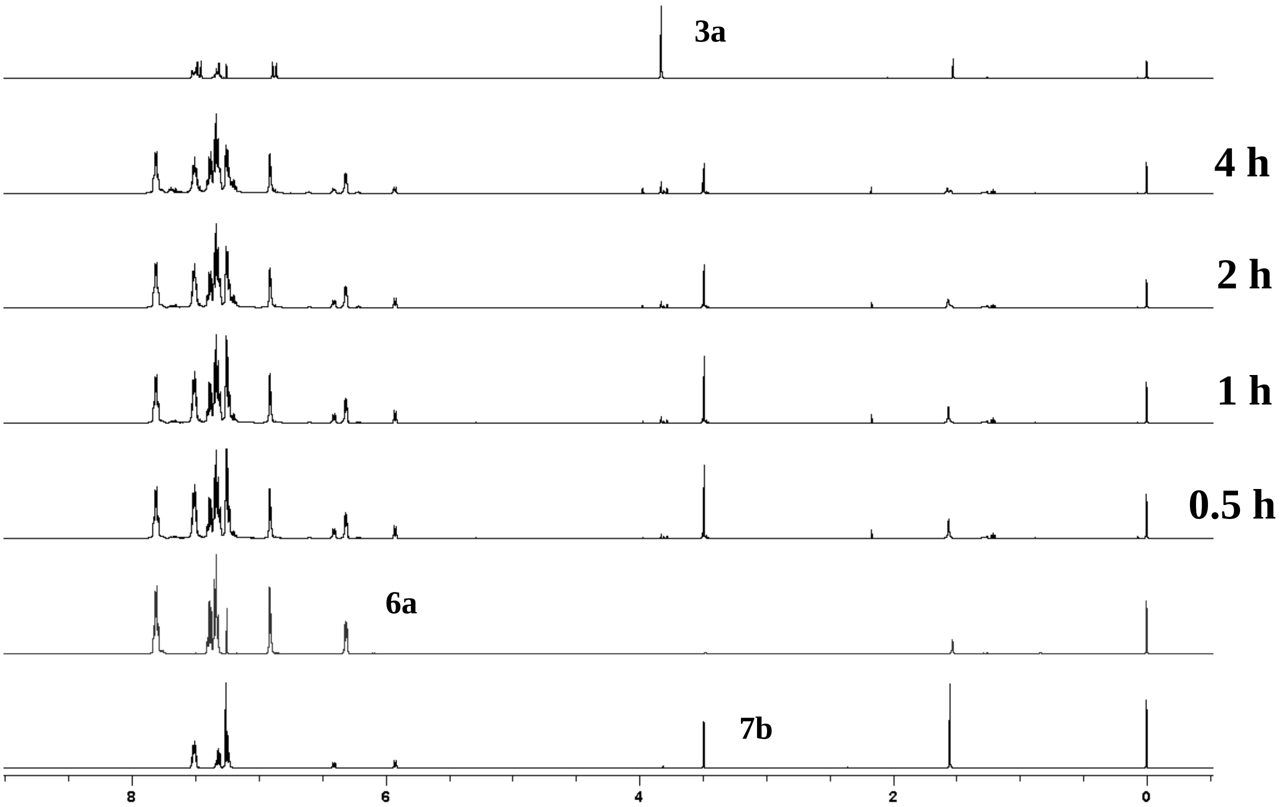

Supplementary Figure 2. Stack of ^{1}H NMR spectra for transmetallation of **7b** and **6a**.

Transmetallation of 7e and 6a

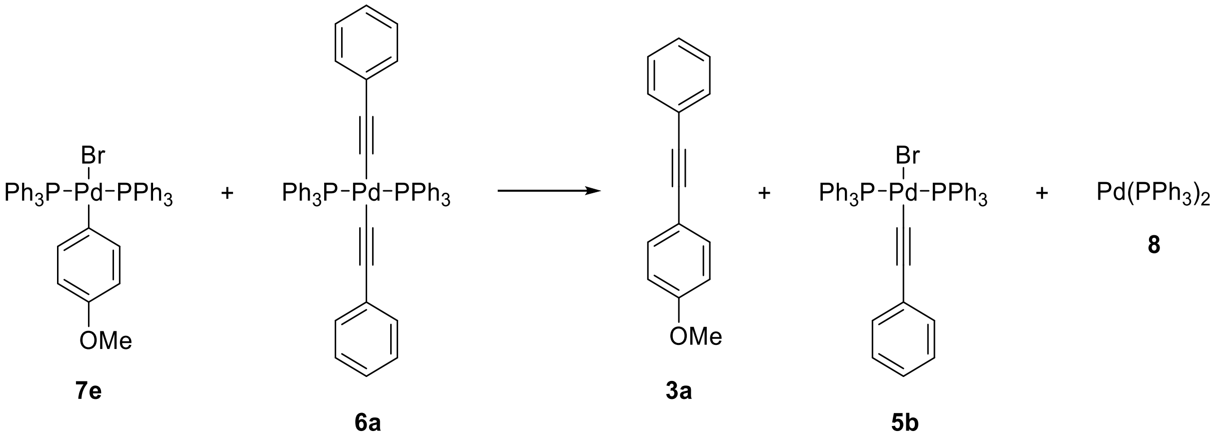

Following *GP11* using bis(triphenylphosphine)palladium(II) (4-methoxyphenyl)ide bromide (**7e**) (5.9 mg, 7.2 μmol), bis(triphenylphosphine)palladium(II) bis(phenylethynide) (**6a**) (6.1 mg, 7.3 μmol), $CDCl_3$ (0.700 mL).

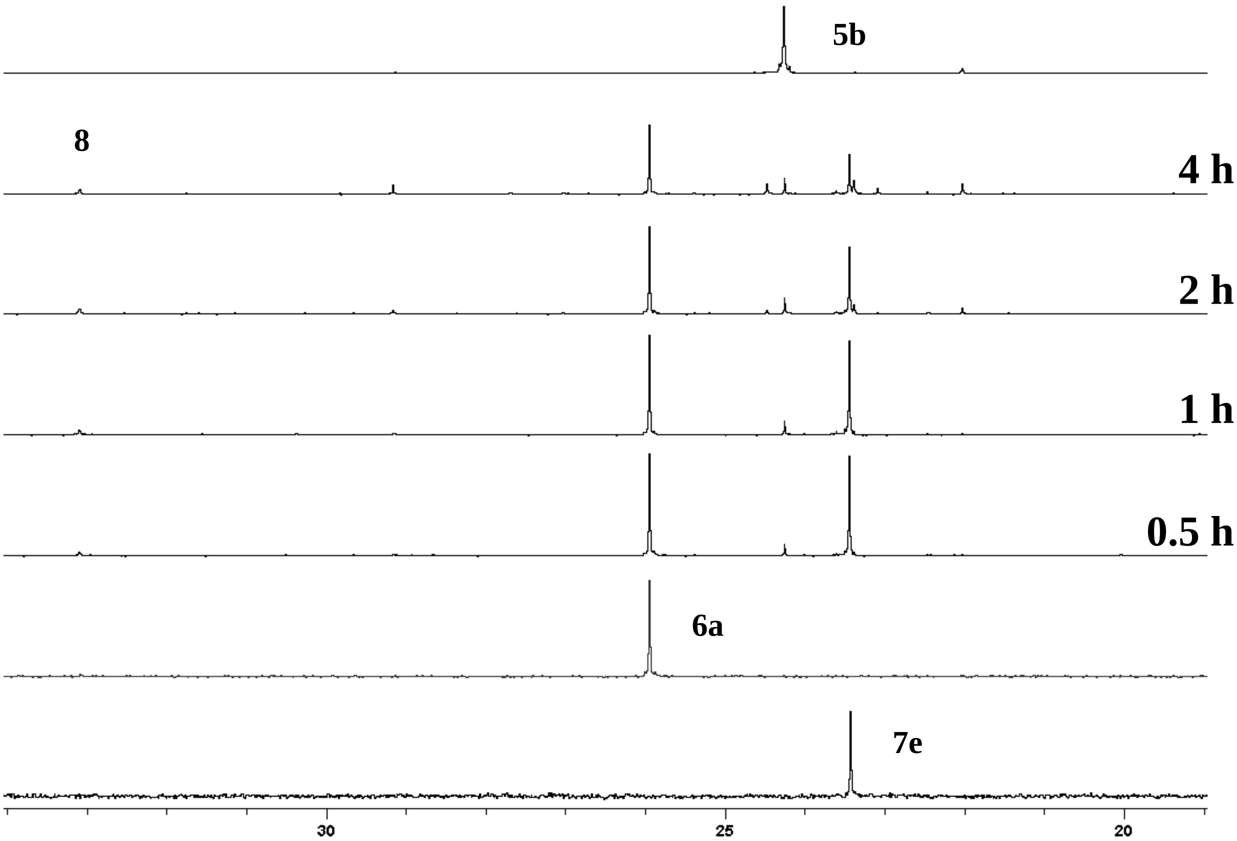

Supplementary Figure 3. Stack of $^{31}P\{^{1}H\}$ NMR spectra for transmetallation of **7e** and **6a**.

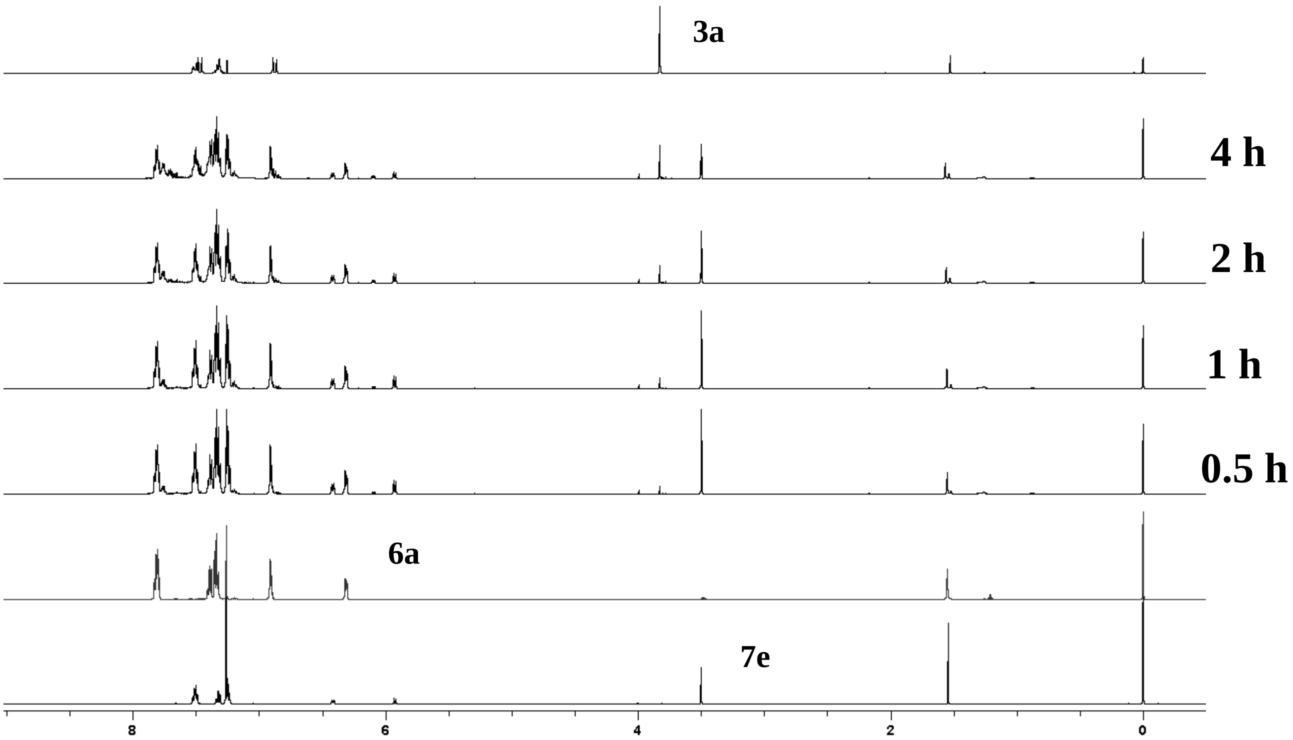

Supplementary Figure 4. Stack of ^{1}H NMR spectra for transmetallation of **7e** and **6a**.

Transmetallation of 7h and 6a

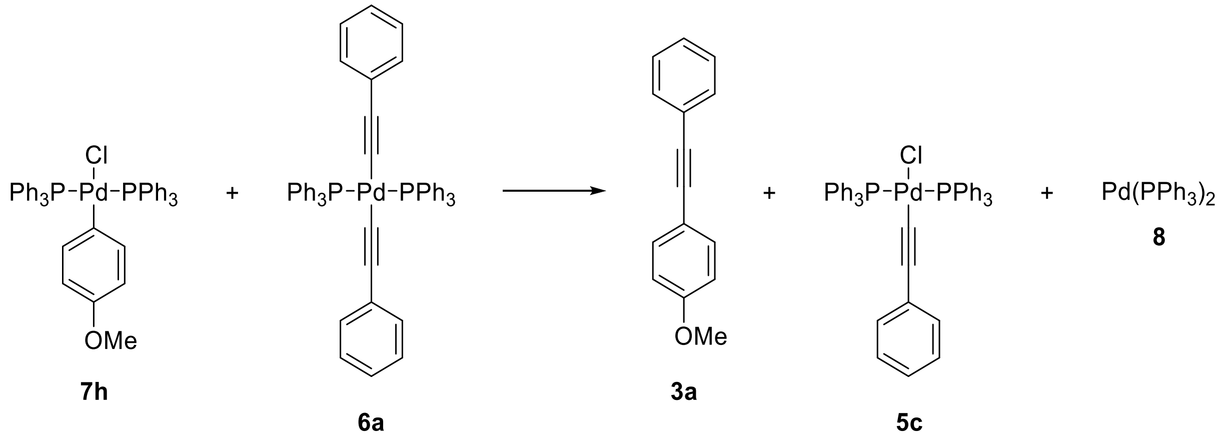

Following *GP11* using bis(triphenylphosphine)palladium(II) (4-methoxyphenyl)ide chloride (**7h**) (5.5 mg, 7.1 μmol), bis(triphenylphosphine)palladium(II) bis(phenylethynide) (**6a**) (5.8 mg, 7.0 μmol), $CDCl_3$ (0.700 mL).

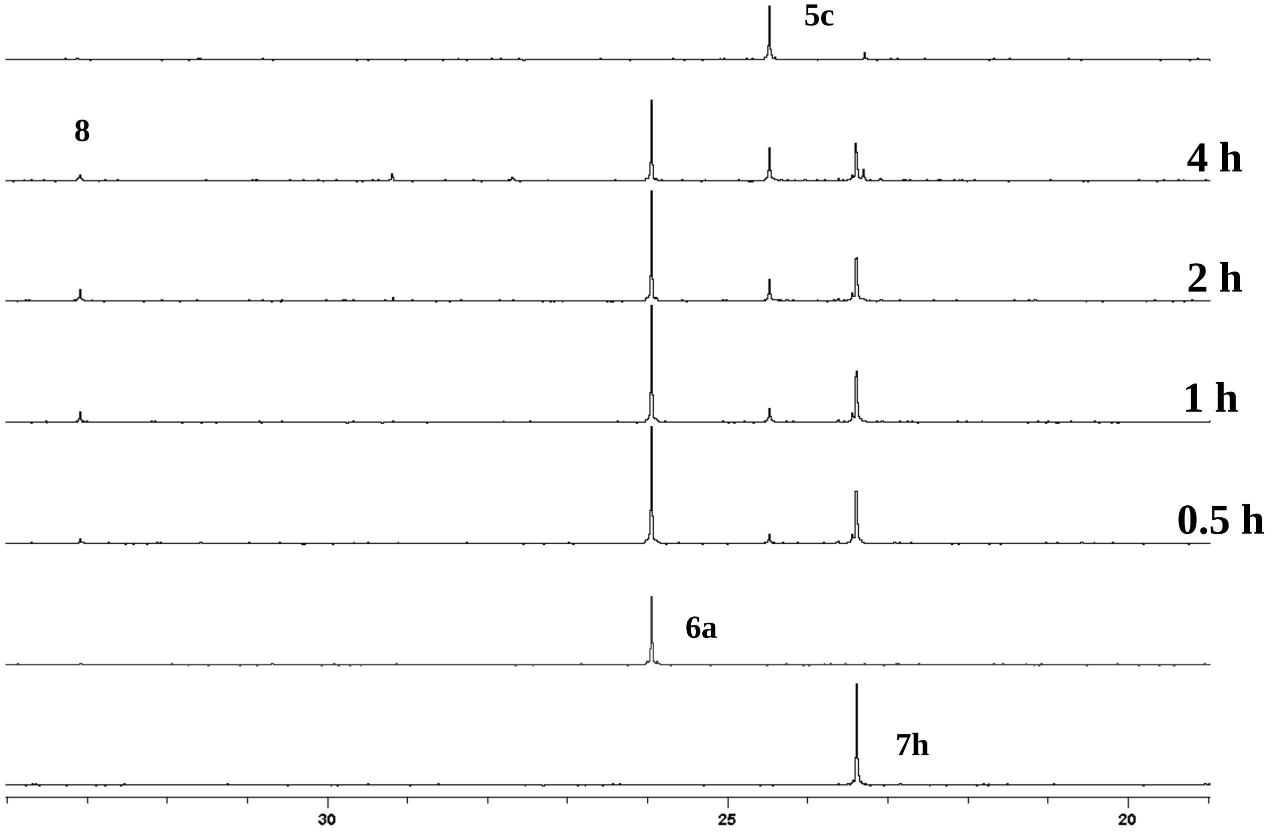

Supplementary Figure 5. Stack of $^{31}P\{^1H\}$ NMR spectra for transmetallation of **7h** and **6a**.

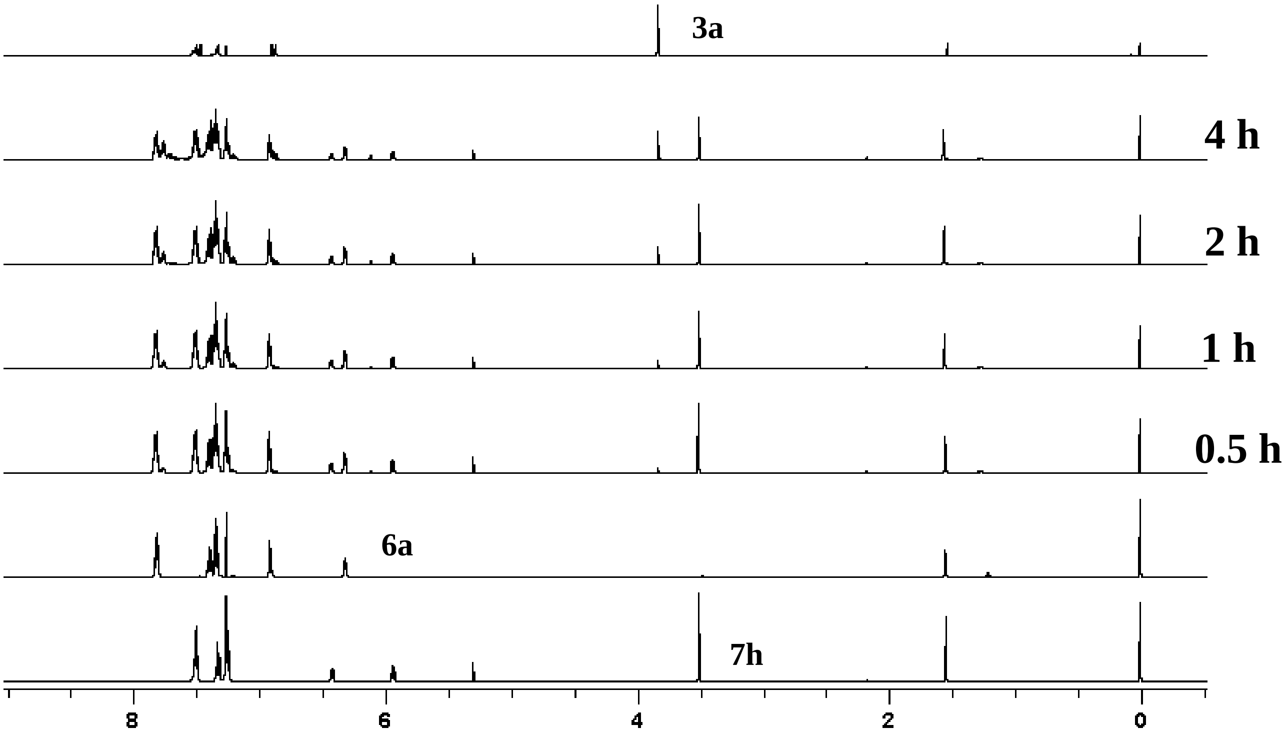

Supplementary Figure 6. Stack of ^{1}H NMR spectra for transmetallation of **7h** and **6a**.

Transmetallation of 7g and 6m

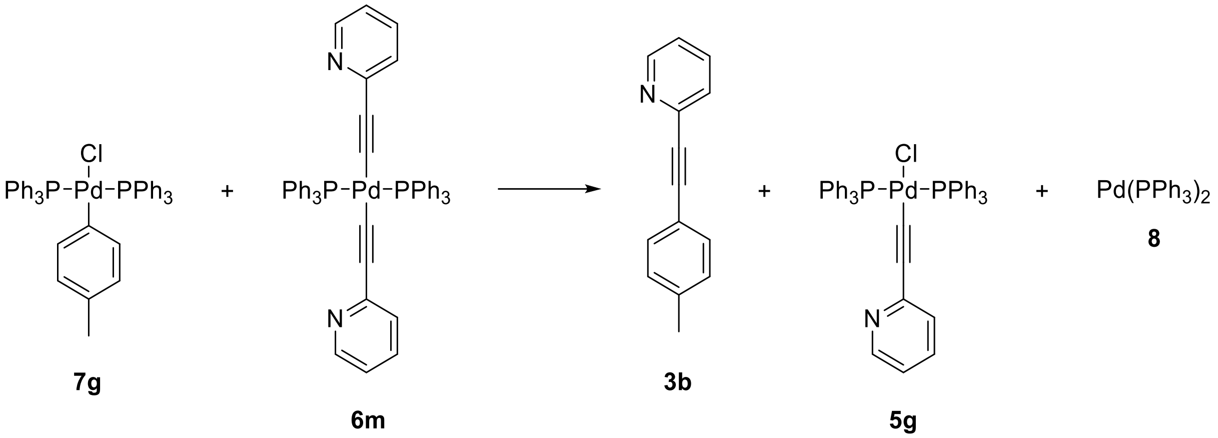

Following *GP11* using bis(triphenylphosphine)palladium(II) (4-methylphenyl)ide chloride (**7g**) (5.2 mg, 6.9 µmol), bis(triphenylphosphine)palladium(II) bis((2-pyridyl)ethyn-1-ide) (**6m**) (5.9 mg, 7.0 µmol), $CDCl_3$ (0.700 mL).

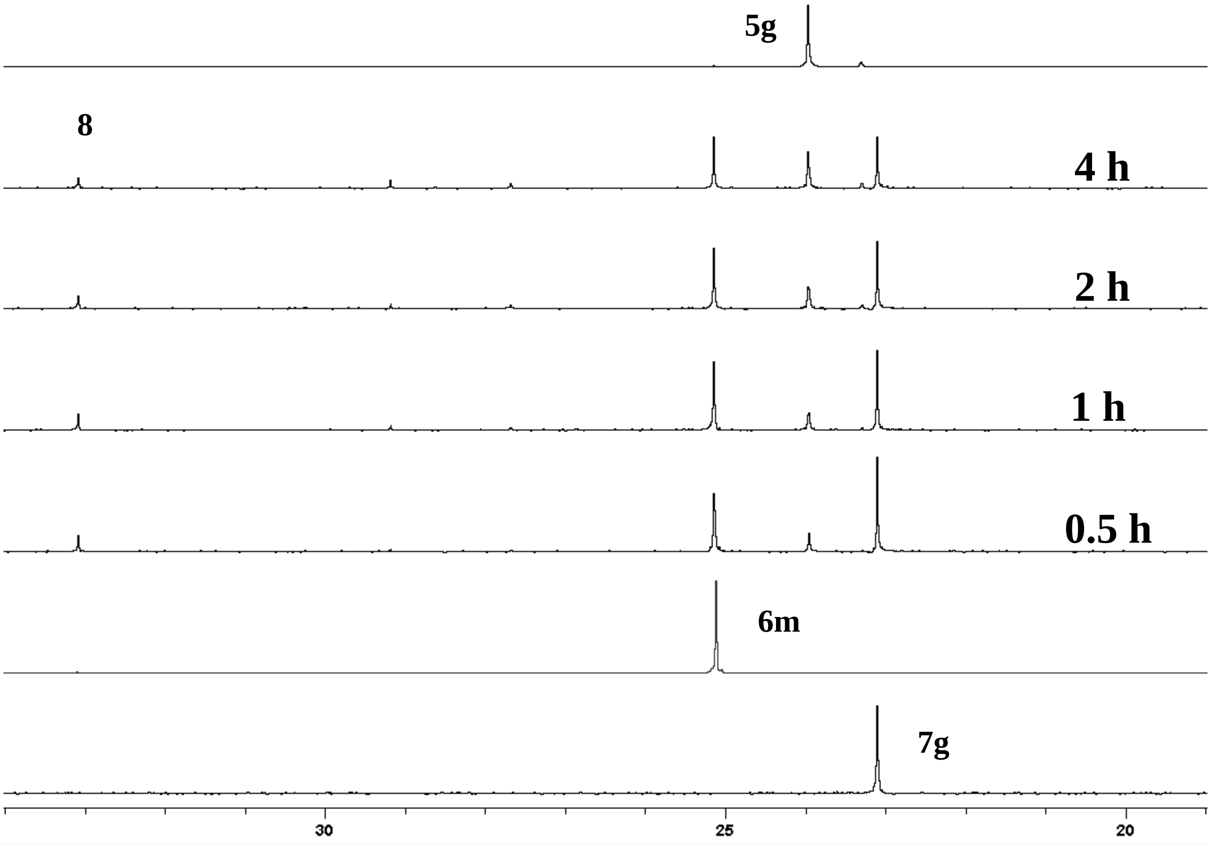

Supplementary Figure 7. Stack of $^{31}P\{^1H\}$ NMR spectra for transmetallation of **7g** and **6m**.

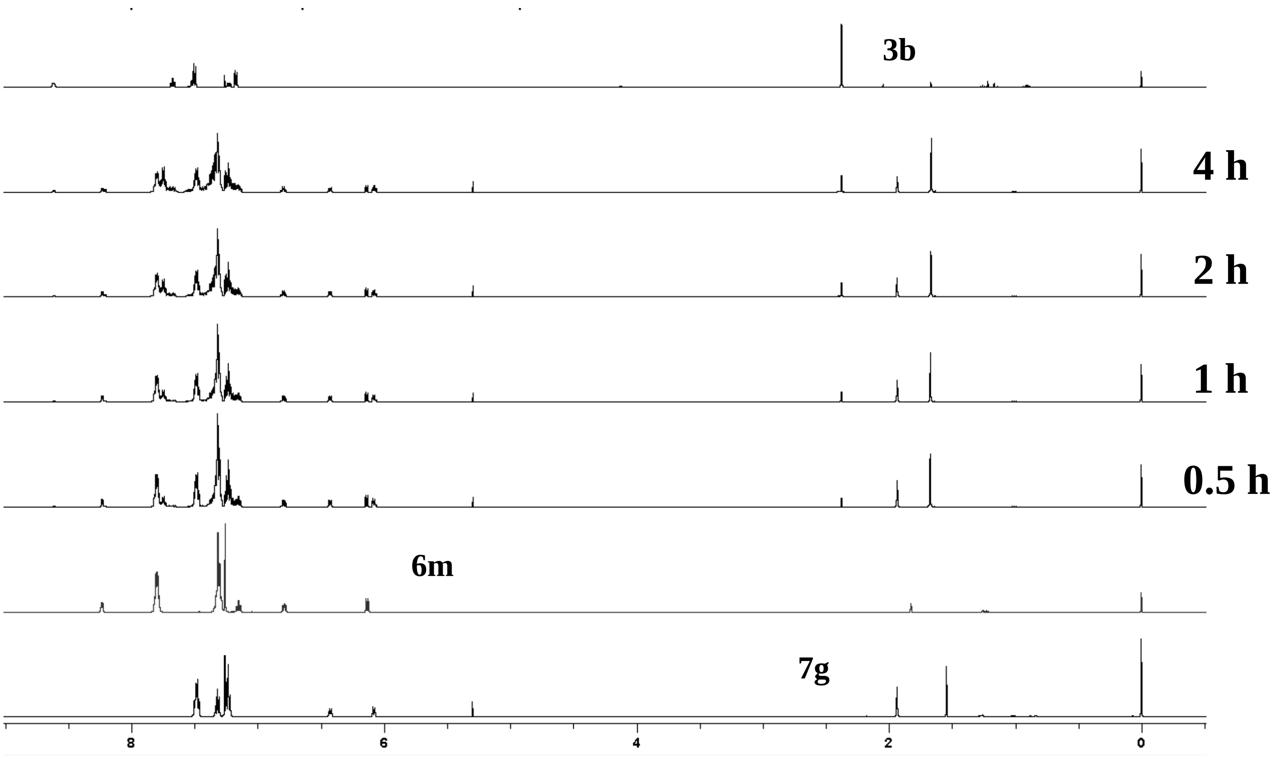

Supplementary Figure 8. Stack of ^{1}H NMR spectra for transmetallation of **7g** and **6m**.

Transmetallation of 7a and 6k

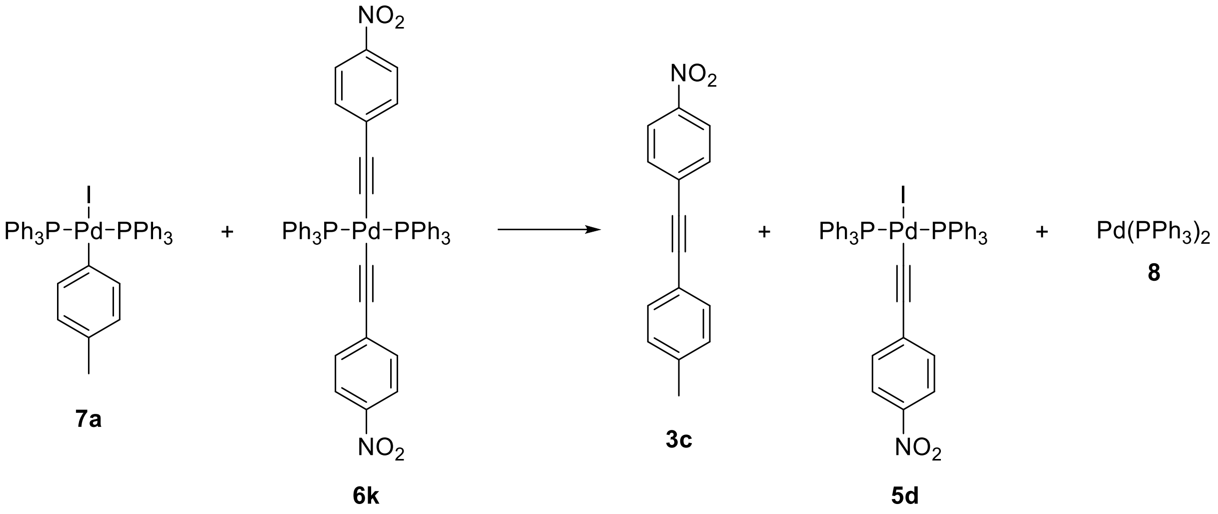

Following *GP11* using bis(triphenylphosphine)palladium(II) (4-methylphenyl)ide iodide (**7a**) (6.0 mg, 7.1 µmol), bis(triphenylphosphine)palladium(II) bis((4-nitrophenyl)ethyn-1-ide) (**6k**) (6.6 mg, 7.2 µmol), $CDCl_3$ (0.700 mL).

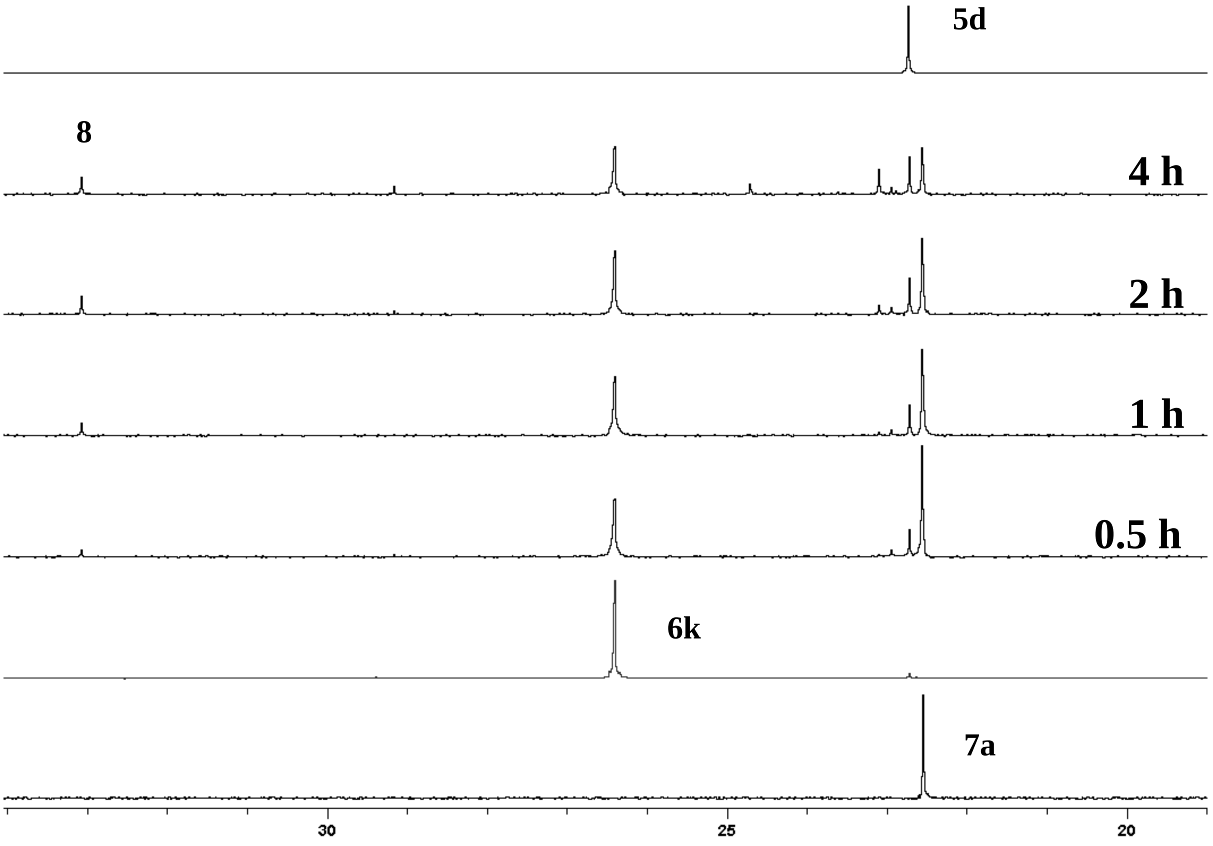

Supplementary Figure 9. Stack of $^{31}P\{^1H\}$ NMR spectra for transmetallation of **7a** and **6k**.

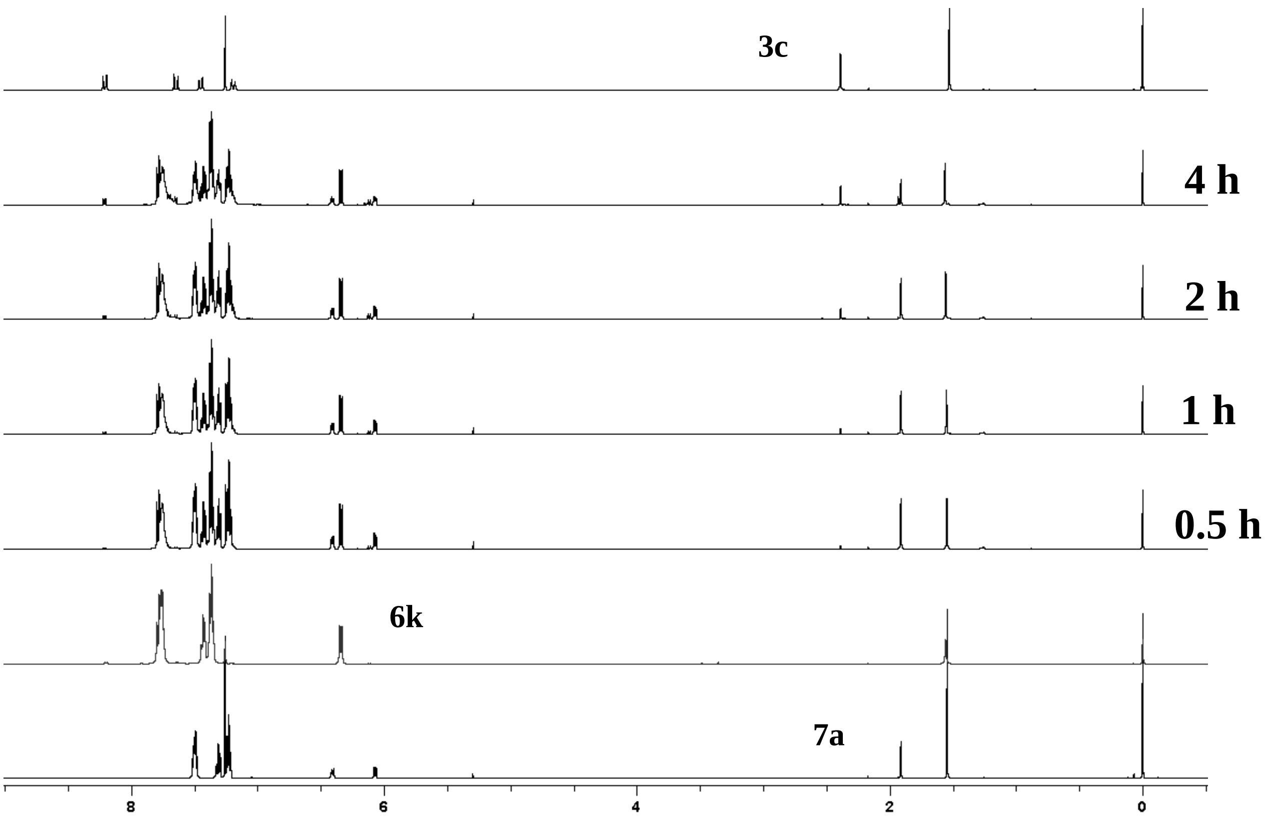

Supplementary Figure 10. Stack of ^{1}H NMR spectra for transmetallation of **7a** and **6k**.

Transmetallation of 7a and 6p

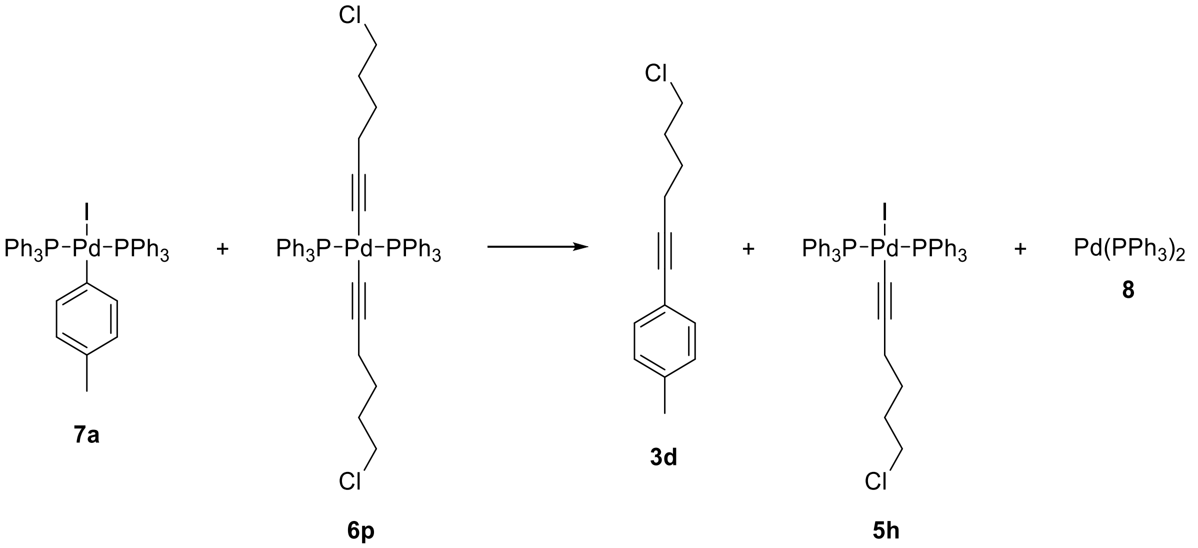

Following *GP11* using bis(triphenylphosphine)palladium(II) (4-methylphenyl)ide iodide (**7a**) (6.1 mg, 7.2 μmol), bis(triphenylphosphine)palladium(II) bis(6-chlorohexyn-1-ide) (**6p**) (6.8 mg, 7.9 μmol), $CDCl_3$ (0.700 mL).

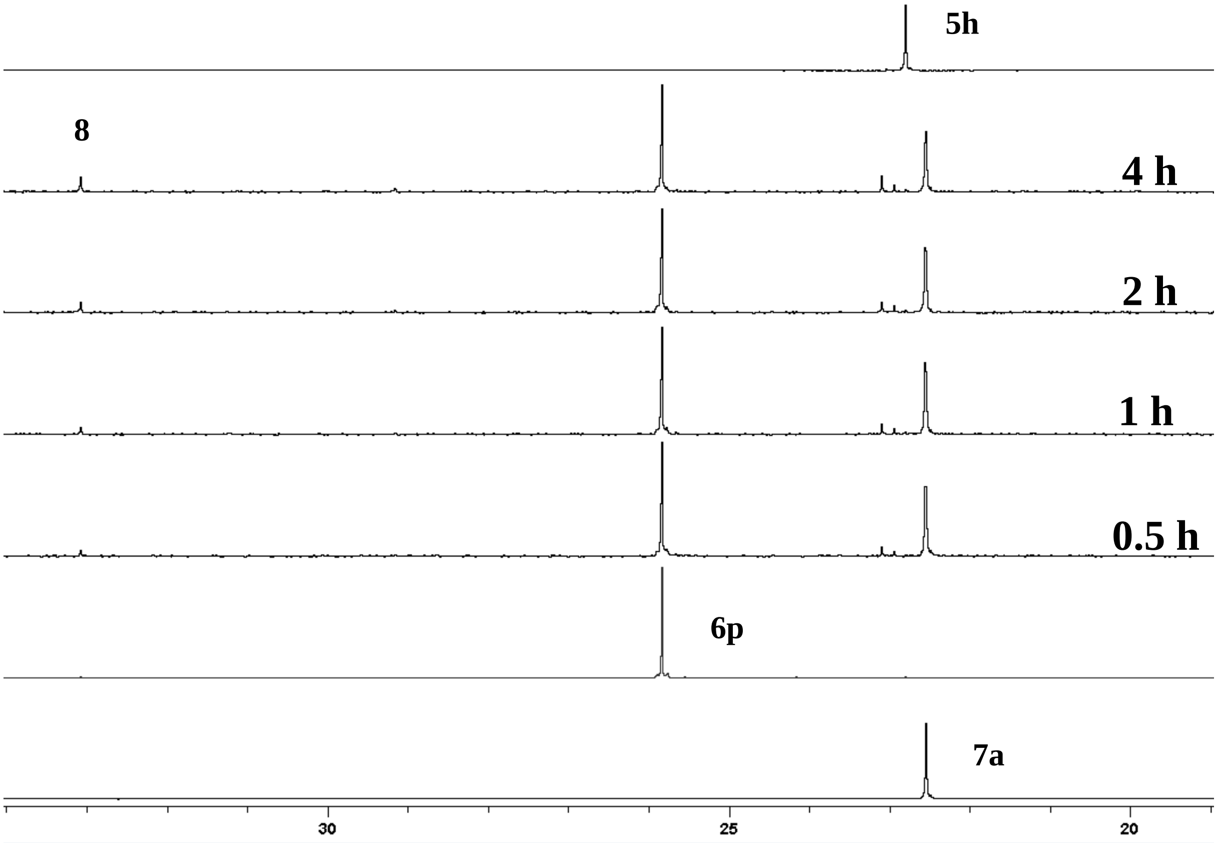

Supplementary Figure 11. Stack of $^{31}P\{^1H\}$ NMR spectra for transmetallation of **7a** and **6p**.

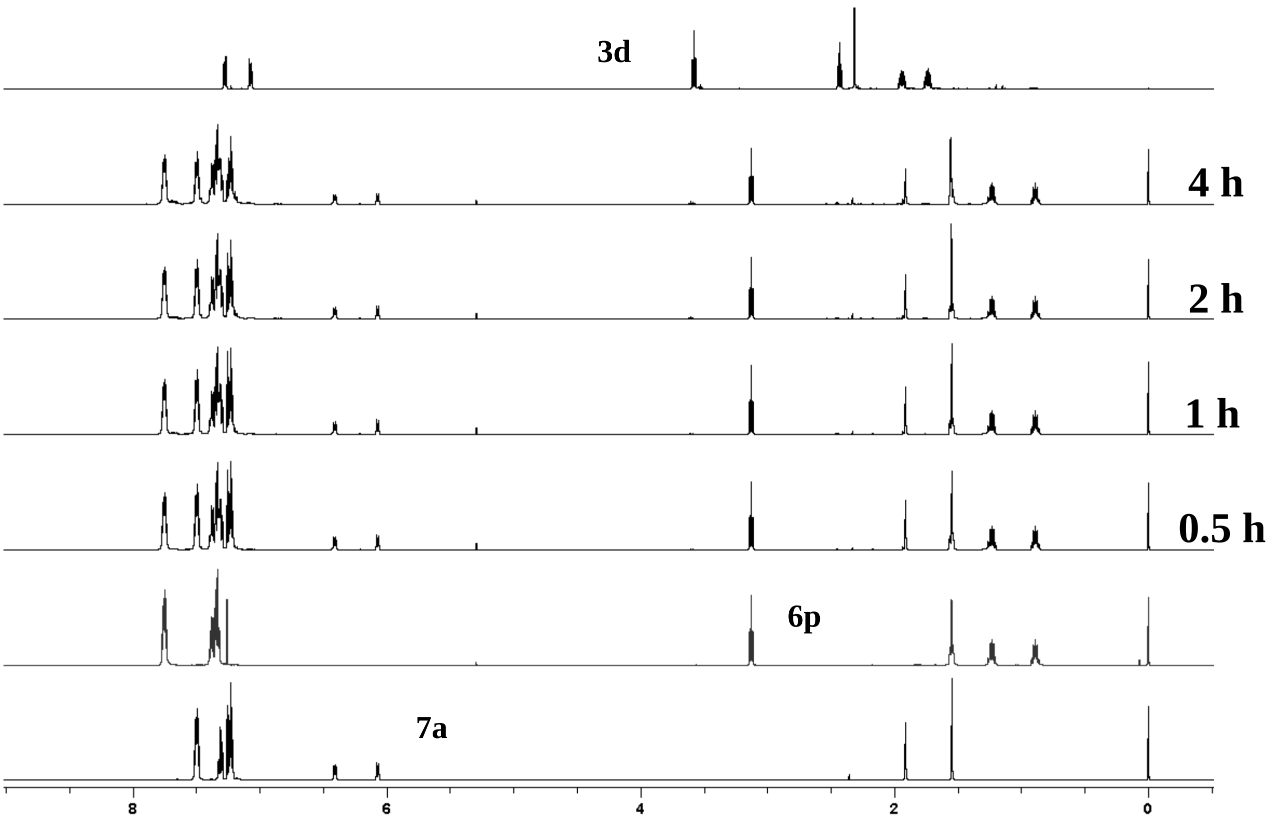

Supplementary Figure 12. Stack of ^{1}H NMR spectra for transmetallation of **7a** and **6p**.

Table of determined conversions (t = 2 hours) (Figure 6c)

R_2—≡—Pd(PPh_3)$_2$—≡—R_2		6d (tBu)	6c (Me)	6a	6f (Cl)	6k (NO_2)	6n	6m	6q	6p
Pd(PPh_3)I, R_1	(7a), CH_3	6	12	6	9	22	9	38	9	9
	(7b), OCH_3	2	7	6	12	5	4	44	12	8
	(7c), NO_2	6	7	4	3	4	12	40	3	4
Pd(PPh_3)Br, R_1	(7d), CH_3	20	27	24	24	38	38	56	36	6
	(7e), OCH_3	35	31	26	30	37	37	61	38	9
	(7f), NO_2	9	5	4	3	3	9	31	11	2
Pd(PPh_3)Cl, R_1	(7g), CH_3	21	22	29	19	31	34	39	30	6
	(7h), OCH_3	29	26	21	28	37	41	38	34	11
	(7i), NO_2	7	3	2	2	2	4	34	7	1

0	10	20	30	40	50	60

Conversion %

The effect of solvent on transmetallation under study

General procedure 12 (*GP12*) – Transmetallation reaction between palladium oxidative addition complex 7a and palladium bisacetylide 6a in different solvents

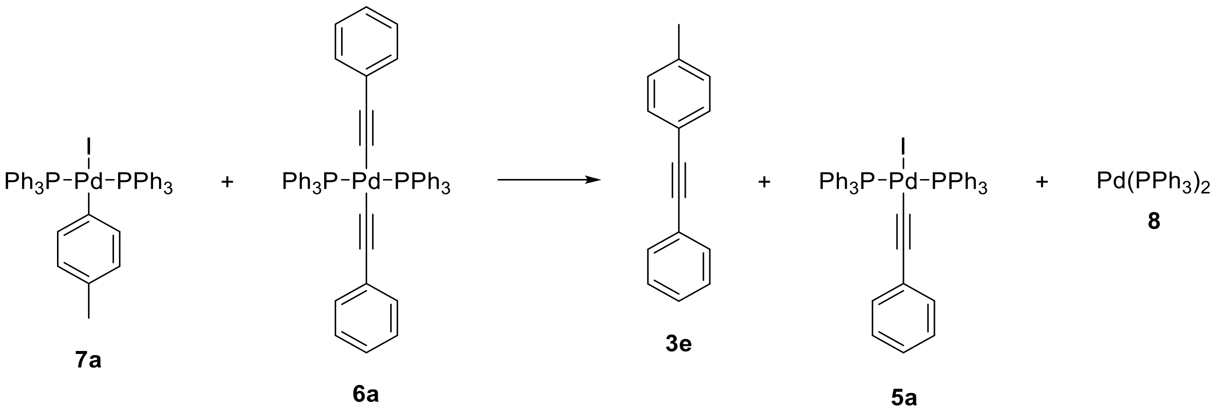

In a vial a solution of bis(triphenylphosphine)palladium (4-methylphenyl)ide iodide **7a** (1 equiv., 0.01 M) in CD_2Cl_2, $CDCl_3$ or THF-d_8 was prepared under argon atmosphere. Bis(triphenylphosphine)palladium bis(phenylethynide) (**6a**) (1.0 equiv.) was added to this solution in one portion. The reaction mixture was sonicated for 0.5 min and transferred to NMR tube, flushed with argon and sealed. NMR spectra were acquired in 2.5 min intervals. The concentration of the product **3e** was determined by comparison of integrals of characteristic resonances of product **3e** with integrals of resonances of an internal standard (1,3,5-trimethoxybenzene).

Transmetallation of 6a and 7a in CD_2Cl_2

Following *GP12* using bis(triphenylphosphine)palladium(II) (4-methylphenyl)ide iodide (**7a**) (6.34 mg, 7.47 µmol), bis(triphenylphosphine)palladium(II) bis(phenylethynide) (**6a**) (6.27 mg, 7.52 µmol), CD_2Cl_2 (0.750 mL).

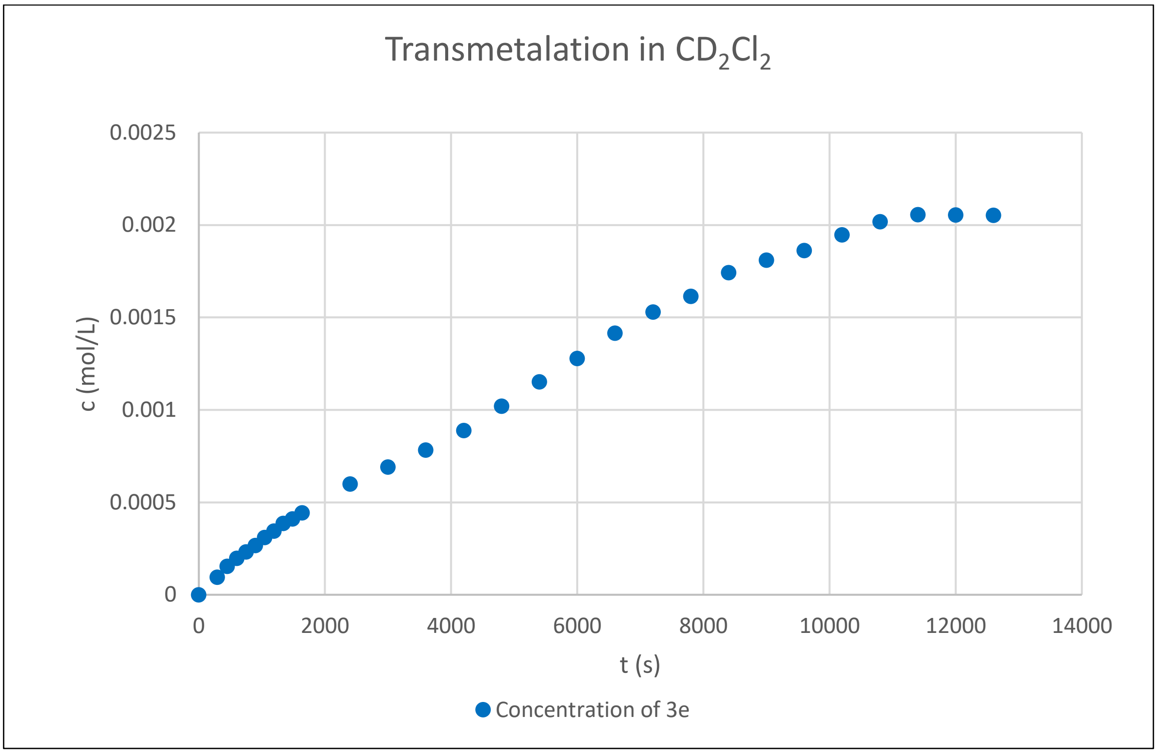

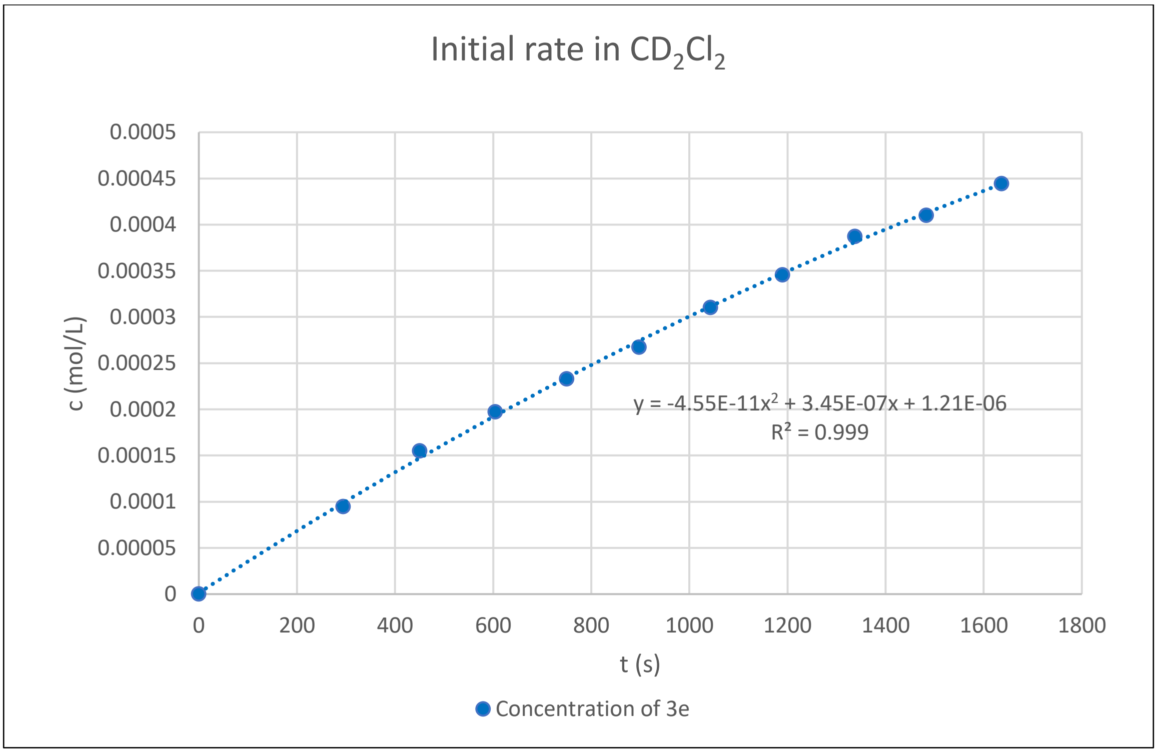

Fitted curve:

$c = A_0 + A_1 \times t + A_2 \times t^2$

A_0	1.21E-06
A_1	3.45E-07
A_2	-4.55E-11

v_0 (CD_2Cl_2) = $(3.5 \pm 0.1) \times 10^{-7}$ mol/Ls

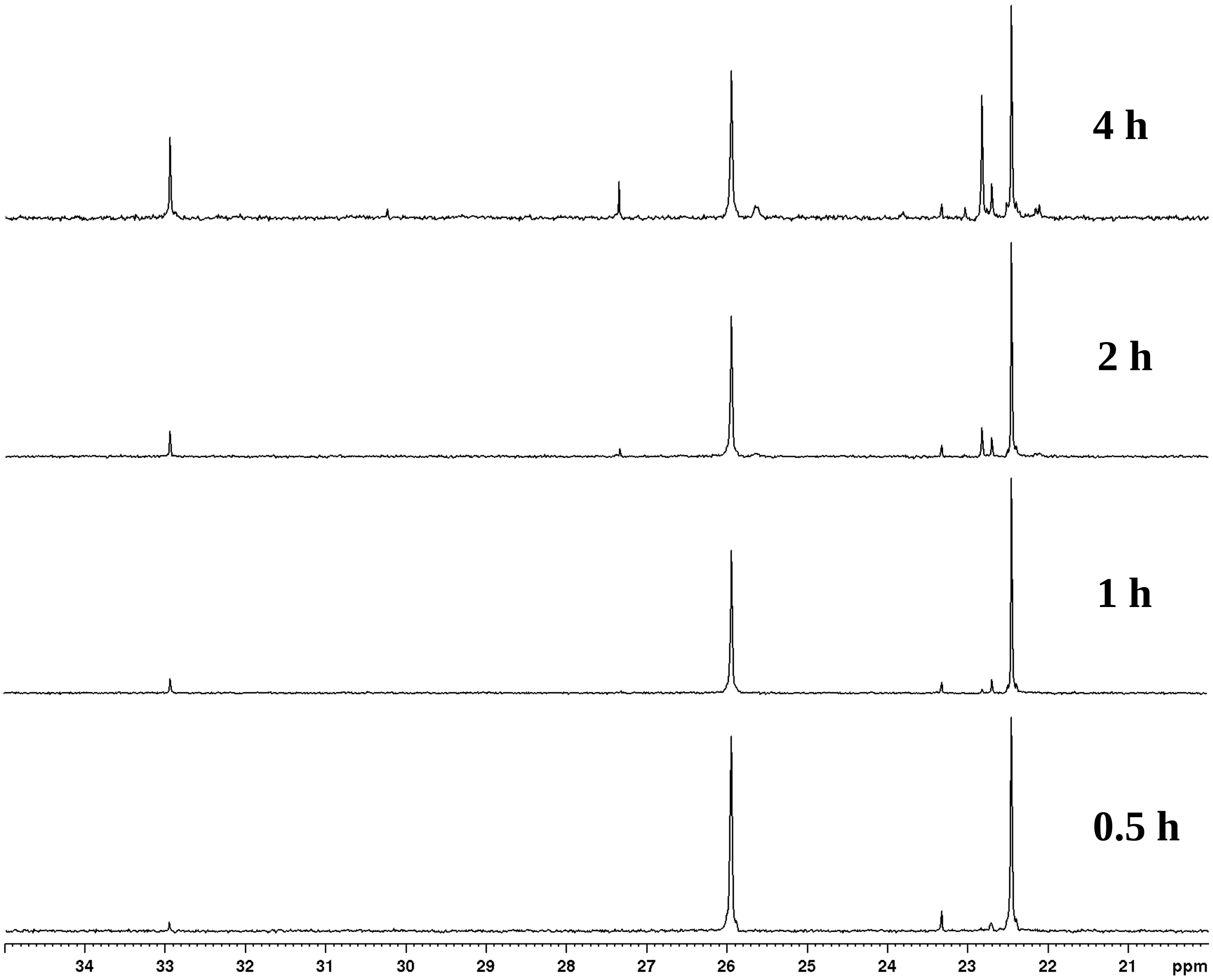

Supplementary Figure 13. Stack of $^{31}P\{^1H\}$ NMR spectra after 0.5 h, 1 h, 2 h and 4 h. After 2 h different byproducts begin to form.

Transmetallation of 6a and 7a in $CDCl_3$

Following *GP12* using bis(triphenylphosphine)palladium(II) (4-methylphenyl)ide iodide (**7a**) (6.78 mg, 7.99 µmol), bis(triphenylphosphine)palladium(II) bis(phenylethynide) (**6a**) (6.65 mg, 7.98 µmol), $CDCl_3$ (0.800 mL).

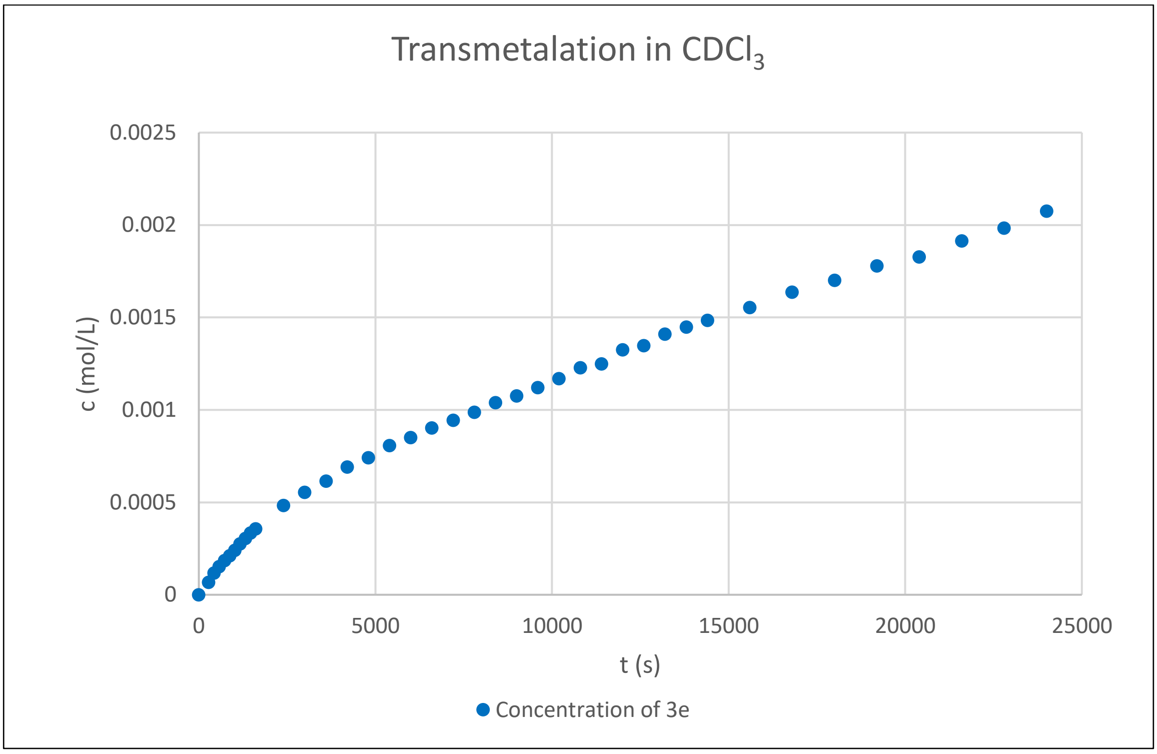

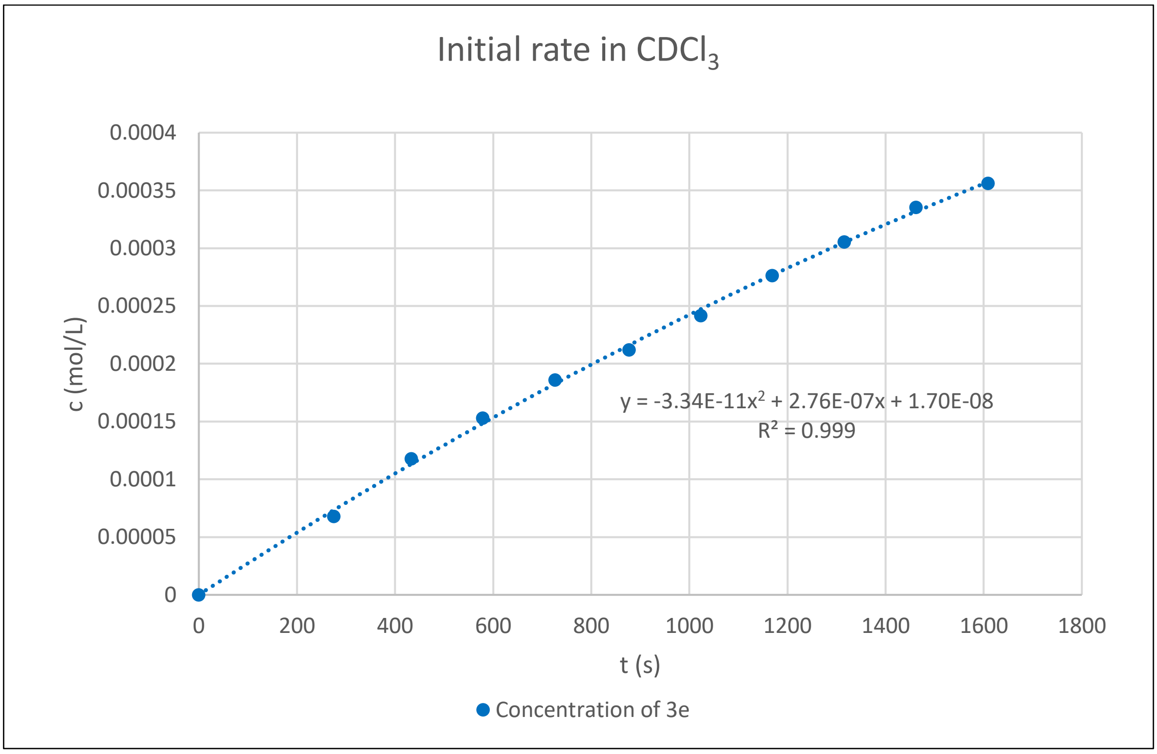

Fitted curve:

$c = A_0 + A_1 \times t + A_2 \times t^2$

A_0	1.70E-08
A_1	2.76E-07
A_2	-3.34E-11

v_0 ($CDCl_3$) = $(2.8 \pm 0.1) \times 10^{-7}$ mol/Ls

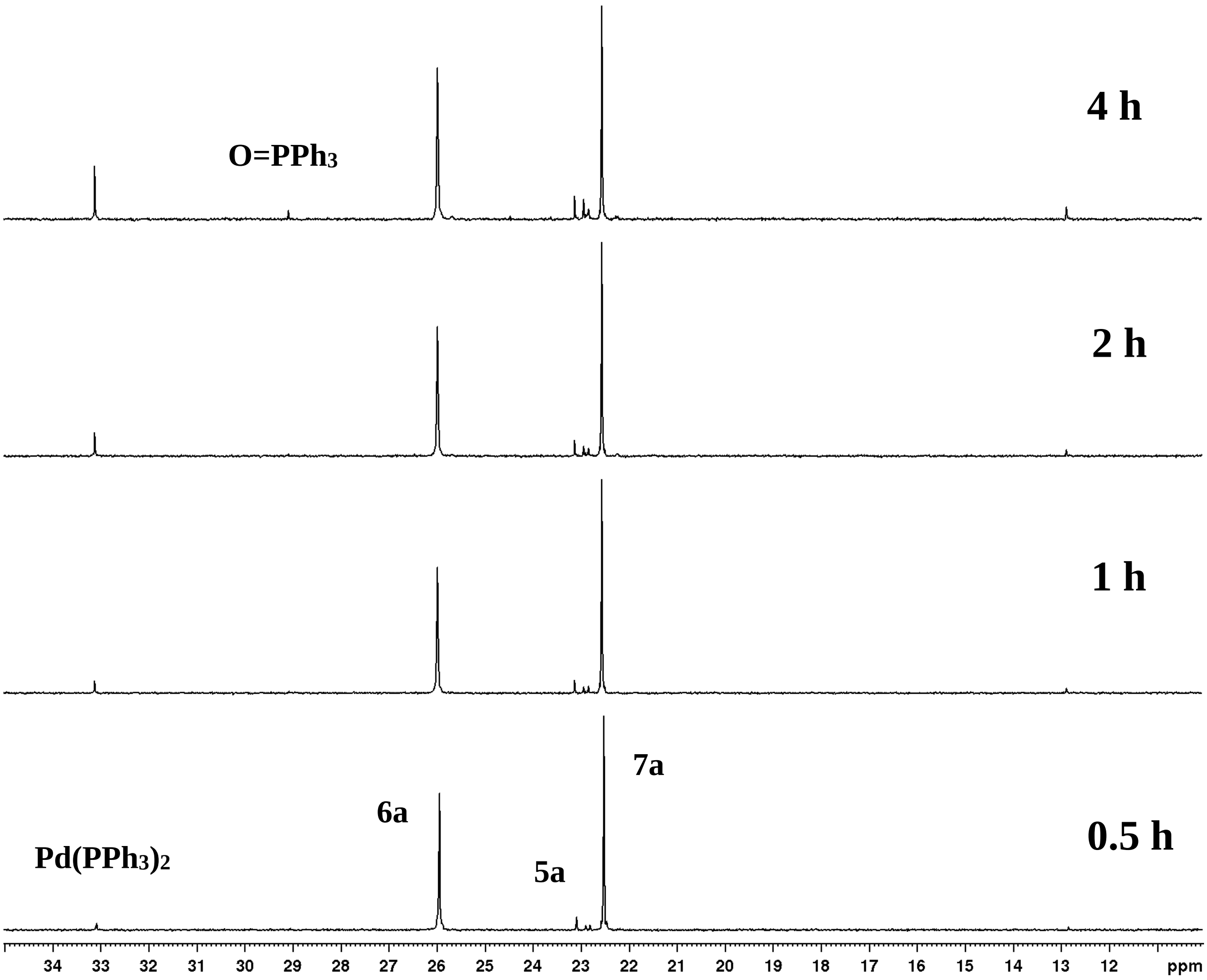

Supplementary Figure 14. Stack of $^{31}P\{^{1}H\}$ NMR spectra after 0.5 h, 1 h, 2 h and 4 h.

Transmetallation of 6a and 7a in $CDCl_3$ (0.04 M concentration in 6a and 7a)

Following *GP12* using bis(triphenylphosphine)palladium(II) (4-methylphenyl)ide iodide (**7a**) (27.25 mg, 32.10 μmol), bis(triphenylphosphine)palladium(II) bis(phenylethynide) (**6a**) (26.79 mg, 32.15 μmol), $CDCl_3$ (0.800 mL).

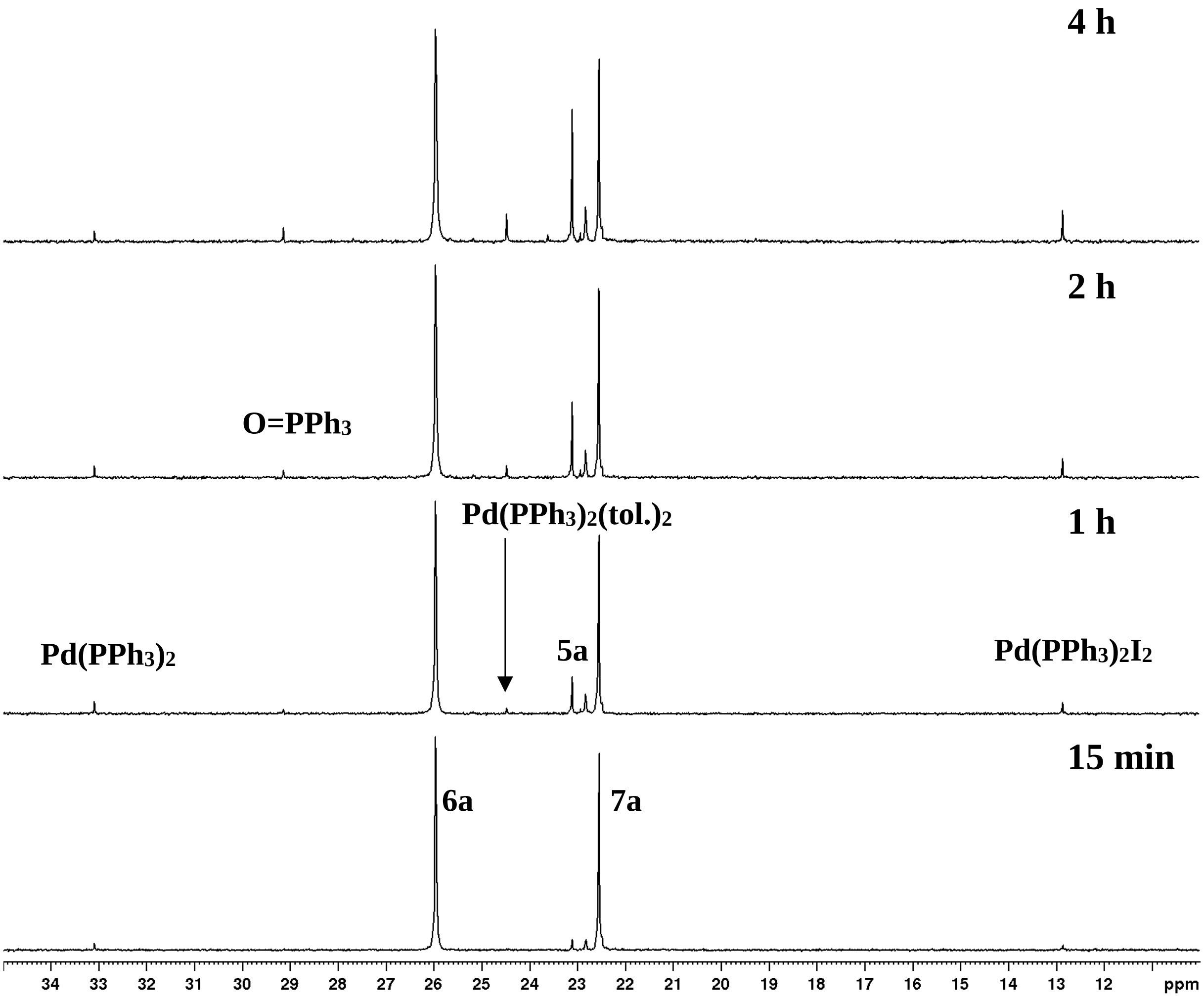

Supplementary Figure 15. Stack of $^{31}P\{^1H\}$ NMR spectra after 0.25 h, 1 h, 2 h and 4 h.

Transmetallation of 6a and 7a in THF-d_8

Following *GP12* using bis(triphenylphosphine)palladium(II) (4-methylphenyl)ide iodide (**7a**) (6.34 mg, 7.47 µmol), bis(triphenylphosphine)palladium(II) bis(phenylethynide) (**6a**) (6.19 mg, 7.43 µmol), THF-d_8 (0.750 mL).

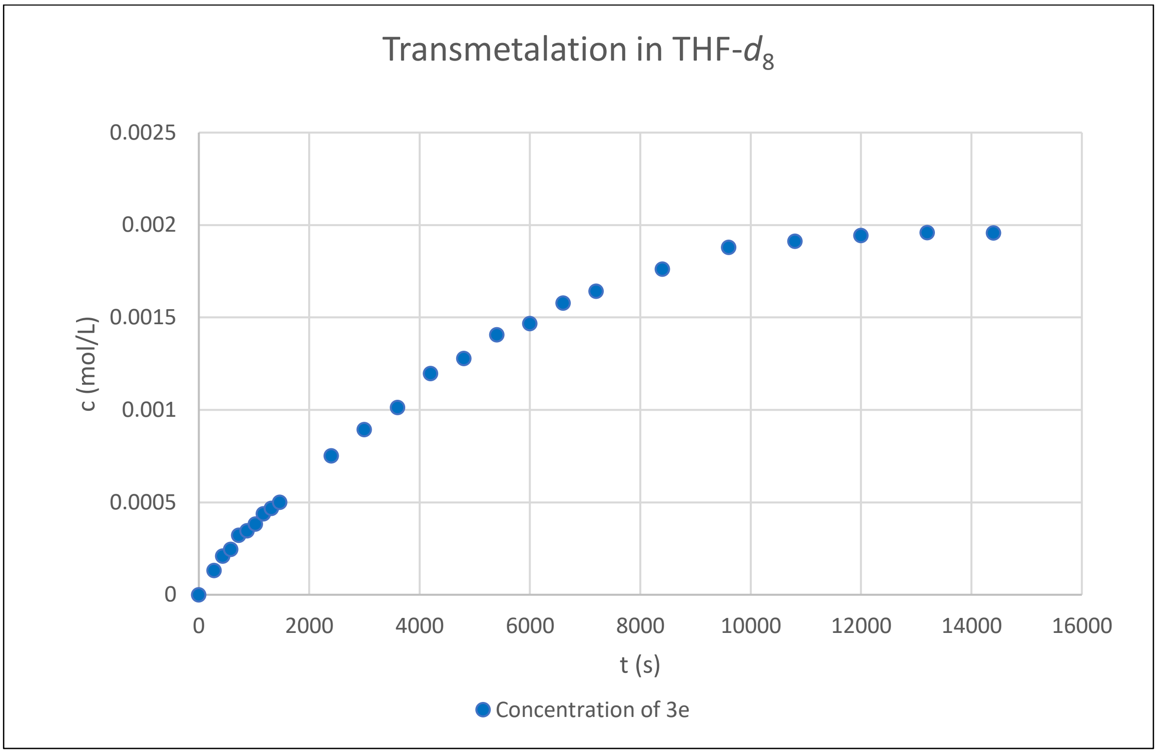

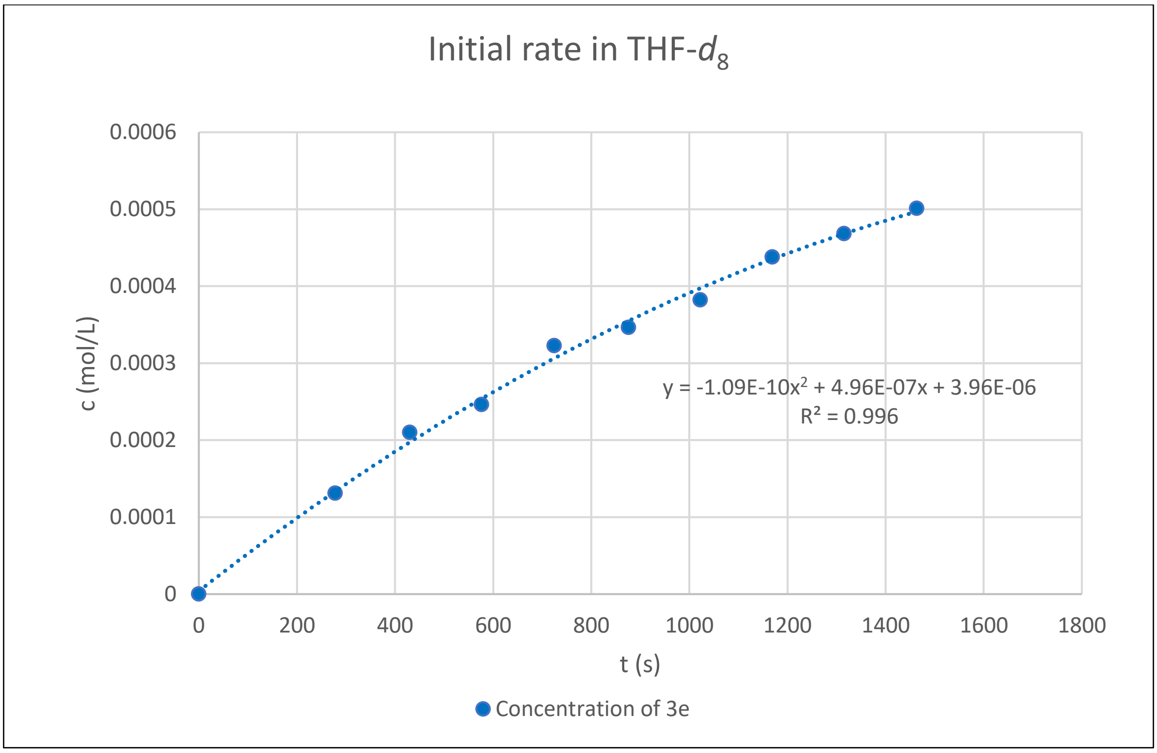

Fitted curve:

$c = A_0 + A_1 \times t + A_2 \times t^2$

A_0	3.96E-06
A_1	4.96E-07
A_2	-1.09E-10

v_0 (THF-d_8) = $(5.0 \pm 0.3) \times 10^{-7}$ mol/Ls

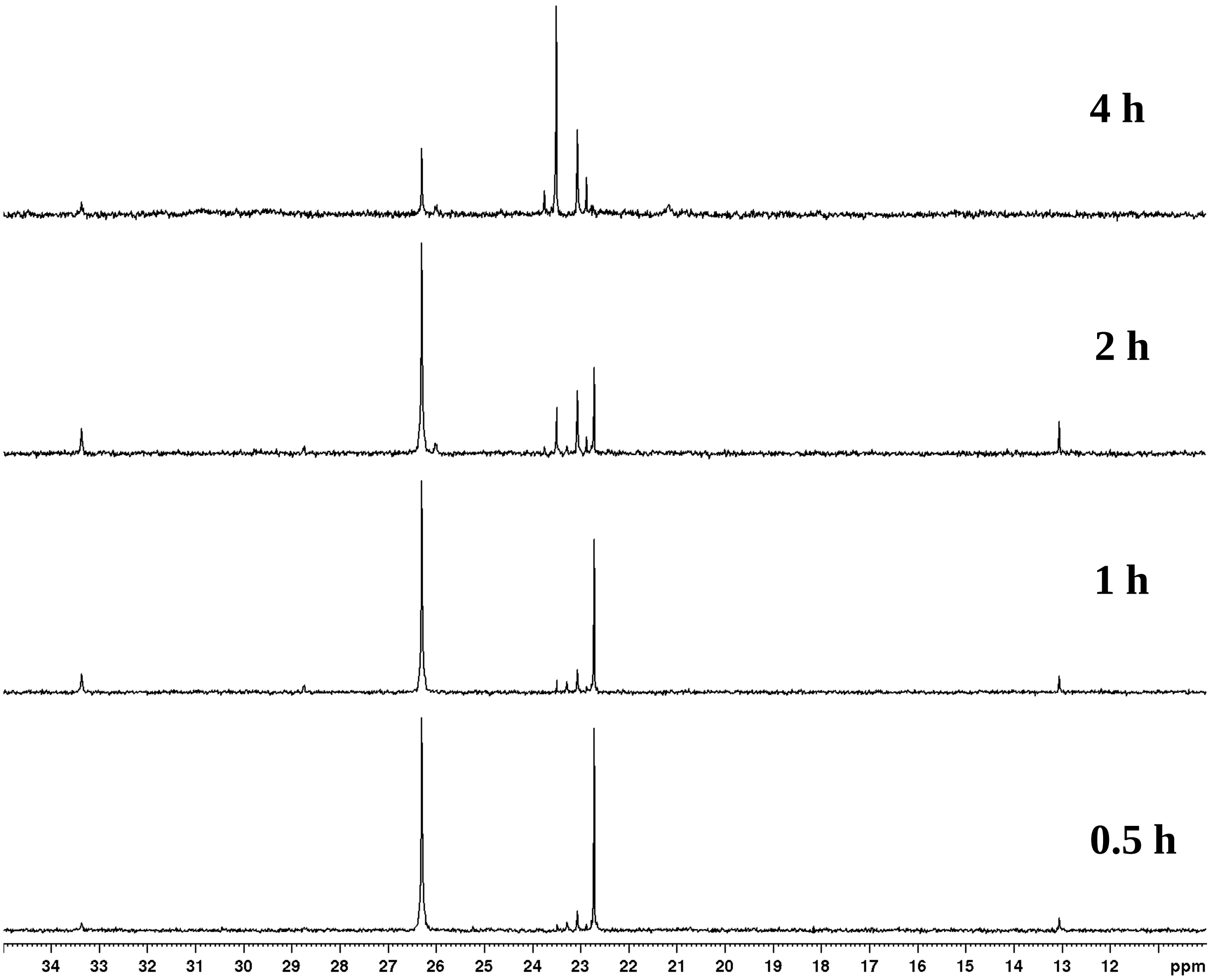

Supplementary Figure 16. Stack of $^{31}P\{^{1}H\}$ NMR spectra of reaction between **6a** and **7a** in THF-d_8 after 0.5 h, 1 h, 2 h and 4 h. Soon after onset of the reaction (approx. 0.5 h) byproducts begin to form.

Comparison of transmetallation rates in different solvents

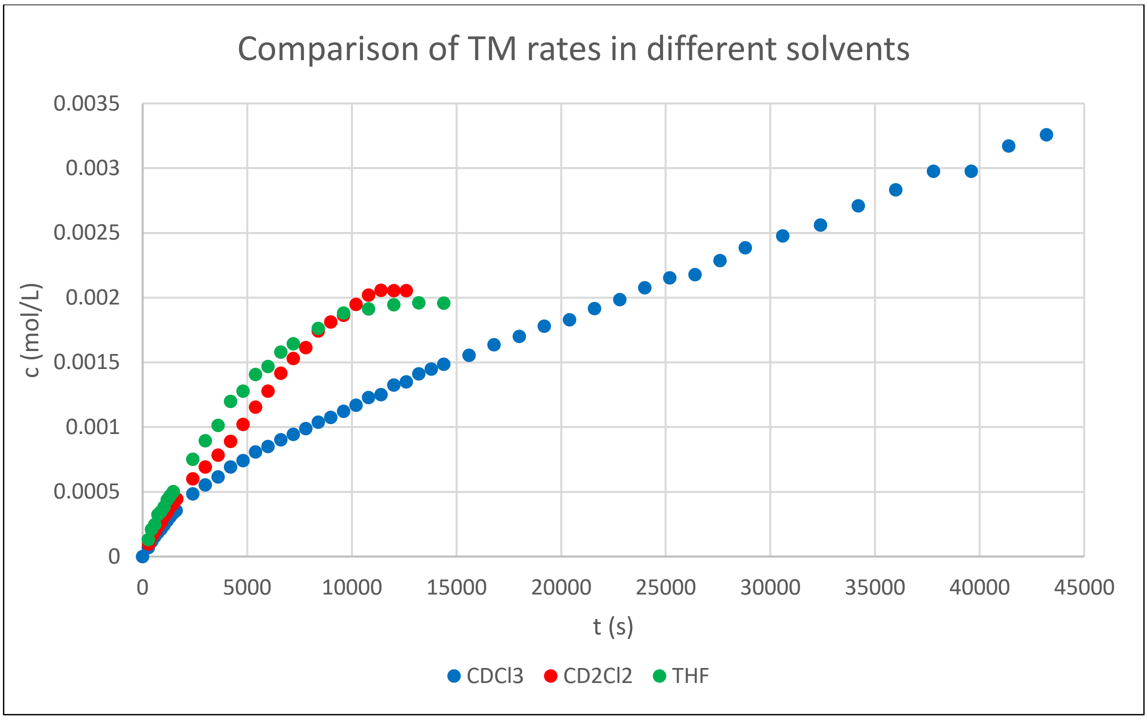

Supplementary Figure 17. Comparison of rates (reaction profiles) of transmetallation of **6a** and **7a** in different deuterated solvents.

Comment on transmetallation reaction of 6a and 7a in different solvents:

Although the initial rate of transmetallation reaction is higher in CD_2Cl_2 and THF-d_8, i.e. v_0 (CD_2Cl_2) = $(3.5 \pm 0.1) \times 10^{-7}$ mol/Ls, v_0 (THF-d_8) = $(5.0 \pm 0.3) \times 10^{-7}$ mol/Ls, than in $CDCl_3$, v_0 ($CDCl_3$) = $(2.8 \pm 0.1) \times 10^{-7}$ mol/Ls, the reactions in CD_2Cl_2 and THF-d_8 slow after 3 hours, most likely due to side products formed (see Supplementary Figs. 13 and 16). Noteworthy, the reaction in $CDCl_3$ proceeds more cleanly (see Supplementary Fig. 14), in comparison with other solvents.

The effect of halide ligands on transmetallation reactions

The transmetallation reactions were carried out in a sealed NMR tube at 302.0 K. The initial concentration of bis(triphenylphosphine)palladium(II) (4-methylphenyl)ide halide **7**, bis(triphenylphosphine)palladium(II) bis(phenylethynide) **6a**, and bis(triphenylphosphine)palladium(II) phenylethynide halide (**5**) was 0.01 M.

The ^{1}H NMR spectra were recorded at 4 min intervals. The concentration of product **3** was determined by comparing the integrals of the characteristic resonances of product **3** with the integrals of the resonances of an internal standard (1,3,5-trimethoxybenzene).

General procedure 13 (*GP13*) – Transmetallation reactions between palladium oxidative addition complexes 7 and palladium bisacetylide 6a

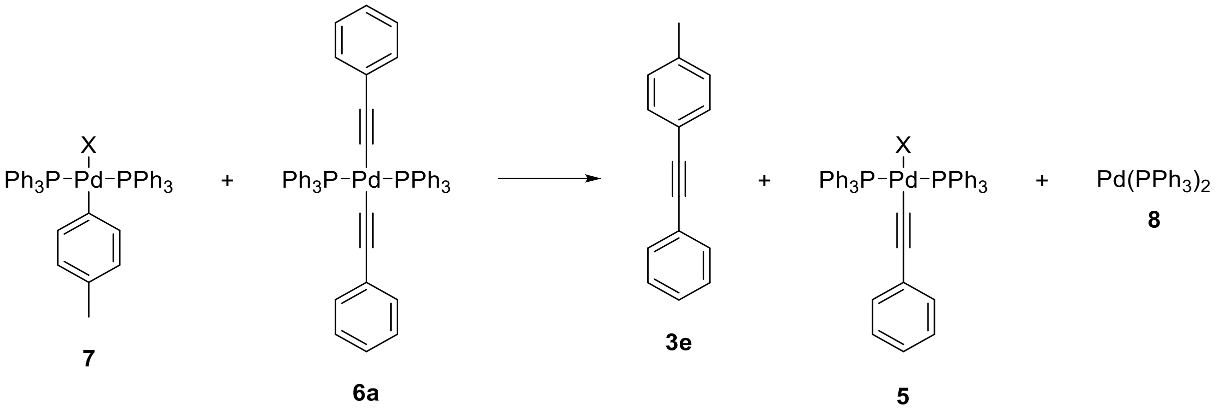

In a vial a solution of bis(triphenylphosphine)palladium (4-methylphenyl)ide halide **7** (1 equiv., 0.01 M) in $CDCl_3$ was prepared under argon atmosphere. Bis(triphenylphosphine)palladium bis(phenylethynide) (**6a**) (1.0 equiv.) was added to this solution in one portion. The reaction mixture was sonicated for 0.5 min and transferred to NMR tube, flushed with argon and sealed. NMR spectra were acquired in 4.0 min intervals.

Transmetallation of 6a and 7a

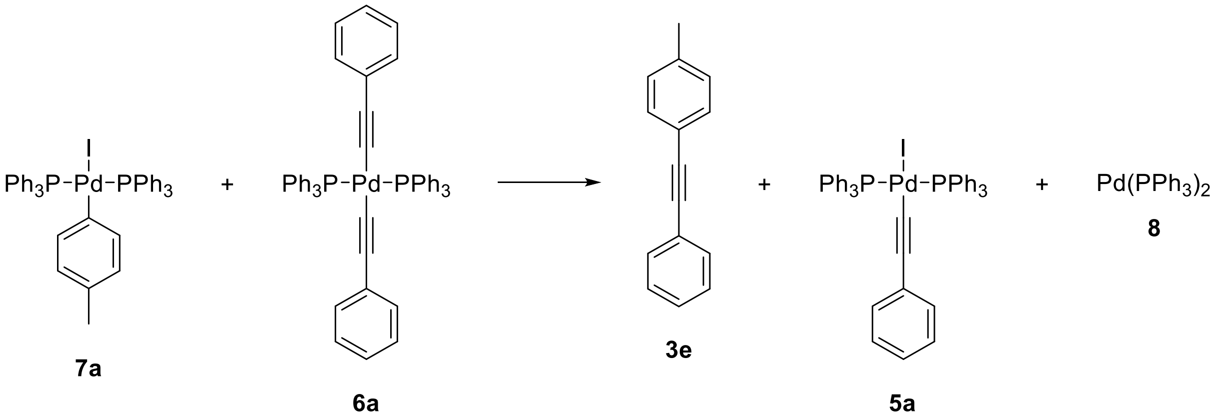

Following *GP13* using bis(triphenylphosphine)palladium(II) (4-methylphenyl)ide iodide (**7a**) (6.83 mg, 8.04 µmol), bis(triphenylphosphine)palladium(II) bis(phenylethynide) (**6a**) (6.68 mg, 8.02 µmol), $CDCl_3$ (0.800 mL).

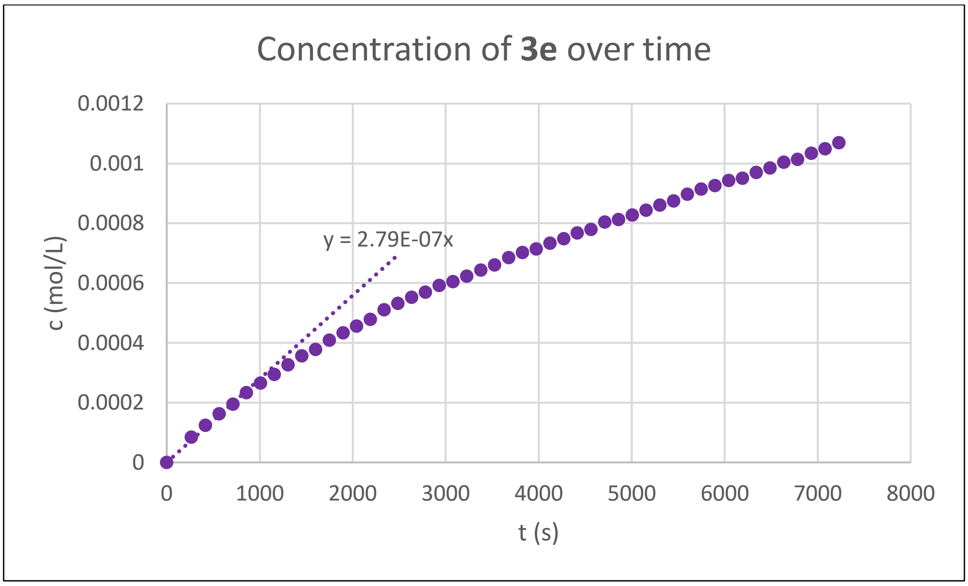

Time [s]	c [mol/L]
0	0
265	8.428E-05
415	1.238E-04
564	1.623E-04
711	1.940E-04
857	2.330E-04

$v_0 = (2.79 \pm 0.05) \times 10^{-7}$ mol/Ls

Transmetallation of 6a and 7d

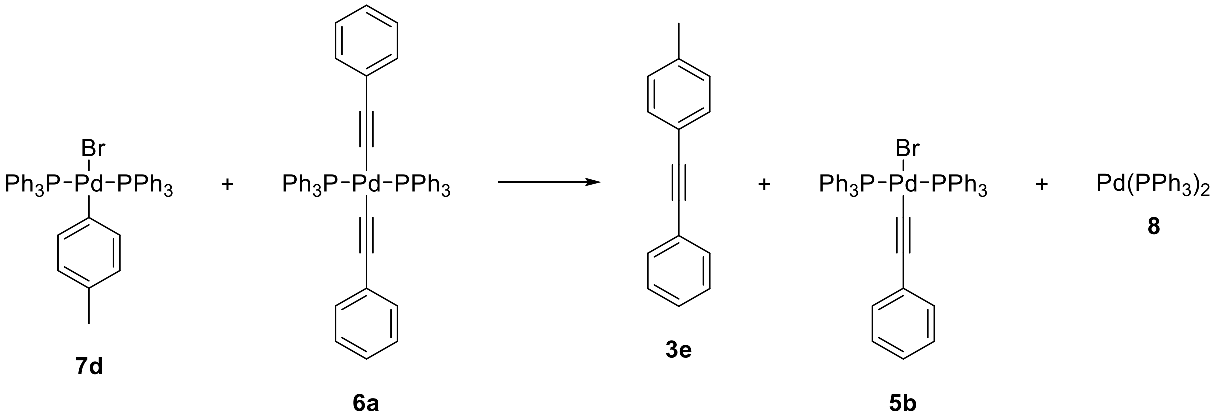

Following *GP13* using bis(triphenylphosphine)palladium(II) (4-methylphenyl)ide bromide (**7d**) (6.42 mg, 8.00 µmol), bis(triphenylphosphine)palladium(II) bis(phenylethynide) (**6a**) (6.70 mg, 8.04 µmol), $CDCl_3$ (0.800 mL).

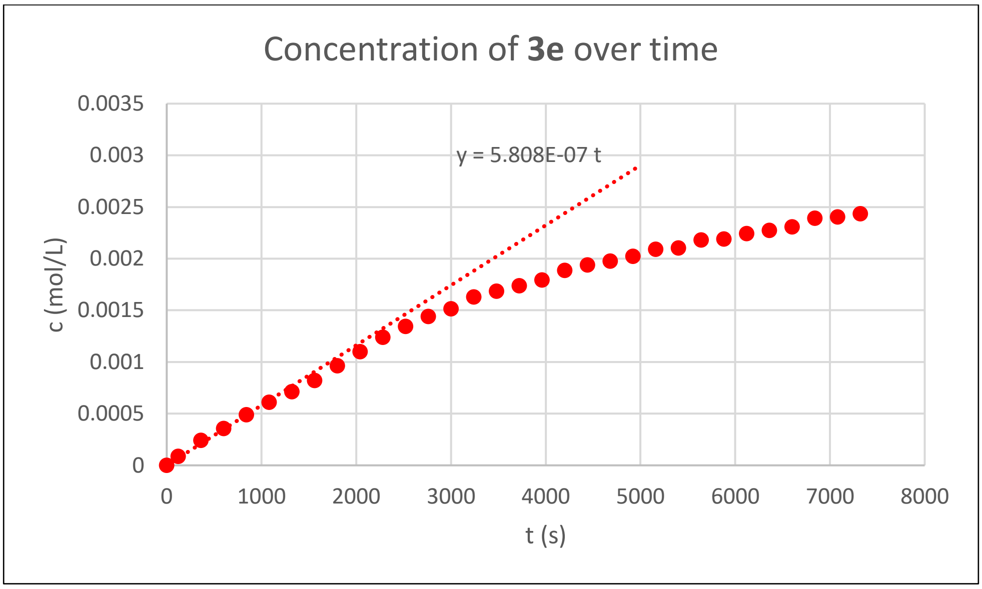

Time [s]	c [mol/L]
0	0
120	8.615E-05
360	2.408E-04
600	3.568E-04
840	4.877E-04
1080	6.105E-04

$v_0 = (5.8 \pm 0.1) \times 10^{-7}$ mol/Ls

Transmetallation of 6a and 7g

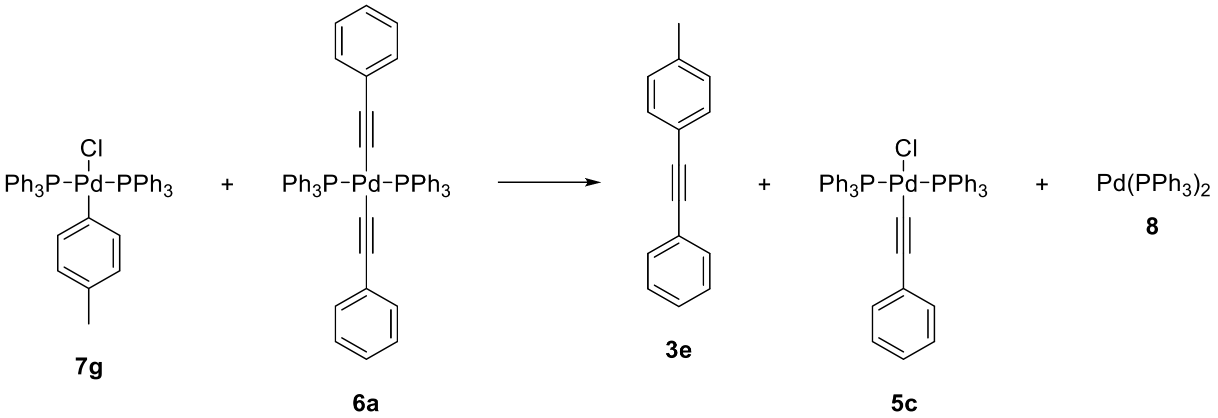

Following *GP13* using bis(triphenylphosphine)palladium(II) (4-methylphenyl)ide chloride (**7g**) (6.42 mg, 8.00 µmol), bis(triphenylphosphine)palladium(II) bis(phenylethynide) (**6a**) (6.70 mg, 8.04 µmol), $CDCl_3$ (0.800 mL).

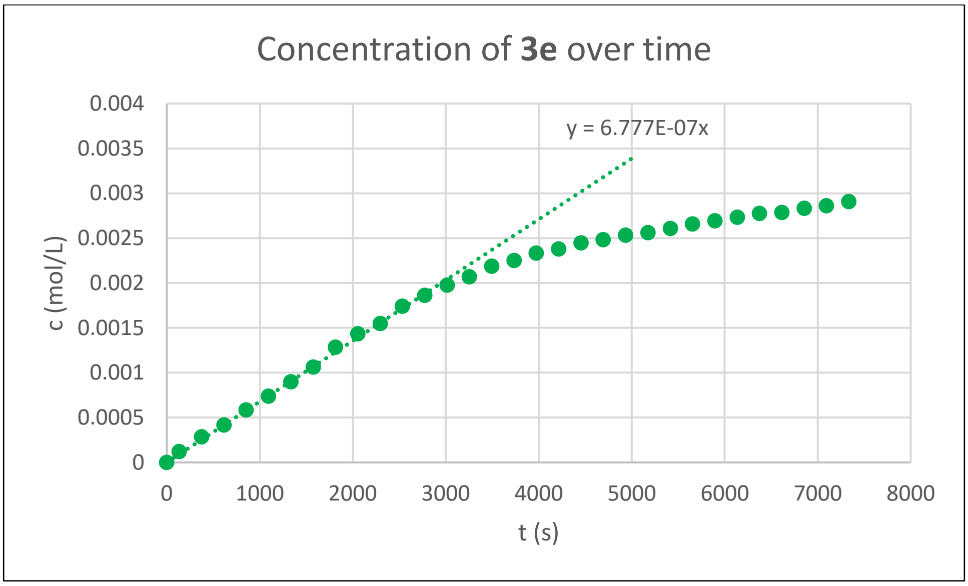

Time [s]	c [mol/L]
0	0
135	1.188E-04
375	2.858E-04
615	4.146E-04
855	5.842E-04
1095	7.381E-04
1335	8.991E-04
1575	1.063E-03

$v_0 = (6.78 \pm 0.06) \times 10^{-7}$ mol/Ls

Transmetallation of 6a and 7j

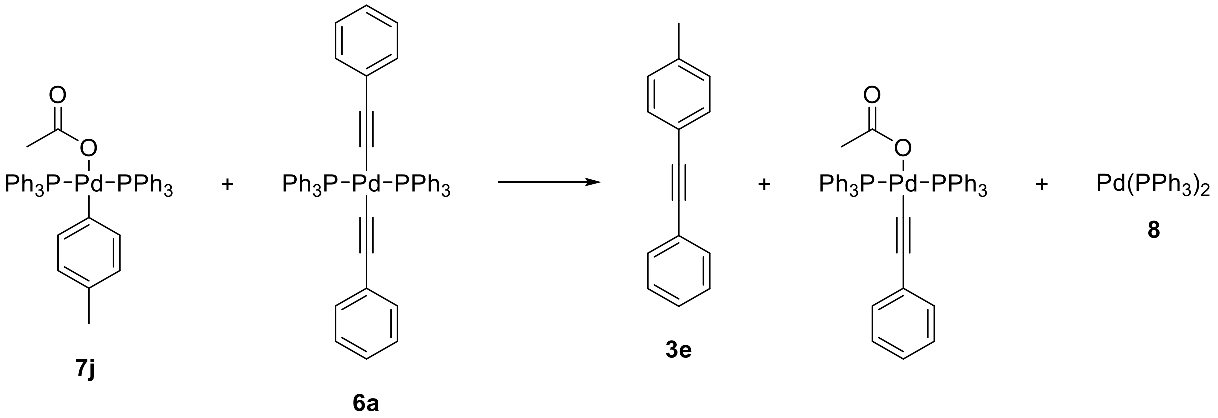

Following *GP13* using bis(triphenylphosphine)palladium(II) (4-methylphenyl)ide acetate (**7j**) (6.25 mg, 8.00 µmol), bis(triphenylphosphine)palladium(II) bis(phenylethynide) (**6a**) (6.69 mg, 8.03 µmol), $CDCl_3$ (0.800 mL).

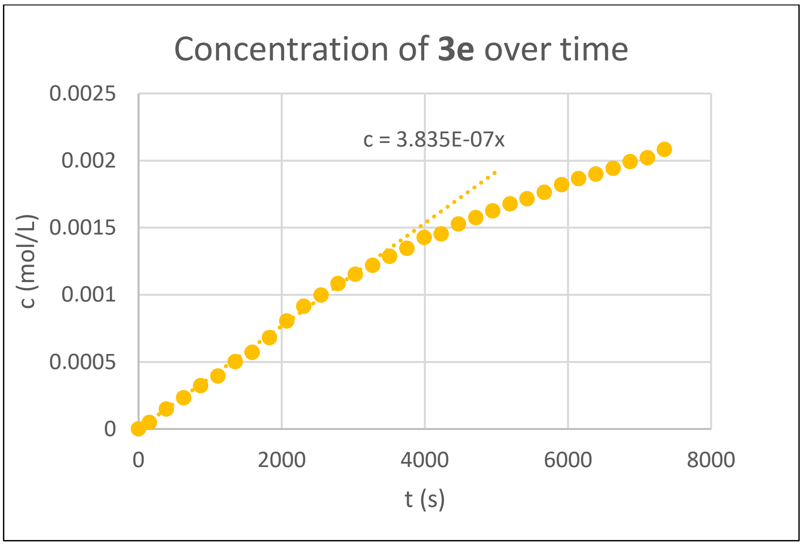

Time [s]	c [mol/L]
0	0
150	4.878E-05
390	1.488E-04
630	2.328E-04
870	3.220E-04
1110	3.934E-04
1350	5.006E-04
1590	5.709E-04
1830	6.809E-04
2070	8.048E-04
2310	9.142E-04
2550	9.986E-04
2790	1.083E-03

$v_0 = (3.84 \pm 0.04) \times 10^{-7}$ mol/Ls

General procedure 14 (*GP14*) – Transmetallation reactions between palladium oxidative addition complex 7a and palladium monoacetylides 5

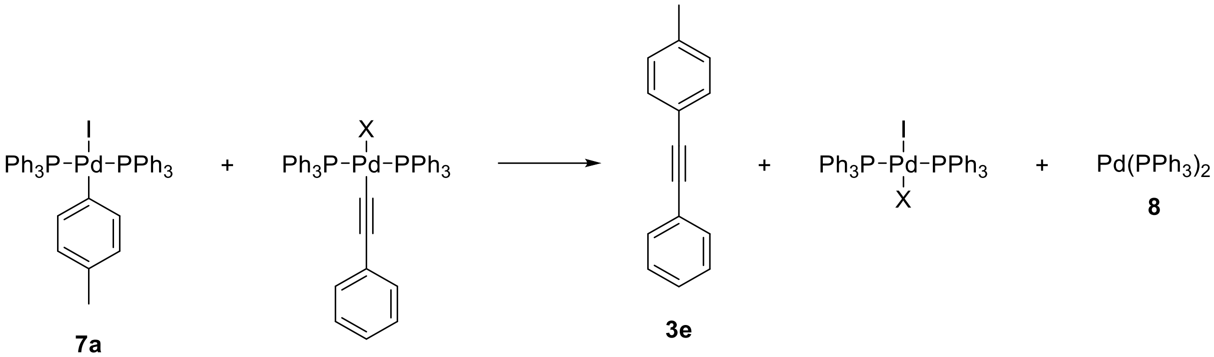

7a **5** **3e**

In a vial a solution of bis(triphenylphosphine)palladium (4-methylphenyl)ide iodide (**7a**) (1 equiv., 0.01 M) in $CDCl_3$ was prepared under argon atmosphere. Bis(triphenylphosphine)palladium phenylethynide halide (**5**) (1.0 equiv.) was added to this solution in one portion. The reaction mixture was sonicated for 0.5 min and transferred to NMR tube, flushed with argon and sealed. NMR spectra were acquired in 4.0 min intervals.

Transmetallation of 5a and 7a

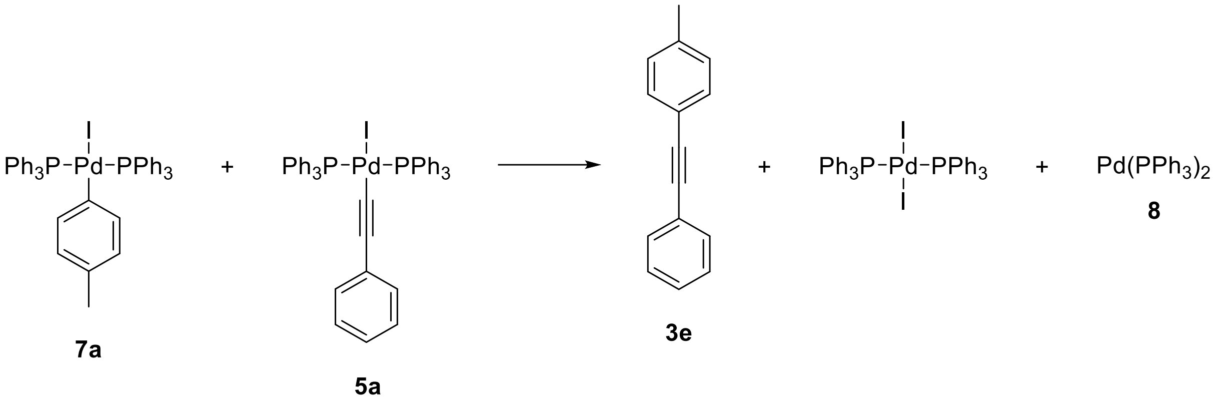

Following *GP14* using bis(triphenylphosphine)palladium(II) (4-methylphenyl)ide iodide (**7a**) (6.85 mg, 8.06 µmol), bis(triphenylphosphine)palladium(II) phenylethynide iodide (**5a**) (6.96 mg, 8.10 µmol), $CDCl_3$ (0.800 mL).

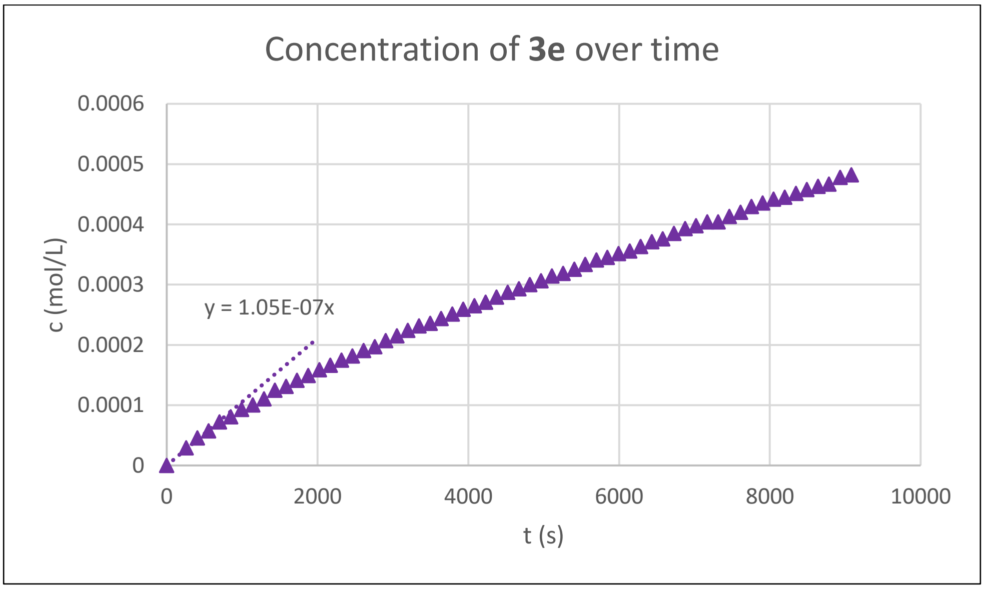

Time [s]	c [mol/L]
0	0
260	2.929E-05
470	4.062E-05
554	5.749E-05
701	7.222E-05

$v_0 = (1.05 \pm 0.02) \times 10^{-7}$ mol/Ls

Transmetallation of 5b and 7a

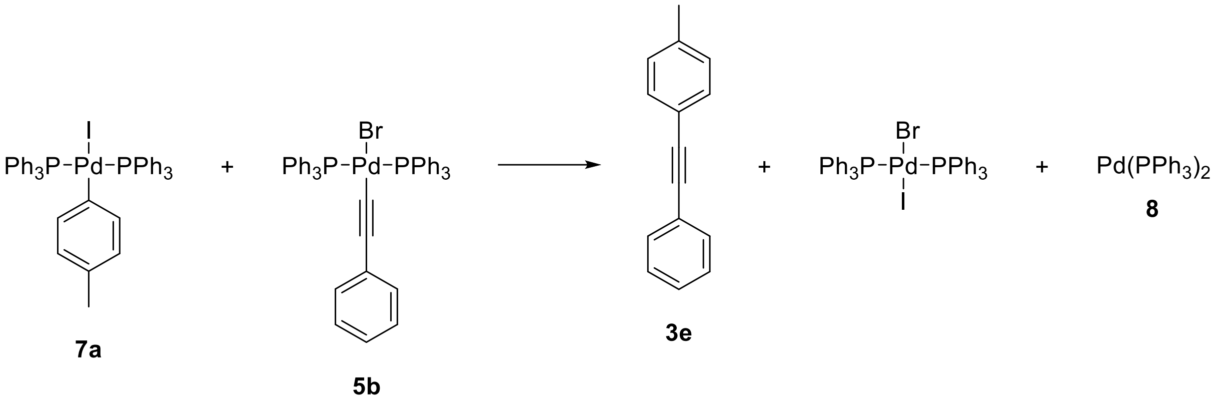

Following *GP14* using bis(triphenylphosphine)palladium(II) (4-methylphenyl)ide iodide (**7a**) (6.80 mg, 8.01 µmol), bis(triphenylphosphine)palladium(II) phenylethyl-1-ide bromide (**5b**) (6.87 mg, 8.00 µmol), $CDCl_3$ (0.800 mL).

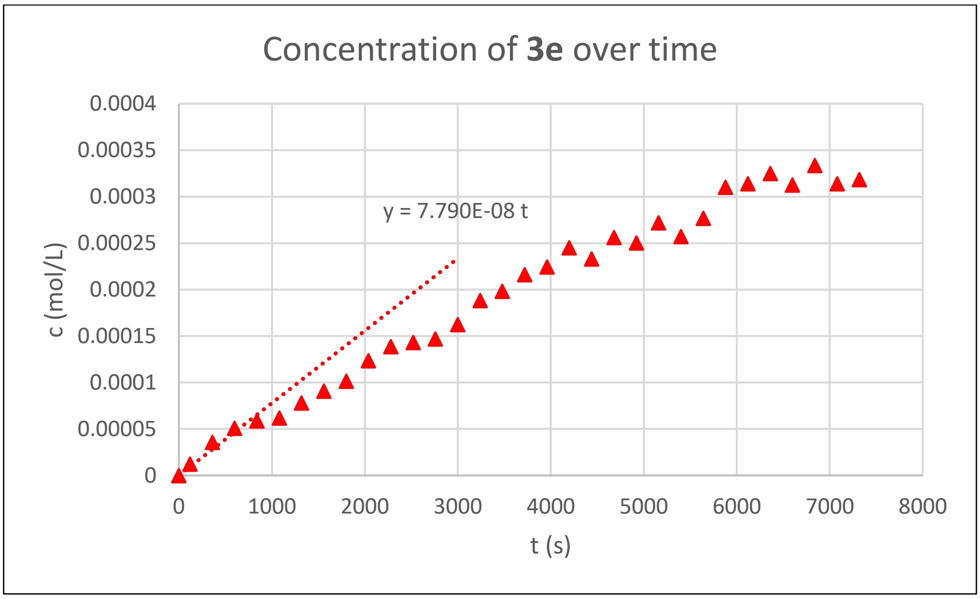

Time [s]	c [mol/L]
0	0
120	1.240E-05
360	3.583E-05
600	5.087E-05
840	5.871E-05

$v_0 = (7.8 \pm 0.2) \times 10^{-8}$ mol/Ls

Transmetallation of 5c and 7a

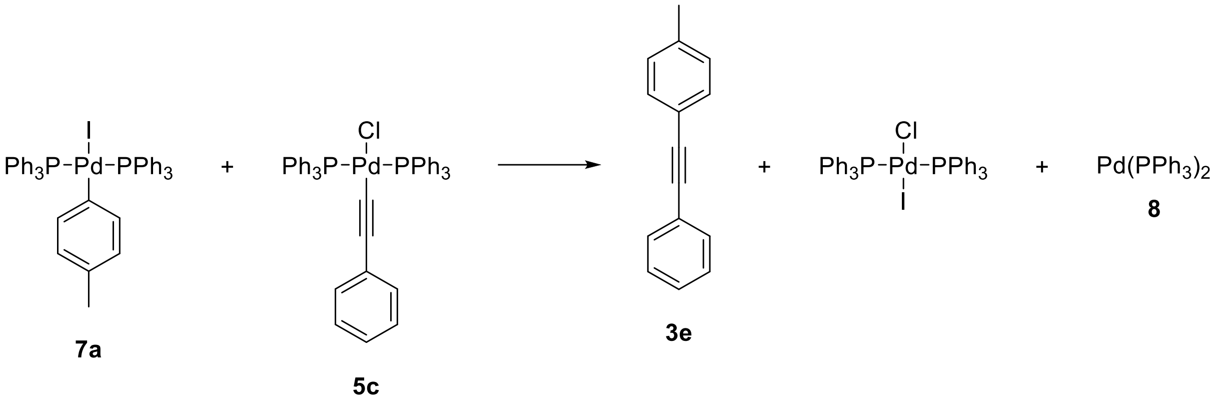

Following *GP14* using bis(triphenylphosphine)palladium(II) (4-methylphenyl)ide iodide (**7a**) (6.81 mg, 8.02 µmol), bis(triphenylphosphine)palladium(II) phenylethynide chloride (**5c**) (6.87 mg, 8.05 µmol), $CDCl_3$ (0.800 mL).

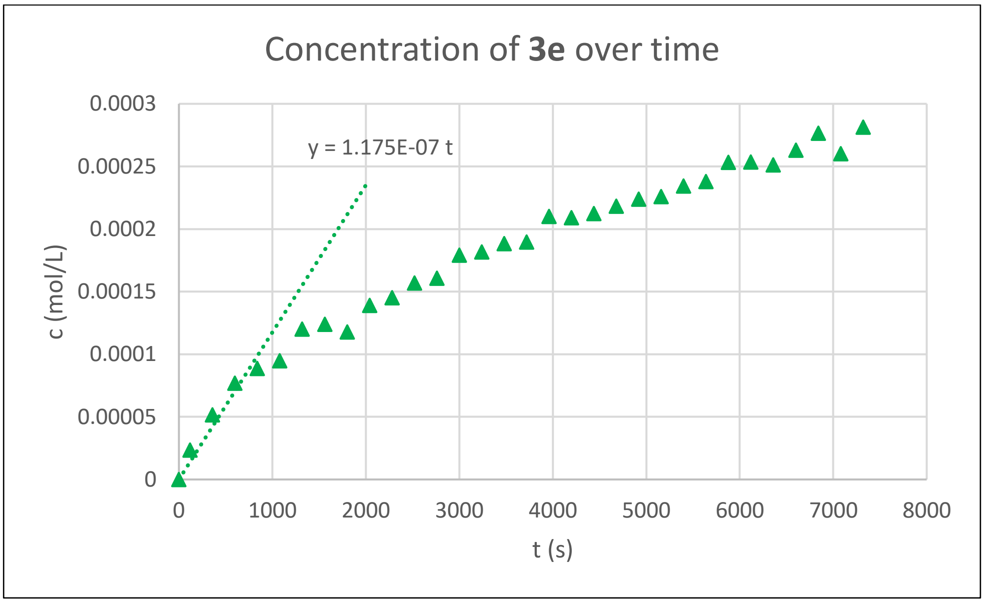

Time [s]	c [mol/L]
0	0
120	2.346E-05
360	5.164E-05
600	7.695E-05
840	8.877E-05

$v_0 = (1.18 \pm 0.08) \times 10^{-7}$ mol/Ls

Comparison of rates of transmetallation reactions of palladium oxidative addition complexes 7 with palladium (bis)acetylides 5 and 6 (Figure 7).

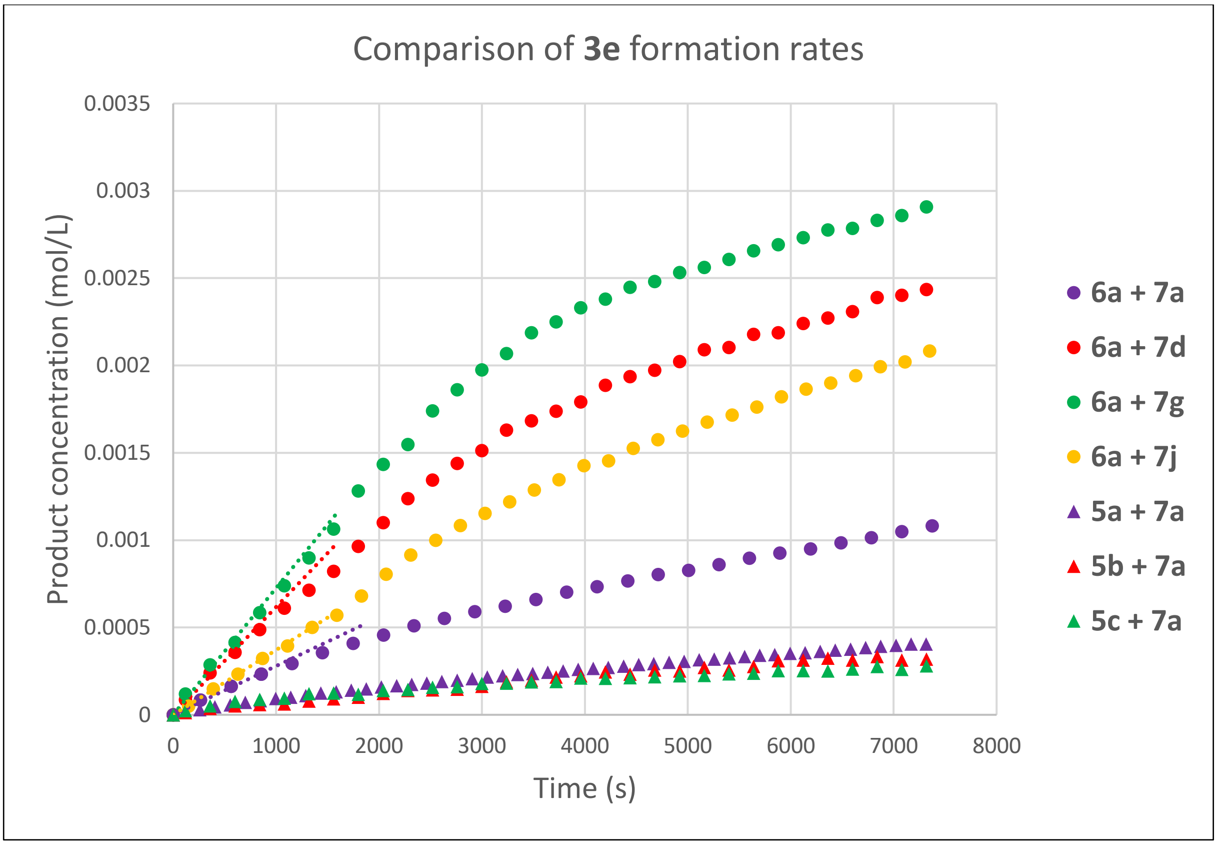

Influence of halide ligand (X) in **7** on the rate of transmetallation reaction with **6a**.

X	v (µmol/Ls)
Cl	0.678
Br	0.58
OAc	0.384
I	0.279

Hammett studies of palladium-palladium transmetallation

The role of *para*-substituent in bis(triphenylphosphine)palladium(II) aryl iodide 7 on the rate of transmetallation with palladium bisacetylide 6a

Transmetallation reactions were carried out in a sealed NMR tube at 302.0 K. The starting concentration of bis(triphenylphosphine)palladium(II) aryl iodide **7** was 0.01 M. Bis(triphenylphosphine)palladium bis(phenylethynide) **6a** was added in excess amount (1.15 equiv.). ^{1}H NMR spectra were recorded in 6.25 min intervals. The concentration of the product **3** was determined by comparison of integrals of characteristic resonances of product **3** with integrals of resonances of an internal standard (1,3,5-trimethoxybenzene).

General procedure 15 (*GP15*) – Transmetallation reaction between 7 and 6a.

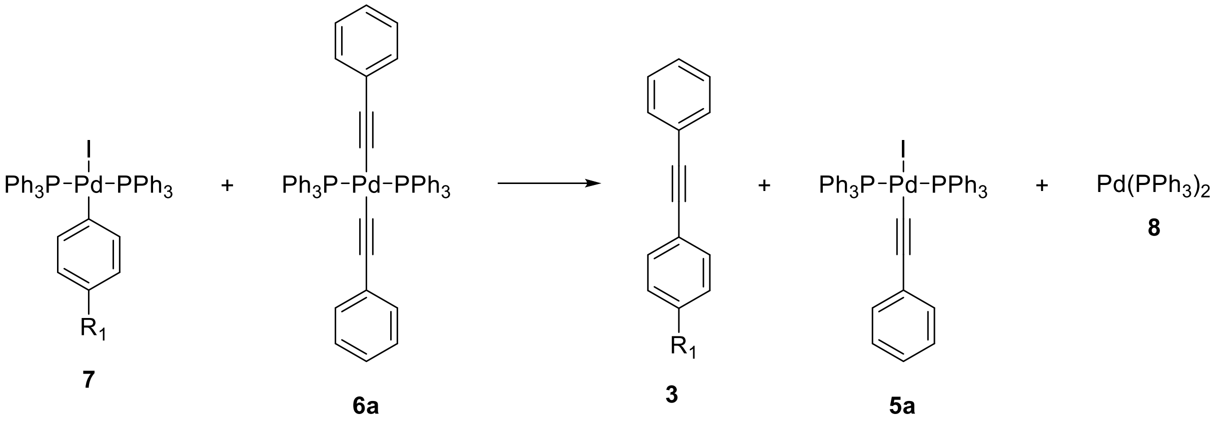

A solution of bis(triphenylphosphine)palladium aryl iodide **7** (1 equiv., 0.01 M) in $CDCl_3$ was prepared in a vial under argon atmosphere. Bis(triphenylphosphine)palladium bis(phenylethynide) (**6a**) (1.15 equiv.) was added to this solution in one portion. The reaction mixture was sonicated for 0.5 min and transferred to an NMR tube, purged with argon and sealed. NMR spectra were recorded at 6.25-minute intervals.

Transmetallation of 6a and 7b

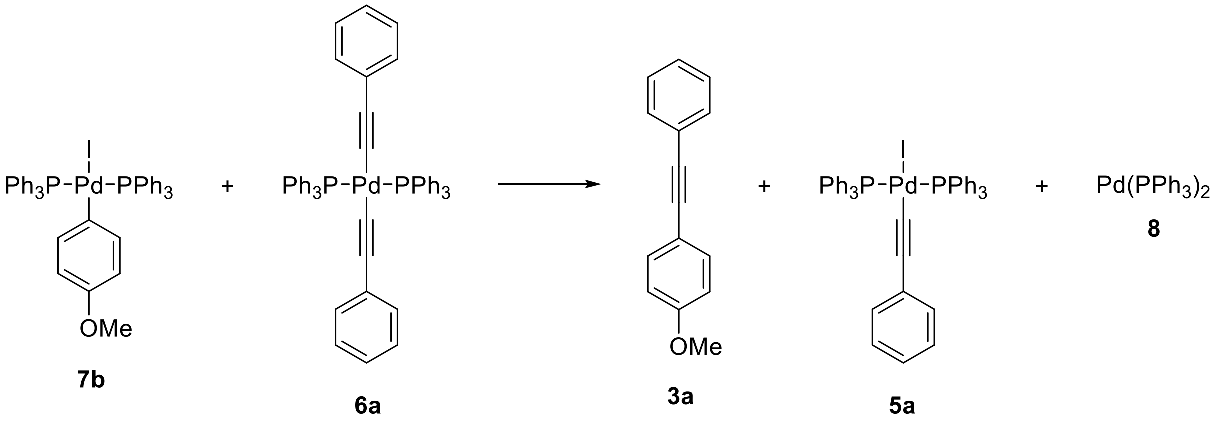

Following *GP15* using bis(triphenylphosphine)palladium(II) (4-methoxyphenyl)ide iodide (**7b**) (6.92 mg, 8.00 µmol), bis(triphenylphosphine)palladium(II) bis(phenylethynide) (**6a**) (7.72 mg, 9.26 µmol), $CDCl_3$ (0.800 mL).

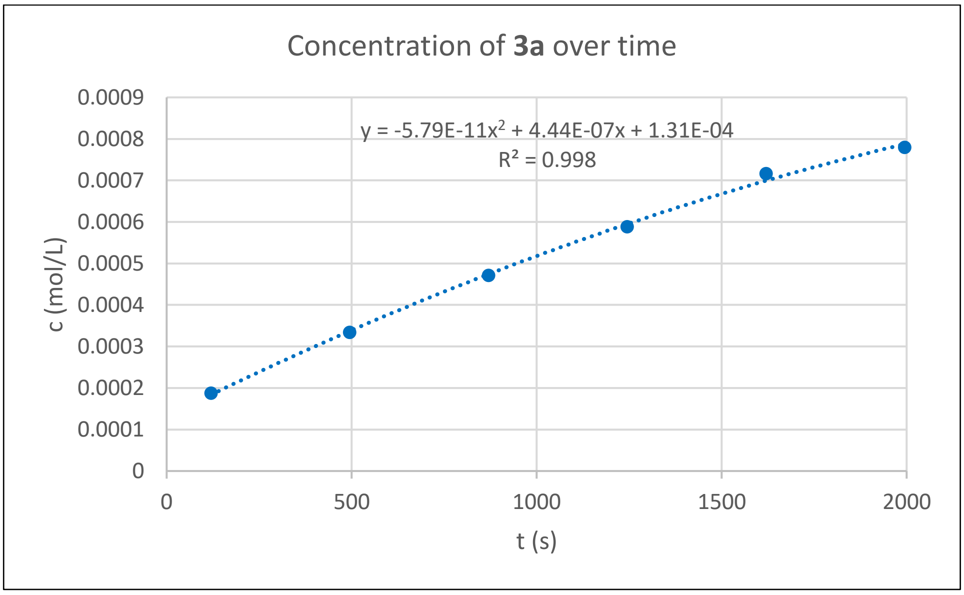

Time [s]	c [mol/L]
120	1.873E-04
495	3.337E-04
870	4.710E-04
1245	5.888E-04
1620	7.166E-04
1995	7.794E-04

$v_0 = (4.4 \pm 0.3) \times 10^{-7}$ mol/Ls

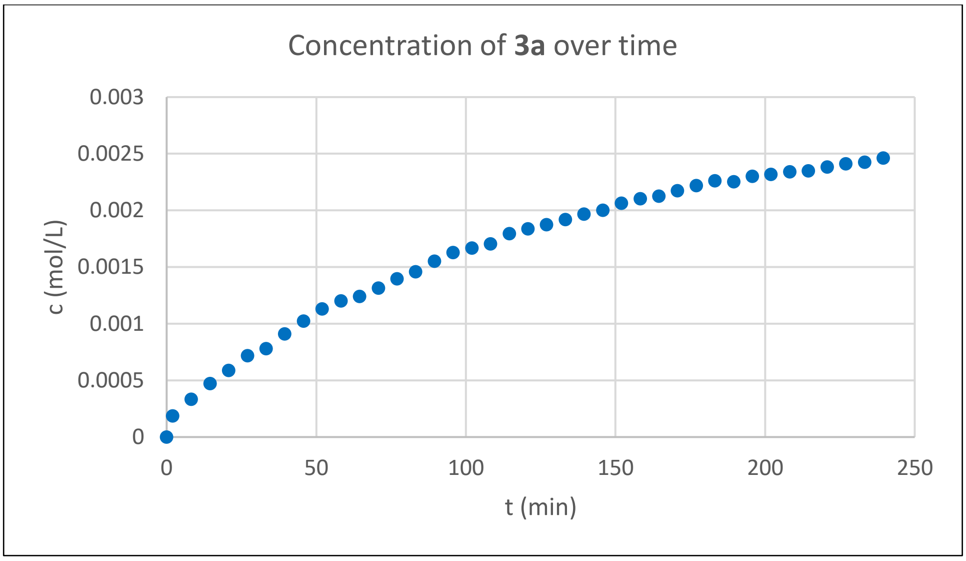

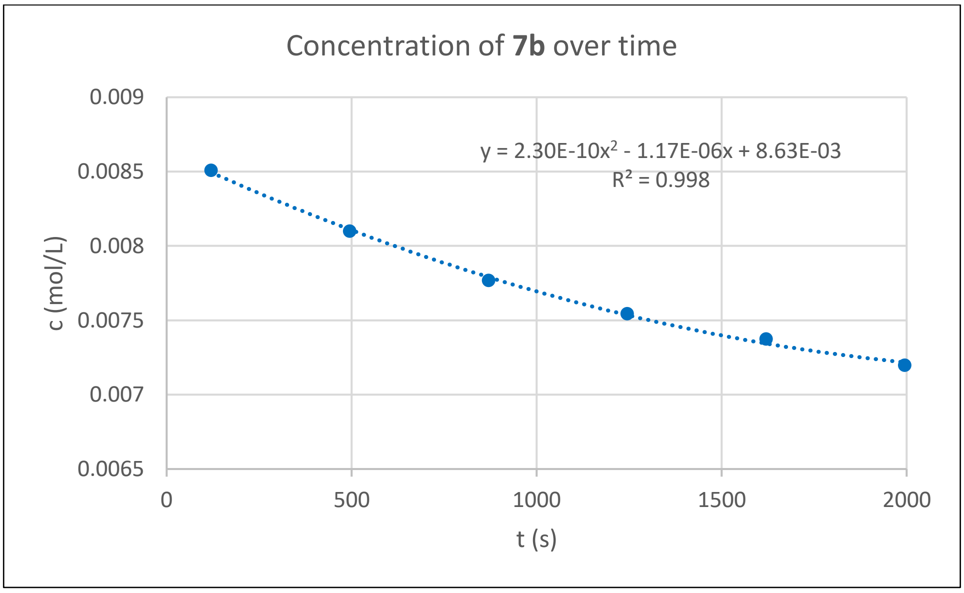

Time [s]	c [mol/L]
120	8.508E-03
495	8.010E-03
870	7.768E-03
1245	7.545E-03
1620	7.374E-03
1995	7.199E-03

$v_0 = -(1.17 \pm 0.07) \times 10^{-6}$ mol/Ls

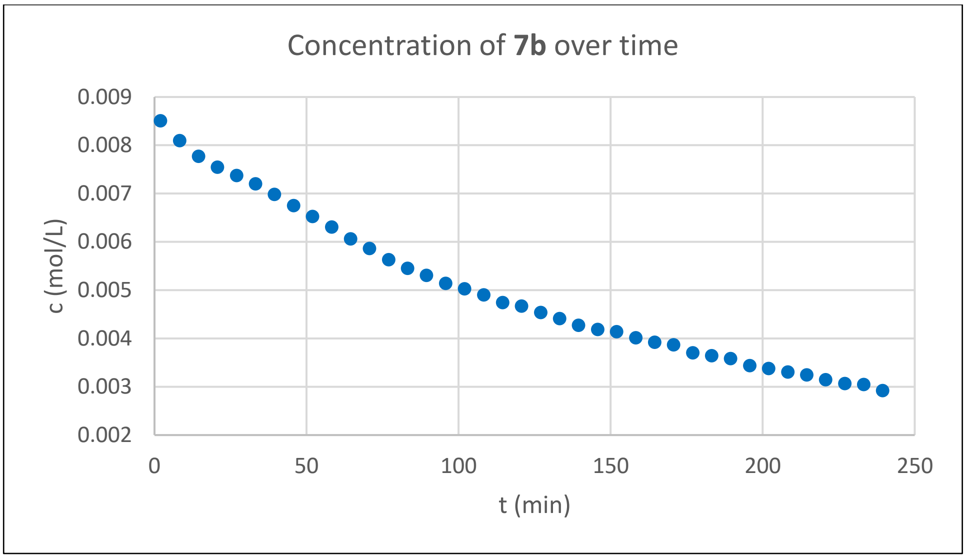
Concentration of **7b** over time
c (mol/L)
0.009
0.008
0.007
0.006
0.005
0.004
0.003
0.002
0
50
100
150
200
250
t (min)

Transmetallation of 6a and 7a

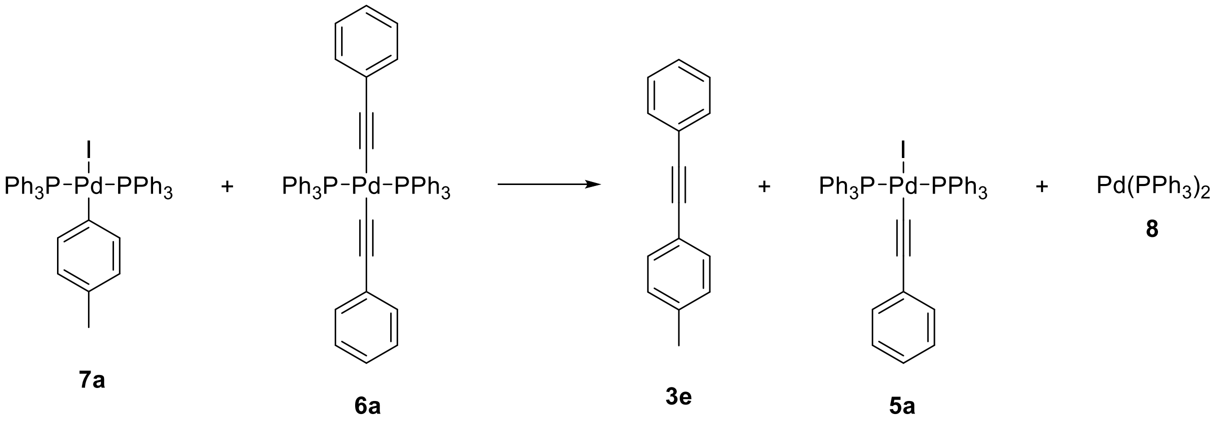

Following *GP15* using bis(triphenylphosphine)palladium(II) (4-methylphenyl)ide iodide (**7a**) (6.78 mg, 7.99 µmol), bis(triphenylphosphine)palladium(II) bis(phenylethynide) (**6a**) (7.74 mg, 9.29 µmol), $CDCl_3$ (0.800 mL).

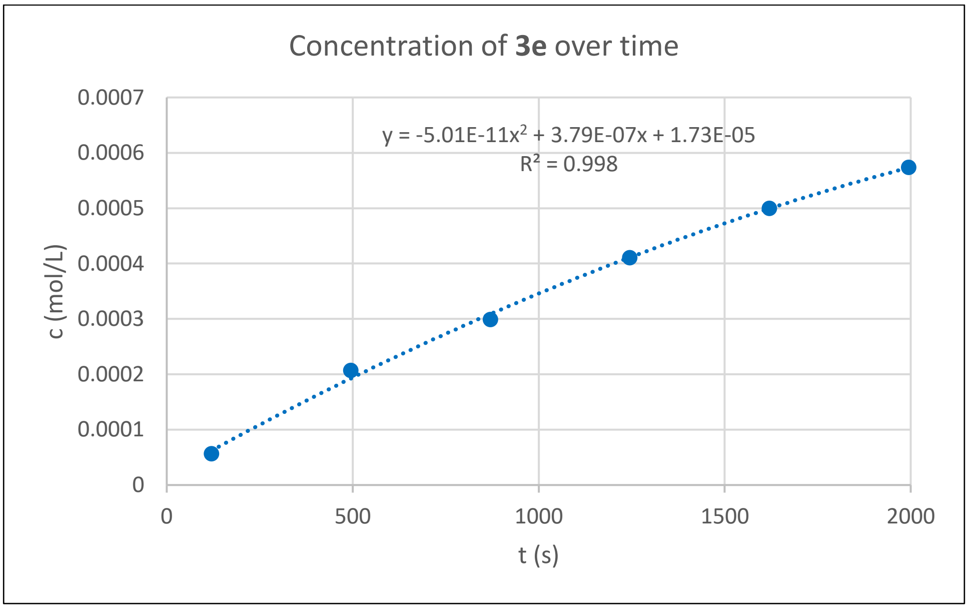

Time [s]	c [mol/L]
120	5.640E-05
495	2.068E-04
870	2.992E-04
1245	4.108E-04
1620	4.998E-04
1995	5.741E-04

$v_0 = (3.8 \pm 0.3) \times 10^{-7}$ mol/Ls

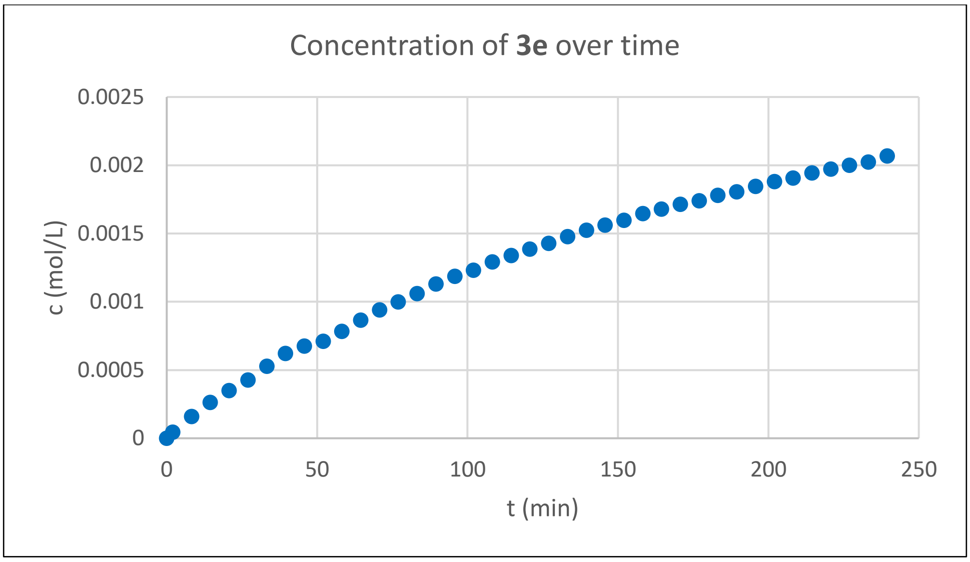

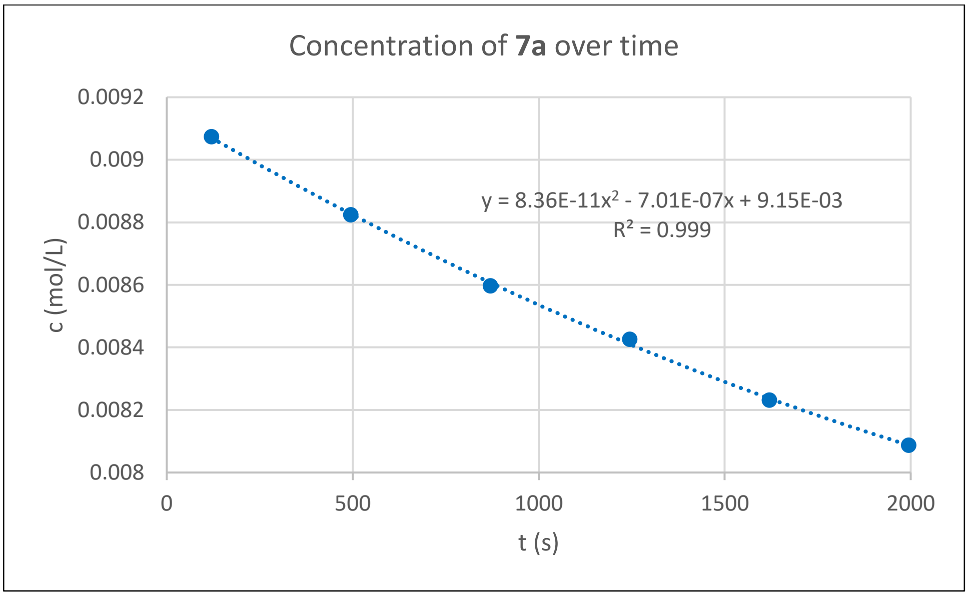

Time [s]	c [mol/L]
120	9.074E-03
495	8.824E-03
870	8.597E-03
1245	8.426E-03
1620	8.232E-03
1995	8.087E-03

$v_0 = -(7.0 \pm 0.3) \times 10^{-7}$ mol/Ls

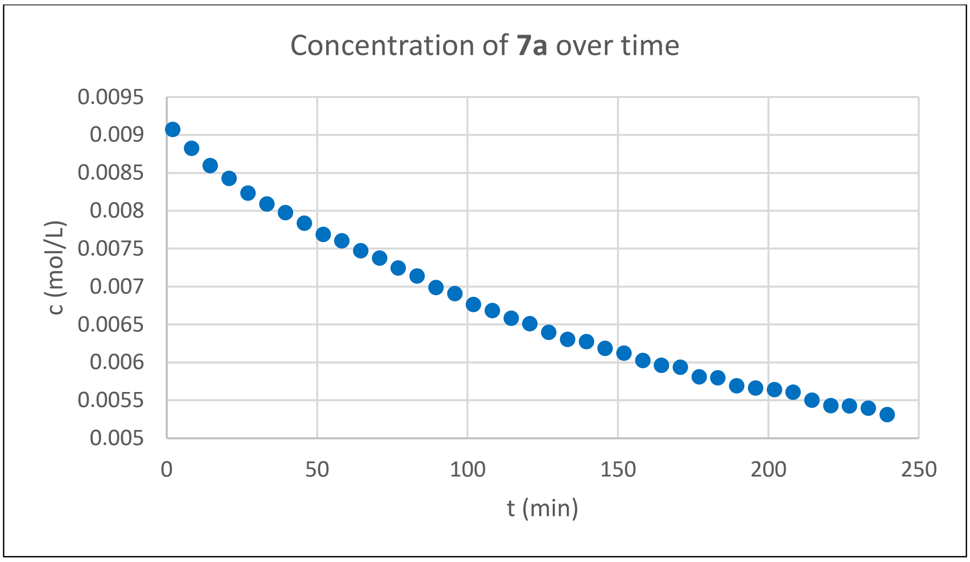
Concentration of **7a** over time
0.0095
0.009
0.0085
0.008
0.0075
0.007
0.0065
0.006
0.0055
0.005
c (mol/L)
0
50
100
150
200
250
t (min)

Transmetallation of 6a and 7l

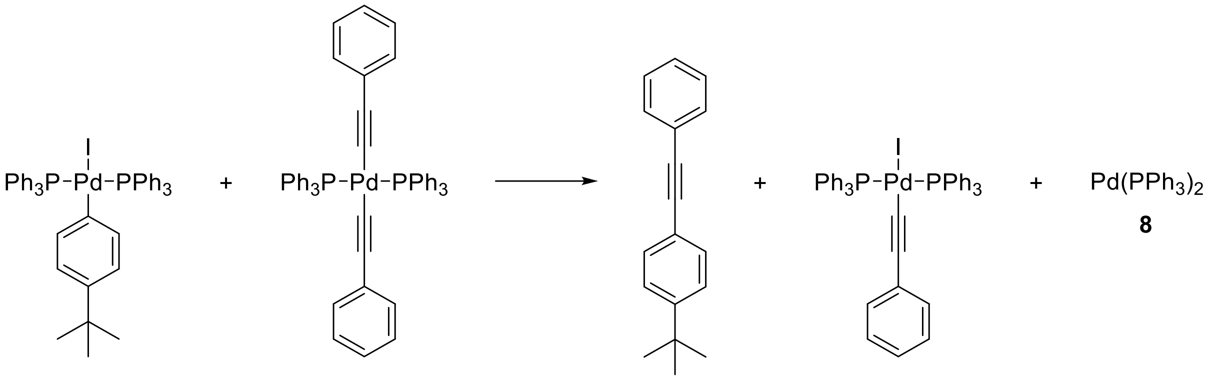

7l 6a 3f 5a

Following *GP15* using bis(triphenylphosphine)palladium(II) (4-*tert*-butylphenyl)ide iodide (**7l**) (7.18 mg, 8.06 µmol), bis(triphenylphosphine)palladium(II) bis(phenylethynide) (**6a**) (7.82 mg, 9.38 µmol), $CDCl_3$ (0.800 mL).

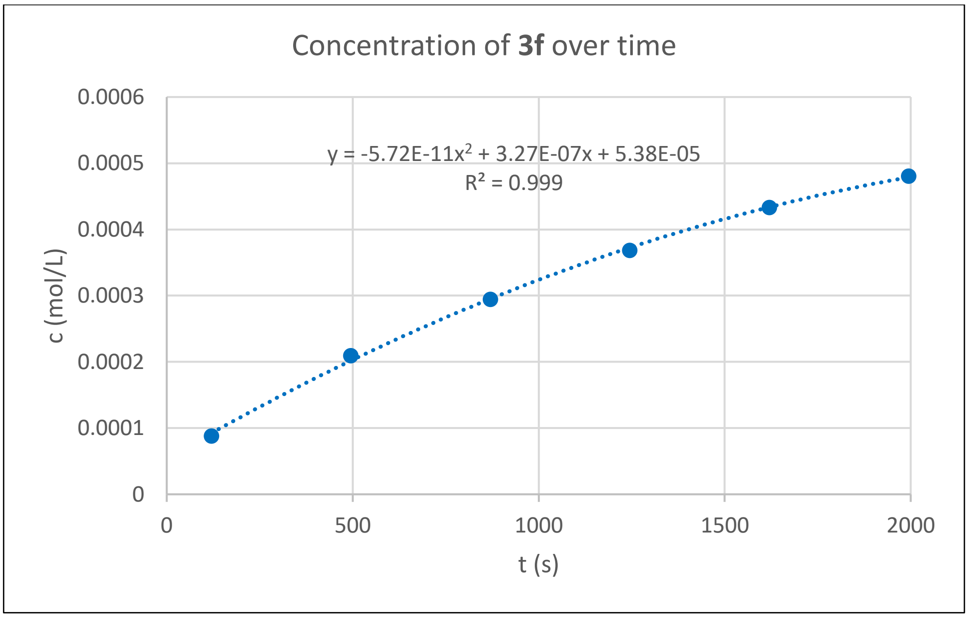

Time [s]	c [mol/L]
120	8.823E-05
495	2.094E-04
870	2.944E-04
1245	3.685E-04
1620	4.331E-04
1995	4.805E-04

$v_0 = (3.3 \pm 0.1) \times 10^{-7}$ mol/Ls

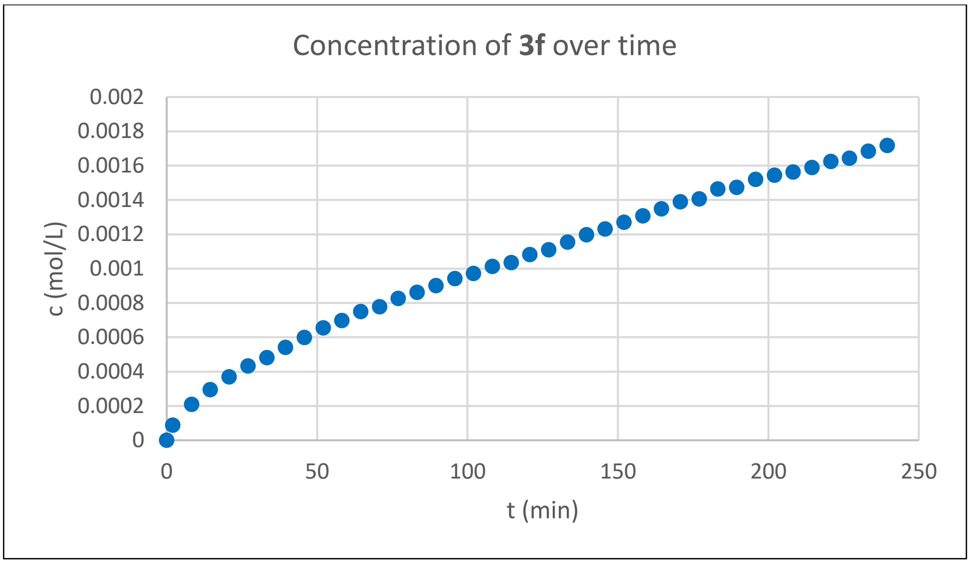

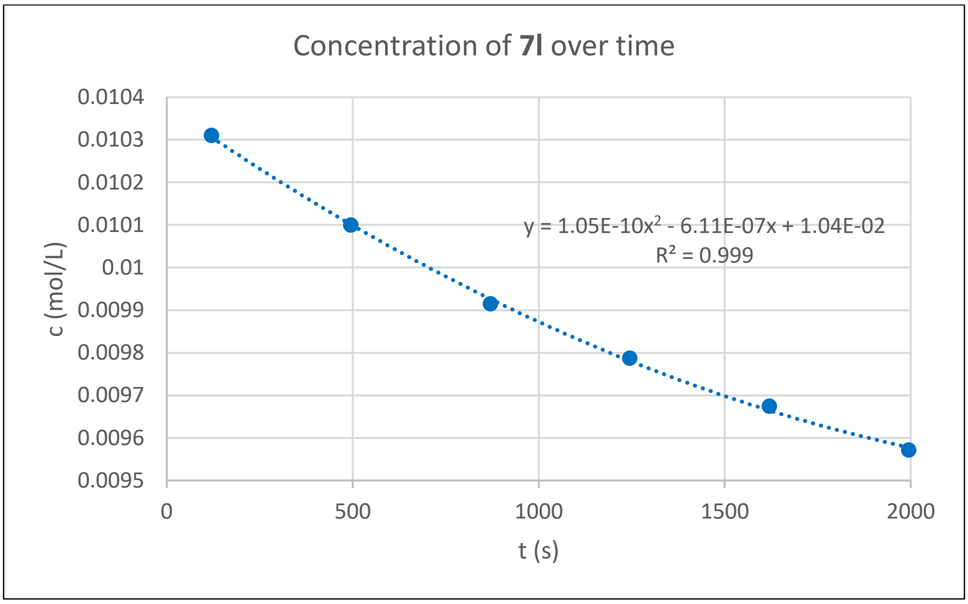

Time [s]	c [mol/L]
120	1.031E-02
495	1.010E-02
870	9.915E-03
1245	9.787E-03
1620	9.674E-03
1995	9.571E-03

$v_0 = -(6.1 \pm 0.3) \times 10^{-7}$ mol/Ls

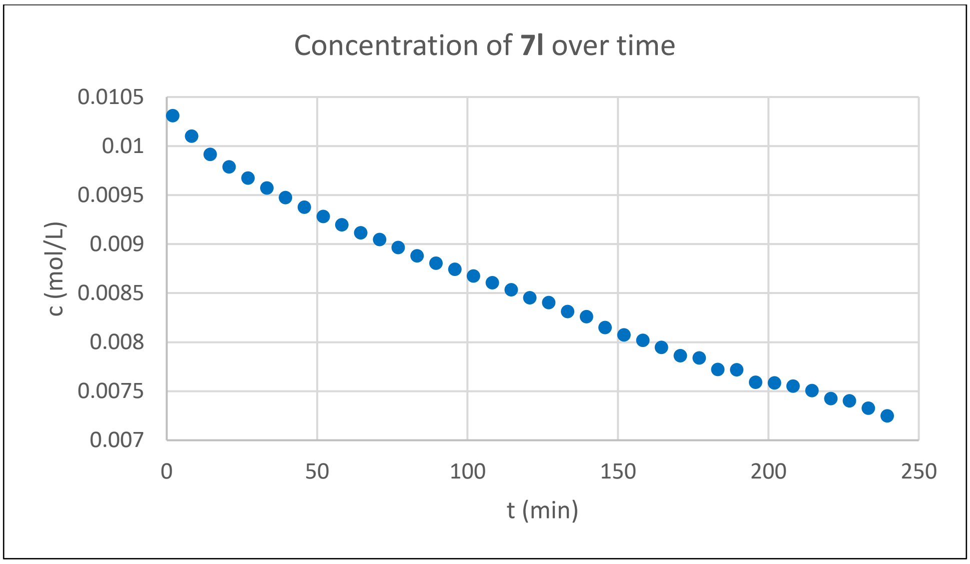
Concentration of **7l** over time
0.0105
0.01
0.0095
0.009
0.0085
0.008
0.0075
0.007
c (mol/L)
0
50
100
150
200
250
t (min)

Transmetallation of 6a and 7m

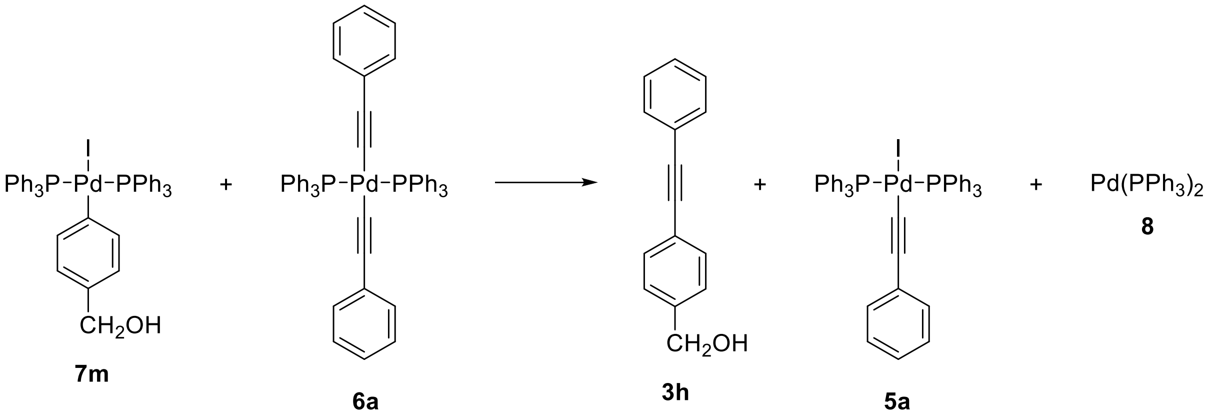

Following *GP15* using bis(triphenylphosphine)palladium(II) (4-hydroxymethyl)phenylide iodide (**7m**) (7.03 mg, 8.09 µmol), bis(triphenylphosphine)palladium(II) bis(phenylethynyl-1-ide) (**6a**) (7.70 mg, 9.24 µmol), $CDCl_3$ (0.800 mL).

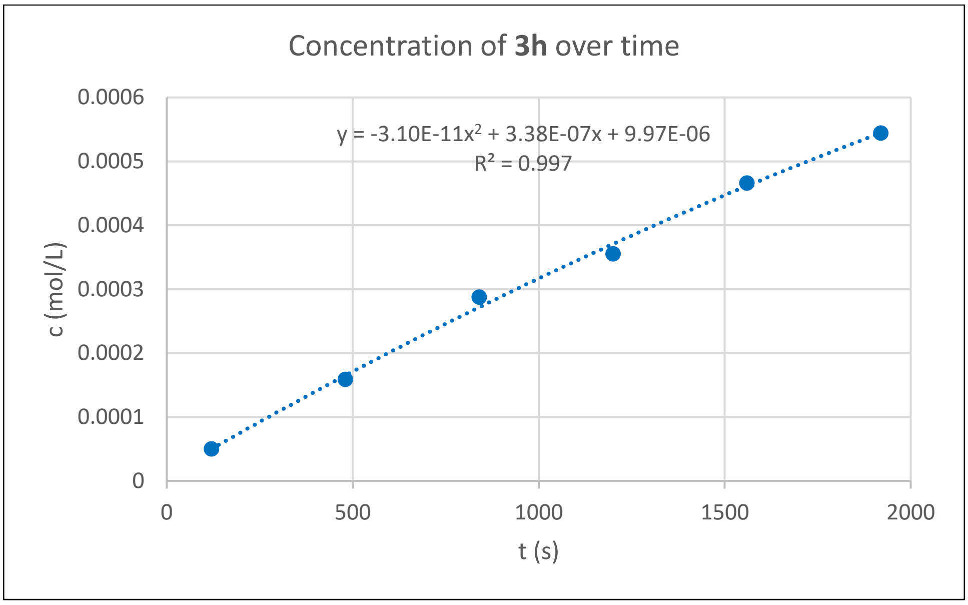

Time [s]	c [mol/L]
120	5.038E-05
480	1.590E-04
840	2.880E-04
1200	3.554E-04
1560	4.665E-04
1920	5.444E-04

$v_0 = (3.4 \pm 0.4) \times 10^{-7}$ mol/Ls

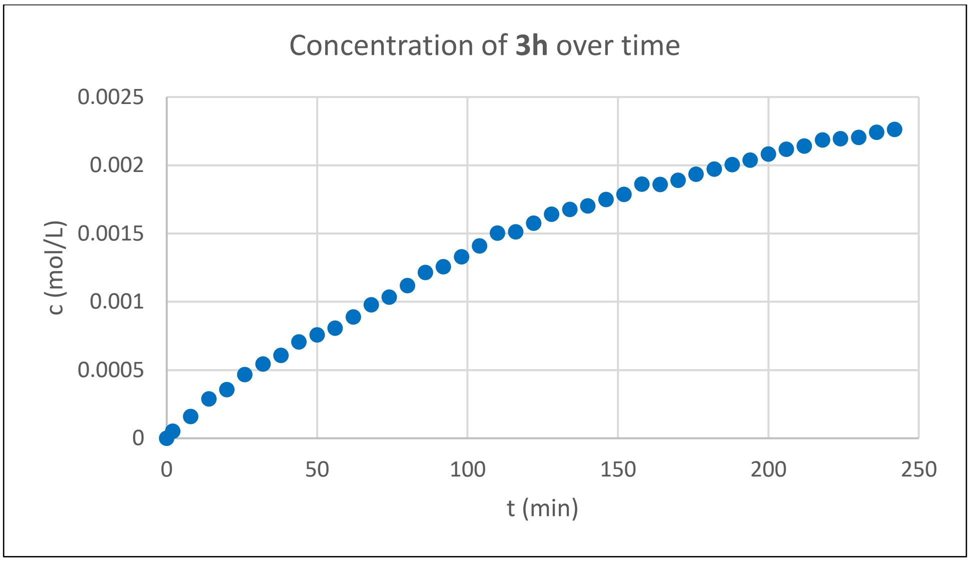

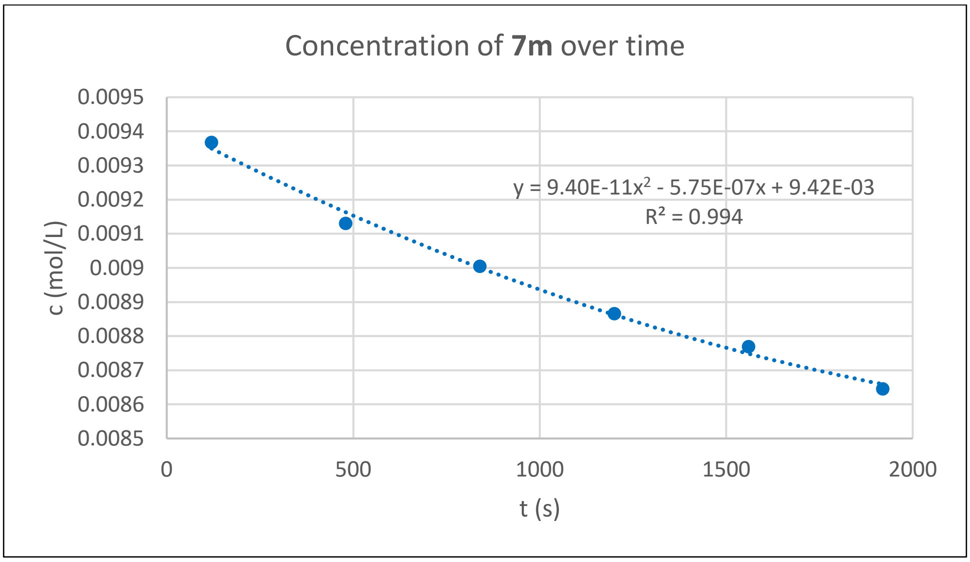

Time [s]	c [mol/L]
120	9.367E-03
480	9.130E-03
840	9.005E-03
1200	8.866E-03
1560	8.769E-03
1920	8.645E-03

$v_0 = -(5.8 \pm 0.7) \times 10^{-7}$ mol/Ls

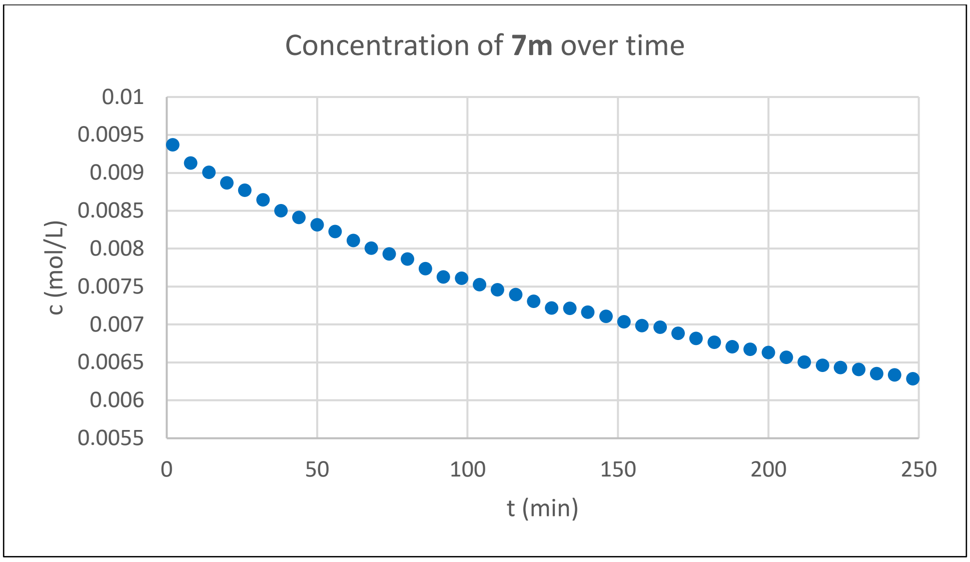
Concentration of **7m** over time
0.01
0.0095
0.009
0.0085
0.008
0.0075
0.007
0.0065
0.006
0.0055
c (mol/L)
0
50
100
150
200
250
t (min)

Transmetallation of 6a and 7q

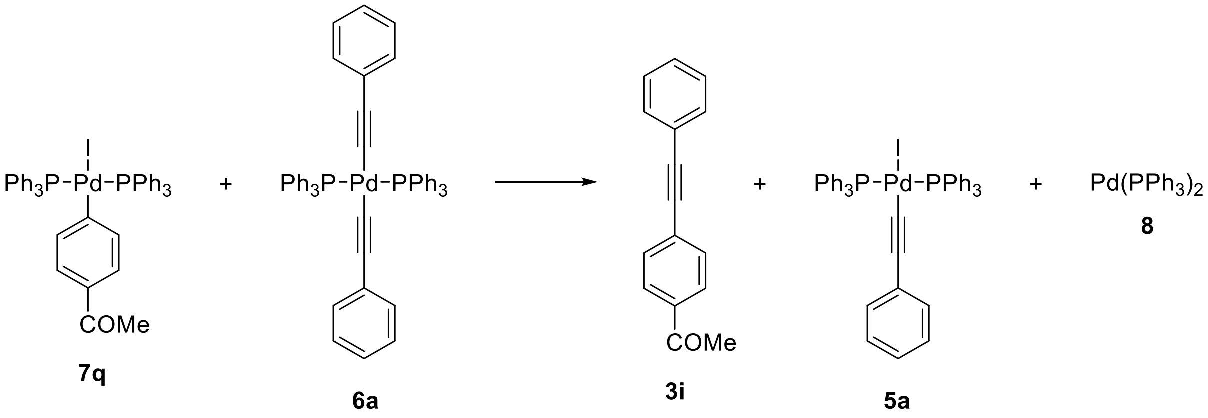

Following *GP15* using bis(triphenylphosphine)palladium(II) (4-acetylphenyl)ide iodide (**7q**) (7.28 mg, 8.30 µmol), bis(triphenylphosphine)palladium(II) bis(phenylethynide) (**6a**) (7.78 mg, 9.34 µmol), $CDCl_3$ (0.800 mL).

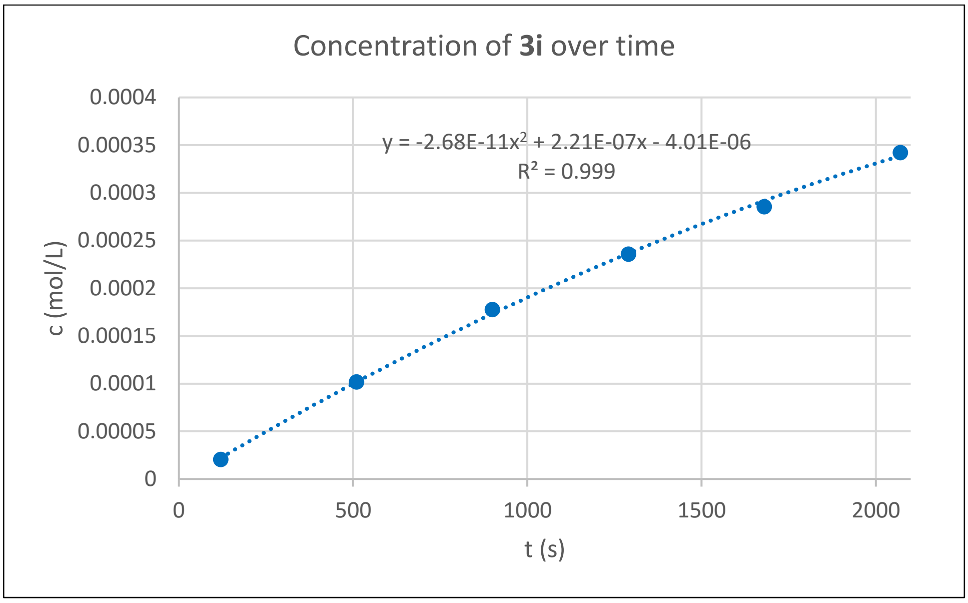

Time [s]	c [mol/L]
120	2.064E-05
510	1.020E-04
900	1.779E-04
1290	2.359E-04
1680	2.854E-04
2070	3.422E-04

$v_0 = (2.2 \pm 0.1) \times 10^{-7}$ mol/Ls

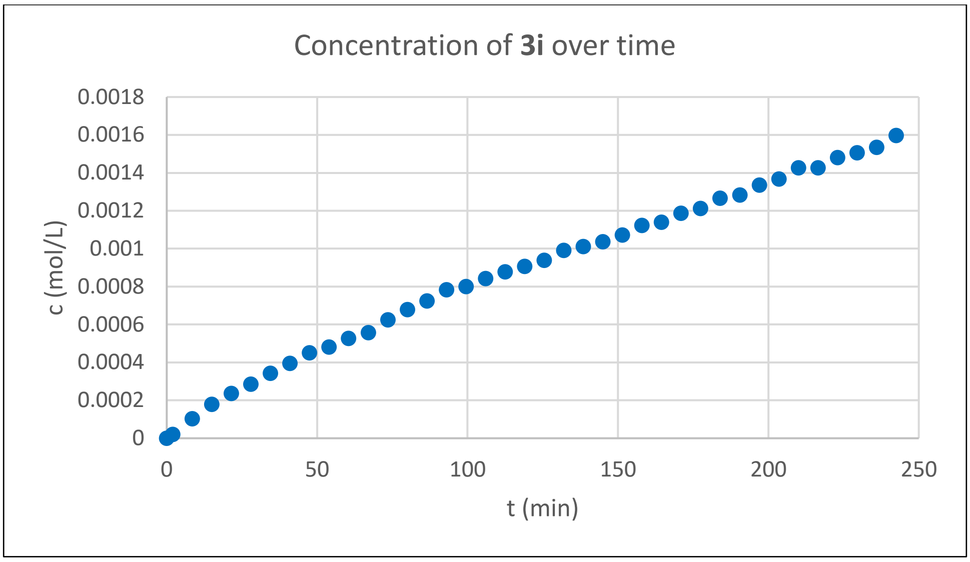

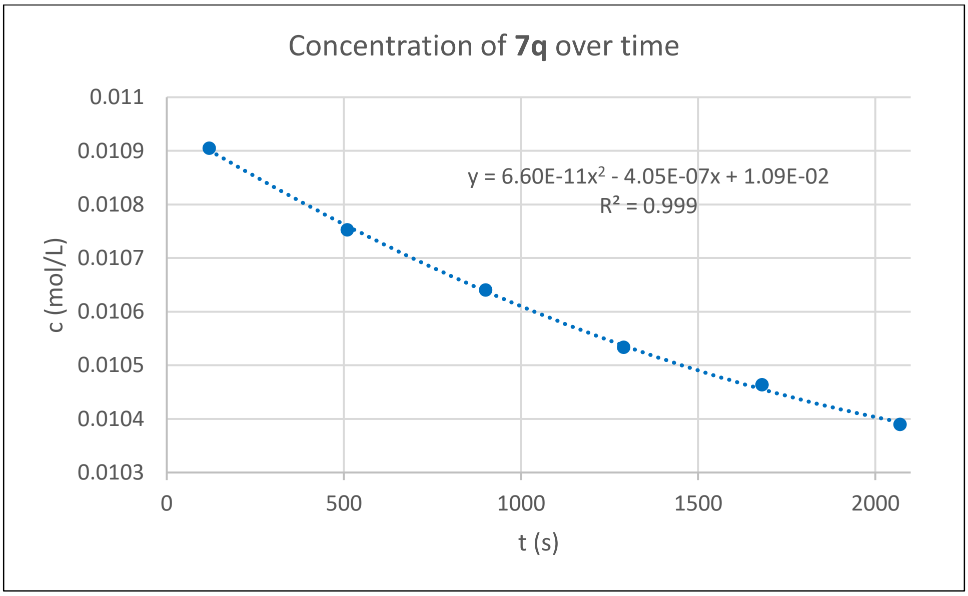

Time [s]	c [mol/L]
120	1.091E-02
510	1.075E-02
900	1.064E-02
1290	1.053E-02
1680	1.046E-02
2070	1.039E-02

$v_0 = -(4.0 \pm 0.2) \times 10^{-7}$ mol/Ls

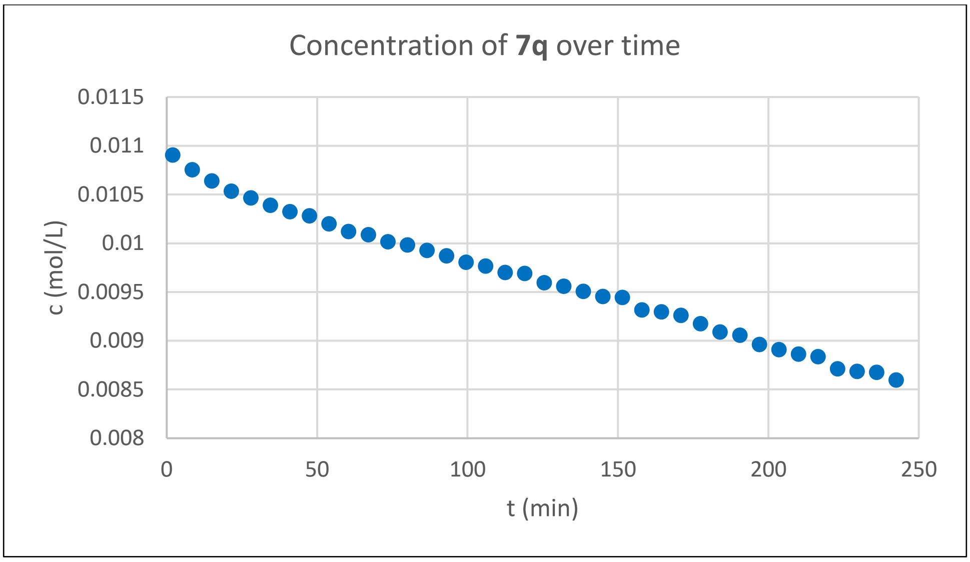
Concentration of **7q** over time
c (mol/L)
0.0115
0.011
0.0105
0.01
0.0095
0.009
0.0085
0.008
0
50
100
150
200
250
t (min)

Transmetallation of 6a and 7o

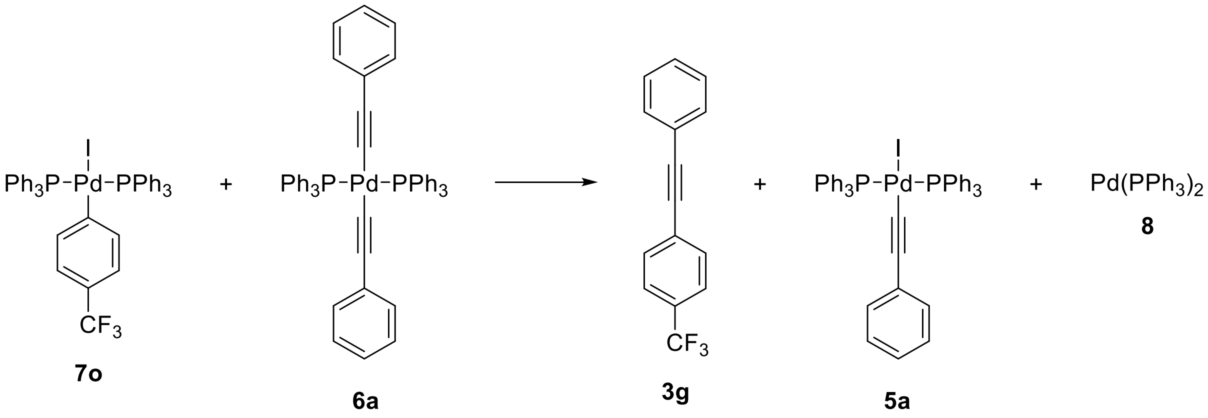

Following *GP15* using bis(triphenylphosphine)palladium(II) (4-trifluoromethylphenyl)ide iodide (**7a**) (7.22 mg, 8.00 µmol), bis(triphenylphosphine)palladium(II) bis(phenylethynyl-1-ide) (**6a**) (7.72 mg, 9.26 µmol), $CDCl_3$ (0.800 mL).

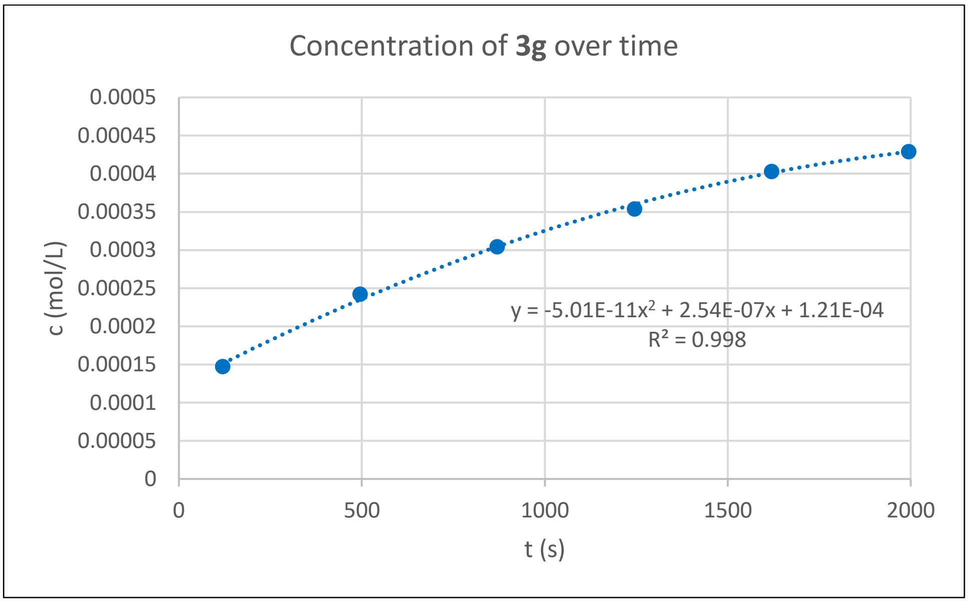

Time [s]	c [mol/L]
120	1.475E-04
495	2.422E-04
870	3.044E-04
1245	3.540E-04
1620	4.030E-04
1995	4.293E-04

$v_0 = (2.5 \pm 0.2) \times 10^{-7}$ mol/Ls

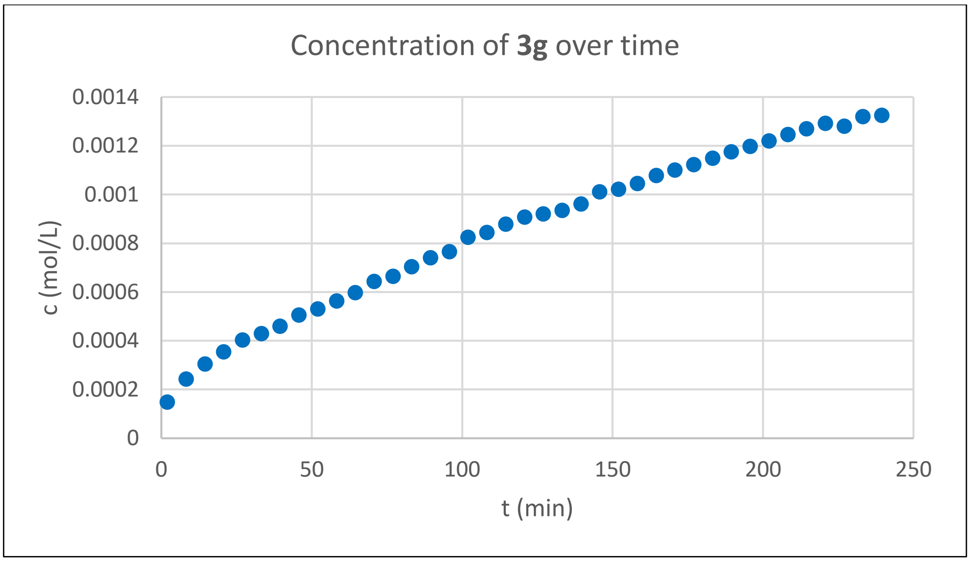

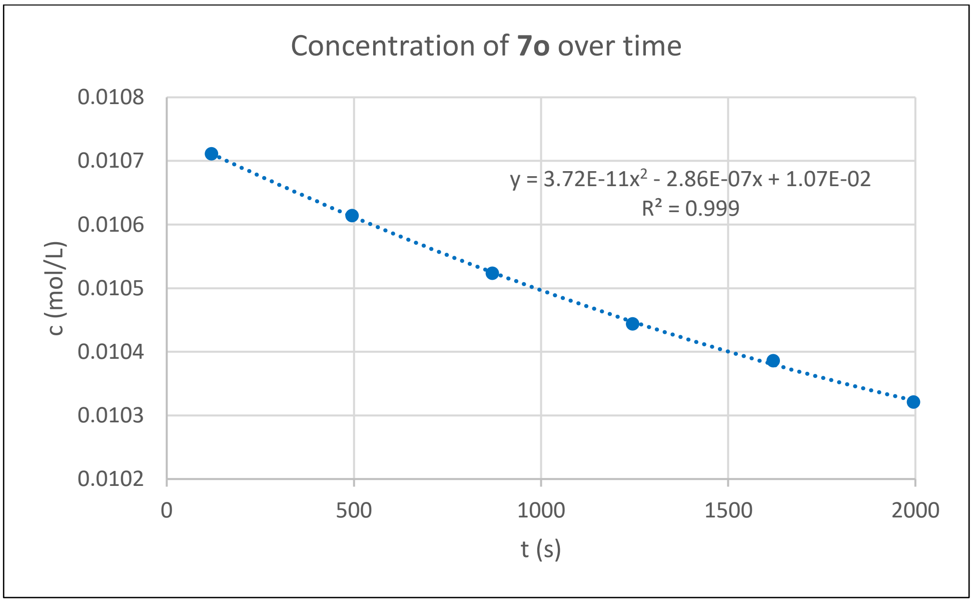

Time [s]	c [mol/L]
120	1.071E-02
495	1.061E-02
870	1.052E-02
1245	1.044E-02
1620	1.039E-02
1995	1.032E-02

$v_0 = -(2.9 \pm 0.1) \times 10^{-7}$ mol/Ls

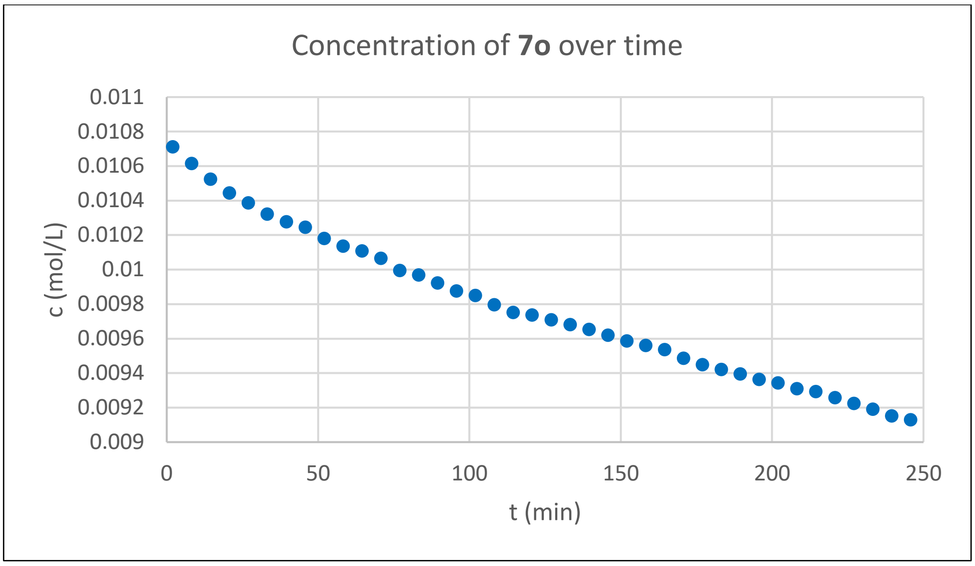
Concentration of 7o over time
c (mol/L)
0.011
0.0108
0.0106
0.0104
0.0102
0.01
0.0098
0.0096
0.0094
0.0092
0.009
0
50
100
150
200
250
t (min)

Transmetallation of 6a and 7c

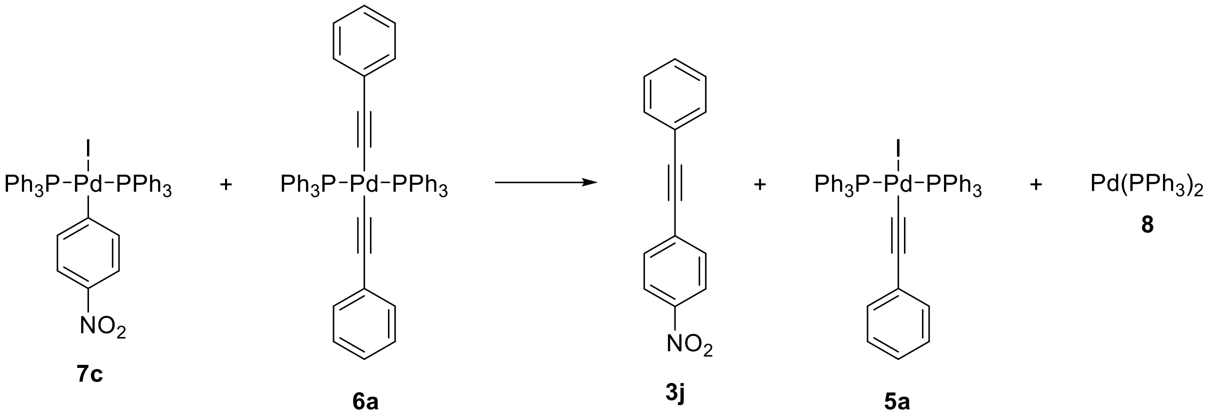

Following *GP15* using bis(triphenylphosphine)palladium(II) (4-nitrophenyl)ide iodide (**7c**) (7.08 mg, 8.05 µmol), bis(triphenylphosphine)palladium(II) bis(phenylethynyl-1-ide) (**6a**) (7.72 mg, 9.29 µmol), $CDCl_3$ (0.800 mL).

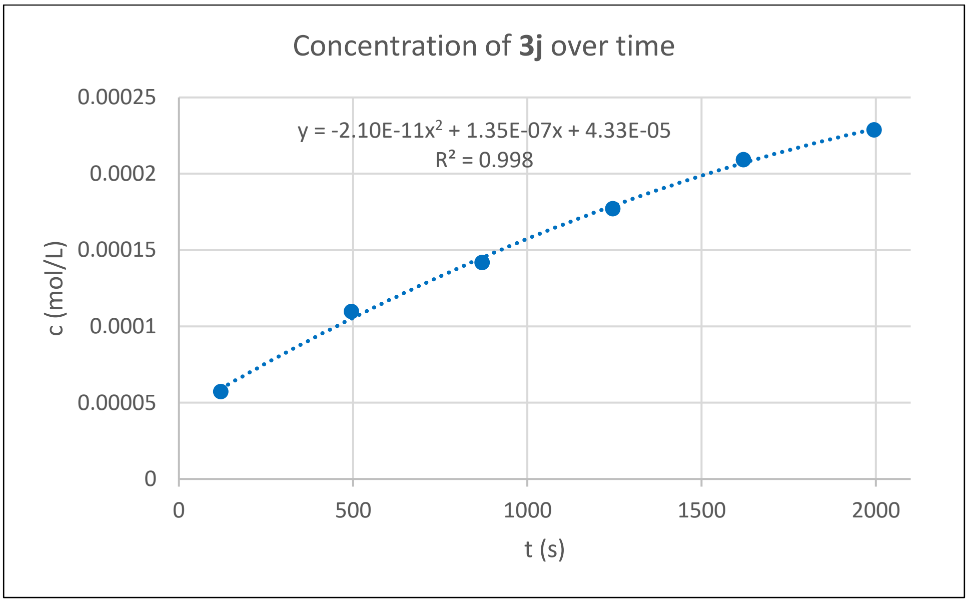

Time [s]	c [mol/L]
120	5.736E-05
495	1.098E-04
870	1.420E-04
1245	1.772E-04
1620	2.094E-04
1995	2.288E-04

$v_0 = (1.4 \pm 0.1) \times 10^{-7}$ mol/Ls

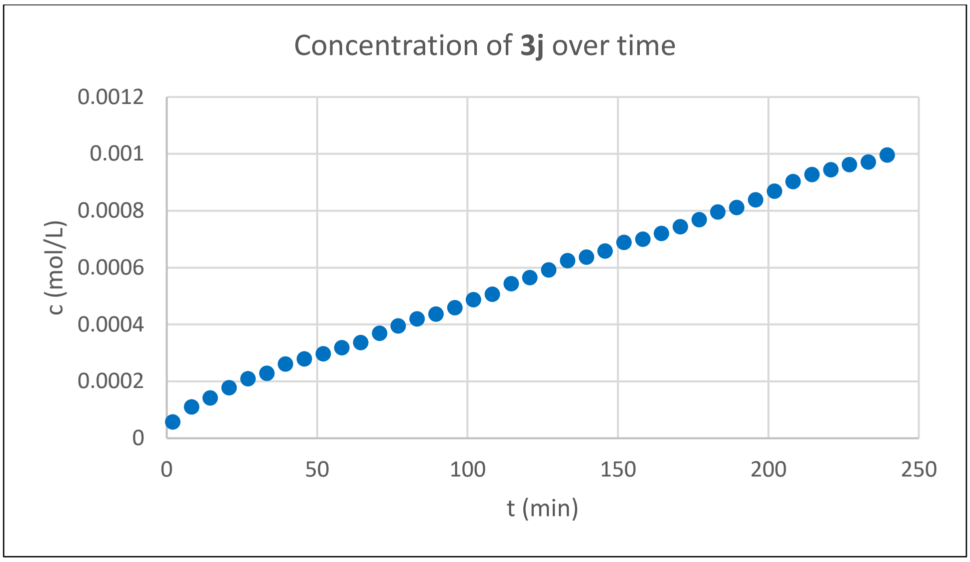

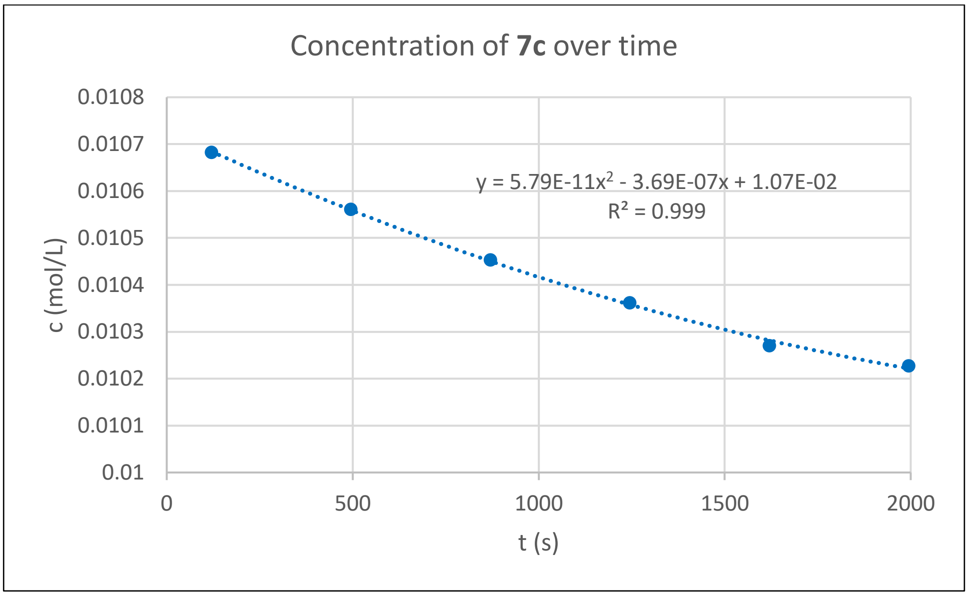

Time [s]	c [mol/L]
120	1.068E-02
495	1.056E-02
870	1.045E-02
1245	1.036E-02
1620	1.027E-02
1995	1.023E-02

$v_0 = -(3.7 \pm 0.2) \times 10^{-7}$ mol/Ls

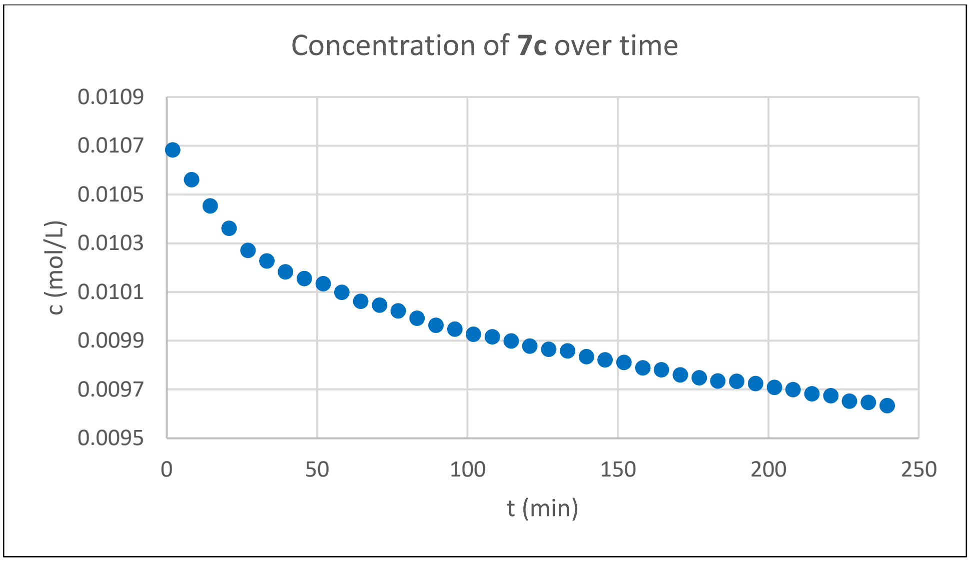
Concentration of **7c** over time
0.0109
0.0107
0.0105
0.0103
0.0101
0.0099
0.0097
0.0095
c (mol/L)
0
50
100
150
200
250
t (min)

Substituent effect of *p*-substituted bis(triphenylphosphine)palladium(II) aryl iodides 7 on transmetallation reaction using Hammett σ values (Figure 8a)

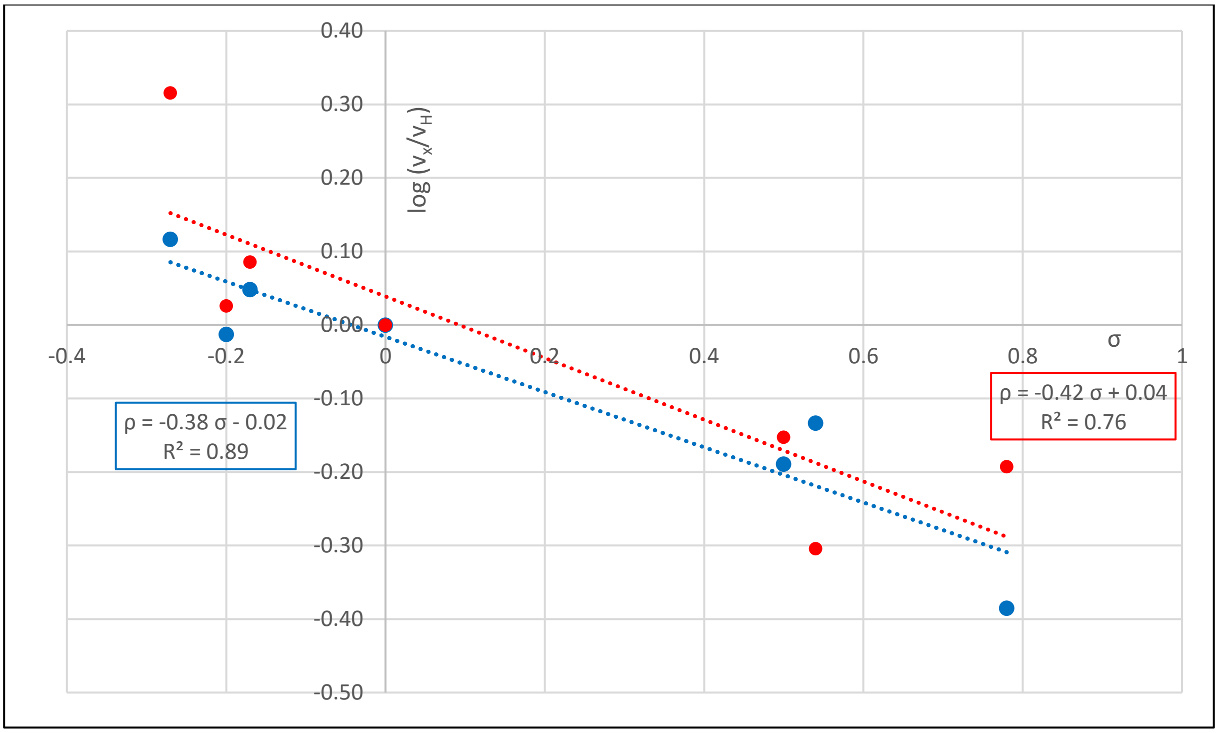

Figure 8a. Hammett plot for product **3** formation and palladium oxidative addition complex **7** conversion for transmetallation reaction between **7** and **6** using Hammett σ values.

Bis(triphenylphosphine)palladium aryl iodide (7) conversion Hammett correlation

Substituent	σ	v [mol/Ls]	$\log(v_x/v_H)$
OMe	-0.27	-1.2E-06	0.3155
*t*Bu	-0.20	-6.1E-07	0.0258
Me	-0.17	-7.0E-07	0.0858
CH_2OH	0.00	-5.8E-07	0.0000
COMe	0.50	-4.0E-07	-0.1528
CF_3	0.54	-2.9E-07	-0.3043
NO_2	0.78	-3.7E-07	-0.1926

Product (3) formation Hammett correlation

Substituent	σ	v [mol/Ls]	$\log(v_x/v_0)$
OMe	-0.27	4.4E-07	0.1164
*t*Bu	-0.20	3.3E-07	-0.0130
Me	-0.17	3.8E-07	0.0483
CH_2OH	0.00	3.4E-07	0.0000
COMe	0.50	2.2E-07	-0.1891
CF_3	0.54	2.5E-07	-0.1335
NO_2	0.78	1.4E-07	-0.3854

The σ-values were taken from:

Hansch, C., Leo, A. & Taft, R. W. A Survey of Hammett Substituent Constants and Resonance and Field Parameters. *Chem. Rev.* **91**, 165–195 (1991).

Substituent effect of *p*-substituted bis(triphenylphosphine)palladium(II) aryl iodides 7 on transmetallation reaction using corrected Hammett σ values

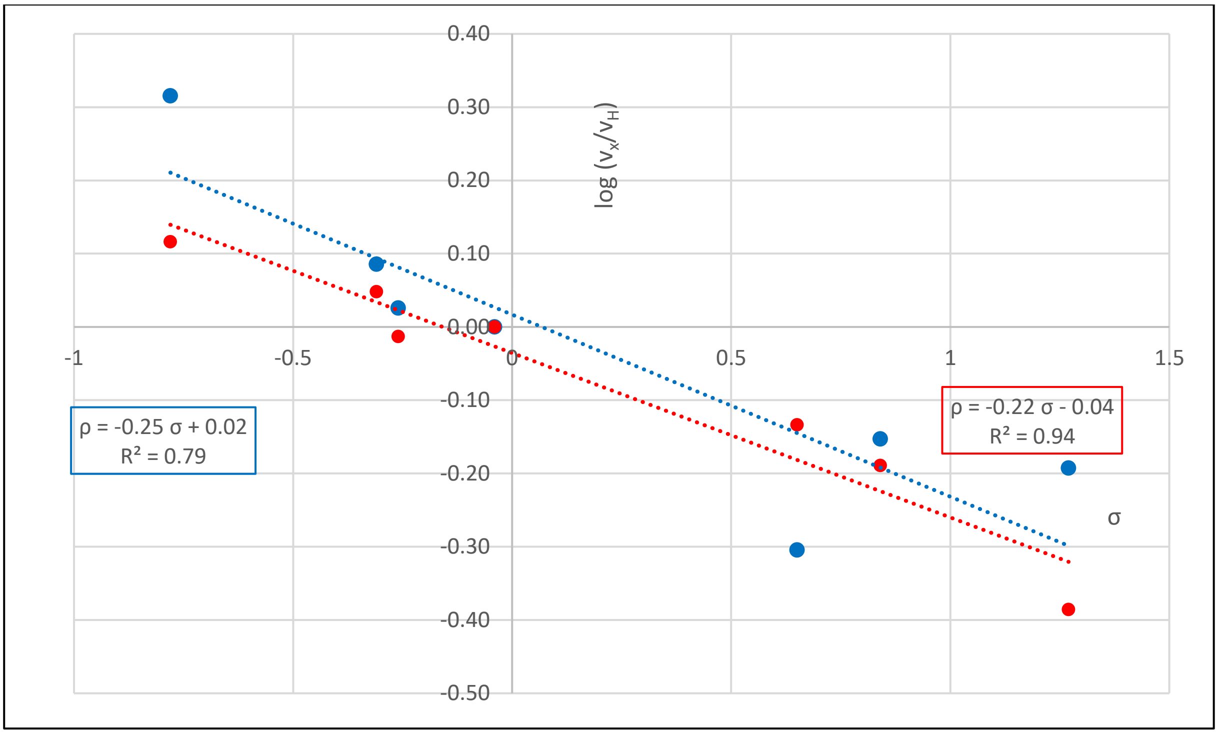

Supplementary Figure 18. Hammett plot for product **3** formation and palladium oxidative addition complex **7** conversion for transmetallation reaction between **7** and **6** using corrected Hammett σ values.

Bis(triphenylphosphine)palladium aryl iodide (7) conversion Hammett correlation

Substituent	σ	v [mol/Ls]	$\log(v_x/v_H)$
OMe	-0.78	-1.2E-06	0.3155
*t*Bu	-0.26	-6.1E-07	0.0258
Me	-0.31	-7.0E-07	0.0858
CH_2OH	-0.04	-5.8E-07	0.0000
COMe	0.84	-4.0E-07	-0.1528
CF_3	0.65	-2.9E-07	-0.3043
NO_2	1.27	-3.7E-07	-0.1926

Product (3) formation Hammett correlation

Substituent	σ	v [mol/Ls]	$\log(v_x/v_0)$
OMe	-0.78	4.4E-07	0.1164
*t*Bu	-0.26	3.3E-07	-0.0130
Me	-0.31	3.8E-07	0.0483
CH_2OH	-0.04	3.4E-07	0.0000
COMe	0.84	2.2E-07	-0.1891
CF_3	0.65	2.5E-07	-0.1335
NO_2	1.27	1.4E-07	-0.3854

The σ-values were taken from:

Hansch, C., Leo, A. & Taft, R. W. A Survey of Hammett Substituent Constants and Resonance and Field Parameters. *Chem. Rev.* **91**, 165–195 (1991).

The effect of *para*-substituent (R^2) in 6 on the rate of transmetallation

Transmetallation reactions were carried out in a sealed NMR tube at 302.0 K. The starting concentration of bis(triphenylphosphine)palladium bisacetylide (**6**) was 0.01 M. Bis(triphenylphosphine)palladium (4-methylphenyl)ide iodide (**7a**) was added in excess amount (1.15 equiv.). ^{1}H-NMR spectra were recorded in intervals (6.25 min). The concentration of the product **3** was determined by comparison of integrals of characteristic resonances of product 3 with integrals of resonances of an internal standard (1,3,5-trimethoxybenzene).

General procedure 16 (*GP16*) – Transmetallation reactions between bis(triphenylphosphine)palladium (4-methylphenyl)ide iodide (7a) and palladium bisacetylides 6.

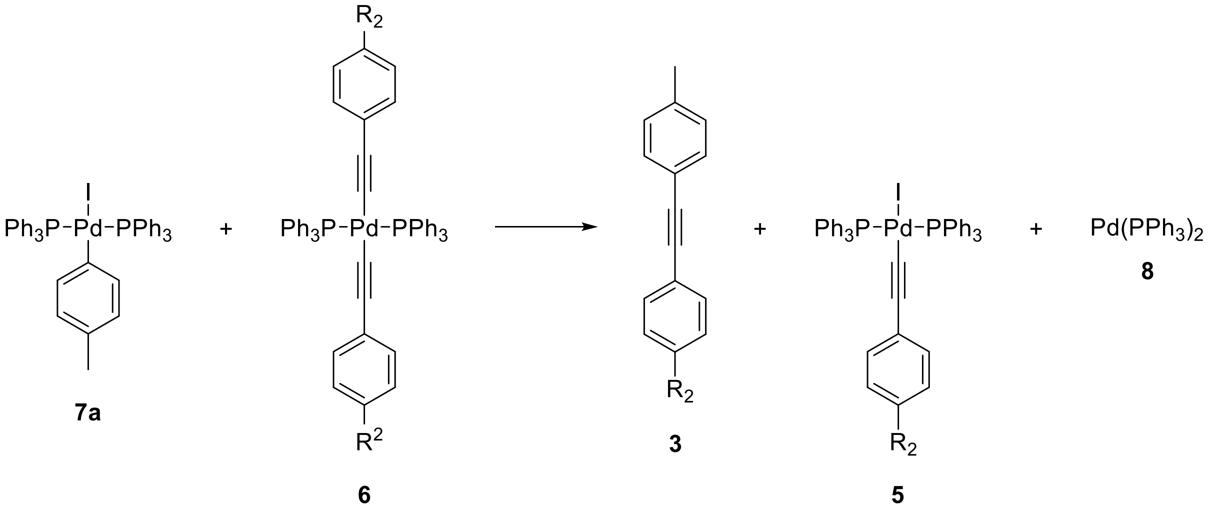

In a vial a solution of bis(triphenylphosphine)palladium bisacetylide (**6**) (1 equiv., 0.01 M) in $CDCl_3$ was prepared under argon atmosphere. Bis(triphenylphosphine)palladium (4-methylphenyl)ide iodide (**7a**) (1.15 equiv.) was added to this solution in one portion. The reaction mixture was sonicated for 0.5 min and transferred to NMR tube, flushed with argon and sealed. NMR spectra were acquired 6.25 min in intervals.

Transmetallation of 6d and 7a

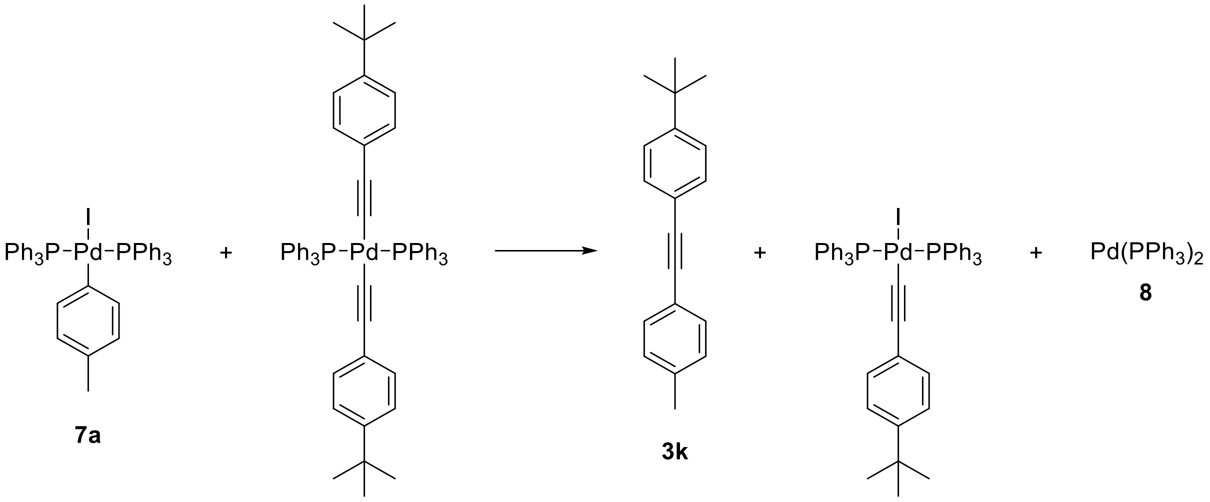

Following *GP16* using bis(triphenylphosphine)palladium(II) (4-methylphenyl)ide iodide (**7a**) (7.75 mg, 9.13 µmol), bis(triphenylphosphine)palladium(II) bis((4-*tert*-butylphenyl)ethyn-1-ide) (**6d**) (7.52 mg, 7.95 µmol), $CDCl_3$ (0.800 mL).

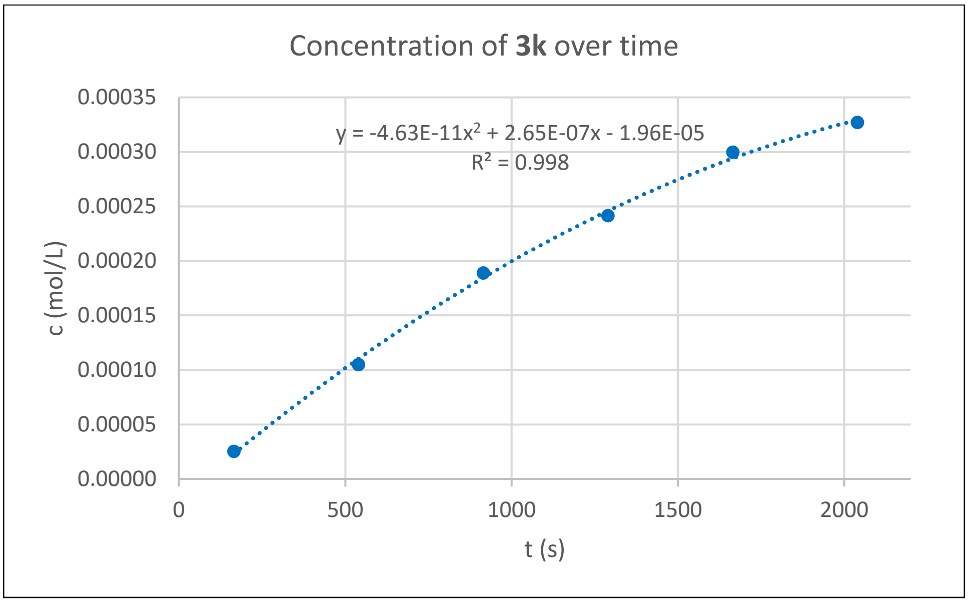

Time [s]	c [mol/L]
165	2.530E-05
540	1.048E-04
915	1.887E-04
1290	2.415E-04
1665	2.996E-04
2040	3.269E-04

$v_0 = (2.7 \pm 0.2) \times 10^{-7}$ mol/Ls

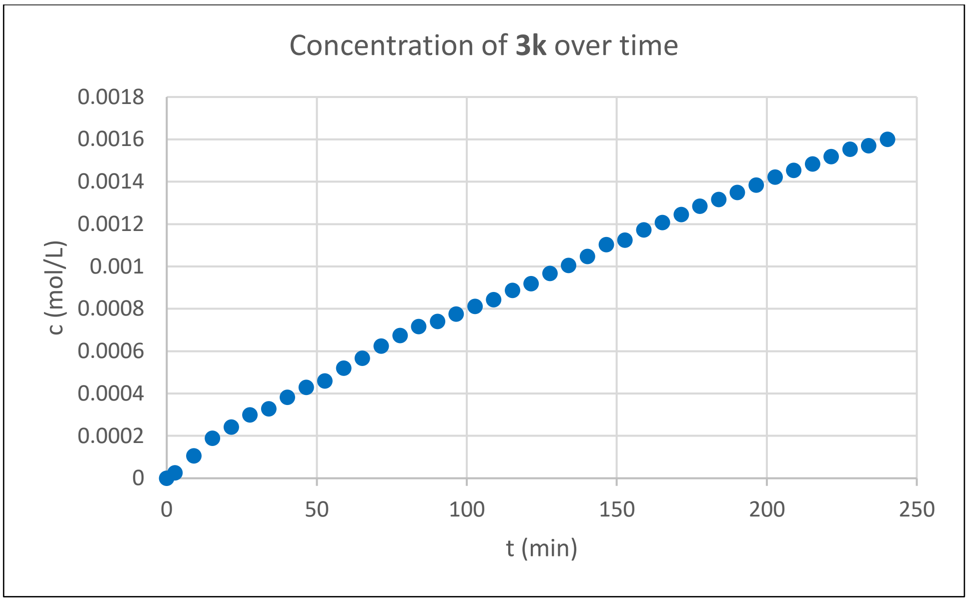

Decomposition rate of **6d** was estimated as negative rate of **3k** formation due to poor solubility of bisacetylide **6d**.

Transmetallation of 6e and 7a

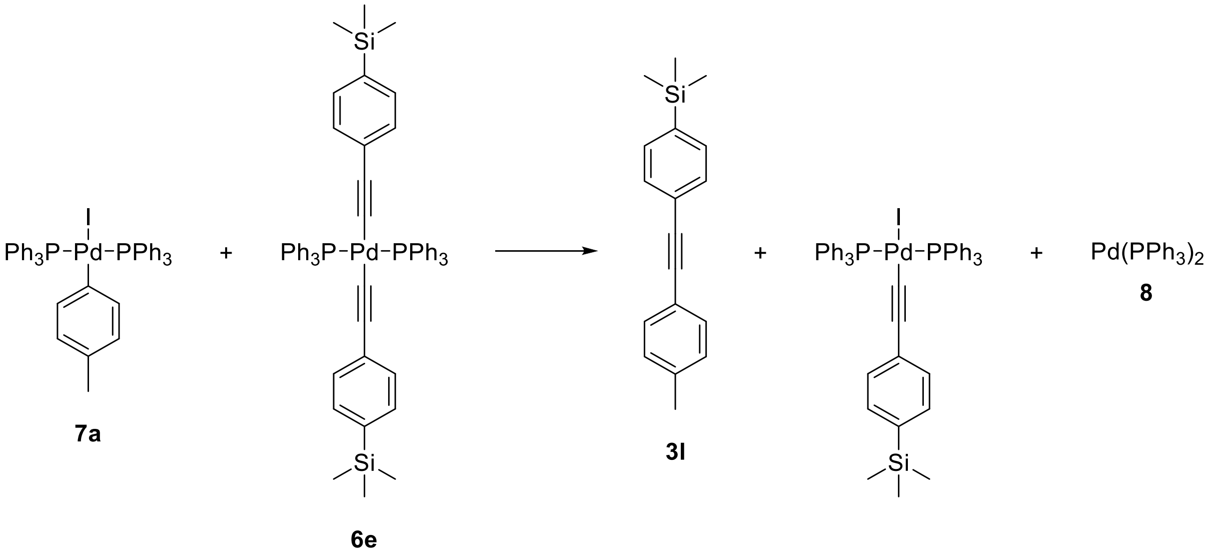

Following *GP16* using bis(triphenylphosphine)palladium(II) (4-methylphenyl)ide iodide (**7a**) (7.74 mg, 9.12 µmol), bis(triphenylphosphine)palladium(II) bis((4-(trimethylsilyl)phenyl)ethyn-1-ide) (**6e**) (7.85 mg, 8.03 µmol), $CDCl_3$ (0.800 mL).

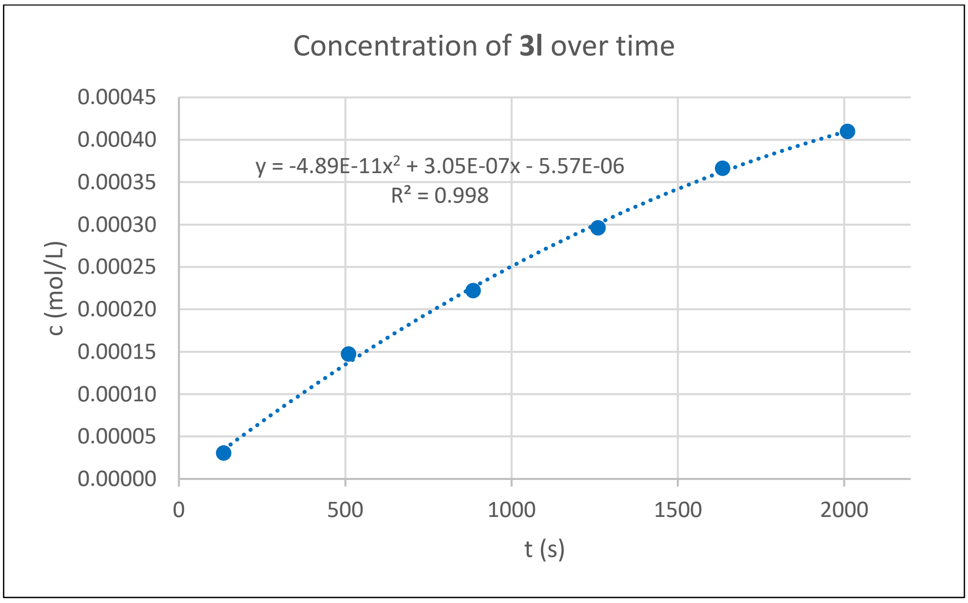

Time [s]	c [mol/L]
135	3.054E-05
510	1.472E-04
885	2.220E-04
1260	2.962E-04
1635	3.663E-04
2010	4.097E-04

$v_0 = (3.1 \pm 0.2) \times 10^{-7}$ mol/Ls

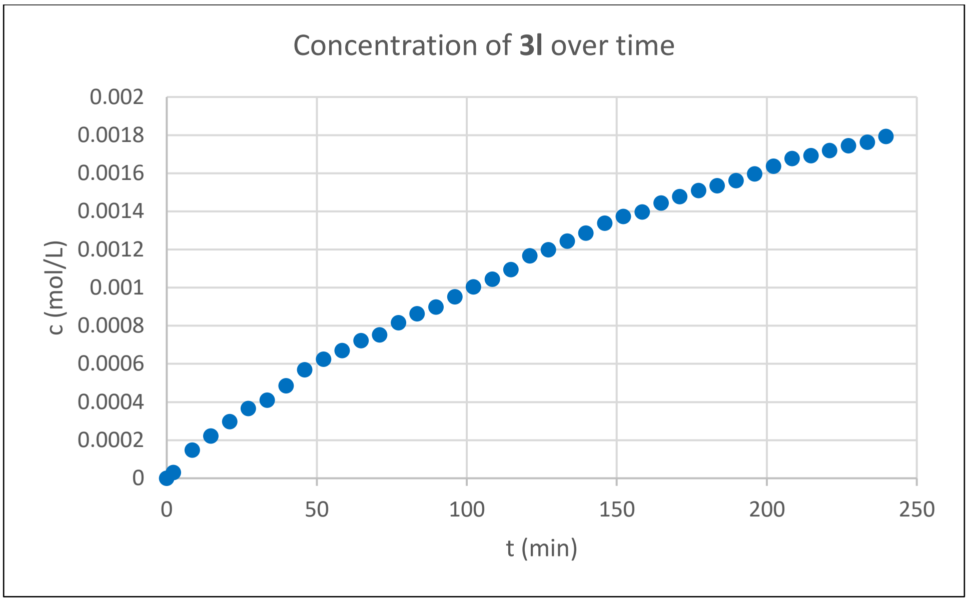

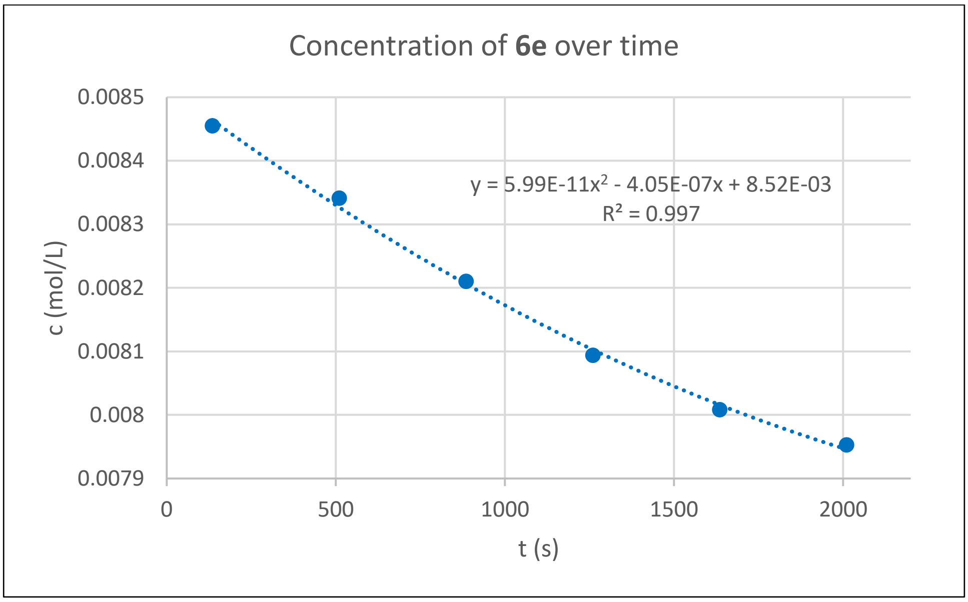

Time [s]	c [mol/L]
135	8.455E-03
510	8.341E-03
885	8.210E-03
1260	8.094E-03
1635	8.008E-03
2010	7.953E-03

$v_0 = -(4.0 \pm 0.3) \times 10^{-7}$ mol/Ls

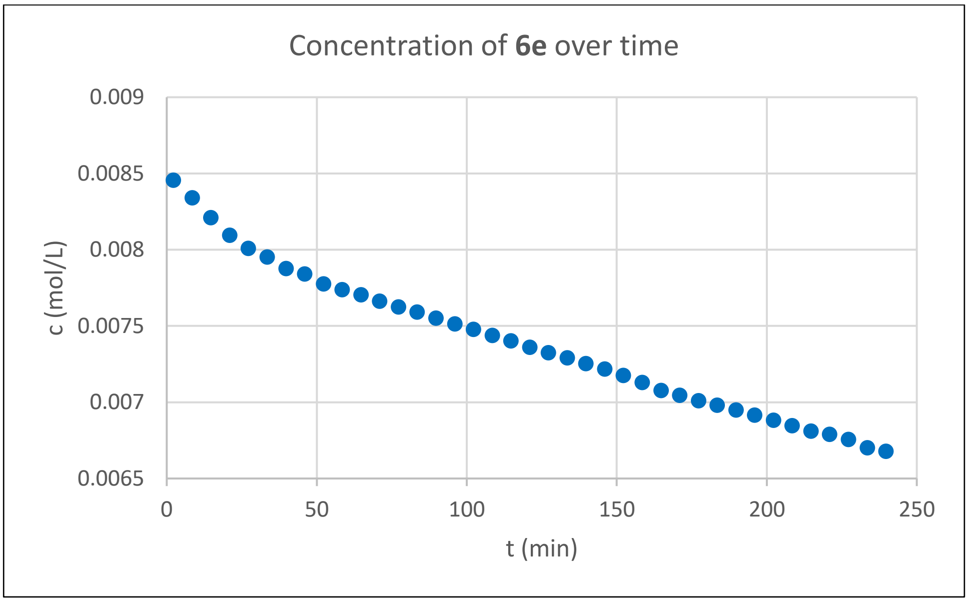
Concentration of 6e over time
c (mol/L)
0.009
0.0085
0.008
0.0075
0.007
0.0065
0
50
100
150
200
250
t (min)

Transmetallation of 6a and 7a

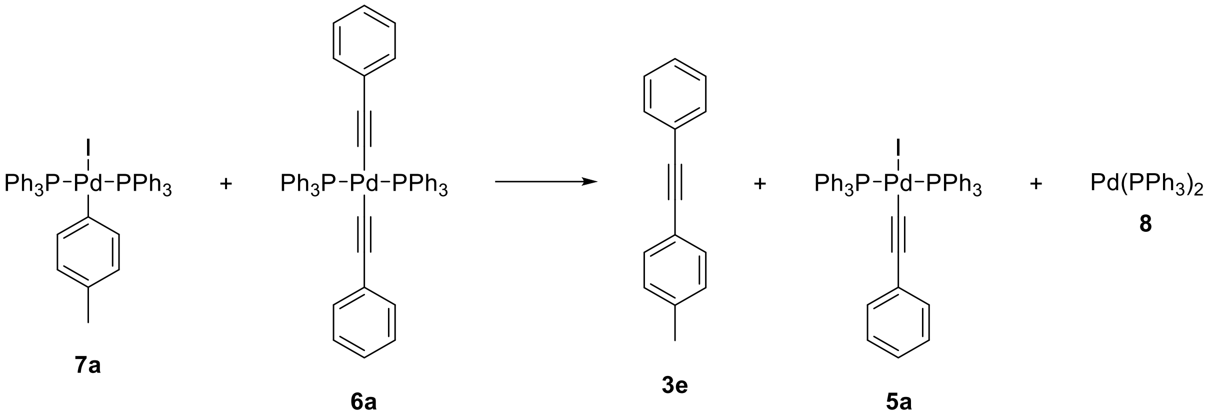

Following *GP16* using bis(triphenylphosphine)palladium(II) (4-methylphenyl)ide iodide (**7a**) (7.77 mg, 9.15 µmol), bis(triphenylphosphine)palladium(II) bis(phenylethynide) (**6a**) (6.67 mg, 8.00 µmol), $CDCl_3$ (0.800 mL).

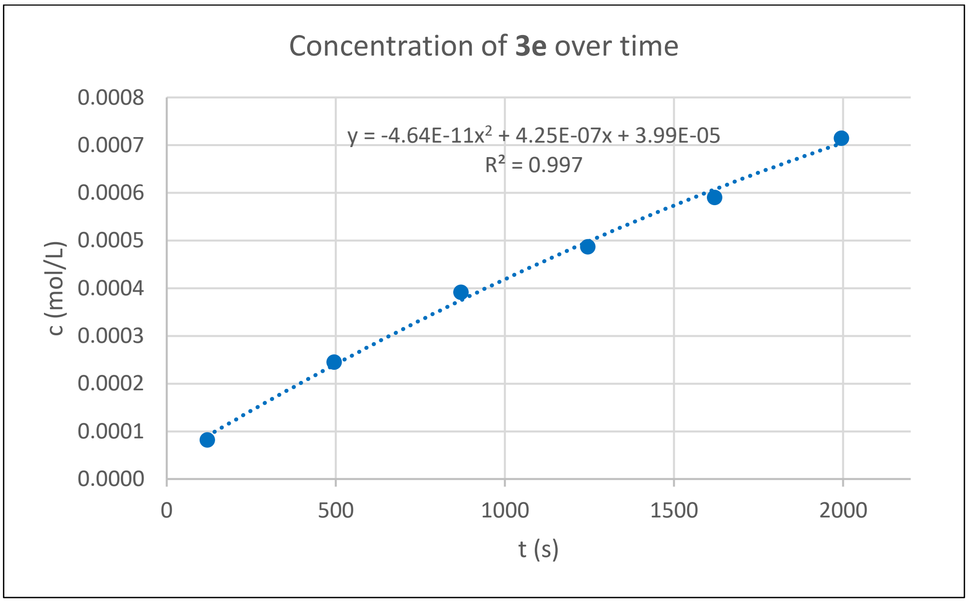

Time [s]	c [mol/L]
120	8.239E-05
495	2.455E-04
870	3.916E-04
1245	4.872E-04
1620	5.908E-04
1995	7.149E-04

$v_0 = (4.3 \pm 0.4) \times 10^{-7}$ mol/Ls

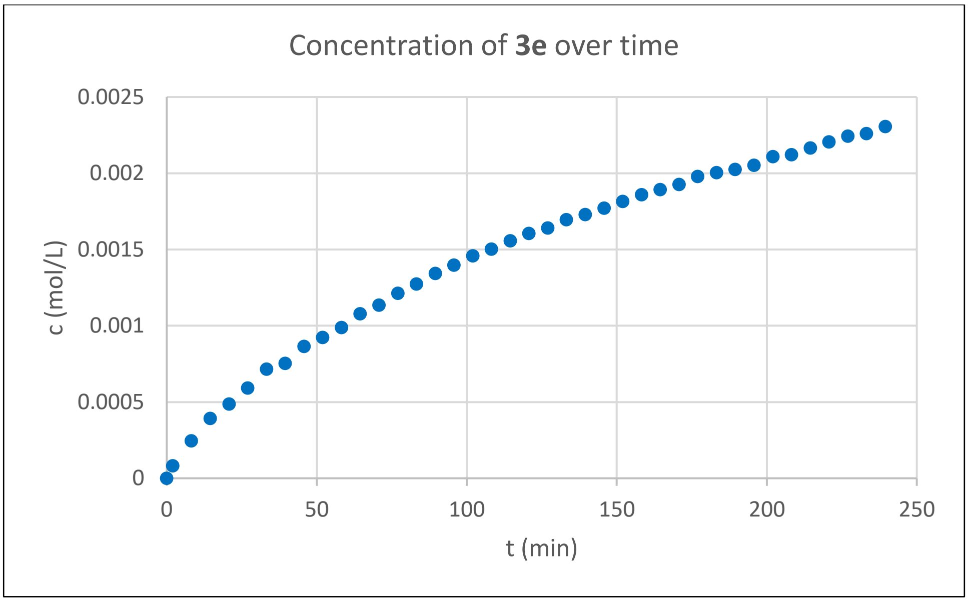

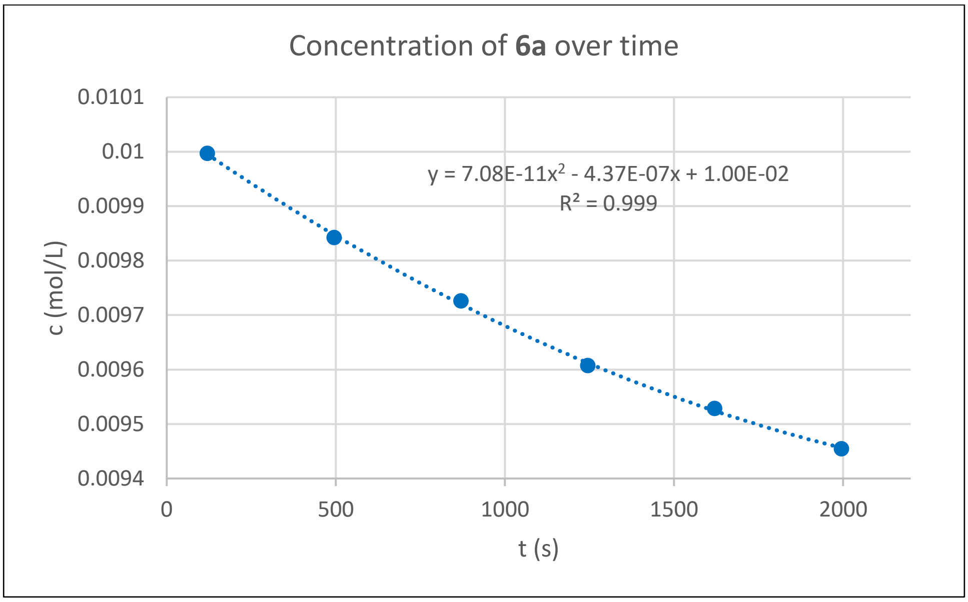

Time [s]	c [mol/L]
120	9.997E-03
495	9.842E-03
870	9.726E-03
1245	9.607E-03
1620	9.528E-03
1995	9.455E-03

$v_0 = -(4.4 \pm 0.2) \times 10^{-7}$ mol/Ls

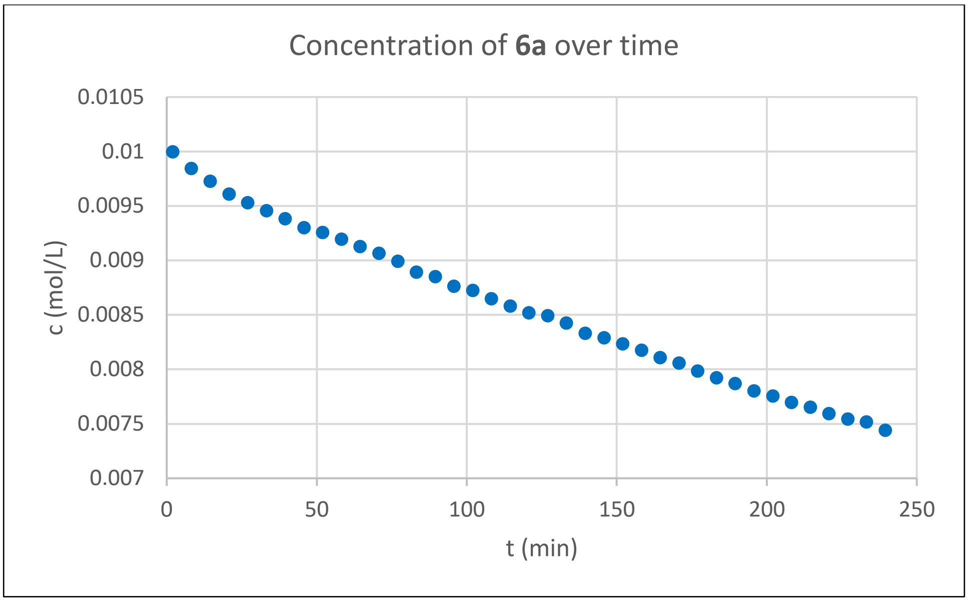
Concentration of **6a** over time
0.0105
0.01
0.0095
0.009
0.0085
0.008
0.0075
0.007
c (mol/L)
0
50
100
150
200
250
t (min)

Transmetallation of 6f and 7a

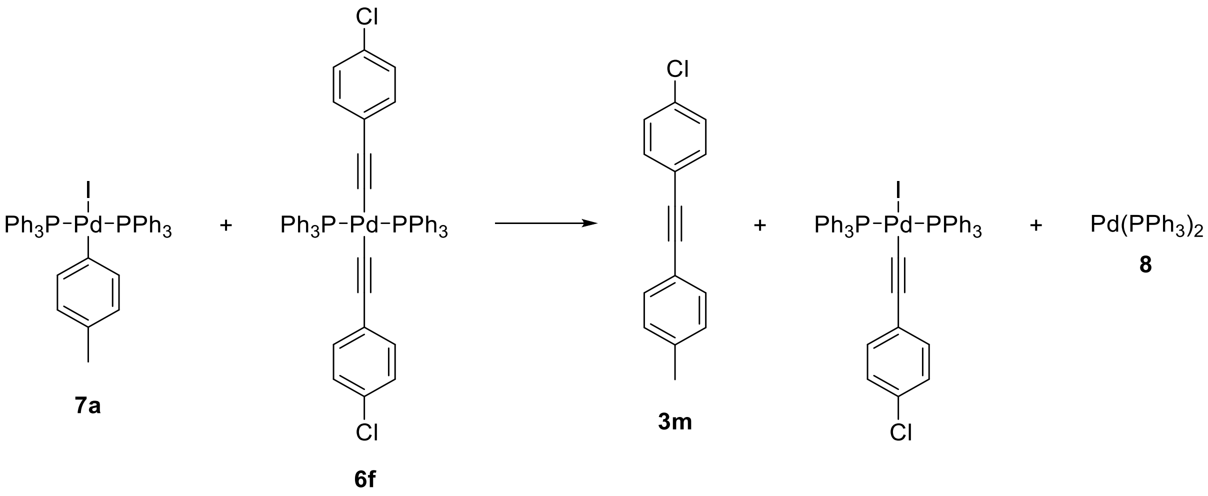

Following *GP16* using bis(triphenylphosphine)palladium(II) (4-methylphenyl)ide iodide (**7a**) (7.80 mg, 9.19 µmol), bis(triphenylphosphine)palladium(II) bis((4-chlorophenyl)ethyn-1-ide) (**6f**) (7.26 mg, 8.05 µmol), $CDCl_3$ (0.800 mL).

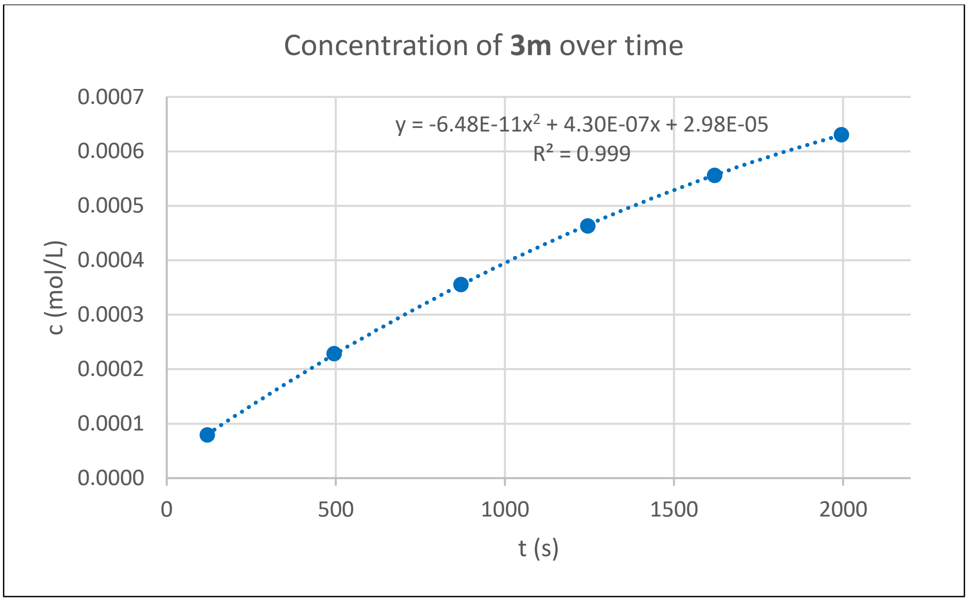

Time [s]	c [mol/L]
120	7.941E-05
495	2.285E-04
870	3.556E-04
1245	4.631E-04
1620	5.560E-04
1995	6.304E-04

$v_0 = (4.30 \pm 0.04) \times 10^{-7}$ mol/Ls

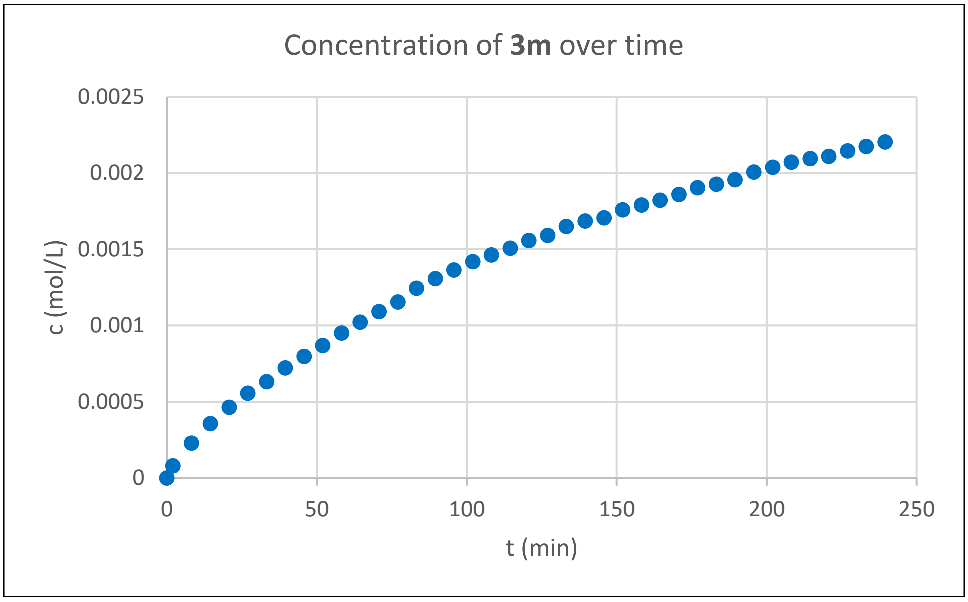

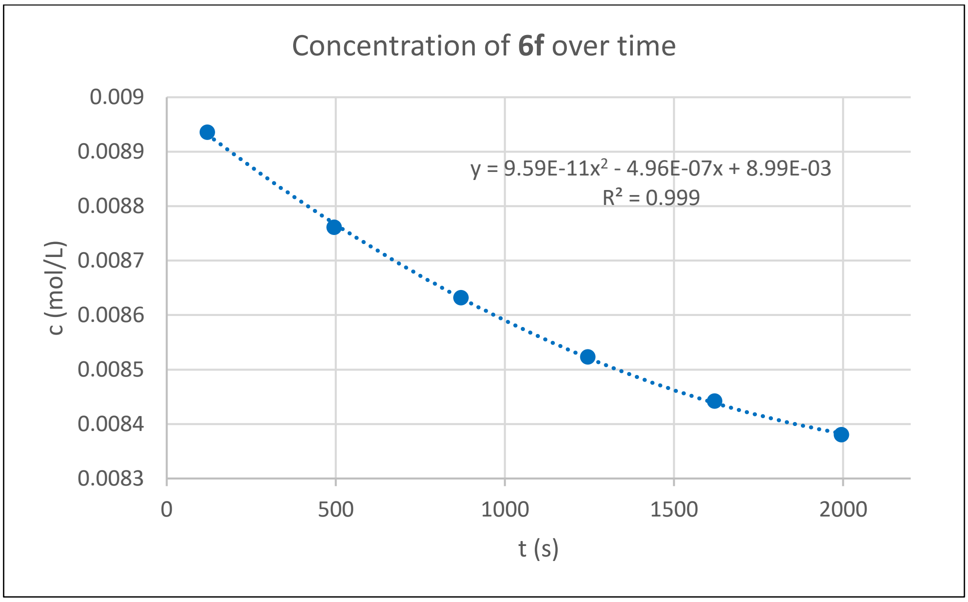

Time [s]	c [mol/L]
120	8.936E-03
495	8.761E-03
870	8.632E-03
1245	8.523E-03
1620	8.442E-03
1995	8.380E-03

$v_0 = -(5.0 \pm 0.1) \times 10^{-7}$ mol/Ls

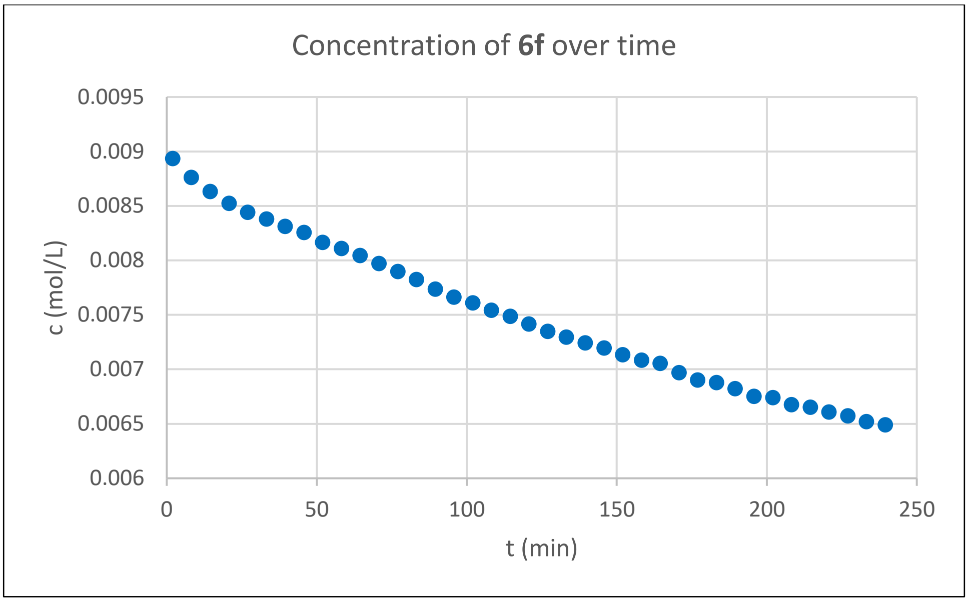
Concentration of **6f** over time
0.0095
0.009
0.0085
0.008
0.0075
0.007
0.0065
0.006
c (mol/L)
0
50
100
150
200
250
t (min)

Transmetallation of 6g and 7a

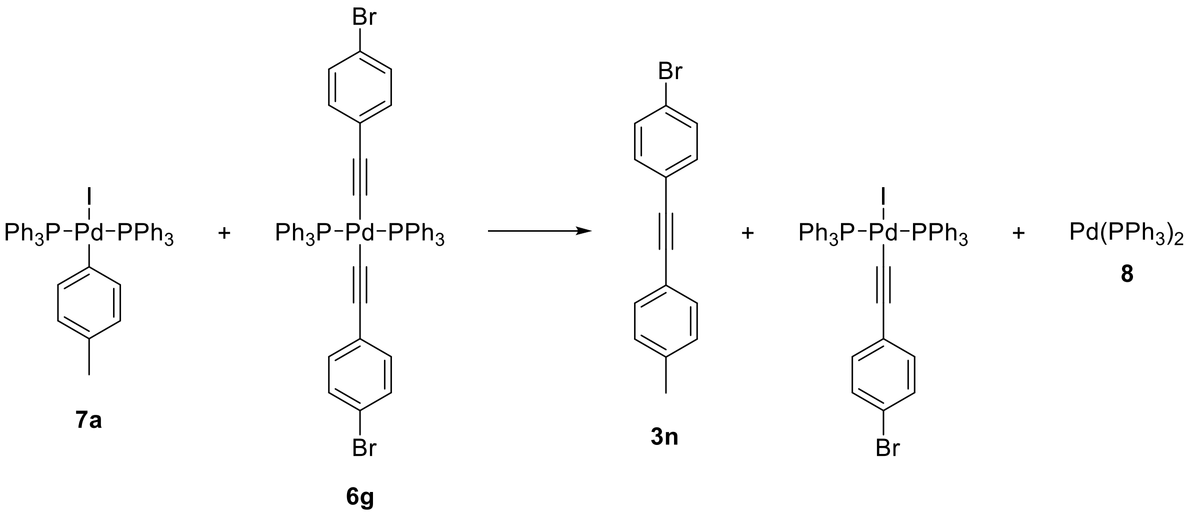

Following *GP16* using bis(triphenylphosphine)palladium(II) (4-methylphenyl)ide iodide (**7a**) (7.83 mg, 9.22 µmol), bis(triphenylphosphine)palladium(II) bis((4-bromophenyl)ethyn-1-ide) (**6g**) (7.98 mg, 8.05 µmol), $CDCl_3$ (0.800 mL).

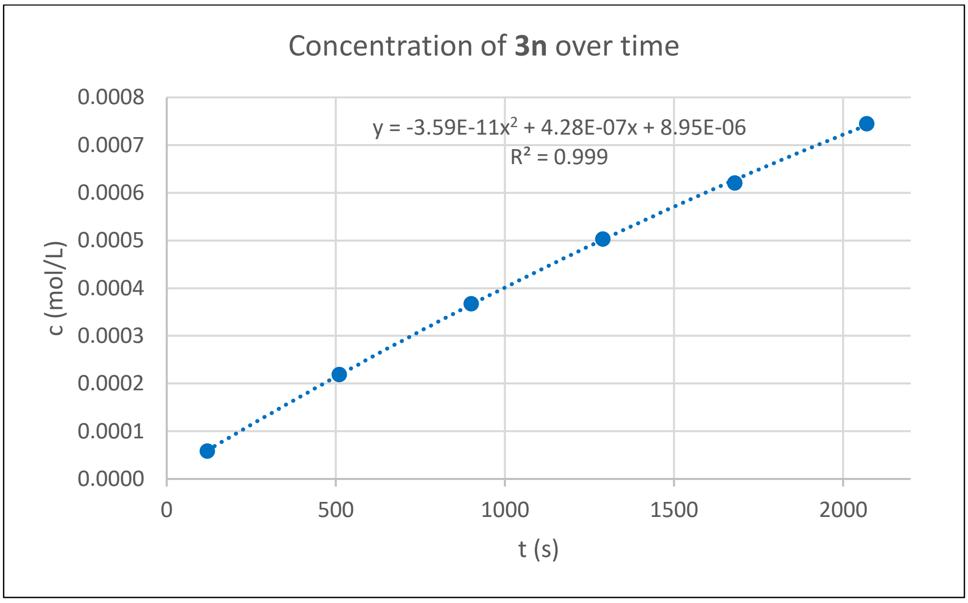

Time [s]	c [mol/L]
120	5.827E-05
495	2.194E-04
870	3.674E-04
1245	5.034E-04
1620	6.204E-04
1995	7.451E-04

$v_0 = (4.3 \pm 0.1) \times 10^{-7}$ mol/Ls

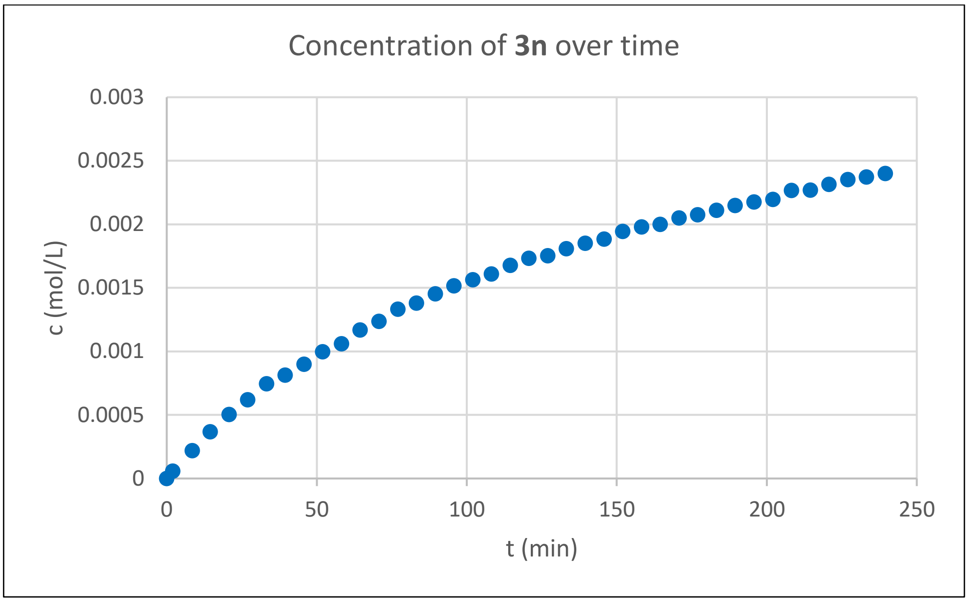

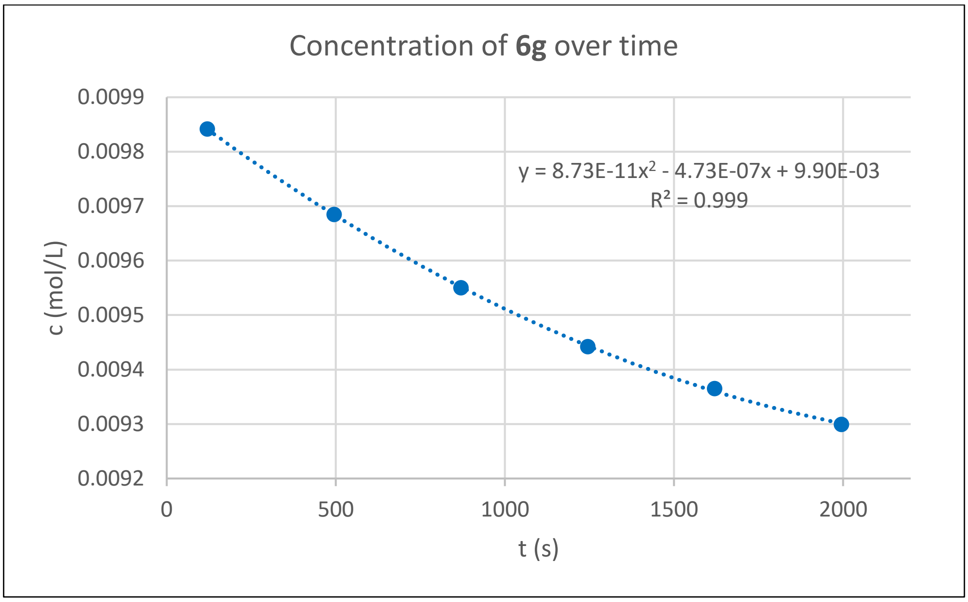

Time [s]	c [mol/L]
120	9.842E-03
495	9.685E-03
870	9.550E-03
1245	9.442E-03
1620	9.365E-03
1995	9.299E-03

$v_0 = -(4.73 \pm 0.08) \times 10^{-7}$ mol/Ls

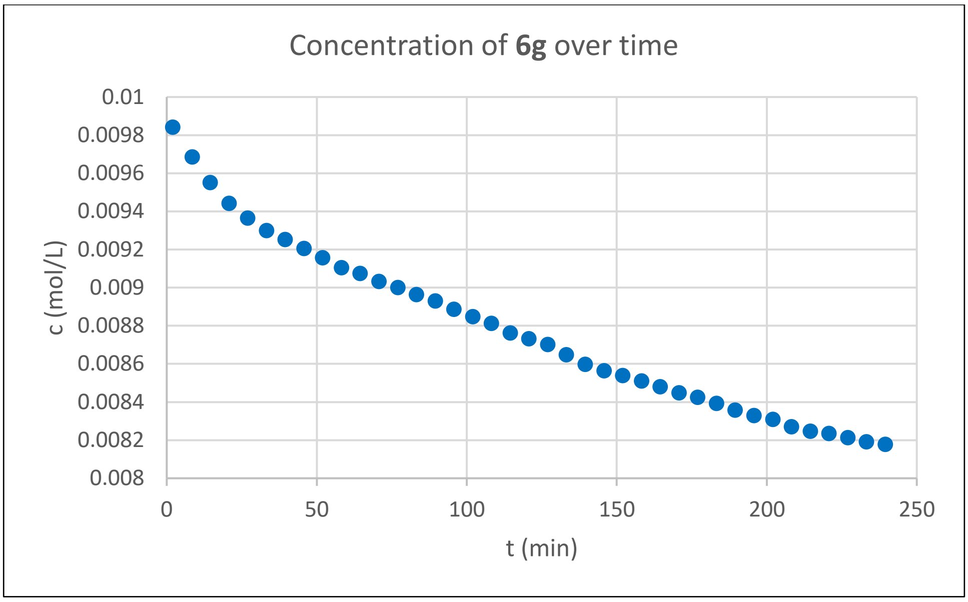
Concentration of **6g** over time
c (mol/L)
0.01
0.0098
0.0096
0.0094
0.0092
0.009
0.0088
0.0086
0.0084
0.0082
0.008
0
50
100
150
200
250
t (min)

Transmetallation of 6j and 7a

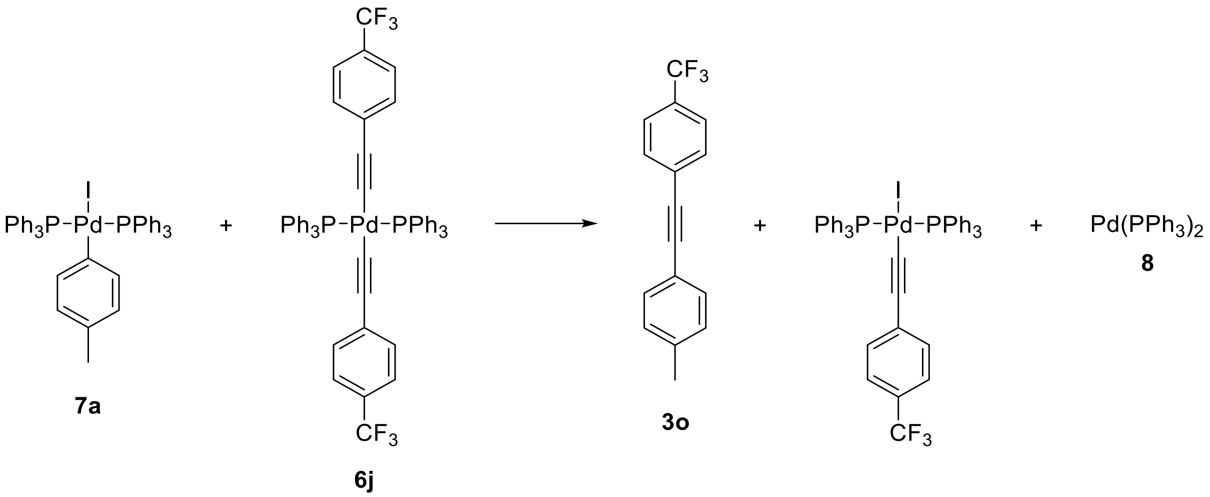

Following *GP16* using bis(triphenylphosphine)palladium(II) (4-methylphenyl)ide iodide (**7a**) (7.76 mg, 9.14 µmol), bis(triphenylphosphine)palladium(II) bis((4-(trifluoromethyl)phenyl)ethyn-1-ide) (**6j**) (7.70 mg, 7.94 µmol), $CDCl_3$ (0.800 mL).

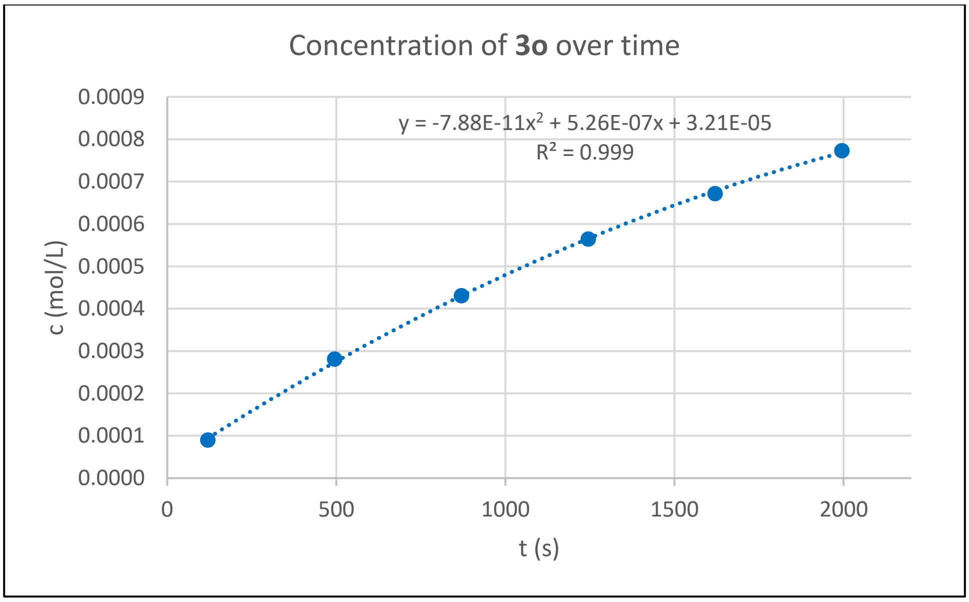

Time [s]	c [mol/L]
120	8.945E-05
495	2.811E-04
870	4.307E-04
1245	5.641E-04
1620	6.715E-04
1995	7.727E-04

$v_0 = (5.3 \pm 0.2) \times 10^{-7}$ mol/Ls

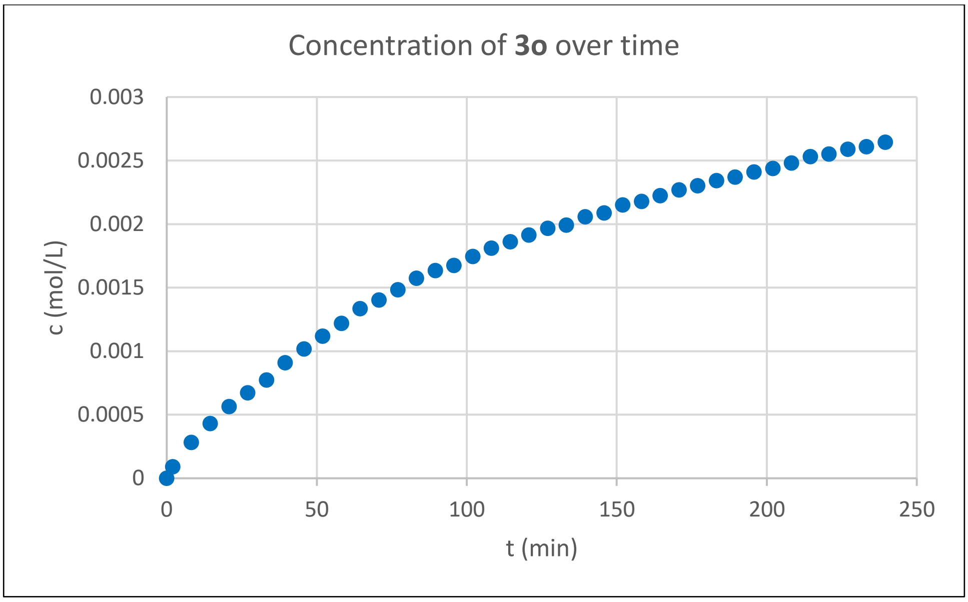

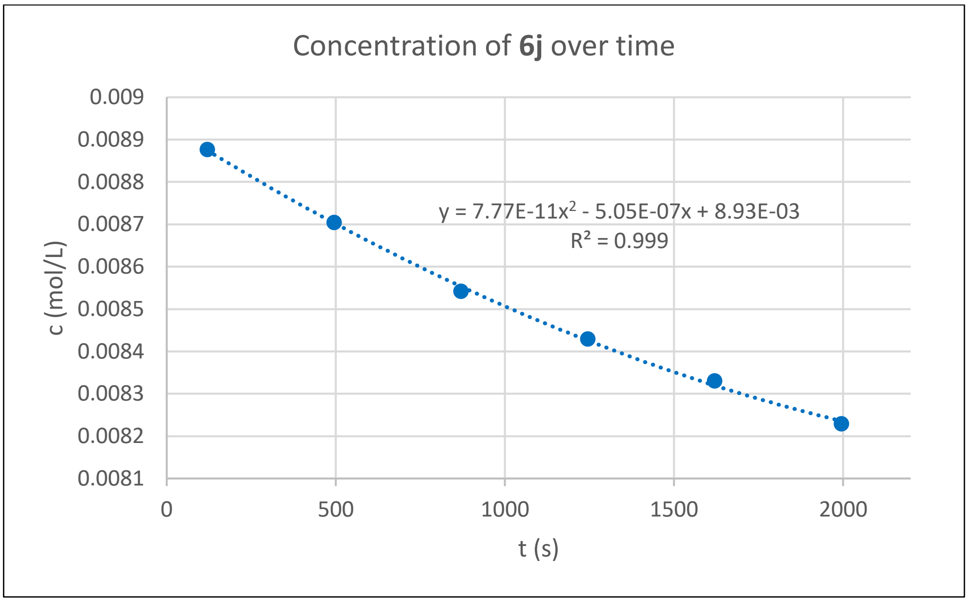

Time [s]	c [mol/L]
120	8.945E-05
495	2.811E-04
870	4.307E-04
1245	5.641E-04
1620	6.715E-04
1995	7.727E-04

$v_0 = -(5.1 \pm 0.3) \times 10^{-7}$ mol/Ls

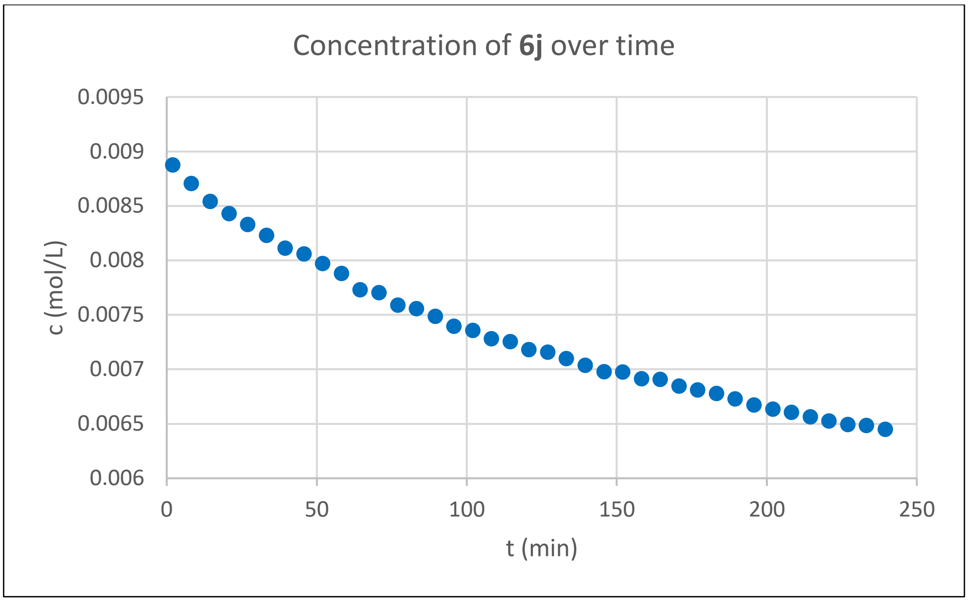

Concentration of **6j** over time
0.0095
0.009
0.0085
0.008
0.0075
0.007
0.0065
0.006
c (mol/L)
0
50
100
150
200
250
t (min)

Transmetallation of 6h and 7a

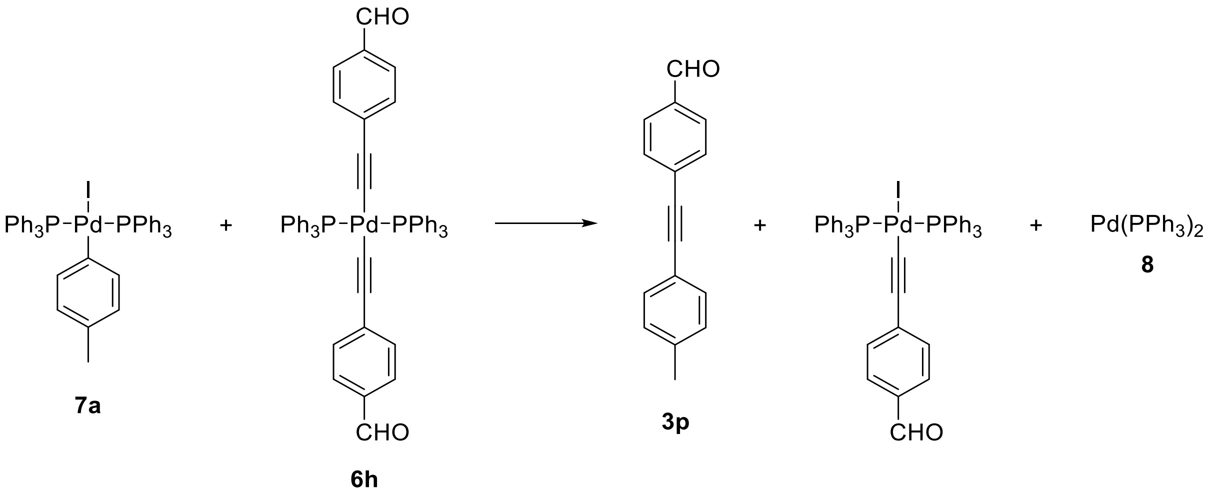

Following *GP16* using bis(triphenylphosphine)palladium(II) (4-methylphenyl)ide iodide (**7a**) (7.77 mg, 9.15 µmol), bis(triphenylphosphine)palladium(II) bis((4-formylphenyl)ethyn-1-ide) (**6h**) (7.13 mg, 8.02 µmol), $CDCl_3$ (0.800 mL).

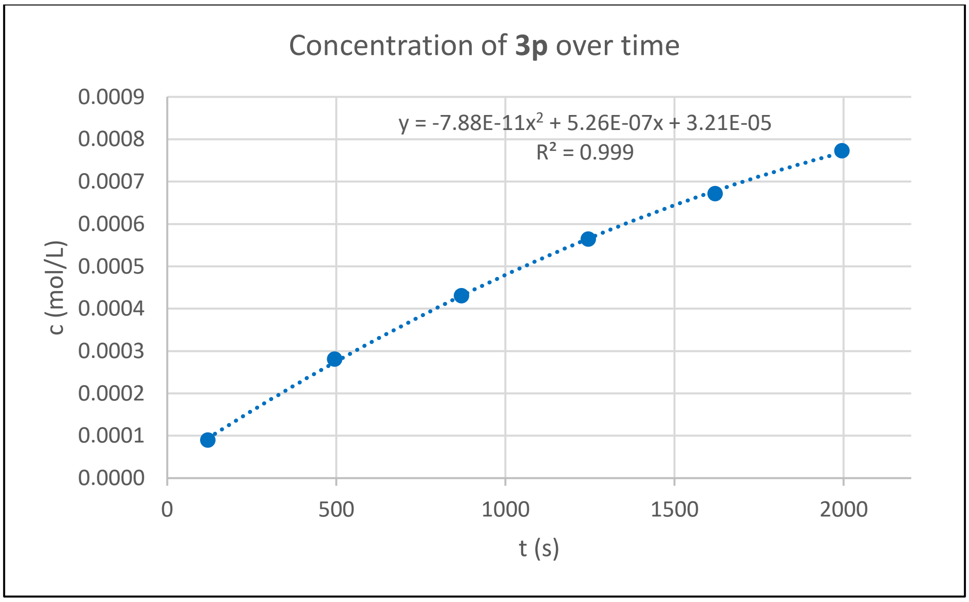

Time [s]	c [mol/L]
132	1.023E-04
507	3.829E-04
882	5.636E-04
1257	7.331E-04
1632	8.828E-04
2007	1.013E-03

$v_0 = (7.1 \pm 0.5) \times 10^{-7}$ mol/Ls

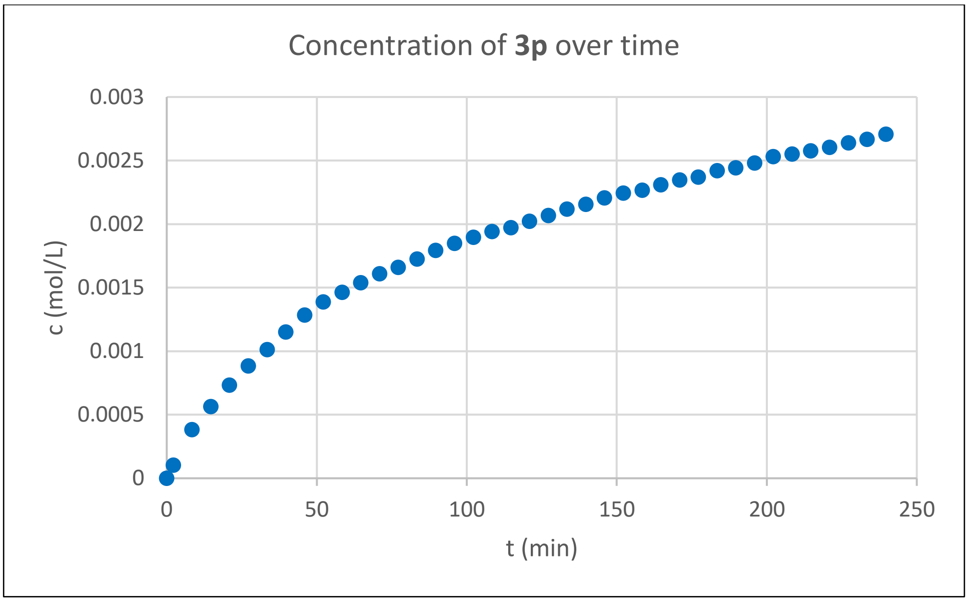

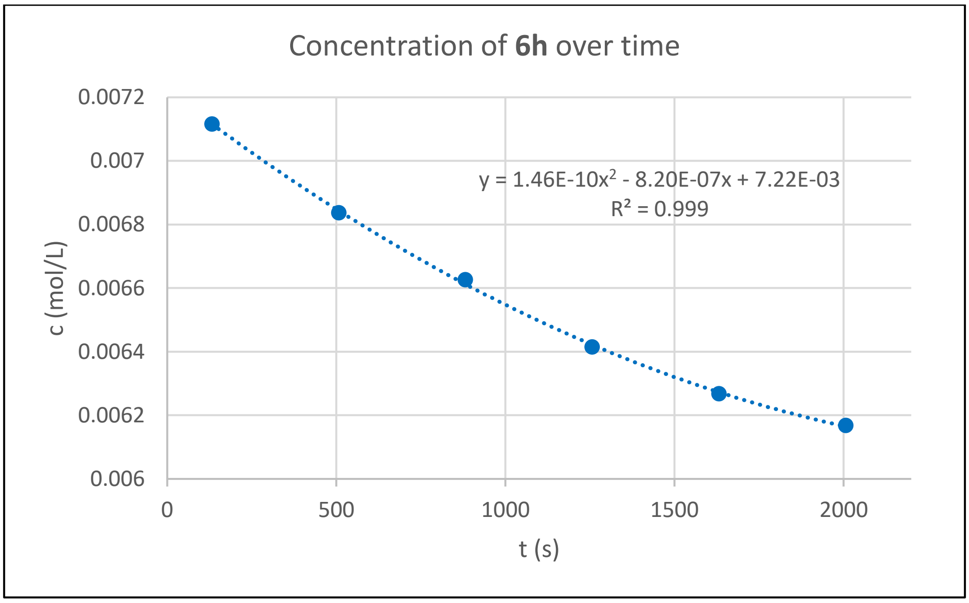

Time [s]	c [mol/L]
132	1.023E-04
507	3.829E-04
882	5.636E-04
1257	7.331E-04
1632	8.828E-04
2007	1.013E-03

$v_0 = -(8.2 \pm 0.3) \times 10^{-7}$ mol/Ls

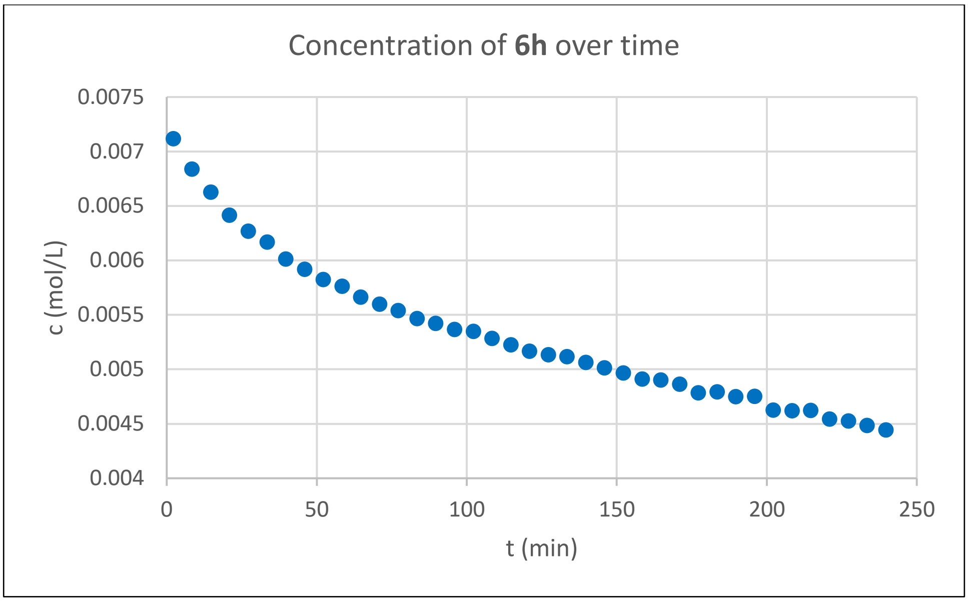
Concentration of **6h** over time
c (mol/L)
0.0075
0.007
0.0065
0.006
0.0055
0.005
0.0045
0.004
0
50
100
150
200
250
t (min)

Transmetallation of 6k and 7a

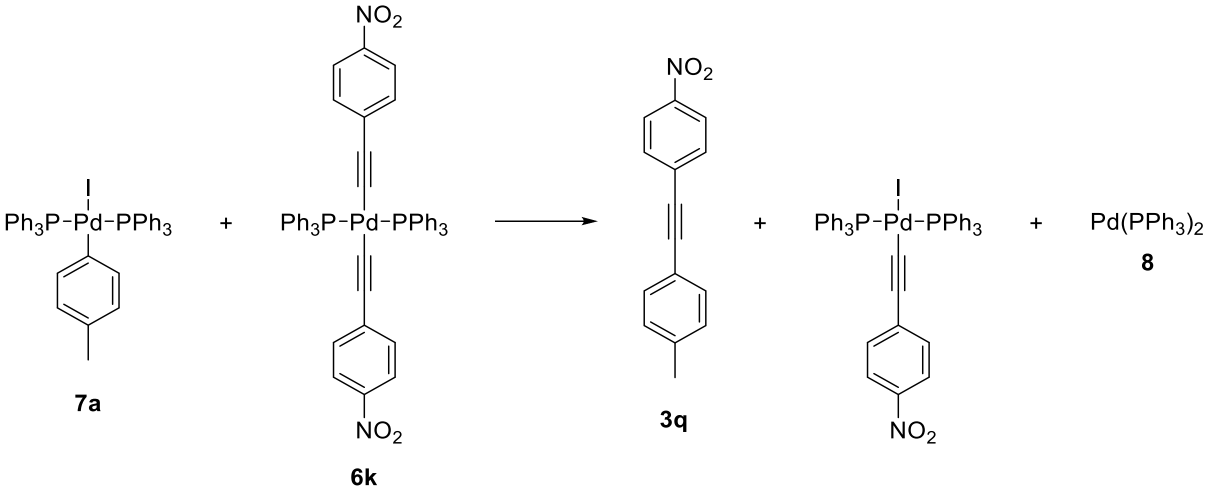

Following *GP16* using bis(triphenylphosphine)palladium(II) (4-methylphenyl)ide iodide (**7a**) (7.75 mg, 9.13 µmol), bis(triphenylphosphine)palladium(II) bis((4-nitrophenyl)ethyn-1-ide) (**6k**) (7.41 mg, 8.03 µmol), $CDCl_3$ (0.800 mL).

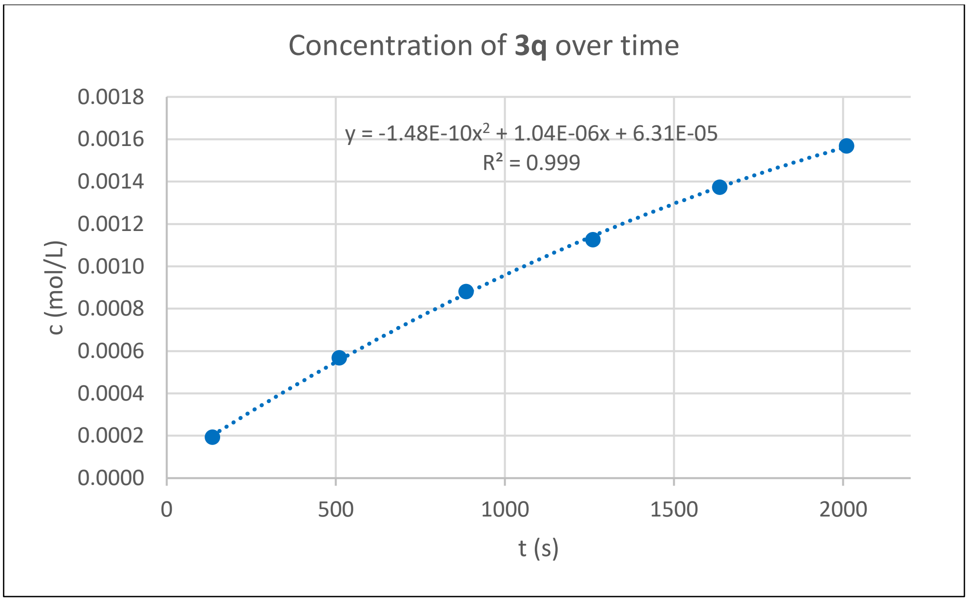

Time [s]	c [mol/L]
135	1.930E-04
510	5.682E-04
885	8.811E-04
1260	1.125E-03
1635	1.374E-03
2010	1.568E-03

$v_0 = (10.4 \pm 0.4) \times 10^{-7}$ mol/Ls

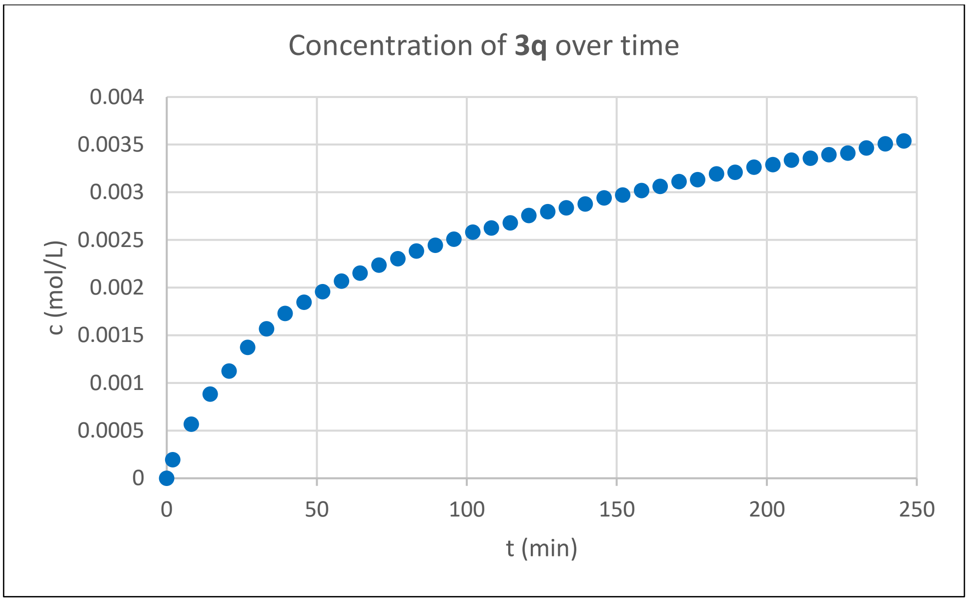

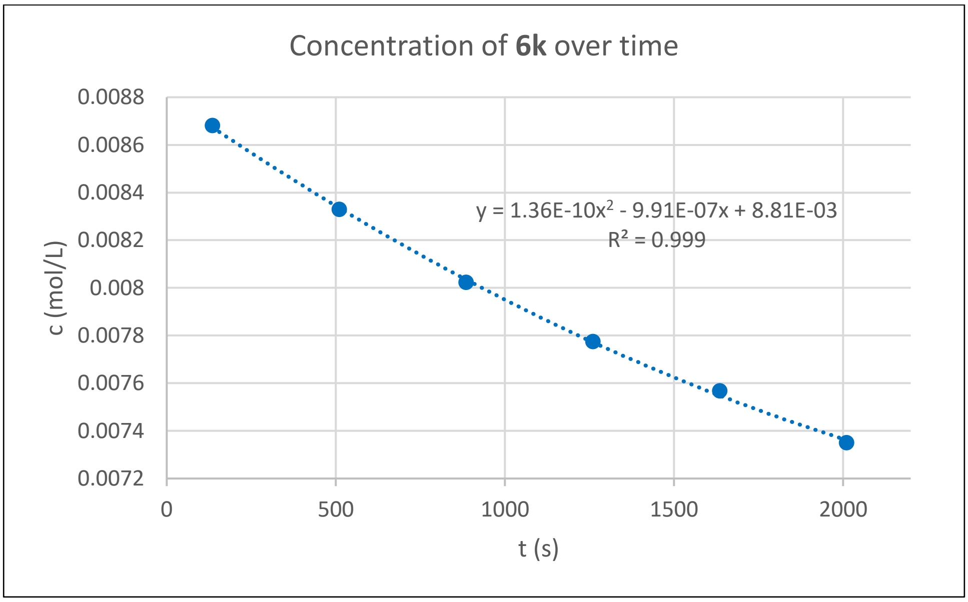

Time [s]	c [mol/L]
135	8.681E-03
510	8.329E-03
885	8.023E-03
1260	7.775E-03
1635	7.568E-03
2010	7.351E-03

$v_0 = -(9.9 \pm 0.4) \times 10^{-7}$ mol/Ls

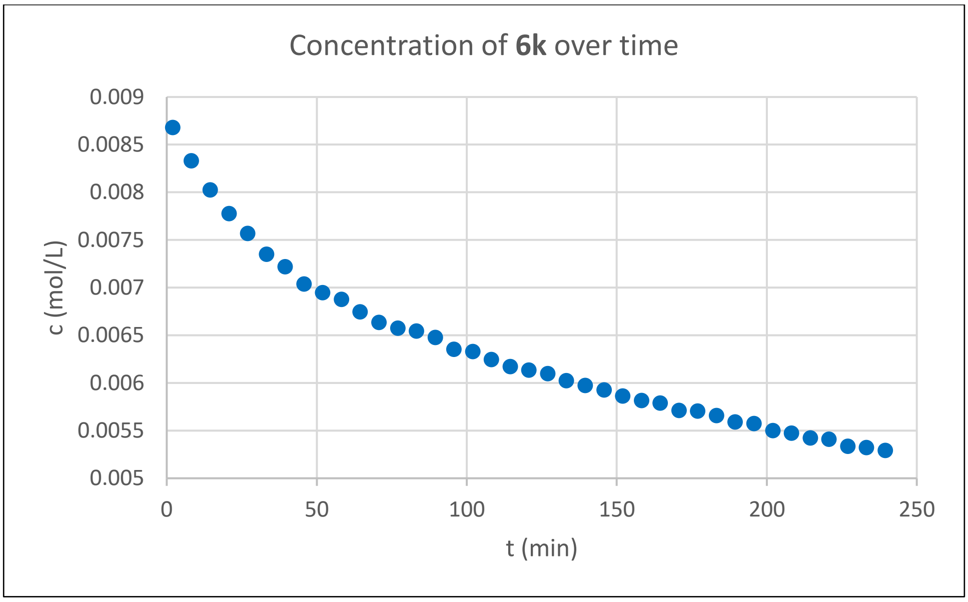
Concentration of **6k** over time
c (mol/L)
0.009
0.0085
0.008
0.0075
0.007
0.0065
0.006
0.0055
0.005
0
50
100
150
200
250
t (min)

Substituent effect of *p*-substituted bis(triphenylphosphine)palladium(II) bisacetylides 6 on transmetallation reaction using Hammett σ values (Figure 8b)

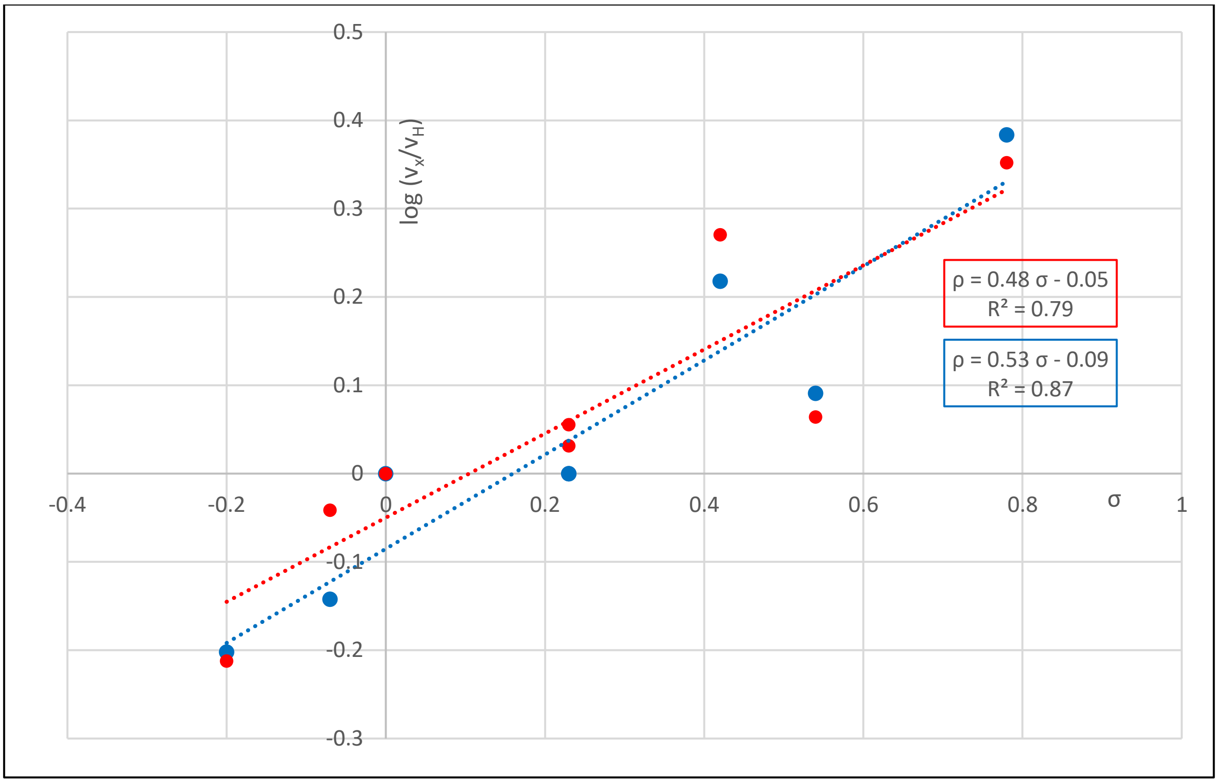

Figure 8b. Hammett plot for product **3** formation and palladium bisacetylide conversion **6** for transmetallation reaction between **7** and **6** using Hammett σ values.

Bis(triphenylphosphine)palladium bisacetylide (6) conversion Hammett correlation

Substituent	σ	v (mol/Ls)	log (v_x/v_H)
tBu	-0.20	-2.7E-07	-0.2121
$SiMe_3$	-0.07	-4.0E-07	-0.0414
H	0.00	-4.4E-07	0.0000
Cl	0.23	-5.0E-07	0.0555
Br	0.23	-4.73E-07	0.0314
CHO	0.42	-8.2E-07	0.2704
CF_3	0.54	-5.1E-07	0.0641
NO_2	0.78	-9.9E-07	0.3522

Product (3) formation Hammett correlation

Substituent	σ	v (mol/Ls)	log (v_x/v_H)
tBu	-0.20	2.7E-07	-0.2021
$SiMe_3$	-0.07	3.1E-07	-0.1421
H	0.00	4.3E-07	0.0000
Cl	0.23	4.30E-07	0.0000
Br	0.23	4.3E-07	0.0000
CHO	0.42	7.1E-07	0.2178
CF_3	0.54	5.3E-07	0.0908
NO_2	0.78	1.04E-06	0.3836

The σ-values were taken from:

Hansch, C., Leo, A. & Taft, R. W. A Survey of Hammett Substituent Constants and Resonance and Field Parameters. *Chem. Rev.* **91**, 165–195 (1991).

Substituent effect of *p*-substituted bis(triphenylphosphine)palladium(II) bisacetylides 6 on transmetallation reaction using corrected Hammett σ values

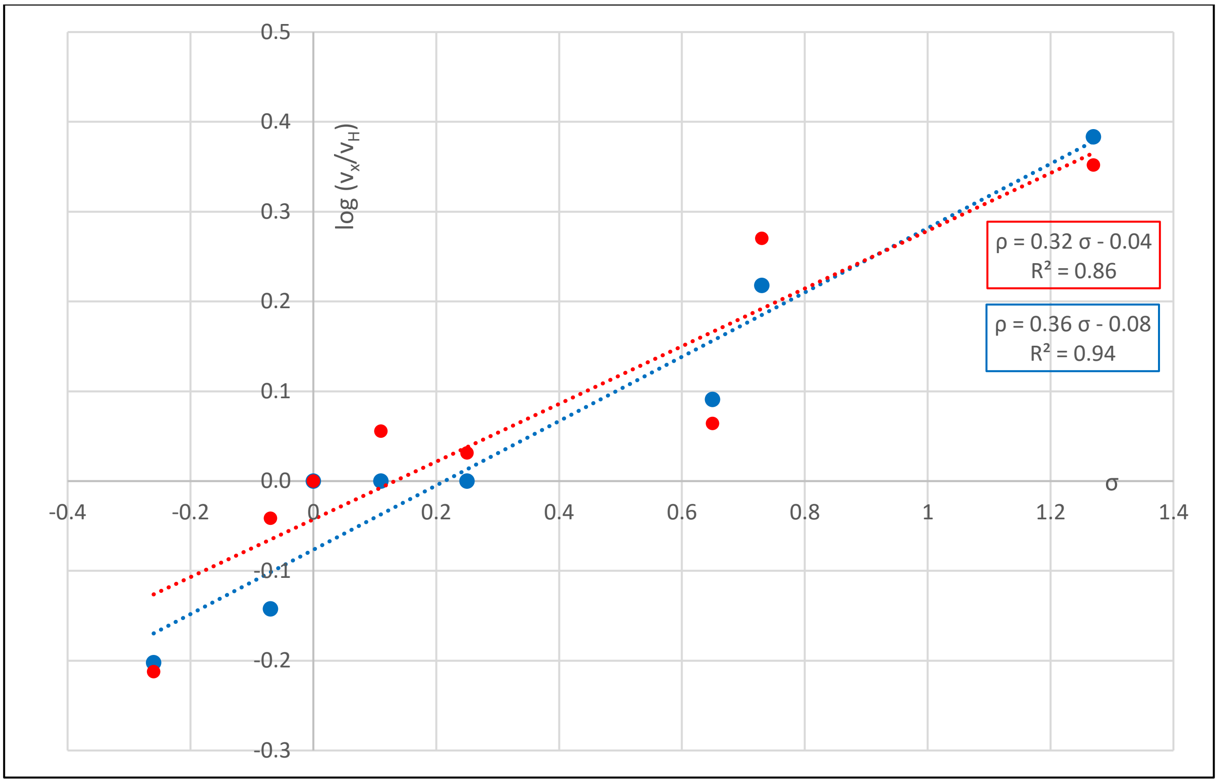

Supplementary Figure 19. Hammett plot for product **3** formation and palladium bisacetylide conversion **6** for transmetallation reaction between **7** and **6** using corrected Hammett σ values.

Bis(triphenylphosphine)palladium bisacetylide (6) conversion rate Hammett correlation

Substituent	σ	v (mol/Ls)	log (v_x/v_H)
tBu	-0.26	-2.7E-07	-0.2121
$SiMe_3$	-0.07	-4.0E-07	-0.0414
H	0.00	-4.4E-07	0.0000
Cl	0.11	-5.0E-07	0.0555
Br	0.25	-4.73E-07	0.0314
CHO	0.73	-8.2E-07	0.2704
CF_3	0.65	-5.1E-07	0.0641
NO_2	1.27	-9.9E-07	0.3522

Product (3) formation Hammett correlation

Substituent	σ	v (mol/Ls)	log (v_x/v_H)
tBu	-0.26	2.7E-07	-0.2021
$SiMe_3$	-0.07	3.1E-07	-0.1421
H	0.00	4.3E-07	0.0000
Cl	0.11	4.30E-07	0.0000
Br	0.25	4.3E-07	0.0000
CHO	0.73	7.1E-07	0.2178
CF_3	0.65	5.3E-07	0.0908
NO_2	1.27	1.04E-06	0.3836

The σ-values were taken from:

Hansch, C., Leo, A. & Taft, R. W. A Survey of Hammett Substituent Constants and Resonance and Field Parameters. *Chem. Rev.* **91**, 165–195 (1991).

Studies of competitive transmetallation reactions

Transmetallation reactions were carried out in a sealed NMR tube at 298.0 K. The conversion to the corresponding product **3** was estimated by ^{1}H NMR by comparing the integrals of the resonances of the starting compounds **7** and **6** with the integrals of the characteristic resonances of the corresponding product **3**.

Competitive transmetallation reactions of Pd oxidative addition complexes 7

The initial rates of reaction for complexes 7a and 7c

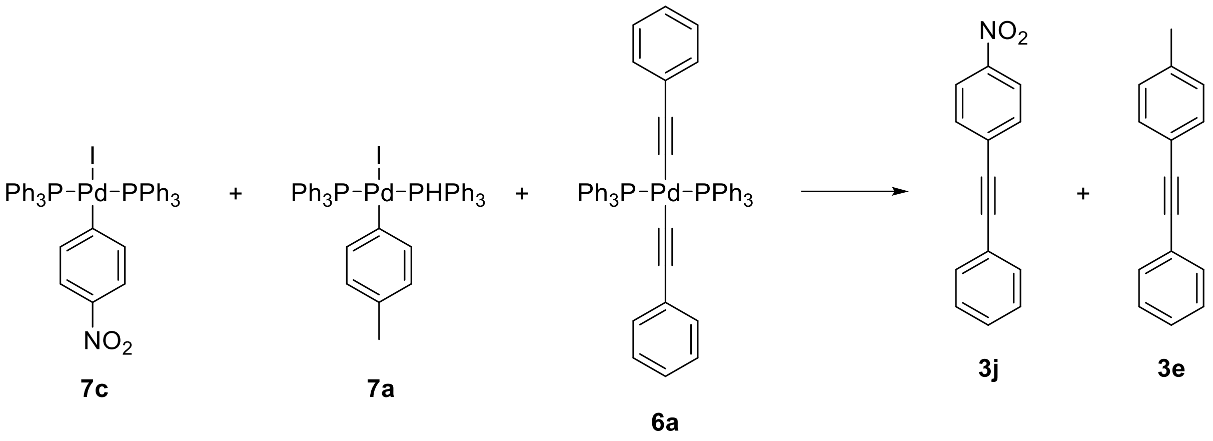

In a vial a solution of bis(triphenylphosphine)palladium bis(phenylethynide) (**6a**) (6.70 mg, 8.04 µmol) in degassed $CDCl_3$ (0.800 mL) was prepared under argon atmosphere. Bis(triphenylphosphine)palladium(II) (4-nitrophenyl)ide iodide (**7c**) (3.46 mg, 3.93 µmol) and bis(triphenylphosphine)palladium(II) (4-methylphenyl)ide iodide (**7a**) (3.44 mg, 4.05 µmol) were added to this solution in one portion. The reaction mixture was sonicated for 0.5 min and transferred into NMR tube, flushed with argon and sealed. NMR spectra were acquired in intervals (156 s).

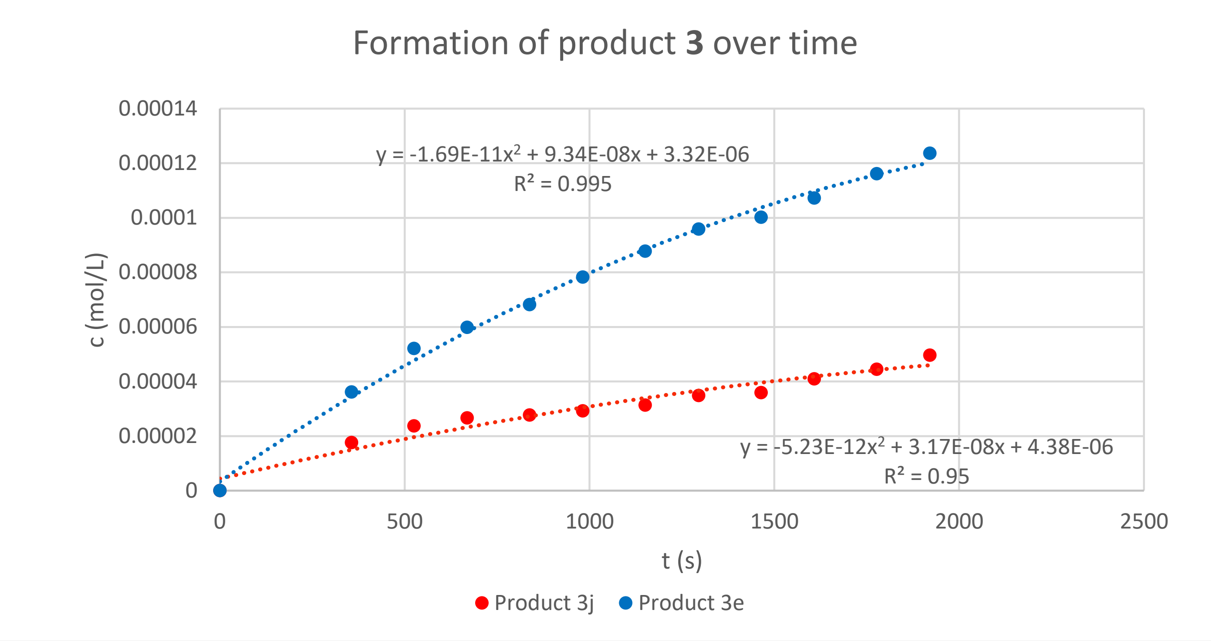

Supplementary Figure 20. Comparison of initial rates of competitive reaction between complexes **7a** and **7c**.

v_0 (**3e**) = 9.34 × 10^{-8} mol/Ls

v_0 (**3j**) = 3.17 × 10^{-8} mol/Ls

v_0 (**3e**) : v_0 (**3j**) = 2.946

The ratio is consistent with Hammett plot results (v_0 (**3e**) : v_0 (**3j**) = 2.714).

Reaction with limiting reactant 6a

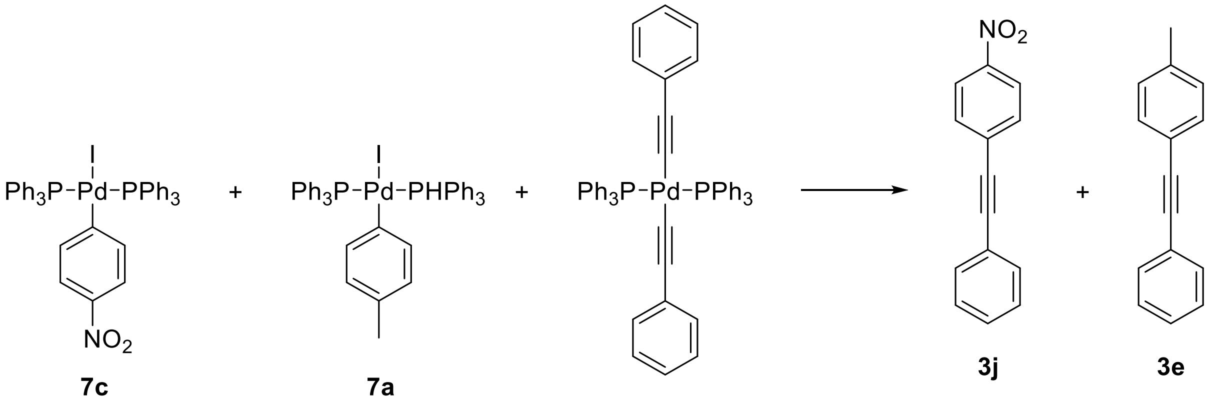

In a vial a solution of bis(triphenylphosphine)palladium bis(phenylethynide) (**6a**) (0.68 mg, 0.82 µmol) in degassed $CDCl_3$ (0.800 mL) was prepared under argon atmosphere. Bis(triphenylphosphine)palladium(II) (4-nitrophenyl)ide iodide (**7c**) (3.61 mg, 4.10 µmol) and bis(triphenylphosphine)palladium(II) (4-methylphenyl)ide iodide (**7a**) (3.44 mg, 4.05 µmol)

were added to this solution in one portion. The reaction mixture was sonicated for 0.5 min and transferred into NMR tube, flushed with argon and sealed. NMR spectra were acquired after 1.5 h.

c (**3e**) = 0.0905 mM
c (**3j**) = 0.0333 mM

c (**3e**) : c (**3j**) = 2.718

The ratio is consistent with Hammett plot results (v_0 (**3e**) : v_0 (**3j**) = 2.714).

Competitive transmetallation reactions of palladium bisacetylides 6

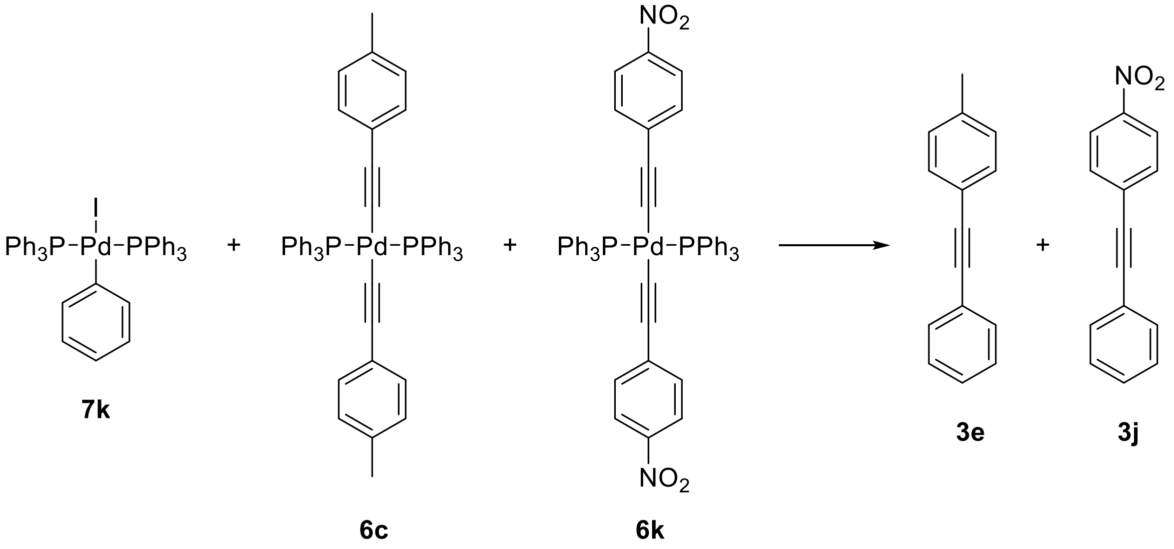

In a vial a solution of bis(triphenylphosphine)palladium(II) phenylide iodide (**7k**) (6.69 mg, 8.01 μmol) in degassed $CDCl_3$ (0.800 mL) was prepared under argon atmosphere. Bis(triphenylphosphine)palladium(II) bis((4-methylphenyl)ethyn-1-ide) (**6c**) (3.51 mg, 4.08 μmol) and bis(triphenylphosphine)palladium(II) bis((4-nitrophenyl)ethyn-1-ide) (**6k**) (3.72 mg, 4.03 μmol) were added to this solution in one portion. The reaction mixture was sonicated for 0.5 min and transferred into NMR tube, flushed with argon and sealed. NMR spectra were acquired in intervals (144 s).

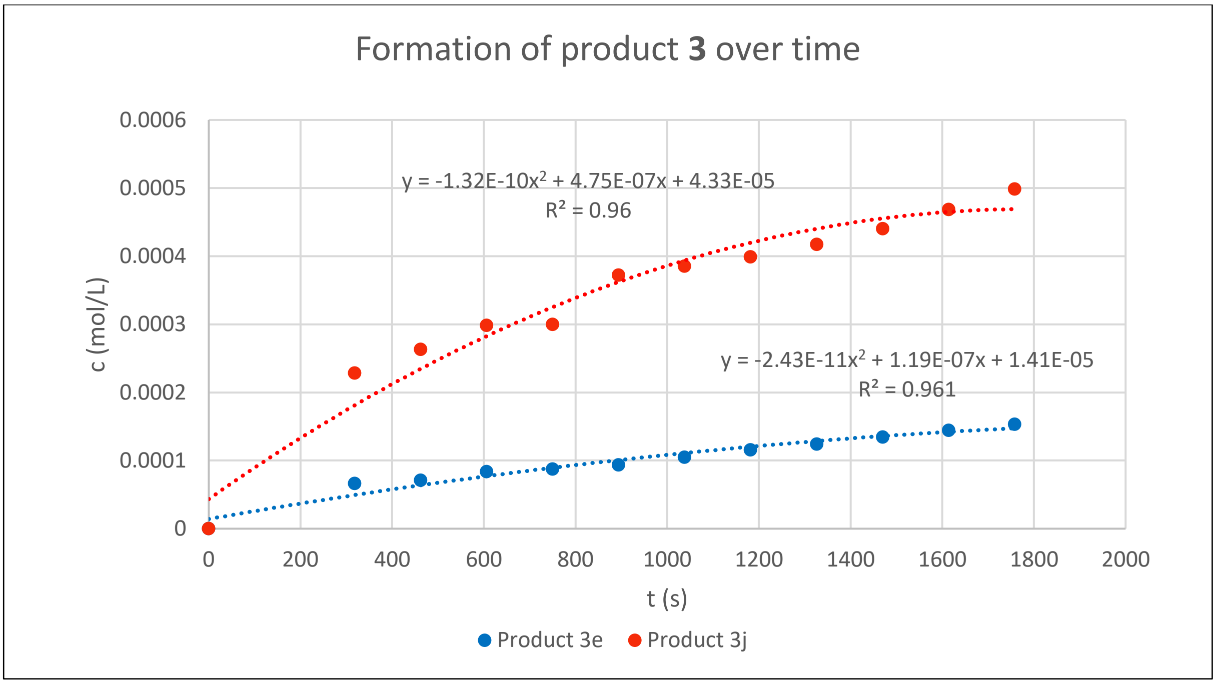

Supplementary Figure 21. Comparison of initial rates of competitive reaction between complexes **6c** and **6k**.

v_0 (**3e**) = 1.19 × 10^{-7} mol/Ls

v_0 (**3j**) = 4.75 × 10^{-7} mol/Ls

v_0 (**3j**) : v_0 (**3e**) = 3.992

The ratio is consistent with Hammett plot results* (v_0 (**3j**) : v_0 (**3e**) = 3.900).

***Transmetallation of 6c and 7a**

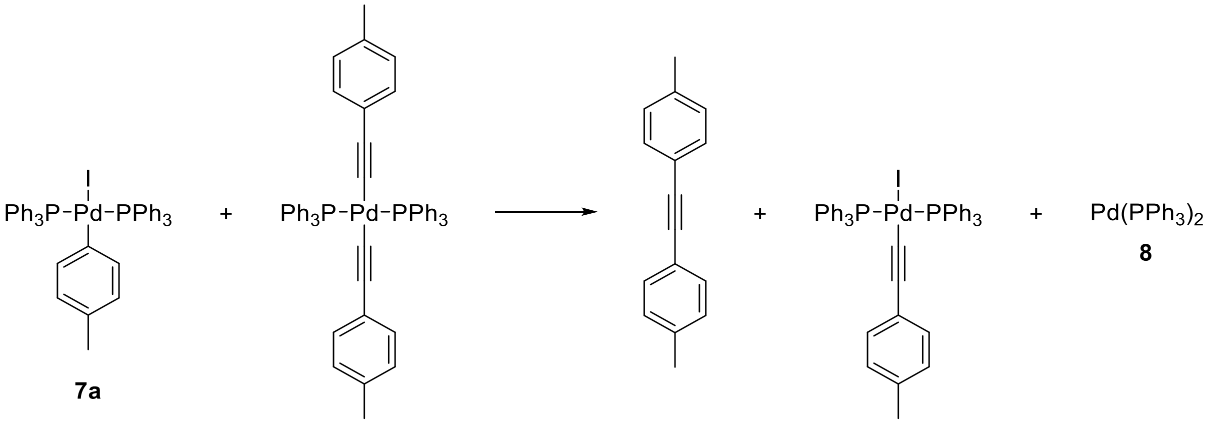

Following *GP16* using bis(triphenylphosphine)palladium(II) (4-methylphenyl)ide iodide (**7a**) (6.77 mg, 7.97 µmol), bis(triphenylphosphine)palladium(II) bis((4-methylphenyl)ethyn-1-ide) (**6c**) (6.92 mg, 8.03 µmol), $CDCl_3$ (0.800 mL).

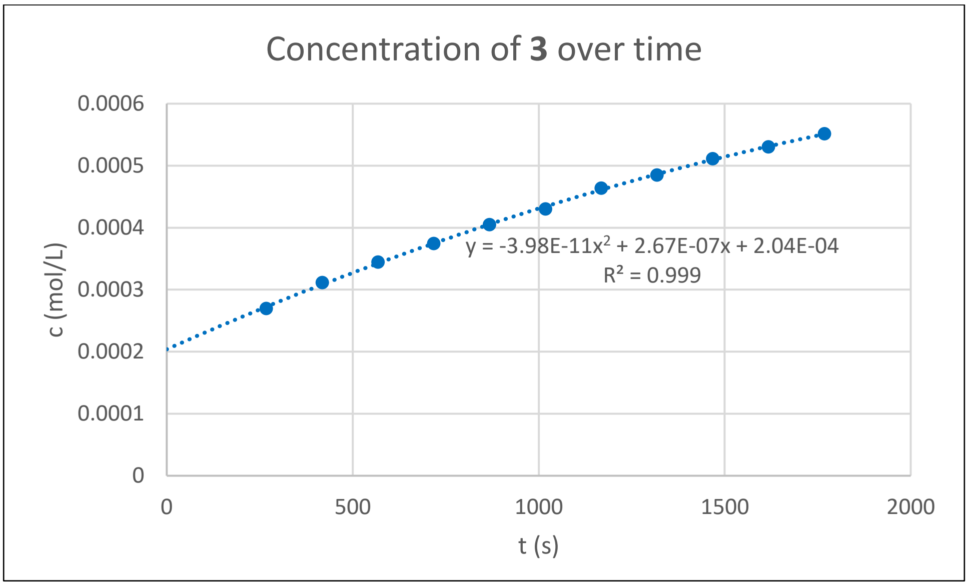

$v_0 = (2.67 \pm 0.08) \times 10^{-7}$ mol/Ls

Reaction with 7k as the limiting reagent

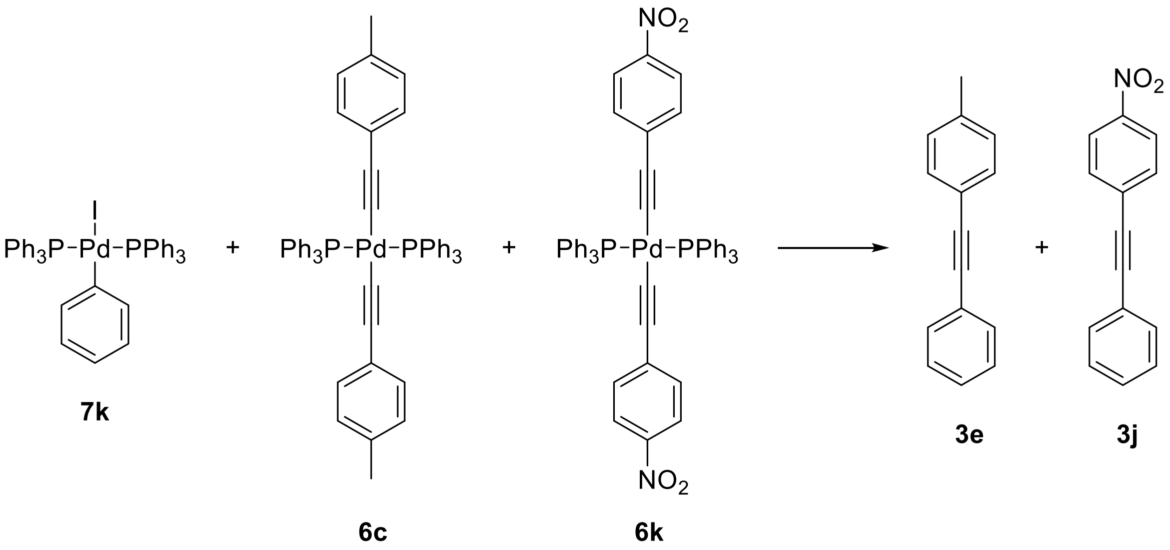

In a vial a solution of bis(triphenylphosphine)palladium(II) phenylide iodide (**7k**) (0.73 mg, 0.87 µmol) in degassed $CDCl_3$ (0.800 mL) was prepared under argon atmosphere. Bis(triphenylphosphine)palladium(II) bis((4-methylphenyl)ethyn-1-ide) (**6c**) (3.48 mg, 4.04 µmol) and bis(triphenylphosphine)palladium(II) bis((4-nitrophenyl)ethyn-1-ide) (**6k**) (3.78 mg, 4.09 µmol) were added to this solution in one portion. The reaction mixture was sonicated for 0.5 min and transferred into NMR tube, flushed with argon and sealed. NMR spectra were acquired after 2 h.

c (**3e**) = 0.0432 mM
c (**3j**) = 0.176 mM

c (**3j**) : c (**3e**) = 4.074

The ratio is consistent with Hammett plot results* (v_0 (**3j**) : v_0 (**3e**) = 3.900).

2.1.5. Kinetic studies of the elementary steps of the mechanism

Determining the rate-determining step of the catalytic reaction of 1a and 2a by comparing the rates of elementary steps of the proposed mechanism (Table 1).

Investigated reactions and their rates:

(a)

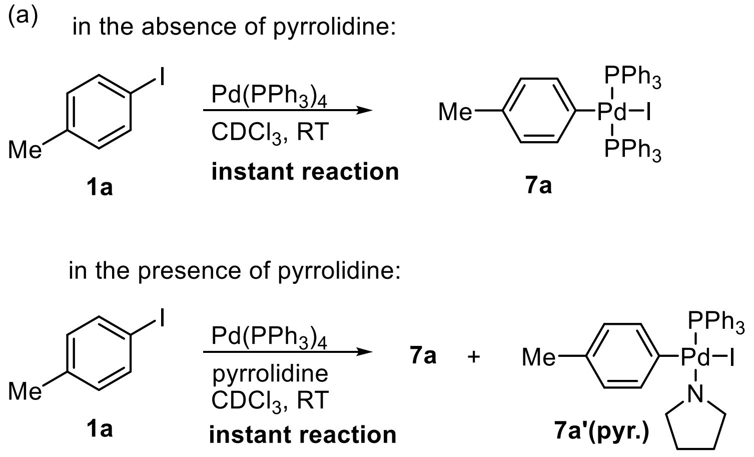

(b)

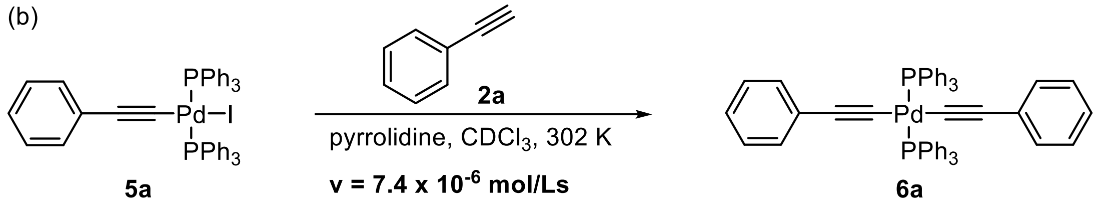

(c)

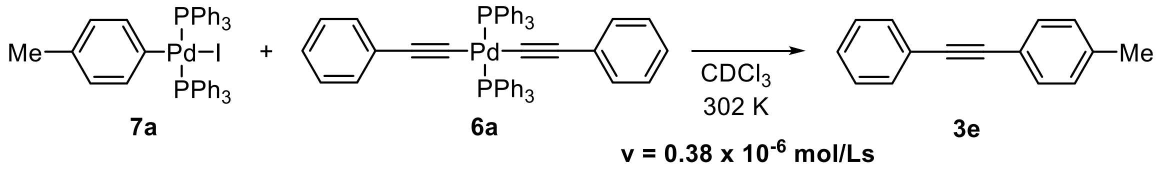

(d)

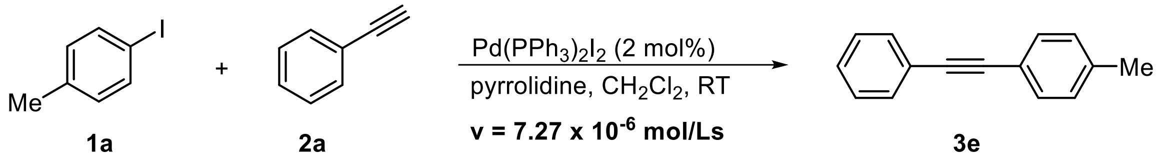

(e)

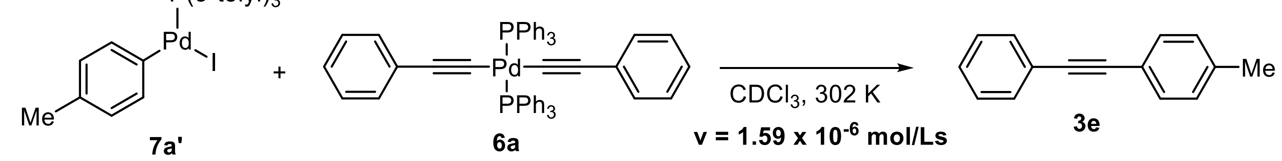

Spectra of Pd(PPh$_3$)$_4$ in different solvents

Palladium(0) tetrakis(triphenylphosphine) (11.5 mg, 0.01 mmol) was dissolved in $CDCl_3$ (1 mL) in a nitrogen-filled glovebox. The solution was transferred to a dry NMR tube. The $^{31}P\{^1H\}$ NMR spectra (given below) of solutions were recorded after 10 min.

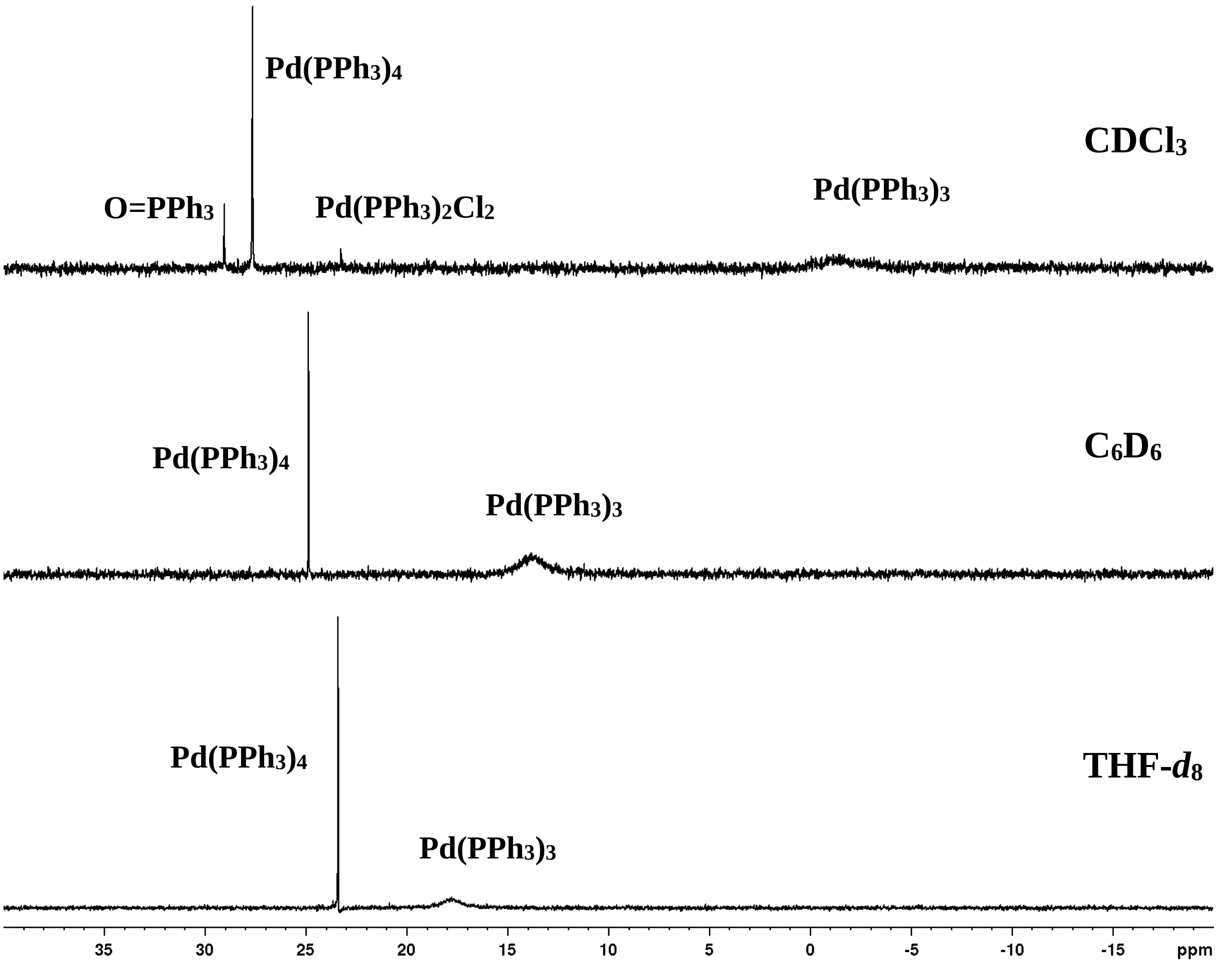

Stack of $^{31}P\{^1H\}$ NMR spectra of Pd(PPh$_3$)$_4$ in different NMR solvents.

Decomposition of $Pd(PPh_3)_4$ in $CDCl_3$

Palladium(0) tetrakis(triphenylphosphine) (11.5 mg, 0.01 mmol) was dissolved in $CDCl_3$ (1 mL) in a nitrogen-filled glovebox. The solution was transferred to a dry NMR tube. The $^{31}P\{^1H\}$ NMR spectrum (given below) of the solution was recorded after 10 min, 2 h and 20 h.

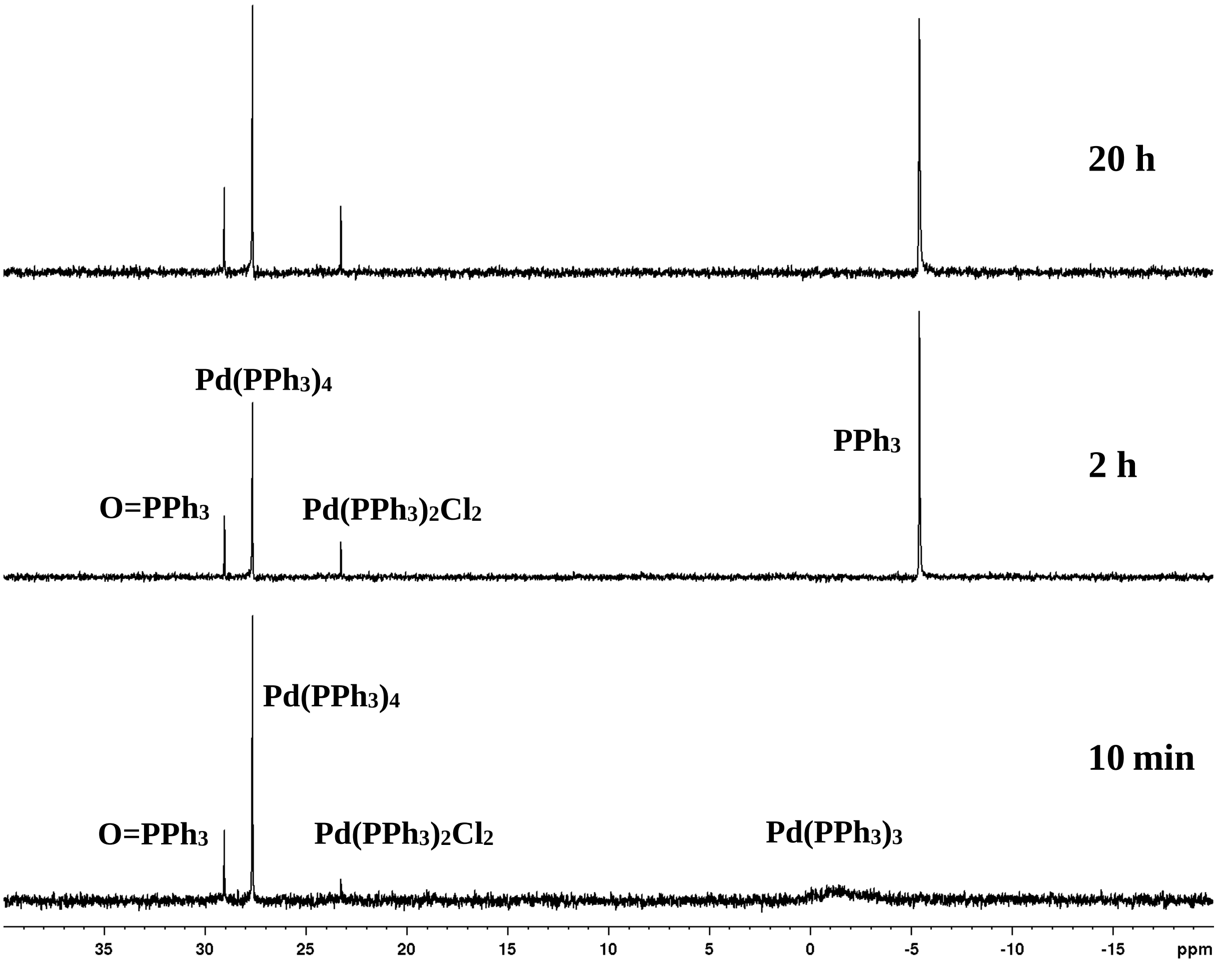

Stack of $^{31}P\{^1H\}$ NMR spectra of $Pd(PPh_3)_4$ in $CDCl_3$ over time. Although decomposition into $Pd(PPh_3)_2Cl_2$ can be observed, it occurs slowly - about 5% $Pd(PPh_3)_2Cl_2$ was observed after 10 min, 11% after 2 h, and 13% after 20 h.

Formation of bis(triphenylphosphine)palladium(II) (4-methylphenyl)ide iodide (7a)

Reaction was performed at room temperature (296 K) in nitrogen-filled glovebox. Solvent ($CDCl_3$) was dried over CaH_2 and distilled before use. Palladium tetrakis(triphenylphosphine) (23.2 mg, 0.02 mmol) was dissolved in $CDCl_3$ (2.00 mL). Part of the solution (1.00 mL) was transferred to vial containing 4-iodotoluene (**1a**) (22.3 mg, 0.102 mmol), mixed for 0.5 min and transferred to dry NMR tube, respectively. Sample was recorded after 10 min and 30 min.

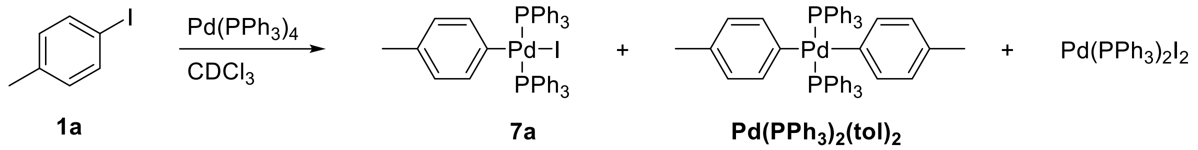

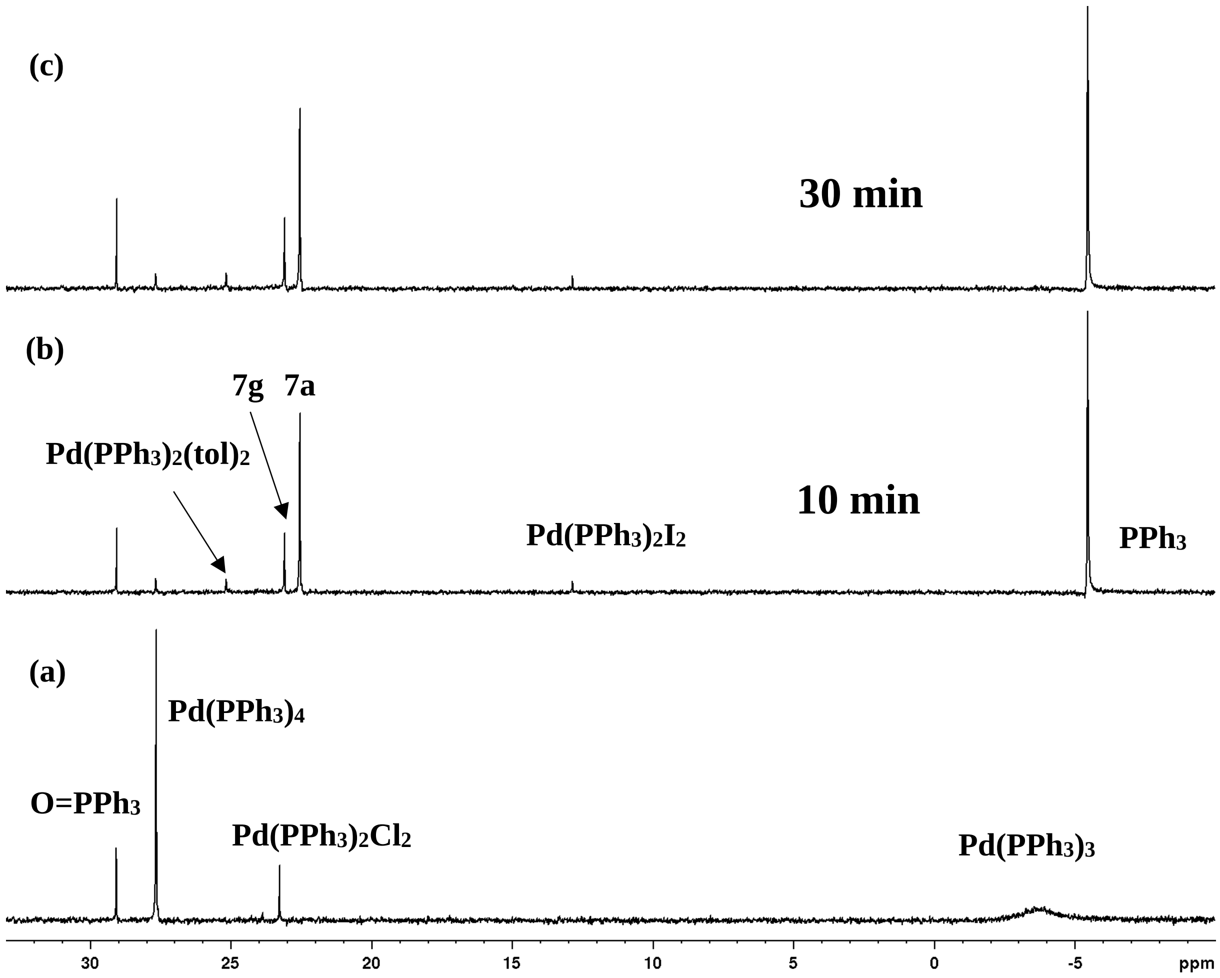

Supplementary Figure 22. Stack of $^{31}P\{^1H\}$ NMR spectra of (a) $Pd(PPh_3)_4$ from commercial source in $CDCl_3$, (b) reaction of $Pd(PPh_3)_4$ with **1a** after 10 minutes and (c) 30 minutes.

Equilibrium (reaction) of $Pd(PPh_3)_4$ with **1a** is formed (completed) within 10 minutes.

Due to reactivity of **7a**, dissociated PPh_3 and presence of $Pd(PPh_3)_2Cl_2$ in starting material, other species can be observed:

(a) oxidation of PPh_3

$PPh_3 \xrightarrow{O_2} O{=}PPh_3$ $^{31}P\{1H\}$ NMR δ +29.7 ppm

(b) transmetallation between two complexes **7a**

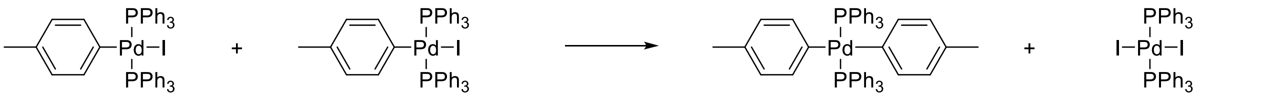

7a $^{31}P\{^1H\}$ NMR δ +22.5 ppm

7a

$Pd(PPh_3)_2(tol)_2$ $^{31}P\{^1H\}$ NMR δ +25.2 ppm

$Pd(PPh_3)_2I_2$ $^{31}P\{^1H\}$ NMR δ +12.9 ppm

(c) transmetallation between **7a** and $Pd(PPh_3)_2Cl_2$

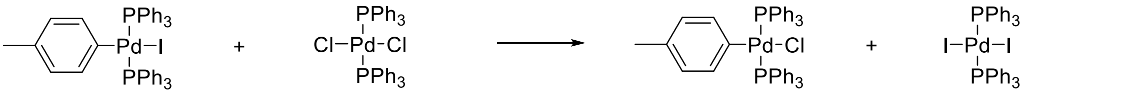

7a $^{31}P\{^1H\}$ NMR +22.5 ppm

$Pd(PPh_3)_2Cl_2$ $^{31}P\{^1H\}$ NMR +23.3 ppm

7g $^{31}P\{^1H\}$ NMR +23.1 ppm

$Pd(PPh_3)_2I_2$ $^{31}P\{^1H\}$ NMR +12.9 ppm

Oxidative addition in presence of pyrrolidine

Reaction was performed at room temperature (296 K) in nitrogen-filled glovebox. Solvent ($CDCl_3$) was dried over CaH_2 and distilled before use. Palladium tetrakis(triphenylphosphine) (23.5 mg, 0.02 mmol) was dissolved in $CDCl_3$ (2.00 mL). Part of the solution (1.00 mL) was transferred to vial containing 4-iodotoluene (**1a**) (22.8 mg, 0.105 mmol) and pyrrolidine (8.28 μL, 0.100 mmol), mixed for 0.5 min and transferred to dry NMR tube, respectively. Sample was recorded after 10 min and 30 min.

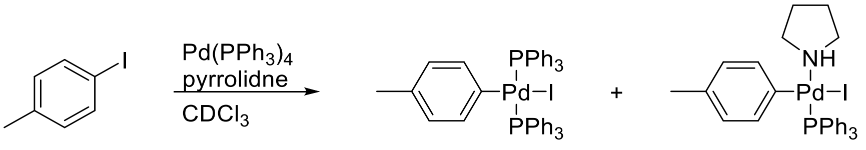

1a **7a** **7a'(pyr.)**

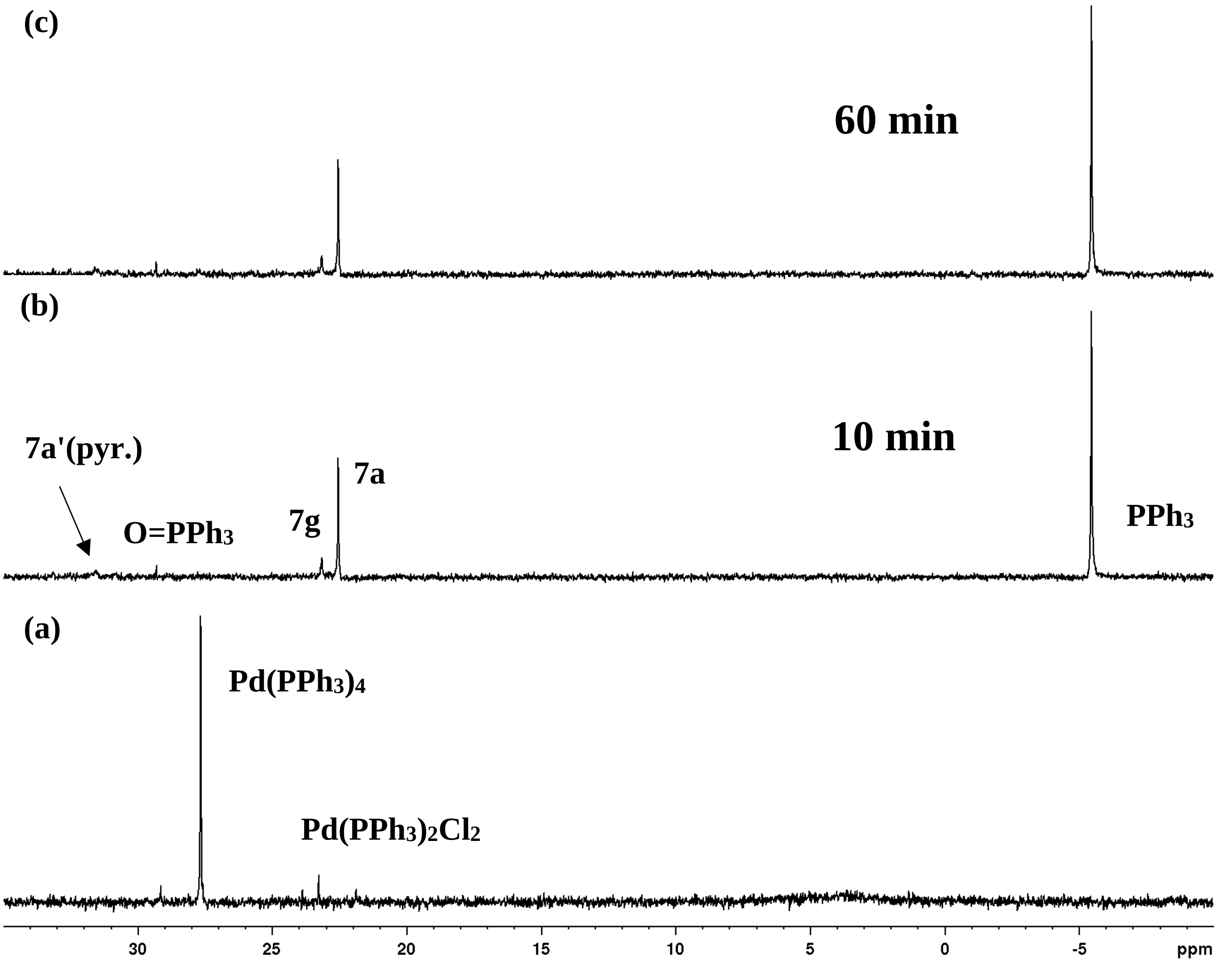

Supplementary Figure 23. Stack of $^{31}P\{^{1}H\}$ NMR spectra of (a) $Pd(PPh_3)_4$ from commercial source in $CDCl_3$, (b) reaction of $Pd(PPh_3)_4$ with **1a** in presence of pyrrolidine after 10 minutes and (c) 60 minutes.

Equilibrium (reaction) of $Pd(PPh_3)_4$ with **1a** in $CDCl_3$/pyrrolidine is formed (completed) within 10 minutes.

Effect of tetrabutylammonium chloride and pyrrolidine on oxidative addition of 4-iodotoluene (1a) to tetrakis(triphenylphosphine)palladium(0)

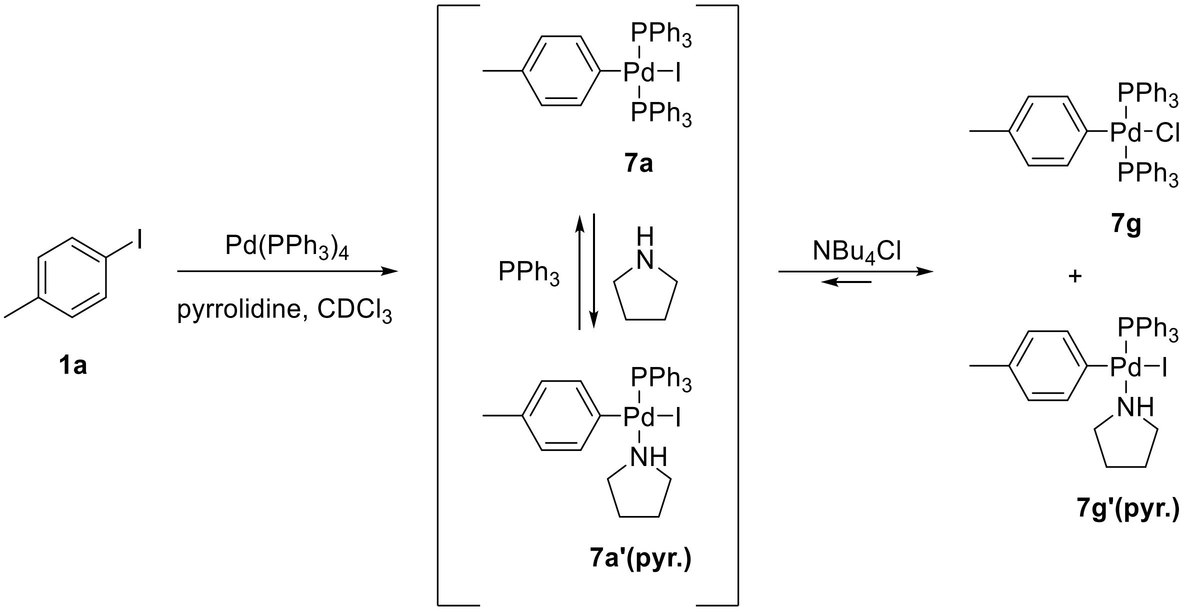

Reaction was performed at room temperature (296 K) in a nitrogen-filled glovebox. In a vial a solution of 4-iodotoluene (**1a**) (17.9 mg, 0.082 mmol) in degassed $CDCl_3$ (0.800 mL) was prepared under argon atmosphere. To this solution, degassed pyrrolidine (6.75 µL, 0.082 mmol) and tetrakis(triphenylphosphine)palladium(0) (9.8 mg, 0.0085 mmol) were added, respectively. Mixture was transferred to a dry NMR tube, sealed and taken out of glovebox. After $^{31}P\{^1H\}$ NMR spectrum was obtained (10 min), tetrabutylammonium chloride (11.3 mg, 0.041 mmol) was added to the reaction mixture in one portion. The reaction mixture was transferred back to the NMR tube, purged with argon and sealed. Reaction was monitored for 1 hour using $^{31}P\{^1H\}$ NMR.

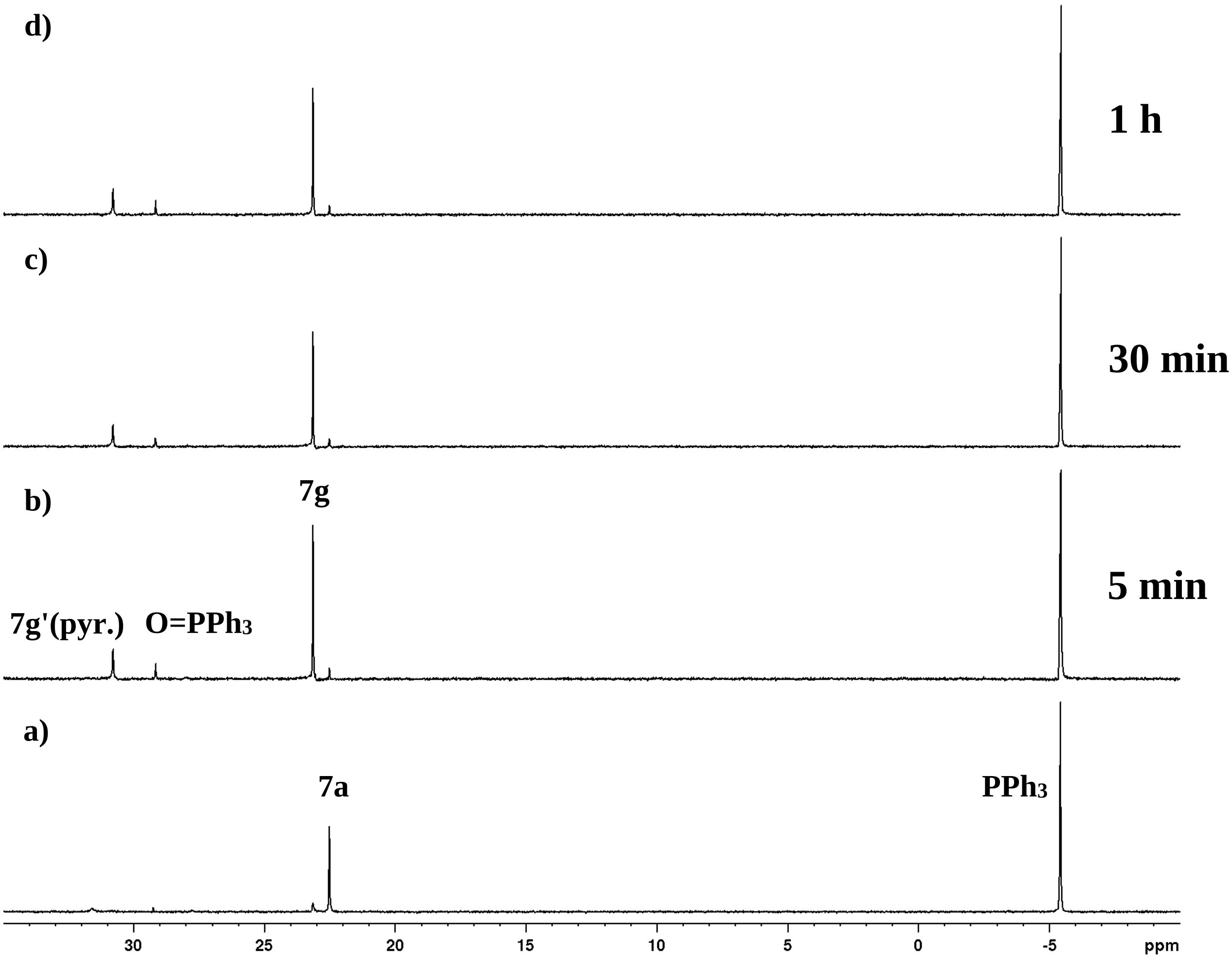

Supplementary Figure 24. Stack of $^{31}P\{^{1}H\}$ NMR spectra of a) oxidative addition of **1a** to $Pd(PPh_3)_4$ in $CDCl_3$ in presence of pyrrolidine and reaction mixture after addition of NBu_4Cl after b) 5 min, c) 30 min and d) 1 h.

Oxidative addition was complete within 5 min after addition of $Pd(PPh_3)_4$ to solution of **1a** in $CDCl_3$ and pyrrolidine. Equilibrium was completed within 5 mins after addition of NBu_4Cl.

Formation of bis(triphenylphosphine)palladium(II) (4-acetylphenyl)ide iodide (7q)

The reaction was carried out at room temperature (296 K) in nitrogen-filled glovebox. Solvent ($CDCl_3$) was dried over P_2O_5 and distilled before use. 1-(4-Iodophenyl)ethan-1-one (75 mg, 0.305 mmol) was dissolved in $CDCl_3$ (1.0 mL) in a vial, and palladium tetrakis(triphenylphosphine) (20.5 mg, 0.018 mmol) was added to this solution. The solution was mixed and transferred to a dry NMR tube. The $^{31}P\{^{1}H\}$ NMR spectrum (given below) of the homogeneous reaction mixture was recorded after 10 min and 30 min.

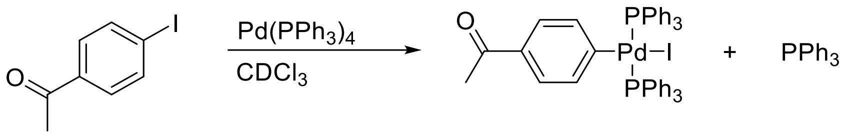

1m **7q**

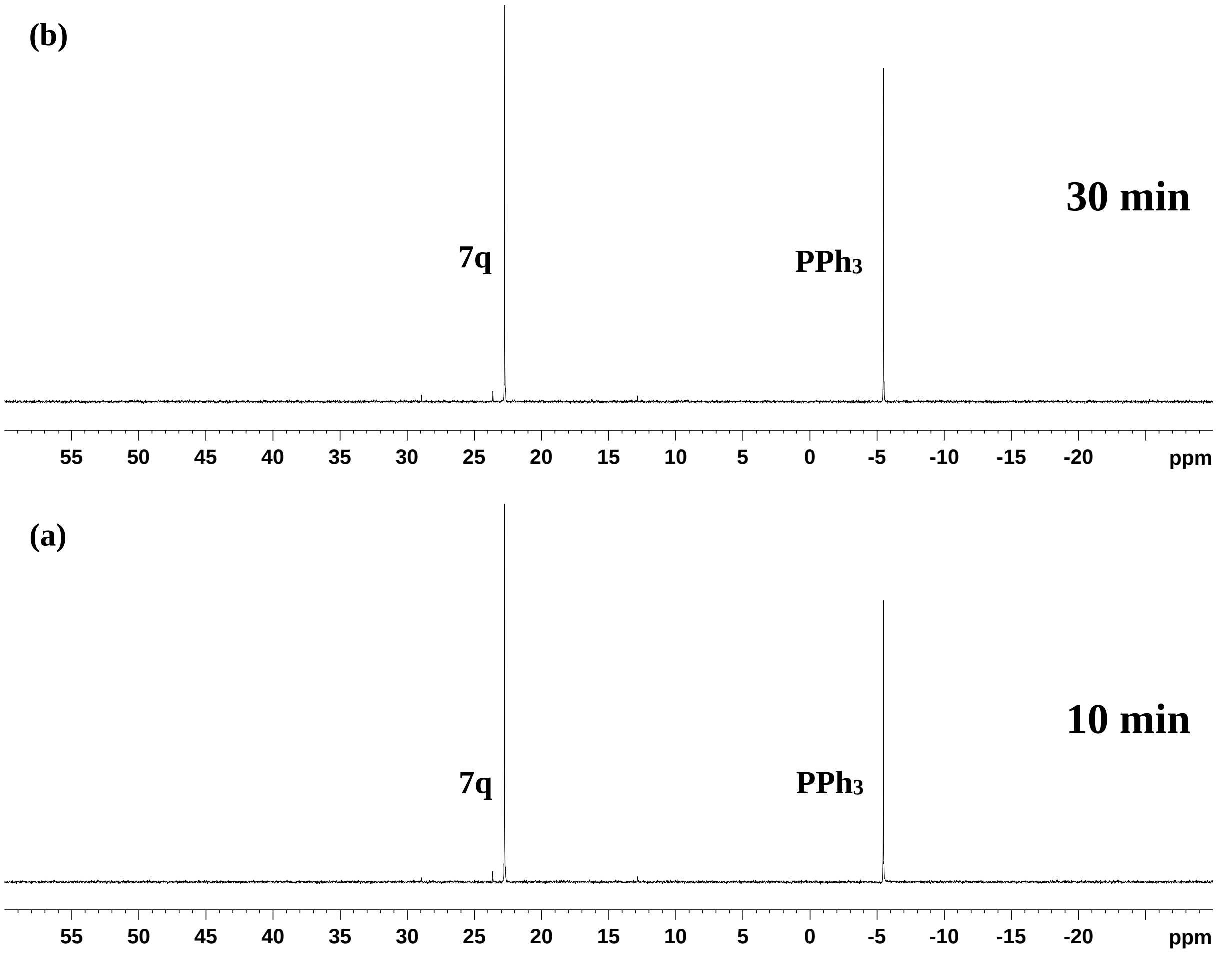

Stack of $^{31}P\{^1H\}$ NMR spectra of (a) reaction of $Pd(PPh_3)_4$ with **1m** after 10 minutes and (b) 30 minutes.

The equilibrium between **1m** and $Pd(PPh_3)_4$ forms within 10 minutes and does not change thereafter, indicating rapid formation of **7q**. In contrast to the oxidative addition of 4-iodotoluene, no additional phosphorus resonances were observed in the $^{31}P\{^1H\}$ NMR spectra in this case. The substituents on the phenyl ring of the aryl iodide affect the formation of the palladium oxidative addition complex as well as its stability. In the case of a palladium oxidative addition adduct containing an electron-rich phenyl ring with a methyl substituent and electron-donating character, an intermolecular transmetallation reaction occurred between two palladium complexes (see Supplementary Fig. 22), whereas no such species was observed in the case of 4-iodoacetophenone with a substituent of electron-withdrawing character.

Effect of excess PPh_3 on transmetallation of 6a and 7a

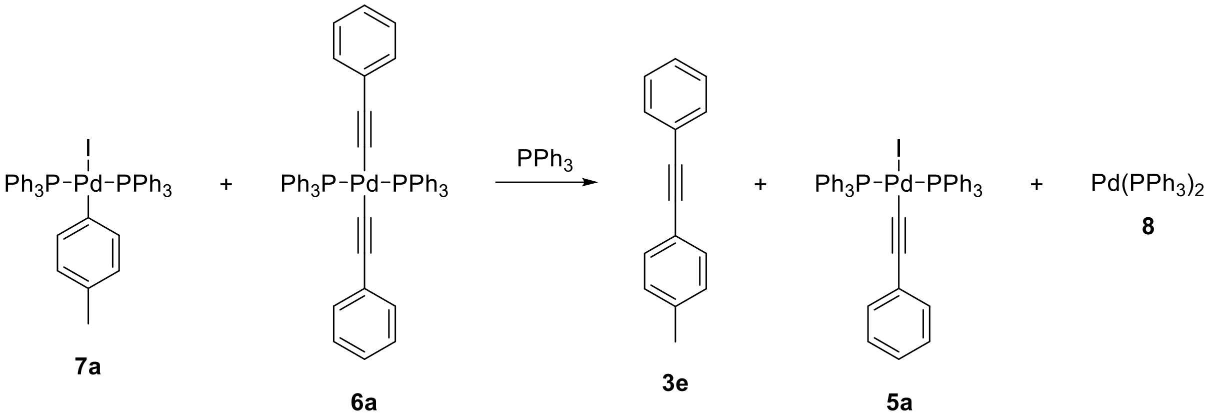

In a vial a solution of bis(triphenylphosphine)palladium(II) bis(phenylethynide) (**6a**) (6.73 mg, 8.08 μmol) in $CDCl_3$ (0.800 mL) was prepared under argon atmosphere. Bis(triphenylphosphine)palladium (4-methylphenyl)ide iodide (**7a**) (6.70 mg, 7.89 μmol) and triphenylphosphine (9.09 mg, 34.7 μmol) were added to this solution in one portion. The reaction mixture was sonicated for 0.5 min and transferred to NMR tube, flushed with argon and sealed. NMR spectra were acquired at 302.0 K in intervals (147 s). The concentration of the product **3e** was determined by comparison of integrals of characteristic resonances of product **3e** with integrals of resonances of an internal standard (1,3,5-trimethoxybenzene).

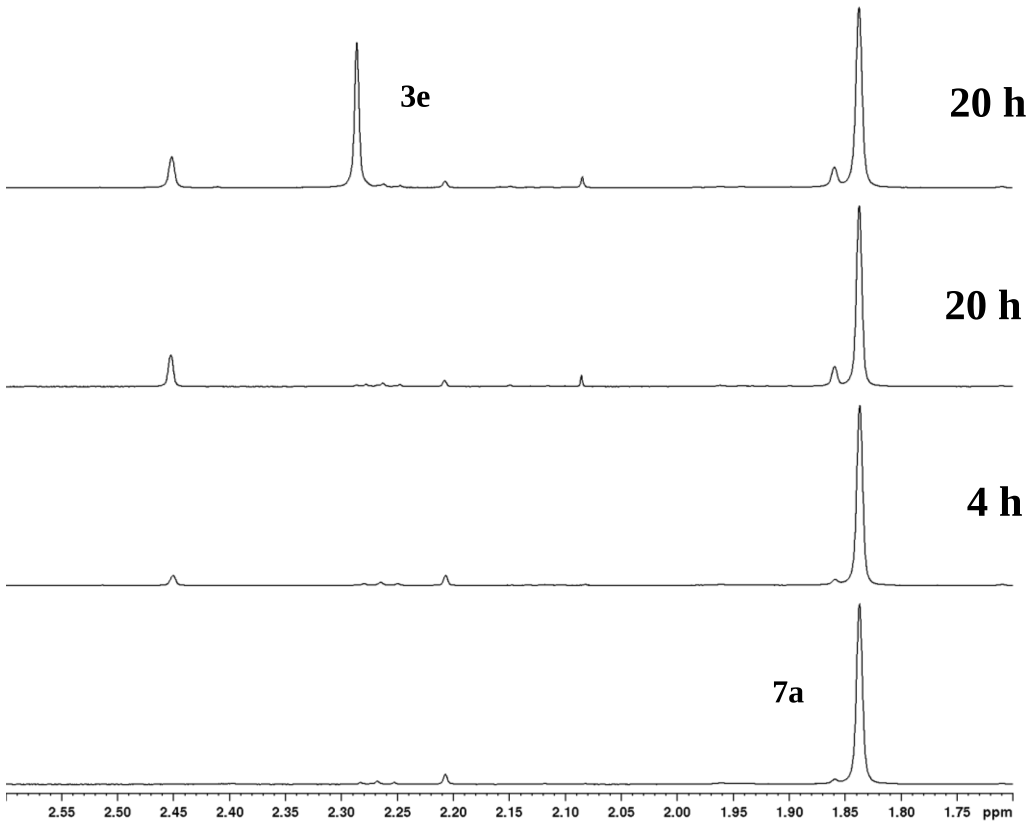

Supplementary Figure 25. Stack of ^{1}H NMR spectra of the characteristic region in the aliphatic part of the spectra, which allows to follow the course of the reaction via characteristic resonances of the methyl groups of the palladium oxidative addition complex **7a** and 1-methyl-4-(phenylethynyl)benzene (**3e**) (a) at the start of the transmetallation of **6a** and **7a** with addition of PPh_3 (10 min), (b) reaction after 4 h, (c) 20 h and (d) 20 h with addition of desired product **3e** (spiking).

Addition of PPh_3 completely stopped the reaction.

Effect of excess PPh_3 on reaction of 1a and 2a, catalyzed by $Pd(PPh_3)_2I_2$

General procedure 17 (*GP17*) – Catalytic reaction between phenylacetylene (2a) and 4-iodotoluene (1a), catalyzed by $Pd(PPh_3)_2I_2$ and addition of triphenylphosphine.

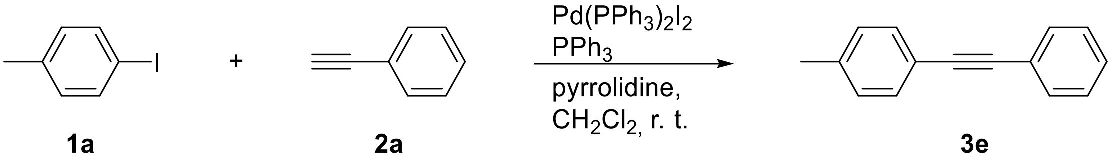

1a **2a** **3e**

An oven-dried round-bottom reaction flask was fitted with a stirring bar and sealed with a rubber septum, which was pierced with a needle connected by a tube to a Schlenk vacuum manifold. The flask was cooled to room temperature under argon atmosphere. The flask was charged with 4-iodotoluene (**1a**) (1 equiv.), phenylacetylene (**2a**) (1.1 equiv.), triphenylphosphine (0.1 or 0.5 equiv.), and pyrrolidine (2 equiv.) by rapidly opening the septum and flushing with argon. Dichloromethane (2 mL/mmol to **1a**) was added with a syringe by piercing the septum. To this solution bis(triphenylphosphine)palladium(II) iodide (0.02 equiv.) was added by rapidly opening the septum and flushing with argon. Samples (50 μL) were taken with a syringe in intervals (15 min) from the solution and transferred to dry NMR tube filled with degassed $CDCl_3$ under argon atmosphere.

Effect of 0.1 eq. of PPh_3

Following *GP17* using 4-iodotoluene (**1a**) (544 mg, 2.50 mmol), phenylacetylene (**2a**) (0.302 mL, 2.75 mmol), triphenylphosphine (69.3 mg, 0.264 mmol), pyrrolidine (0.420 mL, 5.00 mmol), bis(triphenylphosphine)palladium(II) iodide (45.1 mg, 0.05 mmol), dichloromethane (5 mL).

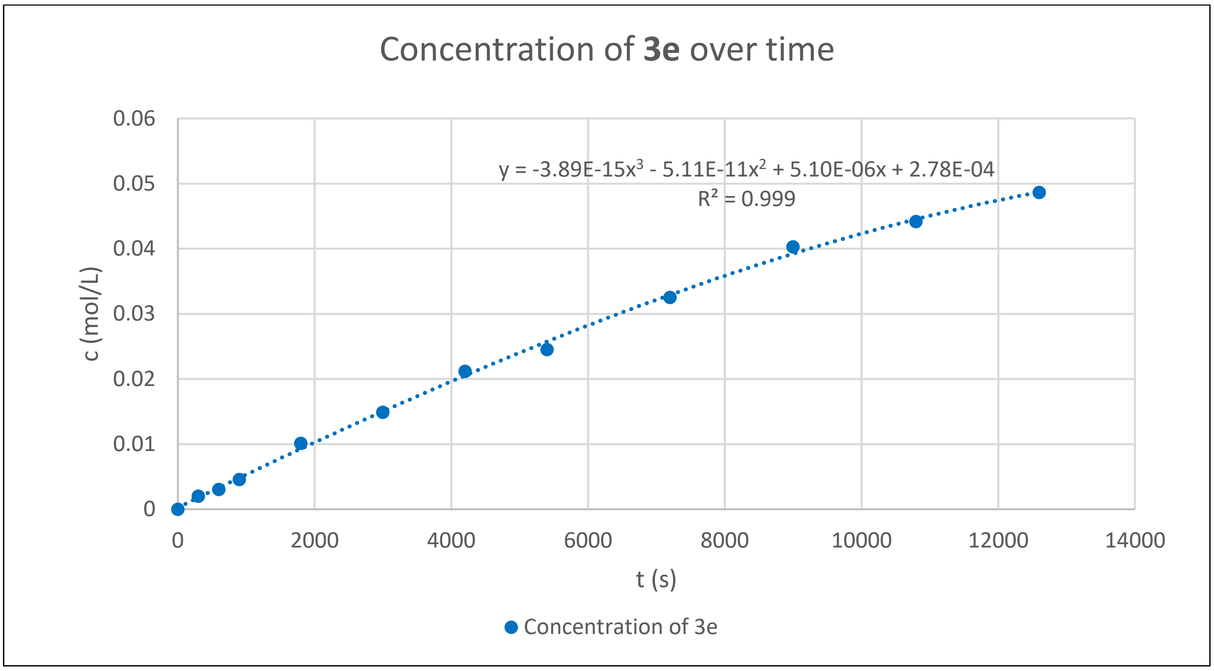

Fitted curve:

$c = A_0 + A_1 \times t + A_2 \times t^2 + A_3 \times t^3$

A_0	2.78E-04
A_1	5.10E-06
A_2	-5.11E-11
A_3	-3.89E-15

t (max) = 0 min

v (max) = $(5.1 \pm 0.4) \times 10^{-6}$ mol/Ls

Effect of 0.5 eq. of PPh_3

Following *GP17* using 4-iodotoluene (**1a**) (544 mg, 2.50 mmol), phenylacetylene (**2a**) (0.302 mL, 2.75 mmol), triphenylphosphine (329 mg, 1.25 mmol), pyrrolidine (0.420 mL, 5.00 mmol), bis(triphenylphosphine)palladium(II) iodide (44.6 mg, 0.05 mmol), dichloromethane (5 mL).

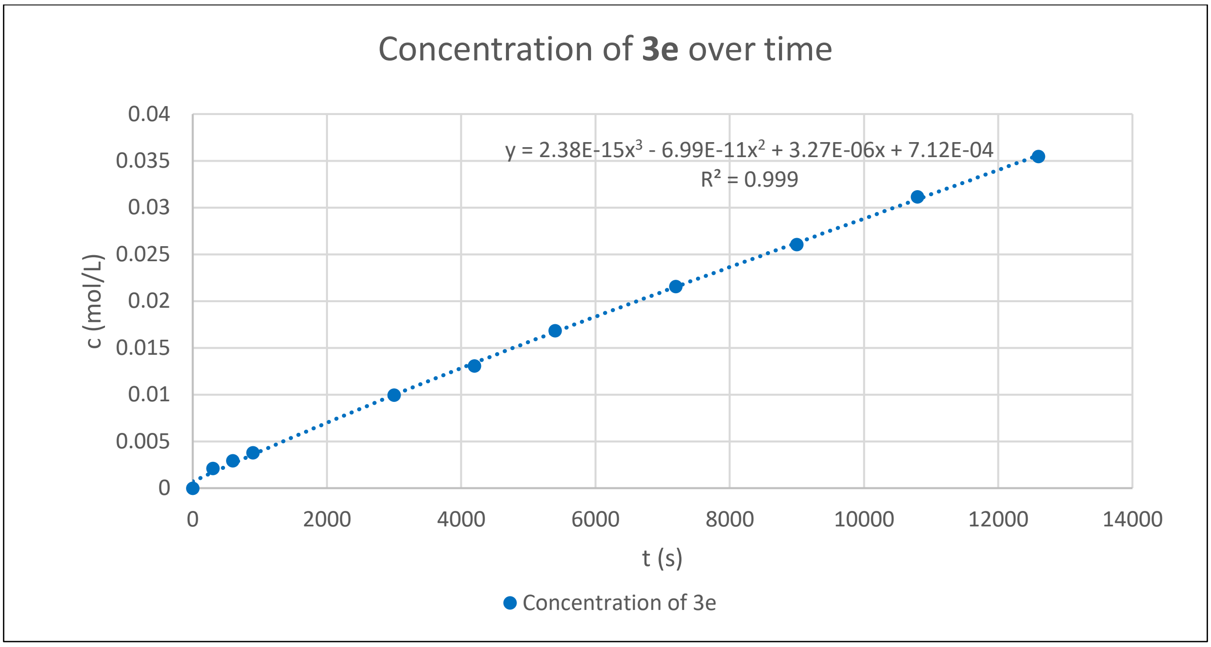

Fitted curve:

$c = A_0 + A_1 \times t + A_2 \times t^2 + A_3 \times t^3$

A_0	7.12E-04
A_1	3.27E-06
A_2	-6.99E-11
A_3	2.38E-15

t (max) = 0 min

v (max) = $(3.3 \pm 0.2) \times 10^{-6}$ mol/Ls

Comparison of the reaction rates for reactions with or without addition of PPh_3

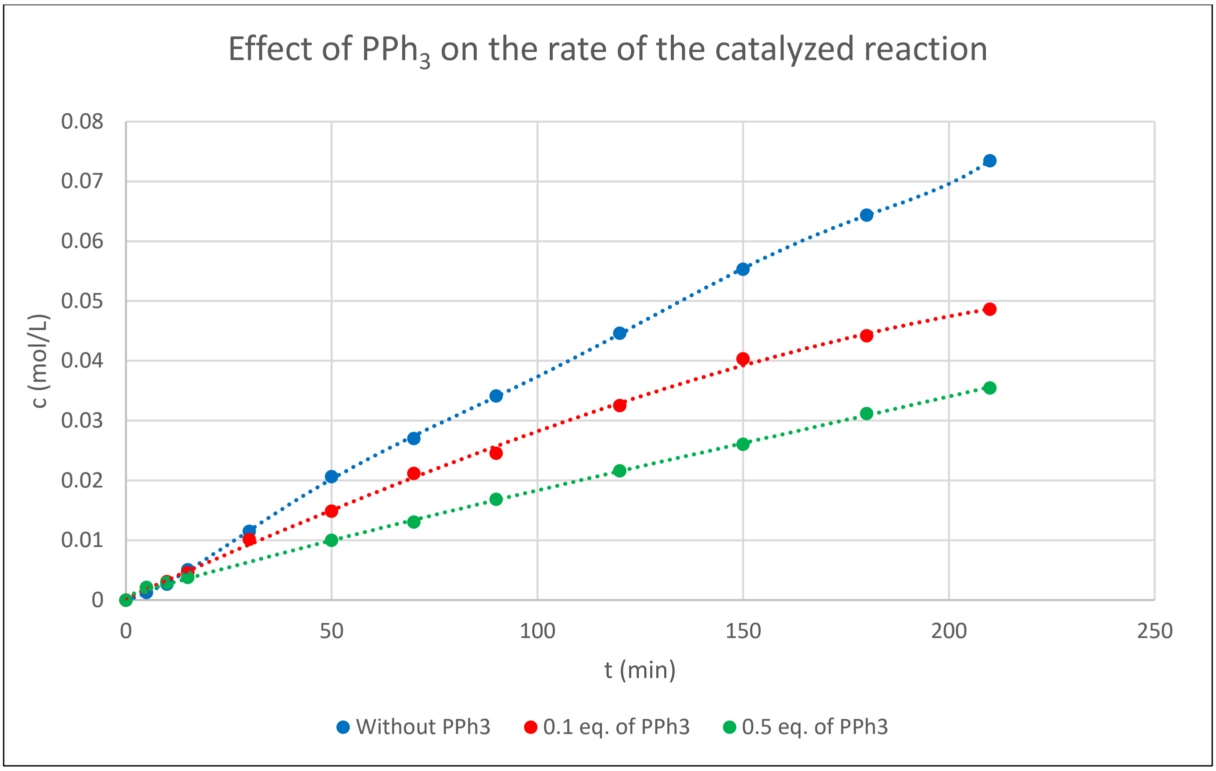

Supplementary Figure 26. Comparison of rates of catalytic reactions performed with or without addition of PPh_3.

Temperature effect on the rate of transmetallation

The effect of temperature on the rate of transmetallation was determined by measuring the initial rates of transmetallation at different temperatures. Reactions were performed according to *GP12* in $CDCl_3$ at 292, 297 and 302 K.

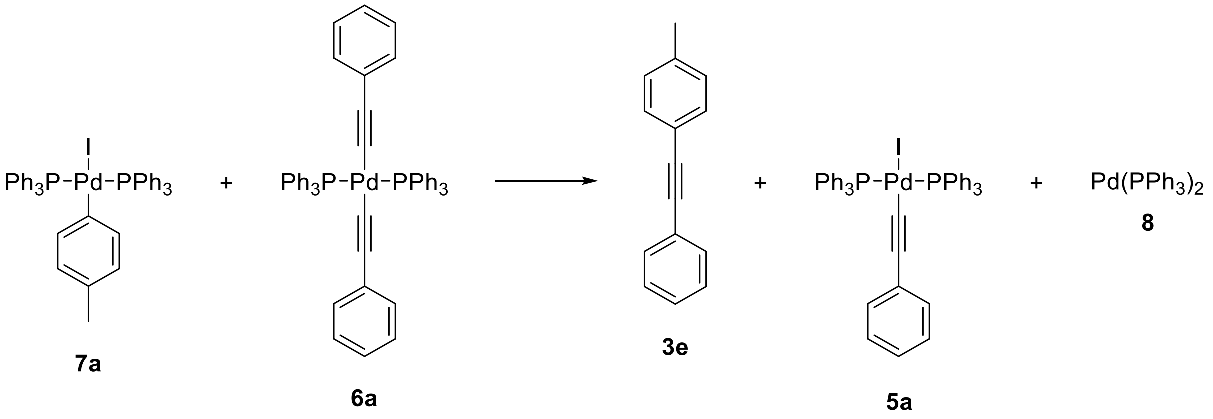

T = 292 K

c_0 (**6a**) = 0.0101 M
c_0 (**7a**) = 0.0099 M

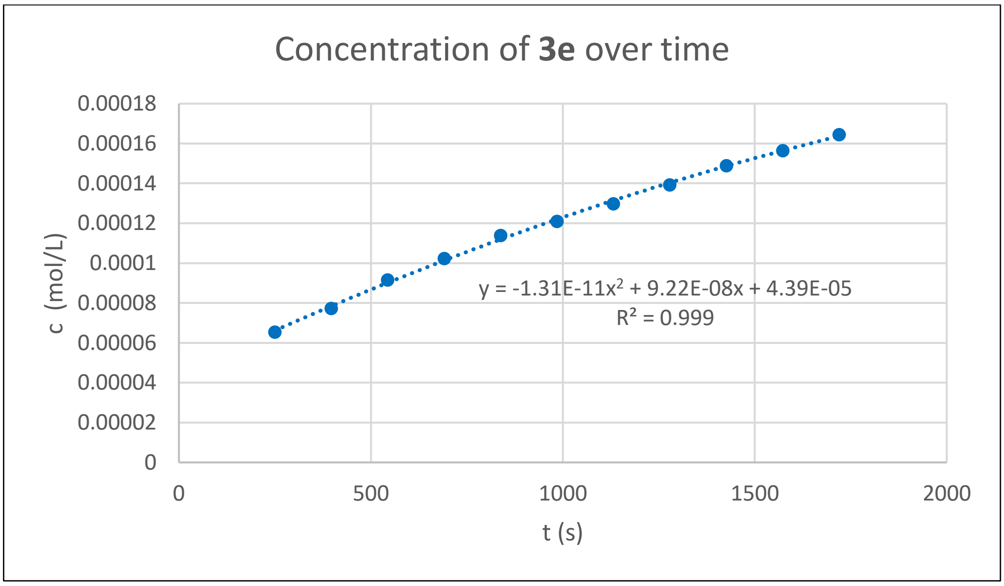

$v_0 = (9.2 \pm 0.4) \times 10^{-8}$ mol/Ls

T = 297 K

c_0 (**6a**) = 0.0102 M
c_0 (**7a**) = 0.0100 M

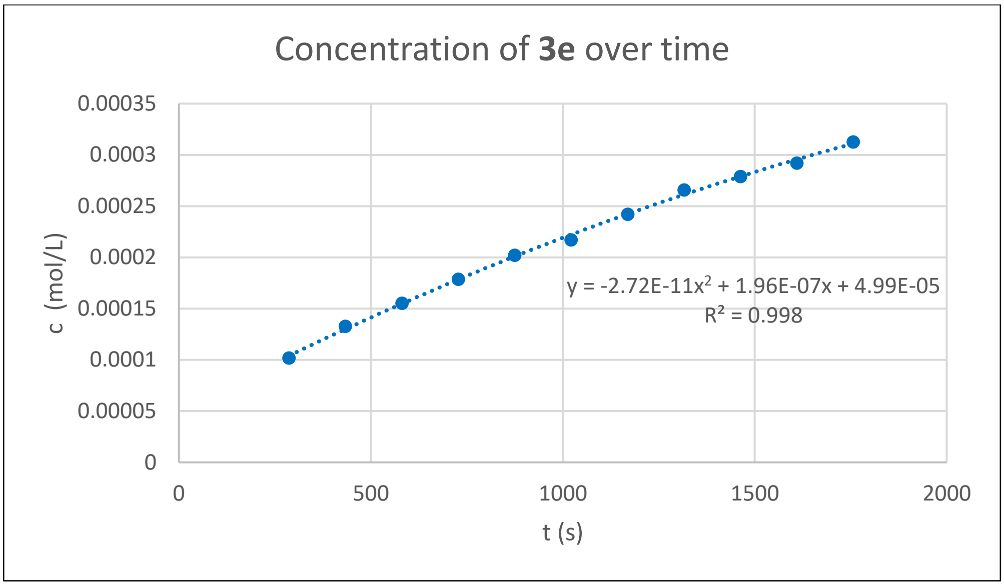

$v_0 = (2.0 \pm 0.1) \times 10^{-7}$ mol/Ls

T = 302 K

c_0 (**6a**) = 0.0102 M
c_0 (**7a**) = 0.0100 M

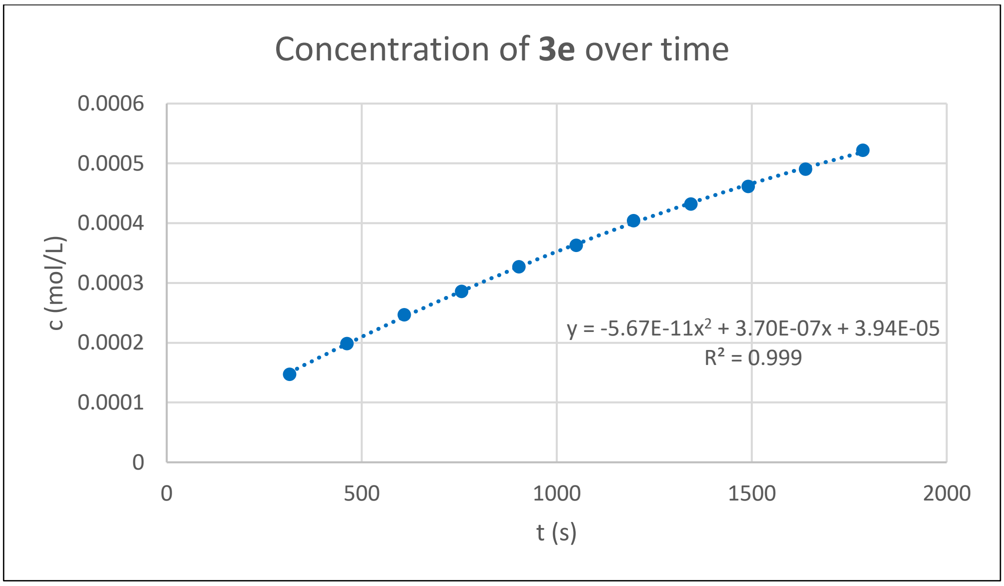

$v_0 = (3.7 \pm 0.1) \times 10^{-7}$ mol/Ls

Calculation of kinetic parameters

Kinetic parameters were calculated using formula:

$$\frac{d\, c(\mathbf{3e})}{dt} = k * [\mathbf{6a}] * [\mathbf{7a}]$$

For (t=0), it can be transformed to:

$$v_0 = k * c_0(\mathbf{6a}) * c_0(\mathbf{7a})$$

where v_0 represents the initial rate of **3e** formation and c_0 the starting concentrations of complexes **6a** and **7a**.

T [K]	c_0 (**6a**) [M]	c_0 (**7a**) [M]	v_0 [mol/L s]	k [L/mol s]
292	0.01014	0.00994	9.2E-08	0.0009151
297	0.01016	0.01000	2.0E-07	0.0019351
302	0.01008	0.01006	3.7E-07	0.0036460

Supplementary Table 1. Calculation of reaction rate constants k for different temperatures.

Calculation of ΔH‡ and ΔS‡

Thermodynamic parameters of transition state were calculated using Eyring equation:

$$\ln\left(\frac{k}{T}\right) = \frac{-\Delta H^{\ddagger}}{R} * \frac{1}{T} + \left(\frac{\Delta S^{\ddagger}}{R} + \ln\left(\frac{\kappa k_B}{h}\right)\right)$$

k…initial rate constant
T…temperature
R…gas constant
k_B…Boltzmann constant
h…Planck constant
ΔH‡…enthalpy of activation
ΔS‡…entropy of activation
κ…transmission coefficient (taken as unity)

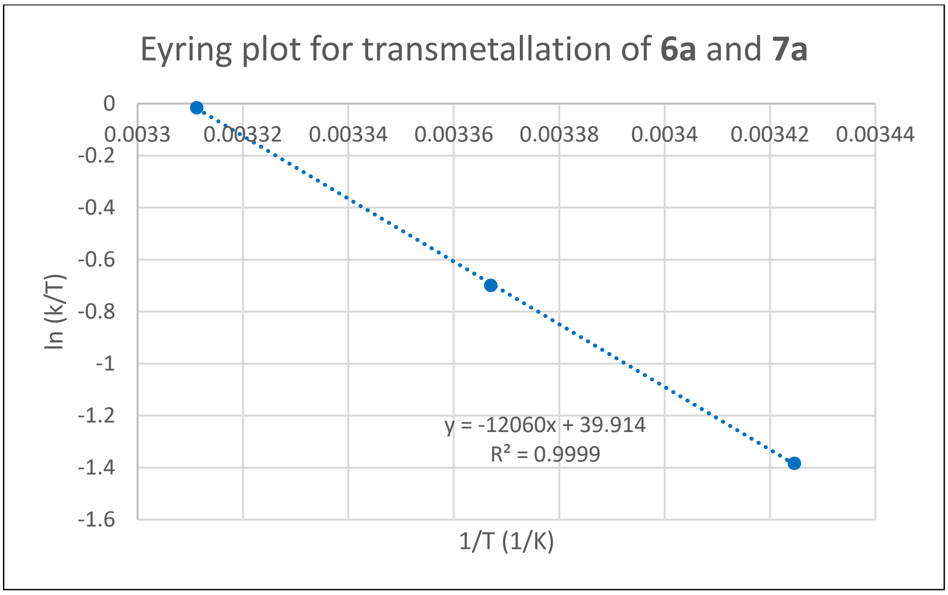

Supplementary Figure 27. Eyring plot for transmetallation of **6a** and **7a**, used for determination of ΔH and ΔS.

k (trendline) = $\frac{-\Delta H^{\ddagger}}{R}$ = - (12060 ± 108) J/mol
ΔH = (100.3 ± 0.9) kJ/mol

n (trendline) = $\left(\frac{\Delta S^{\ddagger}}{R} + \ln\left(\frac{\kappa k_B}{h}\right)\right)$ = 39.914
$\frac{\Delta S^{\ddagger}}{R}$ = (16.2 ± 0.4)

ΔS = (134±3) J/Kmol

Effect of pyrrolidine on palladium complexes 5a, 5c, 6a and 7a

Pyrrolidine is a coordinating base that can act as a ligand and is known that can substitute one of the phosphine ligands in **5a** and **7a**.

We have previously described the equilibrium of **5a** with pyrrolidine (K = 0.03 ($CDCl_3$, 300 K)):[17]

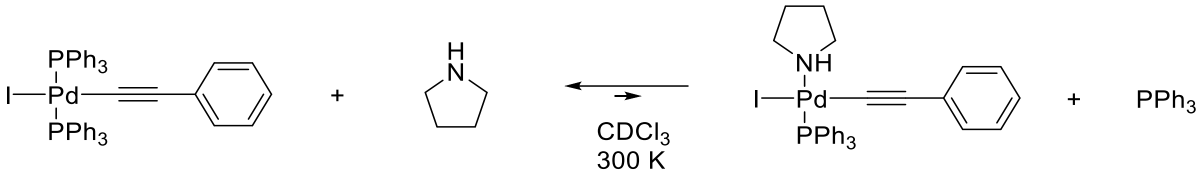

5a $^{31}P\{^1H\}$ NMR δ +22.8 ppm (s)

5a'(pyr.) $^{31}P\{^1H\}$ NMR δ +30.3 ppm (br s)

And the equilibrium of **7a** with pyrrolidine (K = 0.15 ($CDCl_3$, 300 K)):[17]

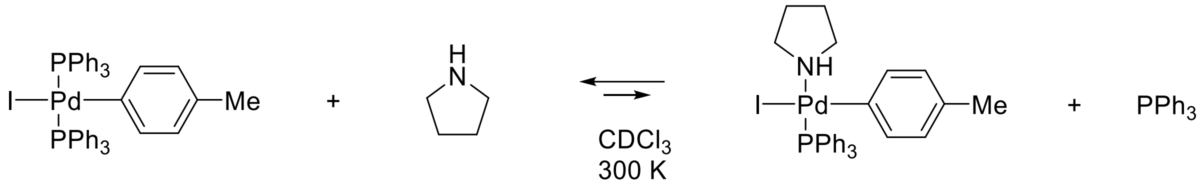

7a $^{31}P\{^1H\}$ NMR δ +22.5 ppm (s)

7a'(pyr.) $^{31}P\{^1H\}$ NMR δ +31.6 ppm (br s)

7g forms equilibrium with pyrrolidine (K = 0.080 ($CDCl_3$, 300 K))

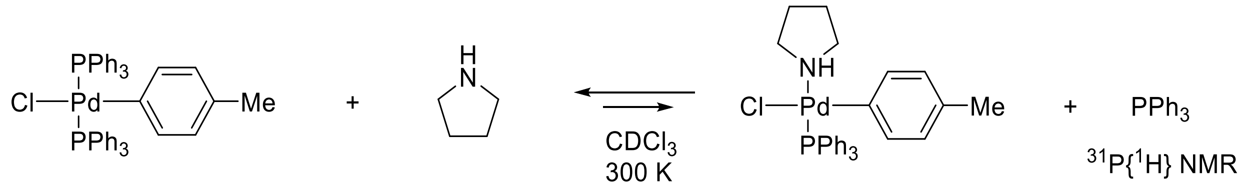

The equilibrium constant was determined by ^{1}H NMR integration (qNMR) of each component (**7g**, **7g'(pyr.)**, pyrrolidine, and triphenylphospine) using 1,3,5-trimethoxybenzene as internal standard. Since the resonances of triphenylphospine were overlapped with resonances of triphenylphospine parts from compounds **7g** and **7g'**, the concentration of triphenylphospine was postulated to be the same as the concentration of **7g'(pyr.)**. The starting amounts of **7g** and pyrrolidine in each experiment (i-iii) are shown below.

$$K = \frac{[\boldsymbol{7g'(pyr.)}][\text{triphenylphosphine}]}{[\boldsymbol{7g}][\text{pyrrolidine}]}$$

i. **7g** (8.15 mg, 0.0108 mmol), pyrrolidine (3.55 mg, 0.050 mmol) in $CDCl_3$ (1.0 mL) at 300 K,

   K = 0.336

ii. **7g** (7.53 mg, 0.00994 mmol), pyrrolidine (6.12 mg, 0.0860 mmol) in $CDCl_3$ (1.0 mL) at 300 K,

   K = 0.354

iii. **7g** (6.94 mg, 0.00916 mmol), pyrrolidine (12.15 mg, 0.171 mmol) in $CDCl_3$ (1.0 mL) at 300 K,

   K = 0.334

K = 0.341 ($CDCl_3$, 300 K)

7g'(pyr.):

^{1}H NMR (500 MHz, $CDCl_3$) δ 6.83–6.79 (m, 2H), 6.47 (d, J = 7.7 Hz, 2H), 3.44 (br s, 1H), 3.25–3.17 (m, 4H), 2.65–2.57 (m, 4H), 2.09 (s, 3H), P(Ph_3) resonances of **7g'(pyr.)** were overleaped with P(Ph_3) resonances from other compounds in the mixture;

$^{31}P\{^1H\}$ NMR (202 MHz, $CDCl_3$) δ +30.80 (s);

HMRS (ESI+) (*m*/*z*): calculated for $C_{29}H_{32}ClNPPd^+$ $[M+H]^+$ 566.0991, found 566.0994.

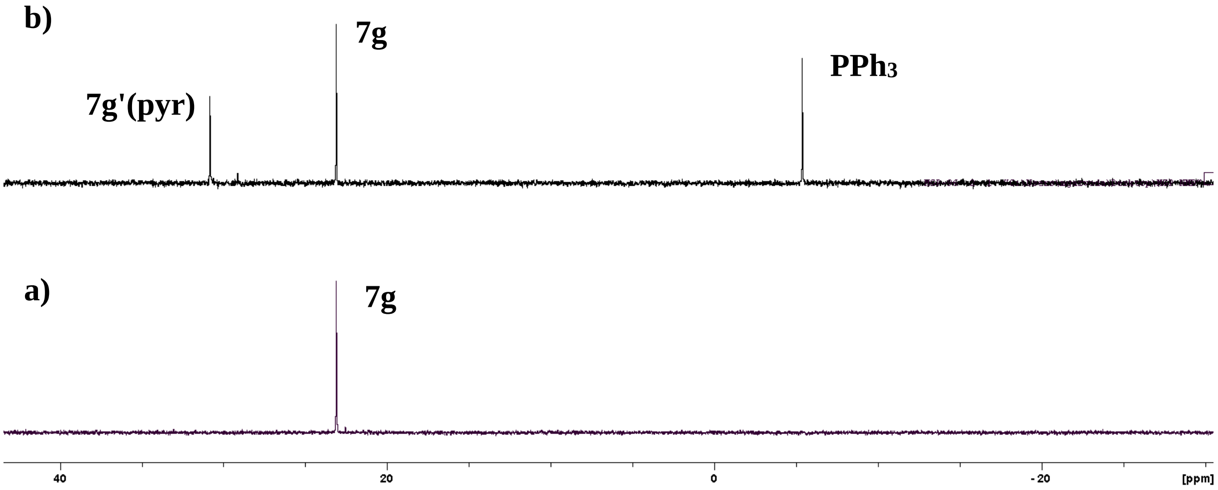

Supplementary Figure 28. $^{31}P\{^1H\}$ NMR spectra of starting **7g** (a) and mixture of **7g** (8.15 mg, 10.89 μmol) in $CDCl_3$ (1.00 mL) in the presence of pyrrolidine (3.55 mg, 50 μmol) after 10 min.

5c forms equilibrium with pyrrolidine (K = 0.080 ($CDCl_3$, 300 K))

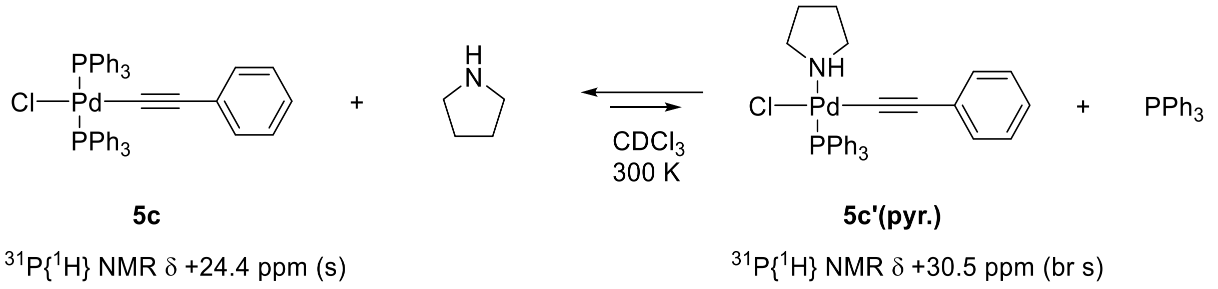

The equilibrium constant was determined by ^{1}H NMR integration (qNMR) of each component (**5c**, **5c'(pyr.)**, pyrrolidine, and triphenylphospine) using 1,3,5-trimethoxybenzene as internal standard. Since the resonances of triphenylphospine were overlapped with resonances of triphenylphospine parts from compounds **5c** and **5c'**, the concentration of triphenylphospine was postulated to be the same as the concentration of **5c'(pyr.)**. The starting amounts of **5c** and pyrrolidine in each experiment (i-iii) are shown below.

$$K = \frac{[\mathbf{5}\boldsymbol{c}'(\boldsymbol{pyr}.)][\text{triphenylphosphine}]}{[\mathbf{5}\boldsymbol{c}][\text{pyrrolidine}]}$$

i. **5c** (7.29 mg, 0.00950 mmol), pyrrolidine (6.05 mg, 0.0851 mmol) in $CDCl_3$ (1.0 mL) at 300 K,
   K = 0.076
ii. **5c** (6.03 mg, 0.00786 mmol), pyrrolidine (8.68 mg, 0.1220 mmol) in $CDCl_3$ (1.0 mL) at 300 K,
   K = 0.087
iii. **5c** (7.18 mg, 0.00935 mmol), pyrrolidine (3.45 mg, 0.0487 mmol) in $CDCl_3$ (1.0 mL) at 300 K,
   K = 0.078

K = 0.080 ($CDCl_3$, 300 K)

5c'(pyr.):

^{1}H NMR (500 MHz, $CDCl_3$) δ 7.08–7.01 (m, 3H), 6.78–6.72 (m, 2H), 3.84 (br s, 1H), 3.43–3.32 (m, 4H), 1.97–1.88 (m, 4H), $P(Ph_3)$ resonances of **5c'(pyr.)** were overleaped with $P(Ph_3)$ resonances from other compounds in the mixture;

$^{31}P\{^1H\}$ NMR (202 MHz, $CDCl_3$) δ +30.50 (br s);

HMRS (ESI+) (*m*/*z*): calculated for $C_{30}H_{29}NPPd^+$ $[M-Cl]^+$ 540.1067, found 540.1067.

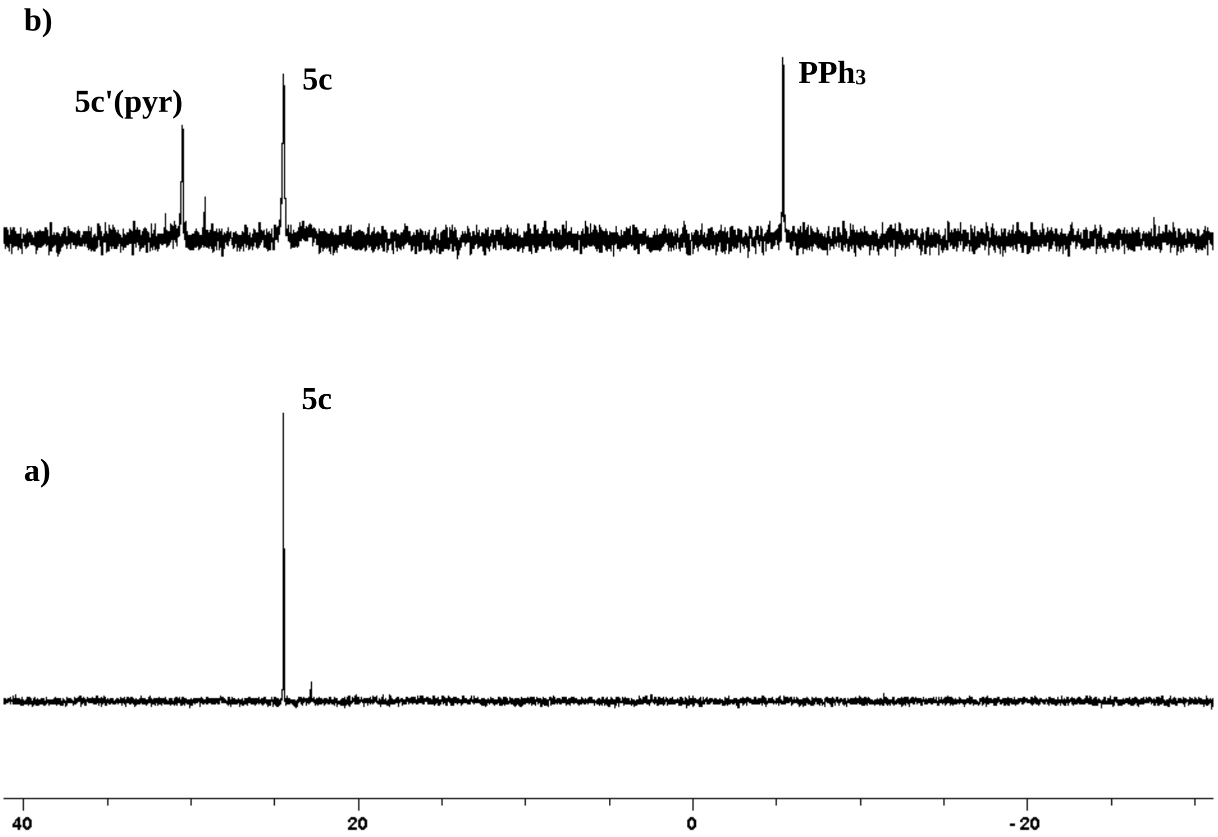

Supplementary Figure 29. $^{31}P\{^1H\}$ NMR spectra of starting **5c** (a) and mixture of **5c** (6.80 mg, 8.9 μmol) in $CDCl_3$ (0.800 mL) in the presence of pyrrolidine (6.0 mg, 84.3 μmol) after 10 min.

Complex **6a** does not form the equilibrium with pyrrolidine, as judged from $^{31}P\{^{1}H\}$ NMR spectra over time, however it starts slowly decomposing after 1 h:

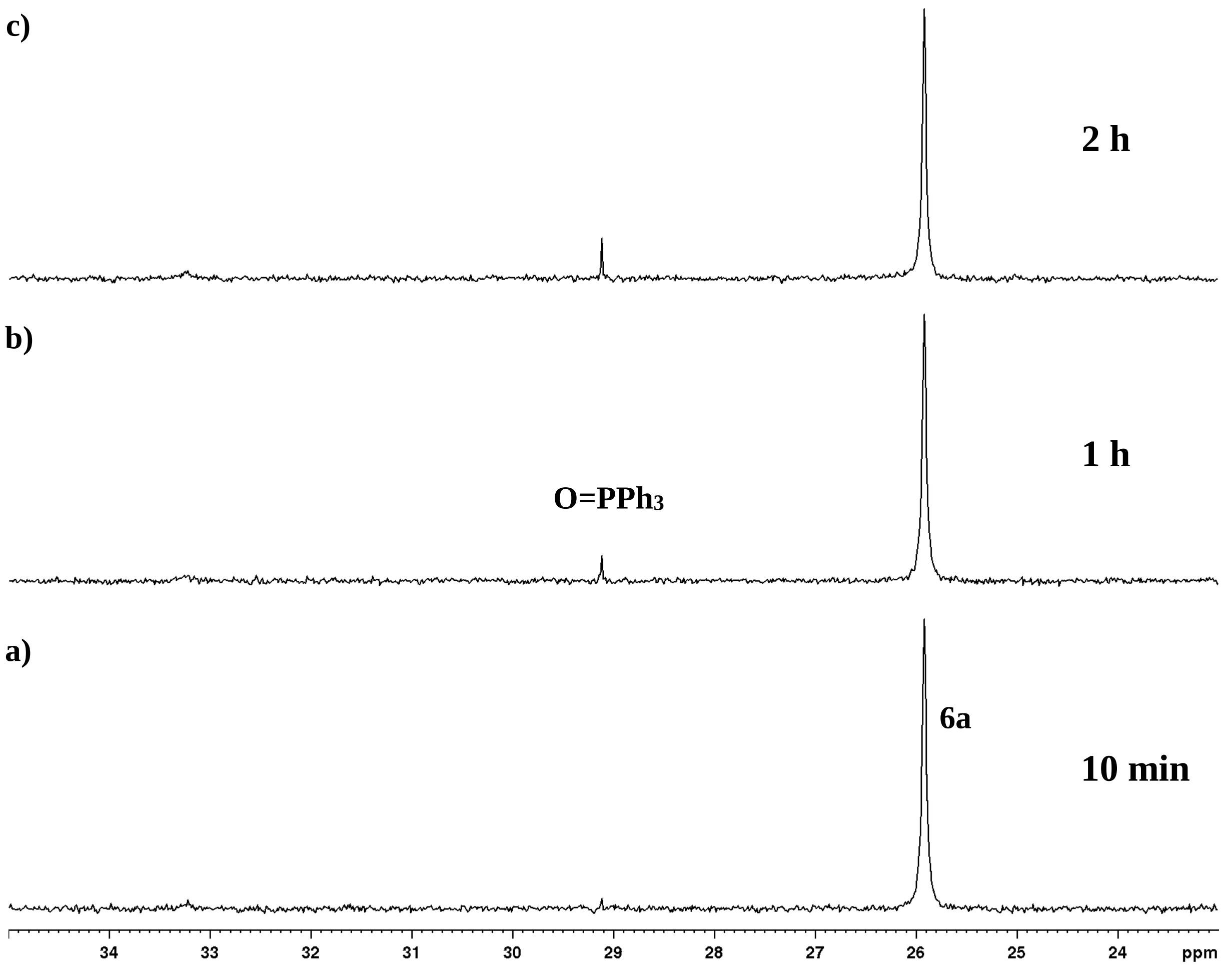

Supplementary Figure 30. Stack of $^{31}P\{^{1}H\}$ NMR spectra of **6a** (6.68 mg, 8.02 μmol) in $CDCl_3$ (0.800 mL) in the presence of pyrrolidine (6.62, 80.1 μmol) after 10 min (a), 1 h (b) and 2 h (c).

Synthesis and reactivity of tricoordinated palladium oxidative addition complex 7a'

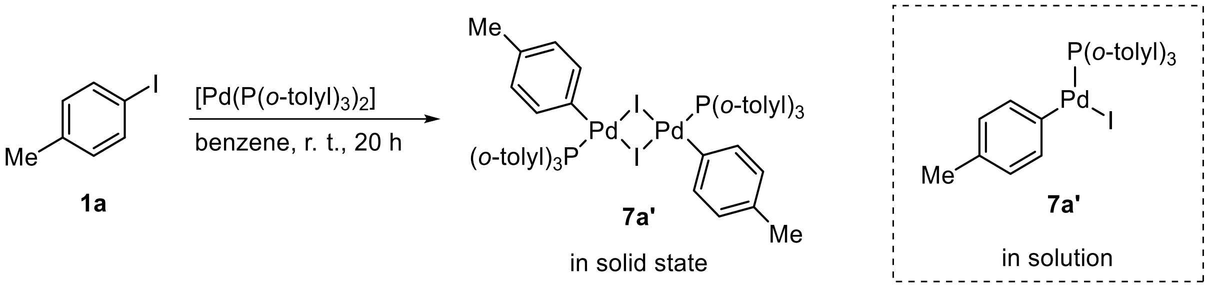

Tricoordinated palladium oxidative addition complex **7a'** was prepared according to the modified literature procedure.[40] Reaction was performed at room temperature (296 K) in nitrogen-filled glovebox. In a vial 4-iodotoluene (**1a**) (170 mg, 0.78 mmol) was dissolved in dry benzene (4 mL). To this solution bis(tri-*o*-tolylphosphine)palladium(0) (96 mg, 0.134 mmol) was added and resulting solution was stirred at room temperature overnight. After 20 h, it was quickly filtered through filter paper. Filtrate was collected and concentrated *in vacuo*. Resulting mixture was suspended in diethyl ether and stored at -20 °C overnight. Brownish suspension was then filtered through filter paper, yielding solid brownish powder (53 mg, 0.0843 mmol, 63%).

Mp: decomposes at 162.3 °C.

^{1}H NMR (500 MHz, $CDCl_3$) δ = 7.85–7.18 (br, 19H, Ar*H* and solvents), 7.18–6.44 (br, 14H, Ar*H*), 6.44–6.20 (br, 4H, Ar*H*), 1.99 (s, 6H, Me).

^{31}P{^{1}H} NMR (202 MHz, $CDCl_3$) δ = +25.4 (br s).

HRMS: calculated for $C_{28}H_{28}PPd$ $[(M/2)-I]^+$ 501.0963, found 501.0958.

Spectroscopic data are in agreement with literature.[40]

Transmetallation of 7a' and 6a

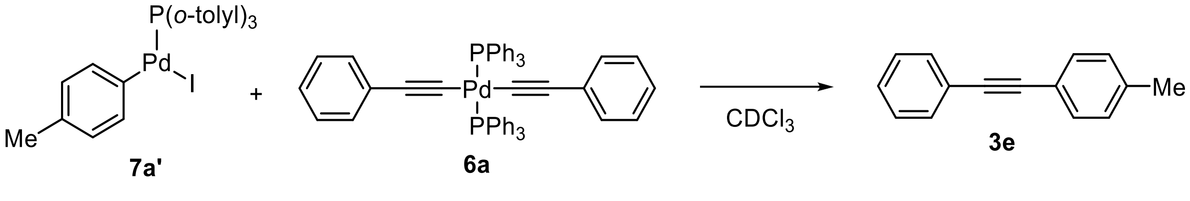

In a vial a solution of (tri(o-tolyl)phosphine)palladium(0) (4-methylphenylide) iodide dimer (**7a'**) (6.40 mg, 5.09 µmol of dimer, 10.2 µmol of monomer) in $CDCl_3$ was prepared under argon atmosphere. Bis(triphenylphosphine)palladium bis(phenylethynide) (**6a**) (6.77 mg, 8.12 µmol) was added to this solution in one portion. The reaction mixture was sonicated for 0.5 min and transferred to NMR tube, flushed with argon and sealed. NMR spectra were acquired in 2.5 min intervals.

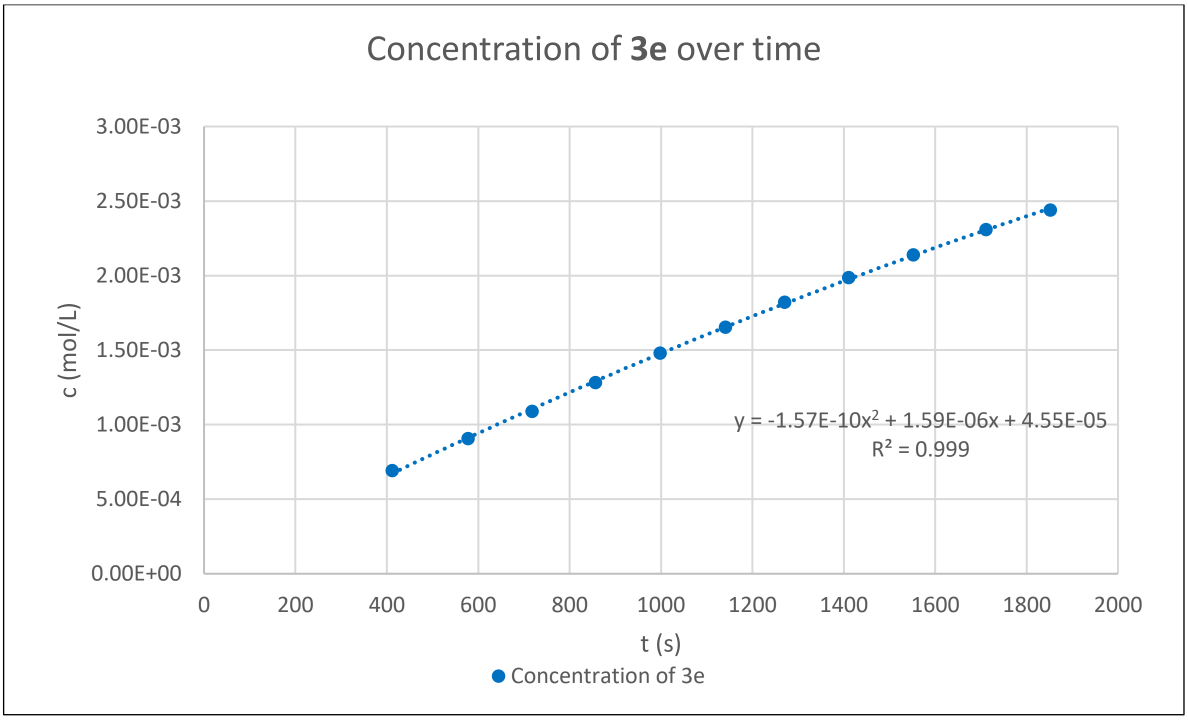

Time [s]	c [mol/L]
0	0
412	6.91E-04
578	9.05E-04
718	1.09E-03
857	1.28E-03
998	1.48E-03
1141	1.65E-03
1271	1.82E-03
1411	1.99E-03
1552	2.14E-03
1711	2.31E-03
1852	2.44E-03

$v_0 = (1.59 \pm 0.04) \times 10^{-6}$ mol/Ls

Slight excess (1.25 equiv.) of **7a’** was used in both experiments due to its instability, i.e. aryl group scrambling and side reactions.[41,42]

Comment:

It should be noted that complex **7a'** is known to be unstable and decomposes during the reaction,[41,42] forming 4,4'-dimethyl-1,1'-biphenyl in addition to the tolane product 1-methyl-4-(phenylethynyl)benzene (**3e**) (see Supplementary Fig. 32). Thus, the actual transmetallation rate

of **7a'** and **6a** could be higher than the observed rate if **7a'** reacts only with **6a**. Most likely, it would be higher than the observed rate for the catalytic reaction and also for the formation of palladium bisacetylide **6a** from palladium monoacetylide **5a**, which was found to be the rate-limiting step of the process.

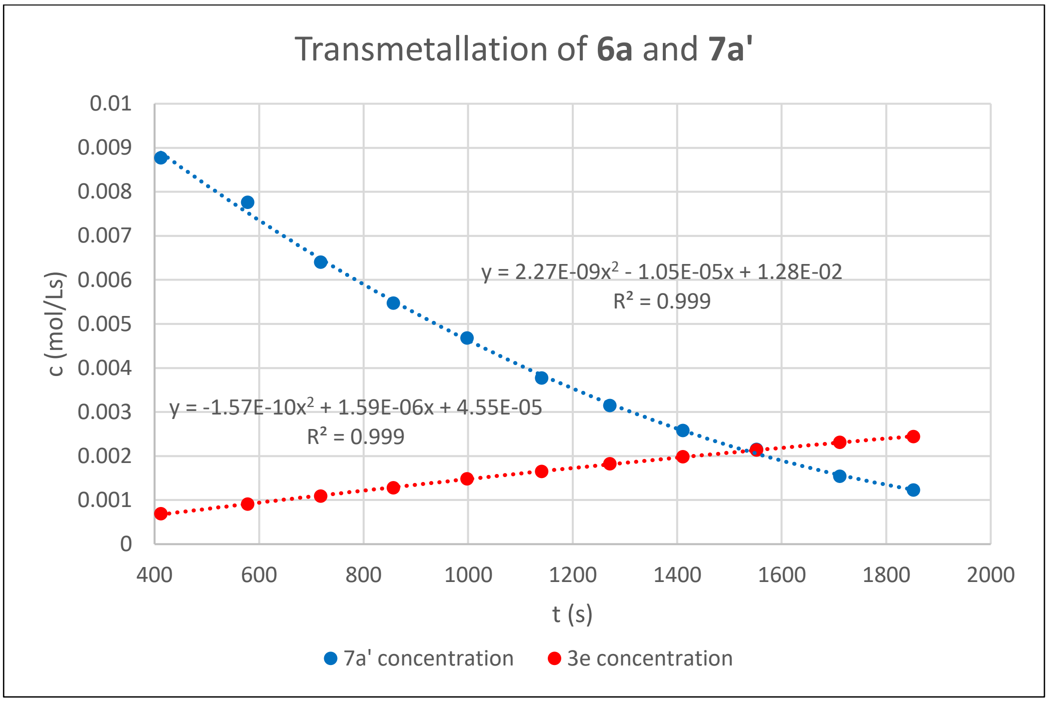

Supplementary Figure 31. Comparison of product **3e** formation and decomposition of **7a'**. Rate of decomposition is 6 times higher.

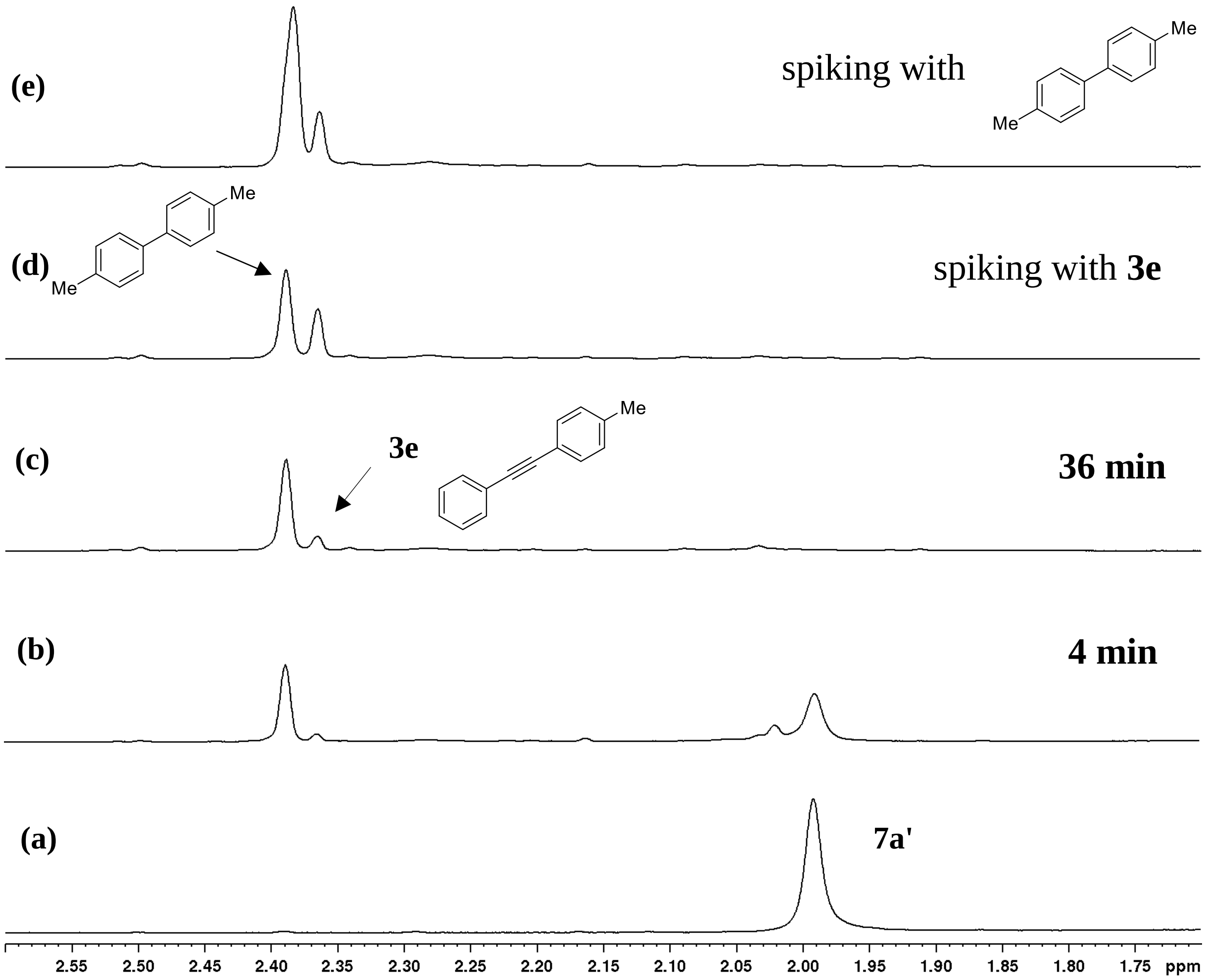

Supplementary Figure 32. Stack of ^{1}H NMR spectra of the characteristic region in the aliphatic part of the spectra, which allows to follow the course of the reaction via characteristic resonances of the methyl groups of the palladium oxidative addition complex **7a'**, 1-methyl-4-(phenylethynyl)benzene (**3e**) and 4,4'-dimethyl-1,1'-biphenyl: (a) palladium complex **7a'**, (b) transmetallation reaction of **7a'** and **6a** after 4 minutes, (c) end of the reaction after 36 min, after which the reaction stopped due to complete consumption of **7a'**, (d) reaction mixture spiked with **3e**, (e) reaction mixture spiked with biaryl standard 4,4'-dimethyl-1,1'-biphenyl.

Reformation of palladium bisacetylide 6a from monoacetylide 5a

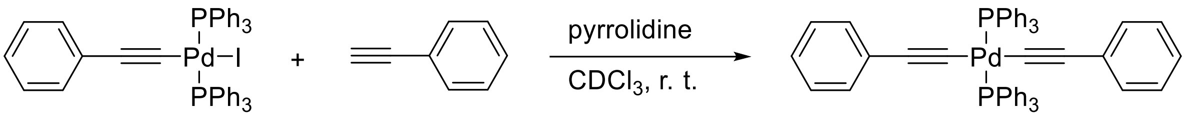

5a **2a** **6a**

In a vial a solution of phenylacetylene (**2a**) (13.2 µL, 0.120 mmol) and pyrrolidine (13.2 µL, 0.160 mmol) in degassed $CDCl_3$ (0.800 mL) was prepared under argon atmosphere. The solution was transferred to the vial containing bis(triphenylphosphine)palladium(II) (phenylethynide) iodide (**5a**) (6.92 mg, 8.06 µmol), mixed for 0.5 min and transferred into NMR tube. NMR tube was purged with argon and sealed. The reaction was analyzed by recording ^1H NMR (T = 302 K, interval 147 s).

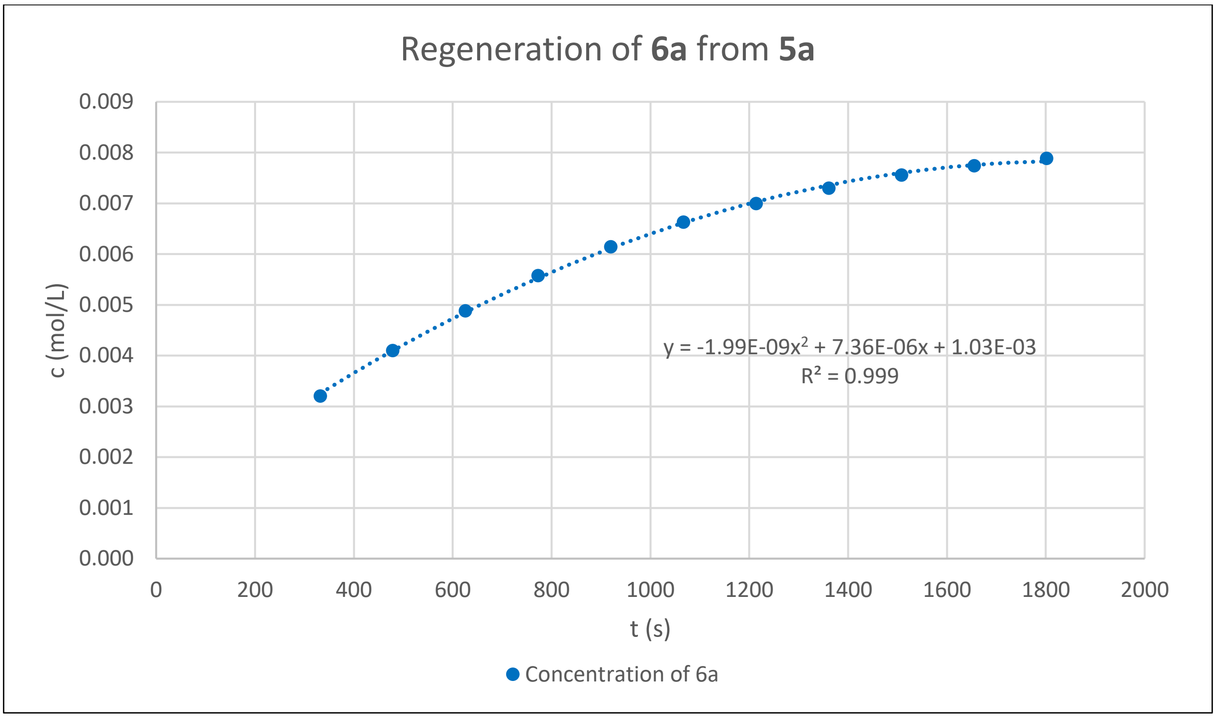

$v_0 = (7.4 \pm 0.2) \times 10^{-6}$ mol/Ls

Time [s]	c [mol/L]
332	3.20E-03
479	4.10E-03
626	4.89E-03
773	5.58E-03
920	6.15E-03
1067	6.63E-03
1214	7.00E-03
1361	7.30E-03
1508	7.56E-03
1655	7.74E-03
1802	7.89E-03

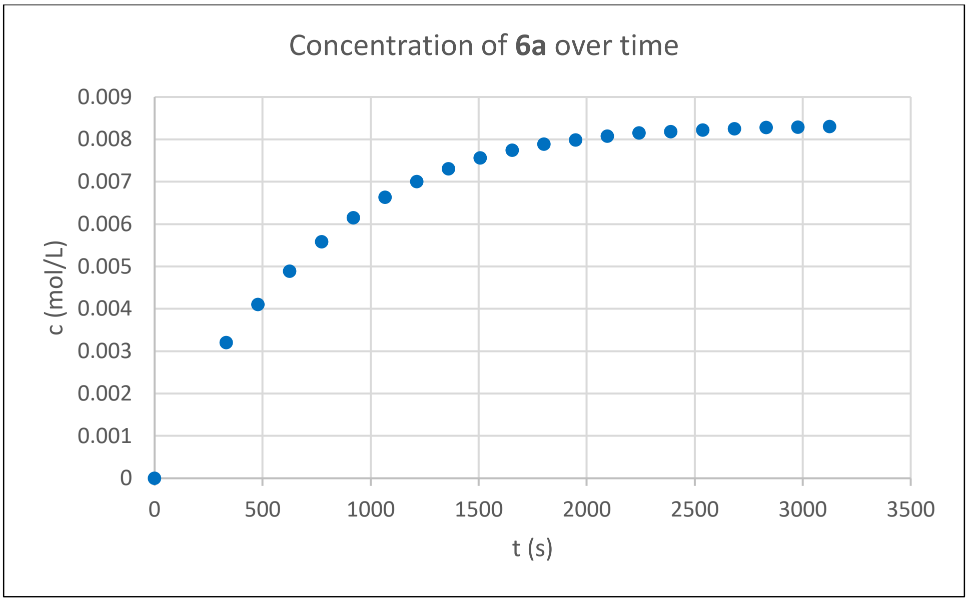

Comparison of formation of 3e in catalytic reaction of 1a and 2a with palladium bisacetylide formation 6a from Pd(PPh$_3$)I$_2$

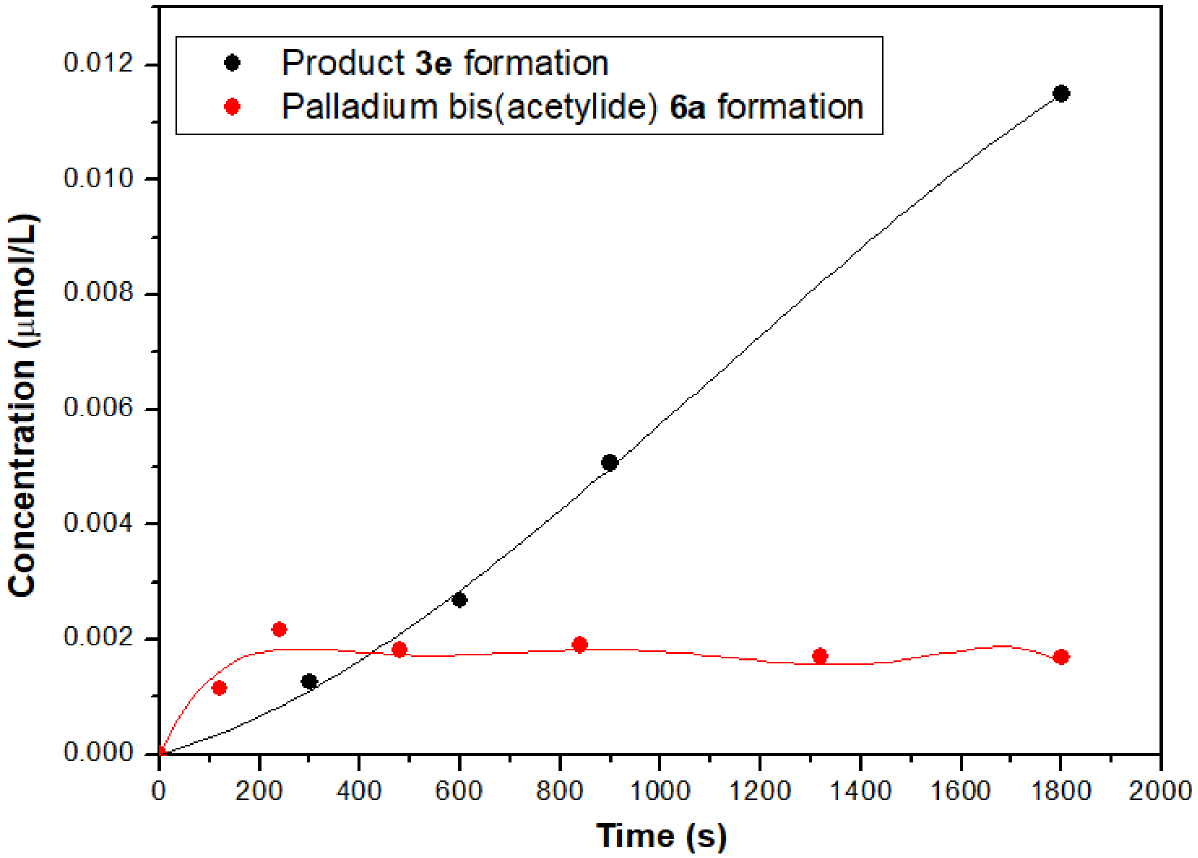

Comparing the formation of product **3e** in catalytic reaction and palladium bisacetylide **6a** under similar reaction conditions.

Kinetic studies of catalytic reactions under synthetically relevant conditions

General procedure 18 (*GP18*) – Catalytic reaction between phenylacetylene (2a) and 4-iodotoluene (1a), catalyzed by $Pd(PPh_3)_2I_2$

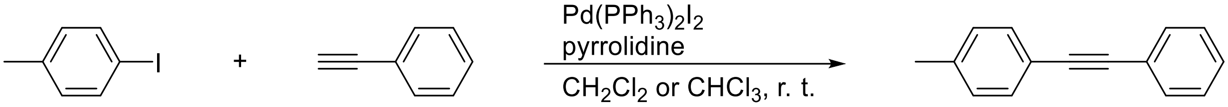

1a **2a** **3e**

An oven-dried round-bottom reaction flask was fitted with a stirring bar and sealed with a rubber septum, which was pierced with a needle connected by a tube to a Schlenk vacuum manifold. The flask was cooled to room temperature under argon atmosphere. The flask was charged with 4-iodotoluene (**1a**) (1 equiv.), phenylacetylene (**2a**) (1.1 equiv.) and pyrrolidine (2 equiv.) by rapidly opening the septum and flushing with argon. Dichloromethane or chloroform (2 mL/mmol to **1a**) was added with a syringe by piercing the septum. To this solution bis(triphenylphosphine)palladium(II) iodide (0.02 equiv.) was added by rapidly opening the septum and flushing with argon. Aliquotes (50 μL) were taken with a syringe in intervals (15 min) from the solution and transferred to dry NMR tube filled with degassed $CDCl_3$ (0.7 mL) under argon atmosphere.

Catalytic reaction of 1a and 2a in dichloromethane

Following *GP18* using 4-iodotoluene (**1a**) (547 mg, 2.51 mmol), phenylacetylene (**2a**) (0.302 mL, 2.75 mmol), pyrrolidine (0.420 mL, 5.00 mmol), bis(triphenylphosphine)palladium(II) iodide (43.9 mg, 0.05 mmol), dichloromethane (5 mL).

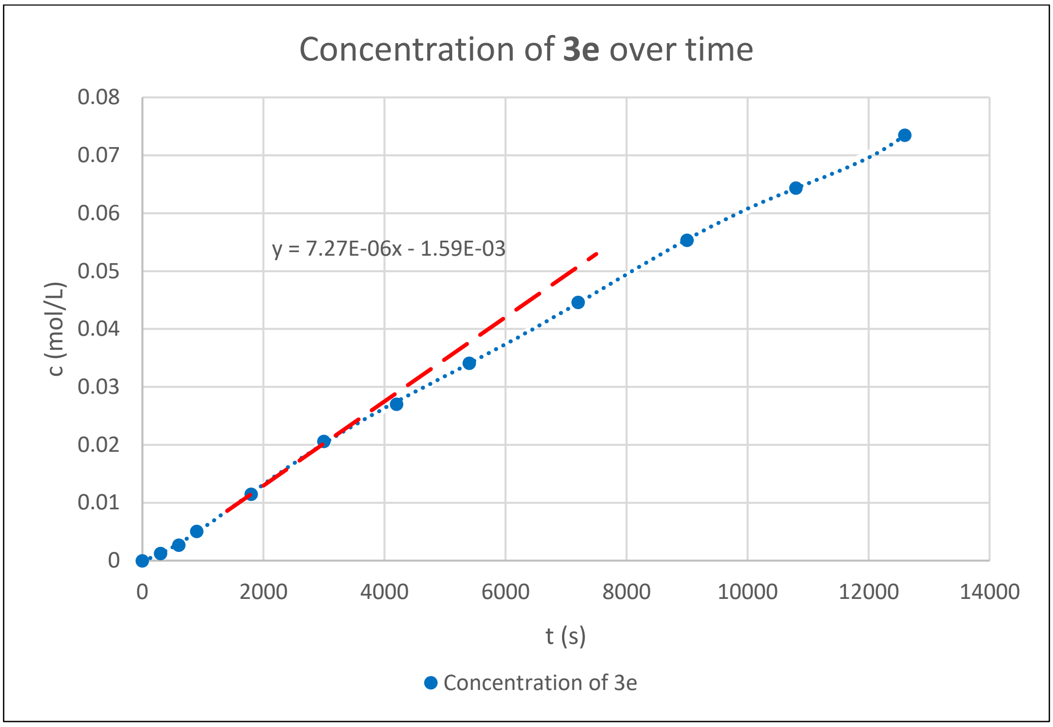

Fitted curve:

$c = A_0 + A_1 \times t + A_2 \times t^2 + A_3 \times t^3 + A_4 \times t^4 + A_5 \times t^5 + A_6 \times t^6$

A_0	-2.52E-04
A_1	4.62E-06
A_2	1.93E-09
A_3	-6.22E-13
A_4	8.60E-17
A_5	-5.48E-21
A_6	1.31E-25

t (max) = 28 min

v (max) = 7.27×10^{-6} mol/Ls

Catalytic reaction of 1a and 2a in chloroform

Following *GP18* using 4-iodotoluene (**1a**) (549 mg, 2.52 mmol), phenylacetylene (**2a**) (0.302 mL, 2.75 mmol), pyrrolidine (0.420 mL, 5.00 mmol), bis(triphenylphosphine)palladium(II) iodide (44.5 mg, 0.05 mmol), chloroform (5 mL).

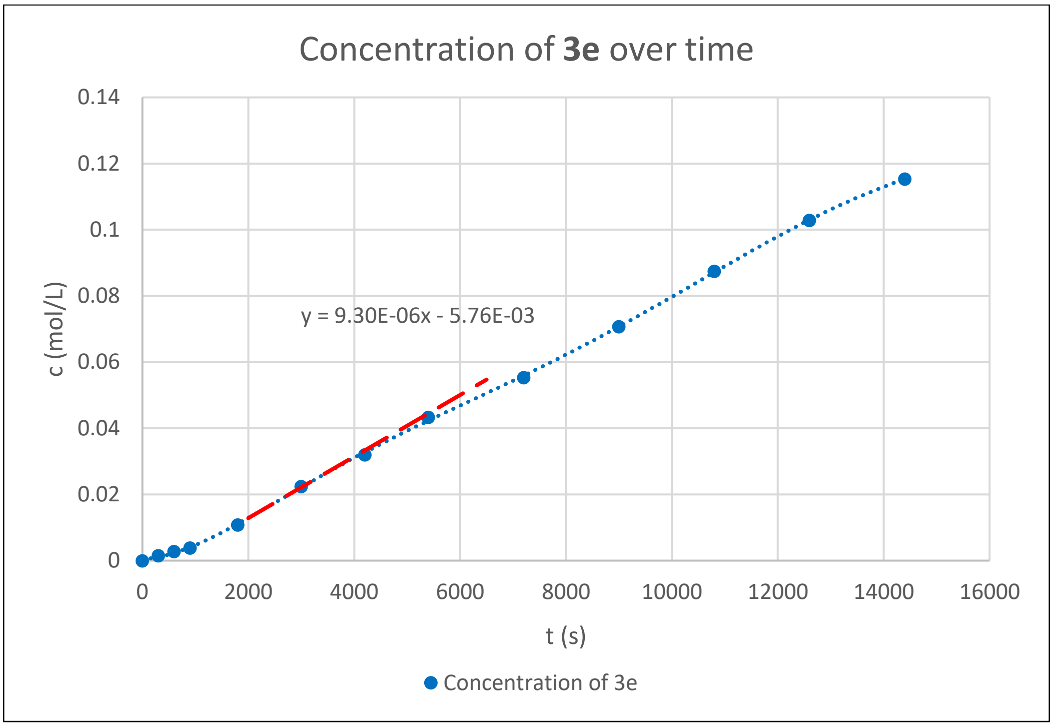

Fitted curve:

$c = A_0 + A_1 \times t + A_2 \times t^2 + A_3 \times t^3 + A_4 \times t^4 + A_5 \times t^5 + A_6 \times t^6$

A_0	4.65E-04
A_1	8.05E-07
A_2	4.35E-09
A_3	-1.03E-12
A_4	1.14E-16
A_5	-5.87E-21
A_6	1.13E-25

t (max) = 43 min

v (max) = 9.30×10^{-6} mol/Ls

Comparison of reaction rates of catalytic reactions in dichloromethane and chloroform

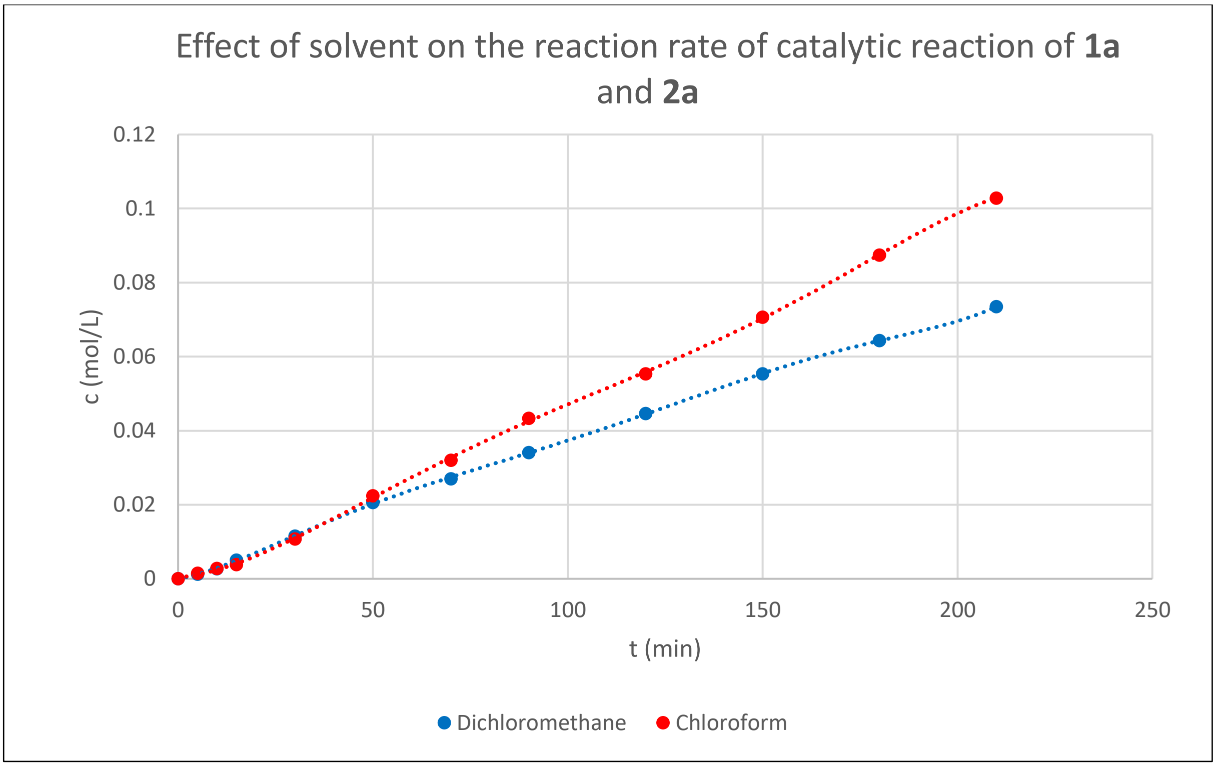

Comparison of rates of catalytic reactions of **1a** and **2a** performed in dichloromethane or chloroform.

Order in palladium in the catalytic reaction employing $Pd(PPh_3)_2I_2$ as precatalyst

Order in $Pd(PPh_3)_2I_2$ was determined by measuring the reaction rates at different catalyst loadings. Reactions were performed according to *GP12* in CH_2Cl_2 at room temperature (296 K).

1 mol% of $Pd(PPh_3)_2I_2$

Following *GP18* using 4-iodotoluene (**1a**) (551 mg, 2.53 mmol), phenylacetylene (**2a**) (0.302 mL, 2.75 mmol), pyrrolidine (0.420 mL, 5.00 mmol), bis(triphenylphosphine)palladium(II) iodide (22.6 mg, 0.026 mmol), dichloromethane (5 mL).

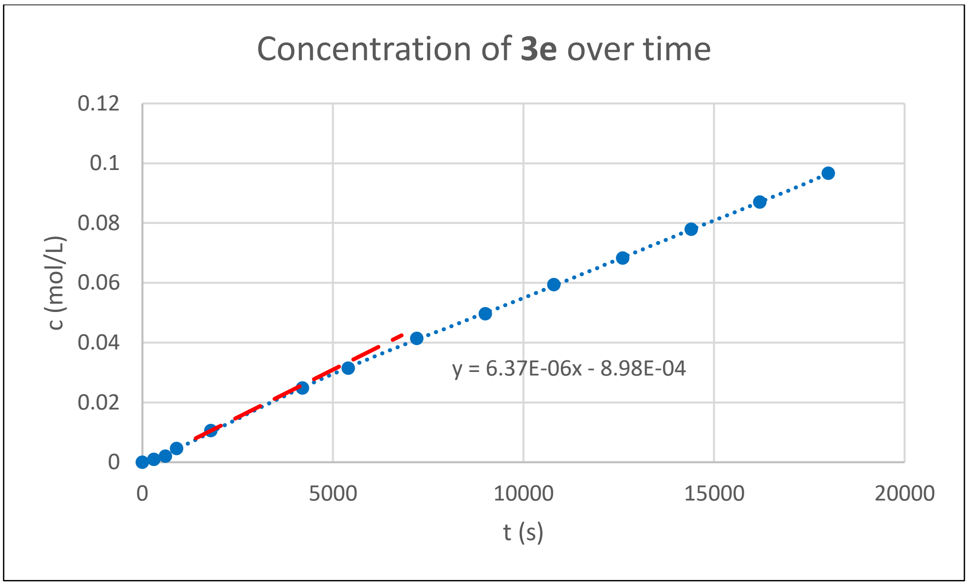

Fitted curve:

$c = A_0 + A_1 \times t + A_2 \times t^2 + A_3 \times t^3 + A_4 \times t^4 + A_5 \times t^5 + A_6 \times t^6$

A_0	-4.25E-04
A_1	4.85E-06
A_2	8.53E-09
A_3	-2.07E-13
A_4	2.07E-17
A_5	-9.43E-22
A_6	1.63E-26

t (max) = 36 min

v (max) = 6.37×10^{-6} mol/Ls

2 mol% of $Pd(PPh_3)_2I_2$

Following *GP18* using 4-iodotoluene (**1a**) (547 mg, 2.51 mmol), phenylacetylene (**2a**) (0.302 mL, 2.75 mmol), pyrrolidine (0.420 mL, 5.00 mmol), bis(triphenylphosphine)palladium(II) iodide (43.9 mg, 0.05 mmol), dichloromethane (5 mL).

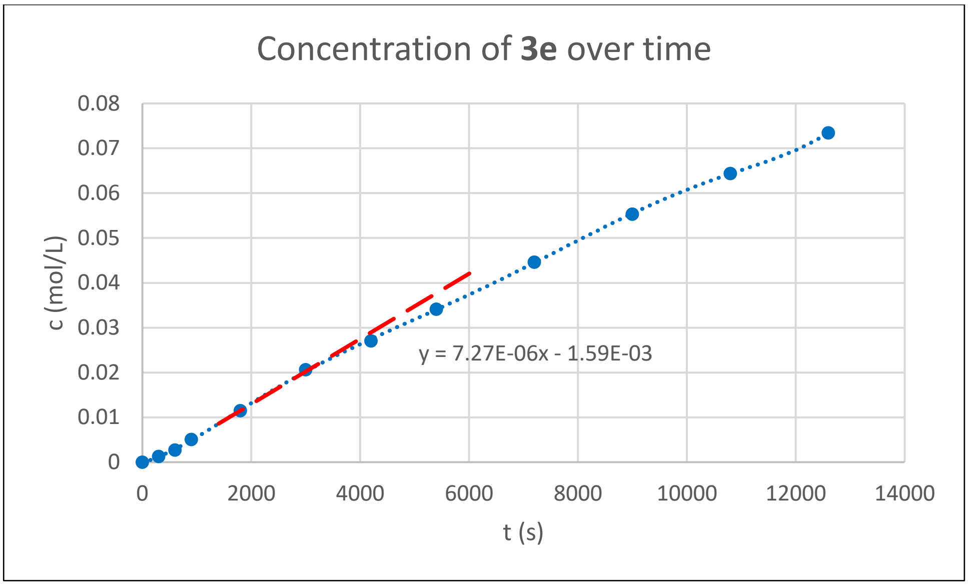

Fitted curve:

$c = A_0 + A_1 \times t + A_2 \times t^2 + A_3 \times t^3 + A_4 \times t^4 + A_5 \times t^5 + A_6 \times t^6$

A_0	-2.52E-04
A_1	4.62E-06
A_2	1.93E-09
A_3	-6.22E-13
A_4	8.60E-17
A_5	-5.48E-21
A_6	1.31E-25

t (max) = 28 min

v (max) = 7.27×10^{-6} mol/Ls

4 mol% of $Pd(PPh_3)_2I_2$

Following *GP18* using 4-iodotoluene (**1a**) (545 mg, 2.50 mmol), phenylacetylene (**2a**) (0.302 mL, 2.75 mmol), pyrrolidine (0.420 mL, 5.00 mmol), bis(triphenylphosphine)palladium(II) iodide (89.5 mg, 0.10 mmol), dichloromethane (5 mL).

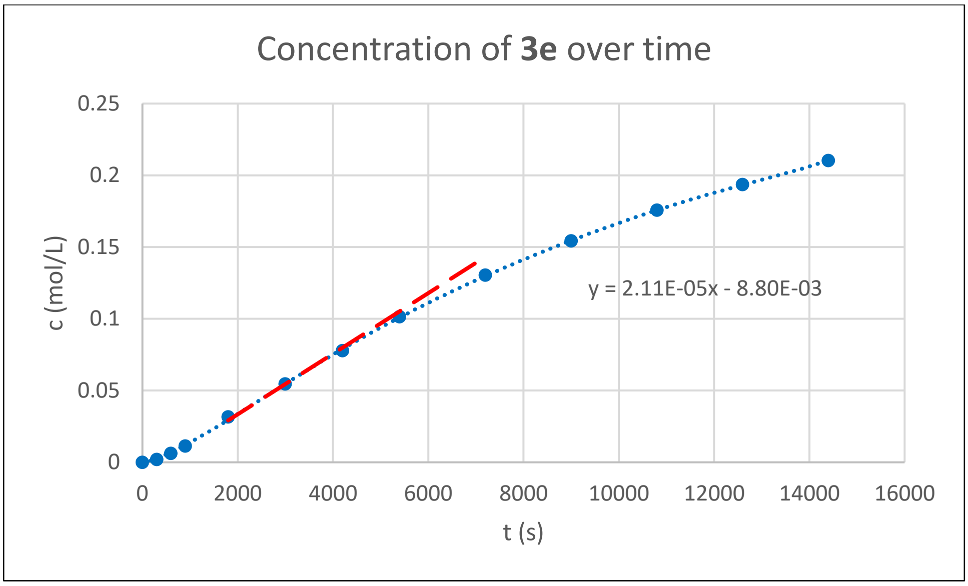

Fitted curve:

$c = A_0 + A_1 \times t + A_2 \times t^2 + A_3 \times t^3 + A_4 \times t^4 + A_5 \times t^5 + A_6 \times t^6$

A_0	-1.05E-03
A_1	1.08E-05
A_2	5.24E-09
A_3	-1.20E-12
A_4	1.26E-16
A_5	-6.57E-21
A_6	1.36E-25

t (max) = 42 min

v (max) = 2.11×10^{-5} mol/Ls

6 mol% of $Pd(PPh_3)_2I_2$

Following *GP18* using 4-iodotoluene (**1a**) (543.5 mg, 2.49 mmol), phenylacetylene (**2a**) (0.302 mL, 2.75 mmol), pyrrolidine (0.420 mL, 5.00 mmol), bis(triphenylphosphine)palladium(II) iodide (134.5 mg, 0.15 mmol), dichloromethane (5 mL).

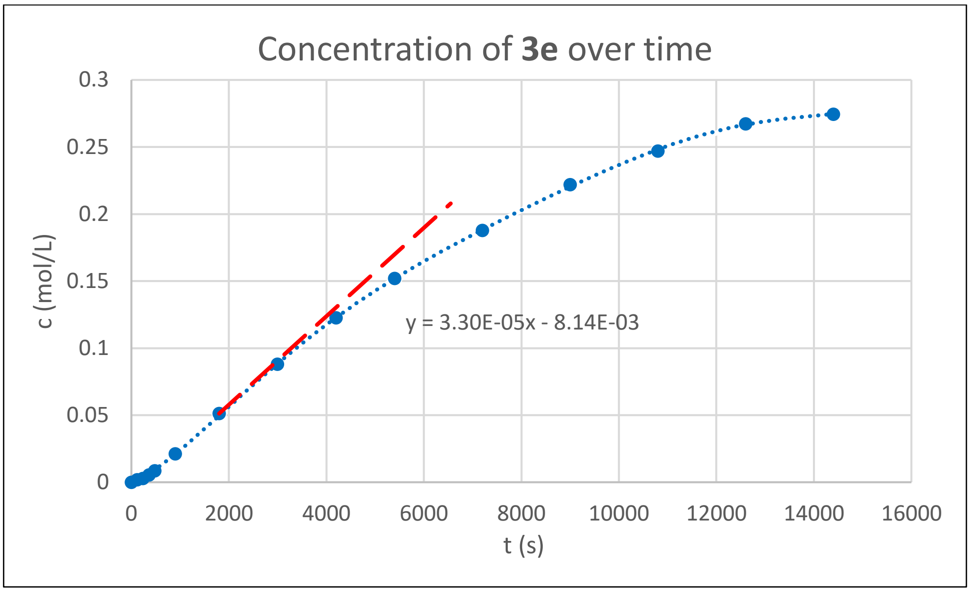

Fitted curve:

$$c = A_0 + A_1 \times t + A_2 \times t^2 + A_3 \times t^3 + A_4 \times t^4 + A_5 \times t^5 + A_6 \times t^6$$

A_0	-1.20E-03
A_1	1.83E-05
A_2	9.25E-09
A_3	-2.55E-12
A_4	2.96E-16
A_5	-1.62E-20
A_6	3.35E-25

t (max) = 32 min

v (max) = 3.30×10^{-5} mol/Ls

8 mol% of $Pd(PPh_3)_2I_2$

Following *GP18* using 4-iodotoluene (**1a**) (549 mg, 2.52 mmol), phenylacetylene (**2a**) (0.302 mL, 2.75 mmol), pyrrolidine (0.420 mL, 5.00 mmol), bis(triphenylphosphine)palladium(II) iodide (177 mg, 0.20 mmol), dichloromethane (5 mL).

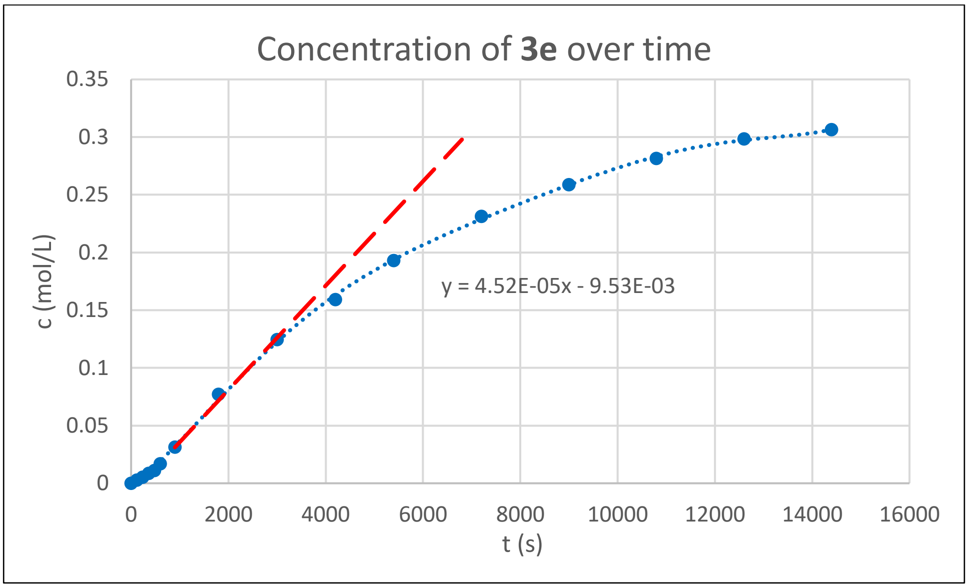

Fitted curve:

$c = A_0 + A_1 \times t + A_2 \times t^2 + A_3 \times t^3 + A_4 \times t^4 + A_5 \times t^5 + A_6 \times t^6$

A_0	-2.76E-03
A_1	3.14E-05
A_2	1.10E-08
A_3	-3.68E-12
A_4	4.61E-16
A_5	-2.65E-20
A_6	5.74E-25

t (max) = 24.5 min

v (max) = 4.52×10^{-5} mol/Ls

10 mol% of $Pd(PPh_3)_2I_2$

Following *GP18* using 4-iodotoluene (**1a**) (546 mg, 2.50 mmol), phenylacetylene (**2a**) (0.302 mL, 2.75 mmol), pyrrolidine (0.420 mL, 5.00 mmol), bis(triphenylphosphine)palladium(II) iodide (220 mg, 0.25 mmol), dichloromethane (5 mL).

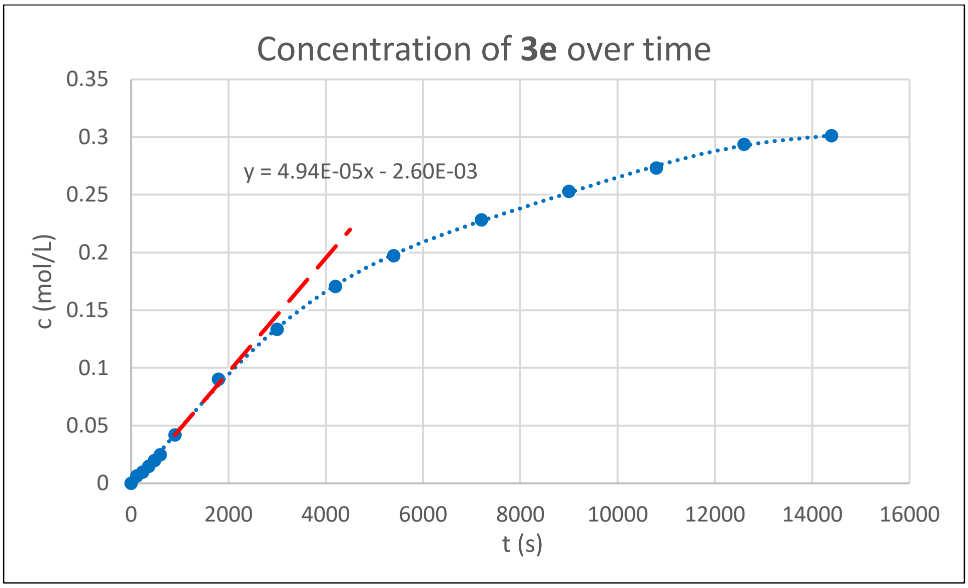

Fitted curve:

$$c = A_0 + A_1 \times t + A_2 \times t^2 + A_3 \times t^3 + A_4 \times t^4 + A_5 \times t^5 + A_6 \times t^6$$

A_0	-1.53E-03
A_1	4.67E-05
A_2	4.01E-09
A_3	-2.25E-12
A_4	2.98E-16
A_5	-1.67E-20
A_6	3.47E-25

t (max) = 12 min

v (max) = 4.94×10^{-5} mol/Ls

12 mol% of $Pd(PPh_3)_2I_2$

Following *GP18* using 4-iodotoluene (**1a**) (556 mg, 2.55 mmol), phenylacetylene (**2a**) (0.302 mL, 2.75 mmol), pyrrolidine (0.420 mL, 5.00 mmol), bis(triphenylphosphine)palladium(II) iodide (266 mg, 0.30 mmol), dichloromethane (5 mL).

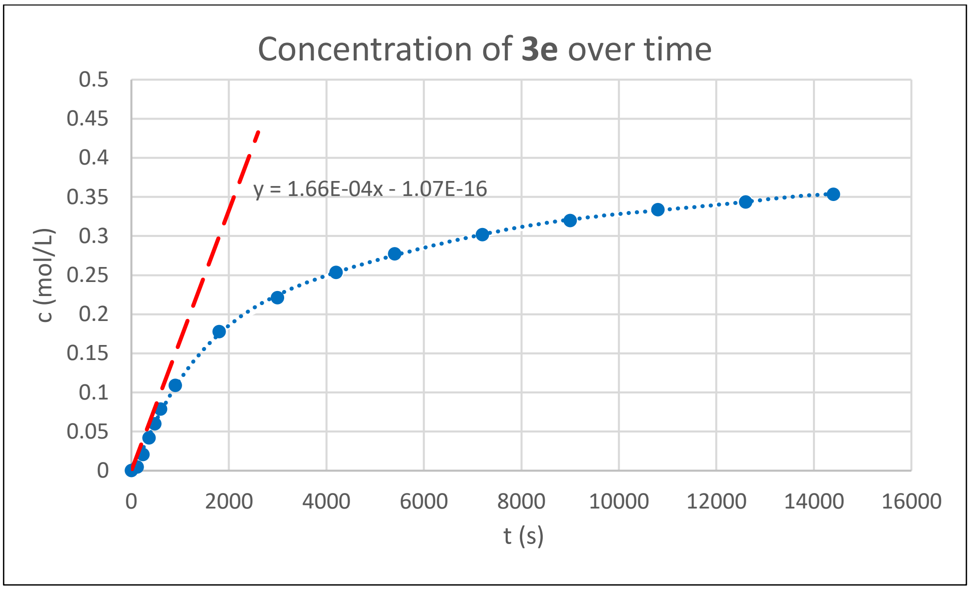

Fitted curve:

$$c = A_0 + A_1 \times t + A_2 \times t^2 + A_3 \times t^3 + A_4 \times t^4 + A_5 \times t^5 + A_6 \times t^6$$

A_0	-9.32E-03
A_1	1.66E-04
A_2	-4.82E-08
A_3	8.26E-12
A_4	-7.83E-16
A_5	3.80E-20
A_6	-7.33E-25

t (max) = 0 min

v (max) = 1.66×10^{-4} mol/Ls

15 mol% of $Pd(PPh_3)_2I_2$

Following *GP18* using 4-iodotoluene (**1a**) (547 mg, 2.51 mmol), phenylacetylene (**2a**) (0.302 mL, 2.75 mmol), pyrrolidine (0.420 mL, 5.00 mmol), bis(triphenylphosphine)palladium(II) iodide (331 mg, 0.374 mmol), dichloromethane (5 mL).

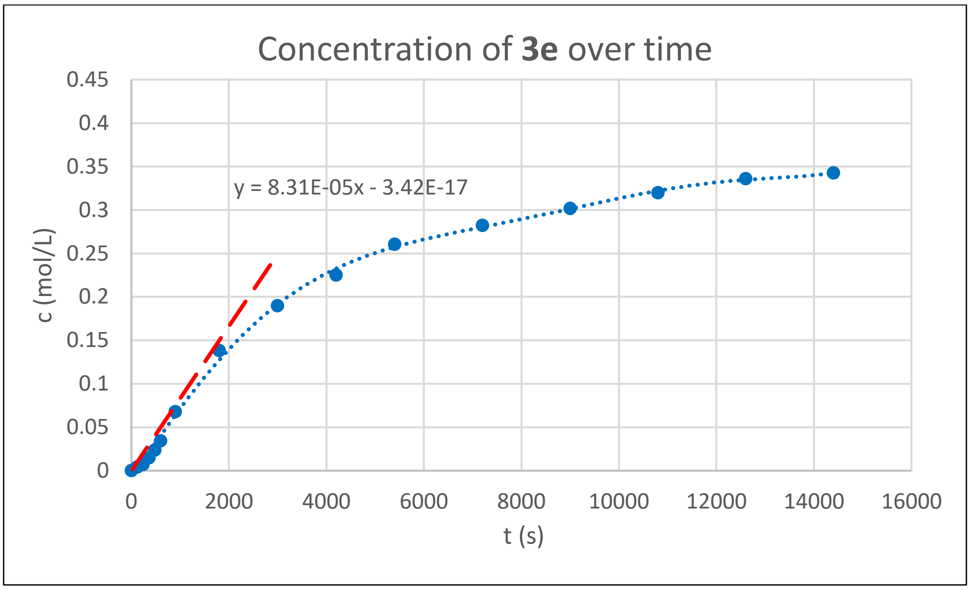

Fitted curve:

$$c = A_0 + A_1 \times t + A_2 \times t^2 + A_3 \times t^3 + A_4 \times t^4 + A_5 \times t^5 + A_6 \times t^6$$

A_0	-8.89E-03
A_1	8.31E-05
A_2	-3.83E-10
A_3	-2.83E-12
A_4	4.58E-16
A_5	-2.88E-20
A_6	6.46E-25

t (max) = 0 min

v (max) = 8.31×10^{-4} mol/Ls

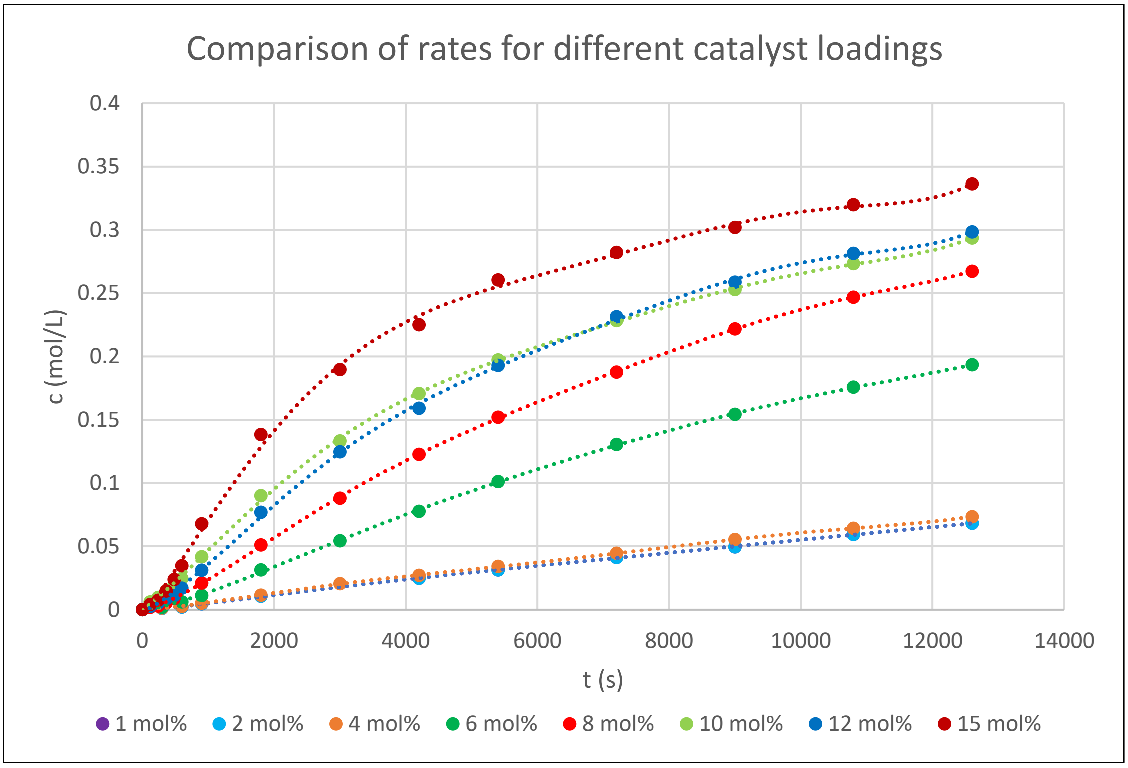

Supplementary Figure 33. Comparison of concentration-time plots for different catalyst loadings.

Reaction rates in respect to concentration are collected in the table below:

**c (cat.)**	**rate**	**ln (c (cat.))**	**ln (v)**
5.108E-03	7.645E-06	-5.27687	-11.78148
1.010E-02	7.560E-06	-4.59484	-11.79264
2.023E-02	2.084E-05	-3.90057	-10.77869
3.040E-02	3.208E-05	-3.49325	-10.34739
4.001E-02	4.913E-05	-3.21866	-9.92114
4.973E-02	5.543E-05	-3.00119	-9.80032
7.482E-02	9.991E-05	-2.59270	-9.21122

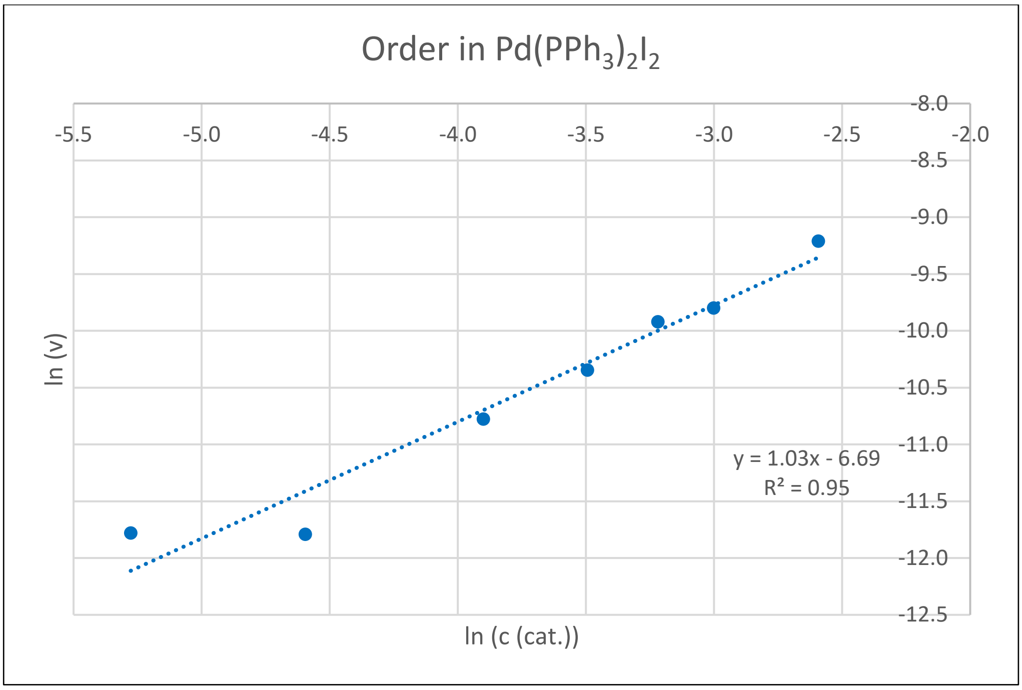

Supplementary Figure 34. Order in $Pd(PPh_3)_2I_2$ is 1.03.

Order in 4-iodotoluene

Order in 4-iodotoluene (**1a**) was determined by measuring the reaction rates at different concentrations of **1a**. Reactions were performed according to *GP18* in CH_2Cl_2 at room temperature (296 K) and at 4 mol% $Pd(PPh_3)_2I_2$ loading.

Reaction at 0.5 M concentration of 1a - for analysis of the reaction please see S184.

Reaction at 0.25 M concentration of 1a

Following *GP18* using 4-iodotoluene (**1a**) (272 mg, 1.25 mmol), phenylacetylene (**2a**) (0.302 mL, 2.75 mmol), pyrrolidine (0.420 mL, 5.00 mmol), bis(triphenylphosphine)palladium(II) iodide (88.4 mg, 0.100 mmol), dichloromethane (5 mL).

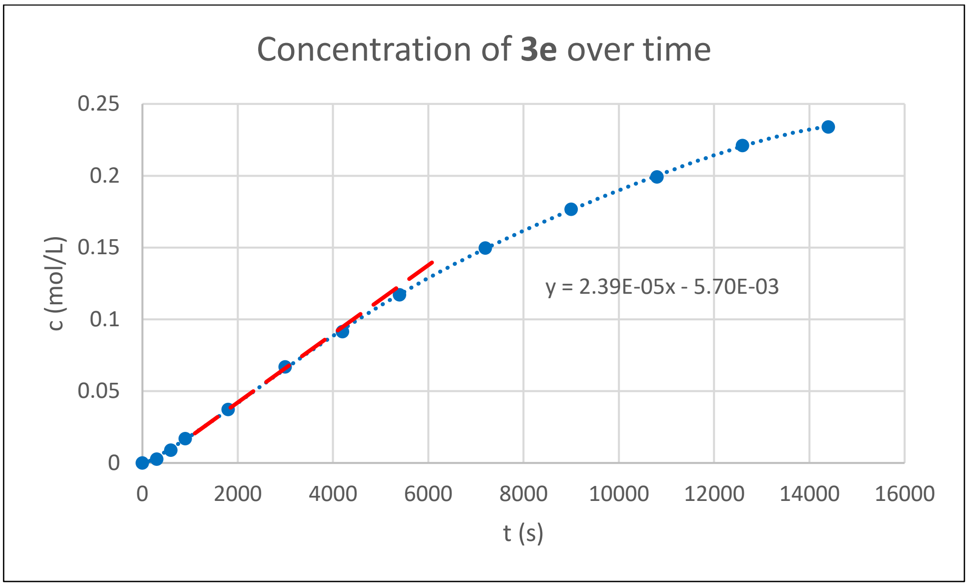

Fitted curve:

$$c = A_0 + A_1 \times t + A_2 \times t^2 + A_3 \times t^3 + A_4 \times t^4 + A_5 \times t^5 + A_6 \times t^6$$

A_0	-1.38E-03
A_1	1.73E-05
A_2	3.43E-09
A_3	-7.52E-13
A_4	6.36E-17
A_5	-2.39E-21
A_6	2.97E-26

t (max) = 38 min

v (max) = 2.39×10^{-5} mol/Ls

Reaction at 1.0 M concentration of 1a

Following *GP18* using 4-iodotoluene (**1a**) (1090 mg, 5.00 mmol), phenylacetylene (**2a**) (0.302 mL, 2.75 mmol), pyrrolidine (0.420 mL, 5.00 mmol), bis(triphenylphosphine)palladium(II) iodide (85.8 mg, 0.097 mmol), dichloromethane (5 mL).

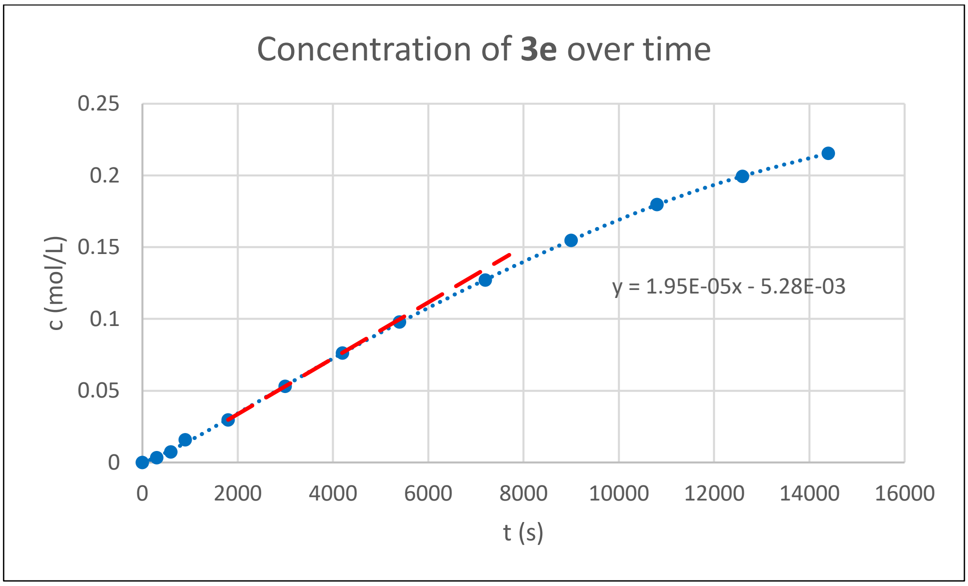

Fitted curve:

$c = A_0 + A_1 \times t + A_2 \times t^2 + A_3 \times t^3 + A_4 \times t^4 + A_5 \times t^5 + A_6 \times t^6$

A_0	-3.54E-04
A_1	1.31E-05
A_2	3.35E-09
A_3	-8.02E-13
A_4	8.90E-17
A_5	-4.87E-21
A_6	1.03E-26

t (max) = 41 min

v (max) = 1.95×10^{-5} mol/Ls

Reaction rates in respect to concentration are collected in the table below:

c (1a)	rate	ln (c (1a))	ln (v)
0.2559281	2.390E-05	-1.3628587	-10.641624
0.4999312	2.111E-05	-0.6932848	-10.765629
0.9998624	1.947E-05	-0.0001376	-10.846762

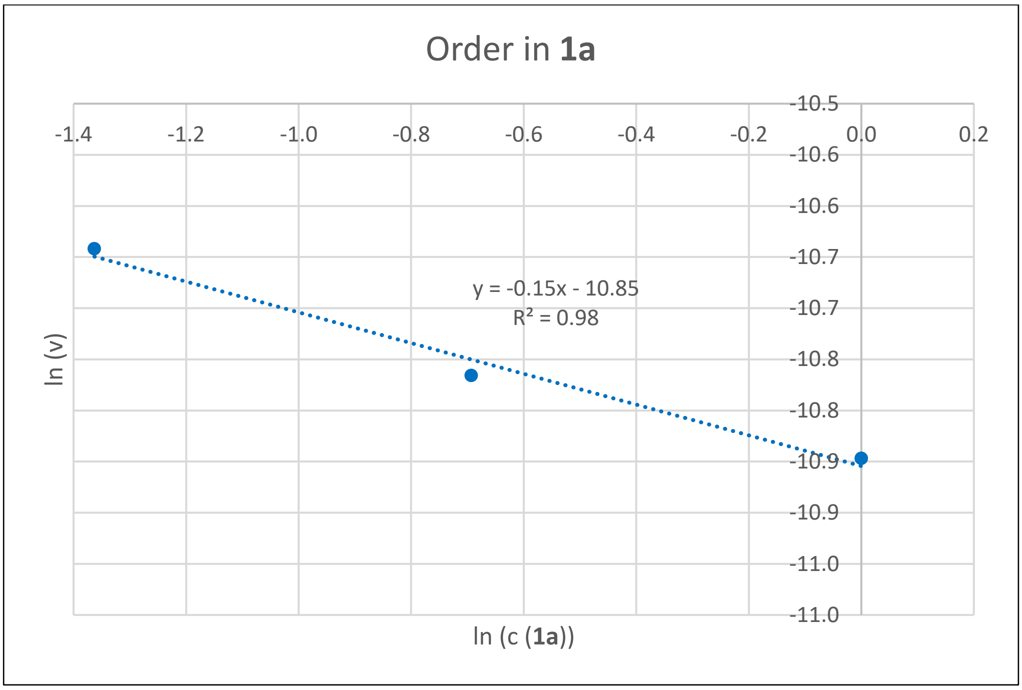

Supplementary Figure 35. Order in 4-iodotoluene is -0.15.

Order in phenylacetylene

Order in phenylacetylene (**2a**) was determined by measuring the reaction rates at different concentrations of **2a**. Reactions were performed according to *GP18* in CH_2Cl_2 at room temperature (296 K) and at 4 mol% $Pd(PPh_3)_2I_2$ loading.

Reaction at 0.55 M concentration of 2a - for analysis of the reaction please see S184.

Reaction at 0.275 M concentration of 2a

Following *GP18* using 4-iodotoluene (**1a**) (550 mg, 2.52 mmol), phenylacetylene (**2a**) (0.151 mL, 1.375 mmol), pyrrolidine (0.420 mL, 5.00 mmol), bis(triphenylphosphine)palladium(II) iodide (88.8 mg, 0.100 mmol), dichloromethane (5 mL).

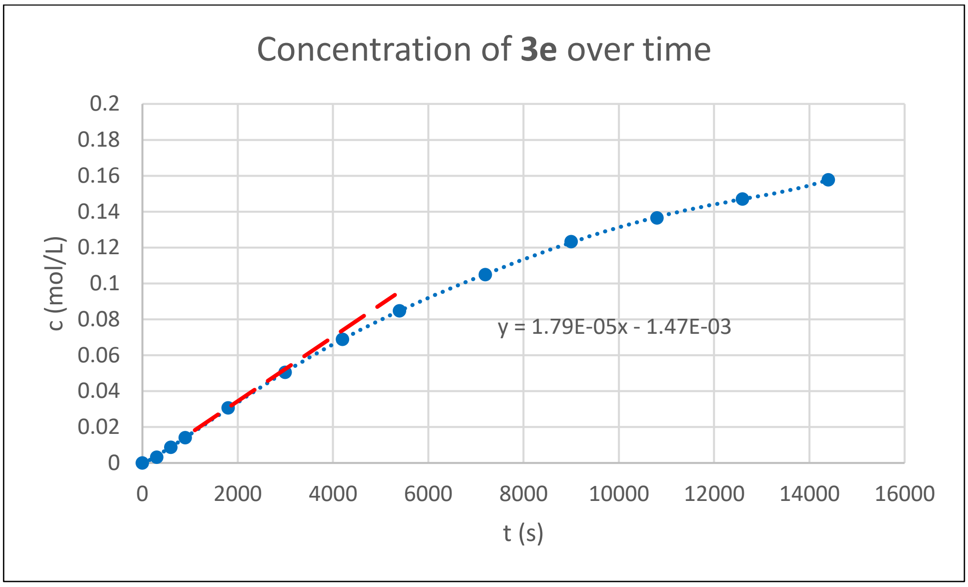

Fitted curve:

$c = A_0 + A_1 \times t + A_2 \times t^2 + A_3 \times t^3 + A_4 \times t^4 + A_5 \times t^5 + A_6 \times t^6$

A_0	-5.71E-04
A_1	1.46E-05
A_2	2.68E-09
A_3	-9.03E-13
A_4	1.16E-16
A_5	-7.04E-21
A_6	1.63E-25

t (max) = 24 min

v (max) = 1.79×10^{-5} mol/Ls

Reaction at 1.10 M concentration of 2a

Following *GP18* using 4-iodotoluene (**1a**) (549 mg, 2.52 mmol), phenylacetylene (**2a**) (0.604 mL, 5.50 mmol), pyrrolidine (0.420 mL, 5.00 mmol), bis(triphenylphosphine)palladium(II) iodide (89.2 mg, 0.101 mmol), dichloromethane (5 mL).

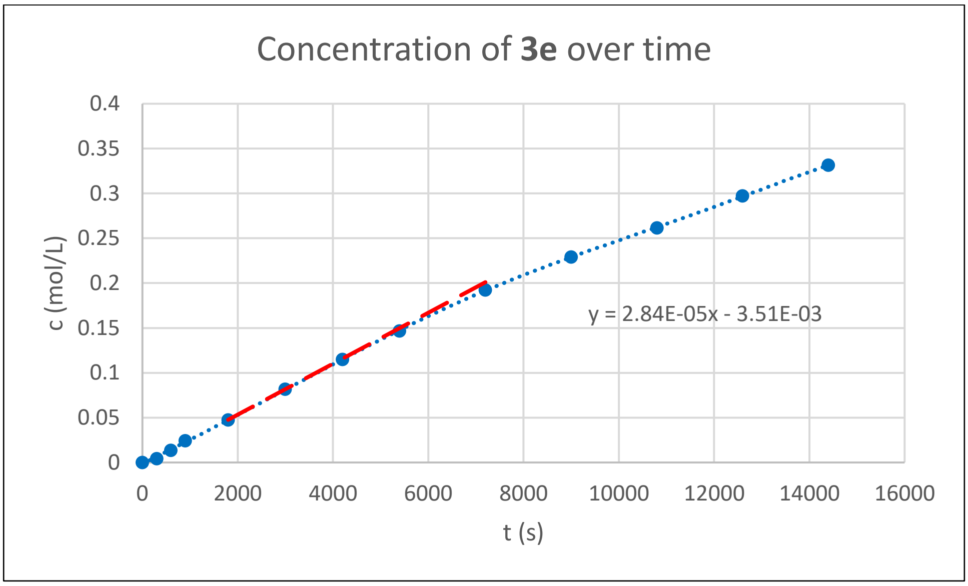

Fitted curve:

$c = A_0 + A_1 \times t + A_2 \times t^2 + A_3 \times t^3 + A_4 \times t^4 + A_5 \times t^5 + A_6 \times t^6$

A_0	-1.67E-03
A_1	2.72E-05
A_2	-4.47E-10
A_3	3.90E-13
A_4	-8.18E-17
A_5	5.97E-21
A_6	-1.47E-25

t (max) = 53 min

v (max) = 2.84×10^{-5} mol/Ls

Reaction rates in respect to concentration are collected in the table below:

c (PhCCH)	rate	ln (c (PhCCH))	ln (v)
0.27499	1.794E-05	-1.2910205	-10.928516
0.549989	2.111E-05	-0.5978570	-10.765629
1.0999775	2.840E-05	0.0952897	-10.469126

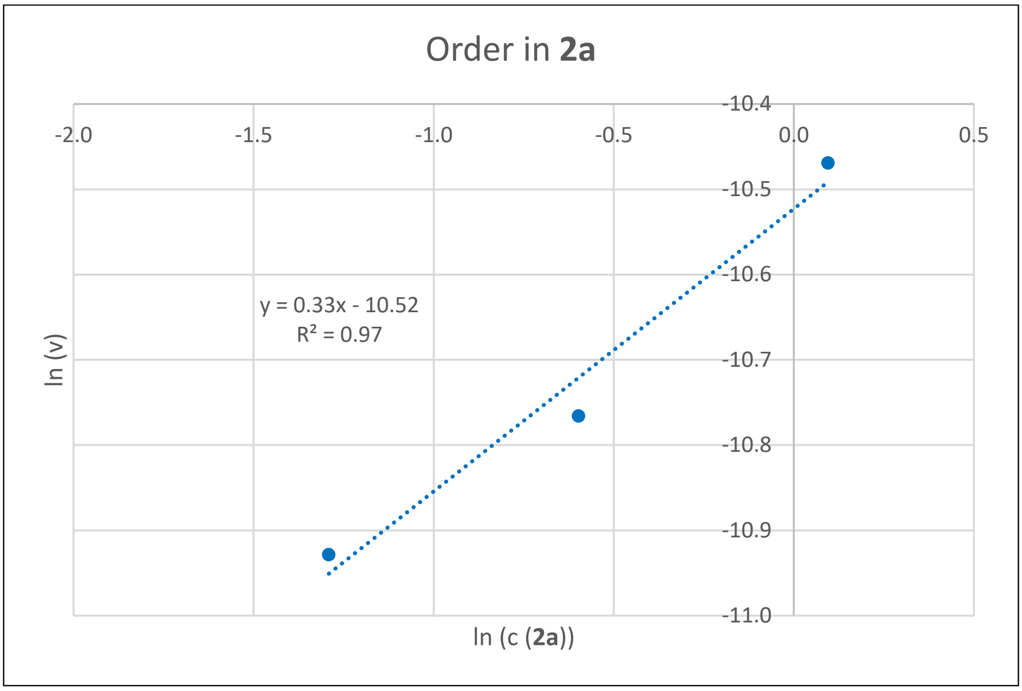

Supplementary Figure 36. Order in phenylacetylene is 0.33.

Order in pyrrolidine

Order in pyrrolidine was determined by measuring the reaction rates at different concentrations. Reactions were performed according to *GP18* in CH_2Cl_2 at room temperature (296 K) and at 4 mol% $Pd(PPh_3)_2I_2$ loading.

Reaction at 1.0 M concentration of pyrrolidine - for analysis of the reaction please see S184.

Reaction at 0.50 M concentration of pyrrolidine

Following *GP18* using 4-iodotoluene (**1a**) (546 mg, 2.50 mmol), phenylacetylene (**2a**) (0.302 mL, 2.75 mmol), pyrrolidine (0.210 mL, 2.50 mmol), bis(triphenylphosphine)palladium(II) iodide (89.8 mg, 0.101 mmol), dichloromethane (5 mL).

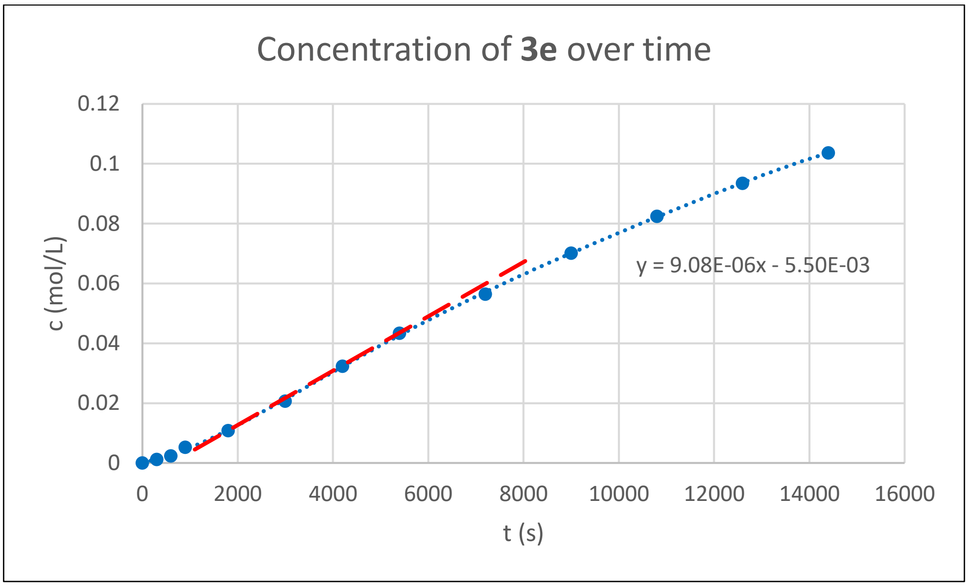

Fitted curve:

$c = A_0 + A_1 \times t + A_2 \times t^2 + A_3 \times t^3 + A_4 \times t^4 + A_5 \times t^5 + A_6 \times t^6$

A_0	1.11E-04
A_1	3.19E-06
A_2	2.14E-09
A_3	-3.51E-13
A_4	2.60E-17
A_5	-8.79E-22
A_6	9.83E-27

t (max) = 59 min

v (max) = 9.08×10^{-6} mol/Ls

Reaction at 2.0 M concentration of pyrrolidine

Following *GP18* using 4-iodotoluene (**1a**) (547 mg, 2.51 mmol), phenylacetylene (**2a**) (0.302 mL, 2.75 mmol), pyrrolidine (0.840 mL, 10.0 mmol), bis(triphenylphosphine)palladium(II) iodide (88.2 mg, 0.100 mmol), dichloromethane (5 mL).

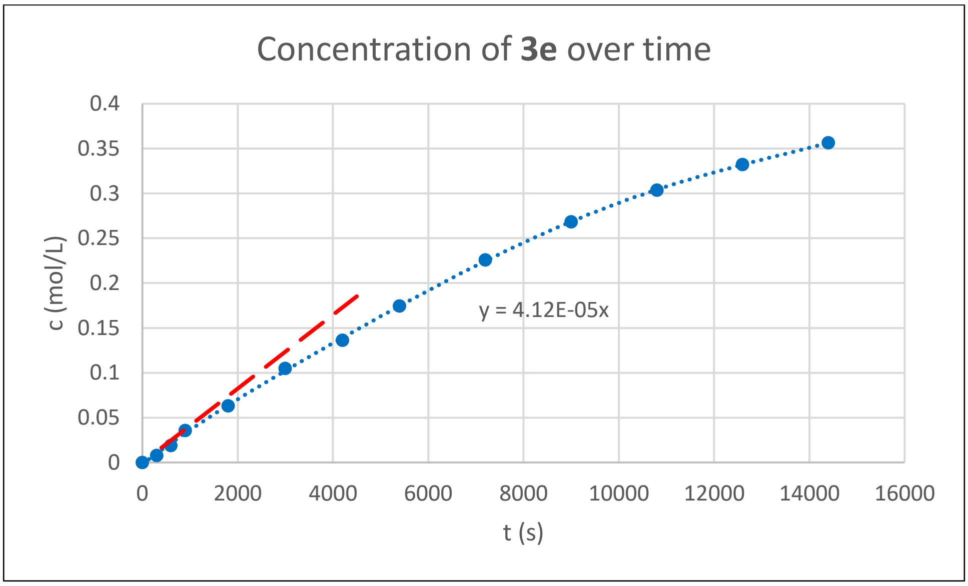

Fitted curve:

$c = A_0 + A_1 \times t + A_2 \times t^2 + A_3 \times t^3 + A_4 \times t^4 + A_5 \times t^5 + A_6 \times t^6$

A_0	-2.02E-03
A_1	4.12E-05
A_2	-3.49E-09
A_3	6.06E-13
A_4	-5.77E-17
A_5	2.29E-21
A_6	-2.87E-26

t (max) = 0 min

v (max) = 4.12×10^{-5} mol/Ls

Reaction rates in respect to concentration are collected in the table below:

c (pyrr.)	rate	ln (c (pyrr.))	ln (v)
0.507874	9.081E-06	-0.6775219	-11.609343
1.015748	2.111E-05	0.0156253	-10.765629
2.031496	4.116E-05	0.7087725	-10.098121

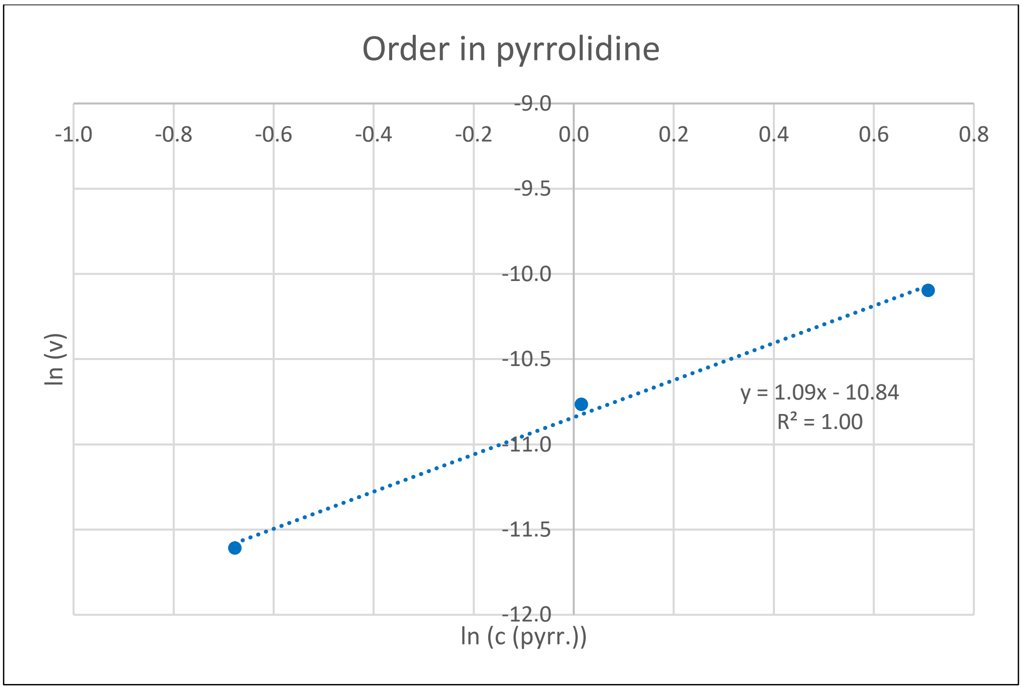

Supplementary Figure 37. Order in pyrrolidine is 1.09.

Variable time normalization analyses of the catalyzed reactions

Variable time normalization analyses for reactions with different concentrations of starting materials (vide supra) were performed according to literature.[43]

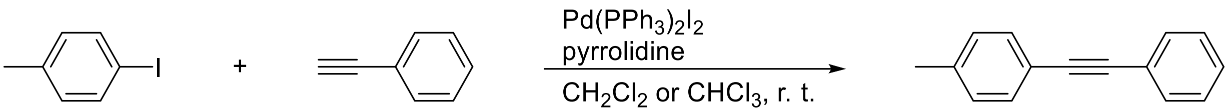

1a **2a** **3e**

Order in $Pd(PPh_3)_2I_2$

Comparison of reactions with 4 mol% and 8 mol% loading of $Pd(PPh_3)_2I_2$.

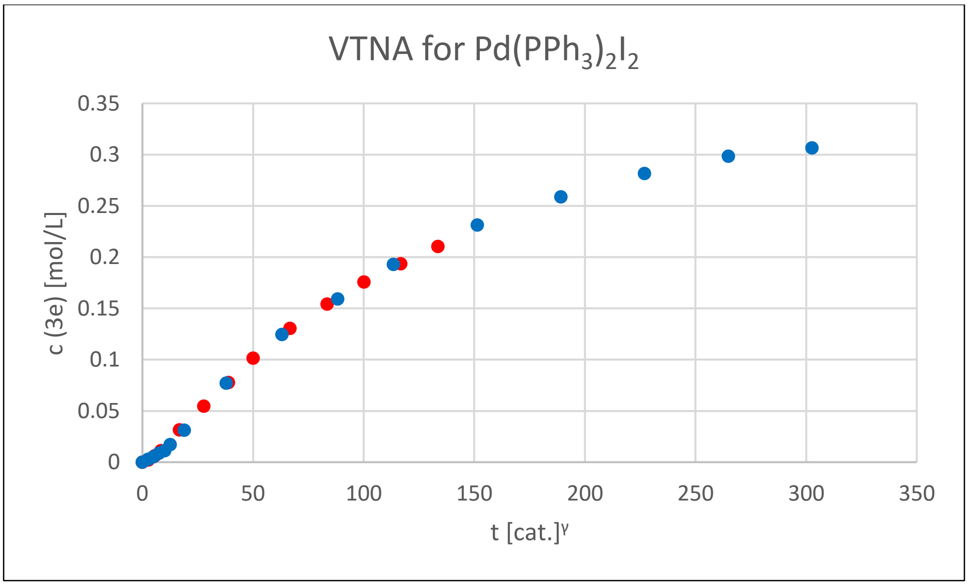

Supplementary Figure 38. VTNA analysis reveals that the order in $Pd(PPh_3)_2I_2$ is 1.2.

Calculation for the reaction with 4 mol% $Pd(PPh_3)_2I_2$ (γ = 1.2).

t	c (cat.)	$[cat.]^{\gamma}$ t	c (3e)
0	0.02023027	0	0
300	0.02023027	2.781783133	0.001955828
600	0.02023027	5.563566267	0.006186602
900	0.02023027	8.3453494	0.011273419
1800	0.02023027	16.6906988	0.031523774
3000	0.02023027	27.81783133	0.054536565
4200	0.02023027	38.94496387	0.077742687
5400	0.02023027	50.0720964	0.101278207
7200	0.02023027	66.7627952	0.130382158
9000	0.02023027	83.453494	0.154220645
10800	0.02023027	100.1441928	0.175634904
12600	0.02023027	116.8348916	0.193429217
14400	0.02023027	133.5255904	0.210259321

Calculation for the reaction with 8 mol% $Pd(PPh_3)_2I_2$ (γ = 1.2).

t	c (cat.)	$[cat.]^{\gamma}$ t	c (3e)
0	0.0400085	0	0
120	0.0400085	2.52210968	0.002702717
240	0.0400085	5.04421936	0.005088403
360	0.0400085	7.56632904	0.008630254
480	0.0400085	10.08843872	0.011151746
600	0.0400085	12.6105484	0.016953923
900	0.0400085	18.9158226	0.031153578
1800	0.0400085	37.8316452	0.07697986
3000	0.0400085	63.052742	0.124569573
4200	0.0400085	88.2738388	0.159024731
5400	0.0400085	113.4949356	0.192923948
7200	0.0400085	151.3265808	0.231410131
9000	0.0400085	189.158226	0.258627385
10800	0.0400085	226.9898712	0.281460473
12600	0.0400085	264.8215164	0.298411898
14400	0.0400085	302.6531616	0.306452828

Order in 4-iodotoluene

Comparison of reactions with 0.25 M and 1.0 M concentration of 4-iodotoluene.

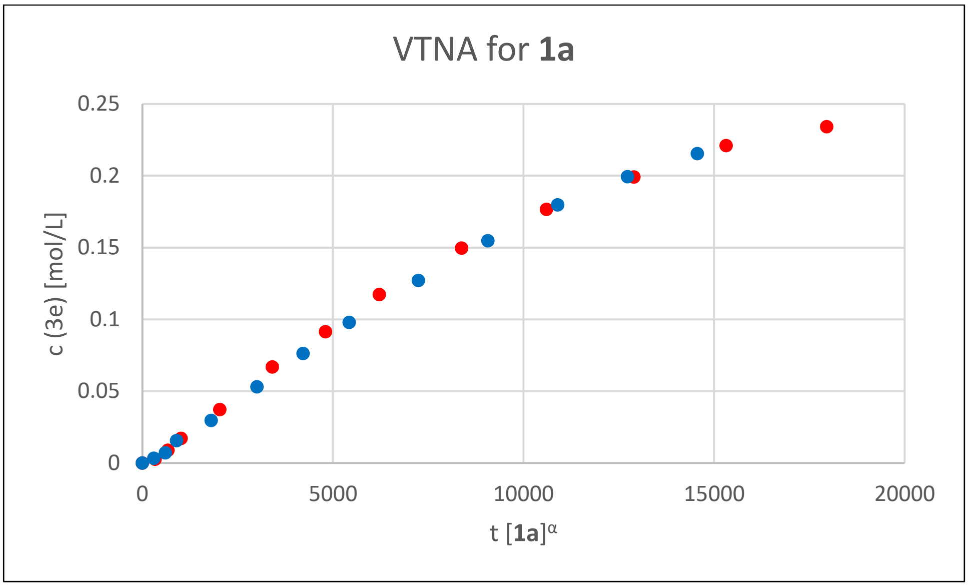

Supplementary Figure 39. VTNA analysis reveals that the order in 4-iodotoluene is -0.08.

Calculation for the reaction with 0.25 M of 4-iodotoluene (α = -0.08).

t	c (1a)	$[1a]^{\alpha}$ t	c (3e)
0	0.2495100	0	0
300	0.2306822	336.2717989	0.00264383
600	0.2272388	673.8235855	0.00898947
900	0.2173079	1012.176764	0.01711545
1800	0.1970444	2032.964214	0.03731750
3000	0.1665809	3408.308158	0.06696982
4200	0.1385808	4803.073828	0.09140853
5400	0.1166178	6217.933566	0.11725998
7200	0.0856403	8380.066748	0.14970334
9000	0.0584292	10601.68353	0.17667108
10800	0.0359201	12899.82004	0.19918426
12600	0.0143492	15316.67577	0.22101021
14400	0.0021396	17958.96198	0.23414877

Calculation for the reaction with 0.50 M of 4-iodotoluene (α = -0.08).

t	c (1a)	$[1a]^{\alpha}$ t	c (3e)
0	0.9998600	0	0
300	0.9934859	300.0799927	0.0033095
600	0.9875998	600.308131	0.0072229
900	0.9751465	900.7597319	0.0157473
1800	0.9632529	1803.015045	0.0296840
3000	0.9409425	3007.73672	0.0531254
4200	0.9107602	4215.155551	0.0762904
5400	0.8905241	5425.243856	0.0979799
7200	0.8615445	7244.403513	0.1270881
9000	0.8342918	9068.31682	0.1547556
10800	0.8122292	10896.54131	0.1798308
12600	0.7927357	12728.50838	0.1994312
14400	0.7799350	14563.45689	0.2154786

Order in phenylacetylene

Comparison of reactions with 0.275 M and 1.1 M concentration of phenylacetylene.

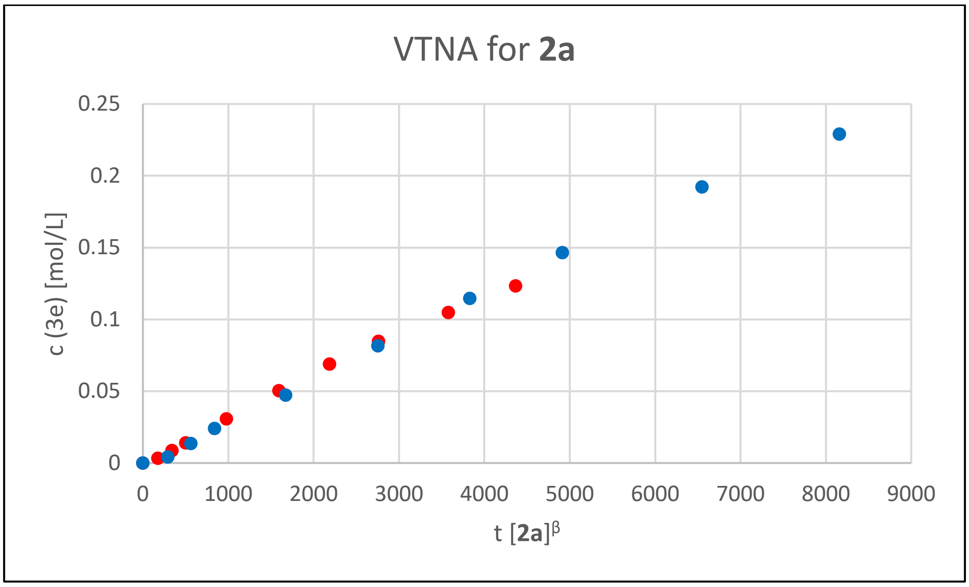

Supplementary Figure 40. VTNA analysis reveals that the order in phenylacetylene is 0.37.

Calculation for the reaction with 0.275 M of phenylacetylene (β = 0.37).

**t**	**c (2a)**	**$[2a]^{\beta}$ t**	**c (3e)**
0	0.2750000	0	0
300	0.1995316	176.1806718	0.003339927
600	0.1922883	340.3084802	0.008711873
900	0.1834567	501.9119392	0.014199453
1800	0.1756742	978.6775759	0.030818317
3000	0.1537650	1594.388742	0.050512279
4200	0.1427871	2186.601182	0.068884475
5400	0.1274631	2758.808714	0.084808893
7200	0.1112226	3578.56953	0.104961705
9000	0.1028496	4365.975395	0.123279549

Calculation for the reaction with 1.1 M of phenylacetylene (β = 0.37).

**t**	**c (2a)**	**$[2a]^{\beta}$ t**	**c (3e)**
0	1.1000000	0	0
300	0.7674812	292.4859201	0.004193476
600	0.7415705	562.7950285	0.013587828
900	0.8474240	838.3164926	0.024044130
1800	0.7818061	1672.563981	0.047374853
3000	0.7308198	2754.747245	0.081688158
4200	0.7490066	3828.188121	0.114772061
5400	0.7738710	4913.079354	0.146610238
7200	0.7731627	6549.919732	0.192345886
9000	0.6995588	8157.216411	0.229195130

Order in pyrrolidine

Comparison of reactions with 0.50 M and 2.0 M concentration of pyrrolidine.

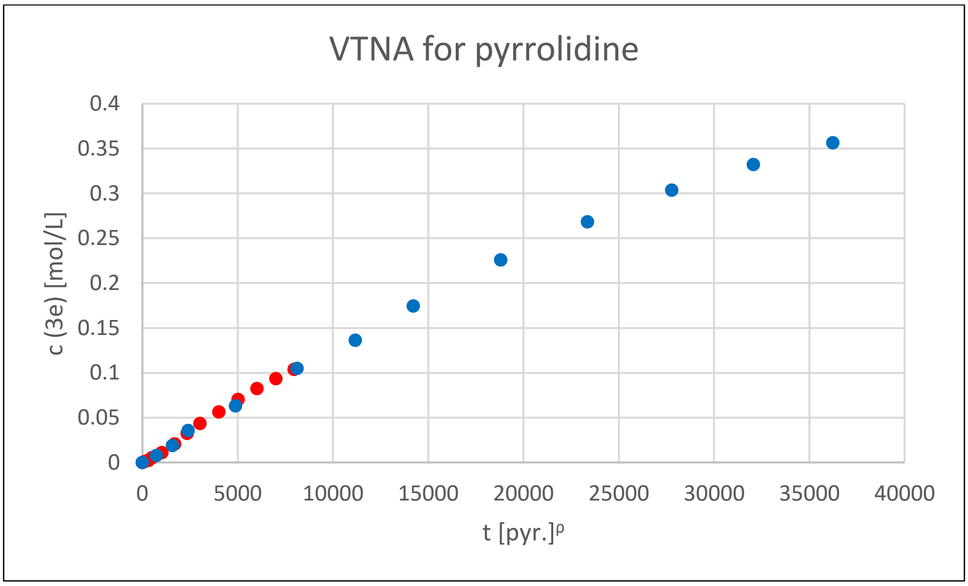

Supplementary Figure 41. VTNA analysis reveals that the order in pyrrolidine is 1.1.

Calculation for the reaction with 0.50 M of pyrrolidine (ρ = 1.10).

t	c (pyr.)	[pyr.]$^{\rho}$ t	c (3e)
0	0.5080000	0	0
300	0.6066094	157.69693	0.00125282
600	0.6044101	330.4614367	0.00235448
900	0.5931360	501.1127947	0.00529178
1800	0.6127832	1017.005725	0.01085000
3000	0.5711758	1691.096852	0.02065164
4200	0.5941561	2353.531227	0.03240219
5400	0.5725843	3016.846355	0.04344194
7200	0.5923624	4010.136554	0.05643617
9000	0.5889638	5018.80003	0.07020162
10800	0.5855531	6021.069929	0.08243176
12600	0.5681456	7003.815592	0.09348912
14400	0.5524443	7955.583121	0.10365642

Calculation for the reaction with 2.00 M of pyrrolidine (ρ = 1.10).

t	c (pyr.)	[pyr.]$^{\rho}$ t	c (3e)
0	2.0310000	0	0
300	2.5059626	738.6351713	0.00793506
600	2.5532250	1571.313246	0.01872618
900	2.5238729	2407.234476	0.03551165
1800	2.5076422	4890.242781	0.06307953
3000	2.3953271	8108.000514	0.10470643
4200	2.3072086	11181.36139	0.13638290
5400	2.3423473	14216.65614	0.17450574
7200	2.3461051	18811.51289	0.22580092
9000	2.2956047	23356.0044	0.26819305
10800	2.2319573	27777.71541	0.30378648
12600	2.1736230	32068.56149	0.33235710
14400	2.1182803	36237.77784	0.35635405

Accelerating catalytic reaction by in situ halide metathesis

Reactions were performed at room temperature (296 K) in an oven-dried round-bottom flasks that were cooled under argon atmosphere prior to use. Solvent (DCM) was distilled over CaH_2 before use. The reactions were performed under atmosphere and monitored by taking aliquots samples (50 μL) from reaction mixture that were transferred directly into NMR tube filled with degassed $CDCl_3$ under argon atmosphere. It was demonstrated that this work-up (dilution) stops the reaction.[17] Concentration of product (**3e**) was determined by comparison of integral of characteristic proton resonances for Me group of product **3e** (δ 2.36 ppm, 3H, Me) with an integrnals of resonances of an internal standard (1,3,5-trimethoxybenzene, δ 6.08 ppm, 3H, ArH, and δ 3.75 ppm, 9H, OMe). Reaction rates were estimated as derivative of the fitted polynomial function.

General procedure 19 (*GP19*) – Catalytic reaction between phenylacetylene (2a) and 4-iodotoluene (1a), catalyzed by $Pd(PPh_3)_2I_2$ or $Pd(PPh_3)_2Cl_2$ and addition of tetrabutylammonium chloride.

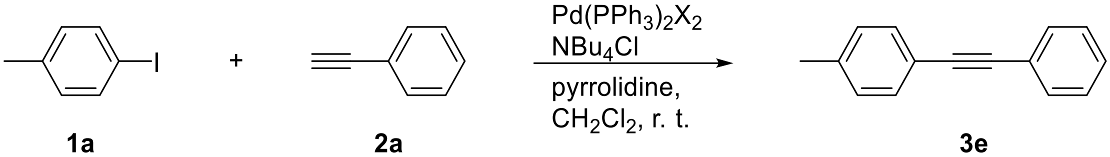

1a **2a** **3e**

An oven-dried round-bottom reaction flask was fitted with a stirring bar and sealed with a rubber septum, which was pierced with a needle connected by a tube to a Schlenk vacuum manifold. The flask was cooled to room temperature under argon atmosphere. The flask was charged with 4-iodotoluene (**1a**) (1 equiv.), phenylacetylene (**2a**) (1.1 equiv.), tetrabutylammonium chloride (0.2 or 1.1 equiv.), and pyrrolidine (2 equiv.) by rapidly opening the septum and flushing with argon. Dichloromethane (2 mL/mmol to **1a**) was added with a syringe by piercing the septum. To this solution bis(triphenylphosphine)palladium(II) halide ($Pd(PPh_3)_2I_2$ or $Pd(PPh_3)_2Cl_2$, 0.1 equiv.) was added by rapidly opening the septum and flushing with argon. Samples (50 μL) were taken with a syringe in intervals (15 min) from the solution and transferred to dry NMR tube filled with degassed $CDCl_3$ under argon atmosphere.

Reaction catalyzed with $Pd(PPh_3)_2I_2$

Following *GP19* using 4-iodotoluene (**1a**) (545 mg, 2.50 mmol), phenylacetylene (**2a**) (0.302 mL, 2.75 mmol), pyrrolidine (0.420 mL, 5.00 mmol), bis(triphenylphosphine)palladium(II) iodide (221 mg, 0.25 mmol), dichloromethane (5 mL).

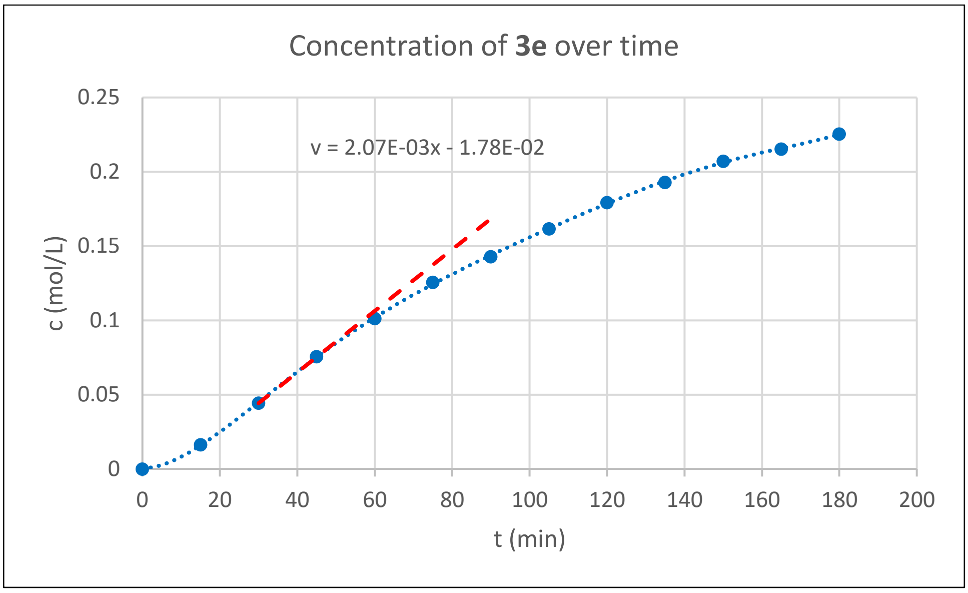

Fitted curve:

$c = A_0 + A_1 \times t + A_2 \times t^2 + A_3 \times t^3 + A_4 \times t^4 + A_5 \times t^5 + A_6 \times t^6$

A_0	1.13E-04
A_1	1.46E-04
A_2	7.97E-05
A_3	-1.51E-06
A_4	1.32E-08
A_5	-5.60E-11
A_6	9.21E-14

t (max) = 31 min

v (max) = 2.07×10^{-3} mol/Lmin

Reaction catalyzed with $Pd(PPh_3)_2Cl_2$

Following *GP19* using 4-iodotoluene (**1a**) (546 mg, 2.50 mmol), phenylacetylene (**2a**) (0.302 mL, 2.75 mmol), pyrrolidine (0.420 mL, 5.00 mmol), bis(triphenylphosphine)palladium(II) chloride (176 mg, 0.25 mmol), dichloromethane (5 mL).

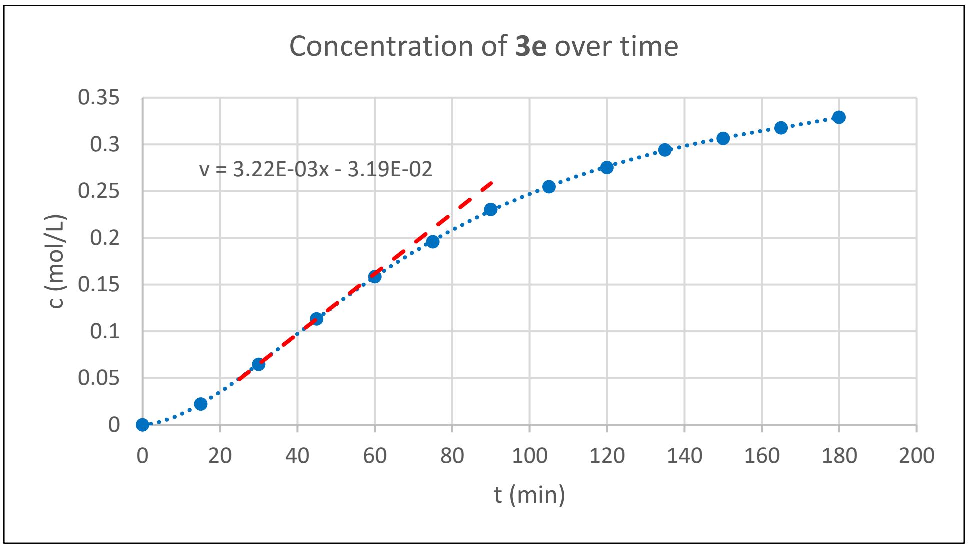

Fitted curve:

$c = A_0 + A_1 \times t + A_2 \times t^2 + A_3 \times t^3 + A_4 \times t^4 + A_5 \times t^5 + A_6 \times t^6$

A_0	2.35E-05
A_1	2.74E-04
A_2	1.02E-04
A_3	-1.60E-06
A_4	1.17E-08
A_5	-4.30E-11
A_6	6.45E-14

t (max) = 37 min

v (max) = 3.22×10^{-3} mol/Lmin

Reaction catalyzed with $Pd(PPh_3)_2I_2$ with addition of 0.2 equiv. of tetrabutylammonium chloride

Following *GP19* using 4-iodotoluene (**1a**) (546 mg, 2.50 mmol), phenylacetylene (**2a**) (0.302 mL, 2.75 mmol), pyrrolidine (0.420 mL, 5.00 mmol), bis(triphenylphosphine)palladium(II) iodide (221 mg, 0.25 mmol), tetrabutylammonium chloride (132 mg, 0.48 mmol), dichloromethane (5 mL).

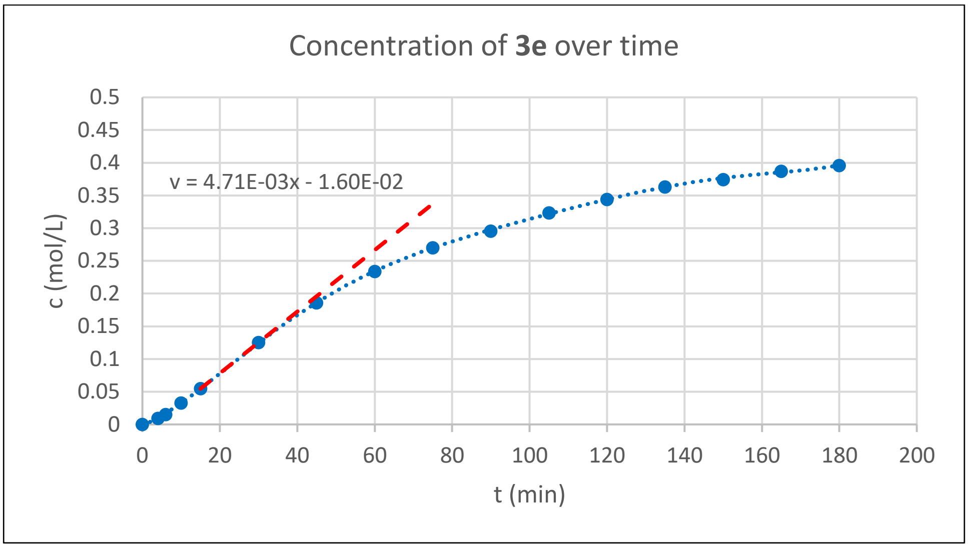

Fitted curve:

$$c = A_0 + A_1 \times t + A_2 \times t^2 + A_3 \times t^3 + A_4 \times t^4 + A_5 \times t^5 + A_6 \times t^6$$

A_0	-1.32E-03
A_1	2.41E-03
A_2	1.26E-04
A_3	-2.95E-06
A_4	2.79E-08
A_5	-1.23E-10
A_6	2.08E-13

t (max) = 22 min

v (max) = 4.71×10^{-3} mol/Lmin

Reaction catalyzed with $Pd(PPh_3)_2I_2$ with addition of 1.1 equiv. of tetrabutylammonium chloride

Following *GP19* using 4-iodotoluene (**1a**) (548 mg, 2.51 mmol), phenylacetylene (**2a**) (0.302 mL, 2.75 mmol), pyrrolidine (0.420 mL, 5.00 mmol), bis(triphenylphosphine)palladium(II) iodide (221 mg, 0.25 mmol, 0.05 M, 10 mol%), tetrabutylammonium chloride (764 mg, 2.75 mmol), dichloromethane (5 mL).

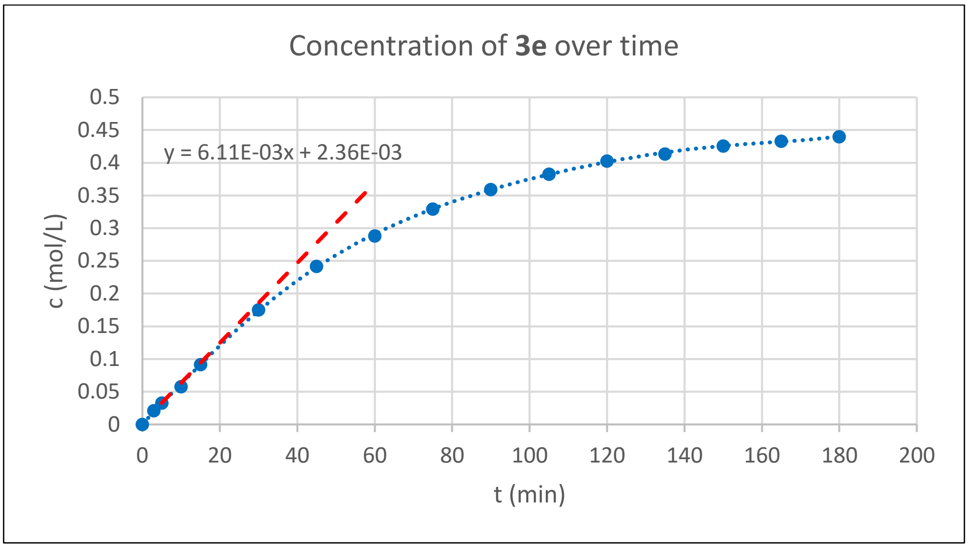

Fitted curve:

$$c = A_0 + A_1 \times t + A_2 \times t^2 + A_3 \times t^3 + A_4 \times t^4 + A_5 \times t^5 + A_6 \times t^6$$

A_0	8.95E-04
A_1	6.04E-03
A_2	1.56E-05
A_3	-1.16E-06
A_4	1.25E-08
A_5	-5.83E-11
A_6	1.02E-13

t (max) = 5 min

v (max) = 6.11×10^{-3} mol/Lmin

Reaction catalyzed with $Pd(PPh_3)_2I_2$ and $Pd(PPh_3)_4$

Following *GP19* using 4-iodotoluene (**1a**) (549 mg, 2.52 mmol), phenylacetylene (**2a**) (0.302 mL, 2.75 mmol), pyrrolidine (0.420 mL, 5.00 mmol), bis(triphenylphosphine)palladium(II) iodide (111 mg, 0.126 mmol), tetrakis(triphenylphosphine)palladium(0) (145 mg, 0.125 mmol), dichloromethane (5 mL).

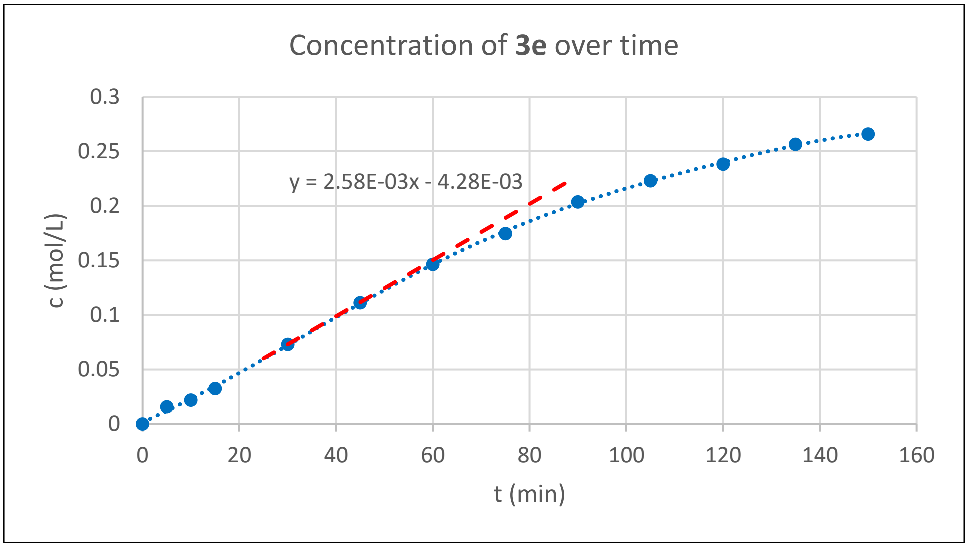

Fitted curve:

$$c = A_0 + A_1 \times t + A_2 \times t^2 + A_3 \times t^3 + A_4 \times t^4 + A_5 \times t^5 + A_6 \times t^6$$

A_0	1.57E-03
A_1	2.03E-03
A_2	1.22E-05
A_3	5.35E-08
A_4	-4.40E-09
A_5	3.72E-11
A_6	-9.57E-14

t (max) = 34 min

v (max) = 2.58×10^{-3} mol/Lmin

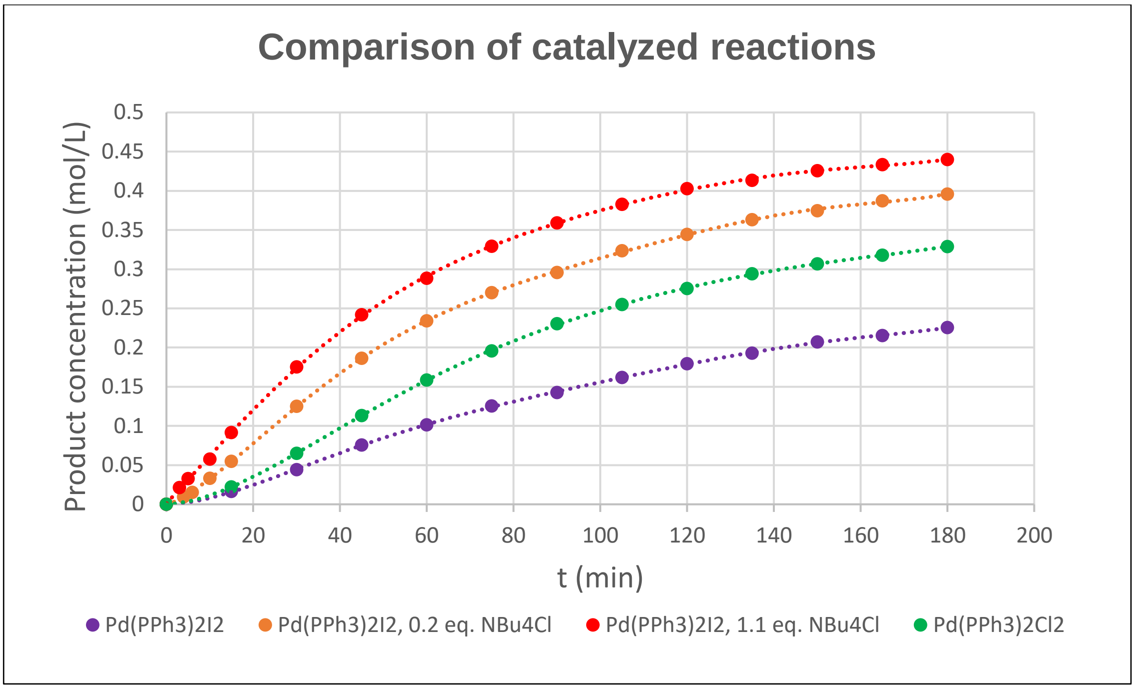

Figure 10b. Comparison of product **3e** formation for reactions, catalyzed by $Pd(PPh_3)_2I_2$ or $Pd(PPh_3)_2Cl_2$ with or without addition of tetrabutylammonium chloride.

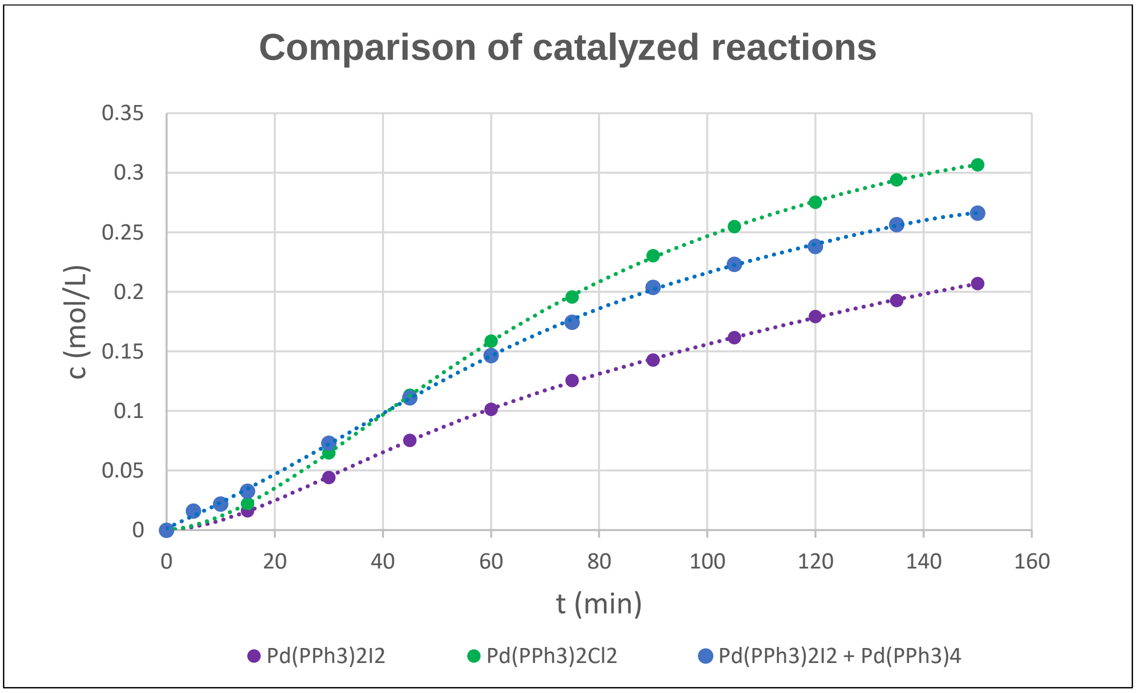

Comparison of product **3e** formation for reactions, catalyzed by $Pd(PPh_3)_2I_2$, $Pd(PPh_3)_2Cl_2$ and $Pd(PPh_3)_2I_2$ with addition of tetrakis(triphenylphosphine)palladium(0).

Effect of tetrabutylammonium chloride on bis(triphenylphosphine)palladium(II) (4-methylphenyl)ide iodide (7a)

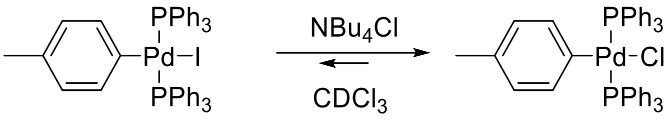

7a **7g**

In a vial a solution of bis(triphenylphosphine)palladium(II) (4-methylphenyl)ide iodide (**7a**) (8.5 mg, 0.01 mmol) in degassed $CDCl_3$ (1.00 mL) was prepared under argon atmosphere. Tetrabutylammonium chloride (13.8 mg, 0.05 mmol) was added to this solution in one portion. The reaction mixture was sonicated for 0.5 min and transferred to a NMR tube, purged with argon and sealed. Reaction was monitored for 1 hour using $^{31}P\{^1H\}$ NMR.

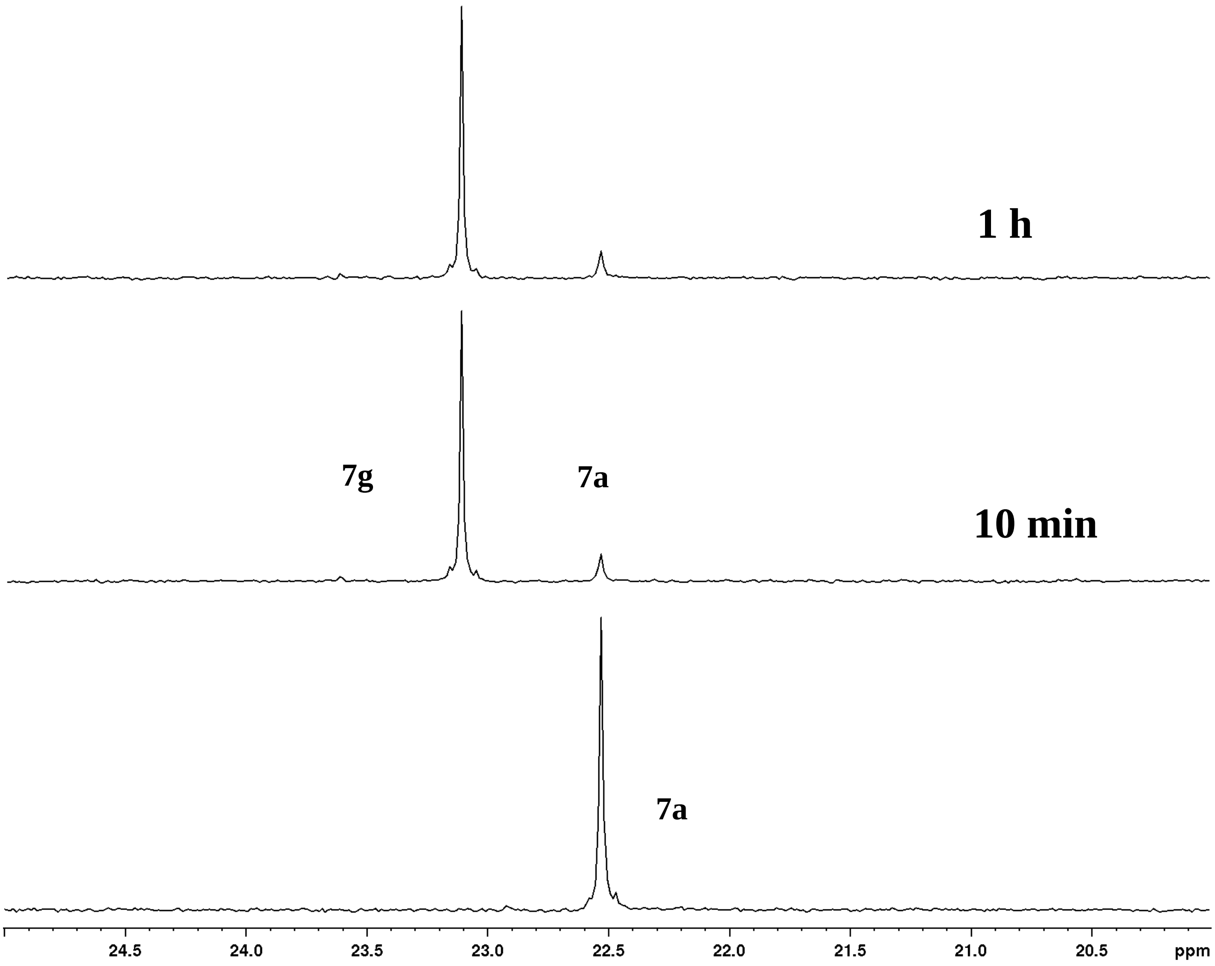

Supplementary Figure 42. Stack of $^{31}P\{^1H\}$ NMR spectra of mixture of **7a** with tetrabutylammonium chloride in $CDCl_3$ after 10 min, 1 hour and 4 hours.

Reaction (equilibrium) was completed in less than 10 min.

Effect of tetrabutylammonium chloride on bis(triphenylphosphine)palladium(II) (4-methylphenyl)ide iodide (5a)

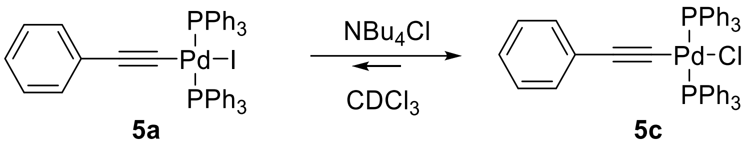

In a vial a solution of **5a** (8.6 mg, 0.01 mmol) in degassed $CDCl_3$ (1.00 mL) was prepared under argon atmosphere. Tetrabutylammonium chloride (13.6 mg, 0.05 mmol) was added to this solution in one portion. The reaction mixture was sonicated for 0.5 min and transferred to a NMR tube, purged with argon and sealed. Reaction was monitored using $^{31}P\{^{1}H\}$ NMR.

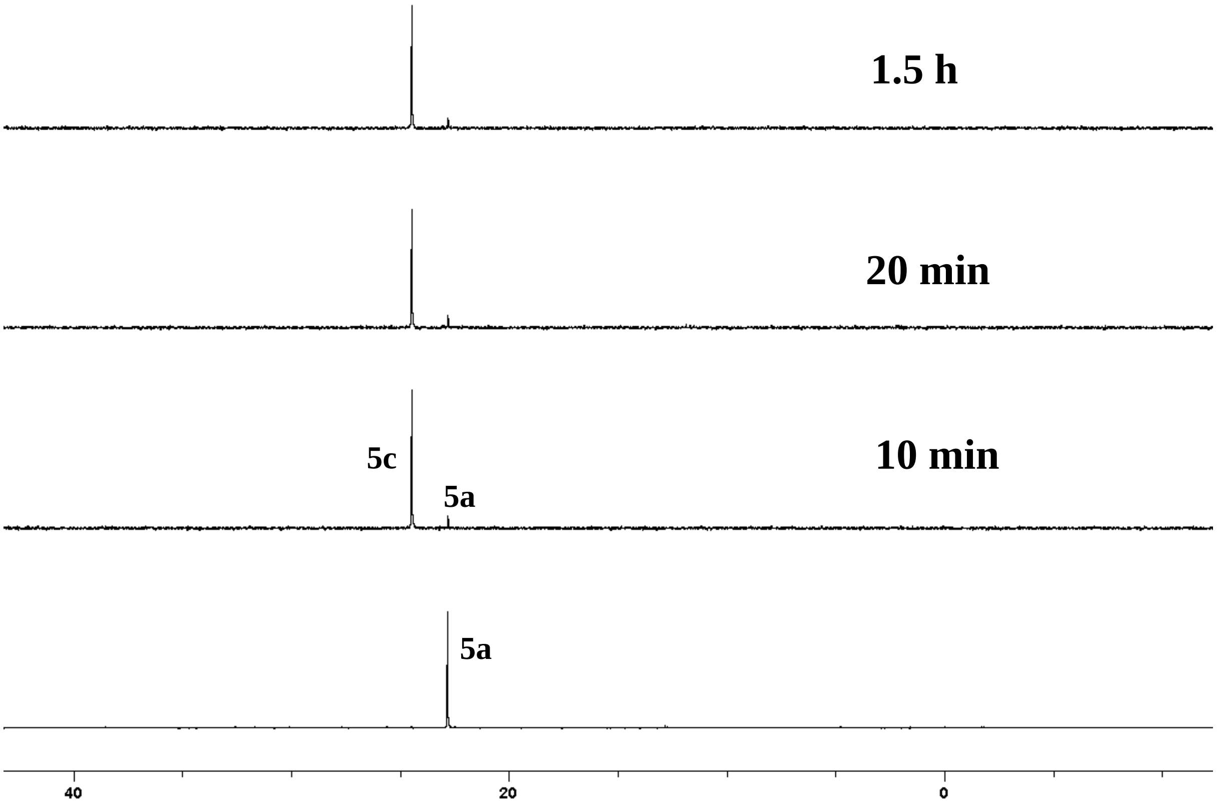

Supplementary Figure 43. Stack of $^{31}P\{^{1}H\}$ NMR spectra of mixture of **5a** with tetrabutylammonium chloride in $CDCl_3$ after 10 min, 20 min and 1.5 hour.

Effect of tetrabutylammonium chloride on bis(triphenylphosphine)palladium(II) bis(phenylethynide) (6a)

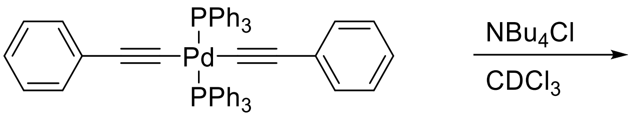

6a

In a vial a solution of bis(triphenylphosphine)palladium(II) bis(phenylethynide) (**6a**) (8.3 mg, 0.01 mmol) in degassed $CDCl_3$ (1.00 mL) was prepared under argon atmosphere. Tetrabutylammonium chloride (27.6 mg, 0.099 mmol) was added to this solution in one portion. The reaction mixture was sonicated for 0.5 min and transferred to a NMR tube, purged with argon and sealed. Reaction was monitored for 4 h using $^{31}P\{^1H\}$ NMR.

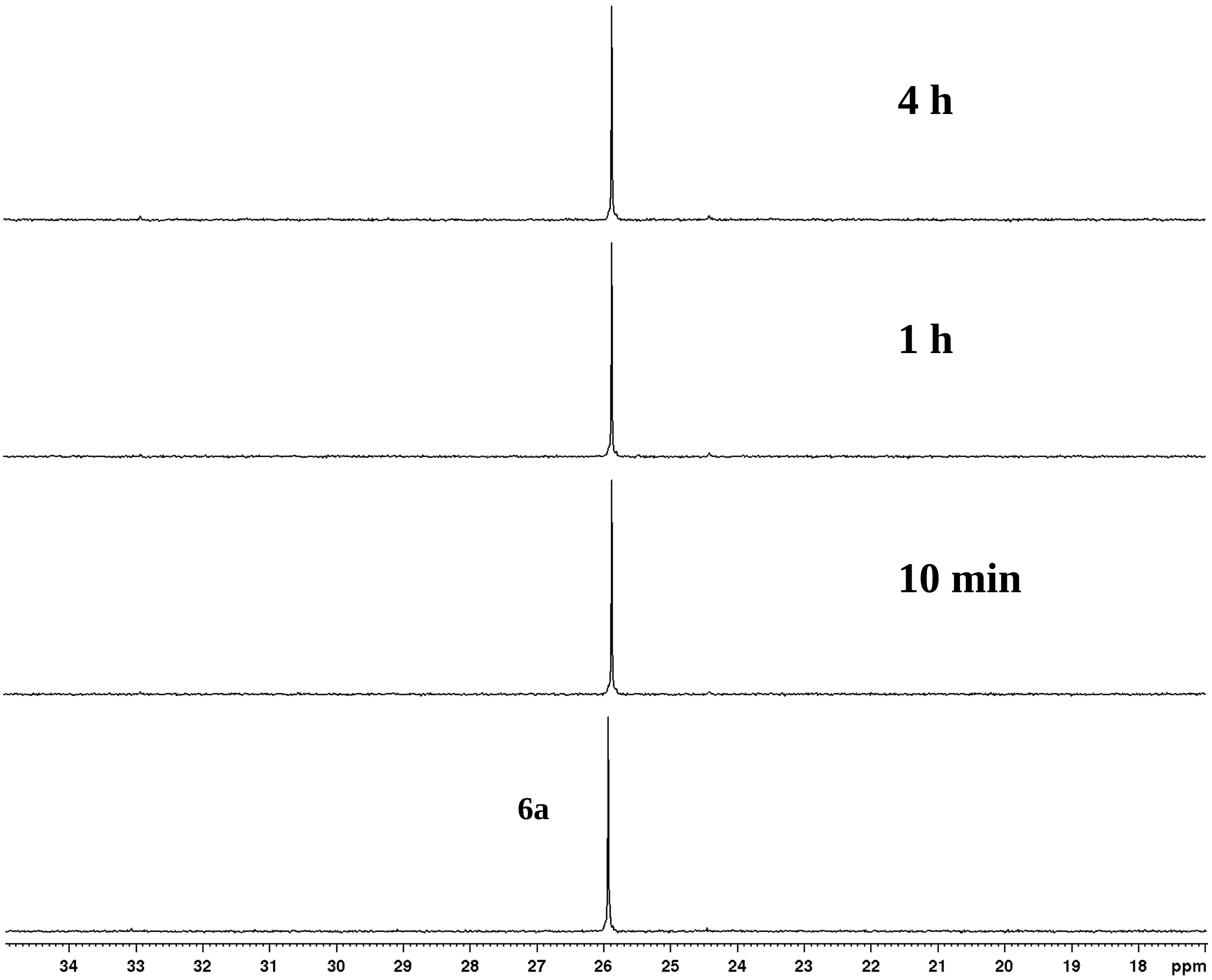

Supplementary Figure 44. Stack of $^{31}P\{^1H\}$ NMR spectra of mixture of **6a** with tetrabutylammonium chloride in $CDCl_3$ after 10 min, 1 hour and 4 hours.

There was no observable reaction.

Effect of tetrabutylammonium chloride on transmetallation of 6a and 7a

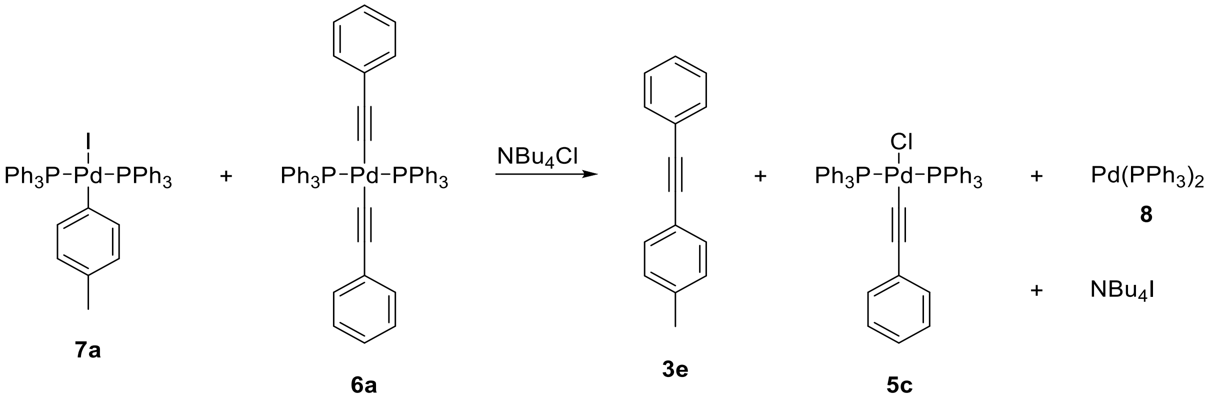

In a vial a solution of bis(triphenylphosphine)palladium (4-methylphenyl)ide iodide (**7a**) (6.93 mg, 8.16 μmol) in degassed $CDCl_3$ (0.800 mL) was prepared under argon atmosphere. Bis(triphenylphosphine)palladium bis(phenylethynide) (**6a**) (7.76 mg, 9.31 μmol) and tetrabutylammonium chloride (4.85 mg, 17.4 μmol) were added to this solution in one portion. The reaction mixture was sonicated for 0.5 min and transferred to NMR tube, flushed with argon and sealed. ^{1}H NMR spectra were acquired in 147 s intervals at 302 K.

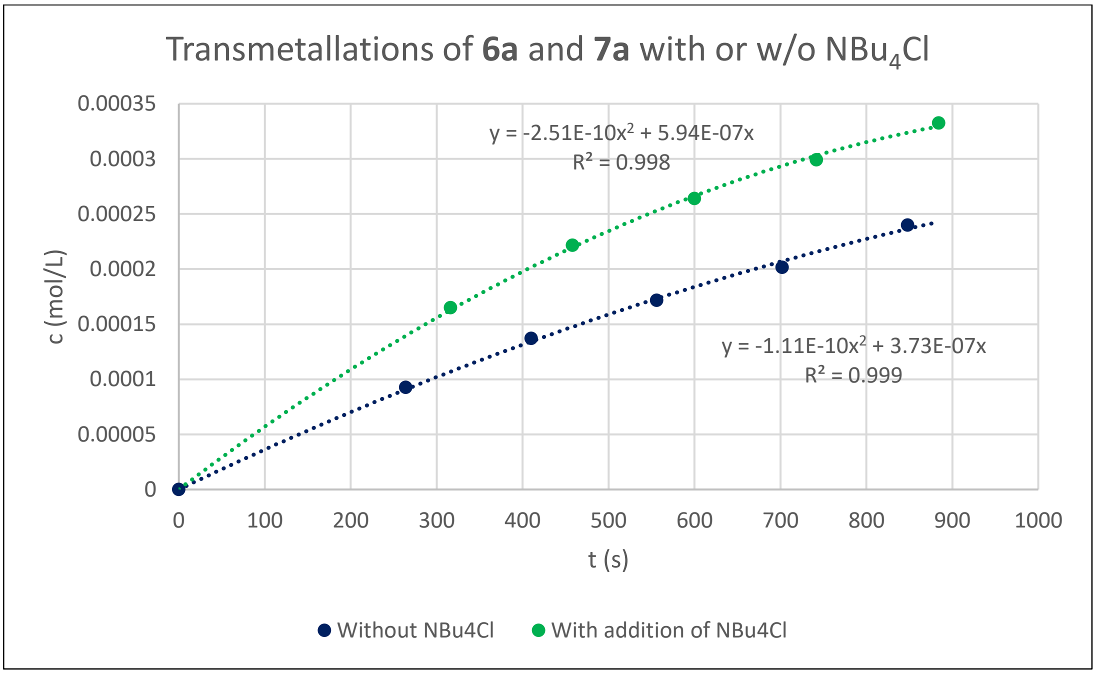

Supplementary Figure 45. Comparison of product **3e** formation for transmetallation between **6a** and **7a** with and without NBu_4Cl.

Rate of transmetallation of **6a** and **7a**: v = 0.37 μmol/Ls (comparable to previously obtained rate: v = 0.38 μmol/Ls; see Transmetallation of **6a** and **7a**, page S90).

Rate of transmetallation of **6a** and **7a** with addition of NBu_4Cl: v = 0.59 μmol/Ls (comparable to previously obtained rate: v = 0.68 μmol/Ls – see Transmetallation of **6a** and **7g**, page S79).

Order in 6a and 7a in transmetallation reaction

Orders in both reactants **6a** and **7a** were determined by measuring initial rates of transmetallation of **6a** and **7a** at different concentrations in each reagent. Reactions were performed according to *GP12* in $CDCl_3$ at 302 K.

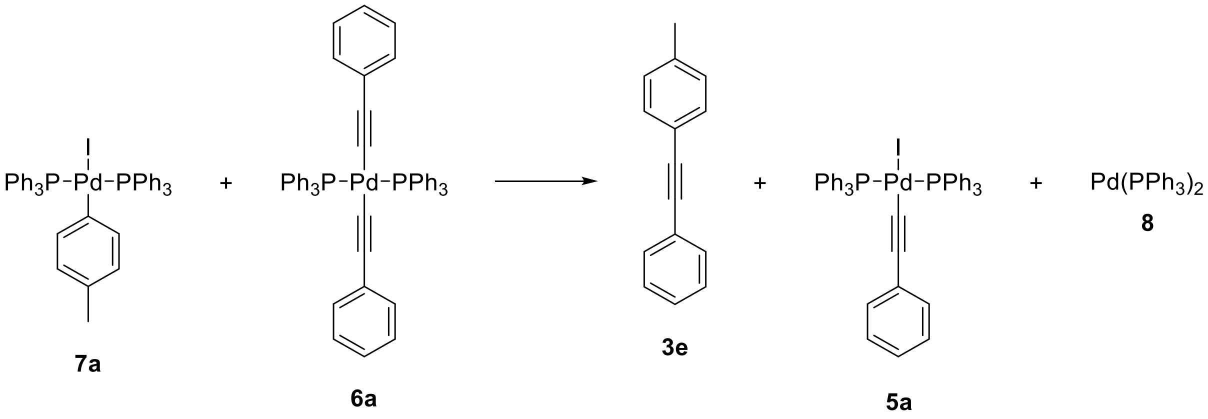

Order in 6a

c_0 (**6a**) = 0.00503 M
c_0 (**7a**) = 0.00493 M

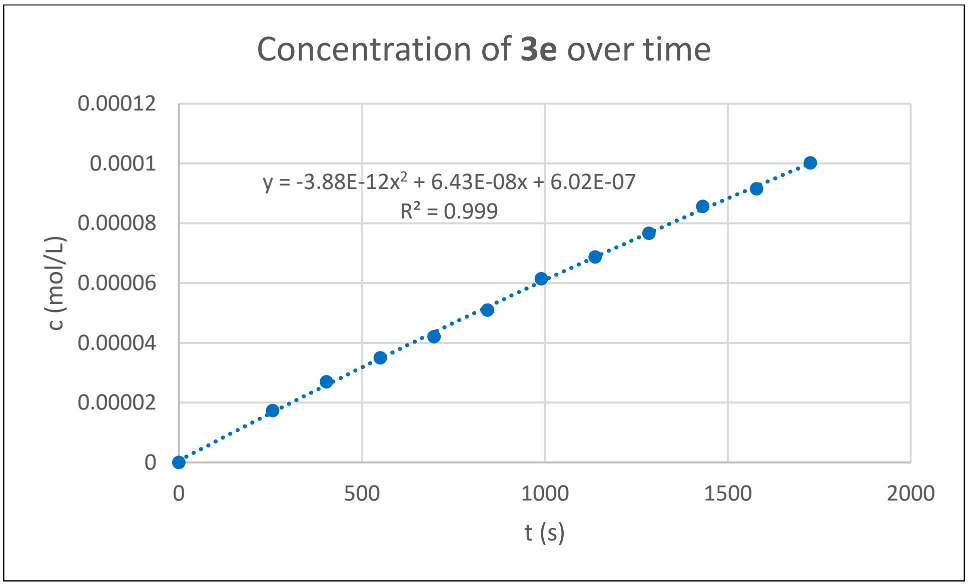

$v_0 = (6.4 \pm 0.2) \times 10^{-8}$ mol/Ls

c_0 (**6a**) = 0.01516 M
c_0 (**7a**) = 0.00489 M

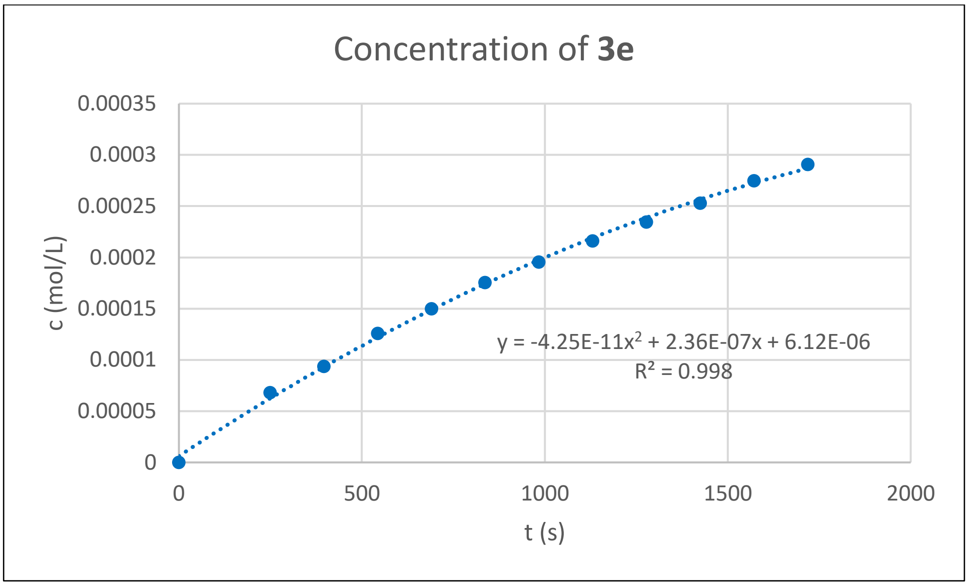

$v_0 = (2.36 \pm 0.09) \times 10^{-7}$ mol/Ls

c_0 (**6a**) = 0.02525 M
c_0 (**7a**) = 0.00506 M

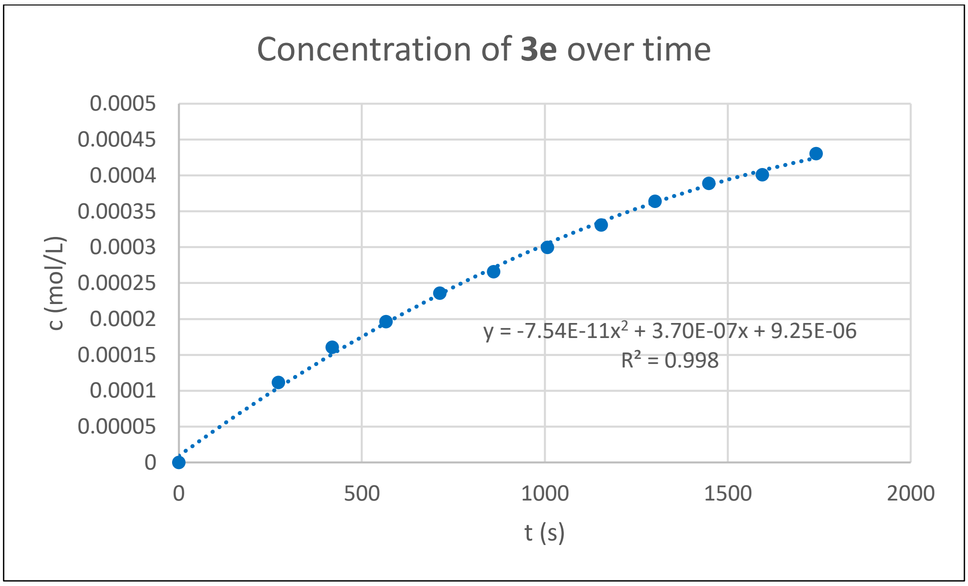

$v_0 = (3.7 \pm 0.1) \times 10^{-7}$ mol/Ls

c (**7a**) [M]	c (**6a**) [M]	ln c (**6a**)	v [mol/Ls]	ln (v)
4.93E-03	5.03E-03	-5.29	6.43E-08	-16.560
4.89E-03	1.52E-02	-4.19	2.36E-07	-15.258
5.06E-03	2.52E-02	-3.68	3.70E-07	-14.811

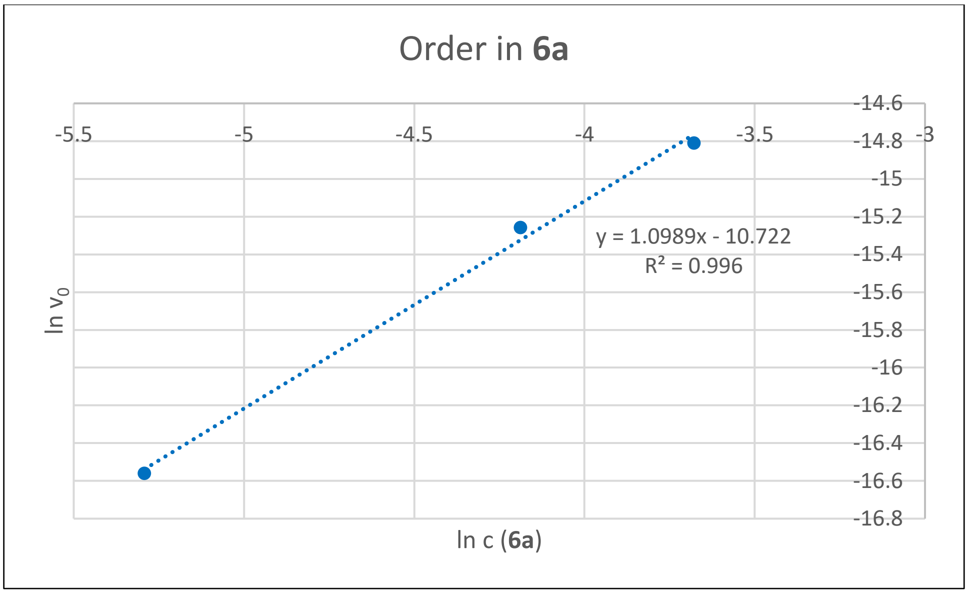

Supplementary Figure 46. Determination of reaction order in **6a**. Results indicate that the reaction is 1st order in **6a**.

Order in 7a

c_0 (**6a**) = 0.00503 M
c_0 (**7a**) = 0.00493 M

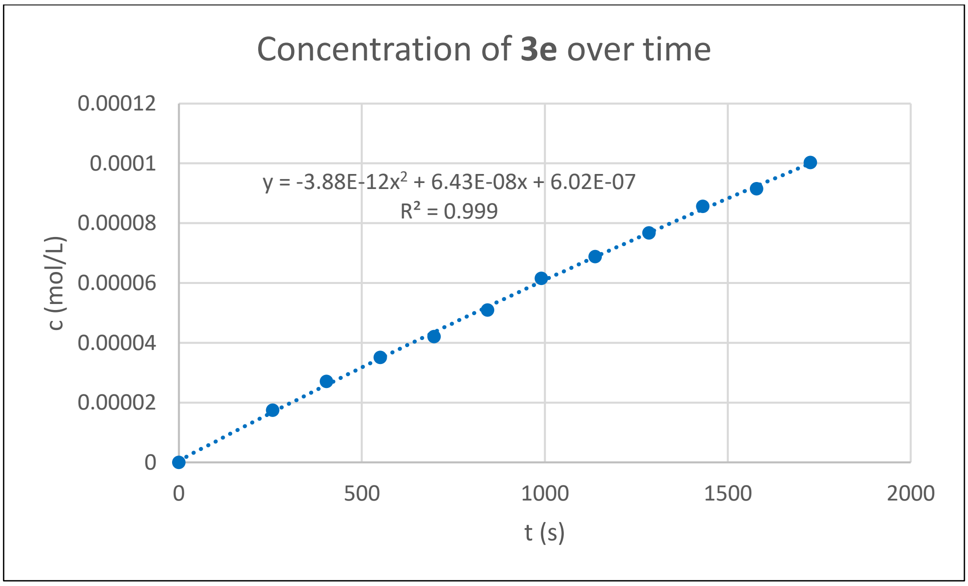

$v_0 = (6.4 \pm 0.2) \times 10^{-8}$ mol/Ls

c_0 (**6a**) = 0.00510 M
c_0 (**7a**) = 0.01499 M

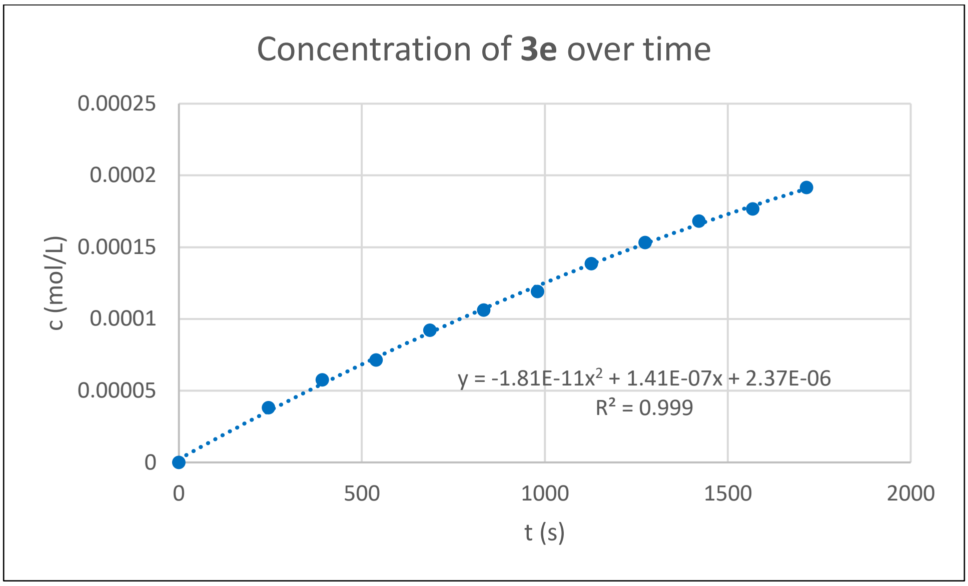

$v_0 = (1.41 \pm 0.05) \times 10^{-7}$ mol/Ls

c_0 (**6a**) = 0.00497 M
c_0 (**7a**) = 0.02513 M

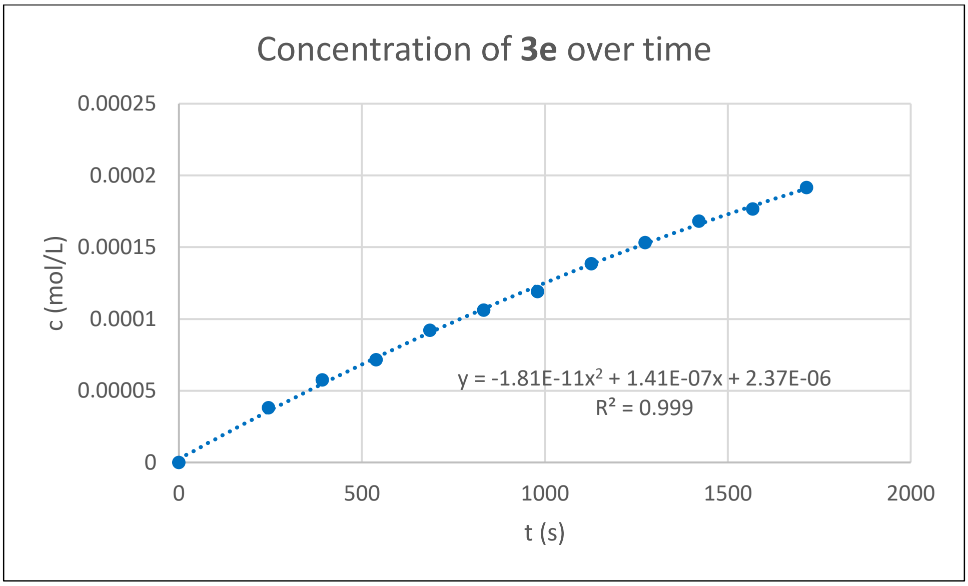

$v_0 = (3.2 \pm 0.2) \times 10^{-7}$ mol/Ls

c (**7a**) [M]	c (**6a**) [M]	ln c (**7a**)	v [mol/Ls]	ln (v)
4.93E-03	5.03E-03	-5.31	6.43E-08	-16.560
1.50E-02	5.10E-03	-4.20	1.41E-07	-15.775
2.51E-02	4.97E-03	-3.68	3.20E-07	-14.954

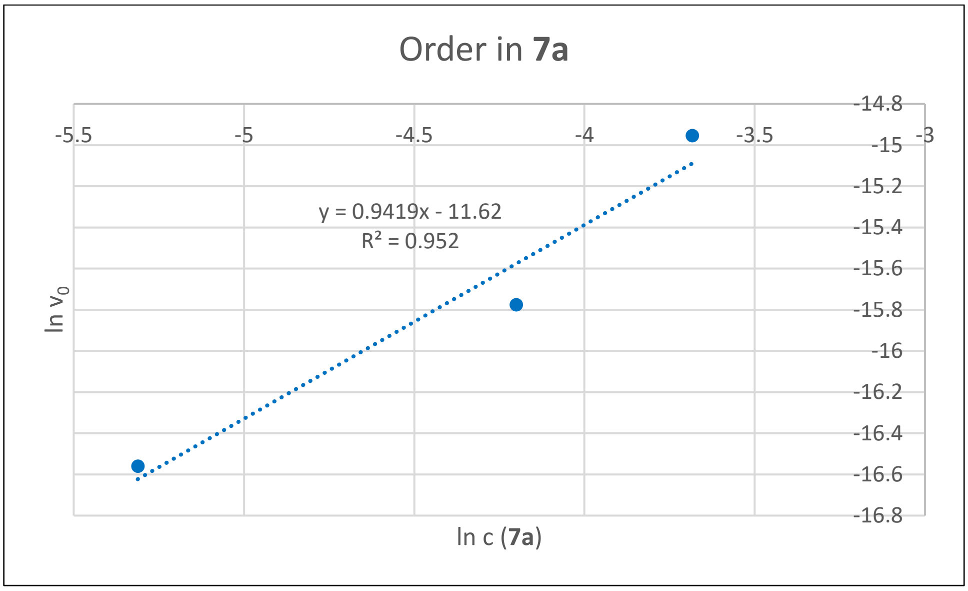

Supplementary Figure 47. Determination of reaction order in **7a**. Results indicate that the reaction is 1st order in **7a**.

Order in 6a and 7a in transmetallation reaction with addition of excess pyrrolidine

Orders in both reactants **6a** and **7a** in transmetallation with excess pyrrolidine were determined by measuring initial rates of transmetallation of **6a** and **7a** at different concentrations in each reagent. Reactions were performed according to *GP12* in $CDCl_3$ with added pyrrolidine (0.1 M in $CDCl_3$) at 302 K. Initial reaction rates were calculated from data for first two hours due to extremely sluggish reactions.

Order in 6a

c_0 (**6a**) = 0.00498 M
c_0 (**7a**) = 0.00499 M

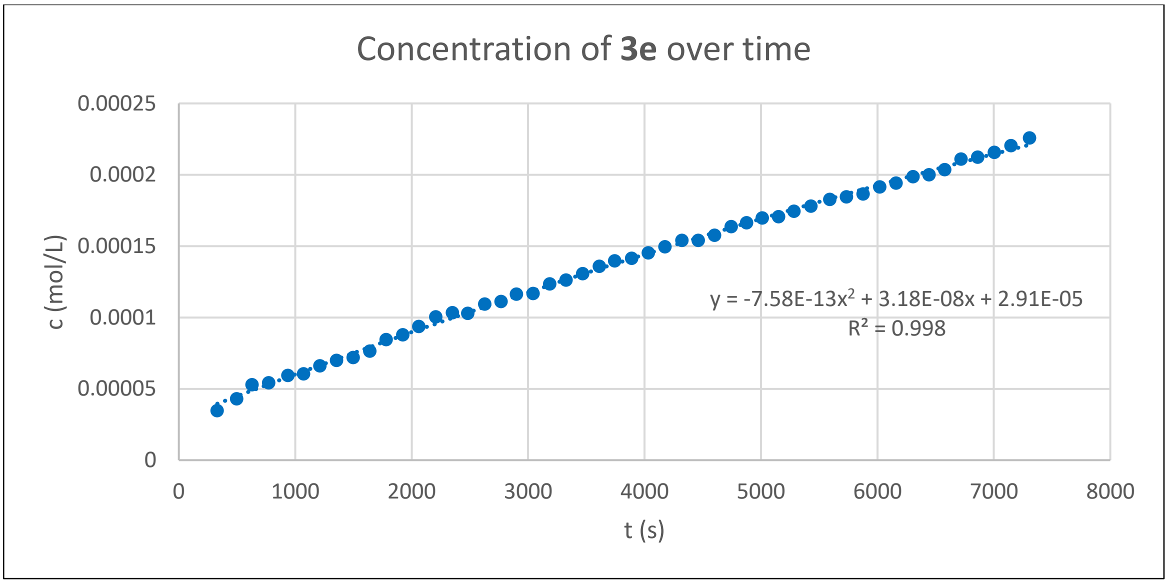

$v_0 = (3.18 \pm 0.06) \times 10^{-8}$ mol/Ls

c_0 (**6a**) = 0.01007 M
c_0 (**7a**) = 0.00496 M

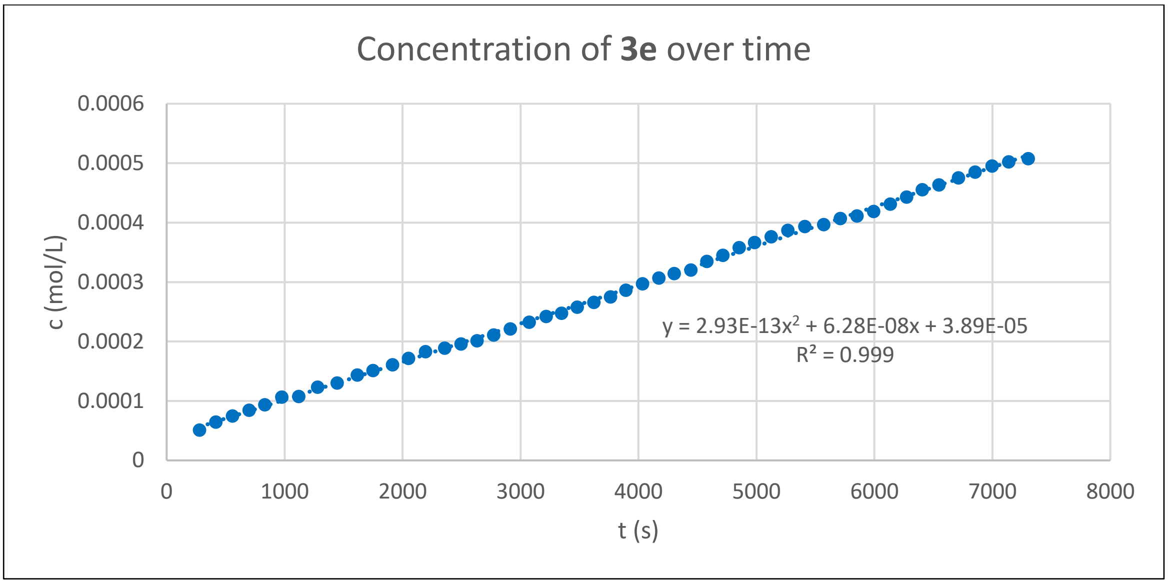

$v_0 = (6.3 \pm 0.1) \times 10^{-8}$ mol/Ls

c_0 (**6a**) = 0.01497 M
c_0 (**7a**) = 0.00502 M

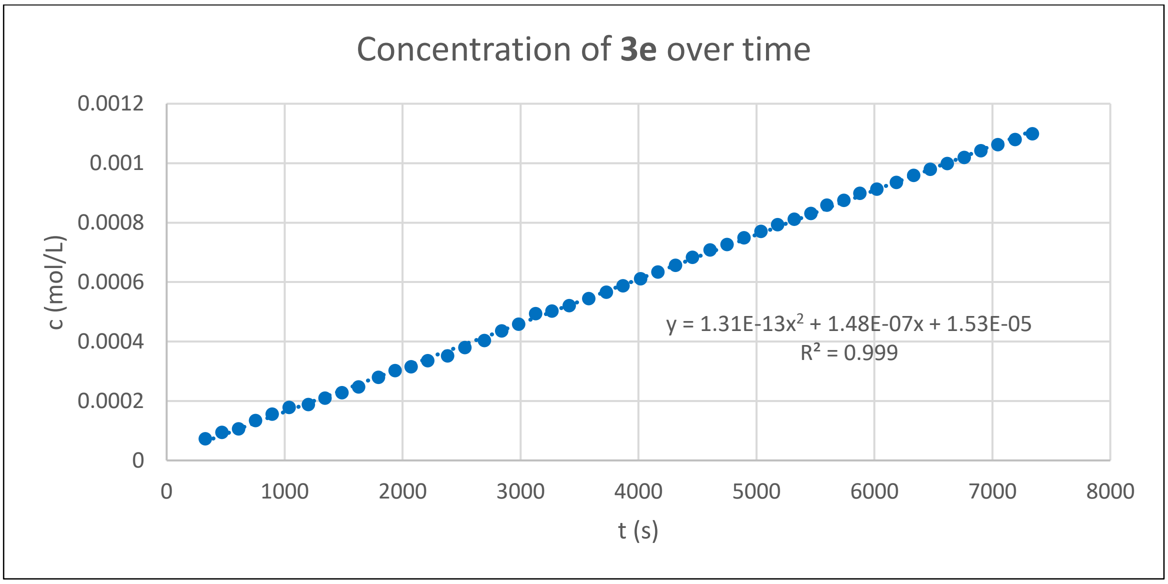

$v_0 = (1.48 \pm 0.02) \times 10^{-7}$ mol/Ls

c_0 (**6a**) = 0.02495 M
c_0 (**7a**) = 0.00499 M

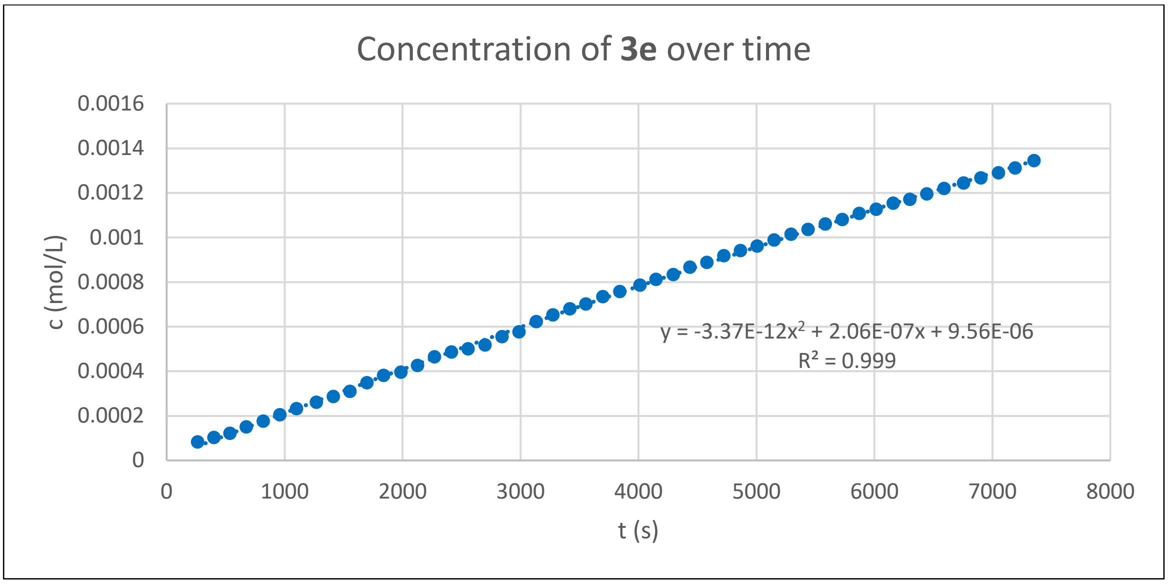

$v_0 = (2.06 \pm 0.02) \times 10^{-7}$ mol/Ls

c (**7a**) [M]	c (**6a**) [M]	ln c (**6a**)	v [mol/Ls]	ln (v)
4.99E-03	4.98E-03	-5.30	3.18E-08	-17.264
4.96E-03	1.01E-02	-4.60	6.28E-08	-16.578
5.02E-03	1.50E-02	-4.20	1.48E-07	-15.726
4.99E-03	2.50E-02	-3.69	2.06E-07	-15.394

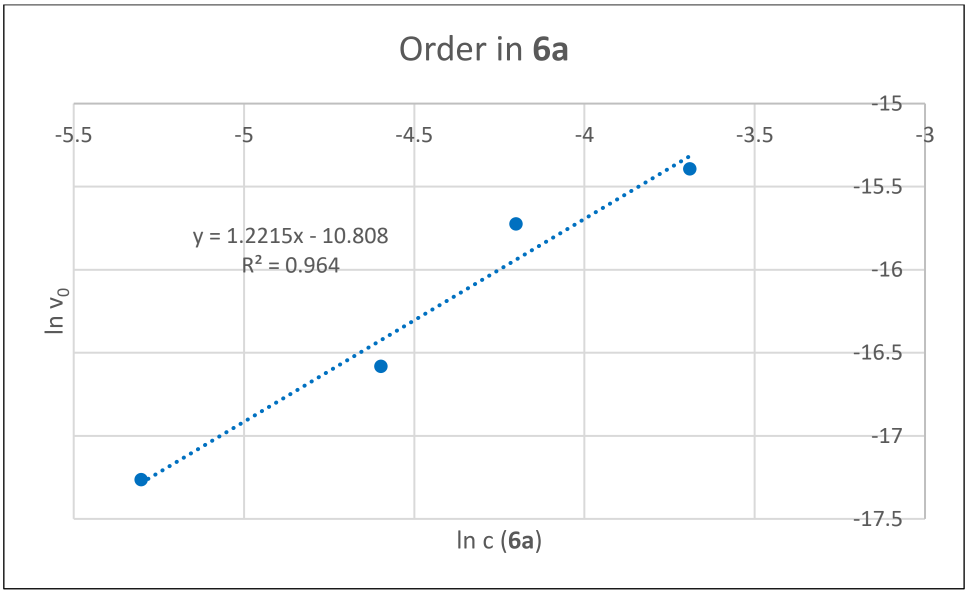

Supplementary Figure 48. Determination of reaction order in **6a** when excess of pyrrolidine is used in transmetallation. Results indicate that the reaction is 1st order in **6a**.

Order in 7a

c_0 (**6a**) = 0.00498 M
c_0 (**7a**) = 0.00499 M

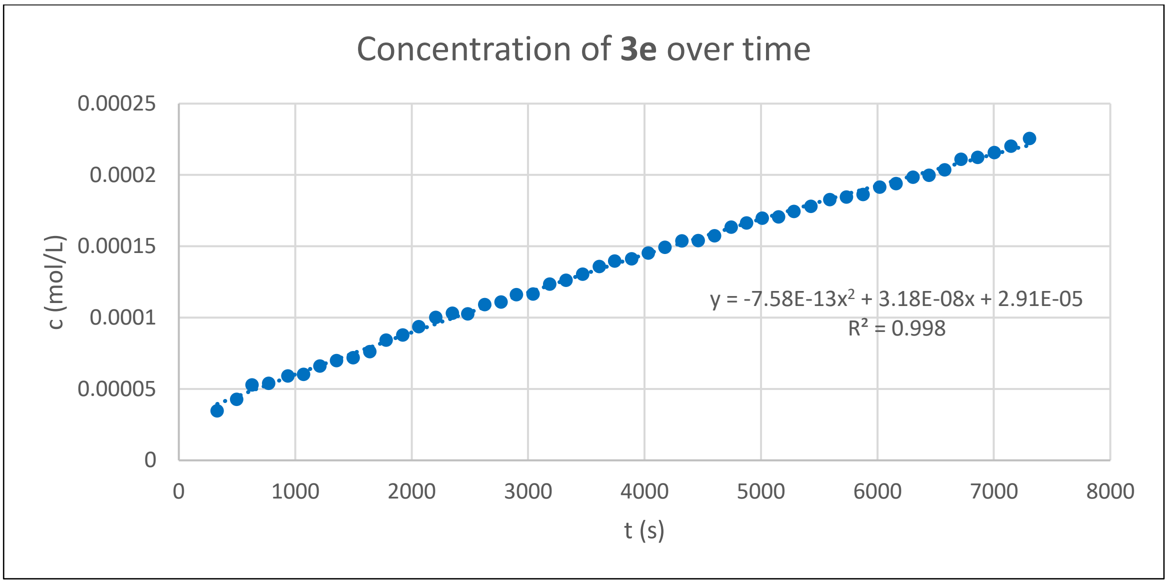

$v_0 = (3.18 \pm 0.06) \times 10^{-8}$ mol/Ls

c_0 (**6a**) = 0.00495 M
c_0 (**7a**) = 0.01006 M

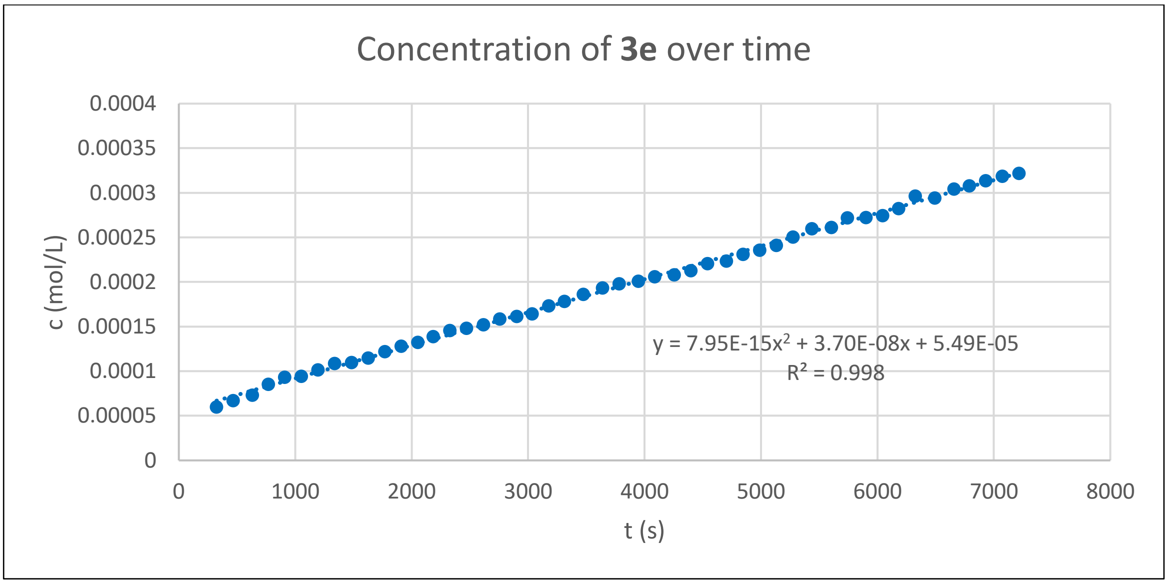

$v_0 = (3.7 \pm 0.1) \times 10^{-8}$ mol/Ls

c_0 (**6a**) = 0.00507 M
c_0 (**7a**) = 0.01509 M

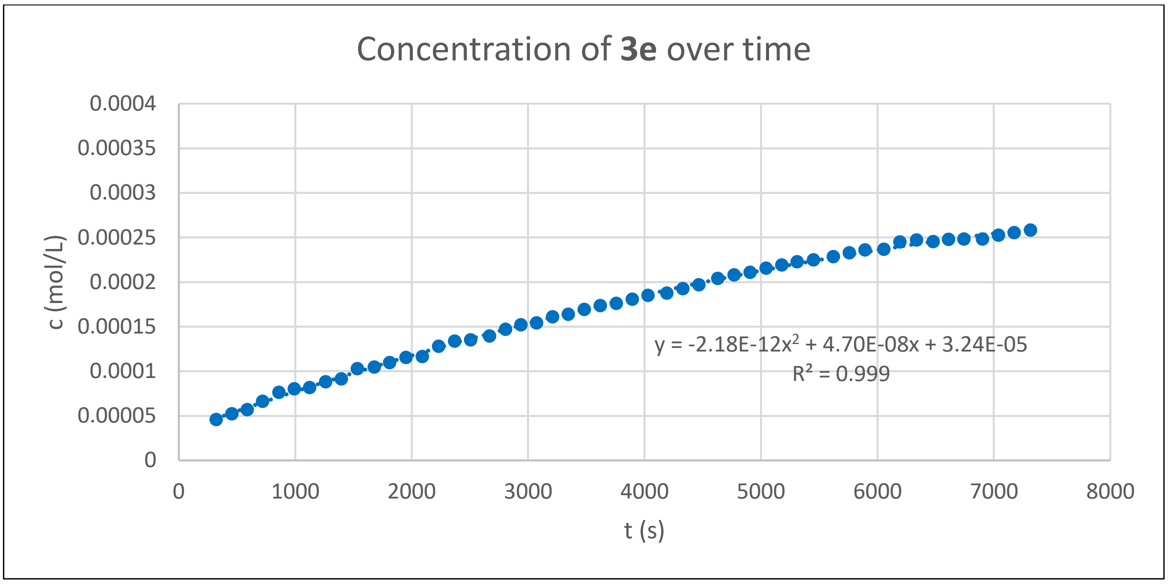

$v_0 = (4.70 \pm 0.07) \times 10^{-8}$ mol/Ls

c_0 (**6a**) = 0.00497 M
c_0 (**7a**) = 0.02491 M

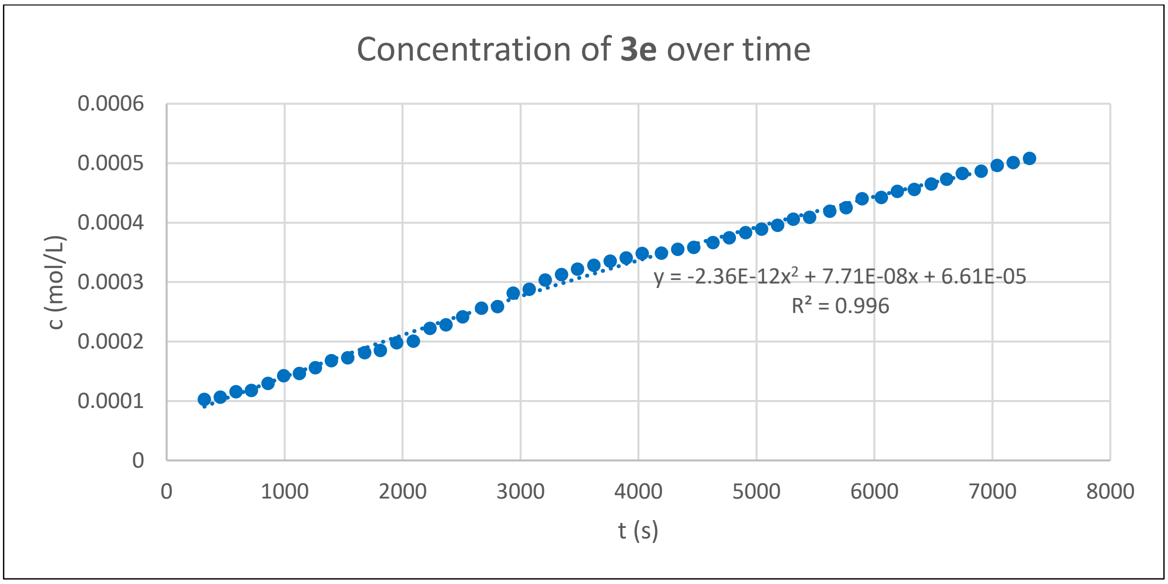

$v_0 = (7.7 \pm 0.2) \times 10^{-8}$ mol/Ls

c (**7a**) [M]	c (**6a**) [M]	ln c (**7a**)	v [mol/Ls]	ln (v)
4.99E-03	4.98E-03	-5.30	3.18E-08	-17.264
1.01E-02	4.95E-03	-4.60	3.70E-08	-17.112
1.51E-02	5.07E-03	-4.19	4.70E-08	-16.874
2.49E-02	4.97E-03	-3.69	7.71E-08	-16.378

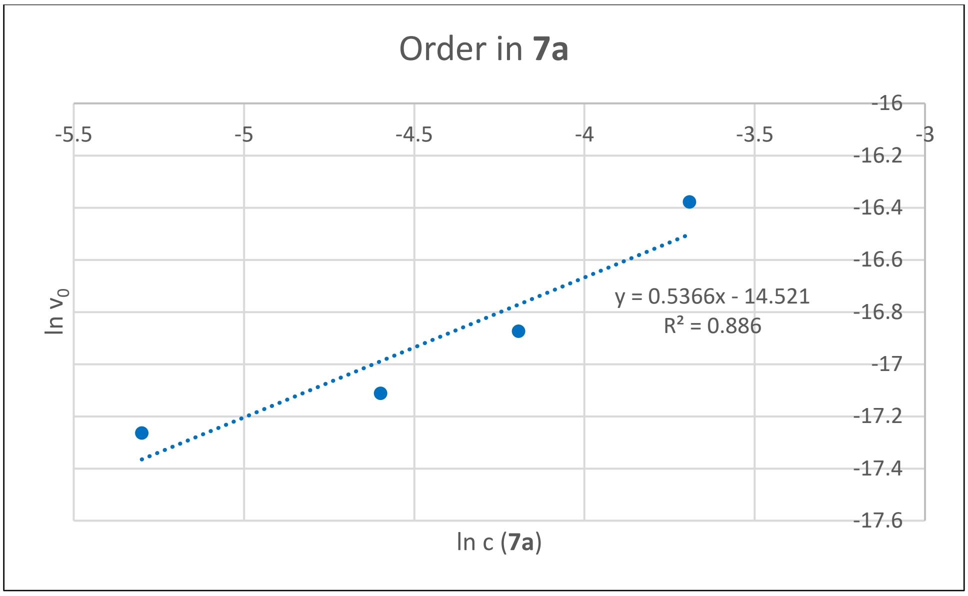

Supplementary Figure 49. Determination of reaction order in **7a** when excess of pyrrolidine is used in transmetallation. Results indicate that the reaction is 0.5th order in **7a**.

Comparison of transmetallation rates in the presence and the absence of pyrrolidine

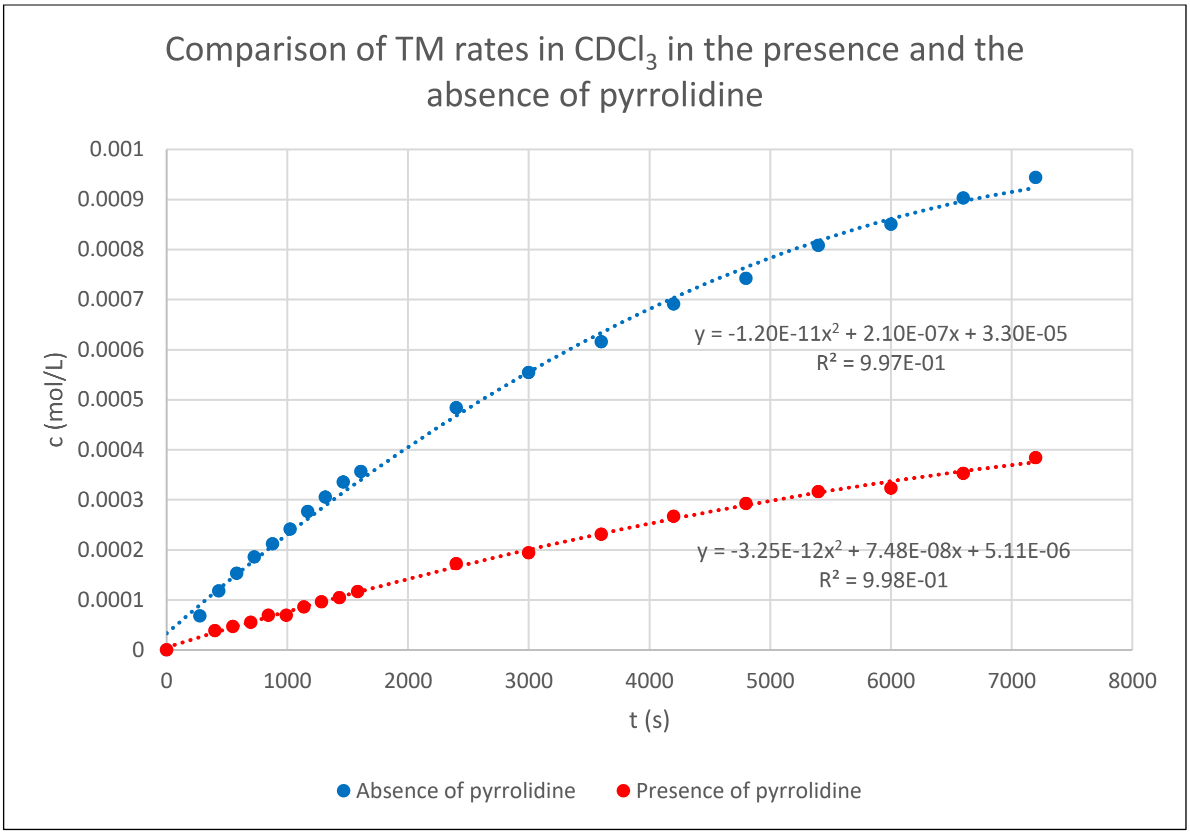

Supplementary Figure 50. Comparison of transmetallation reaction between **6a** and **7a** in the absence and the presence of pyrrolidine. The reaction proceeds faster in the absence of pyrrolidine, further indicating the tricoordinated **7’** as reactive species in catalytic reaction.

3. SUPPLEMENTARY REFERENCES

(1) Picard, J., Lubin-Germain, N., Uziel J. & Augé J. Indium-Mediated Alkynylation in C-Glycoside Synthesis. *Synthesis* **6**, 979–982 (2006).

(2) Stefani, H. A. *et al.* Ultrasound-assisted synthesis of functionalized arylacetylenes. *Tetrahedron Lett*. **12**, 2001–2003 (2005).

(3) Yan, J., Li, J. & Cheng, D. Novel and Efficient Synthesis of 1-Iodoalkynes. *Synlett*. **15**, 2442–2444 (2007).

(4) Gómez-Herrera, A., Nahra, F., Brill, M., Nolan, S. P. & Cazin, C. S. J. Sequential functionalisation of alkynes and alkenes catalysed by Au(I)- and Pd(II)-NHC complexes. *ChemCatChem* **21**, 3381–3388 (2016).

(5) Chen, S. N., Hung, T. T., Lin, T. C. & Tsai, F. Y. Reusable and Efficient CuI/TBAB-Catalyzed Iodination of Terminal Alkynes in Water under Air. *J. Chin. Chem. Soc*. **5**, 1078–1081 (2009).

(6) Osowska, K., Lis, T. & Szafert, S. Protection/Deprotection-Free Syntheses and Structural Analysis of (Keto-aryl)diynes. *Eur. J. Org. Chem.* **27**, 4598–4606 (2008).

(7) Li, L. S. & Wu, Y. L. An efficient method for synthesis of α-keto acid esters from terminal alkynes. *Tetrahedron Lett*. **43**, 2427–2430 (2002).

(8) Jiang, M. X.-W., Rawat, M. & Wulff, W. D. Contingency and Serendipity in the Reactions of Fischer Carbene Complexes with Conjugated Triynes. *J. Am. Chem. Soc.* **126**, 5970–5971 (2004).

(9) Edgars, A., Rubina, K., Abele, R., Gaukhman, A. & Lukevics, E. Mild and Selective Phase Transfer Catalysed Bromination of Terminal Acetylenes Using Carbon Tetrabromide as Reagent. *J. Chem. Res.* **9**, 618–619 (1998).

(10) Molander, G. A. & Fumagalli, T. Palladium(0)-Catalyzed Suzuki-Miyaura Cross-Coupling Reactions of Potassium Aryl- and Heteroaryltrifluoroborates with Alkenyl Bromides. *J. Org. Chem*. **71**, 5743–5747 (2006).

(11) Wang, Y., Shao, Z., Zhang, K. & Liu, Q. Manganese-Catalyzed Dual-Deoxygenative Coupling of Primary Alcohols with 2-Arylethanols. *Angew. Chem. Int. Ed.* **46**, 15143–15147 (2018).

(12) Martins, M. A. P. *et al.* Synthesis of new halo-containing acetylenes. *Tetrahedron Lett*. **25**, 4935–4938 (2004).

(13) Abele, É., Abele, R., Rubina, K. & Lukevics, E. Chlorination of Pyridylacetylenes in the Phase-Transfer Catalytic System CCl_4/KOH/18-Crown-6. *Chem. Heterocycl. Compd.* **34**, 122–123 (1998).

(14) Mann, G., Baranano, D., Hartwig, J. F., Rheingold, A. L. & Guzei, I. A. Carbon-Sulfur Bond-Forming Reductive Elimination Involving sp-, sp^2-, and sp^3-Hybridized Carbon. Mechanism, Steric Effects, and Electronic Effects on Sulfide Formation. *J. Am. Chem. Soc.* **120**, 9205–9219 (1998).

(15) Weigelt, M., Bechner, D., Poetsch, E., Bruhn, C. & Steinborn, D. On the Oxidative Addition of 1-Halogenalk-1-ynes – Synthesis and Structure of Phenylalkynylpalladium Complexes. *Z. Anorg. Allg. Chem.* **9**, 1542–1547 (1999).

(16) Haindl, S., Xu, J., Freese, T., Hübner, E. G. & Schmidt, A. The intersection of allenylidenes and mesomeric betaines. 1-Methylpyridinium-2-acetylide and its palladium complexes. *Tetrahedron* **49**, 7906–7911 (2016).

(17) Gazvoda, M., Virant, M., Pinter, B. & Košmrlj, J. Mechanism of copper-free Sonogashira reaction operates through palladium-palladium transmetallation. *Nat. Commun.* **9**, 4814 (2018).

(18) D'Amato, R. *et al.* Synthesis, characterisation and optical properties of symmetrical and unsymmetrical Pt(II) and Pd(II) bis-acetylides. Crystal structure of *trans*-$[Pt(PPh_3)_2(C{\equiv}C–C_6H_5)(C{\equiv}C–C_6H_4NO_2)]$. *J. Organomet. Chem*. **1**, 13–22 (2001).

(19) Izawa, Y., Shimizu, I. & Yamamoto, A. Palladium-Catalyzed Oxidative Carbonylation of 1-Alkynes into 2-Alkynoates with Molecular Oxygen as Oxidant. *Bull. Chem. Soc. Jap*. **11**, 2033–2045 (2004).

(20) Catellani, M., Chiusoli, G. P., Maria, C. F. & Solari, G. Intramolecular Cyclization of *ortho*-Iodophenyl 3-Butenoate to 4-Methylcoumarin: Catalysis by Palladium Complexes. *Tetrahedron Lett.* **32**, 5919–5922 (1994).

(21) Fu, H. *et al.* Synthesis and characterization of mono- and dinuclear aryl palladium(II) complexes: oxidative additions of 1,4-dihalogenated benzene or 4,4'-dibromobiphenyl to $Pd(PR_3)_4$. *J. Coord. Chem.* **3**, 482–494 (2014).

(22) Herrmann, W. A., Broßmer, C., Priermeier, T. & Öfele, K. Komplexchemie und Mechanismen metallkatalysierter CC-Kupplungsreaktionen II*. Oxidative Addition von Chloraromaten an Pd^0-Komplexe: Synthese, Struktur und Stabilitgt von Arylpalladium(II)-chloriden der Phosphanreihe. *J. Organomet. Chem*. **481**, 97–108 (1994).

(23) Wallow, T. I., Goodson, F. E. & Novak, B. M. New Methods for the Synthesis of $ArPdL_2I$ (L = Tertiary Phosphine) Complexes. *Organometallics* **15**, 3708–3716 (1996).

(24) Zhang, S. *et al.* Sythesis of polyfluorene containing simple functional end group with aryl palladium(II) complexes as initiators. *J. Organomet. Chem.* **825–826**, 100–113 (2016).

(25) Amatore, C., Bensalem, S., Ghalem, S. & Jutand, A. Mechanism of the carbopalladation of alkynes by aryl-palladium complexes. *J. Organomet. Chem.* **24**, 4642–4646 (2004).

(26) Maazaoui, R. *et al.* Catalytic Chemoselective and Stereoselective Semihydrogenation of Alkynes to *E*-Alkenes Using the Combination of Pd Catalyst and ZnI_2. *Org. Lett.* **20**, 7544–7549 (2018).

(27) Park, S. B. & Alper, H. Recyclable Sonogashira coupling reactions in an ionic liquid, effected in the absence of both a copper salt and a phosphine. *Chem. Commun.* **11**, 1306–1307 (2004).

(28) Katritzky, A. R., Rogovoy, B. V. & Mitrokhin, A. Y. The preparation of diarylacetylenes via diphenyl (benzotriazol-1-yl)(aryl)methylphosphonates. *ARKIVOC* **13**, 17–27 (2002).

(29) Gholap, A. R. *et al.* Copper- and Ligand-Free Sonogashira Reaction Catalyzed by Pd(0) Nanoparticles at Ambient Conditions under Ultrasound Irradiation. *J. Org. Chem.* **70**, 4869–4872 (2005).

(30) Ye, C., Li, Y. & Bao, H. Copper-Catalyzed Decarboxylative Alkylation of Terminal Alkynes. *Adv. Synth. Catal.* **21**, 3720–3724 (2017).

(31) Hennrich, G. *et al.* Alkynyl Expanded Donor–Acceptor Calixarenes: Geometry and Second-Order Nonlinear Optical Properties. *Chem. Eur. J.* **27**, 7753–7761 (2007).

(32) Liang, B., Dai, M., Chen, J. & Yang, Z. Copper-Free Sonogashira Coupling Reaction with $PdCl_2$ in Water under Aerobic Conditions. *J. Org. Chem.* **70**, 391–393 (2005).

(33) Zhao, D. *et al.* Copper-catalyzed decarboxylative cross-coupling of alkynyl carboxylic acids with aryl halides. *Chem. Commun.* **47**, 9049–9051 (2010).

(34) He, H. & Wu, Y. J. Copper-catalyzed cross-coupling of aryl iodides and aryl acetylenes using microwave heating. *Tetrahedron Lett*. **16**, 3237–3239 (2004).

(35) Sakaguchi, T., Kameoka, K. & Hashimoto, T. Synthesis and Gas Permeability of Novel Poly(diphenylacetylenes) Having Polyethylene Glycol Moieties. *Polym. Bull*. **60**, 441–448 (2008).

(36) Johnson, S. A. *et al.* Regioselective Coupling of Pentafluorophenyl Substituted Alkynes: Mechanistic Insight into the Zirconocene Coupling of Alkynes and a Facile Route to Conjugated Polymers Bearing Electron-Withdrawing Pentafluorophenyl Substituents. *J. Am. Chem. Soc.* **125**, 4199–4211 (2003).

(37) Shi, Y., Li, X., Liu, J., Jiang, W. & Sun, L. $PdCl_2$-catalyzed cross-coupling reaction of arylacetylene iodides with arylboronic acids to diarylacetylenes. *Tetrahedron Lett*. **28**, 3626–3628 (2010).

(38) Ljungdahl, T., Bennur, T., Dallas, A., Emtenäs, H. & Mårtensson, J. Two Competing Mechanisms for the Copper-Free Sonogashira Cross-Coupling Reaction. *Organometallics* **27**, 2490–2498 (2008).

(39) Shirakawa, E., Kitabata, T., Otsuka, H. & Tsuchimoto, T. A. A simple catalyst system for the palladium-catalyzed coupling of aryl halides with terminal alkynes. *Tetrahedron* **61**, 9878–9885 (2005).

(40) Paul, F., Patt, J. & Hartwig, J. F. Structural Characterization and Simple Synthesis of {Pd[P(*o*-Tol)$_3$]$_2$}, Dimeric Palladium(II) Complexes Obtained by Oxidative Addition of Aryl Bromides, and Corresponding Monometallic Amine Complexes. *Organometallics* **14**, 3030–3039 (1995).

(41) Kong, K.-C. & Cheng, C.-H. Facile Aryl-Aryl Exchange between the Palladium Center and Phosphine Ligands in Palladium(II) Complexes. *J. Am. Chem. Soc.* **16**, 6313–6315 (1991).

(42) Morita, D. K., Stille, J. K. & Norton, J. R. Methyl/Phenyl Exchange between Palladium and a Phosphine Ligand. Consequences for Catalytic Coupling Reactions. *J. Am. Chem. Soc.* **33**, 8576–8581 (1995).

(43) Burés, J. Variable Time Normalization Analysis: General Graphical Elucidation of Reaction Orders from Concentration Profiles. *Angew. Chem. Int. Ed.* **55**, 16084–16087 (2016).
