# Supplementary material for: Elucidating the reaction mechanism of a palladium-palladium dual catalytic process through kinetic studies of proposed elementary steps
Source: Commun Chem. 2023 Mar 18;6:51. doi: 10.1038/s42004-023-00849-x (PMC10024772; doi:10.1038/s42004-023-00849-x)
Supplement: Supplementary file 3 — Description of Additional Supplementary Files [file 42004_2023_849_MOESM3_ESM.pdf]

Description of Additional Supplementary Files

File name: Supplementary Data 1

Description: NMR spectra data
