# Supplementary material for: Elucidating the reaction mechanism of a palladium-palladium dual catalytic process through kinetic studies of proposed elementary steps
Source: Commun Chem. 2023 Mar 18;6:51. doi: 10.1038/s42004-023-00849-x (PMC10024772; doi:10.1038/s42004-023-00849-x)

Supplementary Information for

Elucidating reaction mechanism of palladium-palladium dual catalytic process through kinetic studies of proposed elementary steps

Anže Ivančič, Janez Košmrlj, Martin Gazvoda*

University of Ljubljana, Faculty of Chemistry and Chemical Technology, Večna pot 113, 1001 Ljubljana, Slovenia

email: martin.gazvoda@fkkt.uni-lj.si

SUPPLEMENTARY DATA 1

Copies of NMR spectra

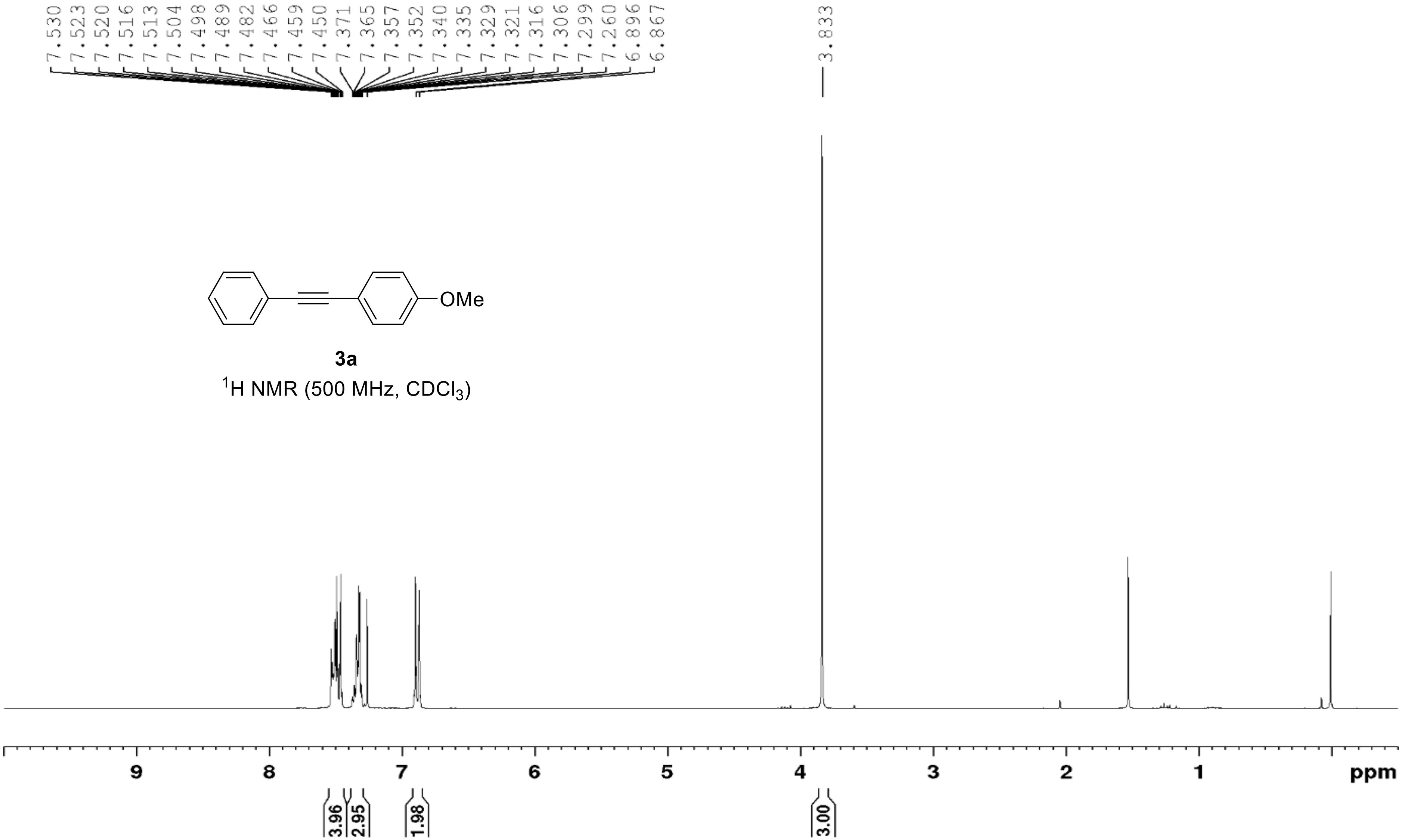
7.530 7.523 7.520 7.516 7.513 7.504 7.498 7.489 7.482 7.466 7.459 7.450 7.371 7.365 7.357 7.352 7.340 7.335 7.329 7.321 7.316 7.306 7.299 7.260 6.896 6.867
3.833
OMe
3a
^{1}H NMR (500 MHz, $CDCl_3$)
9 8 7 6 5 4 3 2 1 ppm
3.96 2.95 1.98 3.00

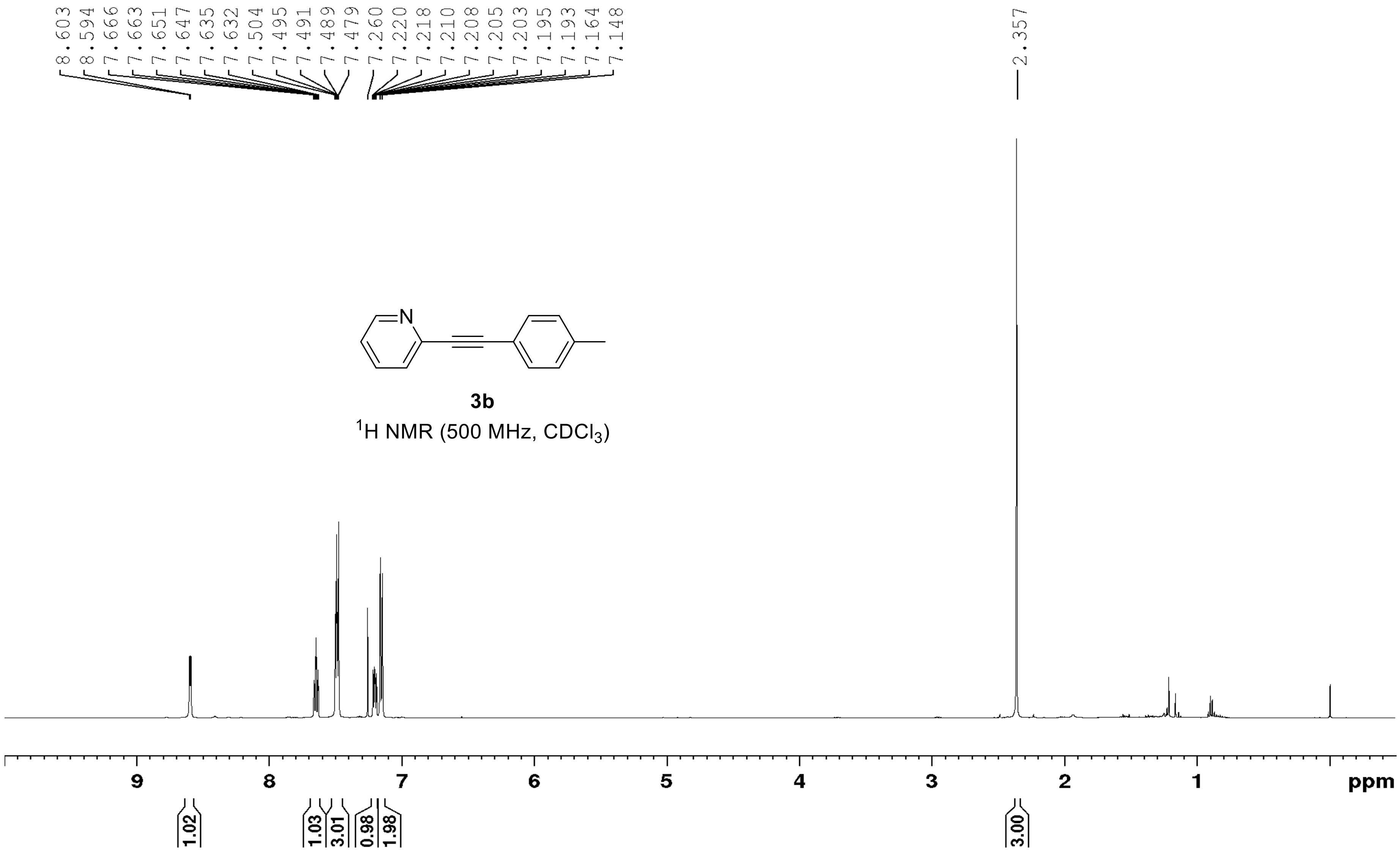
8.603
8.594
7.666
7.663
7.651
7.647
7.635
7.632
7.504
7.495
7.491
7.489
7.479
7.260
7.220
7.218
7.210
7.208
7.205
7.203
7.195
7.193
7.164
7.148
2.357
N
3b
^{1}H NMR (500 MHz, $CDCl_3$)
9
8
7
6
5
4
3
2
1
ppm
1.02
1.03
3.01
0.98
1.98
3.00

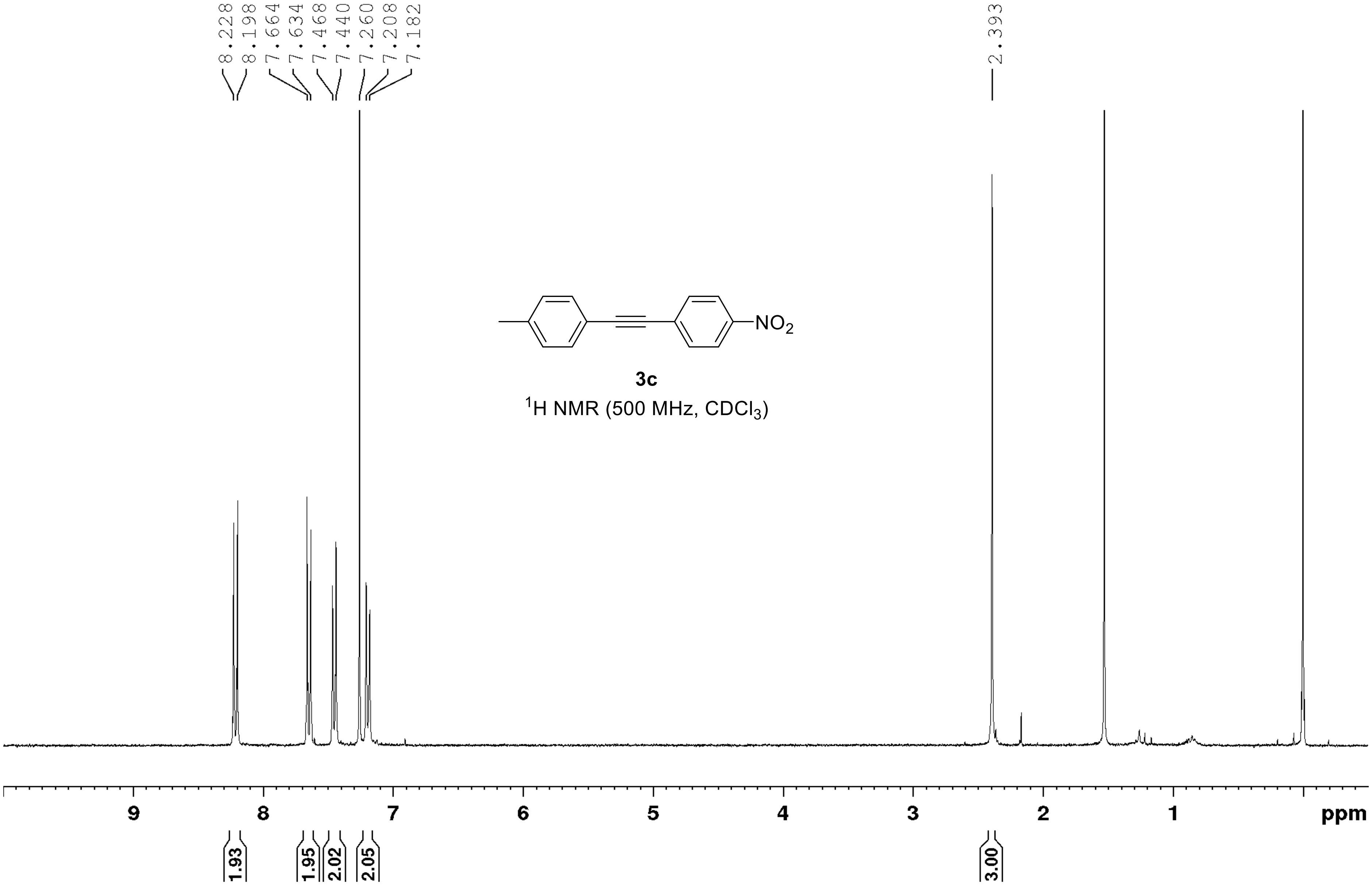

3c

^{1}H NMR (500 MHz, $CDCl_3$)

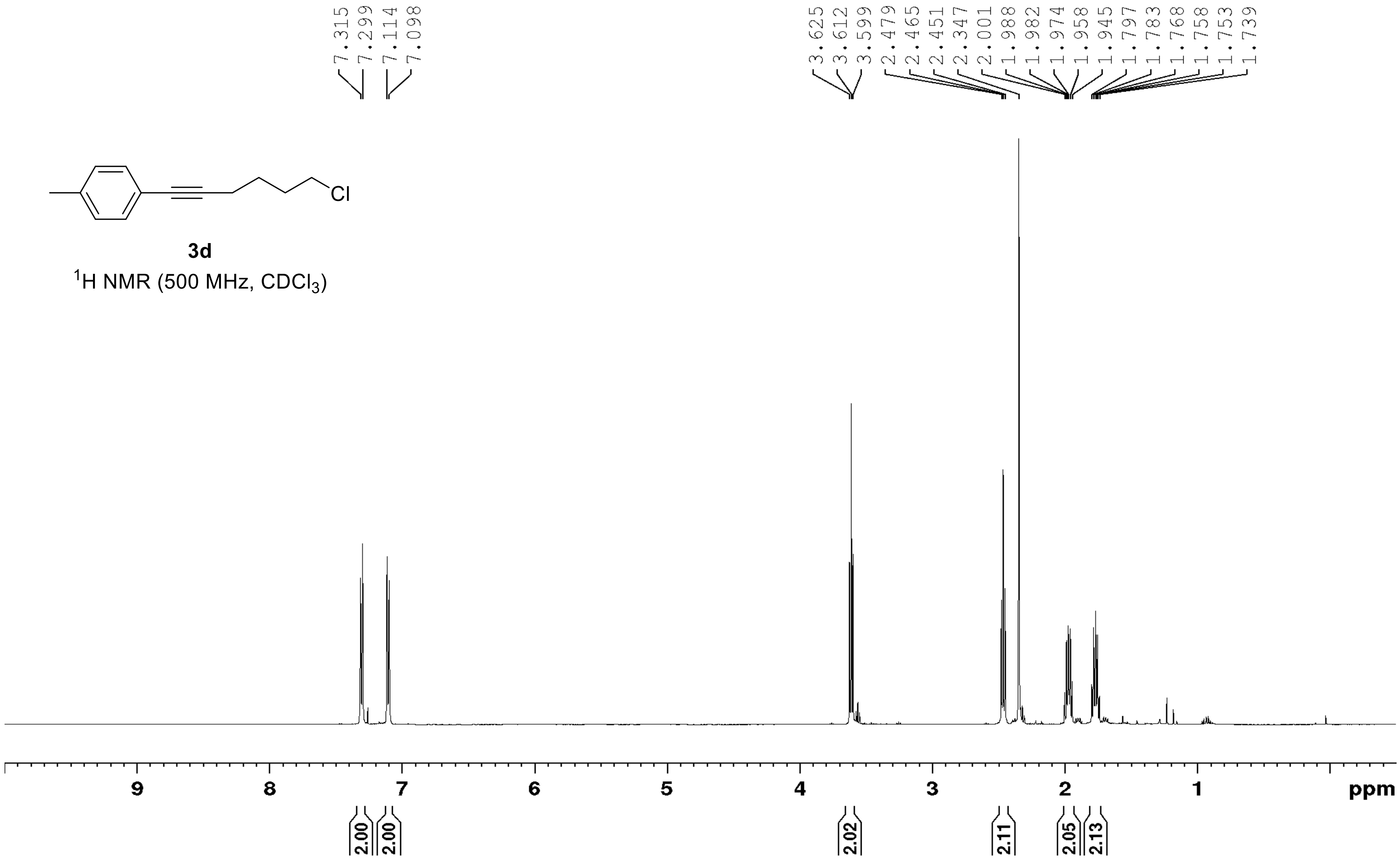

3d

^{1}H NMR (500 MHz, $CDCl_3$)

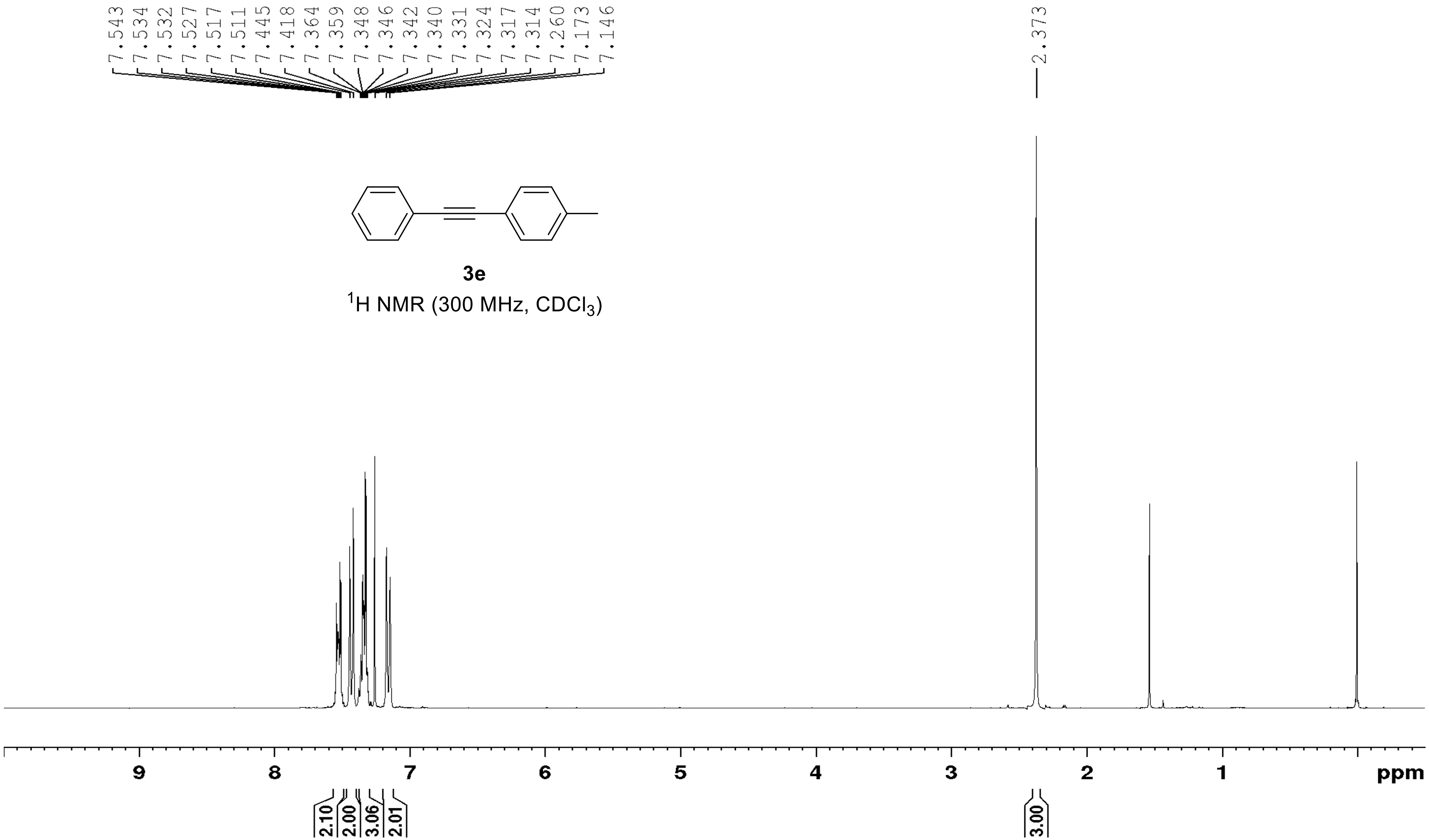
7.543
7.534
7.532
7.527
7.517
7.511
7.445
7.418
7.364
7.359
7.348
7.346
7.342
7.340
7.331
7.324
7.317
7.314
7.260
7.173
7.146
2.373
3e
1H NMR (300 MHz, CDCl3)
9
8
7
6
5
4
3
2
1
ppm
2.10
2.00
3.06
2.01
3.00

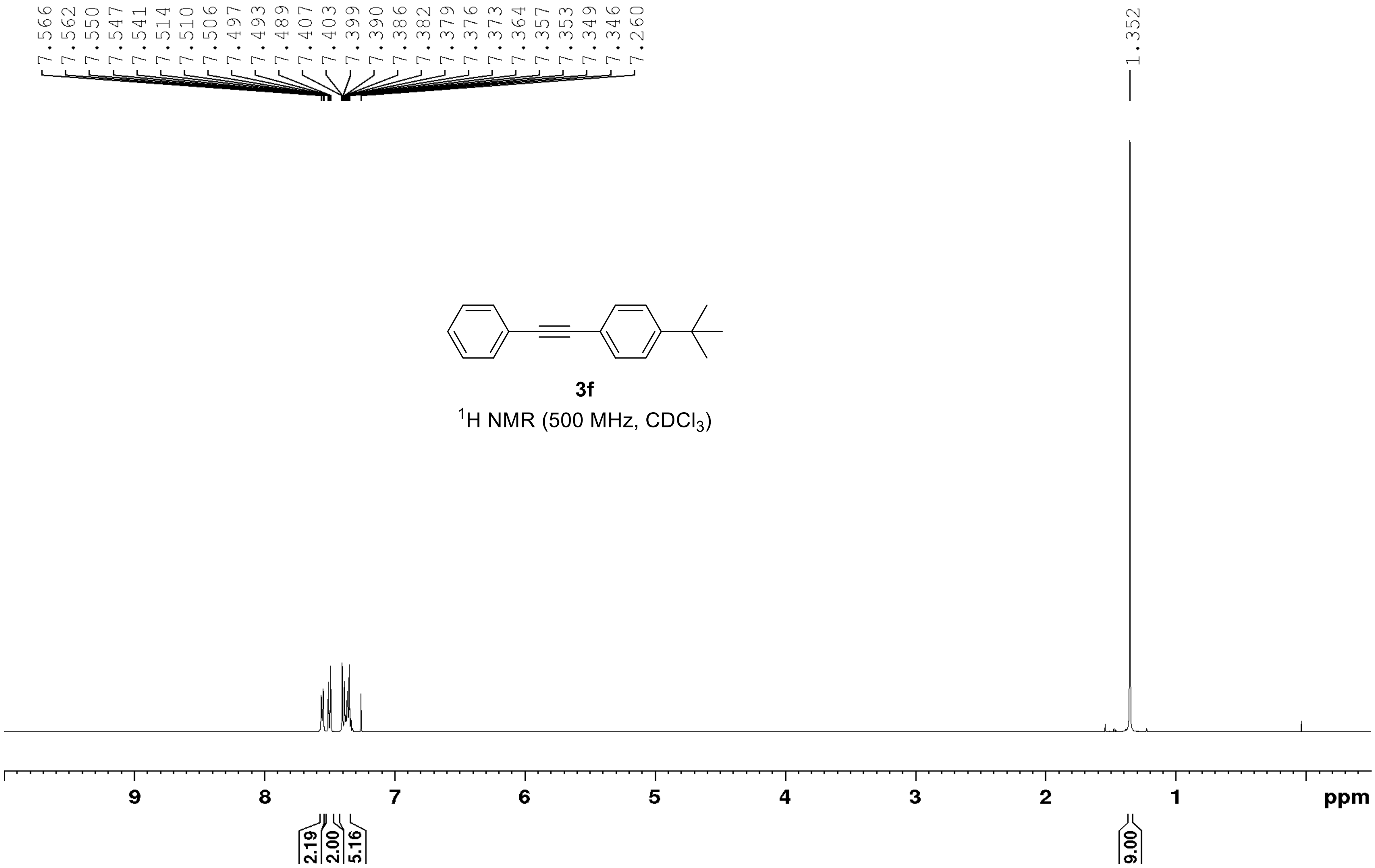
7.566
7.562
7.550
7.547
7.541
7.514
7.510
7.506
7.497
7.493
7.489
7.407
7.403
7.399
7.390
7.386
7.382
7.379
7.376
7.373
7.364
7.357
7.353
7.349
7.346
7.260
1.352
3f
^{1}H NMR (500 MHz, $CDCl_3$)
9
8
7
6
5
4
3
2
1
ppm
2.19
2.00
5.16
9.00

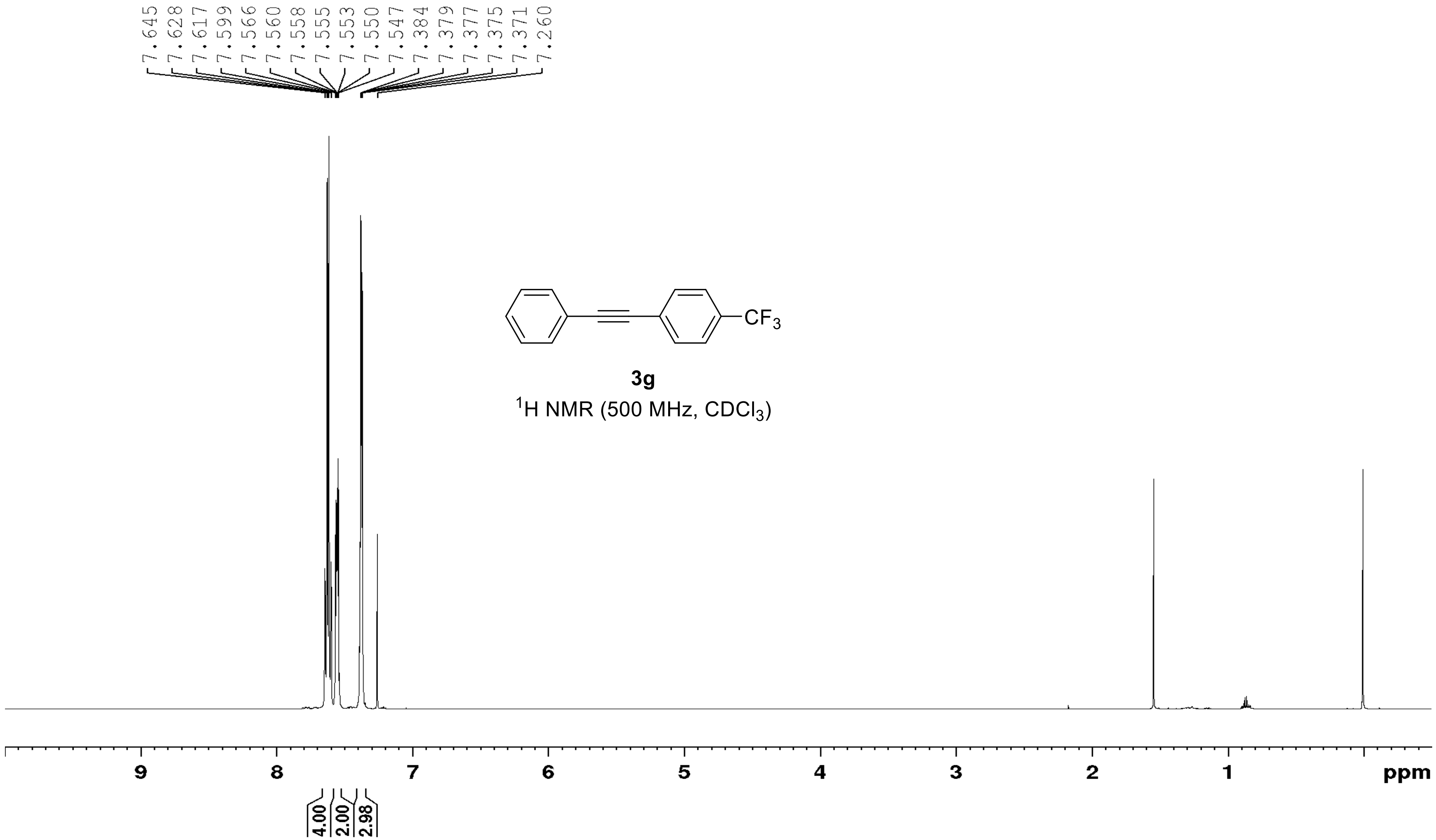
7.645
7.628
7.617
7.599
7.566
7.560
7.558
7.555
7.553
7.550
7.547
7.384
7.379
7.377
7.375
7.371
7.260
CF3
3g
1H NMR (500 MHz, CDCl3)
9
8
7
6
5
4
3
2
1
ppm
4.00
2.00
2.98

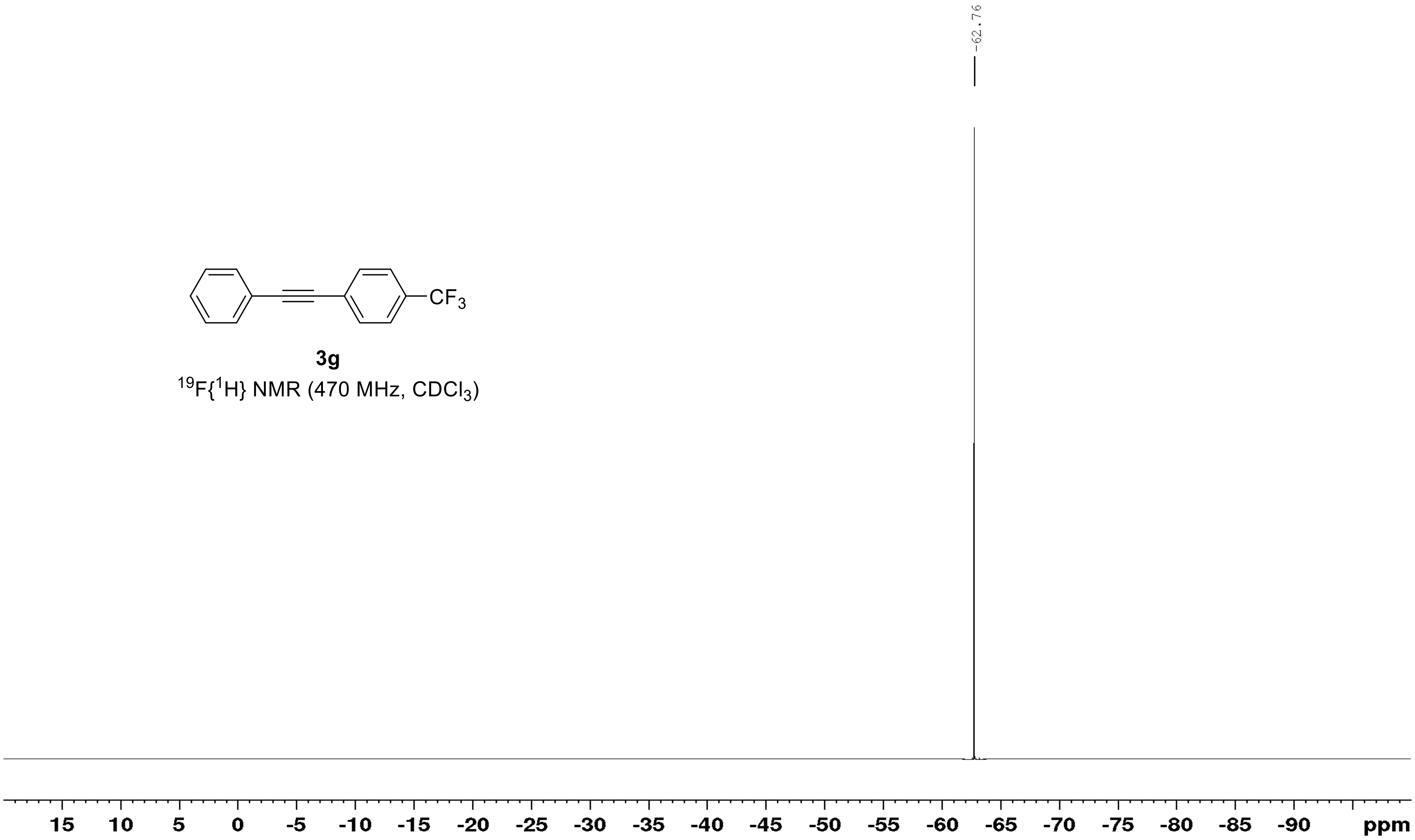

3g

$^{19}F\{^1H\}$ NMR (470 MHz, $CDCl_3$)

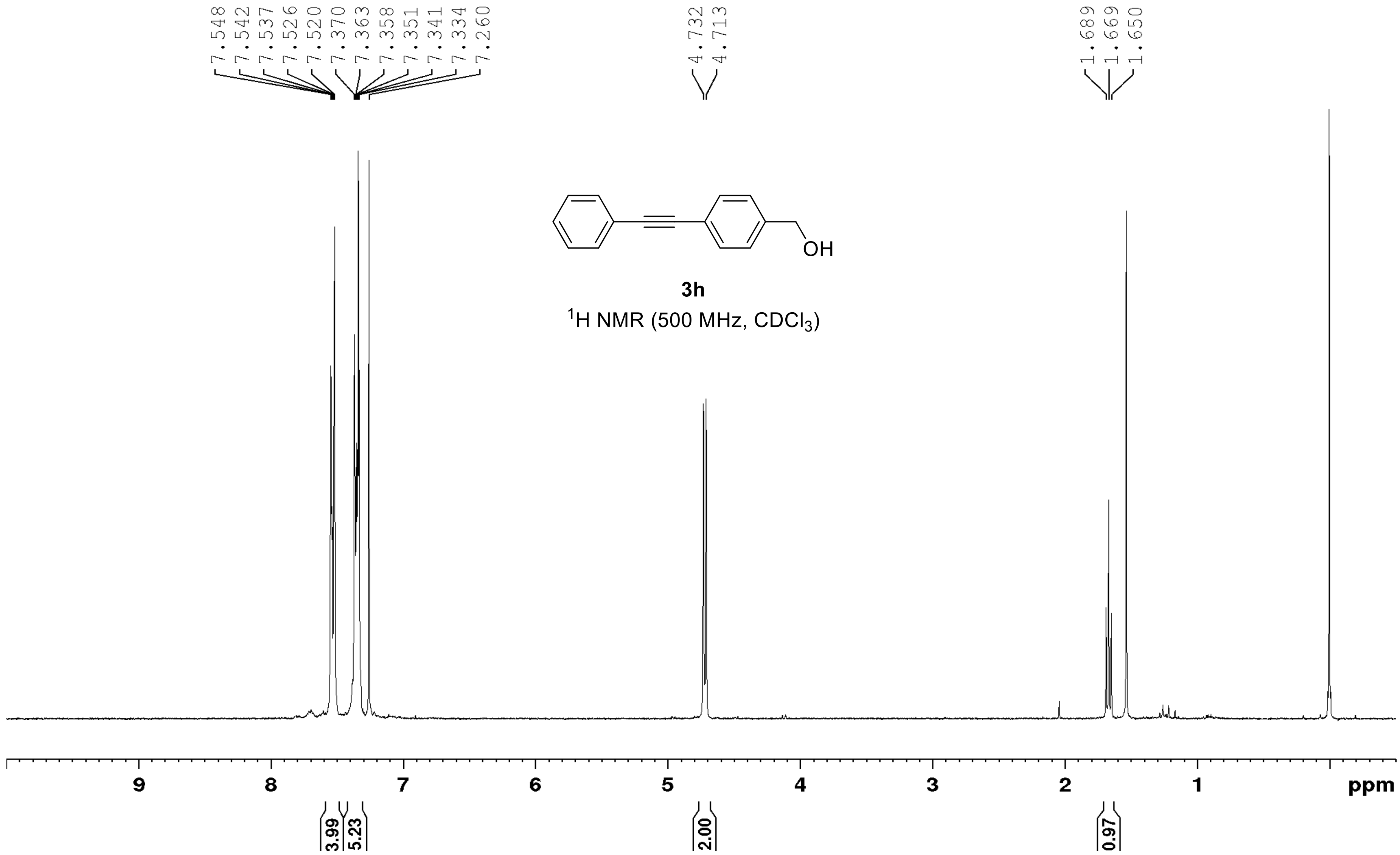
7.548
7.542
7.537
7.526
7.520
7.370
7.363
7.358
7.351
7.341
7.334
7.260
4.732
4.713
1.689
1.669
1.650
OH
3h
1H NMR (500 MHz, CDCl3)
9
8
7
6
5
4
3
2
1
ppm
3.99
5.23
2.00
0.97

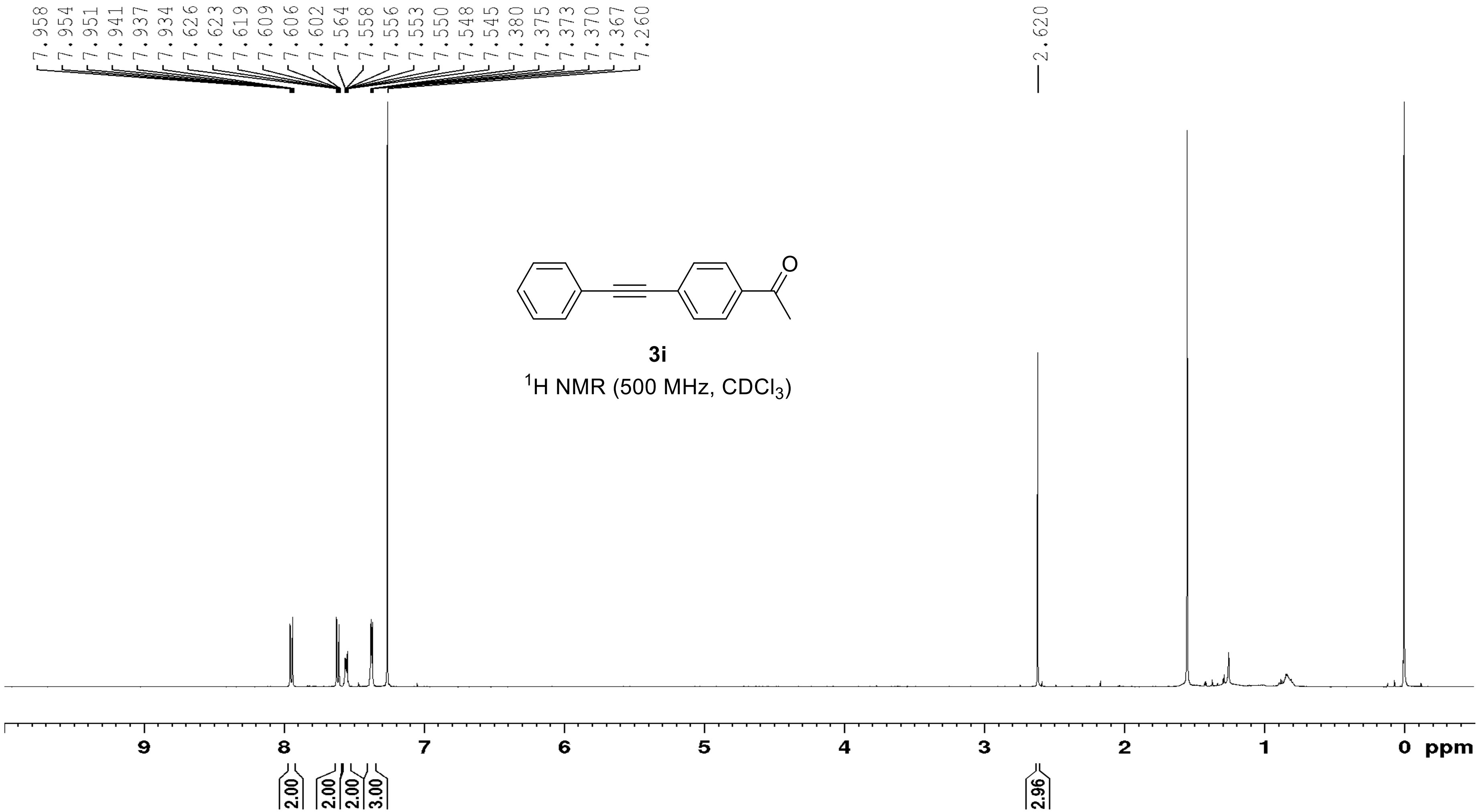
7.958
7.954
7.951
7.941
7.937
7.934
7.626
7.623
7.619
7.609
7.606
7.602
7.564
7.558
7.558
7.556
7.553
7.550
7.548
7.545
7.380
7.375
7.373
7.370
7.367
7.260
2.620
O
3i
1H NMR (500 MHz, CDCl3)
9
8
7
6
5
4
3
2
1
0 ppm
2.00
2.00
2.00
3.00
2.96

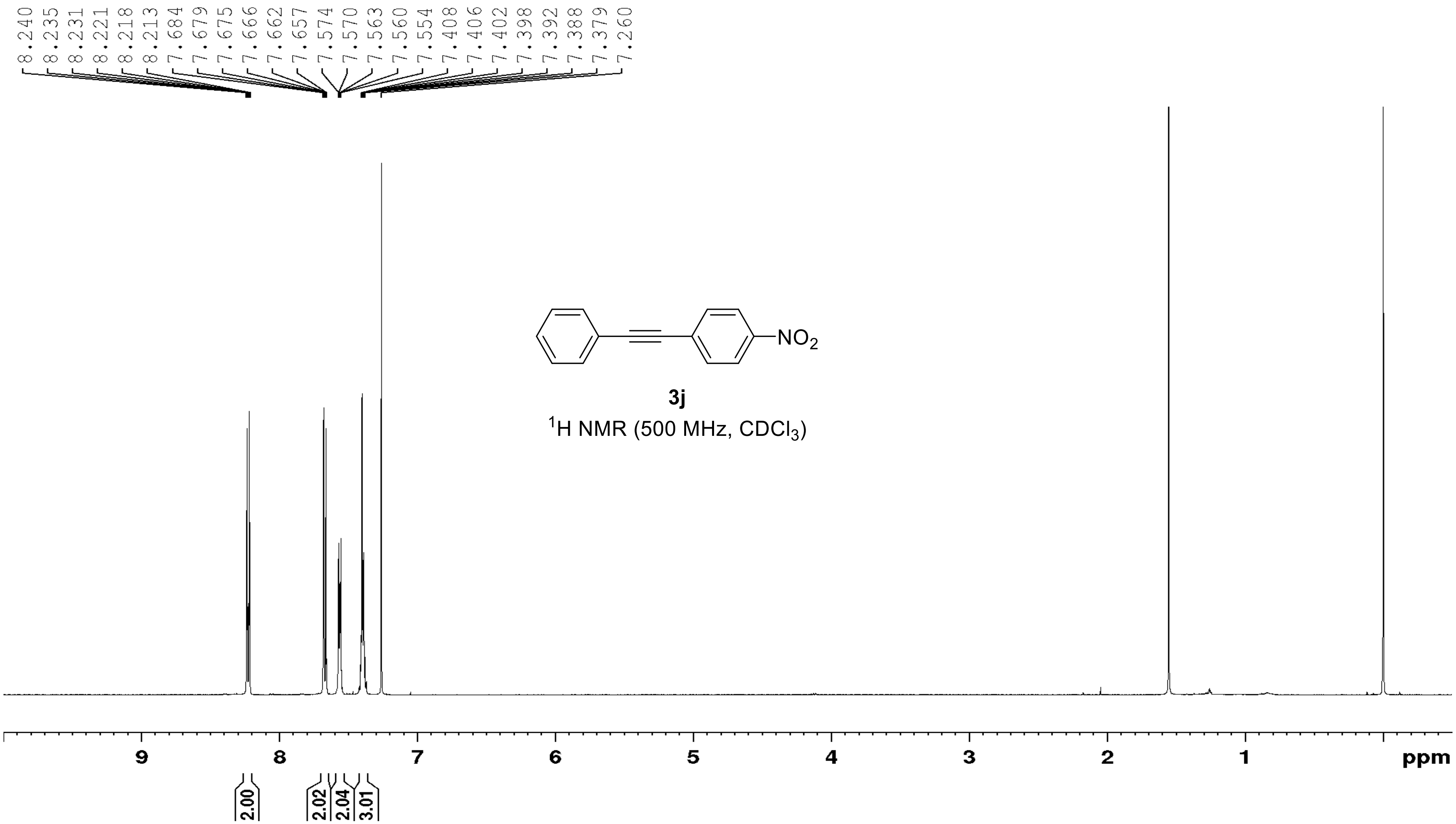
8.240
8.235
8.231
8.221
8.218
8.213
7.684
7.679
7.675
7.666
7.662
7.657
7.574
7.570
7.563
7.560
7.554
7.408
7.406
7.402
7.398
7.392
7.388
7.379
7.260
NO2
3j
1H NMR (500 MHz, CDCl3)
9
8
7
6
5
4
3
2
1
ppm
2.00
2.02
2.04
3.01

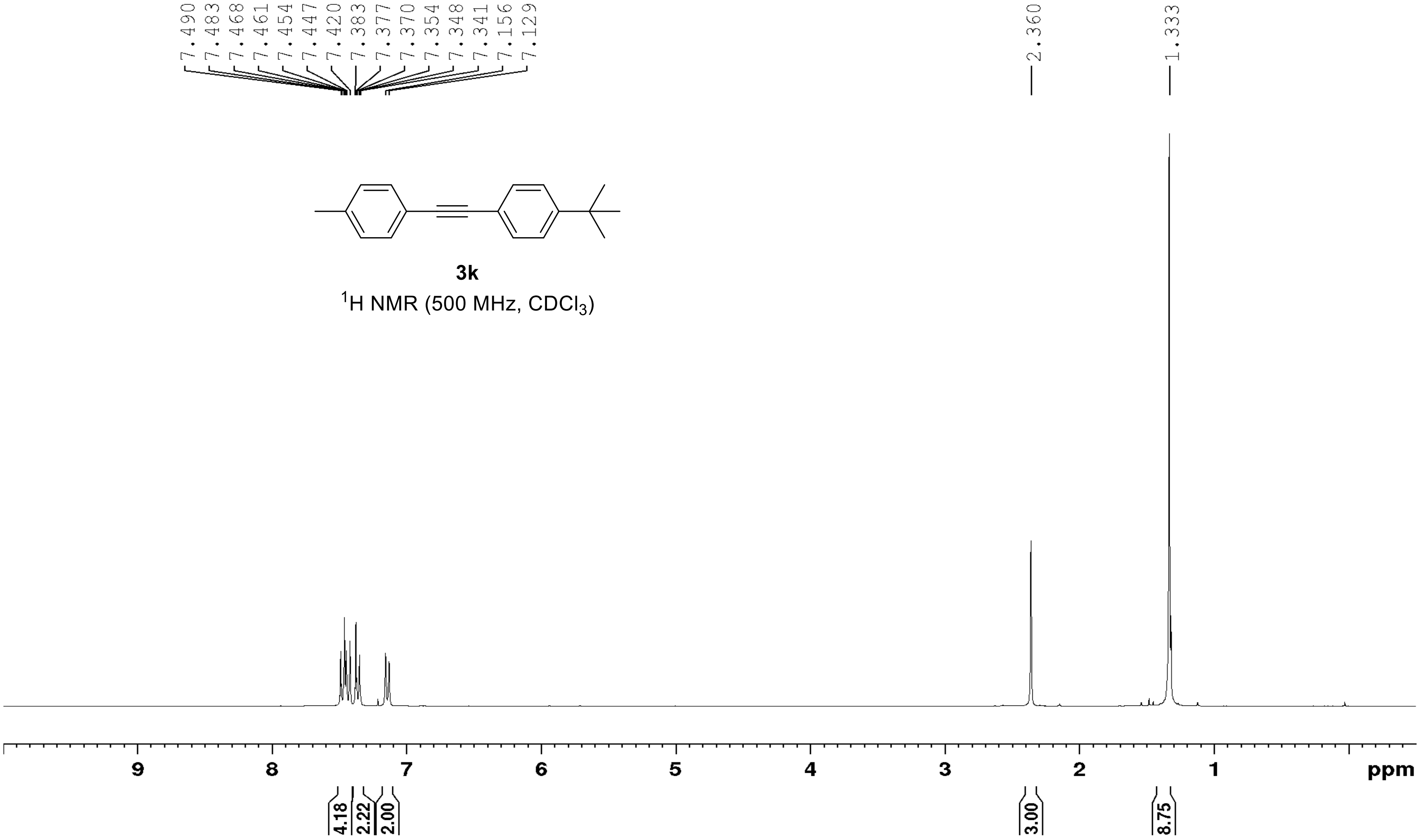

3k

^{1}H NMR (500 MHz, $CDCl_3$)

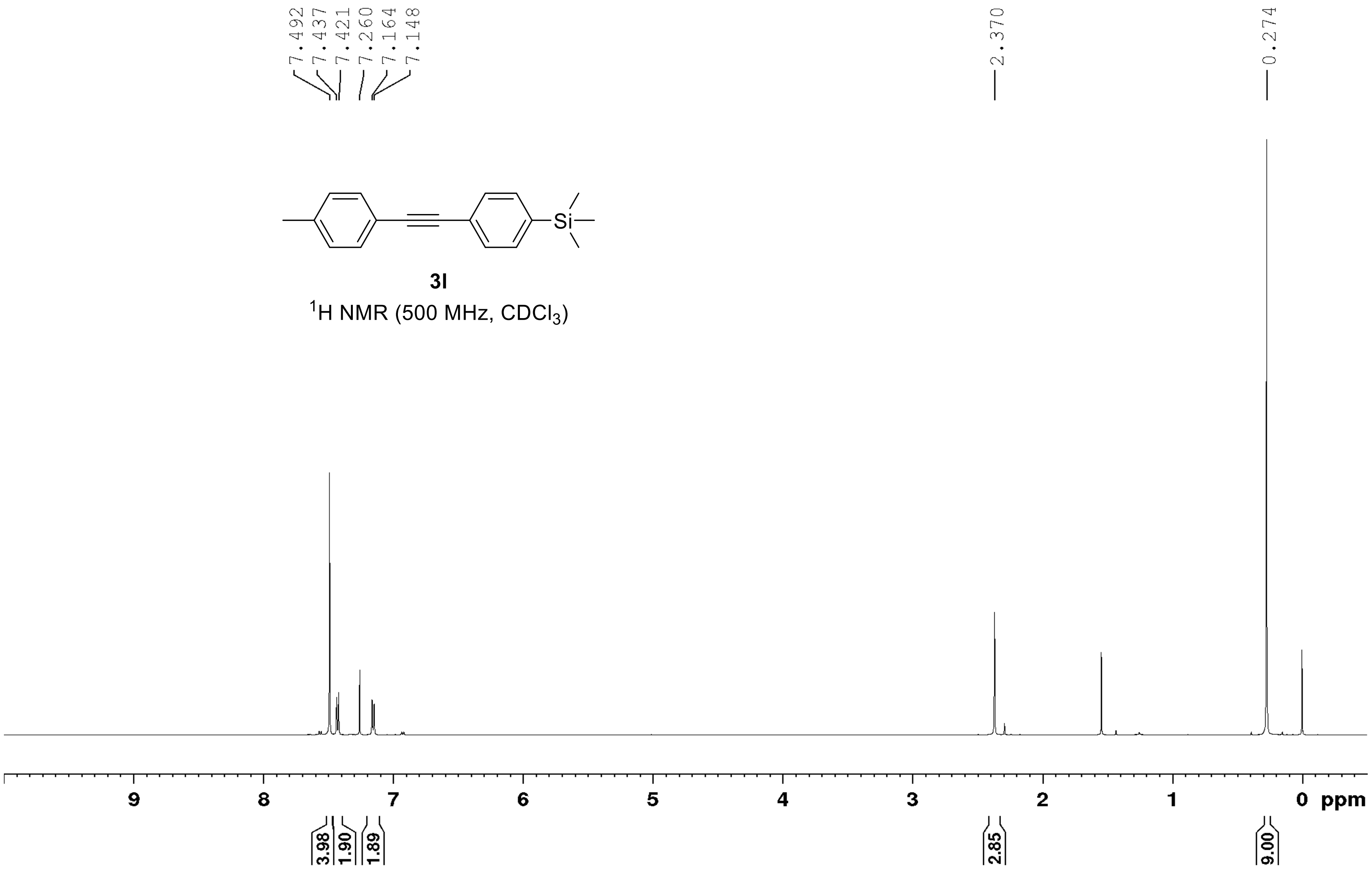
7.492
7.437
7.421
7.260
7.164
7.148
2.370
0.274
Si
3l
^{1}H NMR (500 MHz, CDCl$_3$)
9
8
7
6
5
4
3
2
1
0 ppm
3.98
1.90
1.89
2.85
9.00

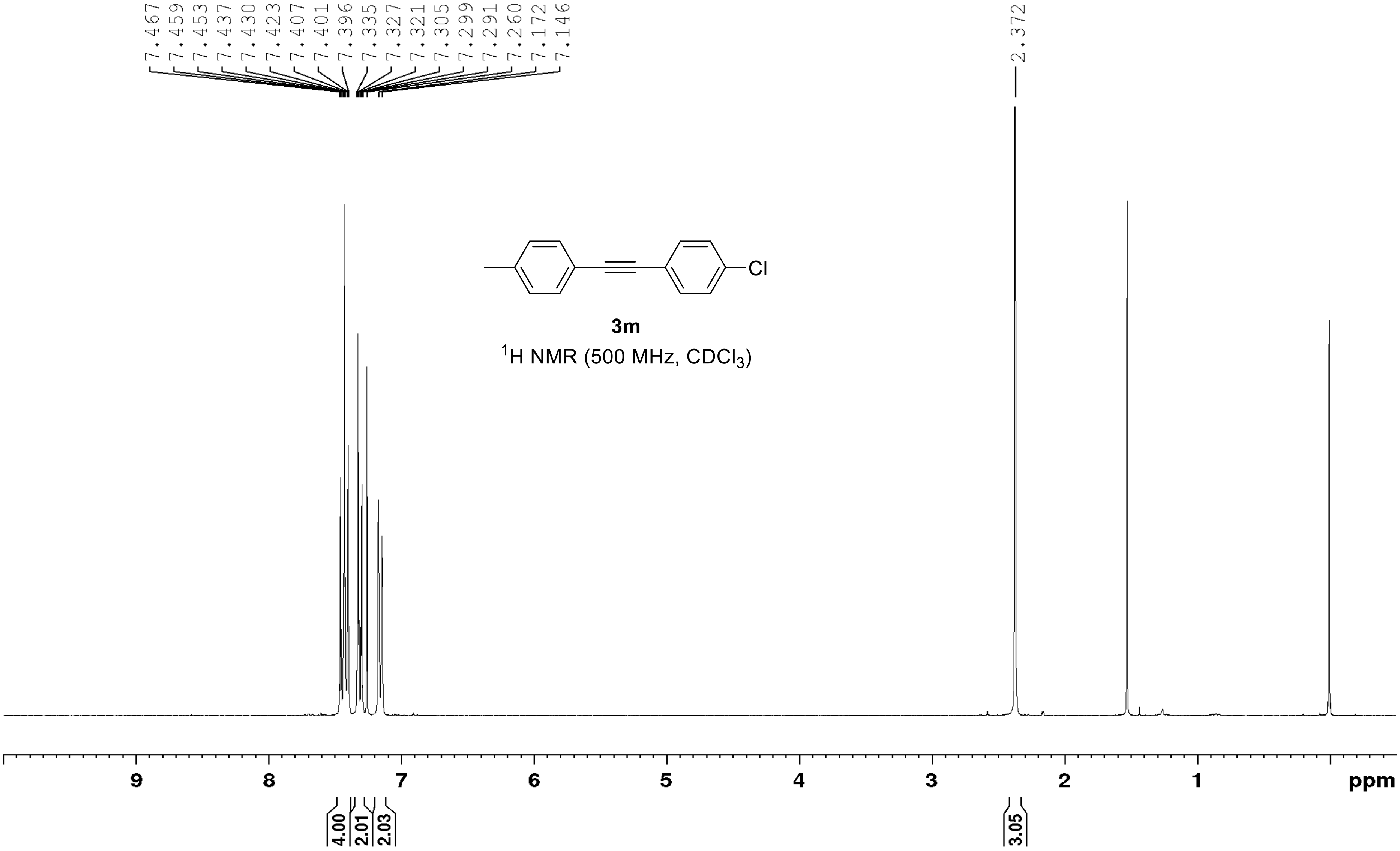
7.467
7.459
7.453
7.437
7.430
7.423
7.407
7.401
7.396
7.335
7.327
7.321
7.305
7.299
7.291
7.260
7.172
7.146
2.372
Cl
3m
1H NMR (500 MHz, CDCl3)
9
8
7
6
5
4
3
2
1
ppm
4.00
2.01
2.03
3.05

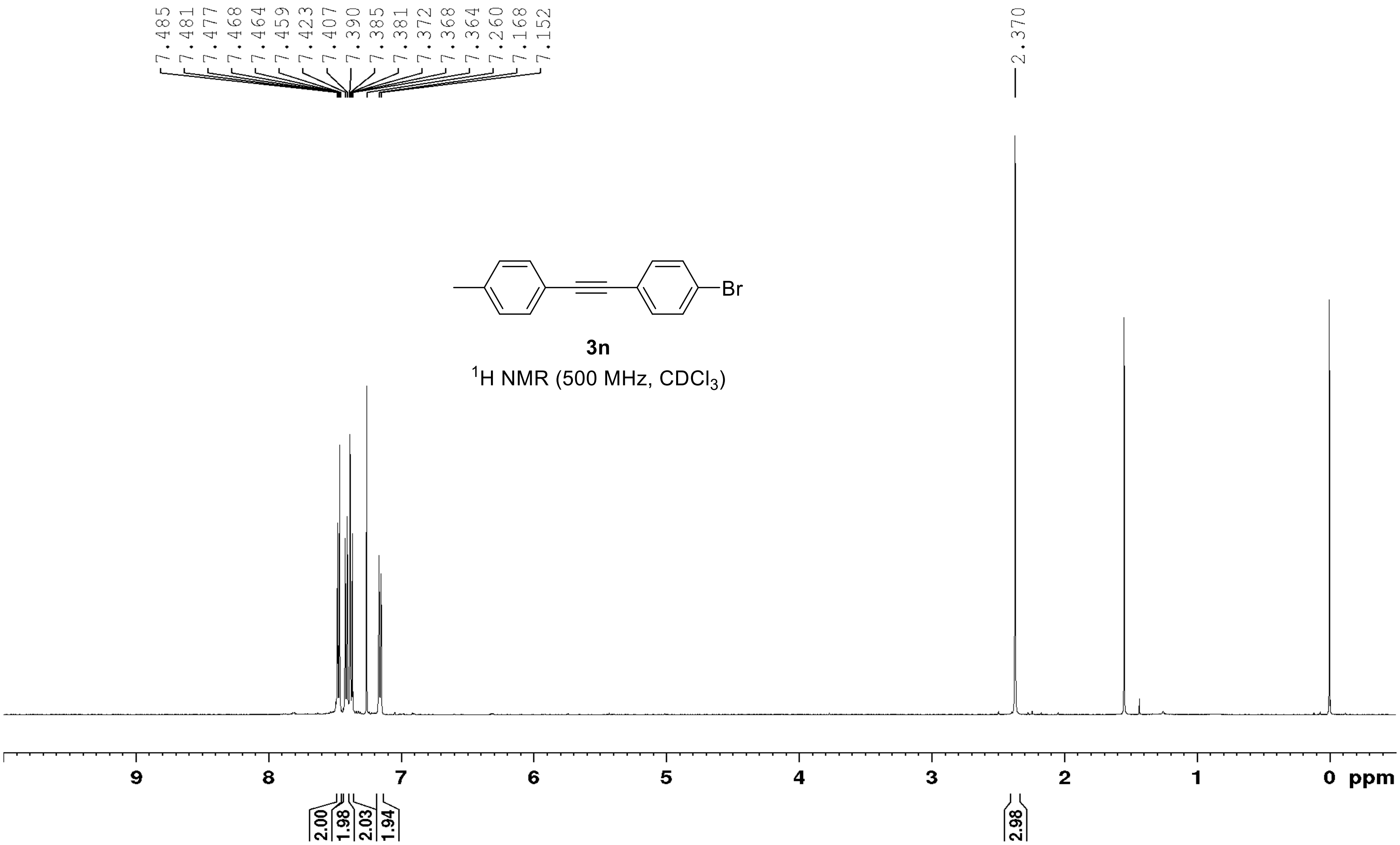
7.485
7.481
7.477
7.468
7.464
7.459
7.423
7.407
7.390
7.385
7.381
7.372
7.368
7.364
7.260
7.168
7.152
2.370
Br
3n
1H NMR (500 MHz, CDCl3)
9
8
7
6
5
4
3
2
1
0
ppm
2.00
1.98
2.03
1.94
2.98

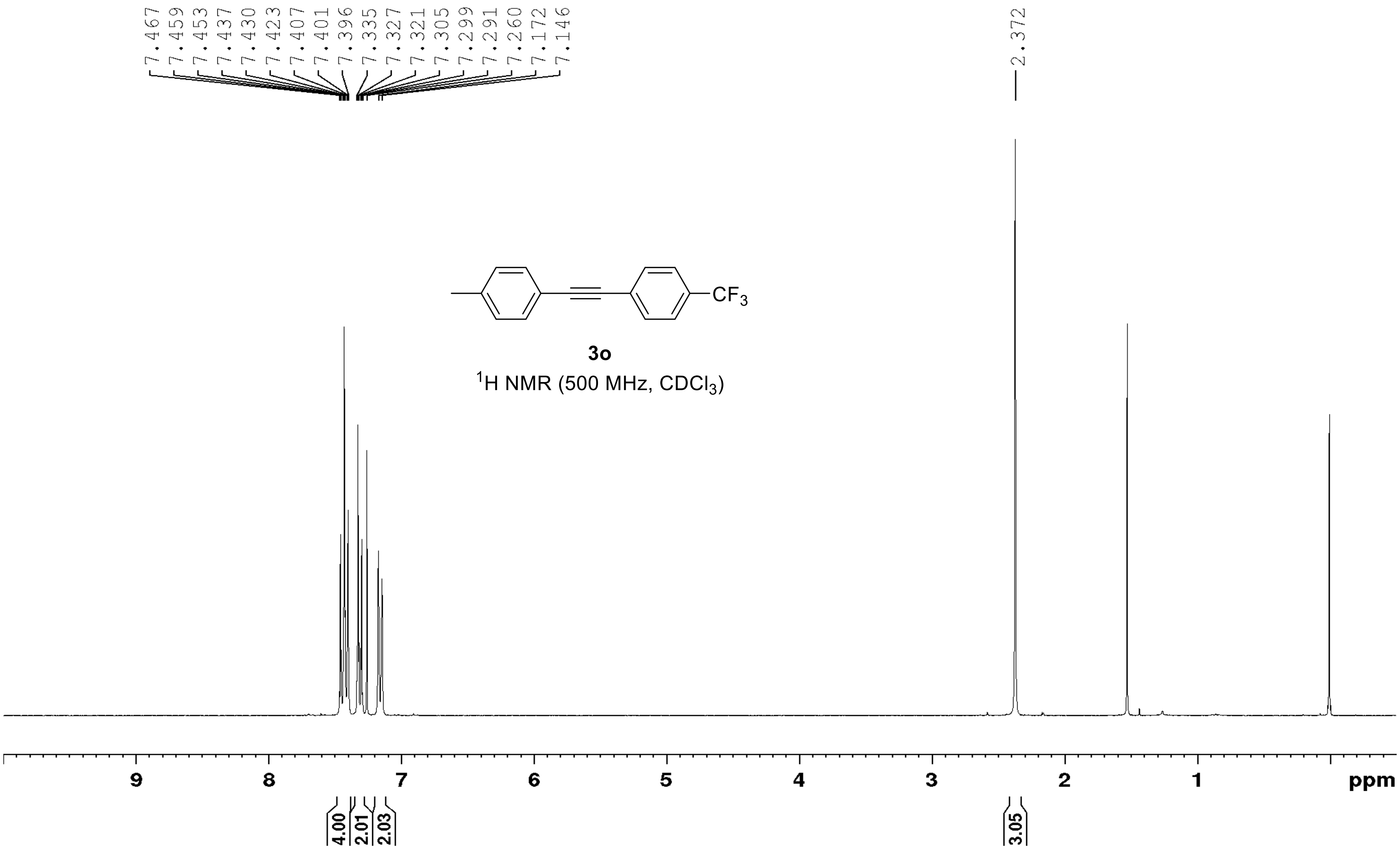

7.467
7.459
7.453
7.437
7.430
7.423
7.407
7.401
7.396
7.335
7.327
7.321
7.305
7.299
7.291
7.260
7.172
7.146
2.372
CF3
3o
1H NMR (500 MHz, CDCl3)
9
8
7
6
5
4
3
2
1
ppm
4.00
2.01
2.03
3.05

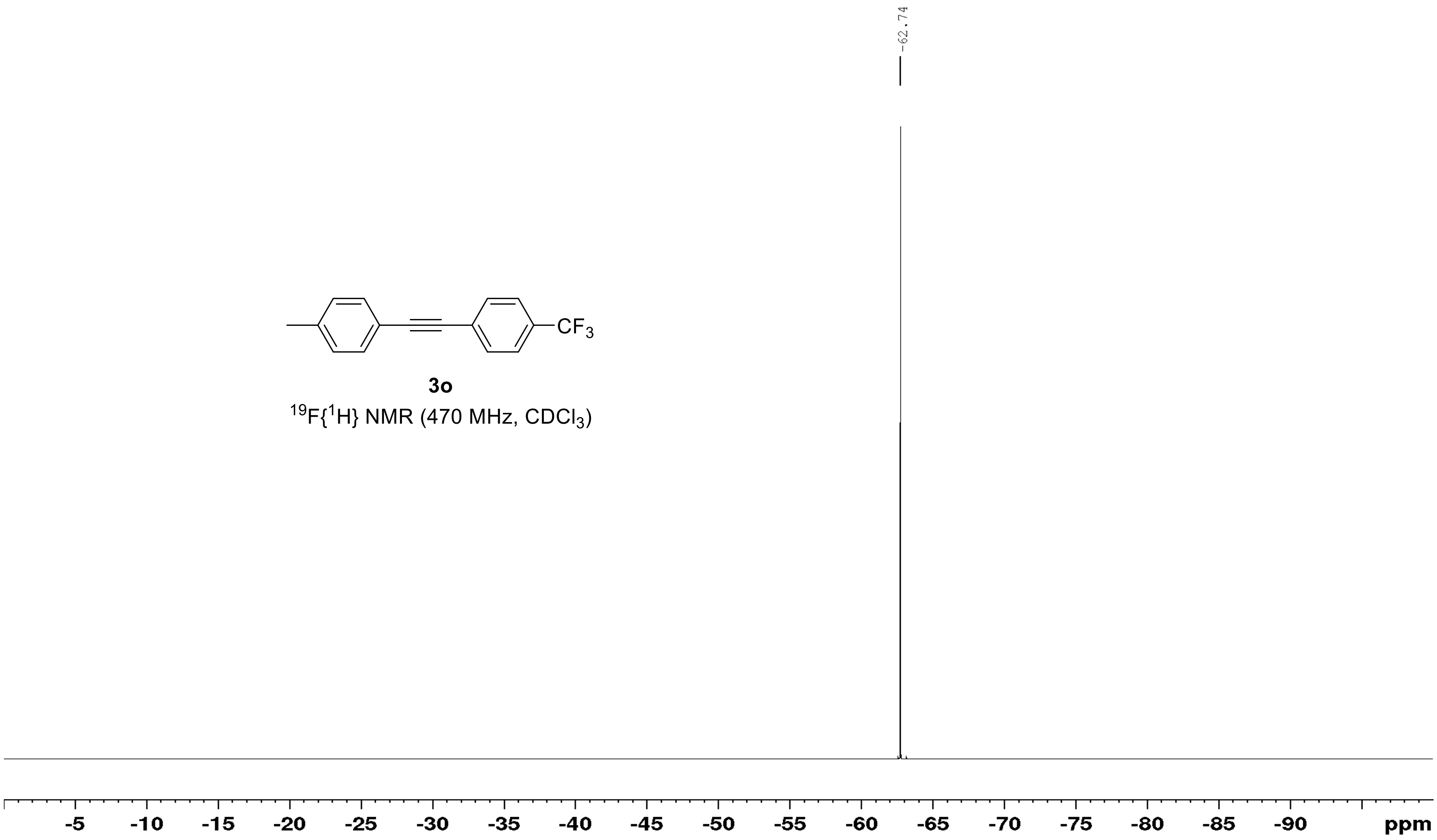
-62.74
CF3
3o
19F{1H} NMR (470 MHz, CDCl3)
-5 -10 -15 -20 -25 -30 -35 -40 -45 -50 -55 -60 -65 -70 -75 -80 -85 -90 ppm

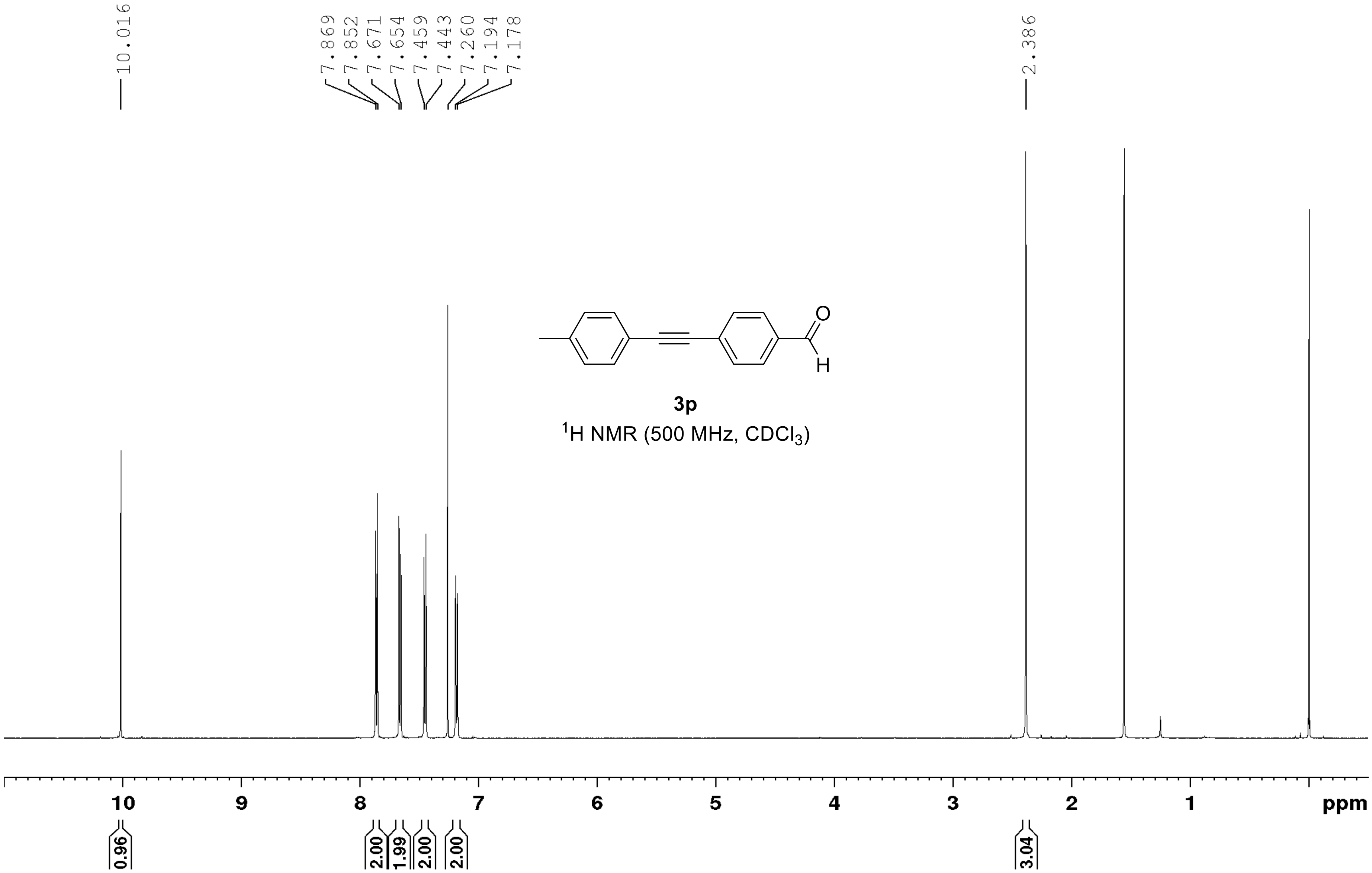

3p

^{1}H NMR (500 MHz, $CDCl_3$)

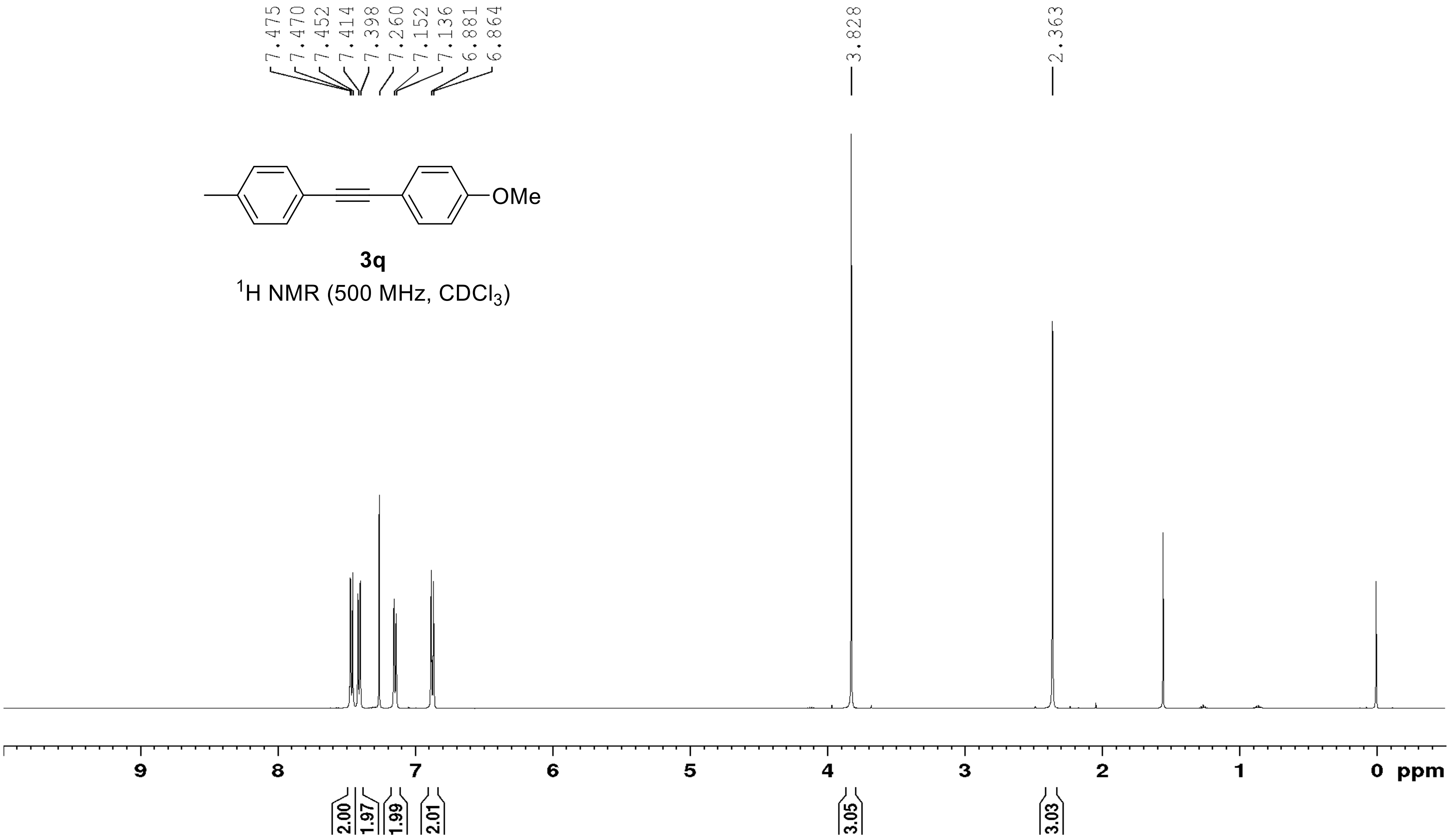

3q

^{1}H NMR (500 MHz, $CDCl_3$)

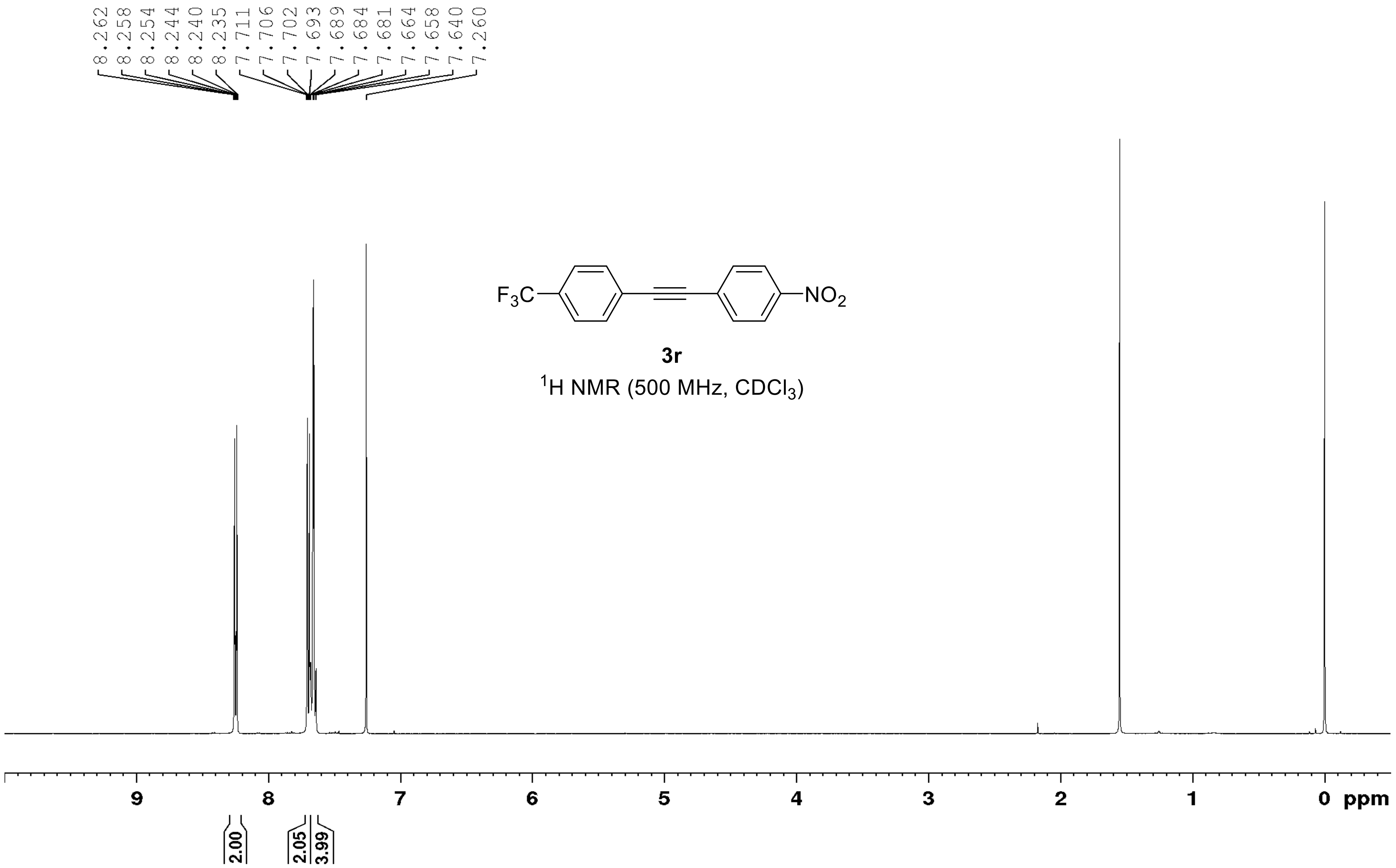
8.262
8.258
8.254
8.244
8.240
8.235
7.711
7.706
7.702
7.693
7.689
7.684
7.681
7.664
7.658
7.640
7.260
F3C
NO2
3r
1H NMR (500 MHz, CDCl3)
9
8
7
6
5
4
3
2
1
0 ppm
2.00
2.05
3.99

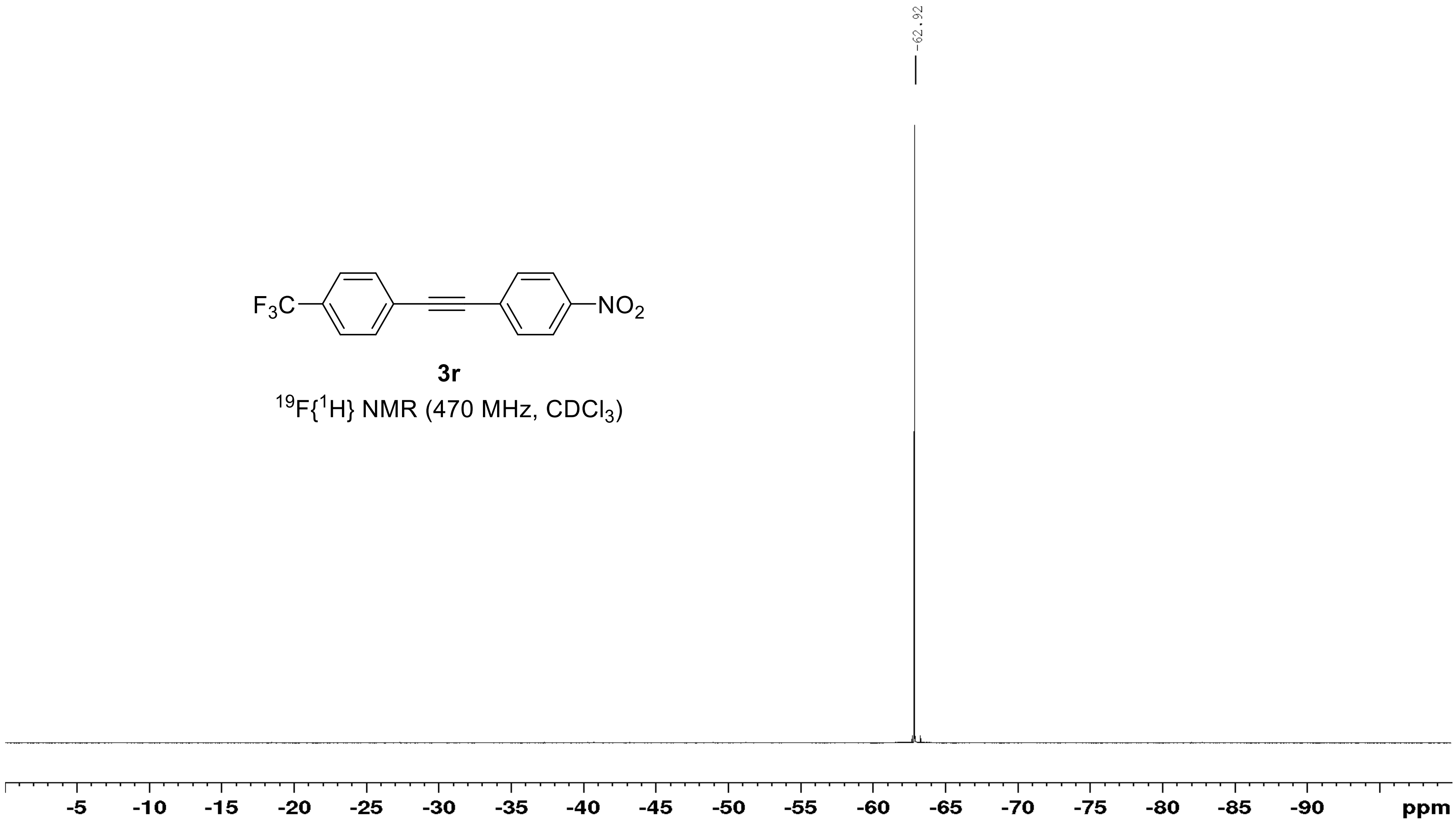

3r

$^{19}F\{^{1}H\}$ NMR (470 MHz, $CDCl_3$)

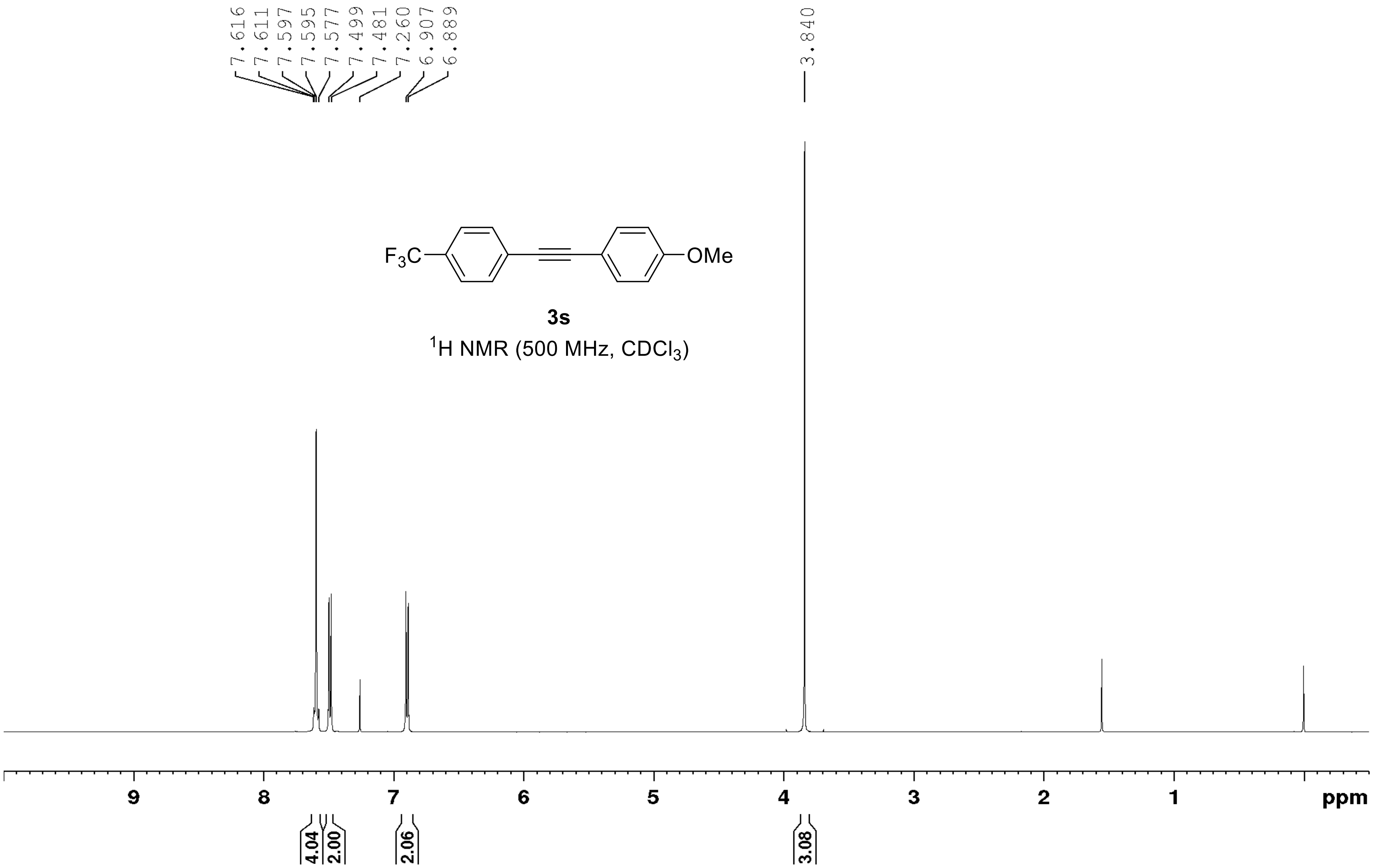

3s

^{1}H NMR (500 MHz, $CDCl_3$)

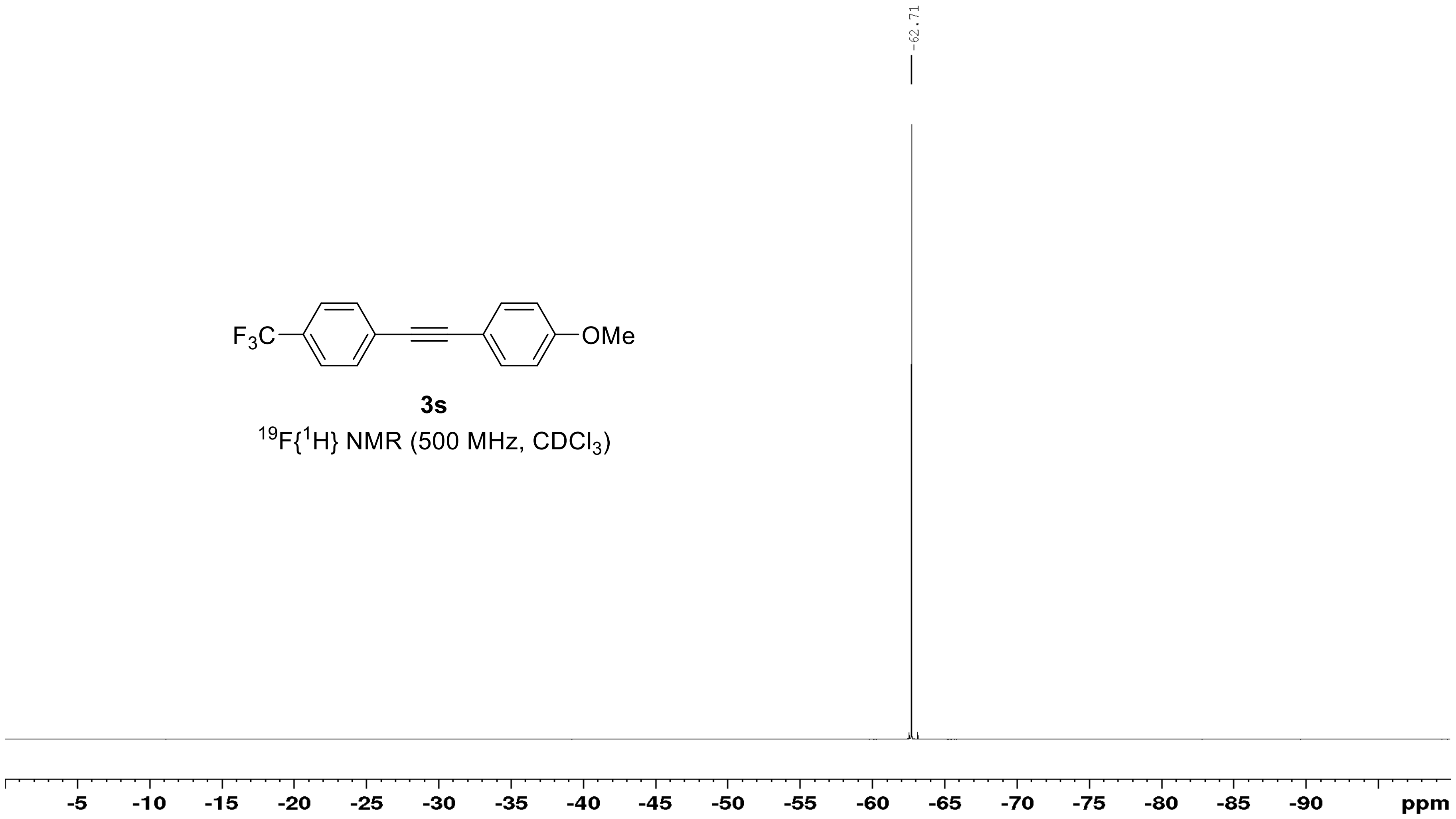
-62.71
F3C
OMe
3s
19F{1H} NMR (500 MHz, CDCl3)
-5 -10 -15 -20 -25 -30 -35 -40 -45 -50 -55 -60 -65 -70 -75 -80 -85 -90 ppm

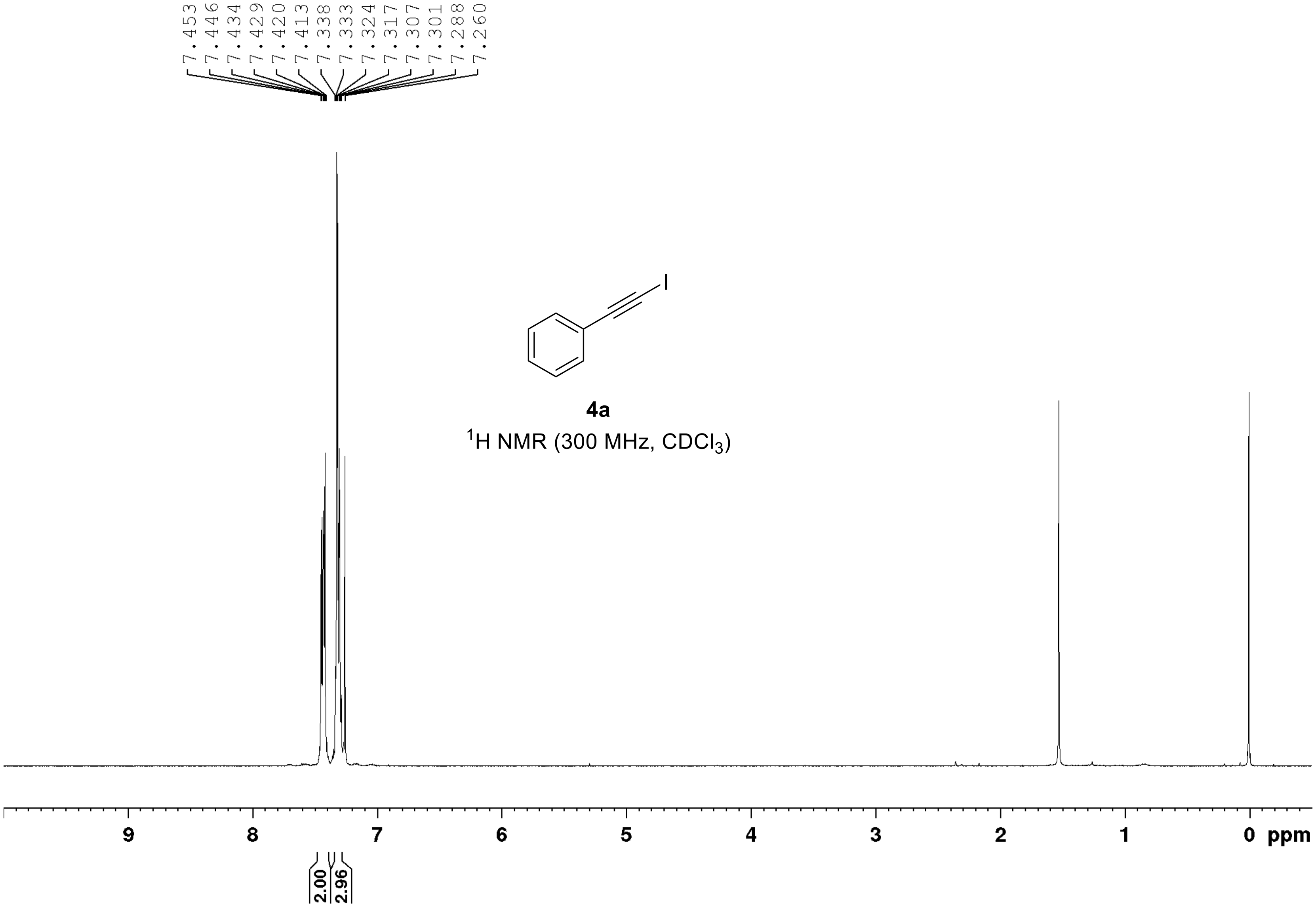
7.453
7.446
7.434
7.429
7.420
7.413
7.338
7.333
7.324
7.317
7.307
7.301
7.288
7.260
I
4a
1H NMR (300 MHz, CDCl3)
9
8
7
6
5
4
3
2
1
0 ppm
2.00
2.96

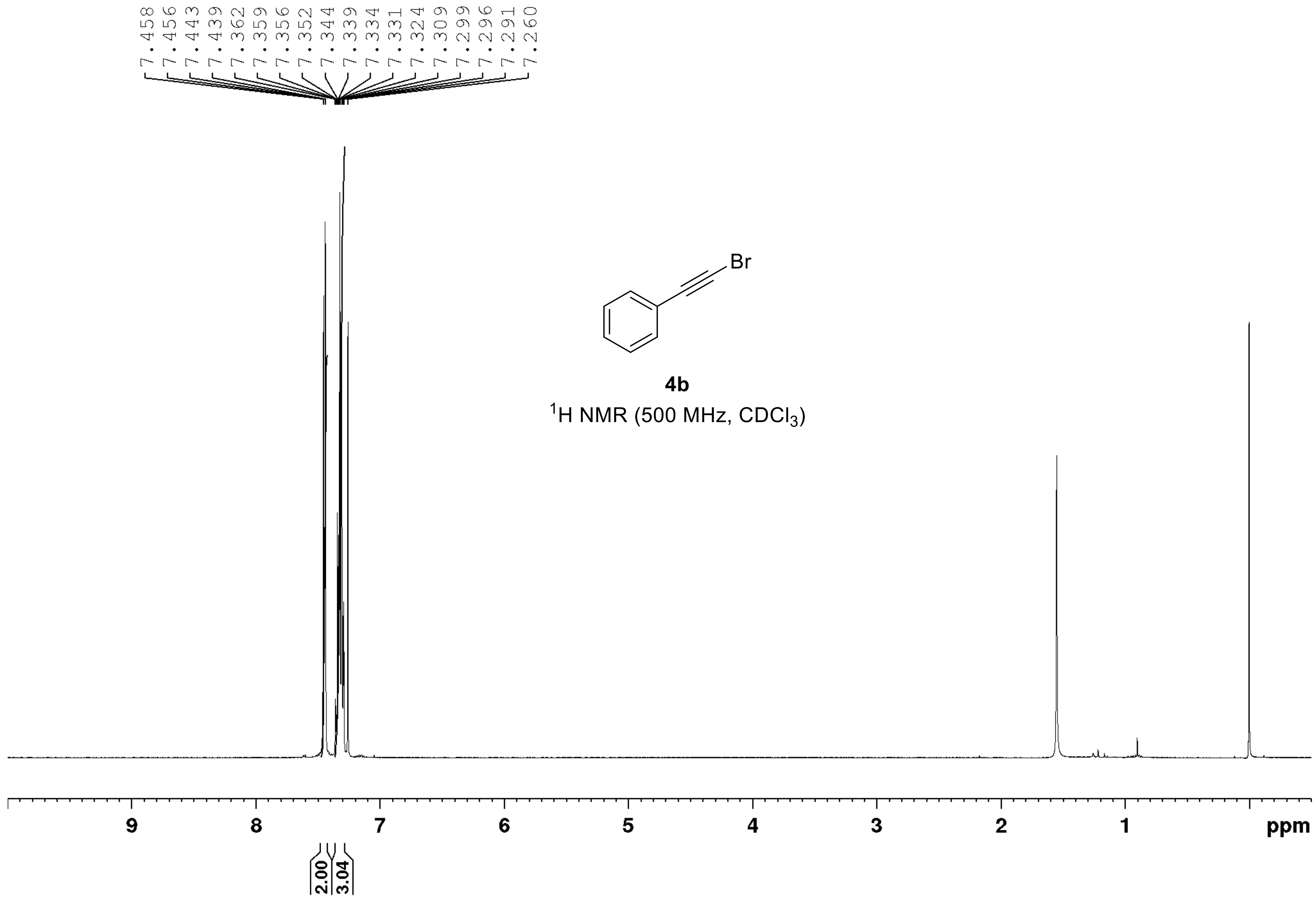
7.458
7.456
7.443
7.439
7.362
7.359
7.356
7.352
7.344
7.339
7.334
7.331
7.324
7.309
7.299
7.296
7.291
7.260
Br
4b
1H NMR (500 MHz, CDCl3)
9
8
7
6
5
4
3
2
1
ppm
2.00
3.04

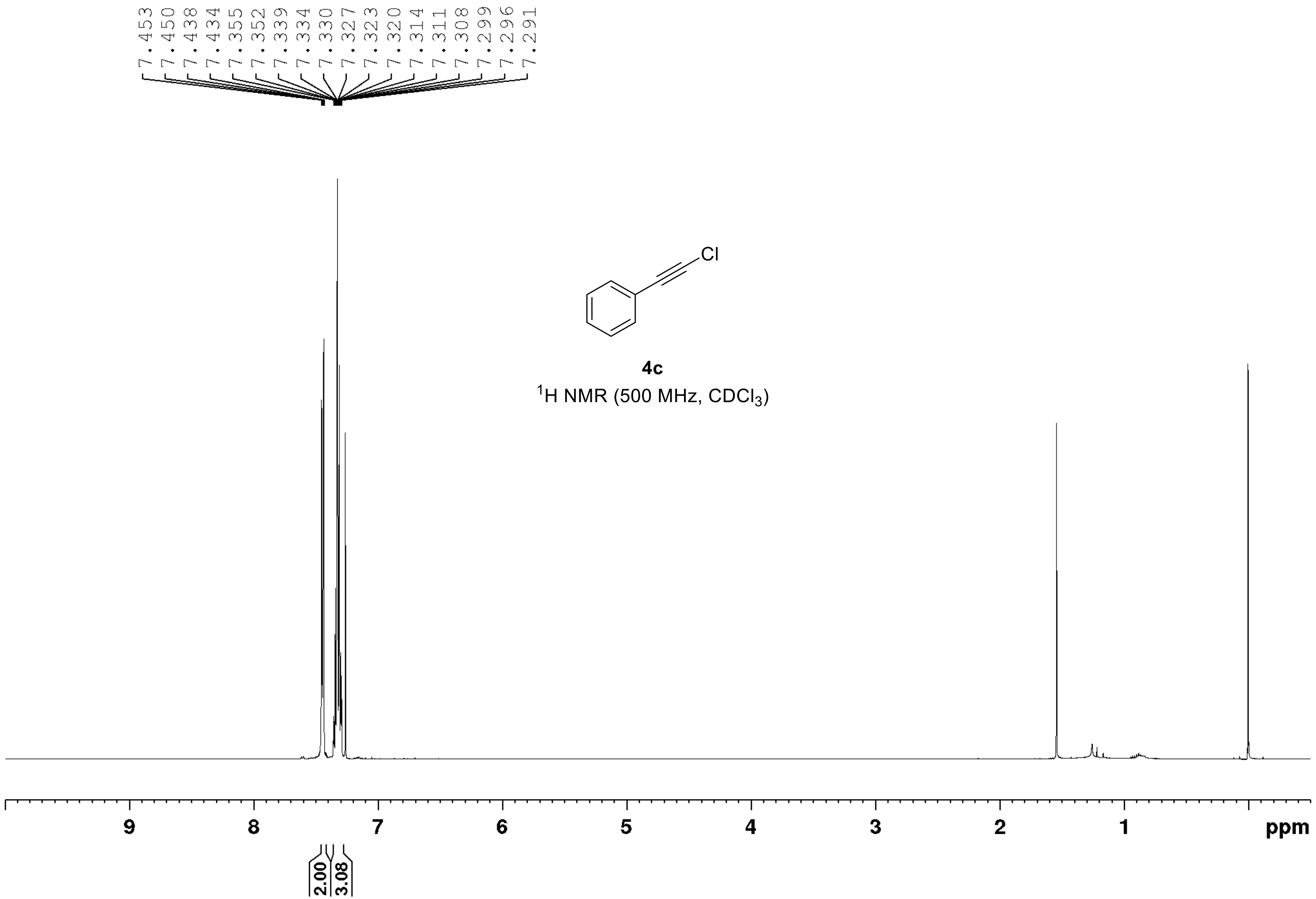
7.453
7.450
7.438
7.434
7.355
7.352
7.339
7.334
7.330
7.327
7.323
7.320
7.314
7.311
7.308
7.299
7.296
7.291
Cl
4c
^{1}H NMR (500 MHz, $CDCl_3$)
9
8
7
6
5
4
3
2
1
ppm
2.00
3.08

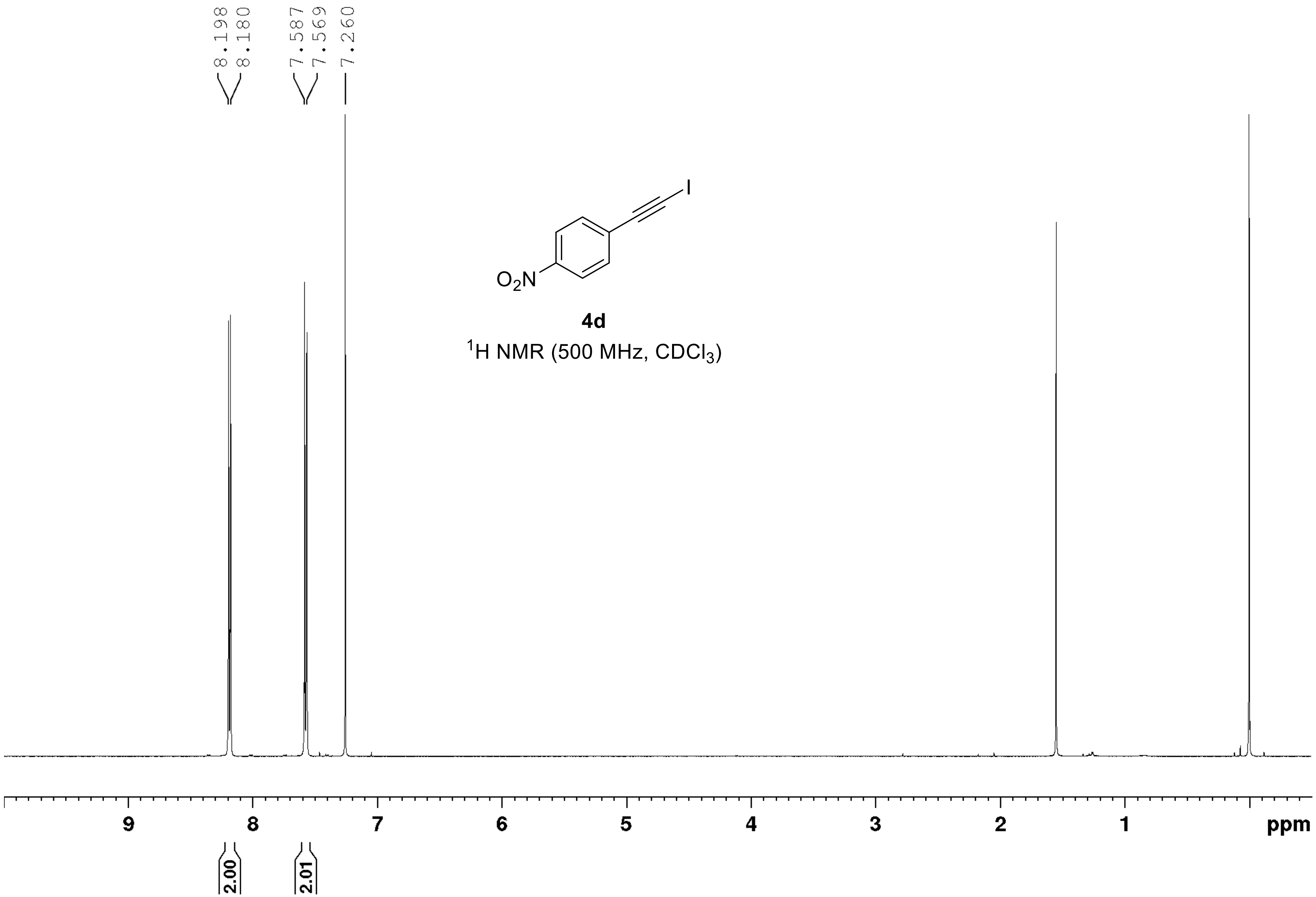
8.198
8.180
7.587
7.569
7.260
O2N
I
4d
1H NMR (500 MHz, CDCl3)
9
8
7
6
5
4
3
2
1
ppm
2.00
2.01

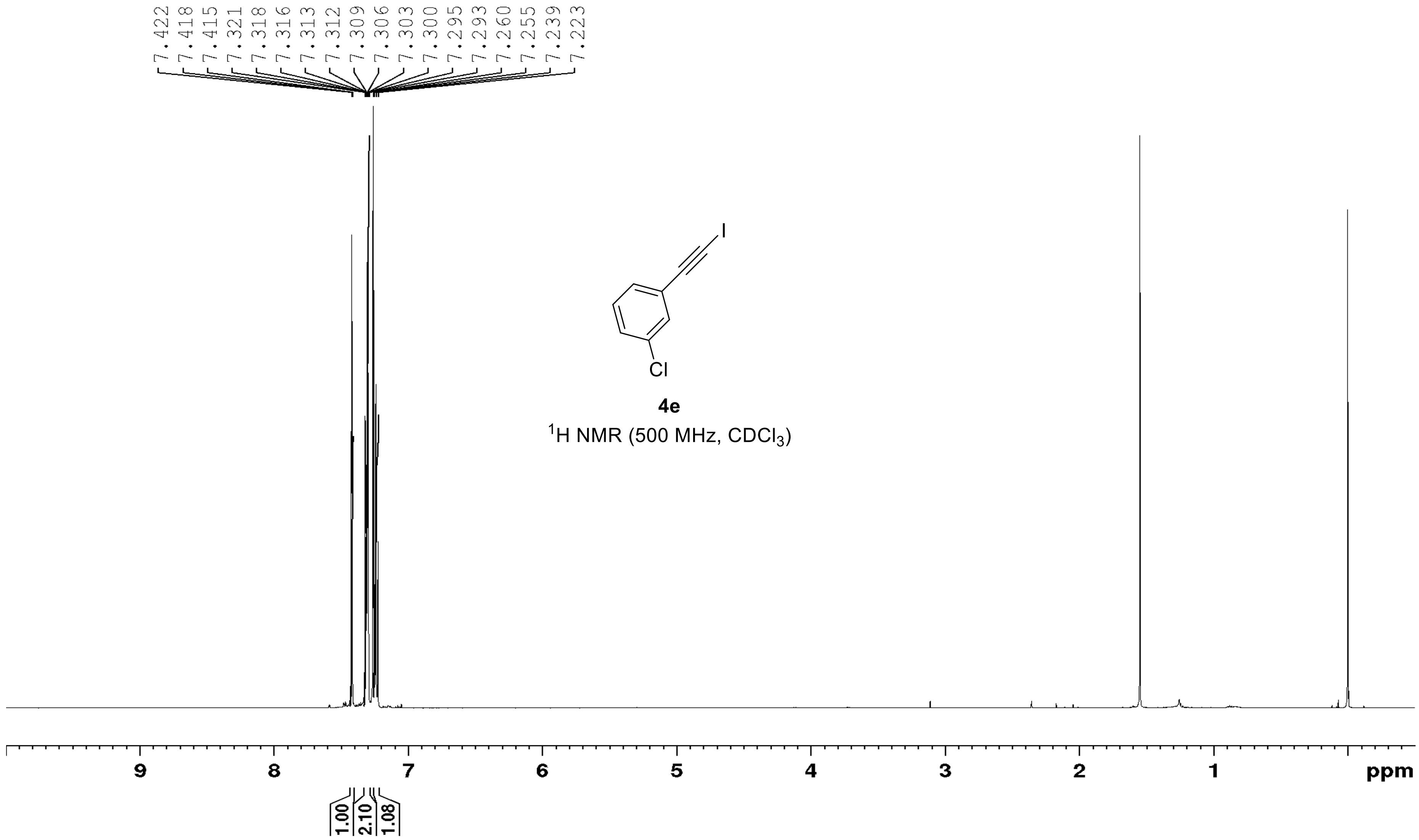
7.422
7.418
7.415
7.321
7.318
7.316
7.313
7.312
7.309
7.306
7.303
7.300
7.295
7.293
7.260
7.255
7.239
7.223
I
Cl
4e
¹H NMR (500 MHz, CDCl3)
9
8
7
6
5
4
3
2
1
ppm
1.00
2.10
1.08

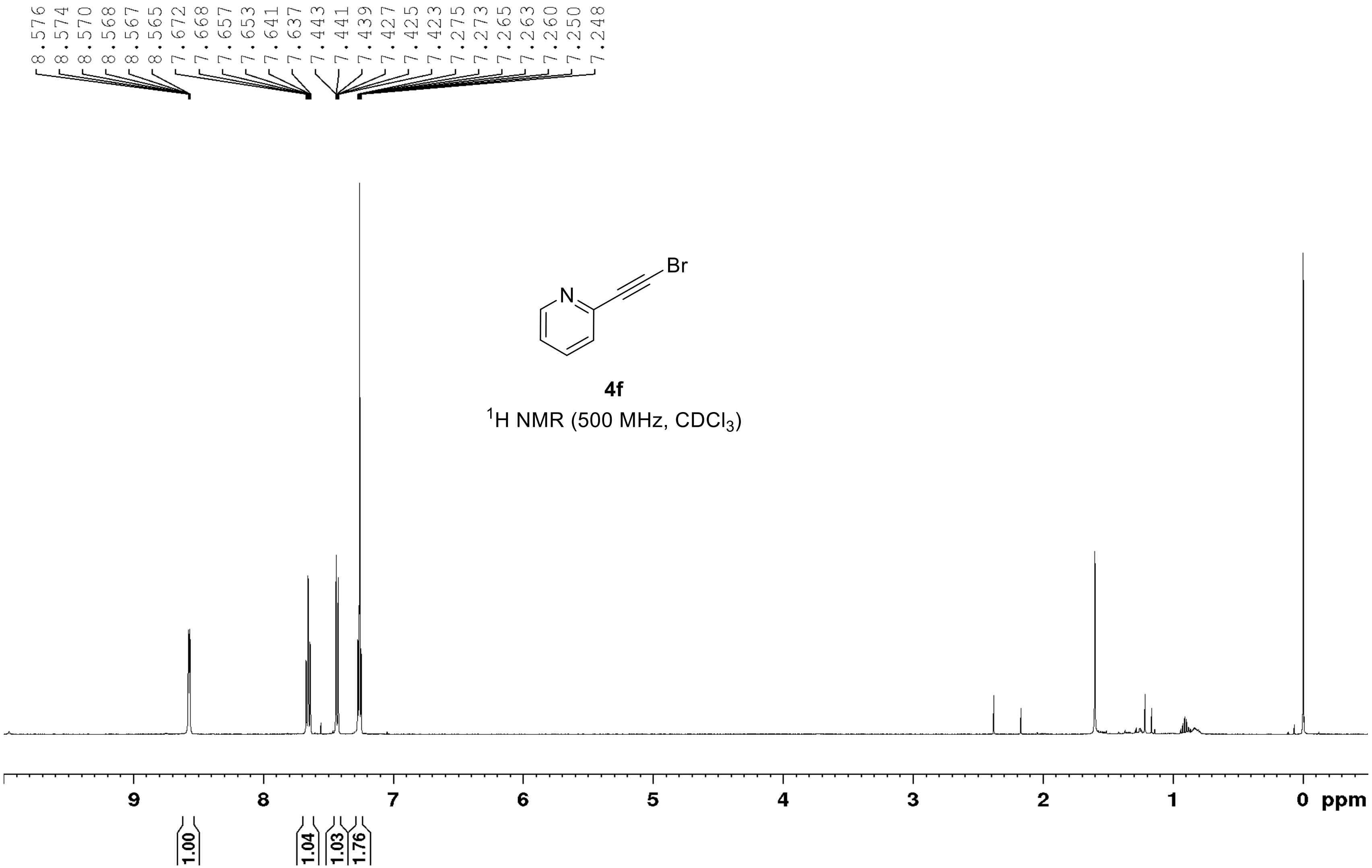
8.576
8.574
8.570
8.568
8.567
8.565
7.672
7.668
7.657
7.653
7.641
7.637
7.443
7.441
7.439
7.427
7.425
7.423
7.275
7.273
7.265
7.263
7.260
7.250
7.248
N
Br
4f
1H NMR (500 MHz, CDCl3)
9
8
7
6
5
4
3
2
1
0 ppm
1.00
1.04
1.03
1.76

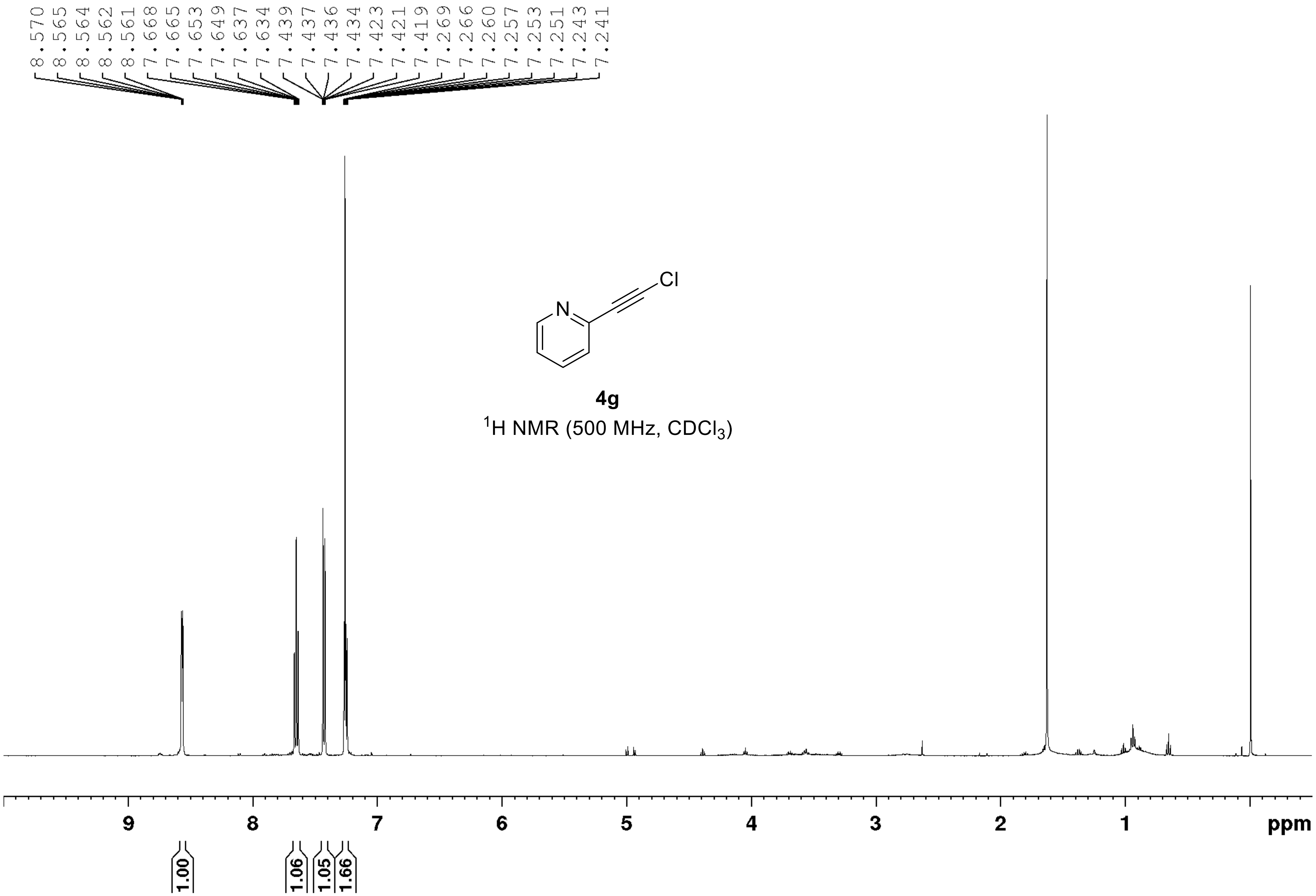
8.570
8.565
8.564
8.562
8.561
7.668
7.665
7.653
7.649
7.637
7.634
7.439
7.437
7.436
7.434
7.423
7.421
7.419
7.269
7.266
7.260
7.257
7.253
7.251
7.243
7.241
N
Cl
4g
1H NMR (500 MHz, CDCl3)
9
8
7
6
5
4
3
2
1
ppm
1.00
1.06
1.05
1.66

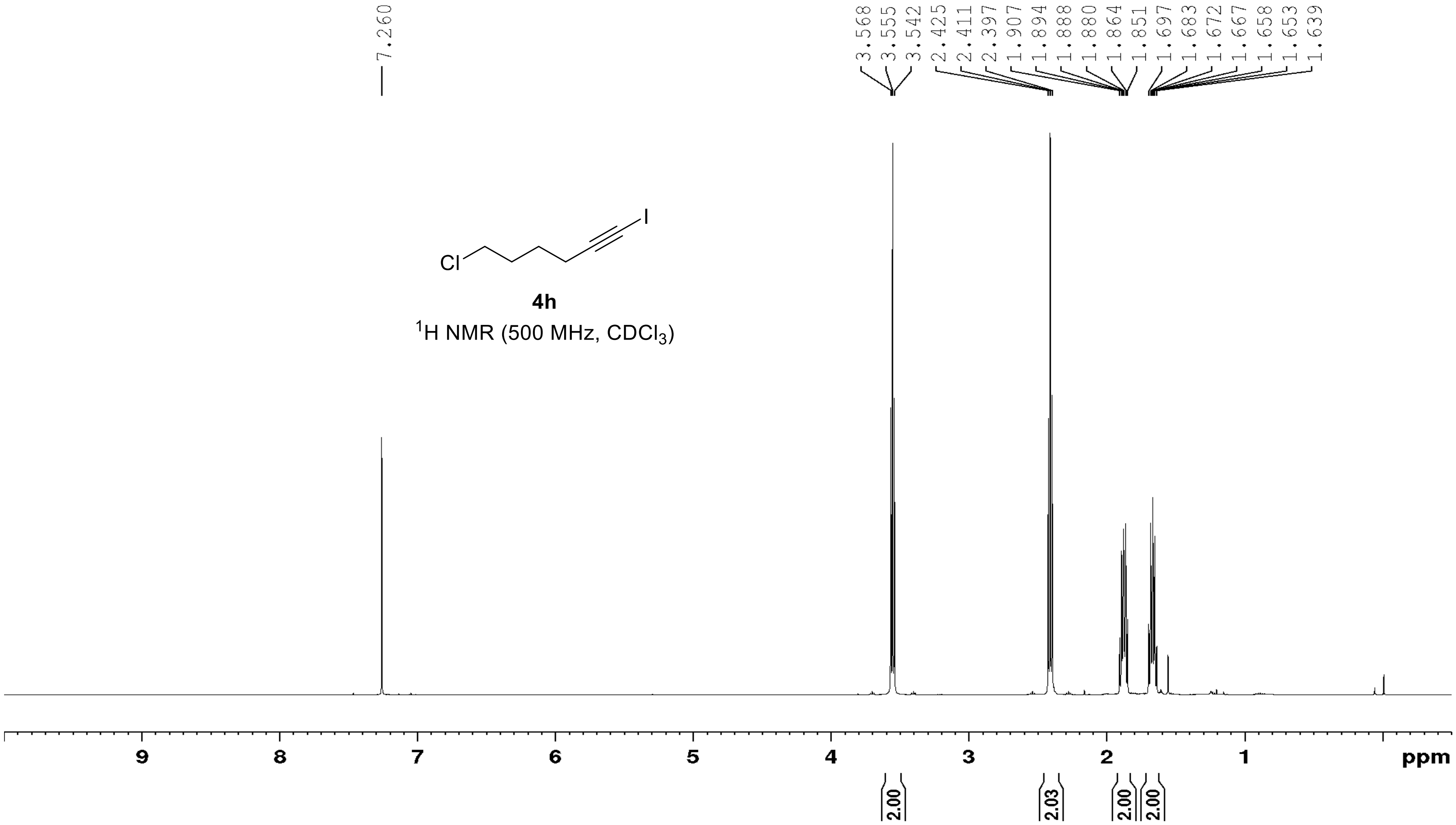
7.260
3.568
3.555
3.542
2.425
2.411
2.397
1.907
1.894
1.888
1.880
1.864
1.851
1.697
1.683
1.672
1.667
1.658
1.653
1.639
Cl
I
4h
^{1}H NMR (500 MHz, $CDCl_3$)
9
8
7
6
5
4
3
2
1
ppm
2.00
2.03
2.00
2.00

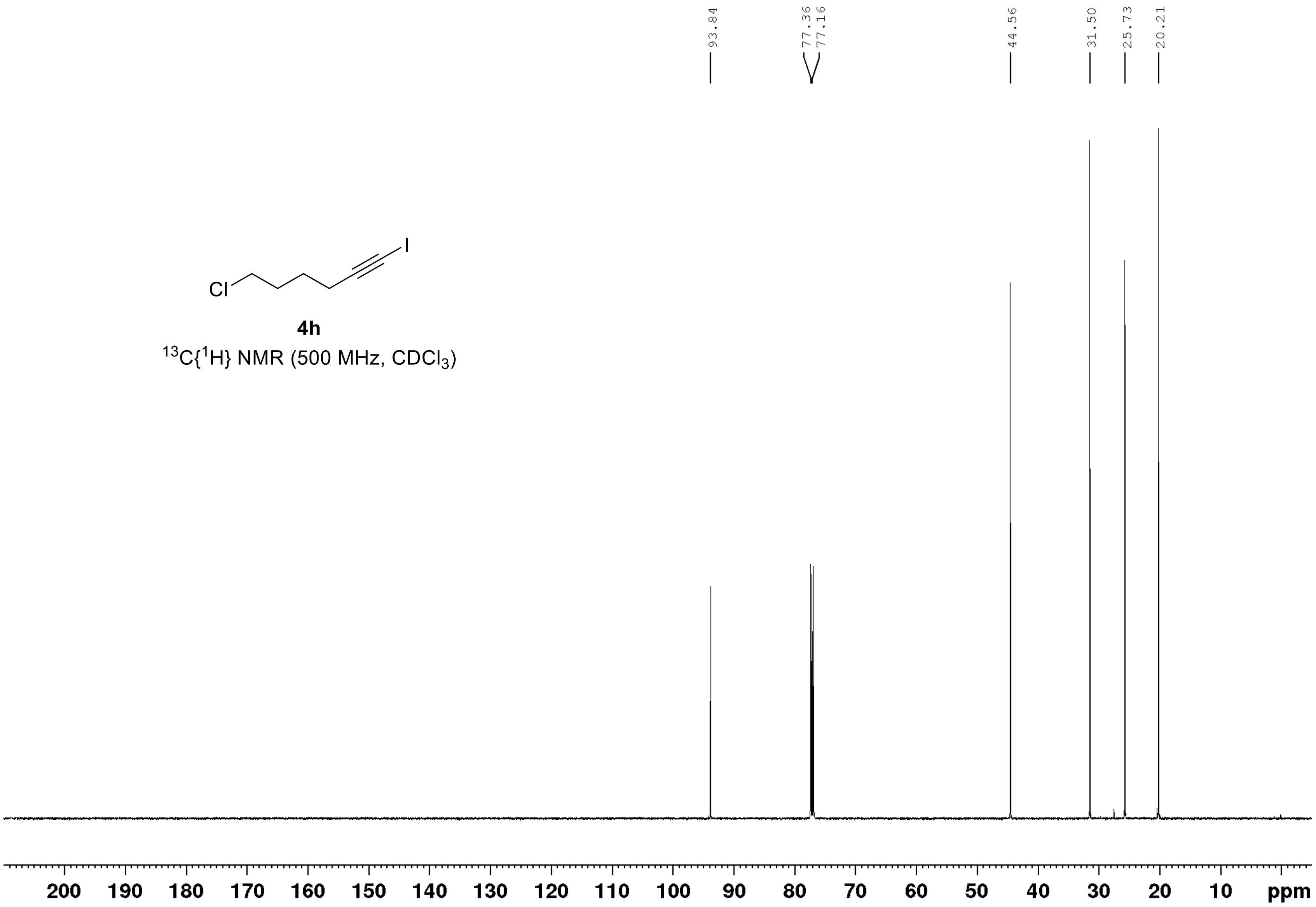

4h

$^{13}C\{^{1}H\}$ NMR (500 MHz, $CDCl_3$)

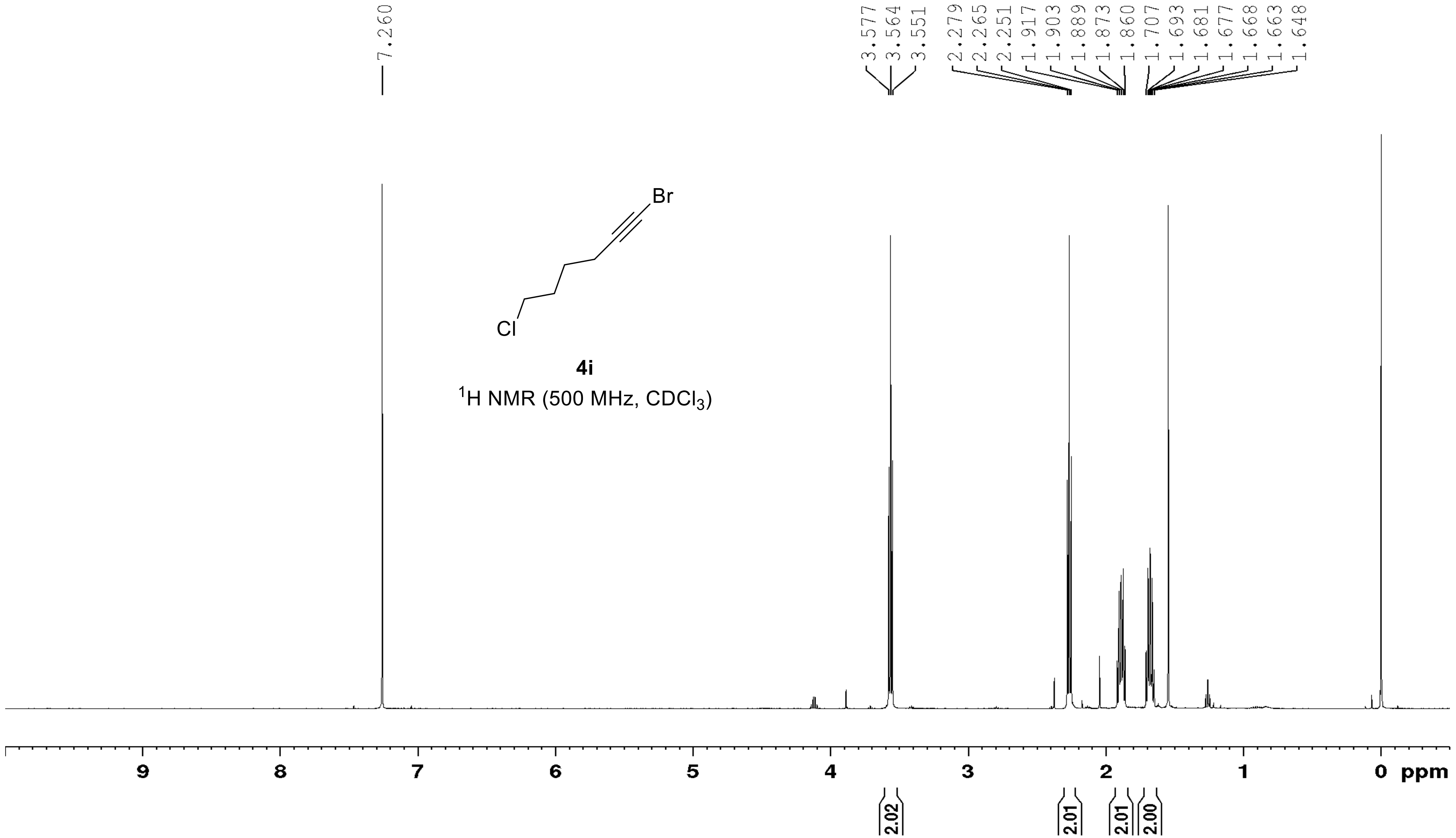
7.260
3.577
3.564
3.551
2.279
2.265
2.251
1.917
1.903
1.889
1.873
1.860
1.707
1.693
1.681
1.677
1.668
1.663
1.648
Br
Cl
4i
^{1}H NMR (500 MHz, $CDCl_3$)
9
8
7
6
5
4
3
2
1
0 ppm
2.02
2.01
2.01
2.00

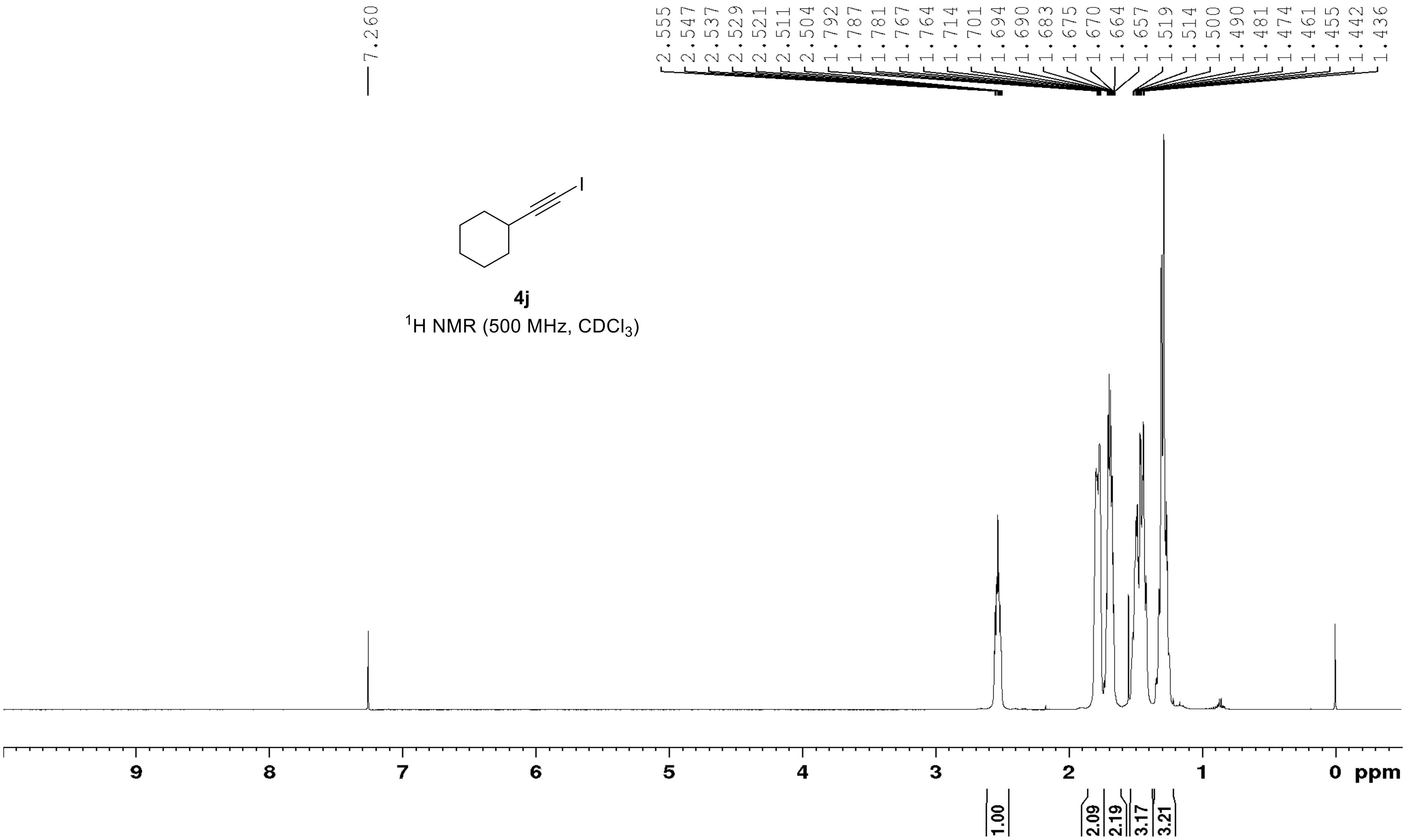

4j

^{1}H NMR (500 MHz, $CDCl_3$)

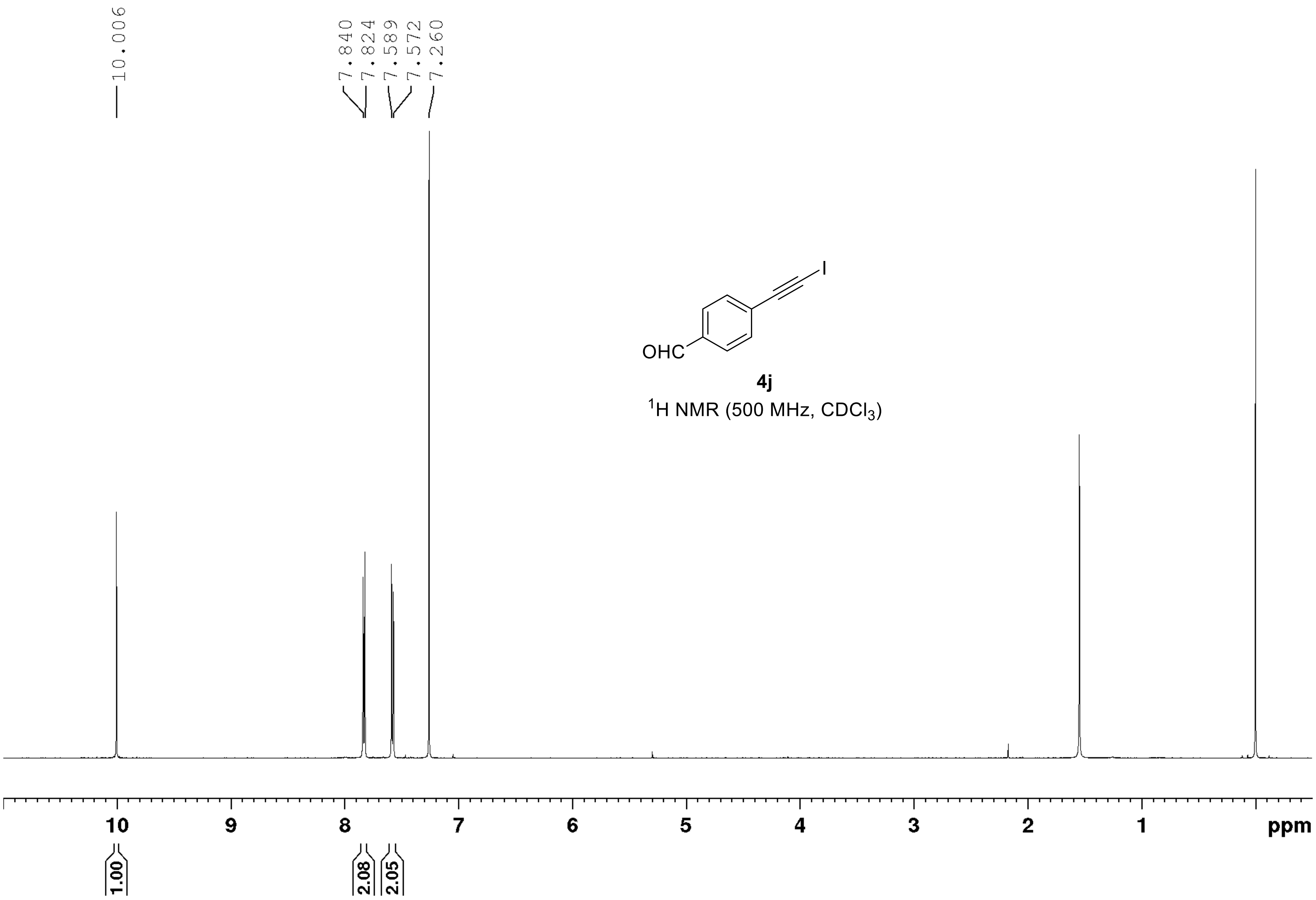
10.006
7.840
7.824
7.589
7.572
7.260
OHC
I
4j
^{1}H NMR (500 MHz, $CDCl_3$)
10
9
8
7
6
5
4
3
2
1
ppm
1.00
2.08
2.05

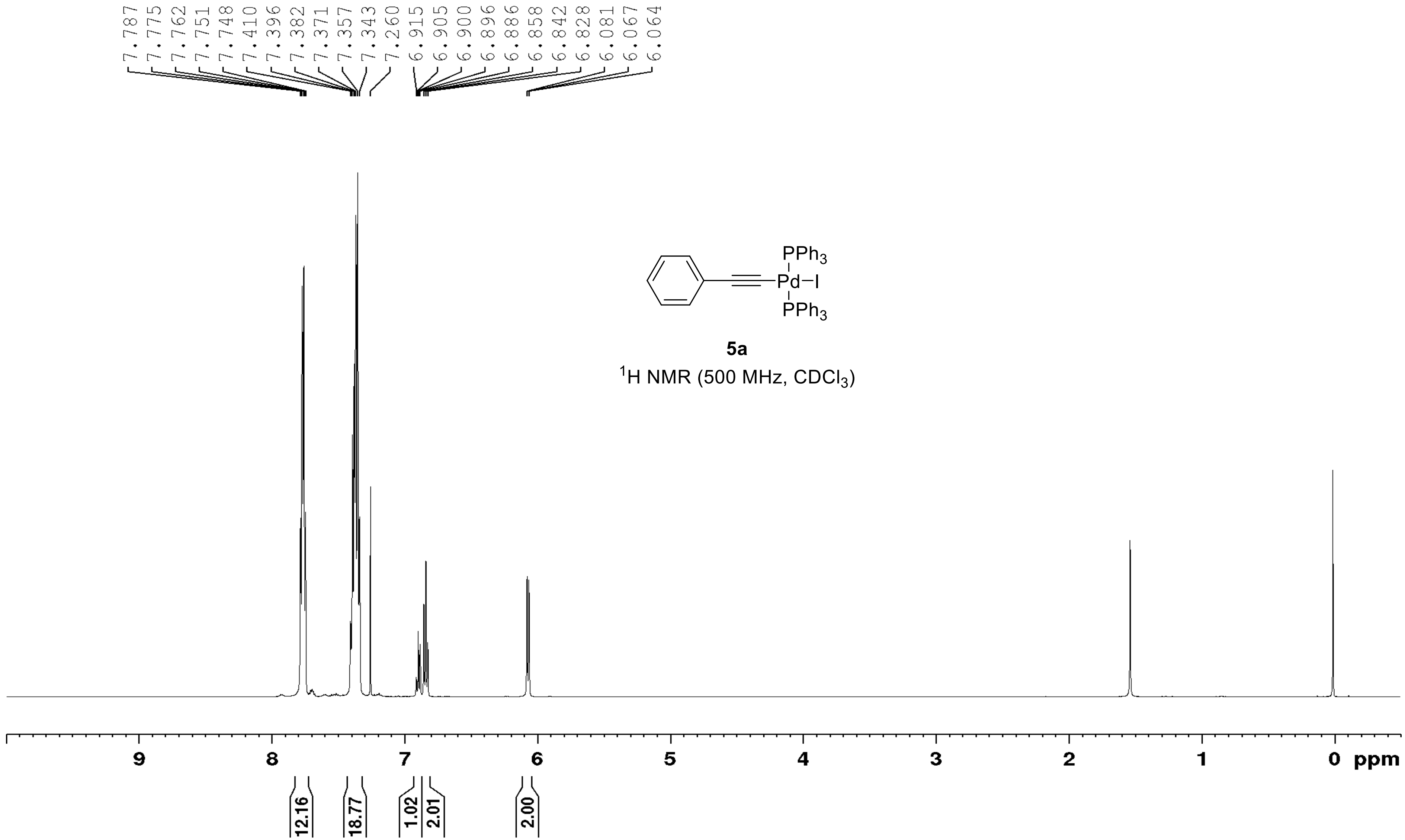
7.787
7.775
7.762
7.751
7.748
7.410
7.396
7.382
7.371
7.357
7.343
7.260
6.915
6.905
6.900
6.896
6.886
6.858
6.842
6.828
6.081
6.067
6.064
PPh3
Pd
I
PPh3
5a
1H NMR (500 MHz, CDCl3)
9
8
7
6
5
4
3
2
1
0
ppm
12.16
18.77
1.02
2.01
2.00

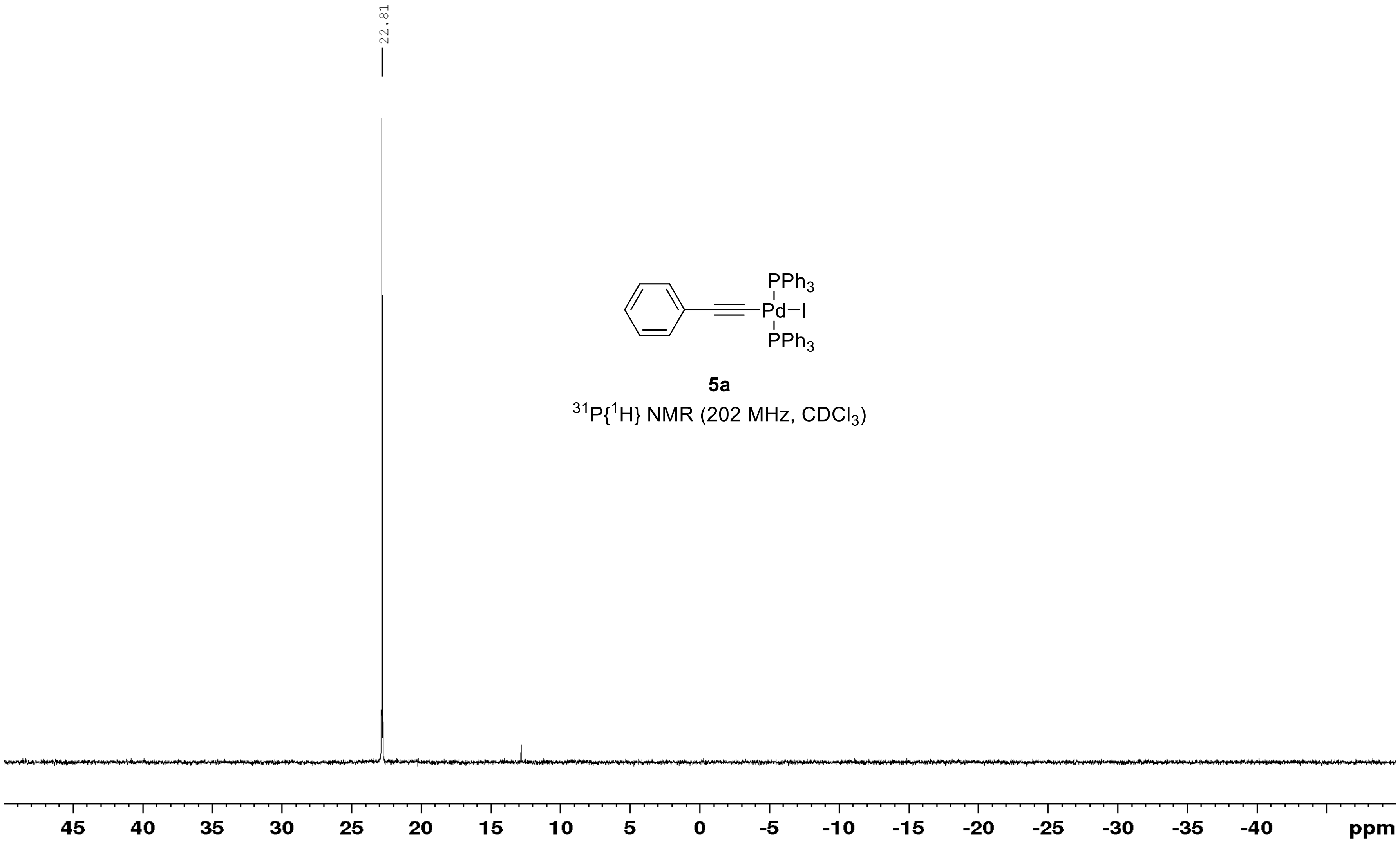
22.81
PPh3
Pd–I
PPh3
5a
31P{1H} NMR (202 MHz, CDCl3)
45
40
35
30
25
20
15
10
5
0
-5
-10
-15
-20
-25
-30
-35
-40
ppm

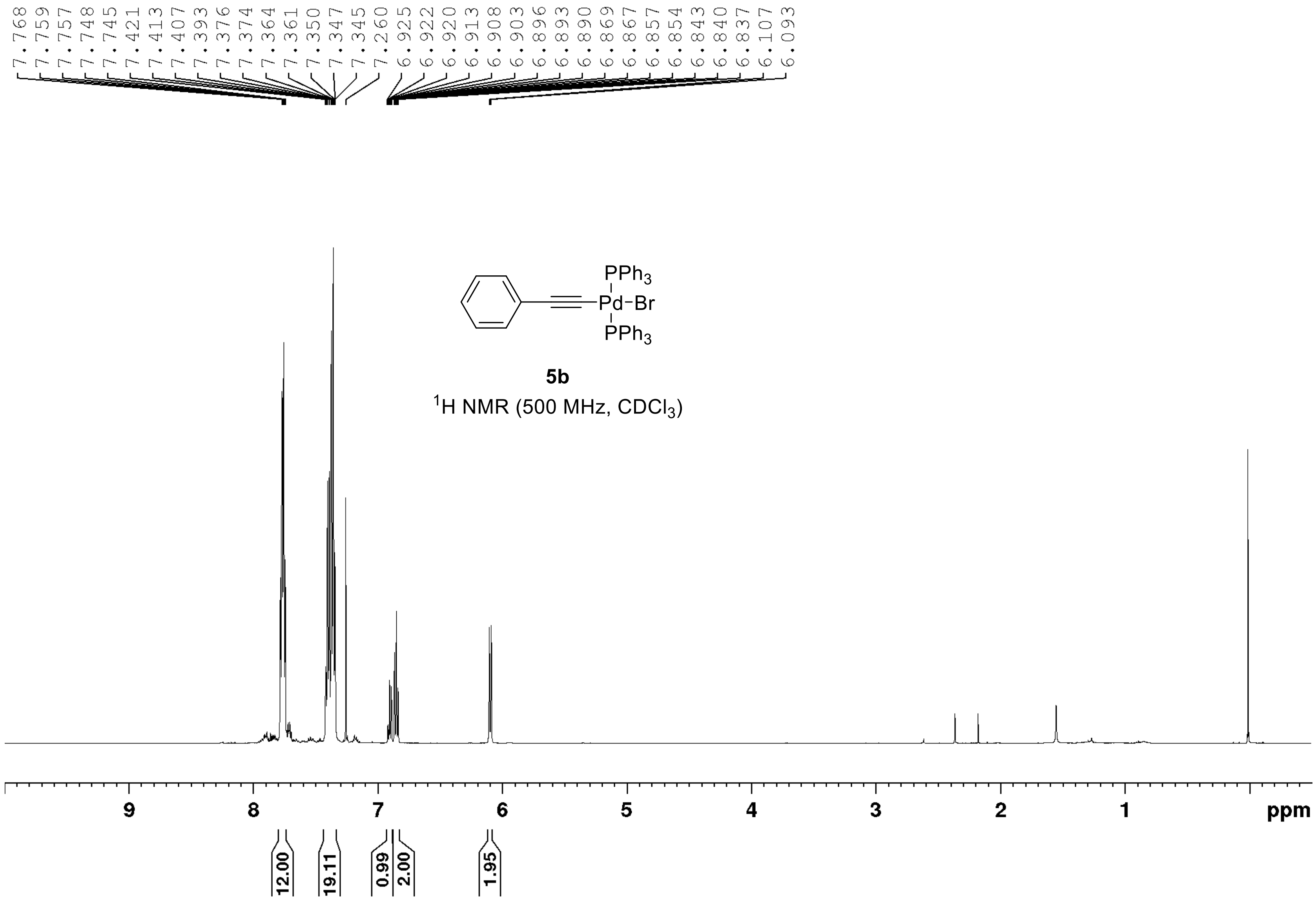
7.768 7.759 7.757 7.748 7.745 7.421 7.413 7.407 7.393 7.376 7.374 7.364 7.361 7.350 7.347 7.345 7.260 6.925 6.922 6.920 6.913 6.908 6.903 6.896 6.893 6.890 6.869 6.867 6.857 6.854 6.843 6.840 6.837 6.107 6.093
PPh3
Pd
Br
PPh3
5b
1H NMR (500 MHz, CDCl3)
9 8 7 6 5 4 3 2 1 ppm
12.00 19.11 0.99 2.00 1.95

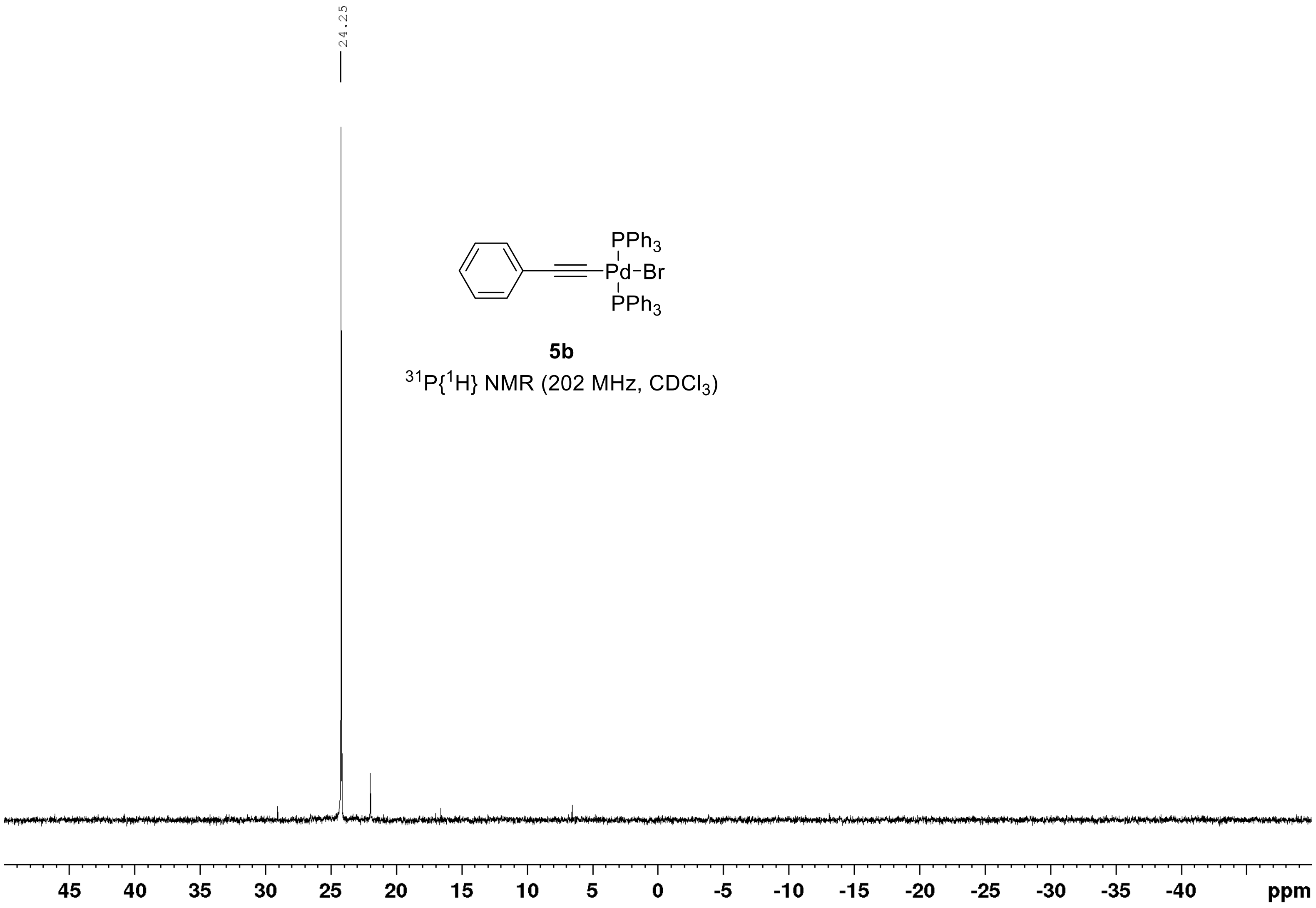
24.25
PPh3
Pd
Br
PPh3
5b
31P{1H} NMR (202 MHz, CDCl3)
45 40 35 30 25 20 15 10 5 0 -5 -10 -15 -20 -25 -30 -35 -40 ppm

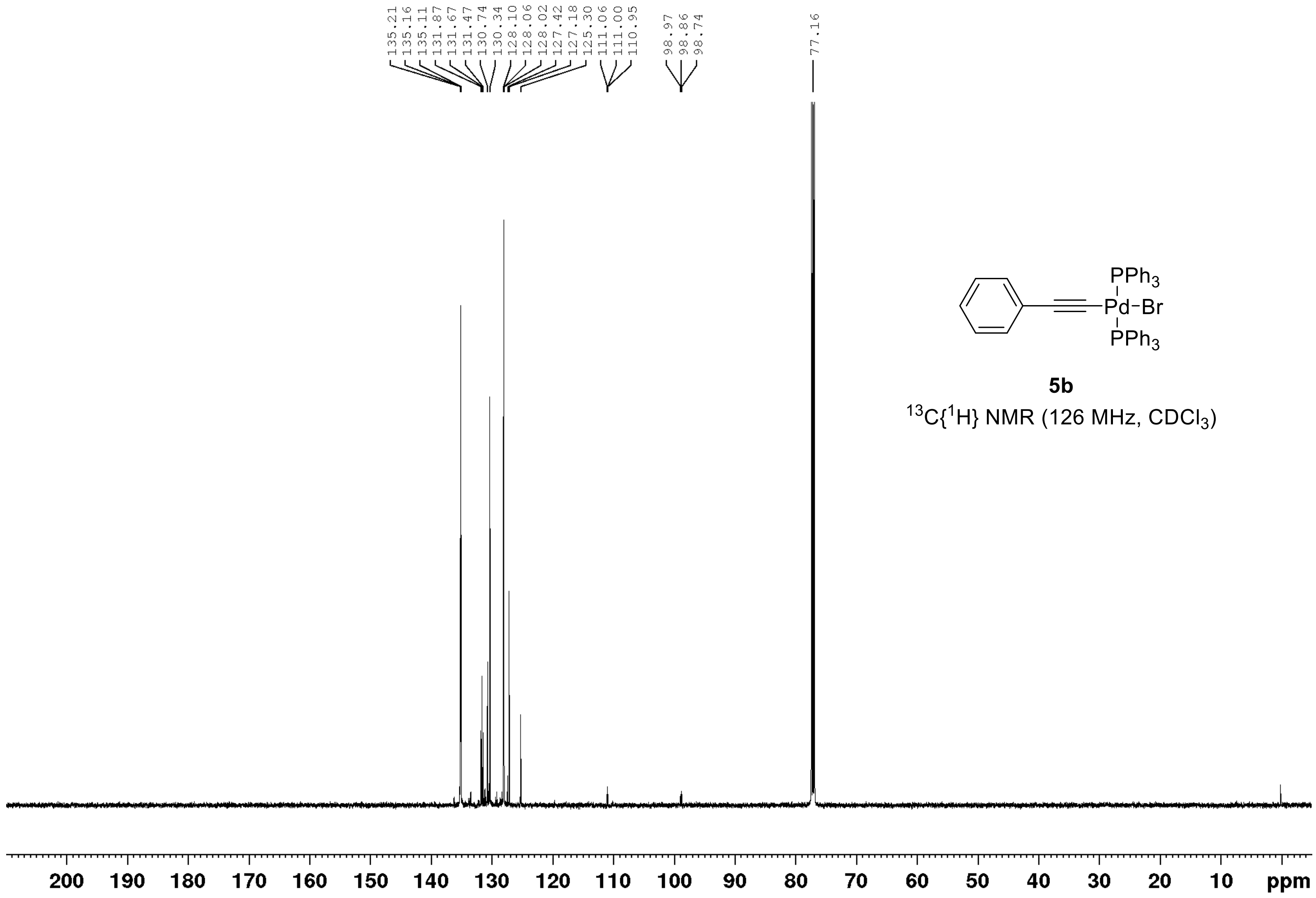
135.21
135.16
135.11
131.87
131.67
131.47
130.74
130.34
128.10
128.06
128.02
127.42
127.18
125.30
111.06
111.00
110.95
98.97
98.86
98.74
77.16
PPh3
Pd–Br
PPh3
5b
13C{1H} NMR (126 MHz, CDCl3)
200 190 180 170 160 150 140 130 120 110 100 90 80 70 60 50 40 30 20 10 ppm

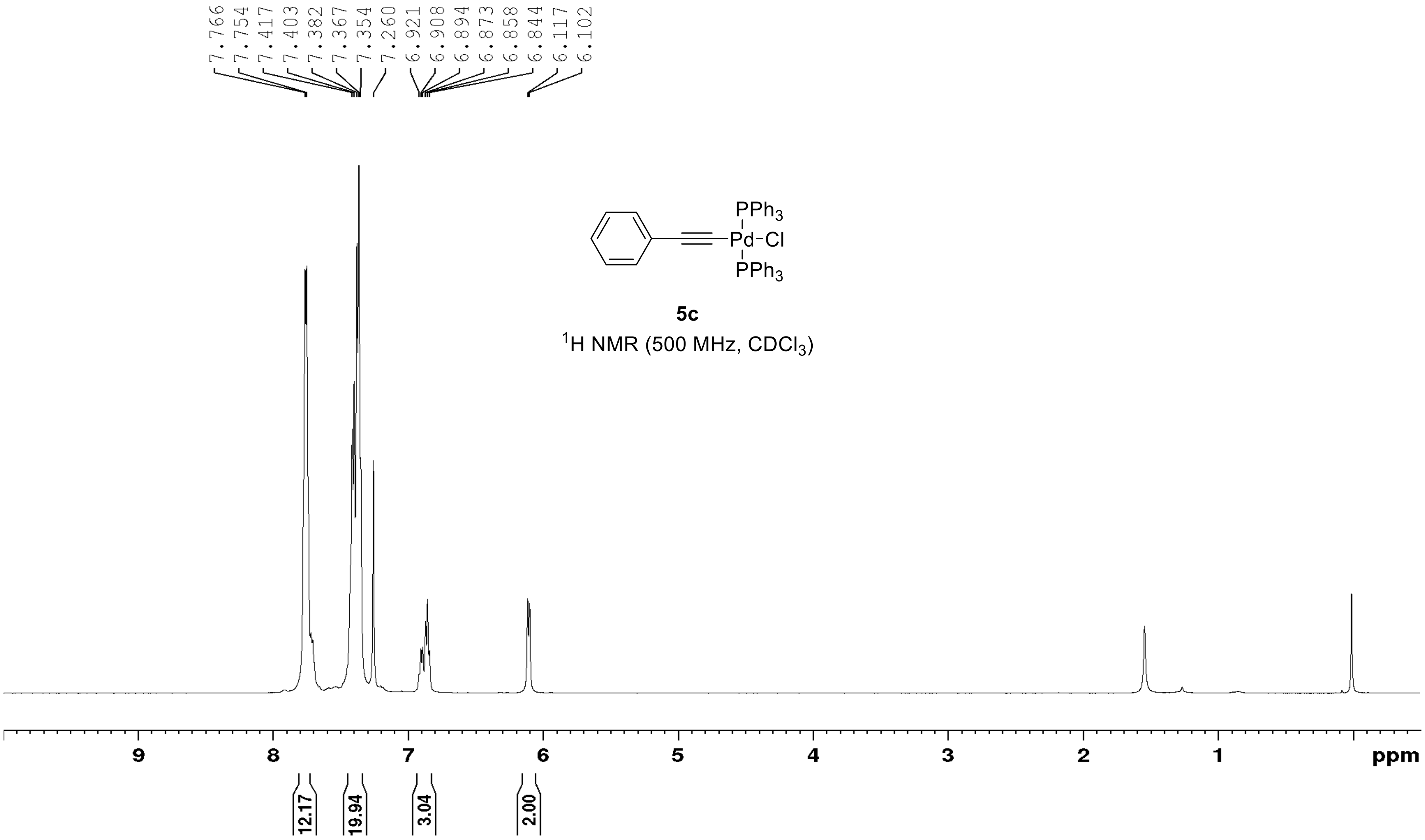

7.766
7.754
7.417
7.403
7.382
7.367
7.354
7.260
6.921
6.908
6.894
6.873
6.858
6.844
6.117
6.102
PPh3
Pd-Cl
PPh3
5c
1H NMR (500 MHz, CDCl3)
9
8
7
6
5
4
3
2
1
ppm
12.17
19.94
3.04
2.00

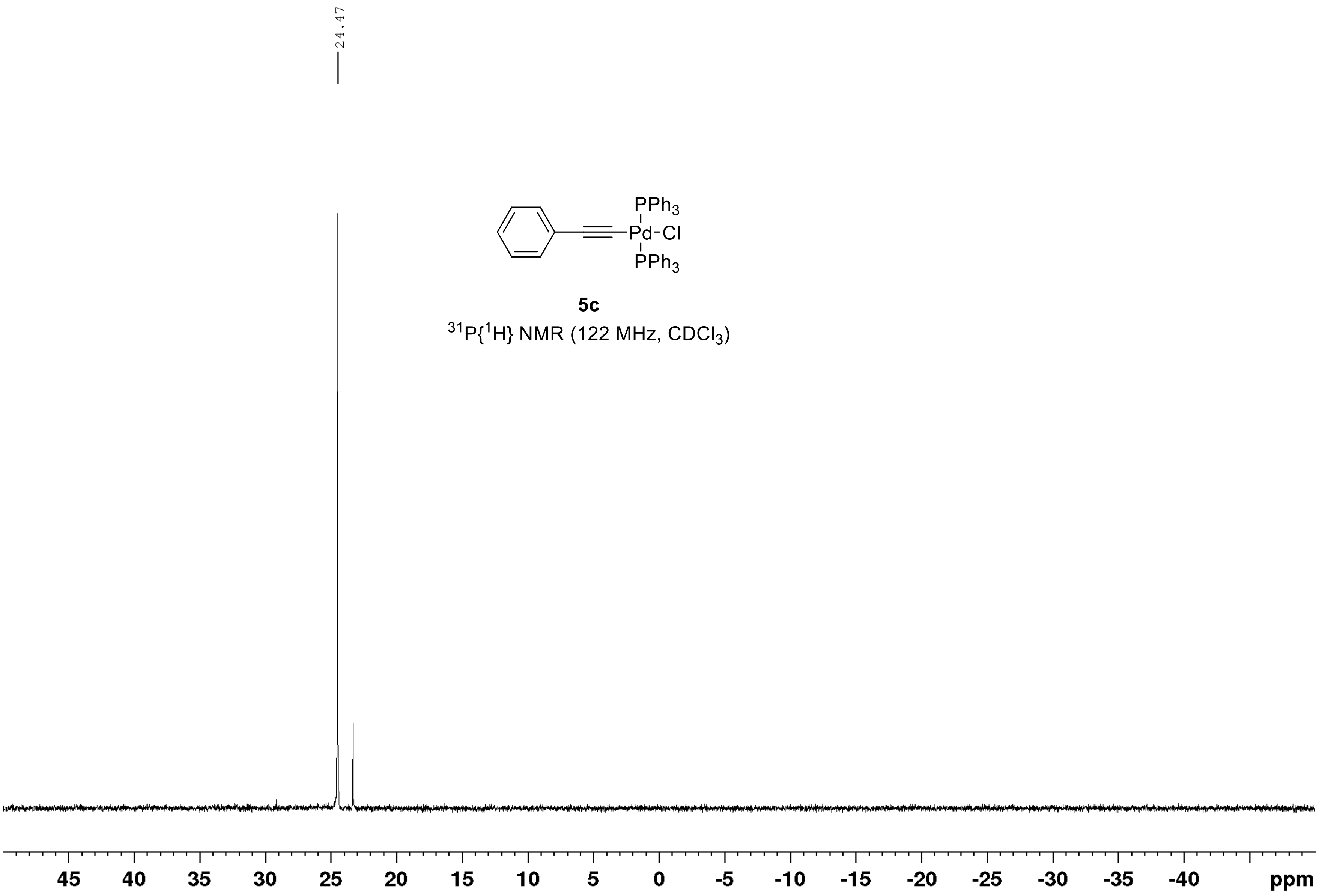

24.47
PPh3
Pd-Cl
PPh3
5c
31P{1H} NMR (122 MHz, CDCl3)
45 40 35 30 25 20 15 10 5 0 -5 -10 -15 -20 -25 -30 -35 -40 ppm

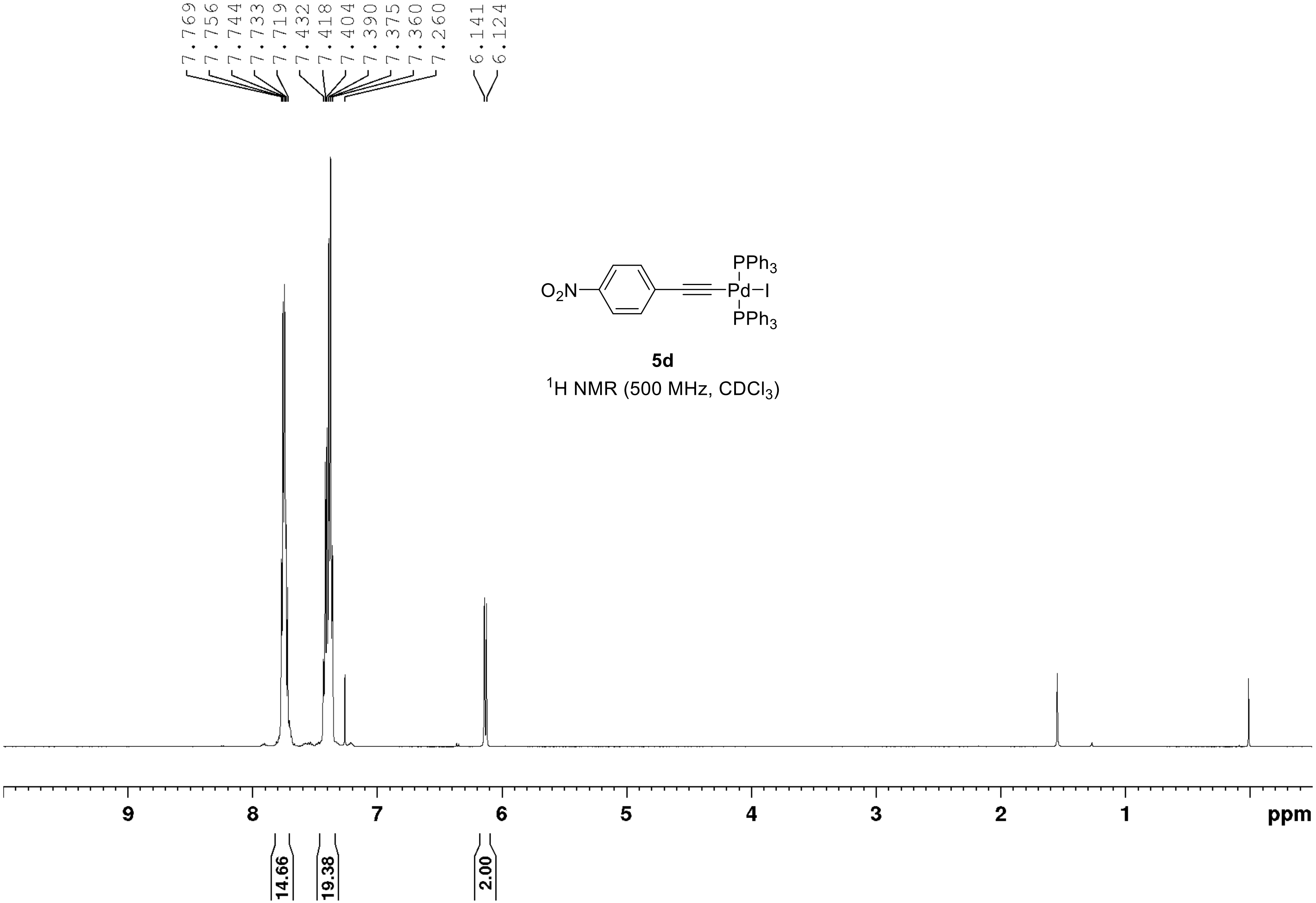

5d

^1H NMR (500 MHz, $CDCl_3$)

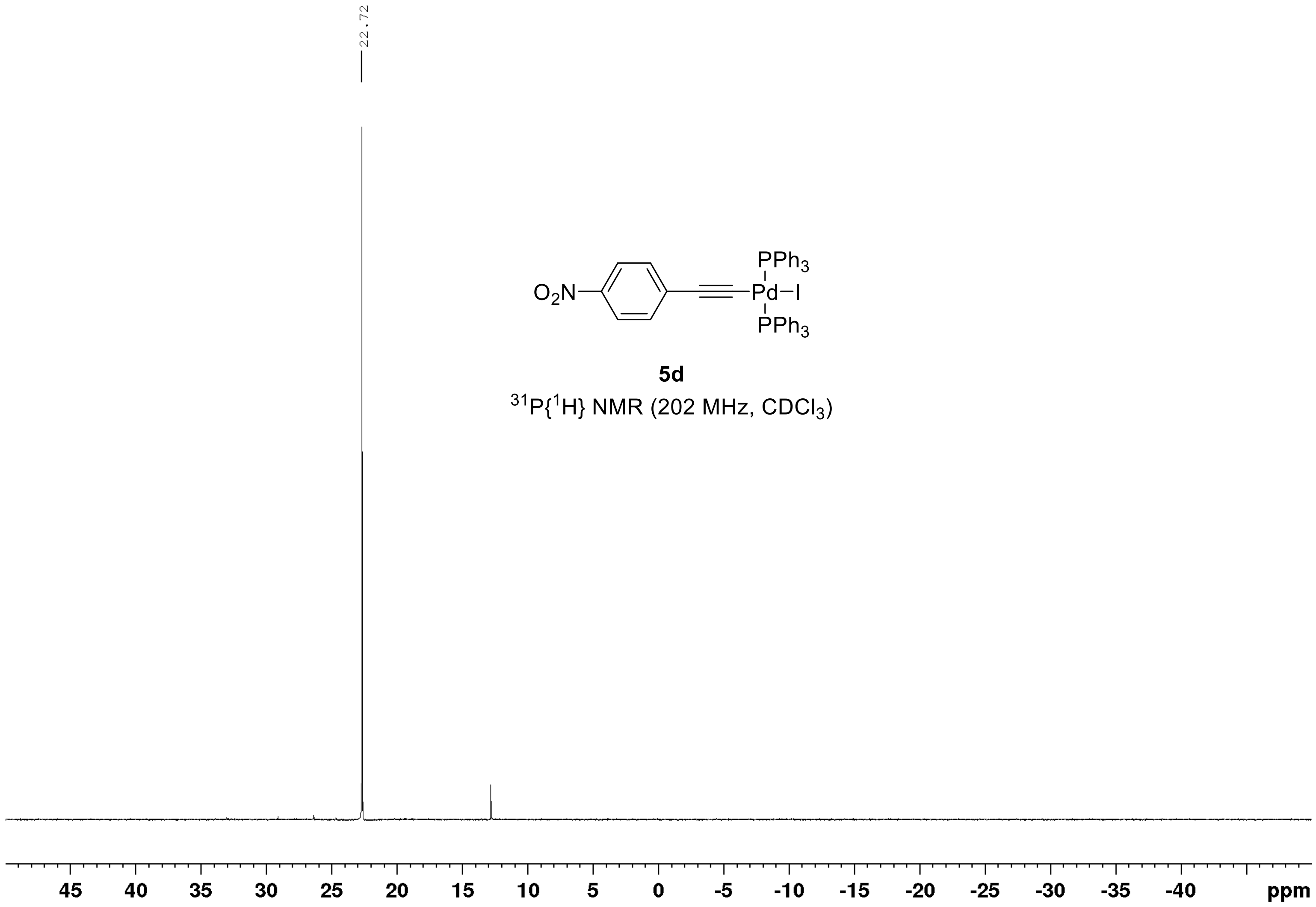

5d

$^{31}P\{^{1}H\}$ NMR (202 MHz, $CDCl_3$)

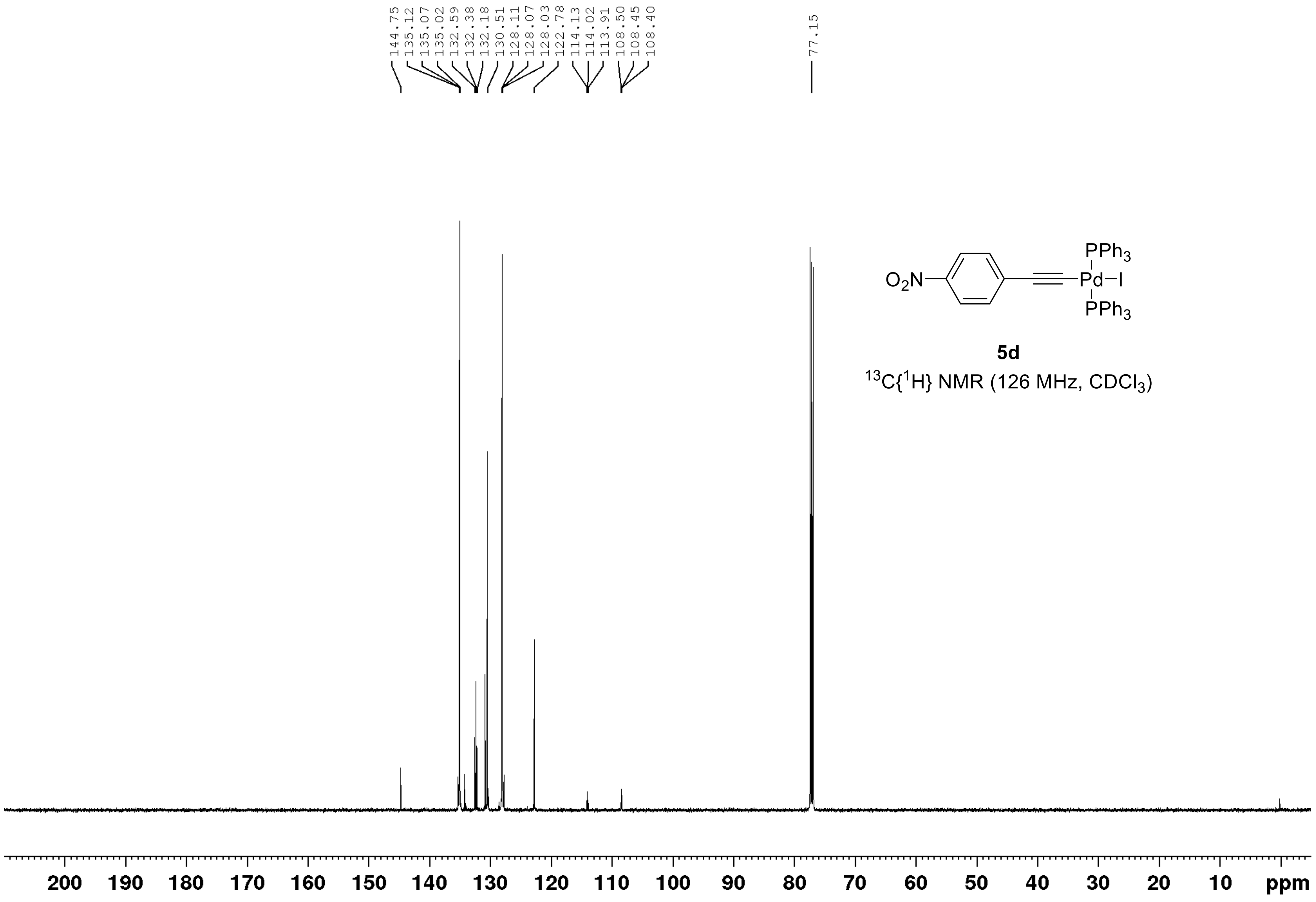

5d

$^{13}C\{^{1}H\}$ NMR (126 MHz, $CDCl_3$)

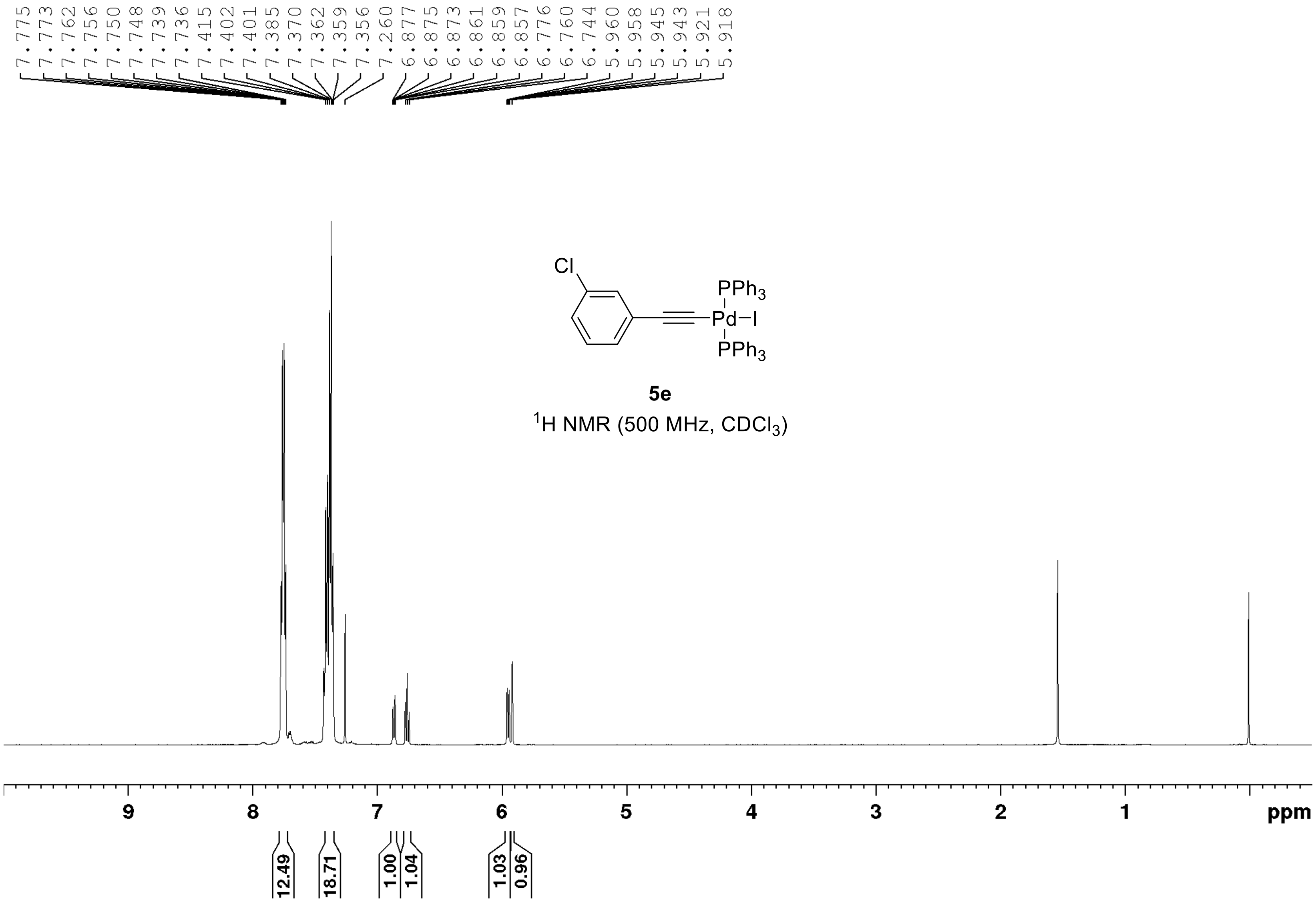
7.775
7.773
7.762
7.756
7.750
7.748
7.739
7.736
7.415
7.402
7.401
7.385
7.370
7.362
7.359
7.356
7.260
6.877
6.875
6.873
6.861
6.859
6.857
6.776
6.760
6.744
5.960
5.958
5.945
5.943
5.921
5.918
Cl
PPh3
Pd–I
PPh3
5e
1H NMR (500 MHz, CDCl3)
9
8
7
6
5
4
3
2
1
ppm
12.49
18.71
1.00
1.04
1.03
0.96

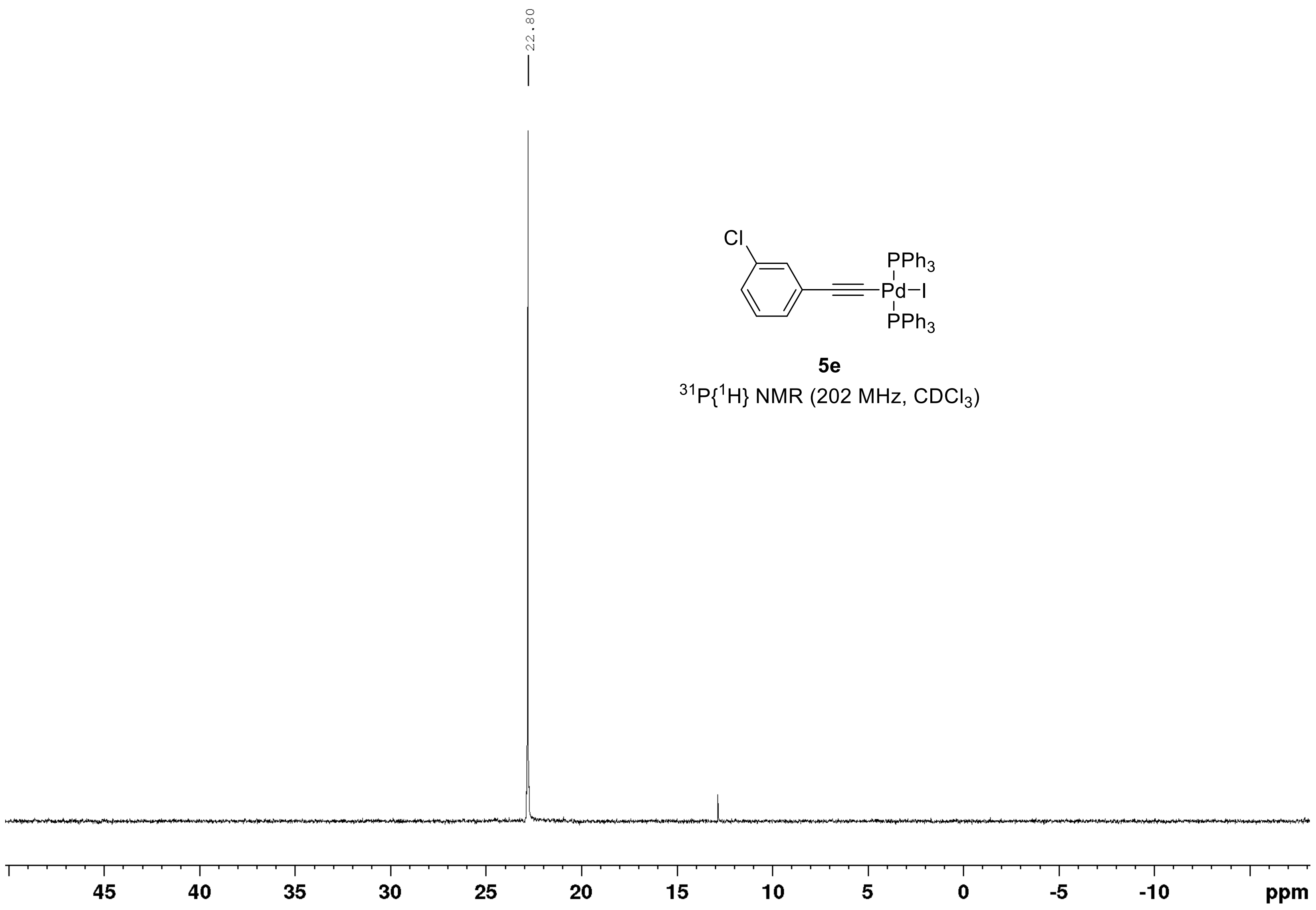
22.80
Cl
PPh3
Pd–I
PPh3
5e
31P{1H} NMR (202 MHz, CDCl3)
45
40
35
30
25
20
15
10
5
0
-5
-10
ppm

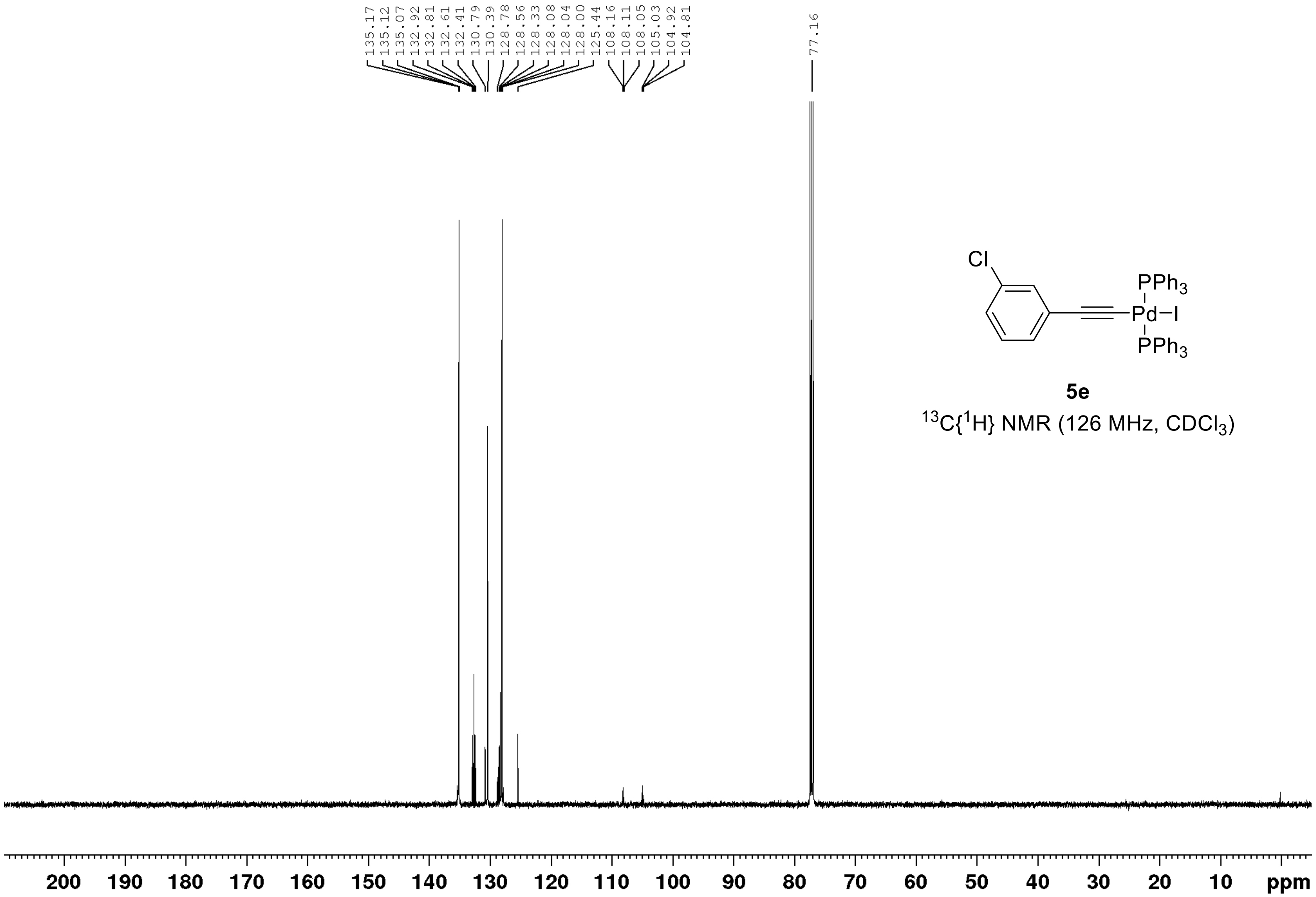

5e

$^{13}C\{^{1}H\}$ NMR (126 MHz, $CDCl_3$)

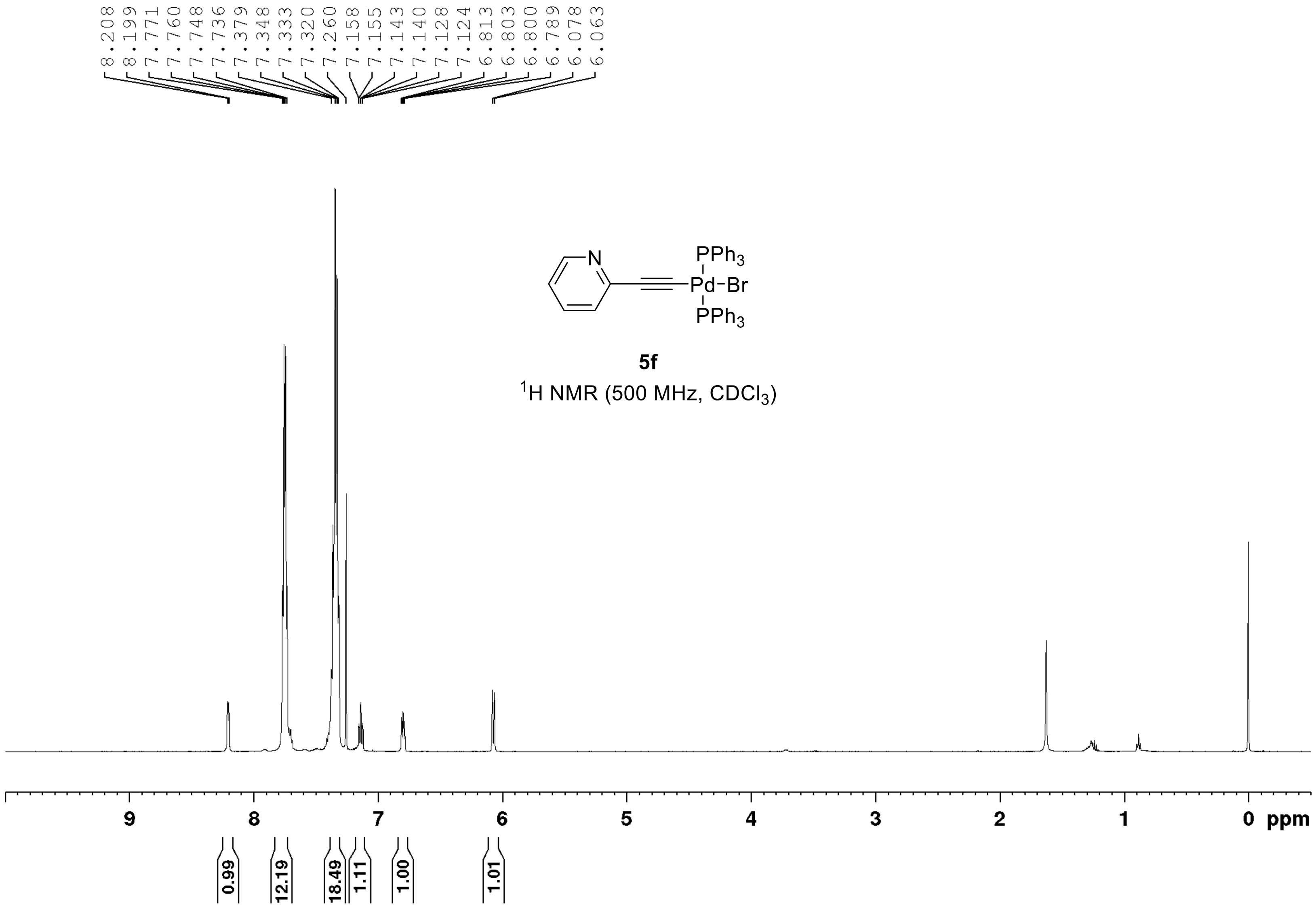
8.208
8.199
7.771
7.760
7.748
7.736
7.379
7.348
7.333
7.320
7.260
7.158
7.155
7.143
7.140
7.128
7.124
6.813
6.803
6.800
6.789
6.078
6.063
N
PPh3
Pd
Br
PPh3
5f
1H NMR (500 MHz, CDCl3)
9
8
7
6
5
4
3
2
1
0 ppm
0.99
12.19
18.49
1.11
1.00
1.01

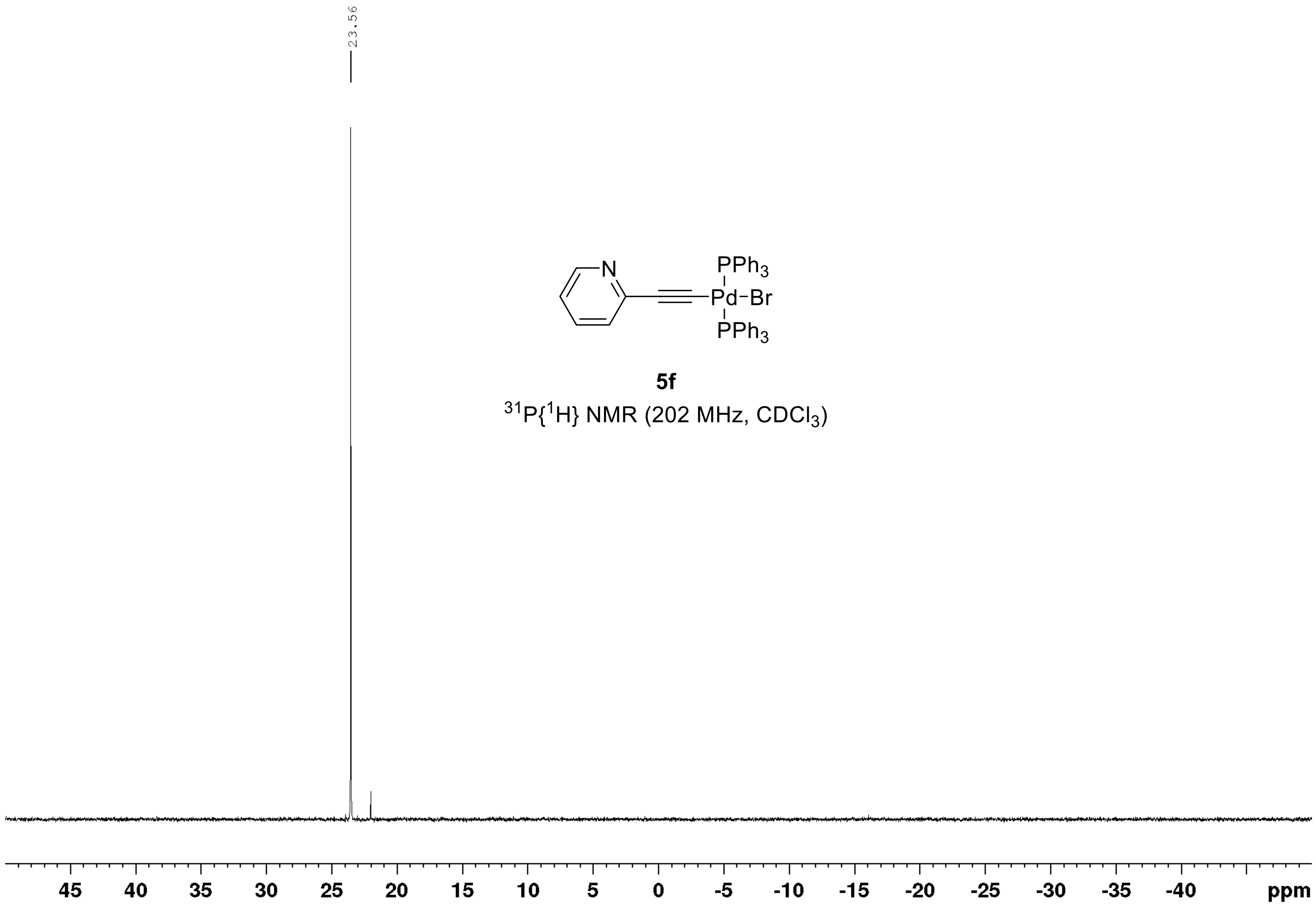

23.56
N
PPh3
Pd-Br
PPh3
5f
31P{1H} NMR (202 MHz, CDCl3)
45 40 35 30 25 20 15 10 5 0 -5 -10 -15 -20 -25 -30 -35 -40 ppm

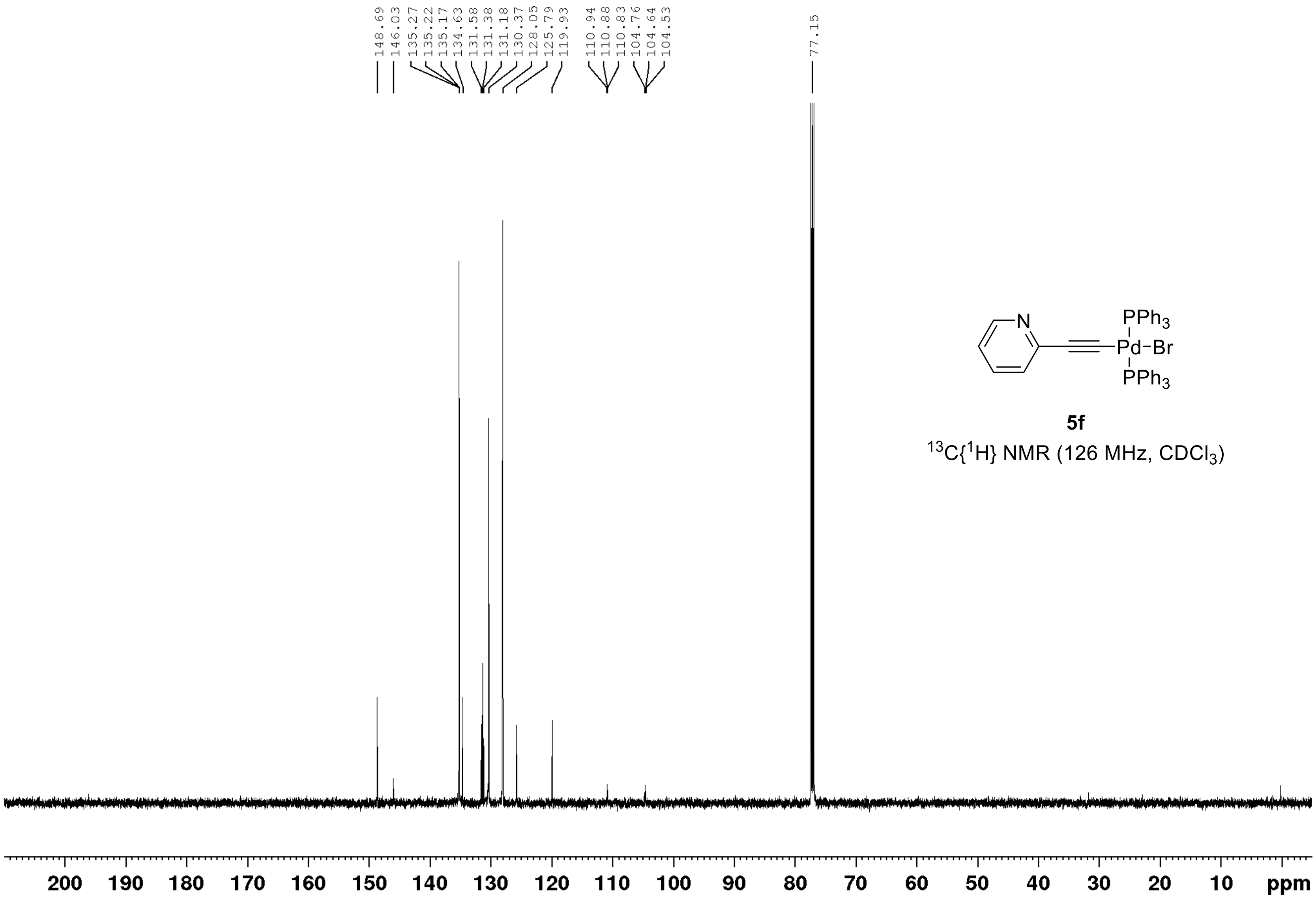

5f

$^{13}C\{^{1}H\}$ NMR (126 MHz, $CDCl_3$)

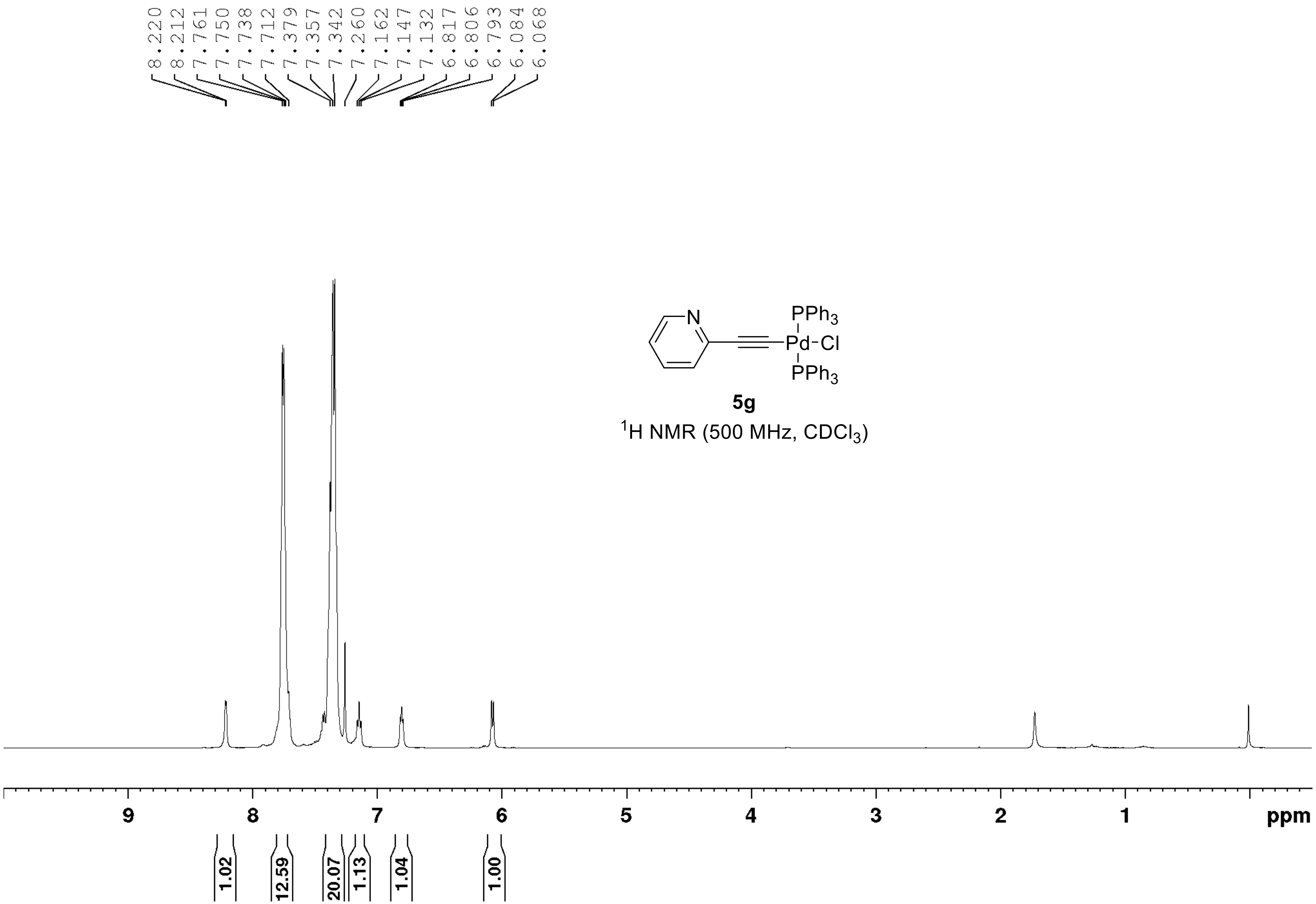

8.220
8.212
7.761
7.750
7.738
7.712
7.379
7.357
7.342
7.260
7.162
7.147
7.132
6.817
6.806
6.793
6.084
6.068
PPh3
N
Pd-Cl
PPh3
5g
1H NMR (500 MHz, CDCl3)
9
8
7
6
5
4
3
2
1
ppm
1.02
12.59
20.07
1.13
1.04
1.00

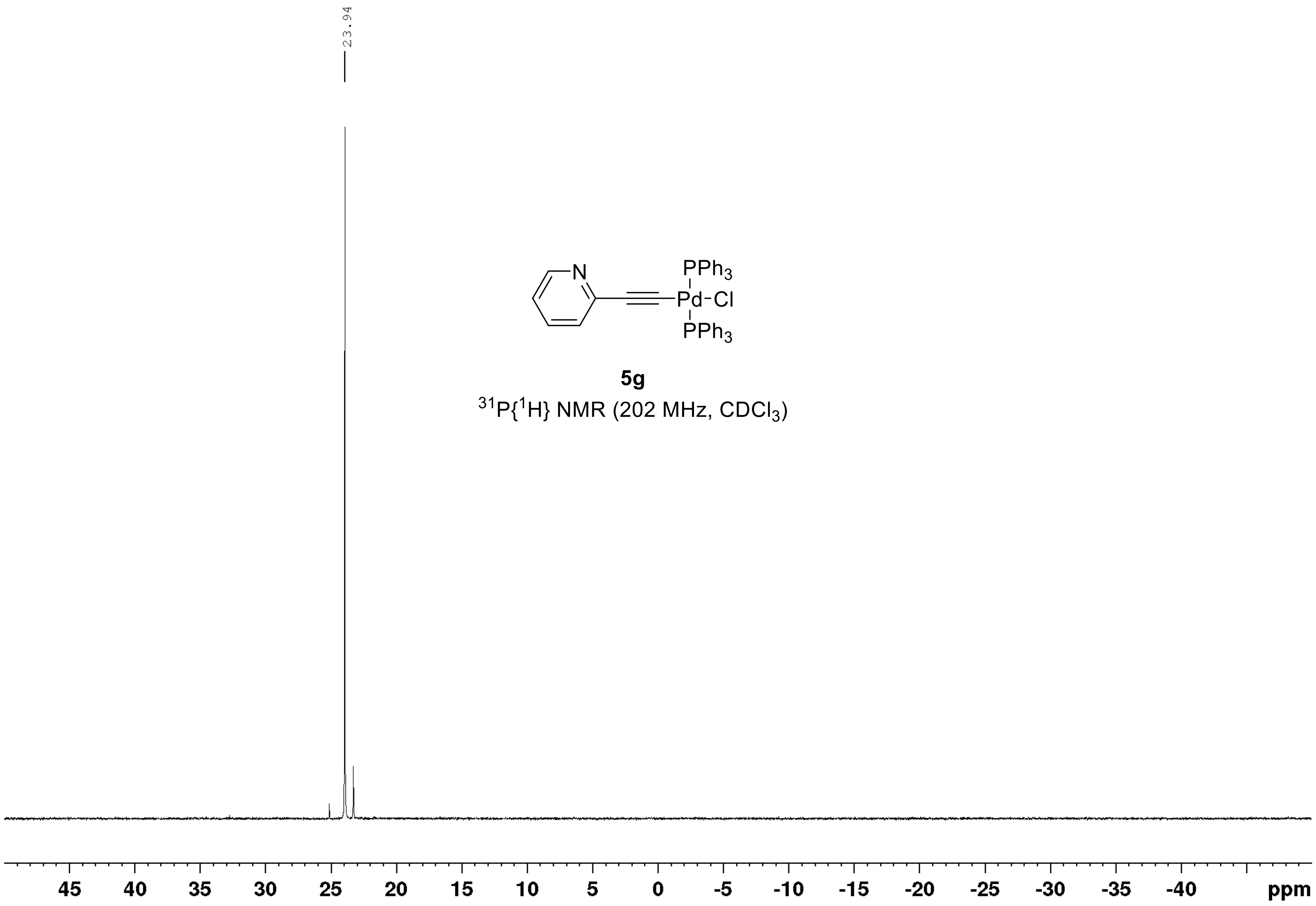

5g

$^{31}P\{^{1}H\}$ NMR (202 MHz, $CDCl_3$)

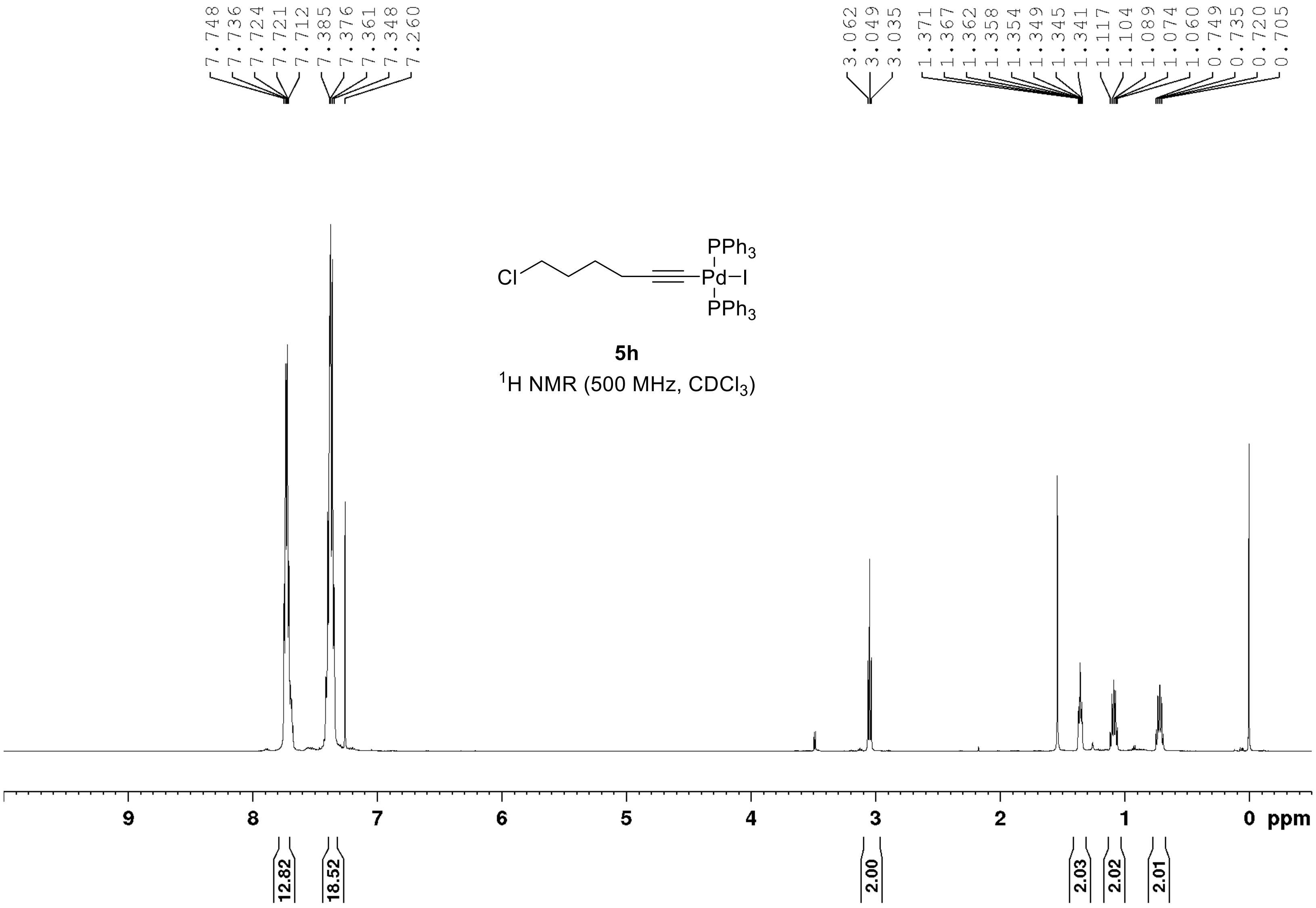
7.748
7.736
7.724
7.721
7.712
7.385
7.376
7.361
7.348
7.260
3.062
3.049
3.035
1.371
1.367
1.362
1.358
1.354
1.349
1.345
1.341
1.117
1.104
1.089
1.074
1.060
0.749
0.735
0.720
0.705
PPh3
Cl
Pd
I
PPh3
5h
1H NMR (500 MHz, CDCl3)
9
8
7
6
5
4
3
2
1
0
ppm
12.82
18.52
2.00
2.03
2.02
2.01

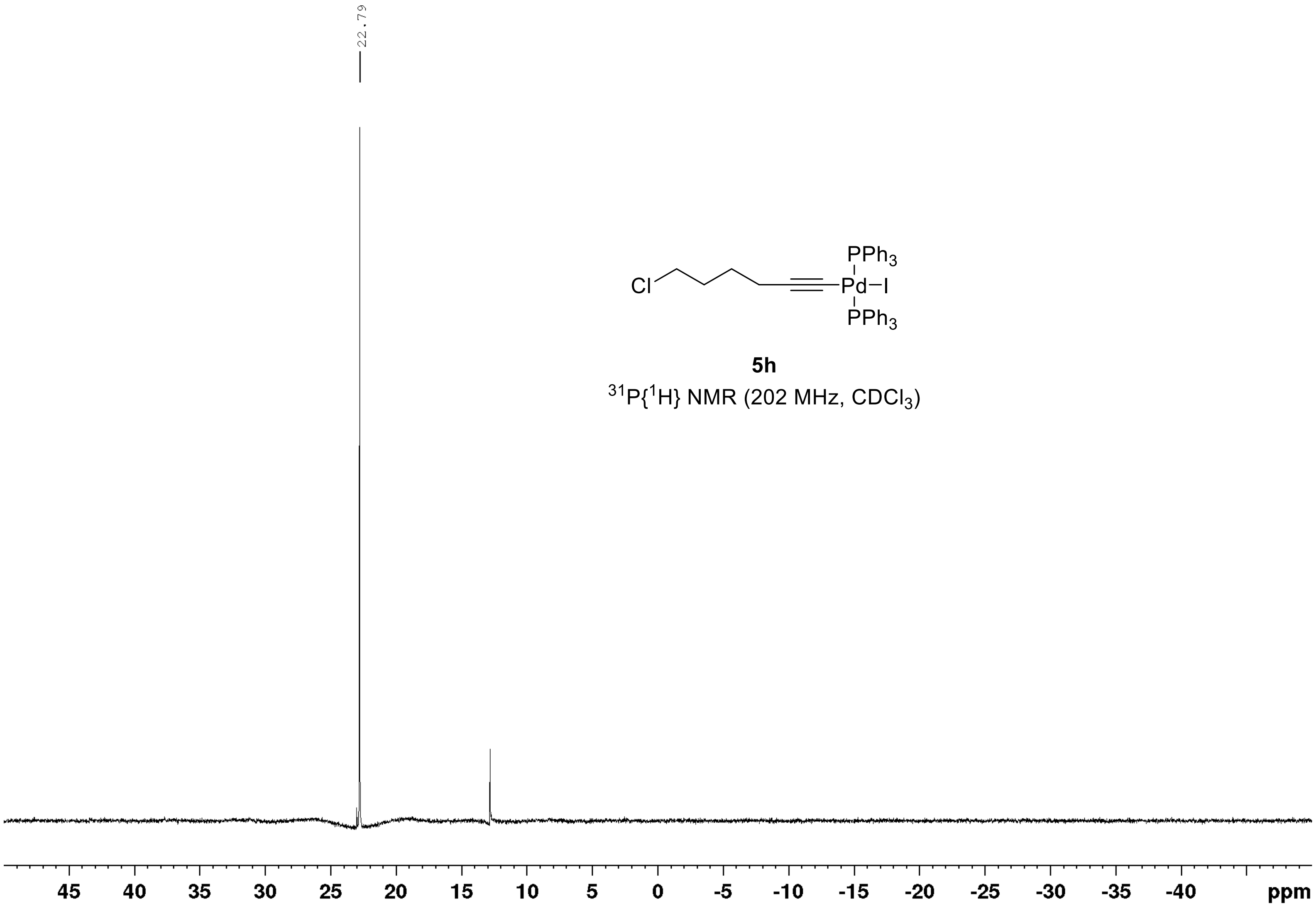
22.79
PPh3
Cl
Pd
I
PPh3
5h
31P{1H} NMR (202 MHz, CDCl3)
45 40 35 30 25 20 15 10 5 0 -5 -10 -15 -20 -25 -30 -35 -40 ppm

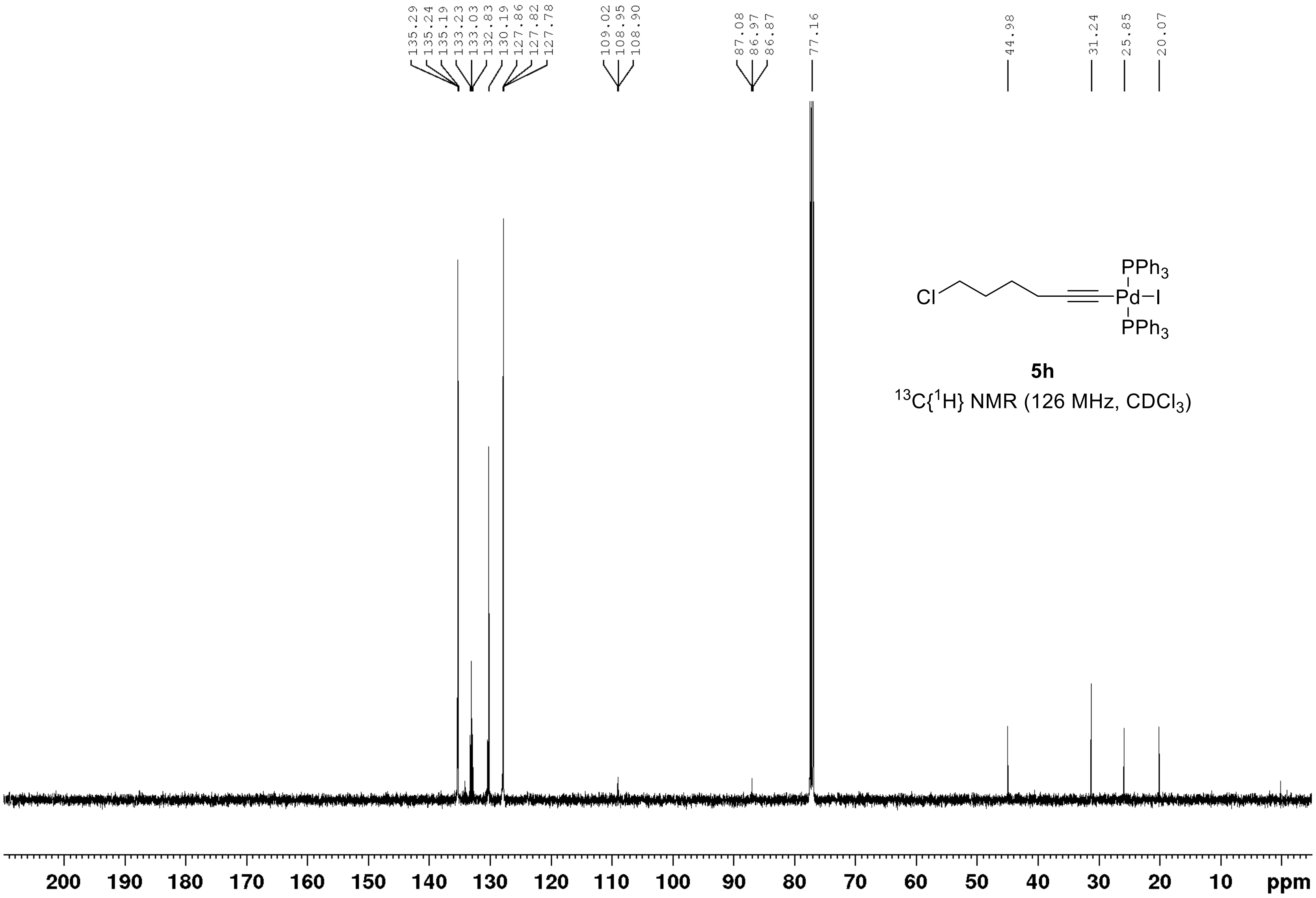
135.29
135.24
135.19
133.23
133.03
132.83
130.19
127.86
127.82
127.78
109.02
108.95
108.90
87.08
86.97
86.87
77.16
44.98
31.24
25.85
20.07
Cl
PPh3
Pd
I
PPh3
5h
13C{1H} NMR (126 MHz, CDCl3)
200
190
180
170
160
150
140
130
120
110
100
90
80
70
60
50
40
30
20
10
ppm

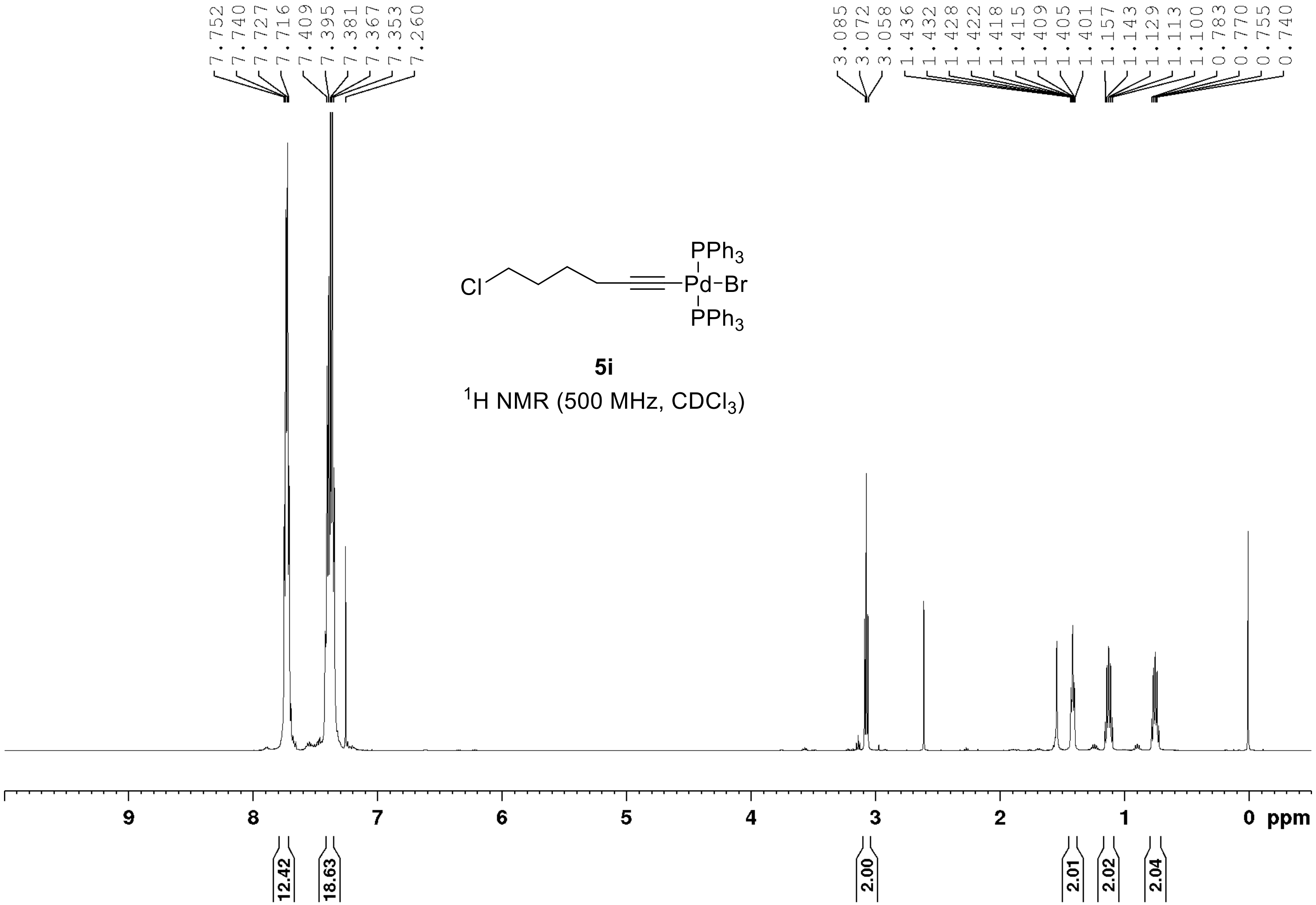

5i

^{1}H NMR (500 MHz, $CDCl_3$)

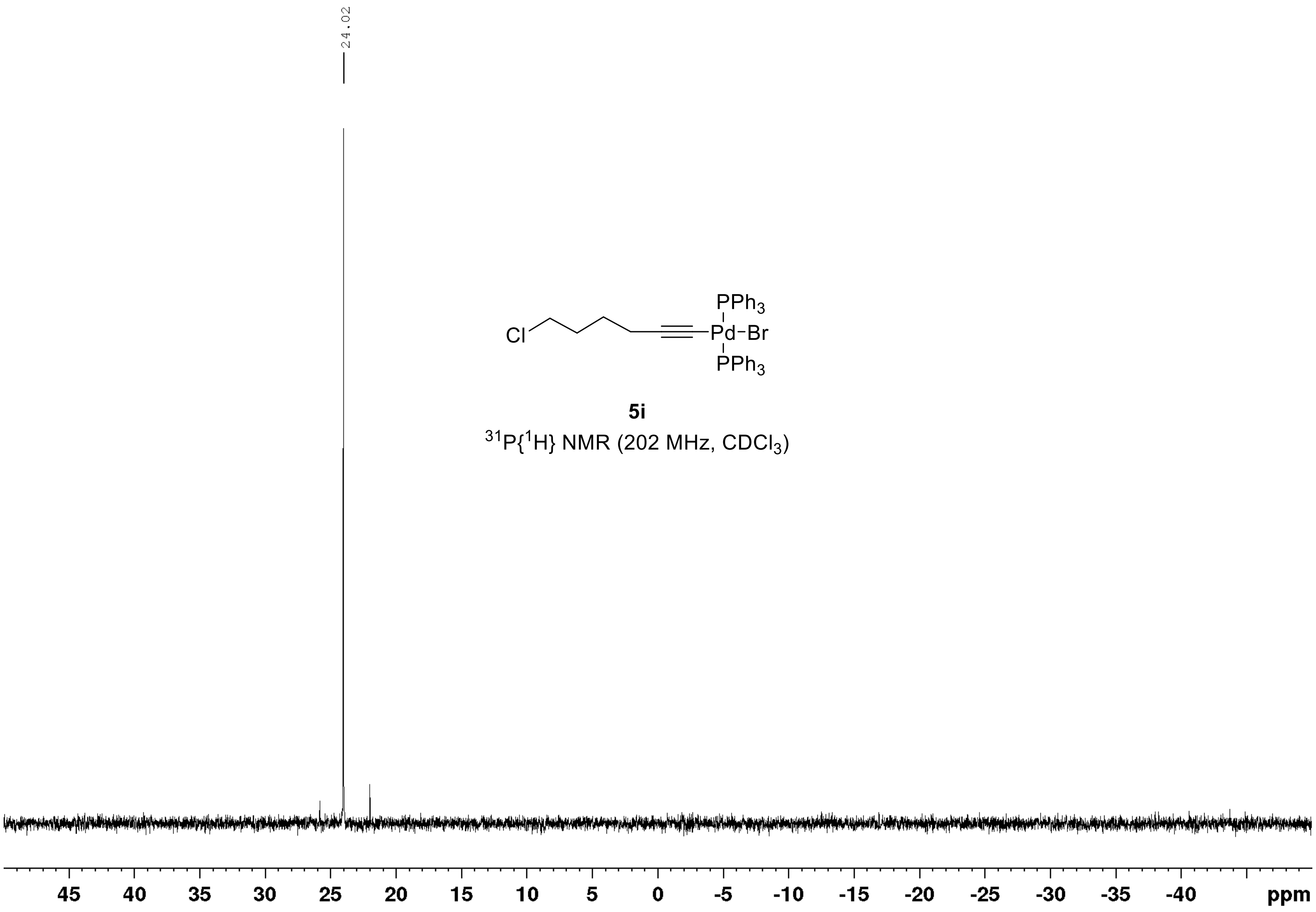

5i

$^{31}P\{^{1}H\}$ NMR (202 MHz, $CDCl_3$)

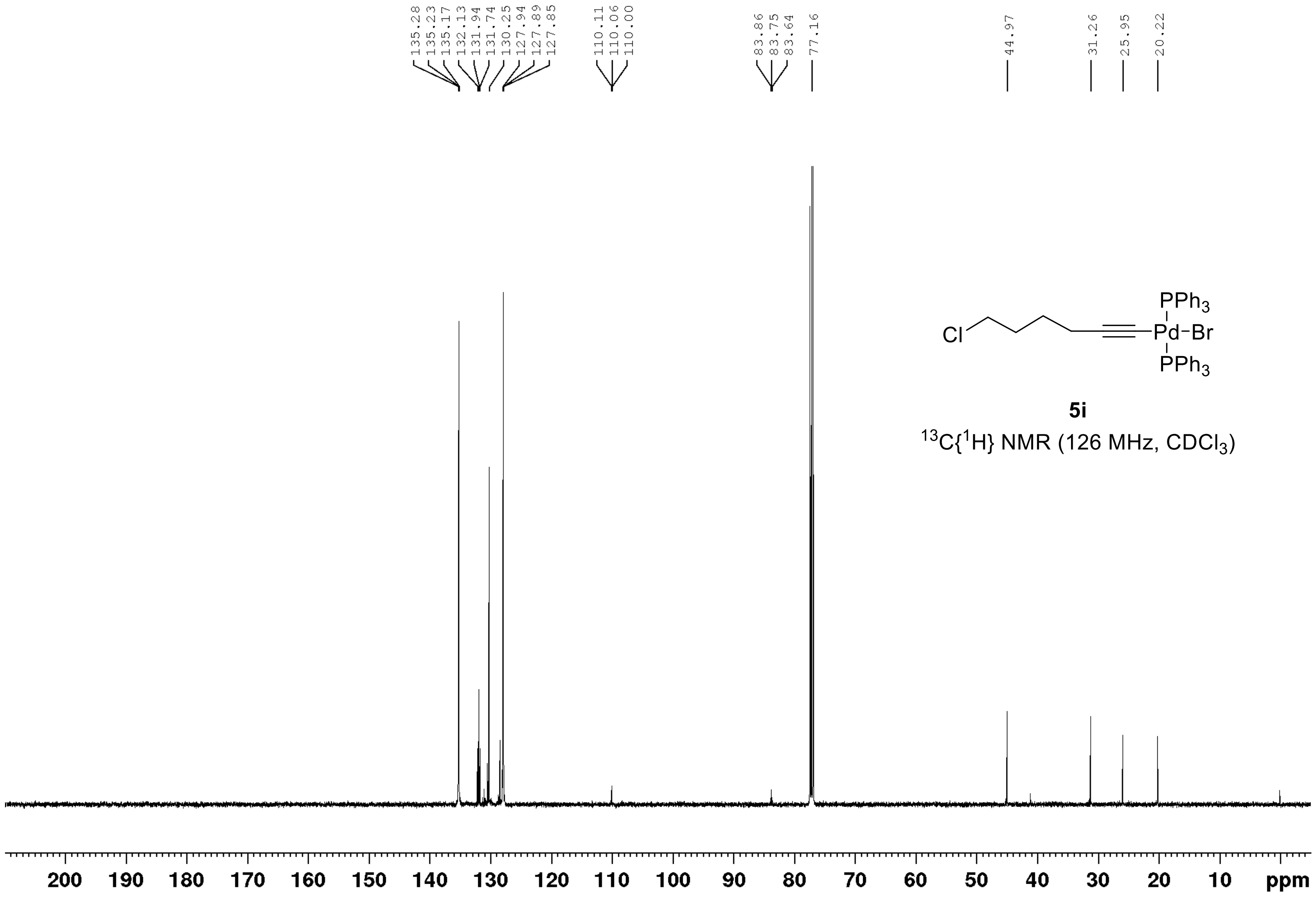

5i

$^{13}C\{^{1}H\}$ NMR (126 MHz, $CDCl_3$)

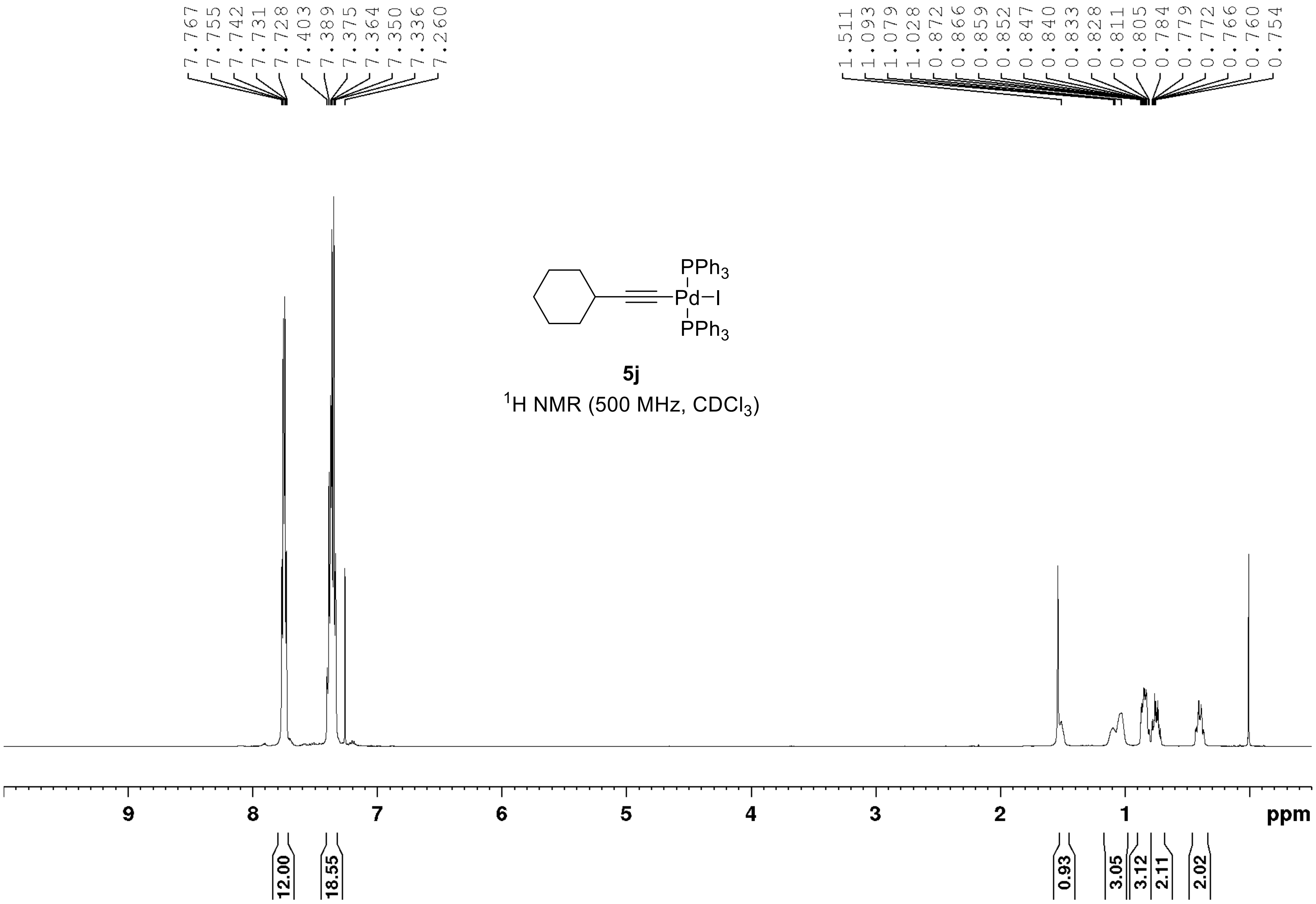
7.767
7.755
7.742
7.731
7.728
7.403
7.389
7.375
7.364
7.350
7.336
7.260
1.511
1.093
1.079
1.028
0.872
0.866
0.859
0.852
0.847
0.840
0.833
0.828
0.811
0.805
0.784
0.779
0.772
0.766
0.760
0.754
PPh3
Pd
I
PPh3
5j
1H NMR (500 MHz, CDCl3)
9
8
7
6
5
4
3
2
1
ppm
12.00
18.55
0.93
3.05
3.12
2.11
2.02

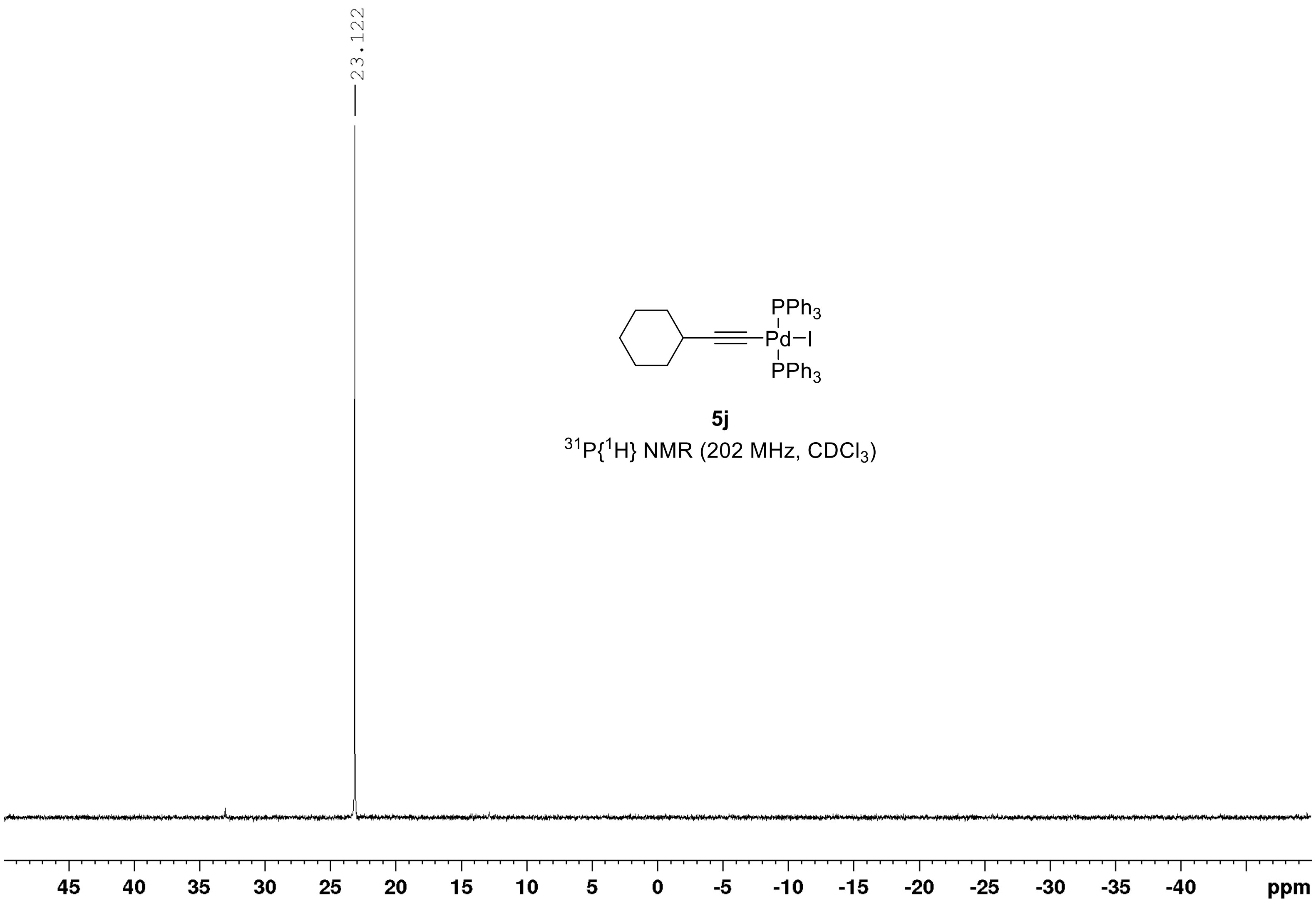
23.122
PPh3
Pd
I
PPh3
5j
31P{1H} NMR (202 MHz, CDCl3)
45 40 35 30 25 20 15 10 5 0 -5 -10 -15 -20 -25 -30 -35 -40 ppm

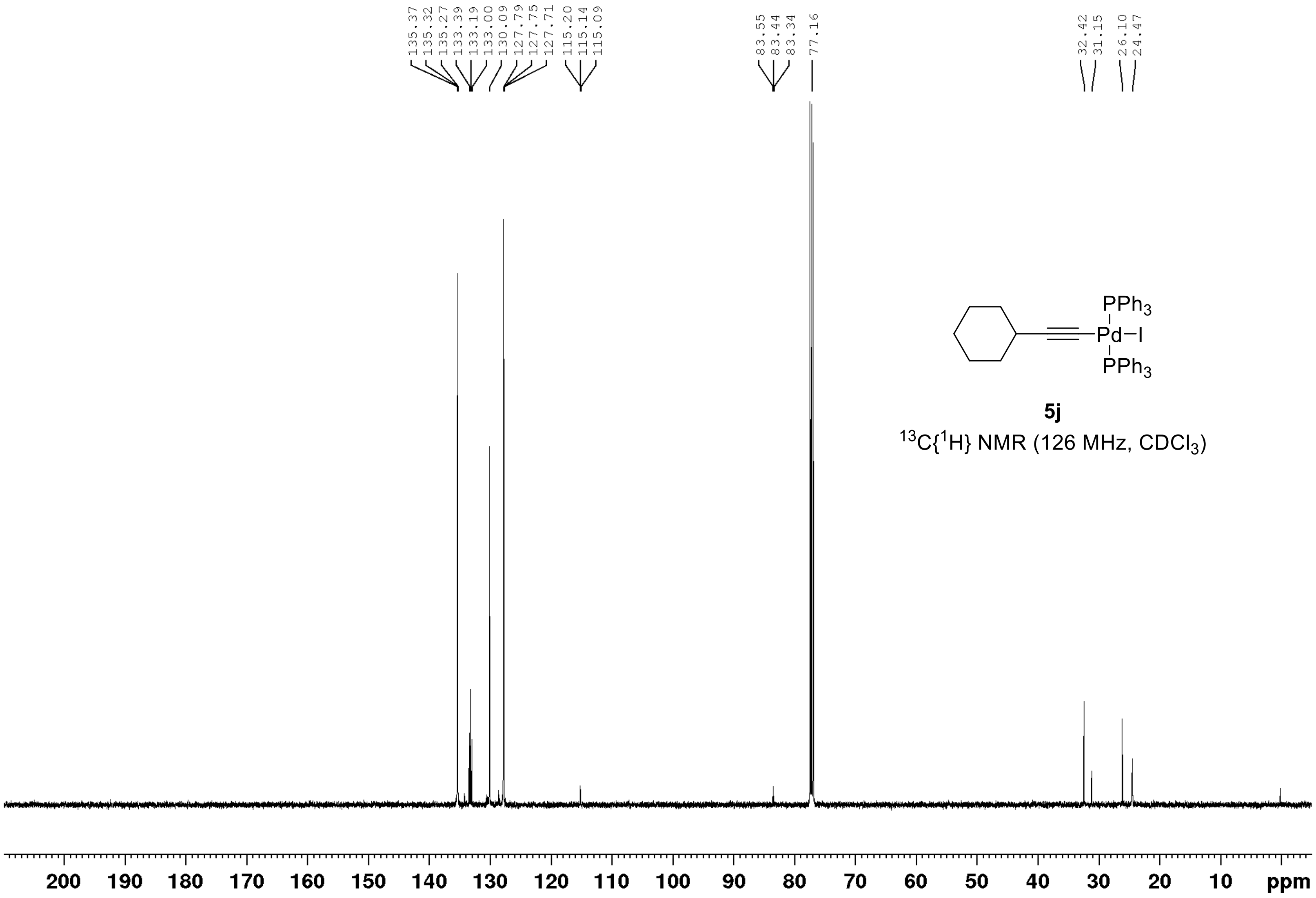

5j

$^{13}C\{^{1}H\}$ NMR (126 MHz, $CDCl_3$)

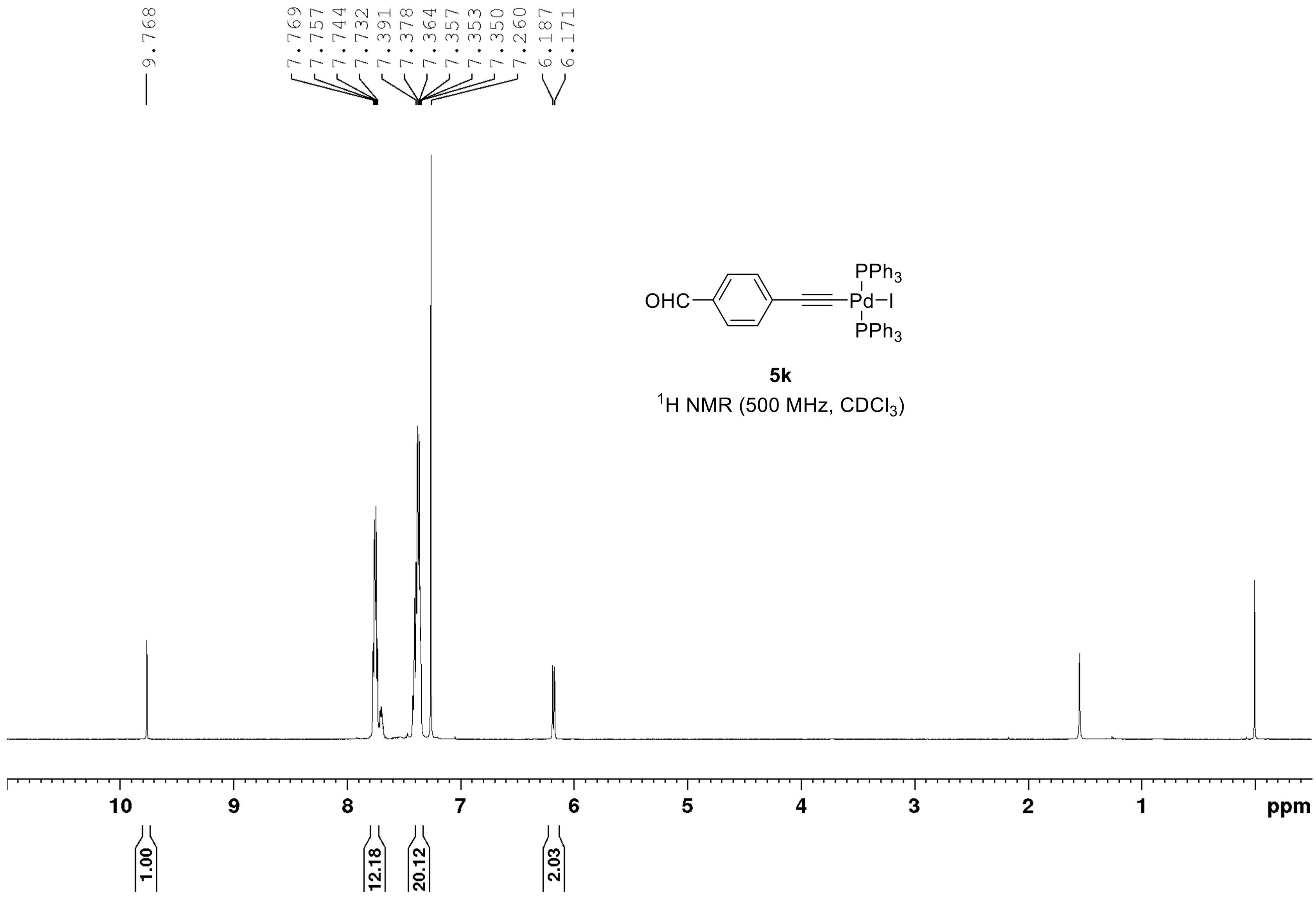
9.768
7.769
7.757
7.744
7.732
7.391
7.378
7.364
7.357
7.353
7.350
7.260
6.187
6.171
OHC
PPh3
Pd
I
PPh3
5k
1H NMR (500 MHz, CDCl3)
10
9
8
7
6
5
4
3
2
1
ppm
1.00
12.18
20.12
2.03

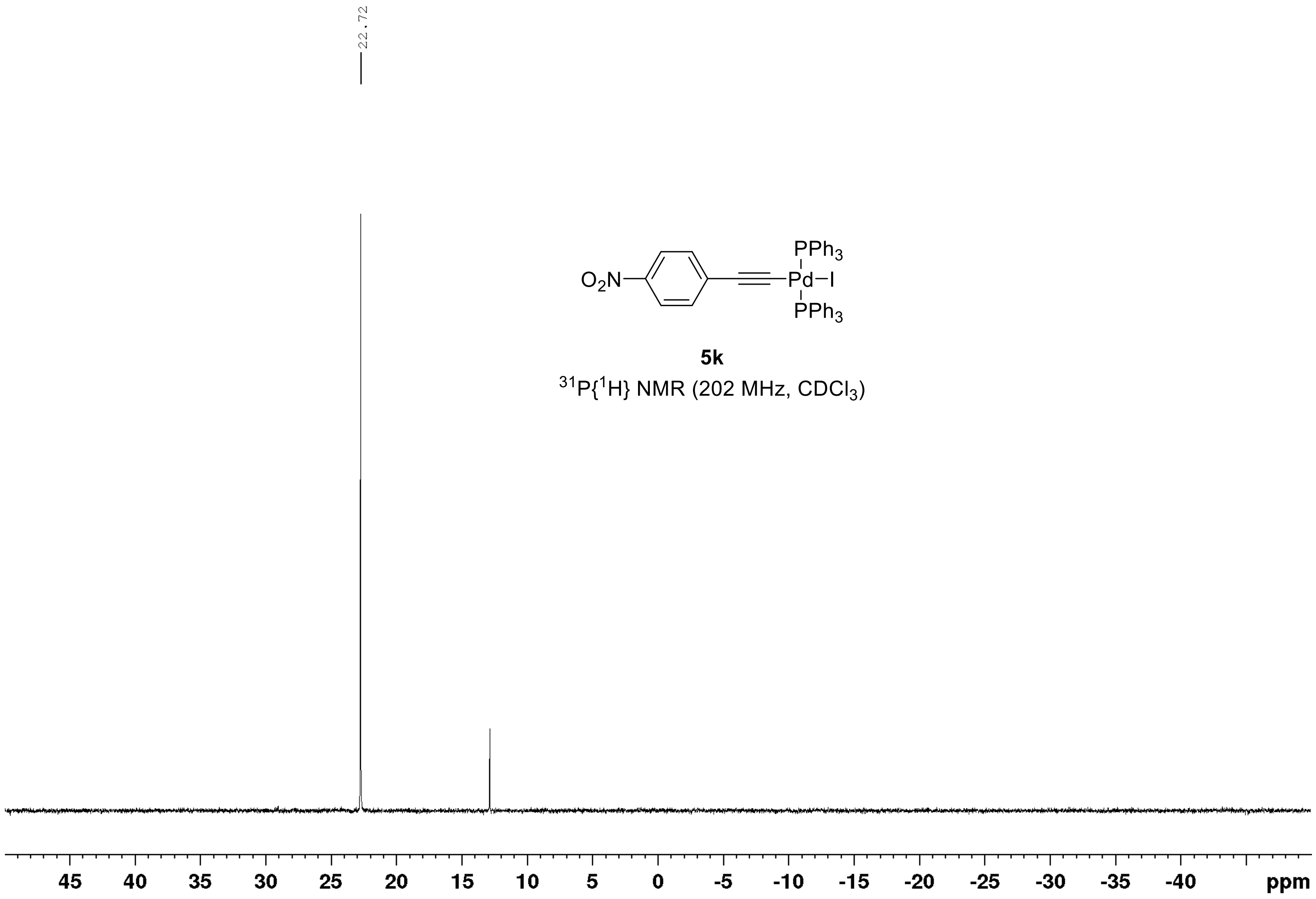

5k

$^{31}P\{^1H\}$ NMR (202 MHz, $CDCl_3$)

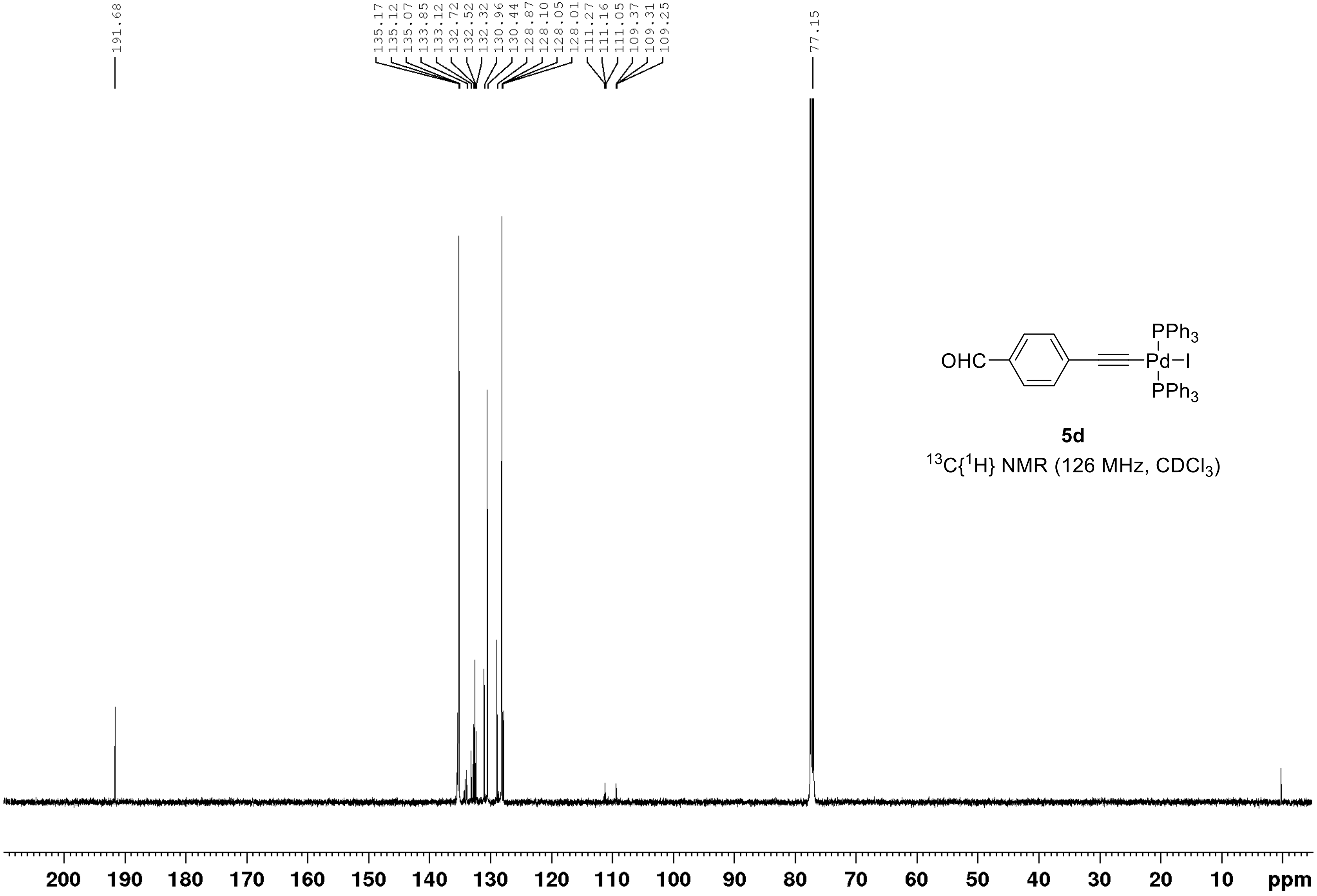

5d

$^{13}C\{^1H\}$ NMR (126 MHz, $CDCl_3$)

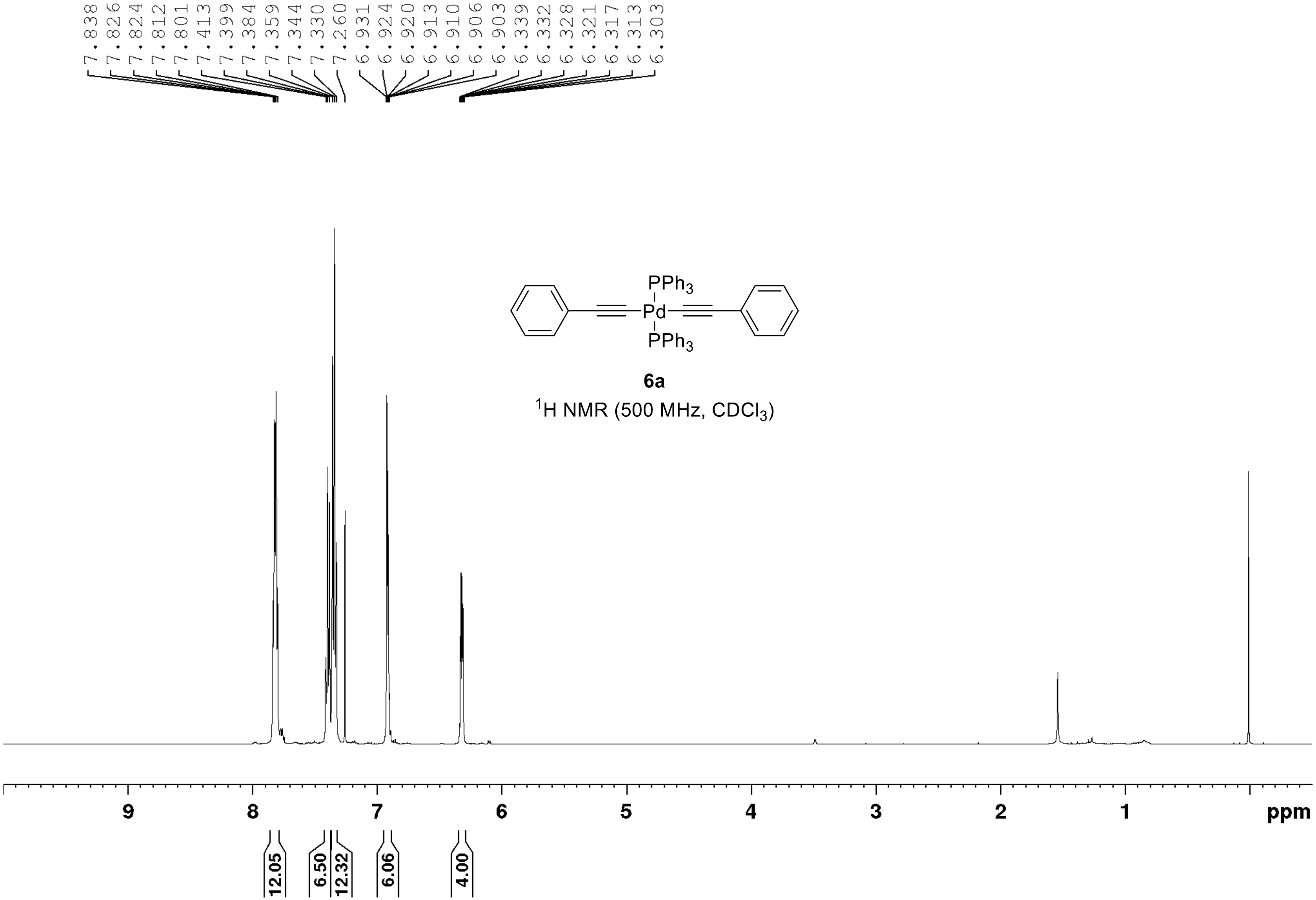
7.838
7.826
7.824
7.812
7.801
7.413
7.399
7.384
7.359
7.344
7.330
7.260
6.931
6.924
6.920
6.913
6.910
6.906
6.903
6.339
6.332
6.328
6.321
6.317
6.313
6.303
PPh3
Pd
PPh3
6a
1H NMR (500 MHz, CDCl3)
9
8
7
6
5
4
3
2
1
ppm
12.05
6.50
12.32
6.06
4.00

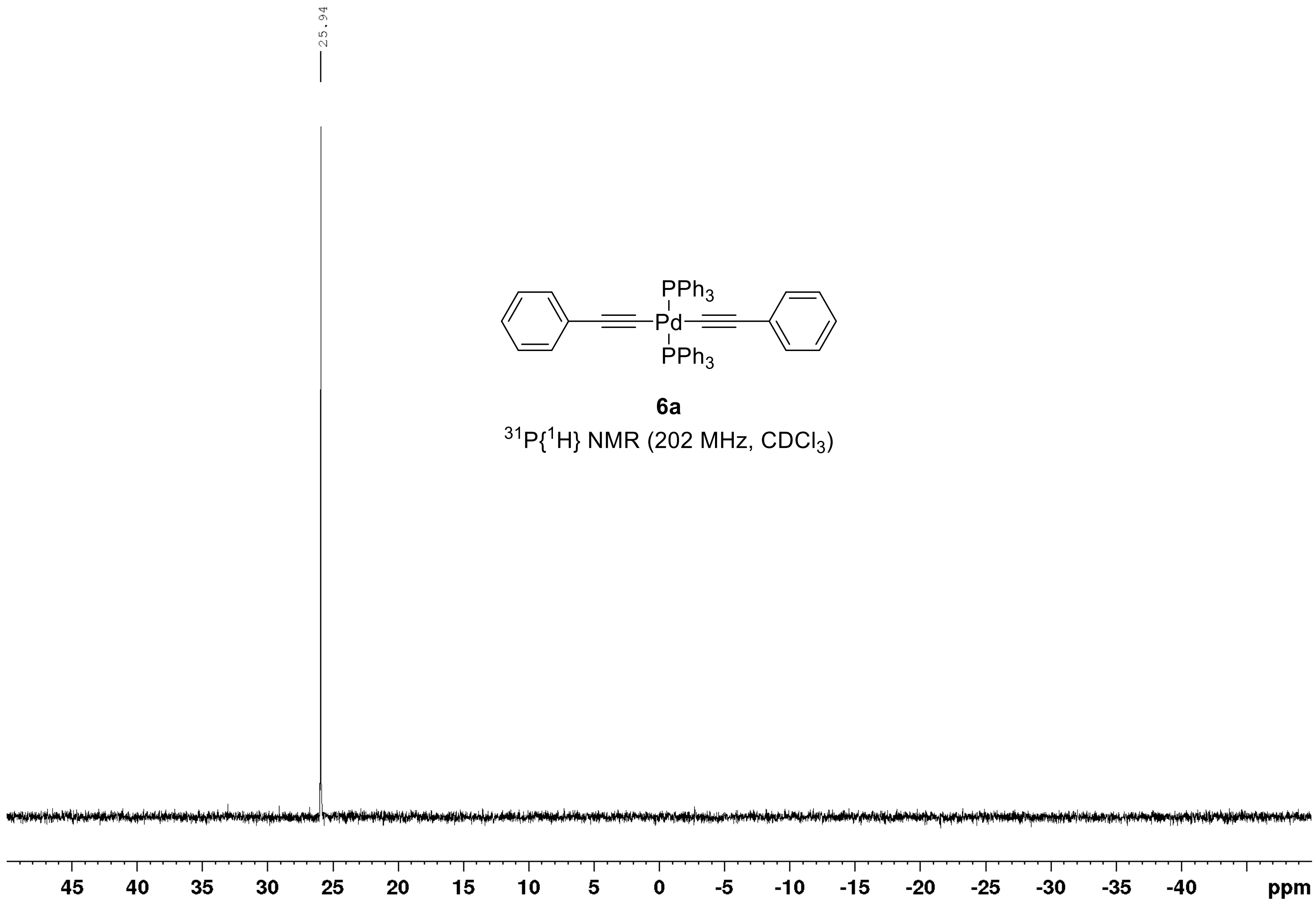

6a

$^{31}P\{^{1}H\}$ NMR (202 MHz, $CDCl_3$)

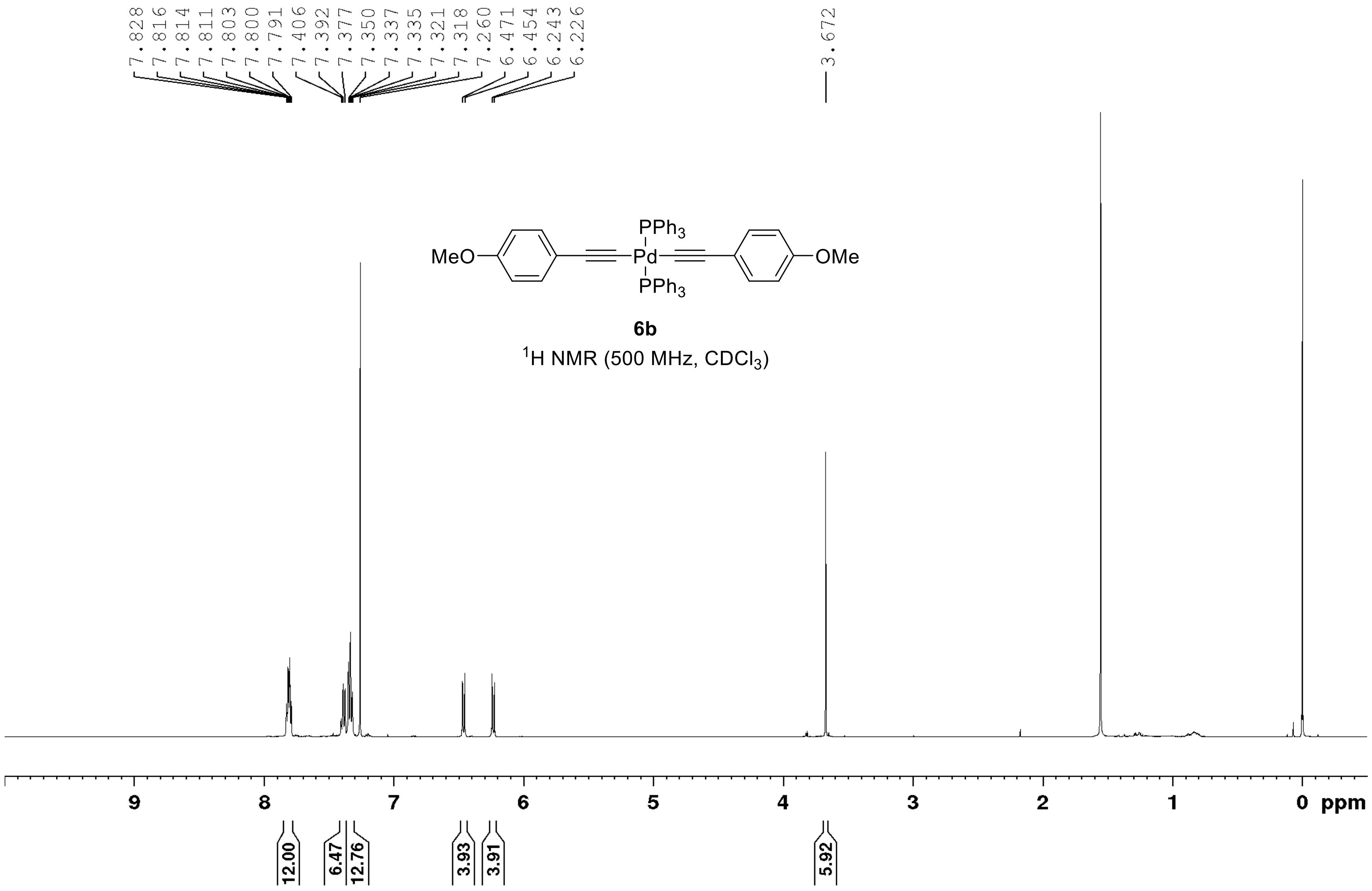
7.828
7.816
7.814
7.811
7.803
7.800
7.791
7.406
7.392
7.377
7.350
7.337
7.335
7.321
7.318
7.260
6.471
6.454
6.243
6.226
3.672
MeO
PPh3
Pd
PPh3
OMe
6b
1H NMR (500 MHz, CDCl3)
9
8
7
6
5
4
3
2
1
0 ppm
12.00
6.47
12.76
3.93
3.91
5.92

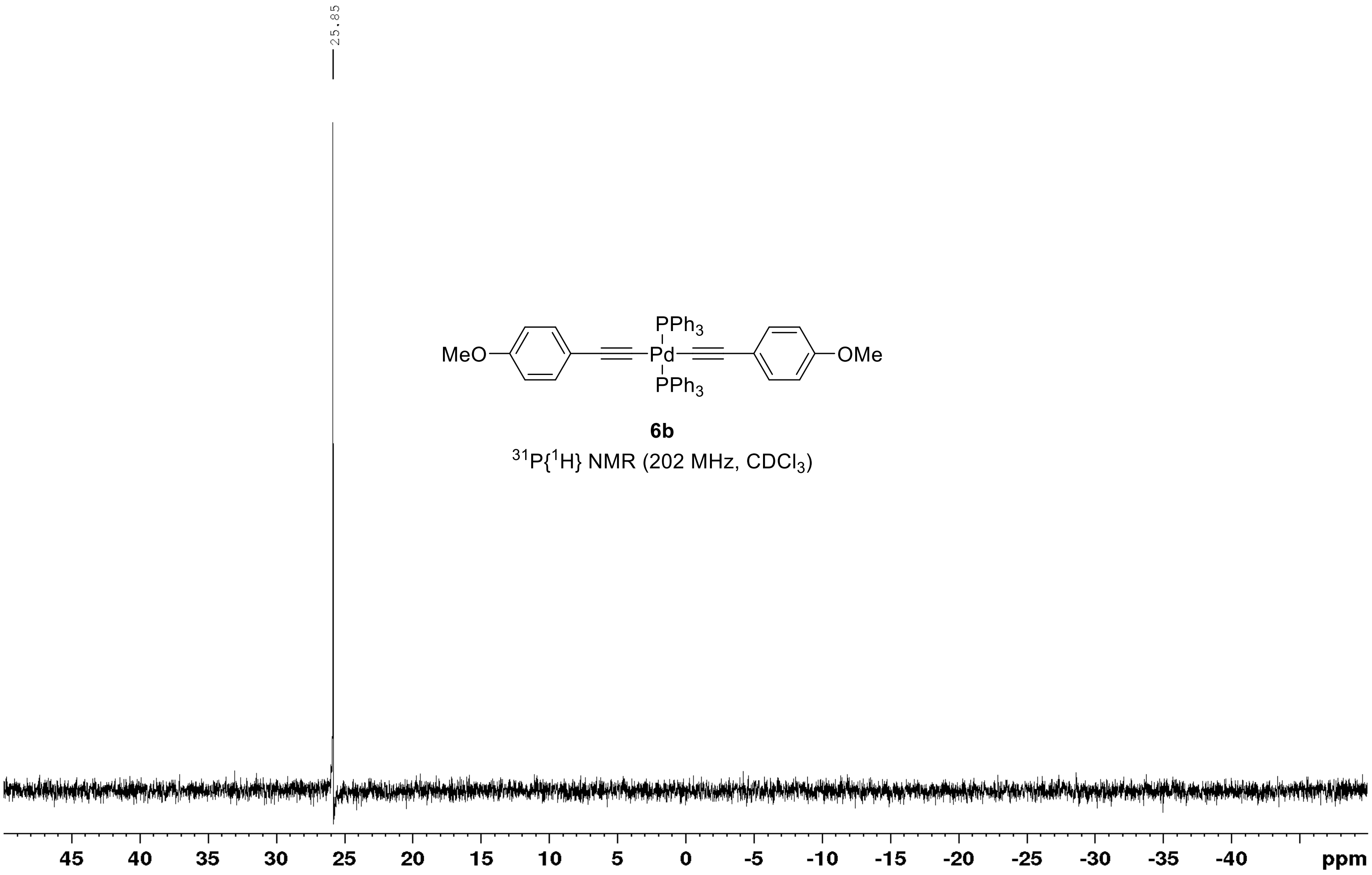
25.85
MeO
OMe
PPh3
Pd
PPh3
6b
31P{1H} NMR (202 MHz, CDCl3)
45 40 35 30 25 20 15 10 5 0 -5 -10 -15 -20 -25 -30 -35 -40 ppm

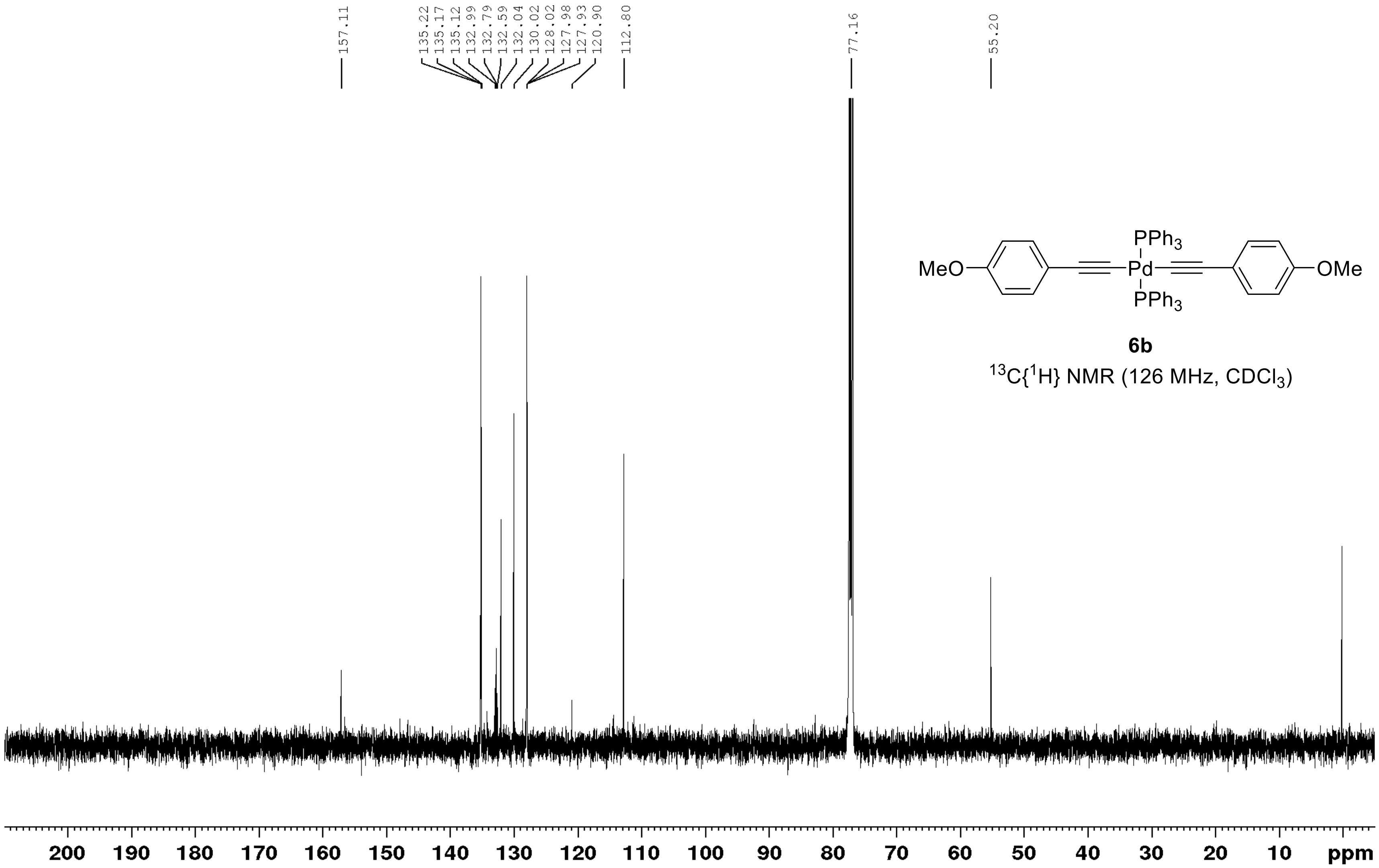

6b

$^{13}C\{^1H\}$ NMR (126 MHz, $CDCl_3$)

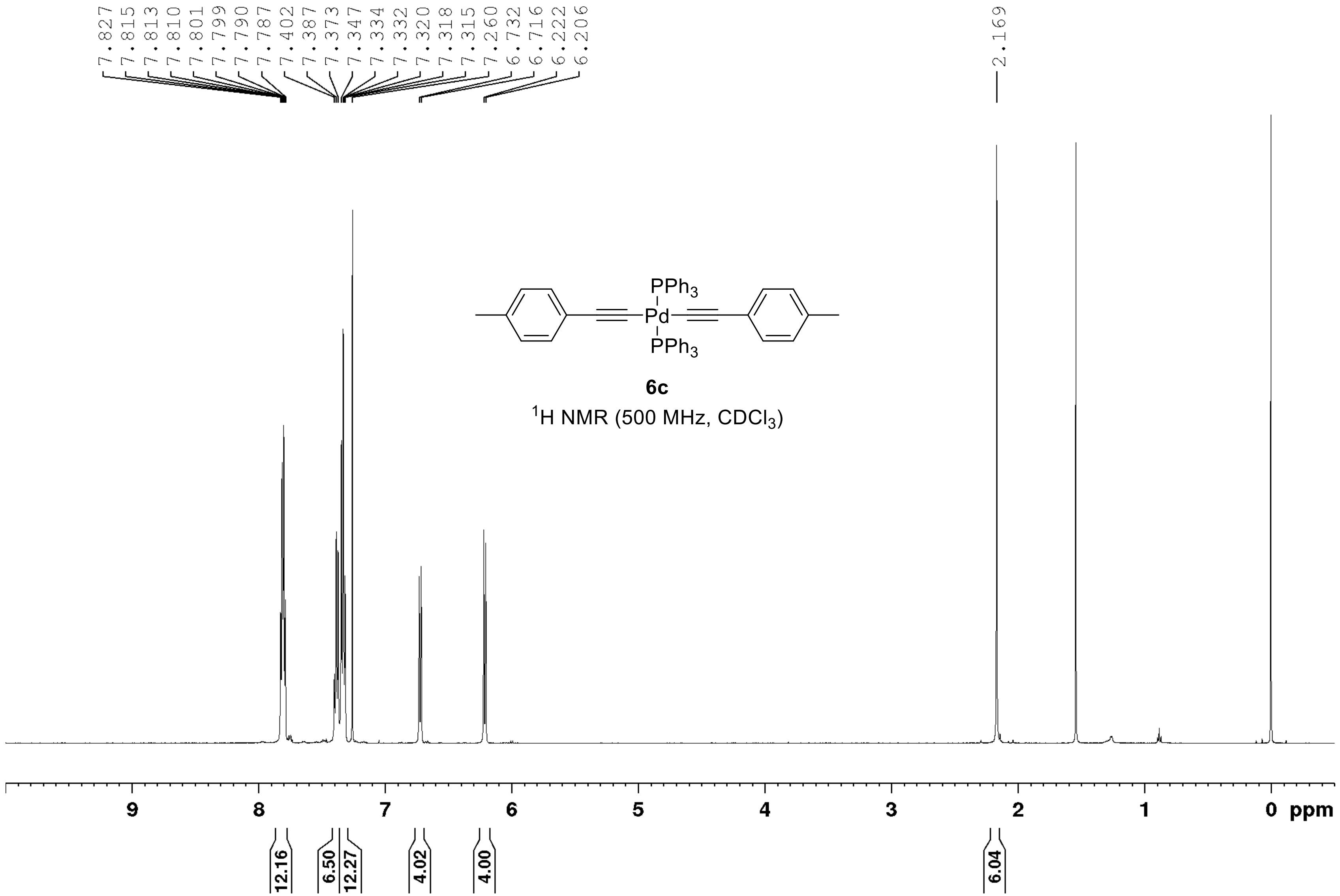

7.827
7.815
7.813
7.810
7.801
7.799
7.790
7.787
7.402
7.387
7.373
7.347
7.334
7.332
7.320
7.318
7.315
7.260
6.732
6.716
6.222
6.206
2.169
PPh3
Pd
PPh3
6c
1H NMR (500 MHz, CDCl3)
9
8
7
6
5
4
3
2
1
0 ppm
12.16
6.50
12.27
4.02
4.00
6.04

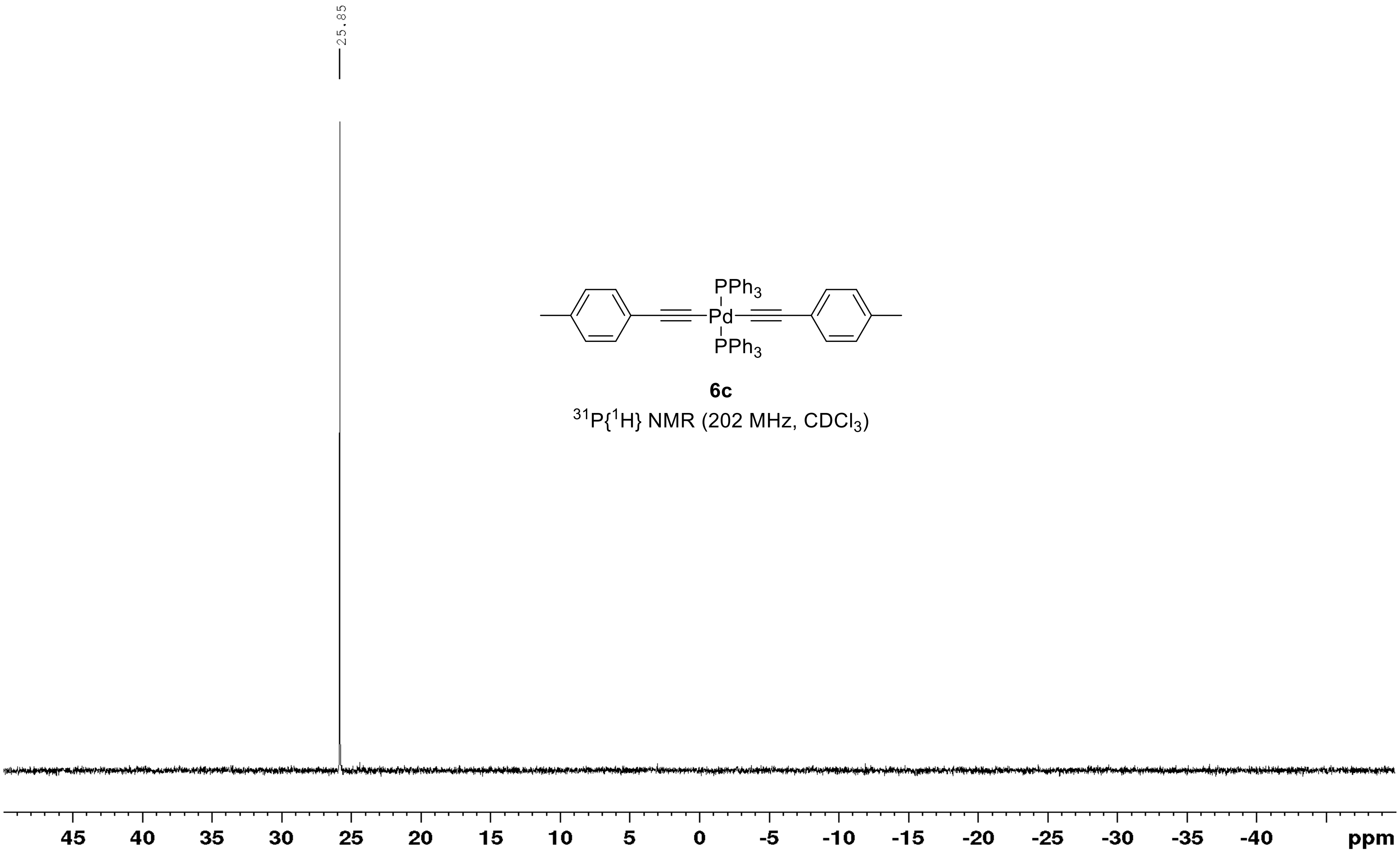

6c

$^{31}P\{^{1}H\}$ NMR (202 MHz, $CDCl_3$)

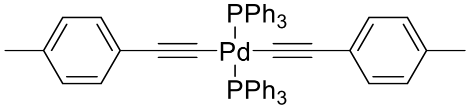

6c

$^{13}C\{^{1}H\}$ NMR (126 MHz, $CDCl_3$)

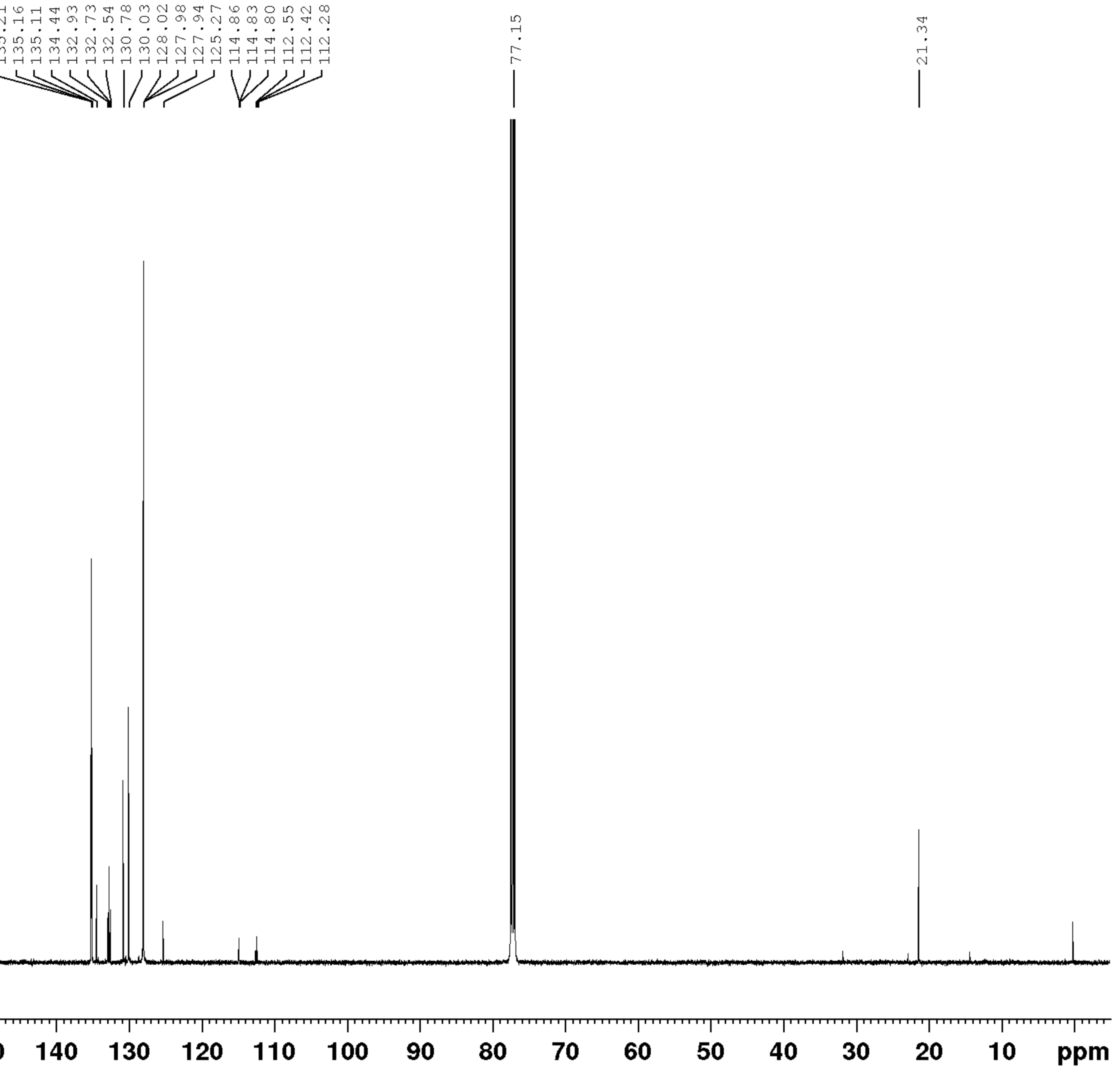

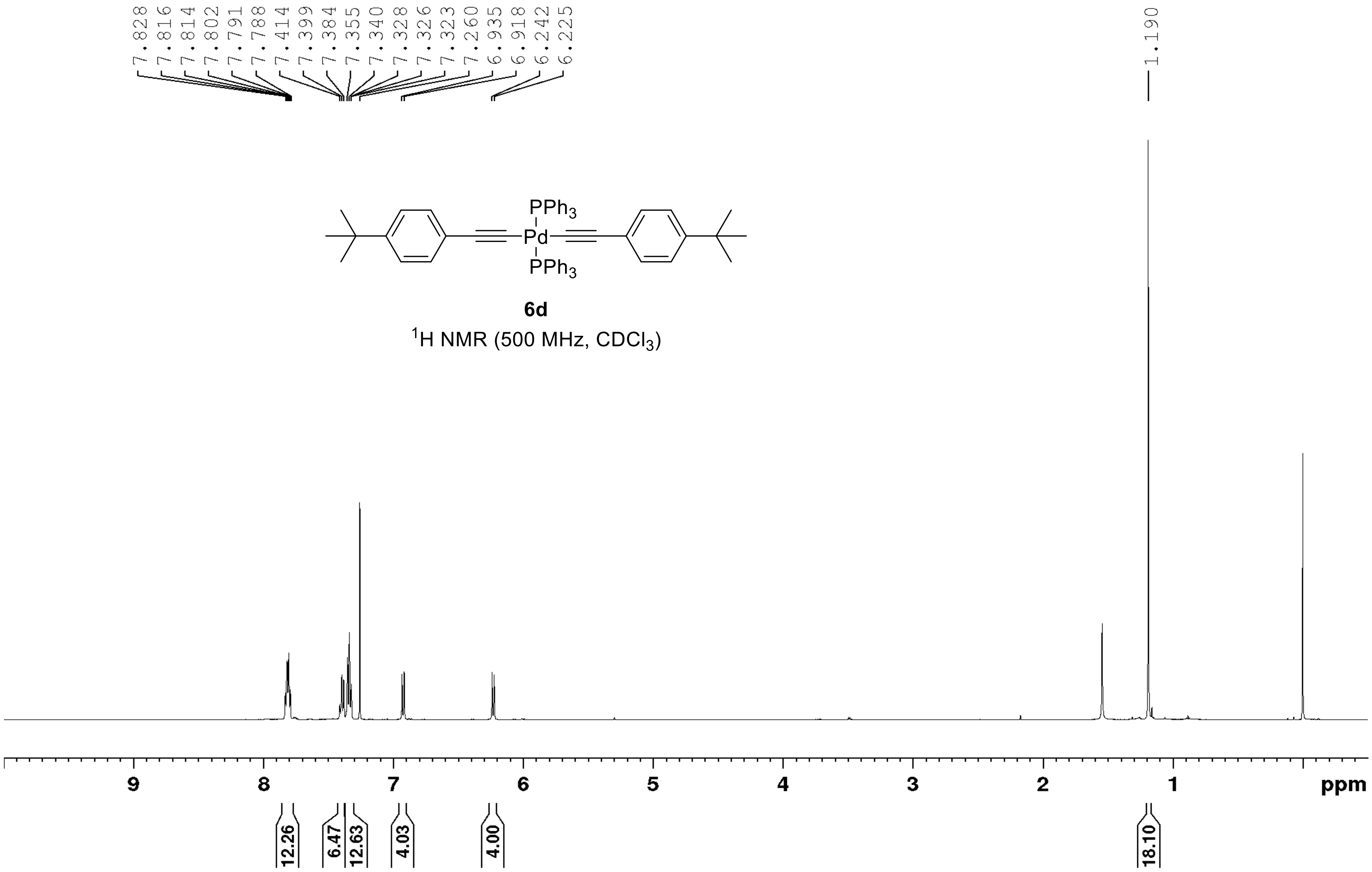
7.828
7.816
7.814
7.802
7.791
7.788
7.414
7.399
7.384
7.355
7.340
7.328
7.326
7.323
7.260
6.935
6.918
6.242
6.225
1.190
PPh3
Pd
PPh3
6d
1H NMR (500 MHz, CDCl3)
9
8
7
6
5
4
3
2
1
ppm
12.26
6.47
12.63
4.03
4.00
18.10

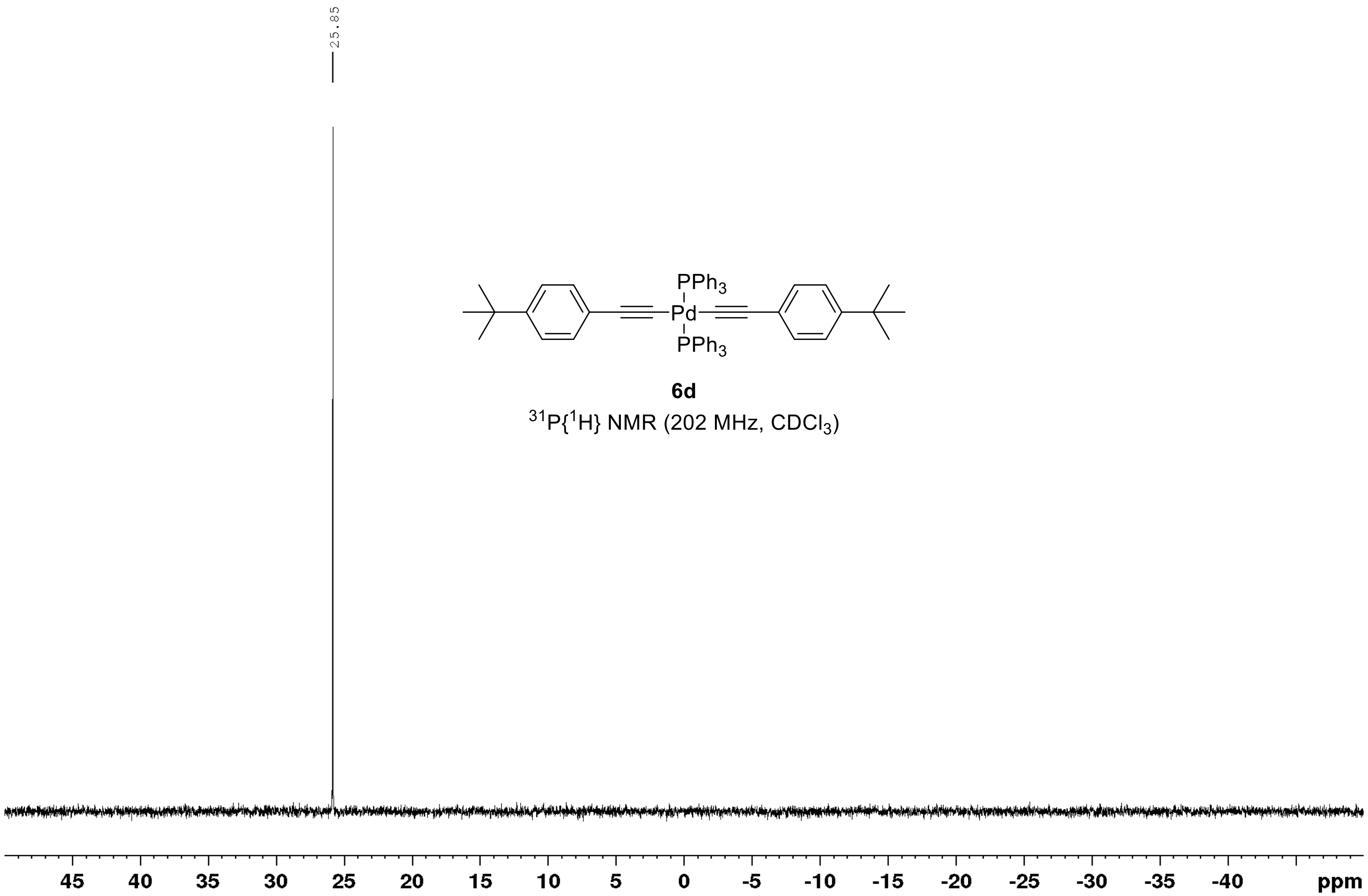

6d

$^{31}P\{^{1}H\}$ NMR (202 MHz, $CDCl_3$)

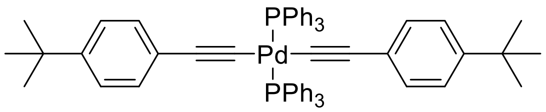

6d

$^{13}C\{^1H\}$ NMR (126 MHz, $CDCl_3$)

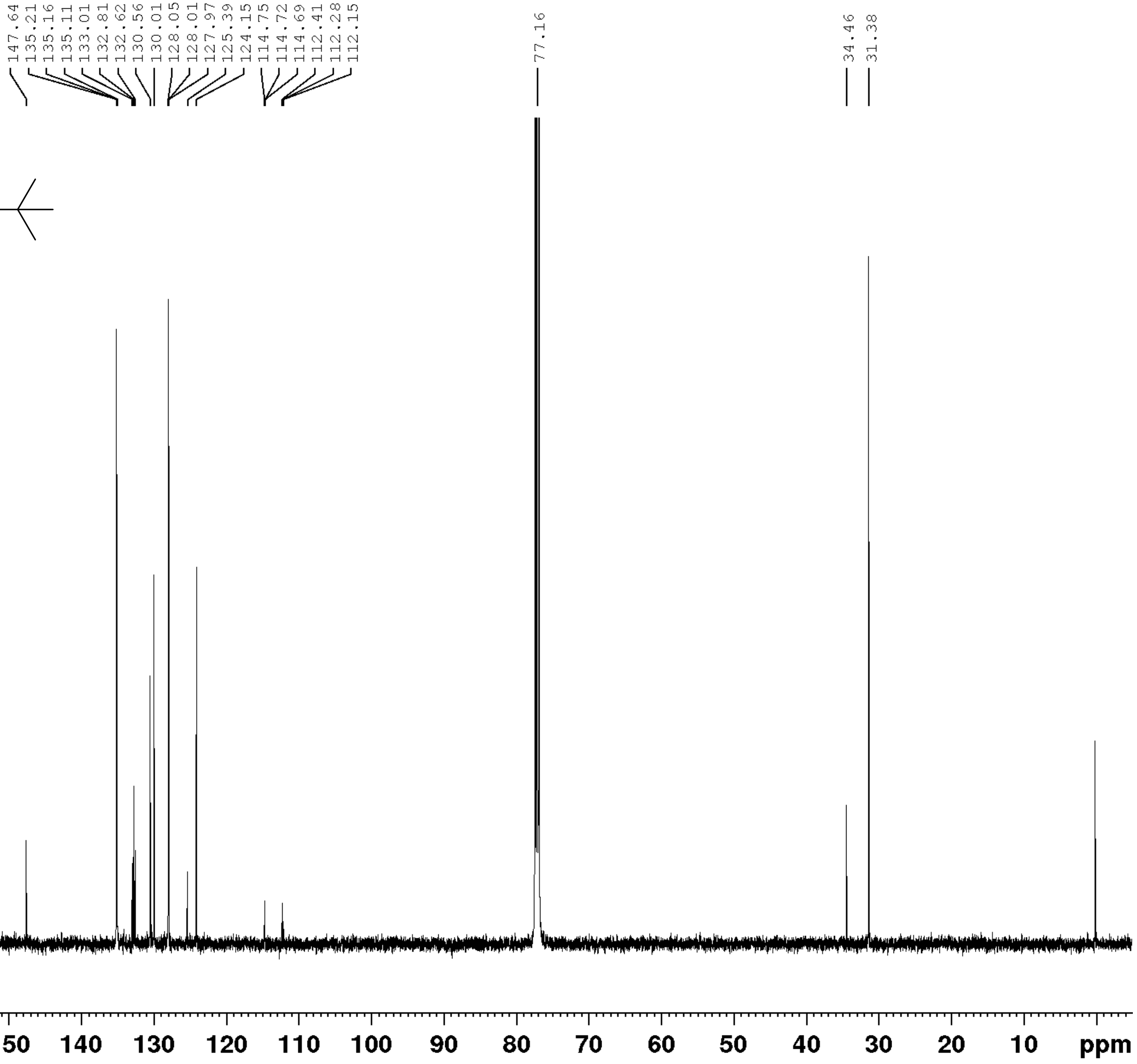

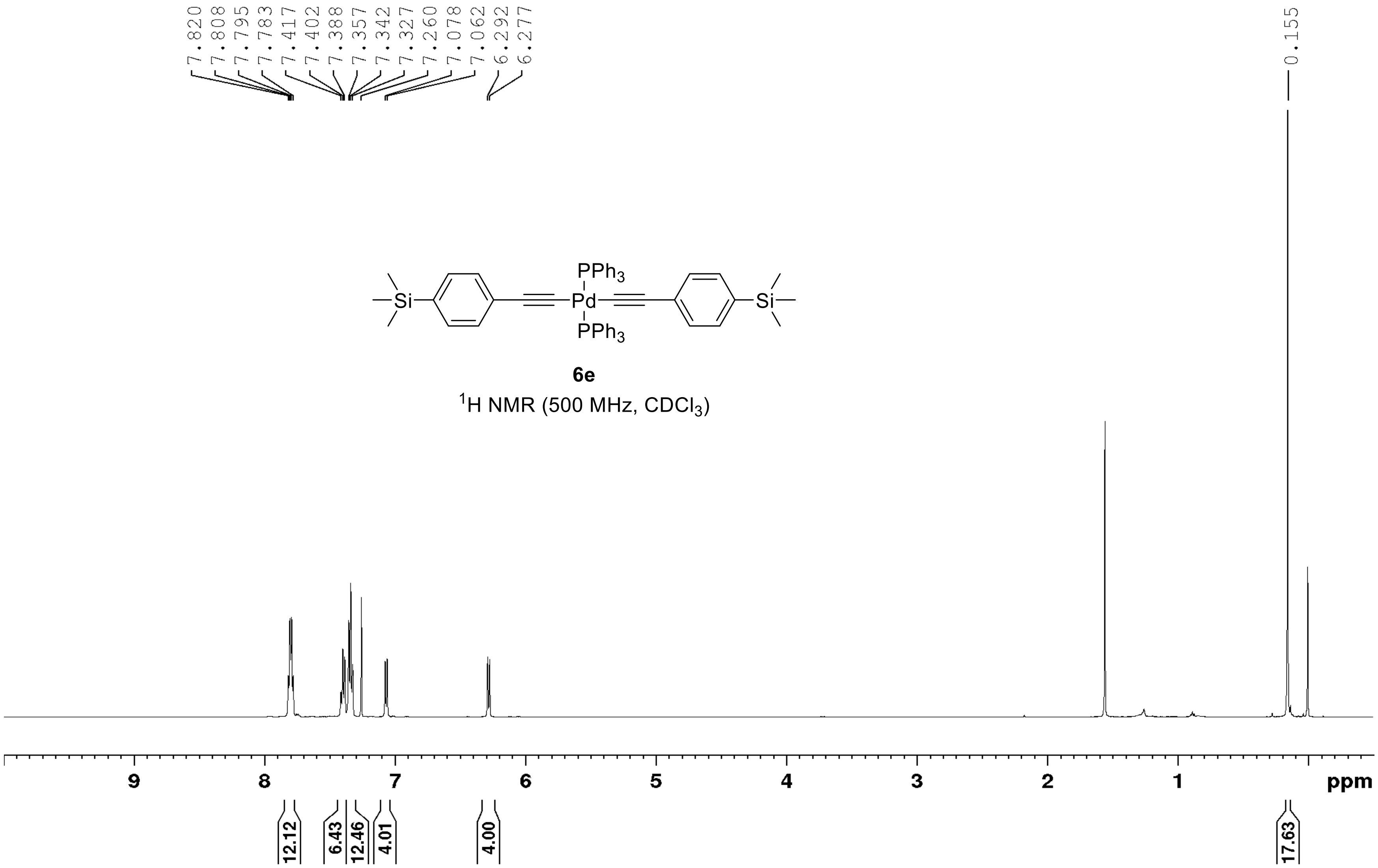
7.820
7.808
7.795
7.783
7.417
7.402
7.388
7.357
7.342
7.327
7.260
7.078
7.062
6.292
6.277
0.155
PPh3
Si
Pd
PPh3
Si
6e
1H NMR (500 MHz, CDCl3)
9
8
7
6
5
4
3
2
1
ppm
12.12
6.43
12.46
4.01
4.00
17.63

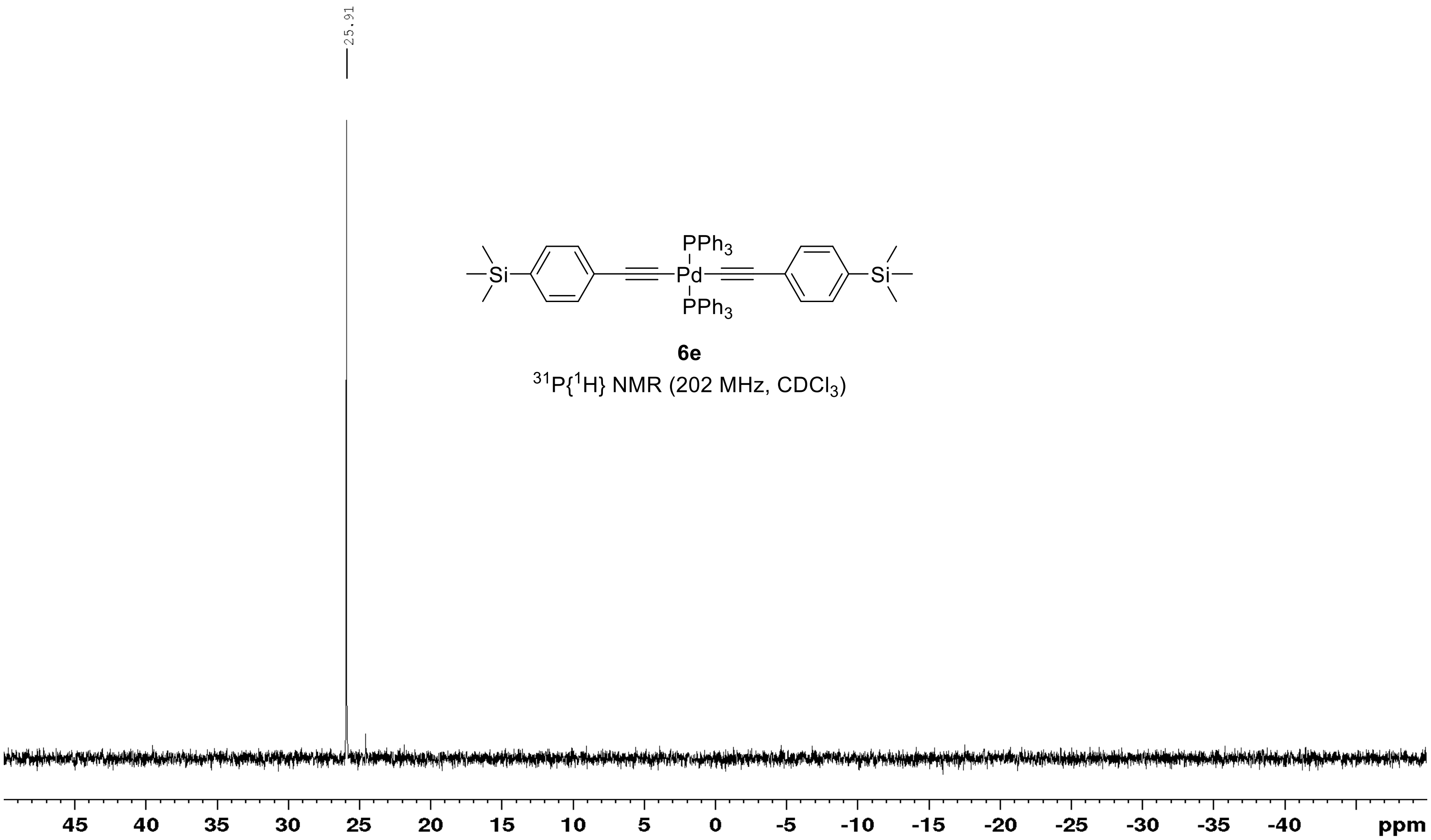

6e

$^{31}P\{^{1}H\}$ NMR (202 MHz, $CDCl_3$)

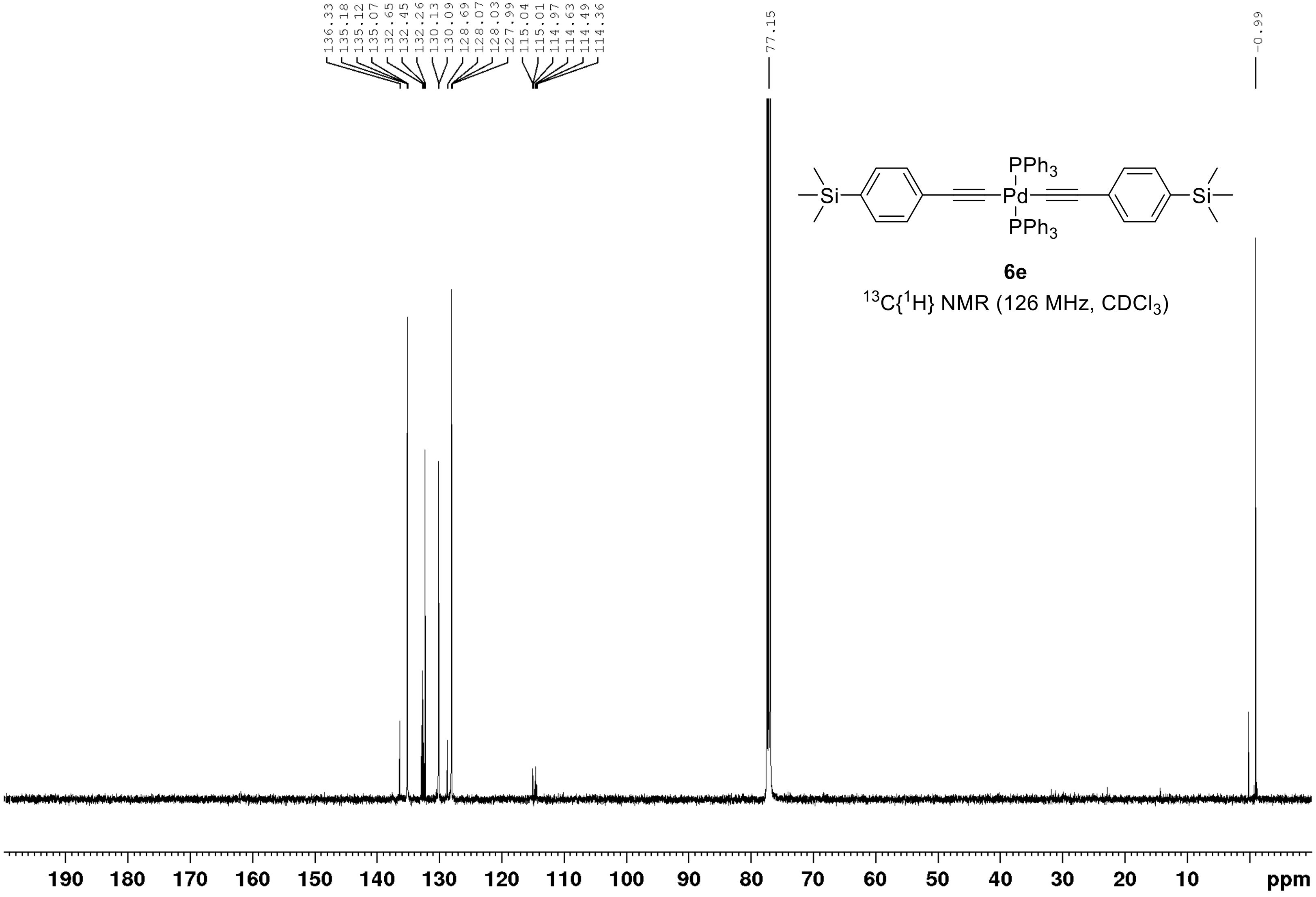

6e

$^{13}C\{^1H\}$ NMR (126 MHz, $CDCl_3$)

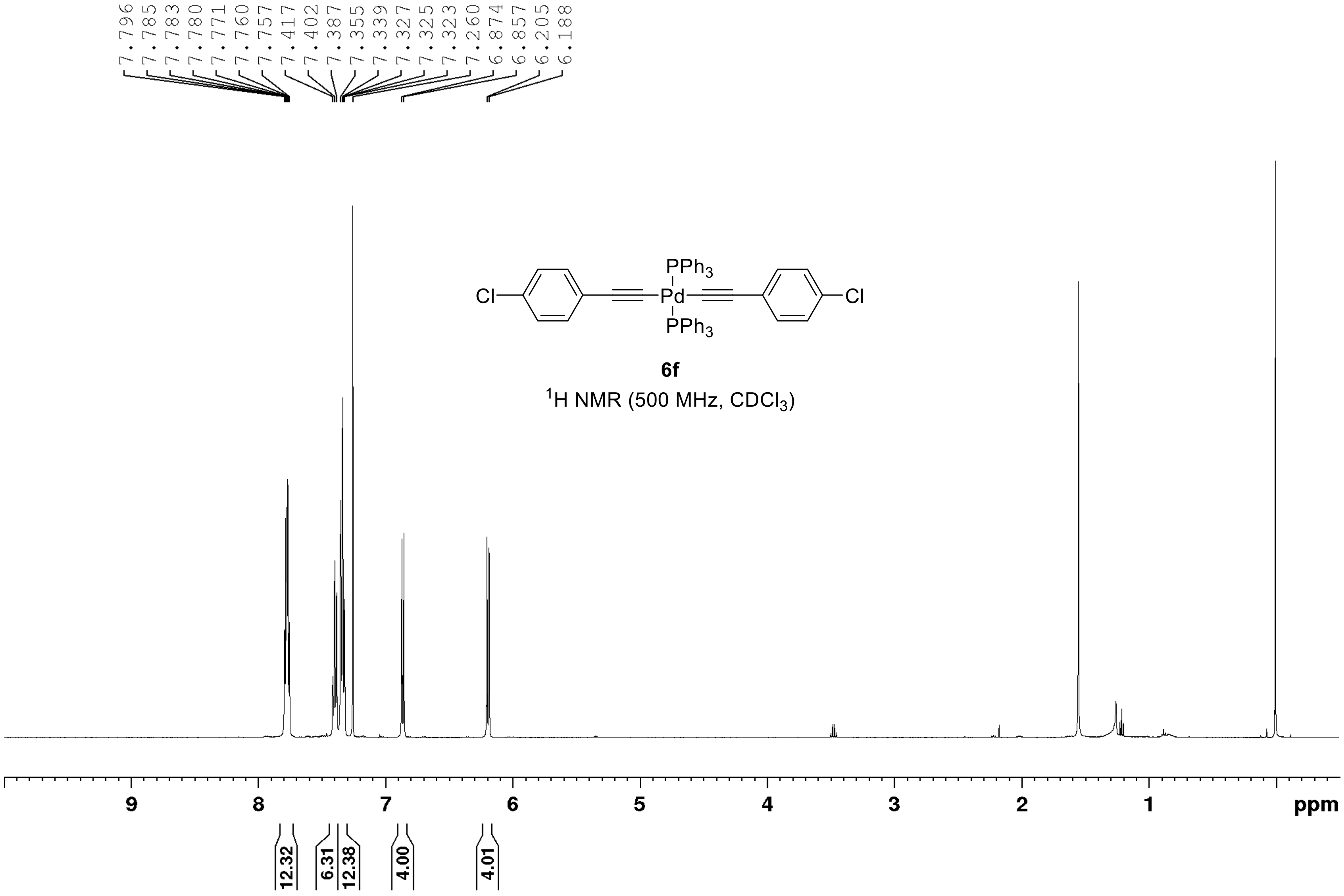
7.796
7.785
7.783
7.780
7.771
7.760
7.757
7.417
7.402
7.387
7.355
7.339
7.327
7.325
7.323
7.260
6.874
6.857
6.205
6.188
PPh3
Cl
Pd
PPh3
Cl
6f
1H NMR (500 MHz, CDCl3)
9
8
7
6
5
4
3
2
1
ppm
12.32
6.31
12.38
4.00
4.01

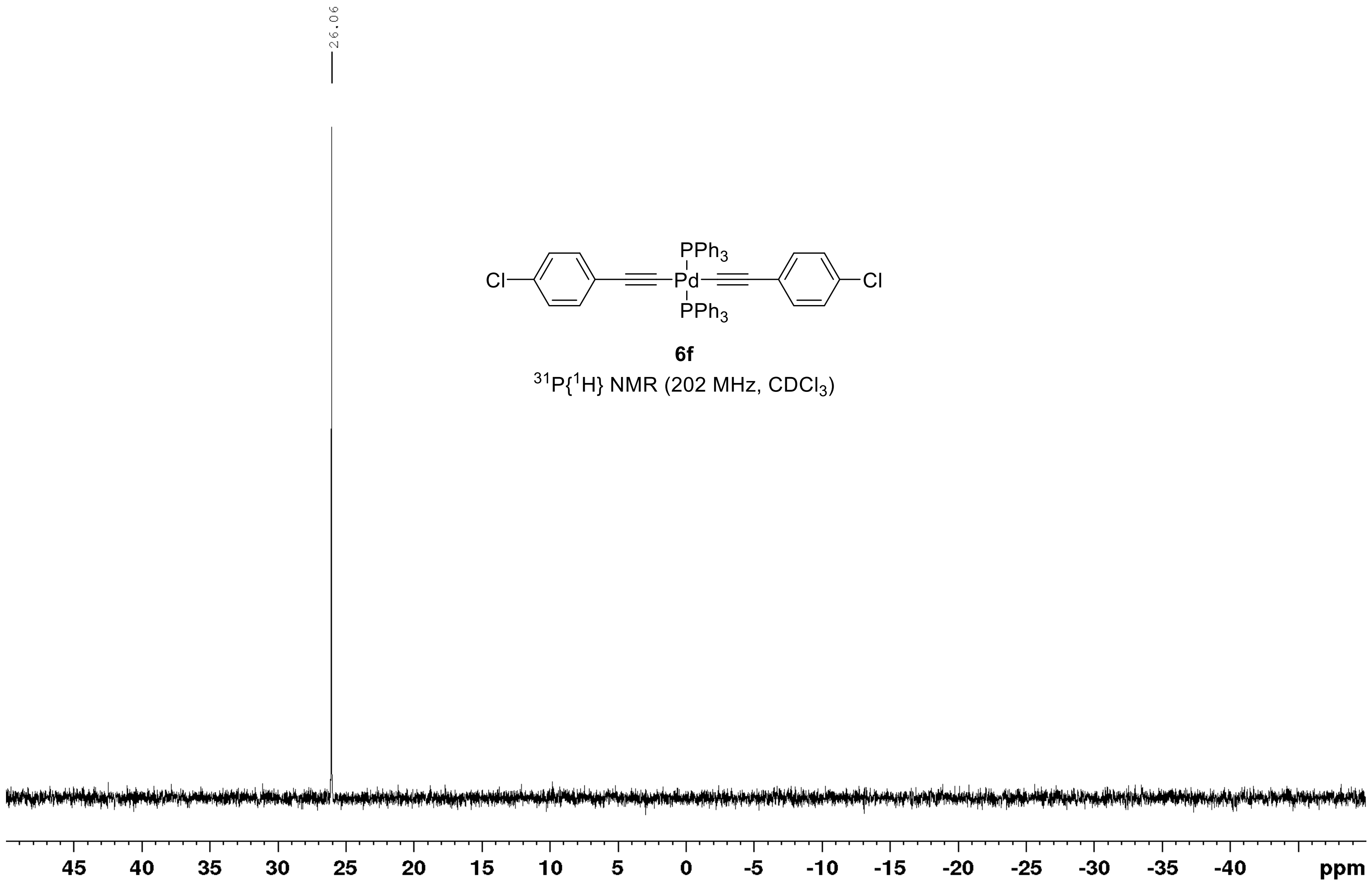

6f

$^{31}P\{^{1}H\}$ NMR (202 MHz, $CDCl_3$)

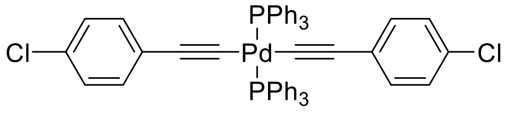

6f

$^{13}C\{^1H\}$ NMR (126 MHz, $CDCl_3$)

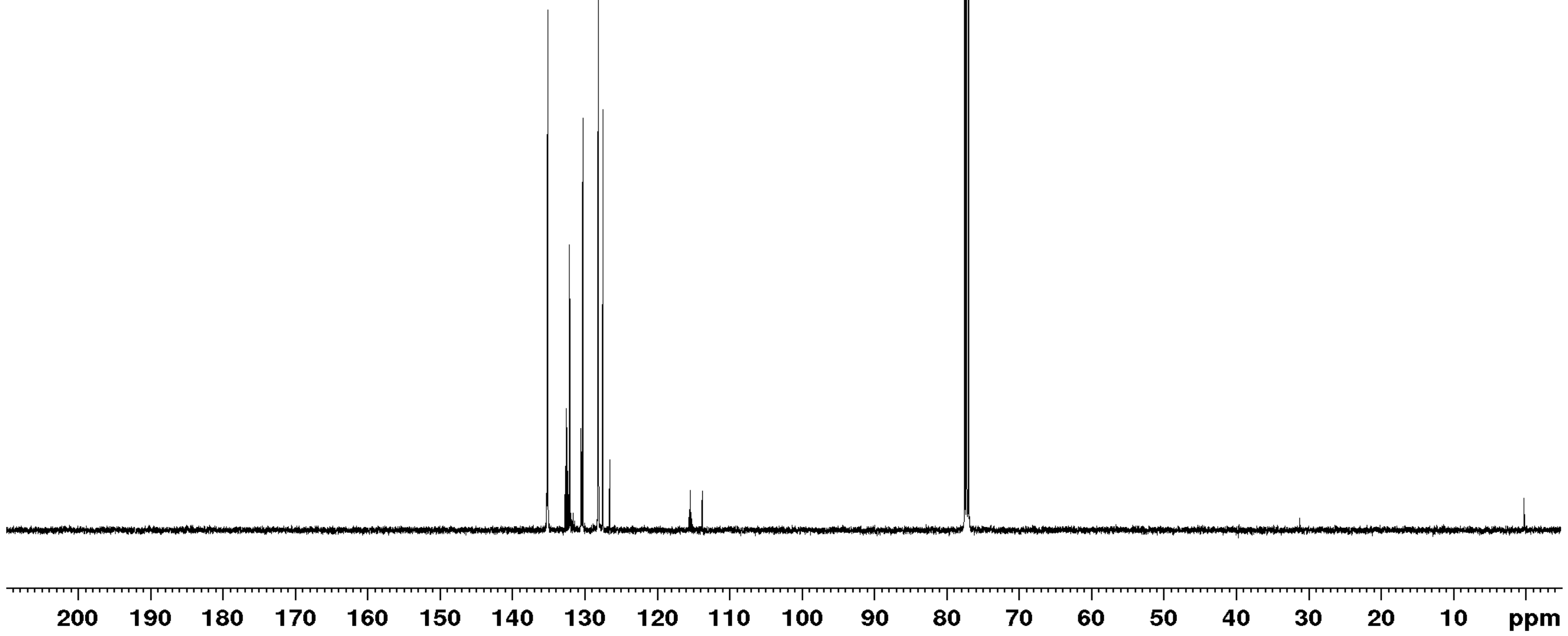

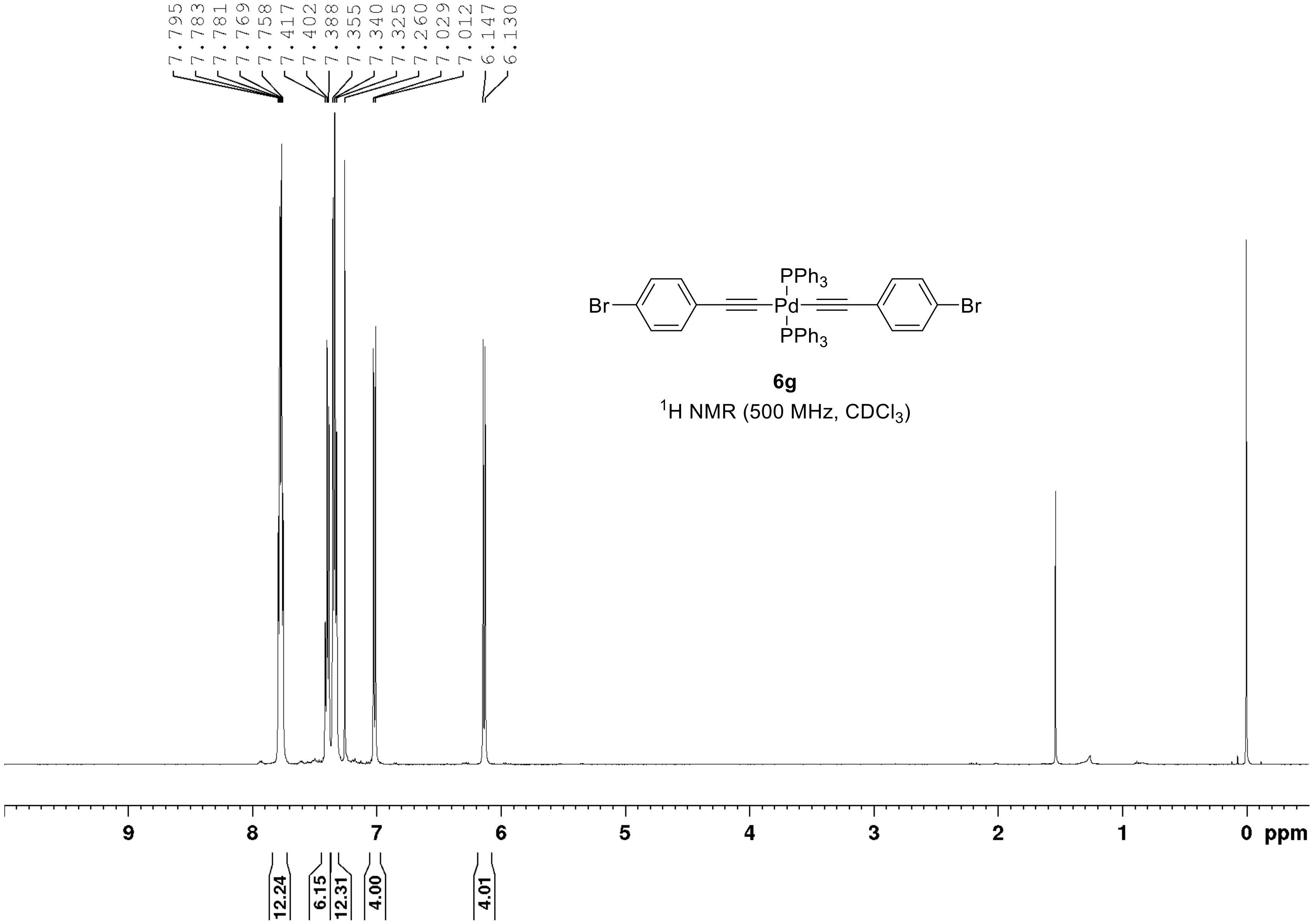
7.795
7.783
7.781
7.769
7.758
7.417
7.402
7.388
7.355
7.340
7.325
7.260
7.029
7.012
6.147
6.130
PPh3
Br
Pd
Br
PPh3
6g
1H NMR (500 MHz, CDCl3)
9
8
7
6
5
4
3
2
1
0 ppm
12.24
6.15
12.31
4.00
4.01

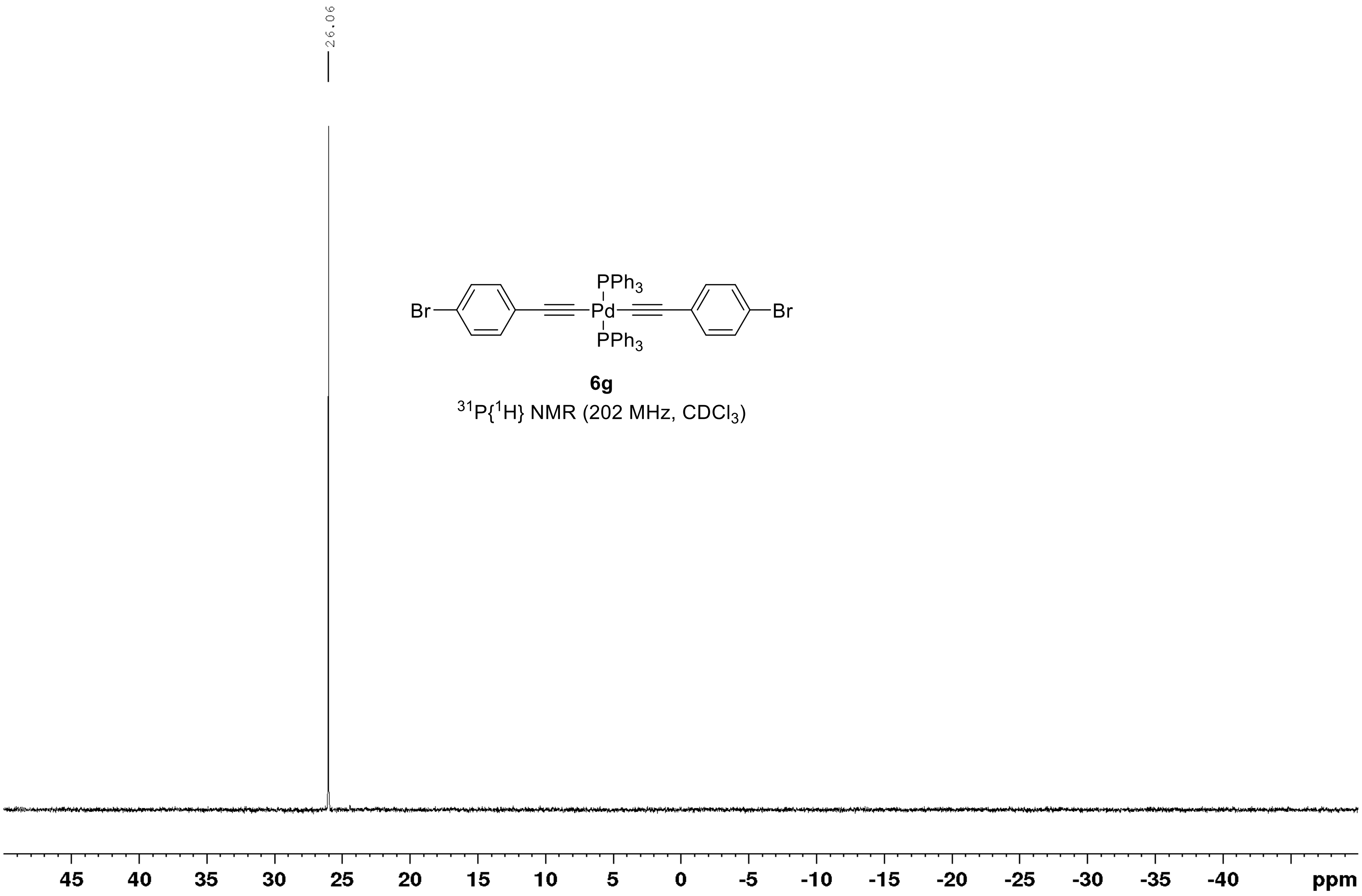
26.06
Br
PPh3
Pd
PPh3
Br
6g
31P{1H} NMR (202 MHz, CDCl3)
45
40
35
30
25
20
15
10
5
0
-5
-10
-15
-20
-25
-30
-35
-40
ppm

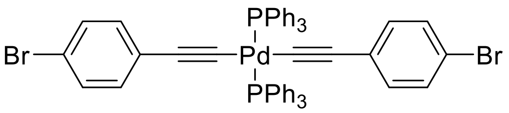

6g

$^{13}C\{^{1}H\}$ NMR (126 MHz, $CDCl_3$)

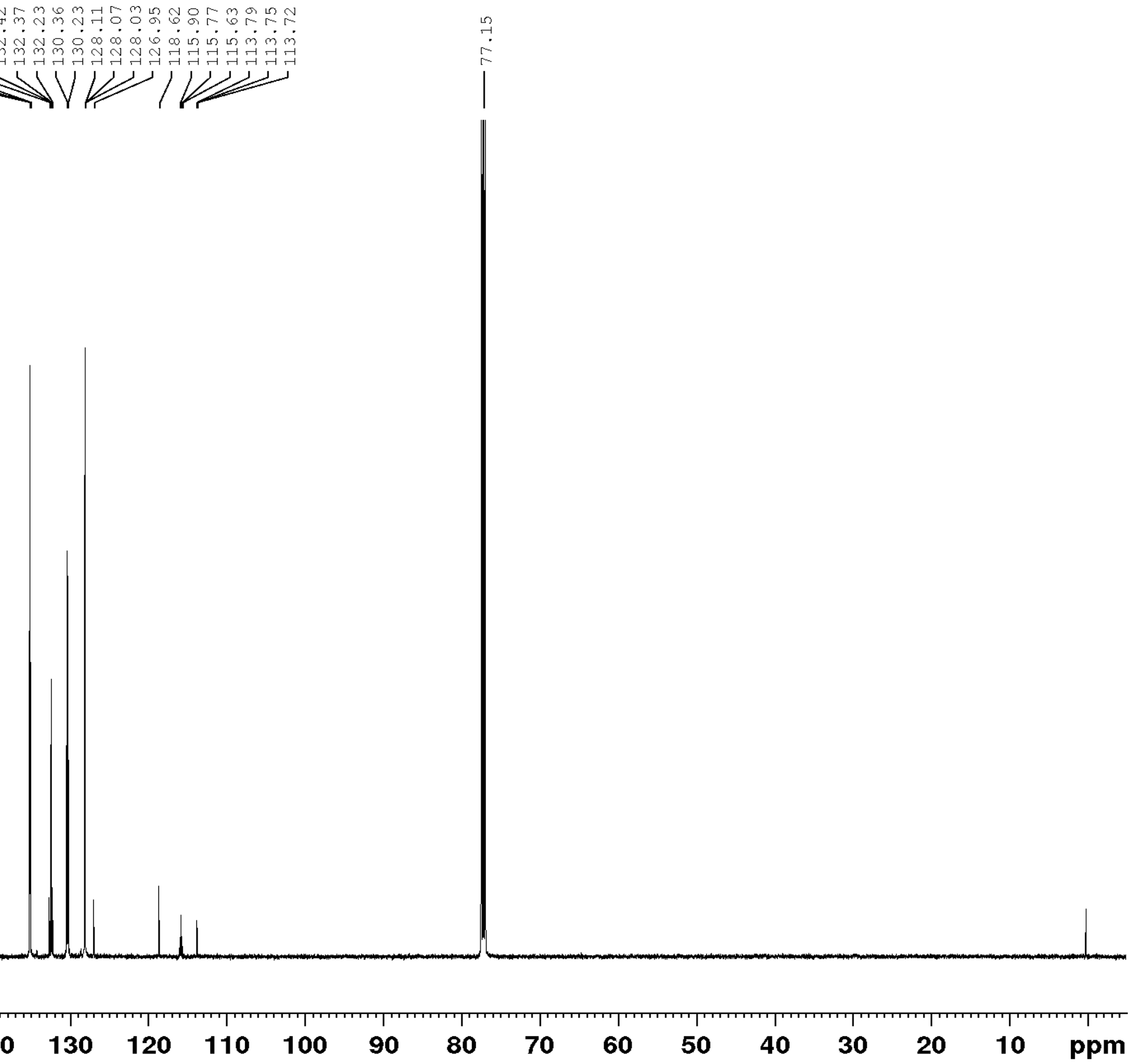

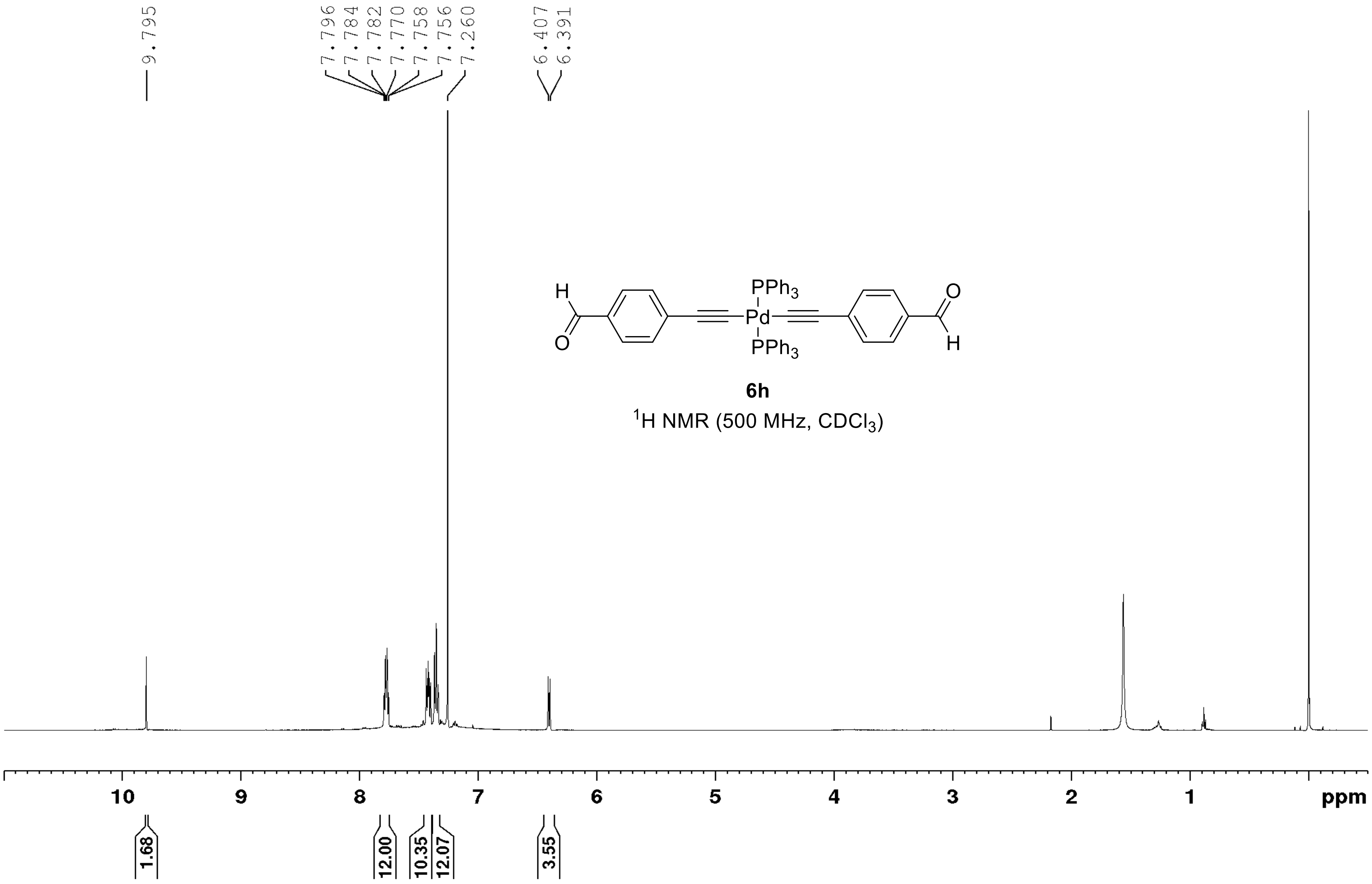

6h

^{1}H NMR (500 MHz, $CDCl_3$)

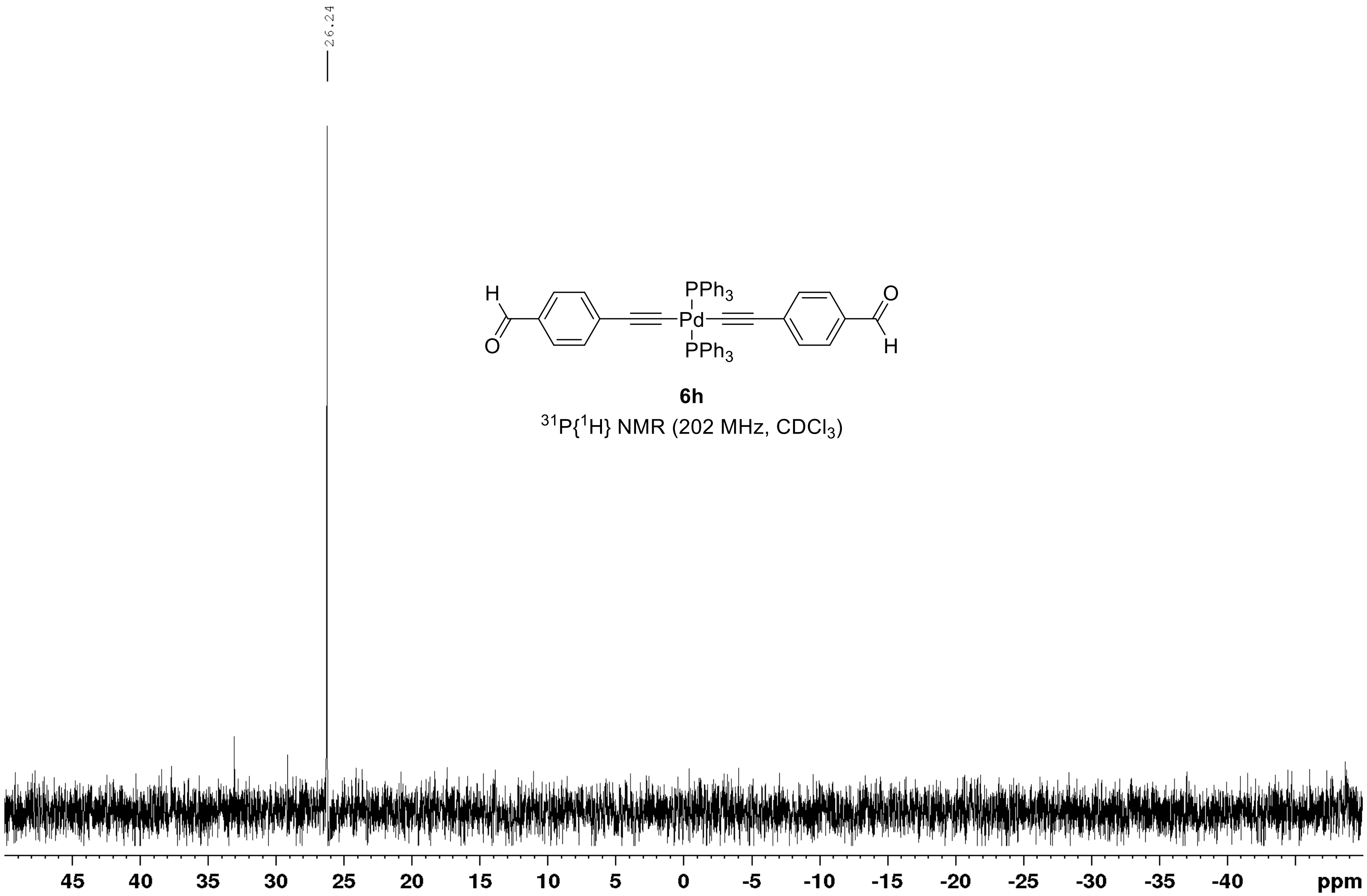

26.24
H
O
PPh3
Pd
PPh3
O
H
6h
$^{31}P\{^{1}H\}$ NMR (202 MHz, $CDCl_3$)
45 40 35 30 25 20 15 10 5 0 -5 -10 -15 -20 -25 -30 -35 -40 ppm

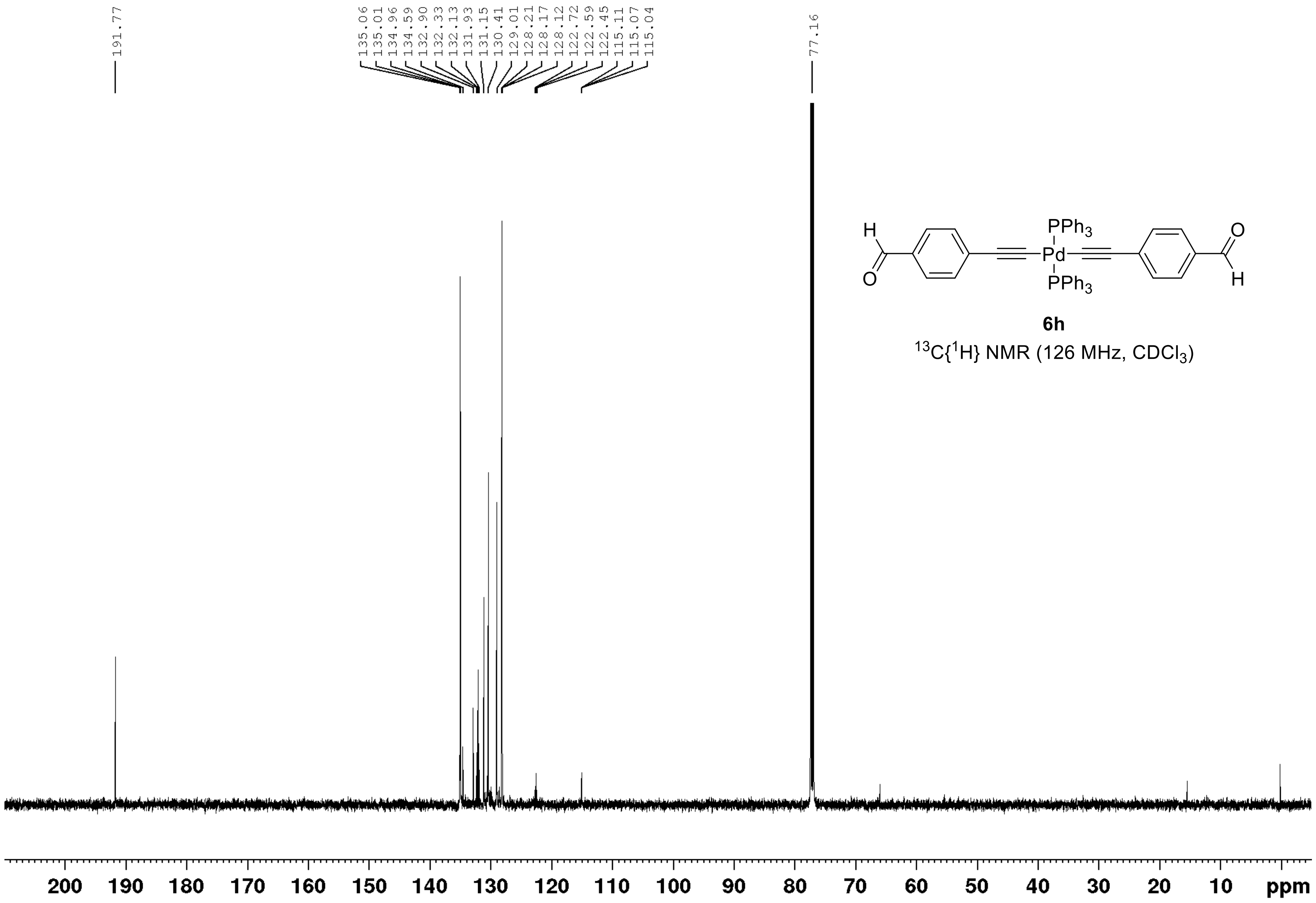
191.77
135.06
135.01
134.96
134.59
132.90
132.33
132.13
131.93
131.15
130.41
129.01
128.21
128.17
128.12
122.72
122.59
122.45
115.11
115.07
115.04
77.16
H
O
PPh3
Pd
PPh3
O
H
6h
13C{1H} NMR (126 MHz, CDCl3)
200 190 180 170 160 150 140 130 120 110 100 90 80 70 60 50 40 30 20 10 ppm

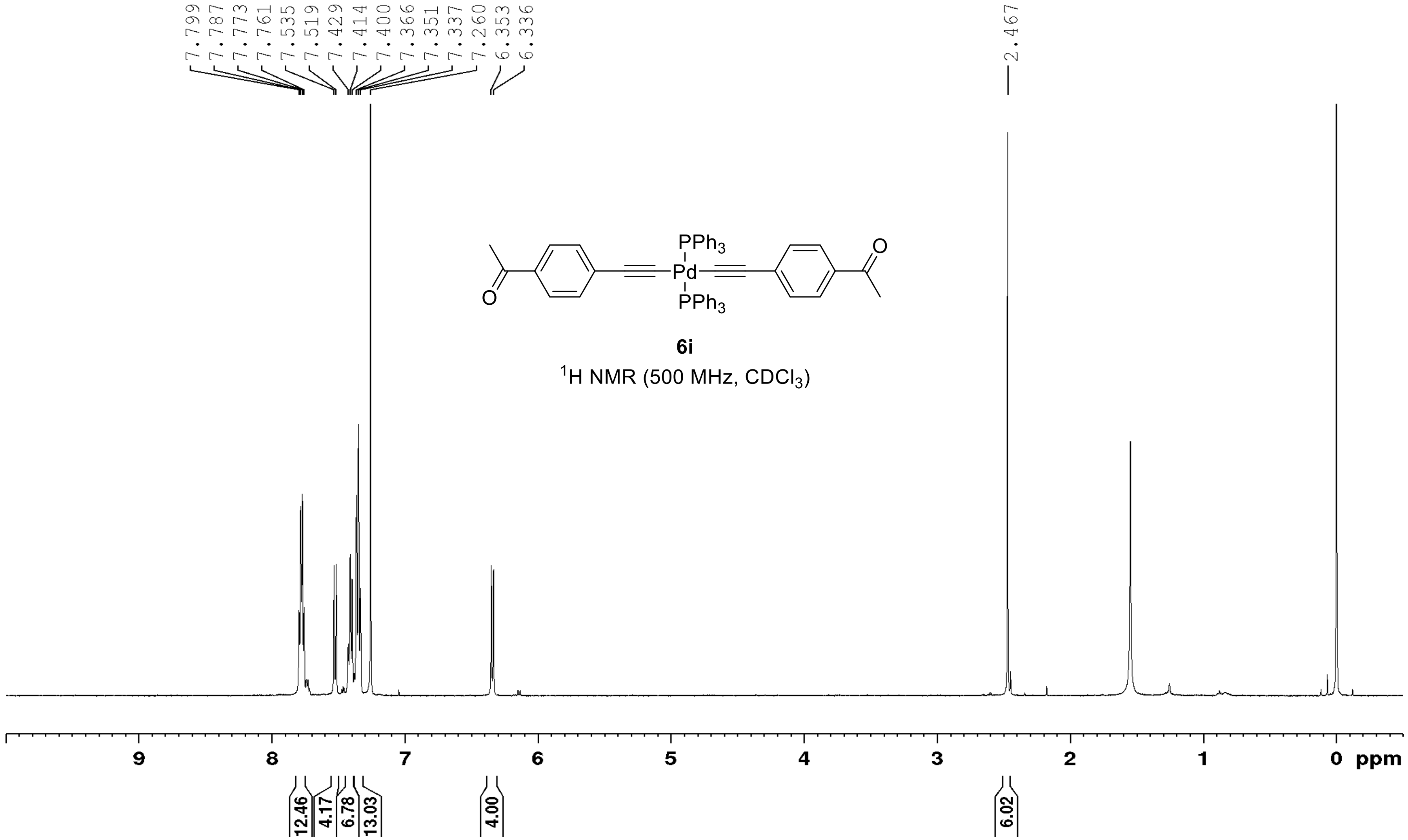
7.799
7.787
7.773
7.761
7.535
7.519
7.429
7.414
7.400
7.366
7.351
7.337
7.260
6.353
6.336
2.467
PPh3
Pd
PPh3
O
O
6i
1H NMR (500 MHz, CDCl3)
9
8
7
6
5
4
3
2
1
0 ppm
12.46
4.17
6.78
13.03
4.00
6.02

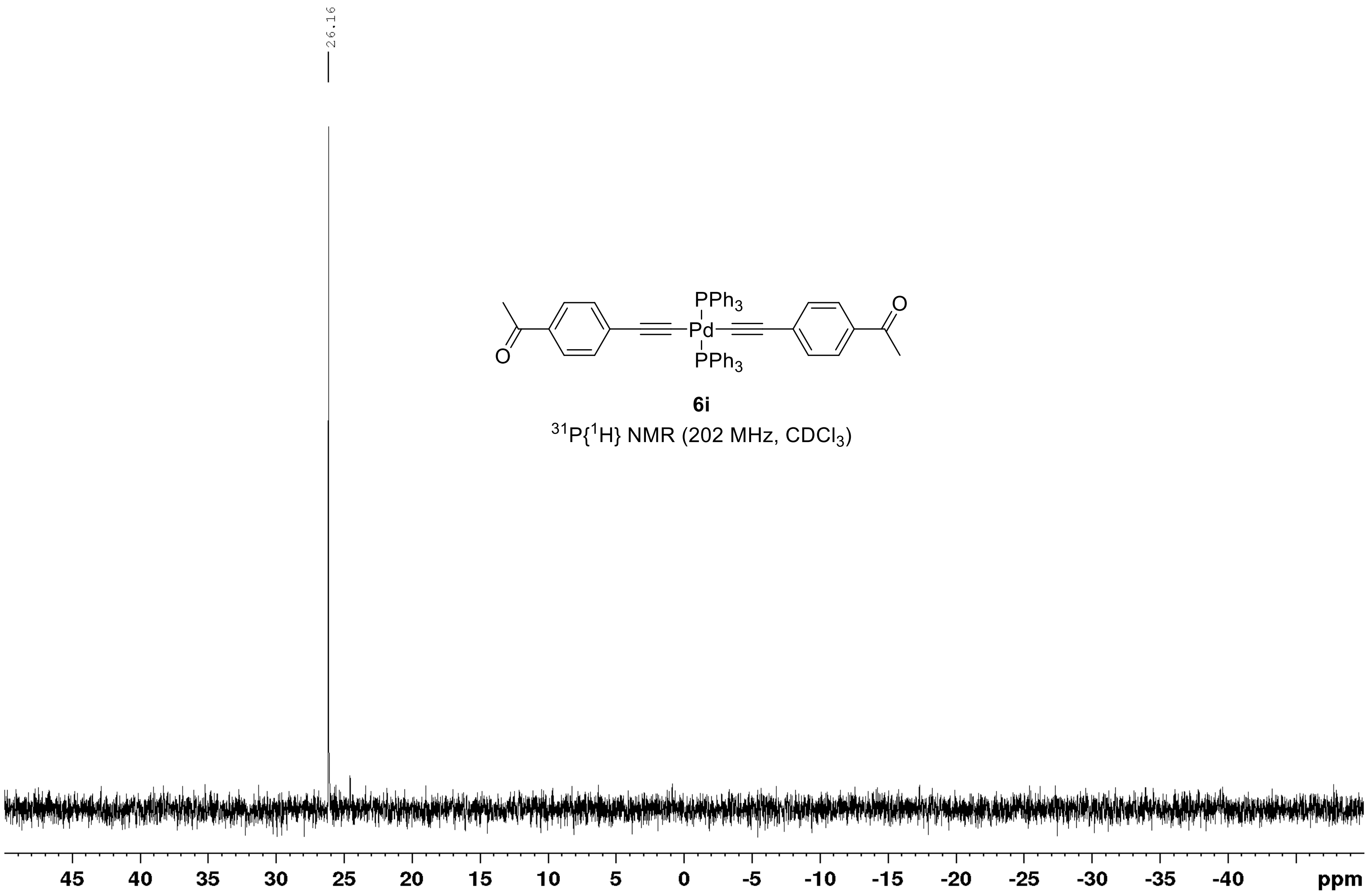
26.16
PPh3
Pd
PPh3
O
O
6i
31P{1H} NMR (202 MHz, CDCl3)
45 40 35 30 25 20 15 10 5 0 -5 -10 -15 -20 -25 -30 -35 -40 ppm

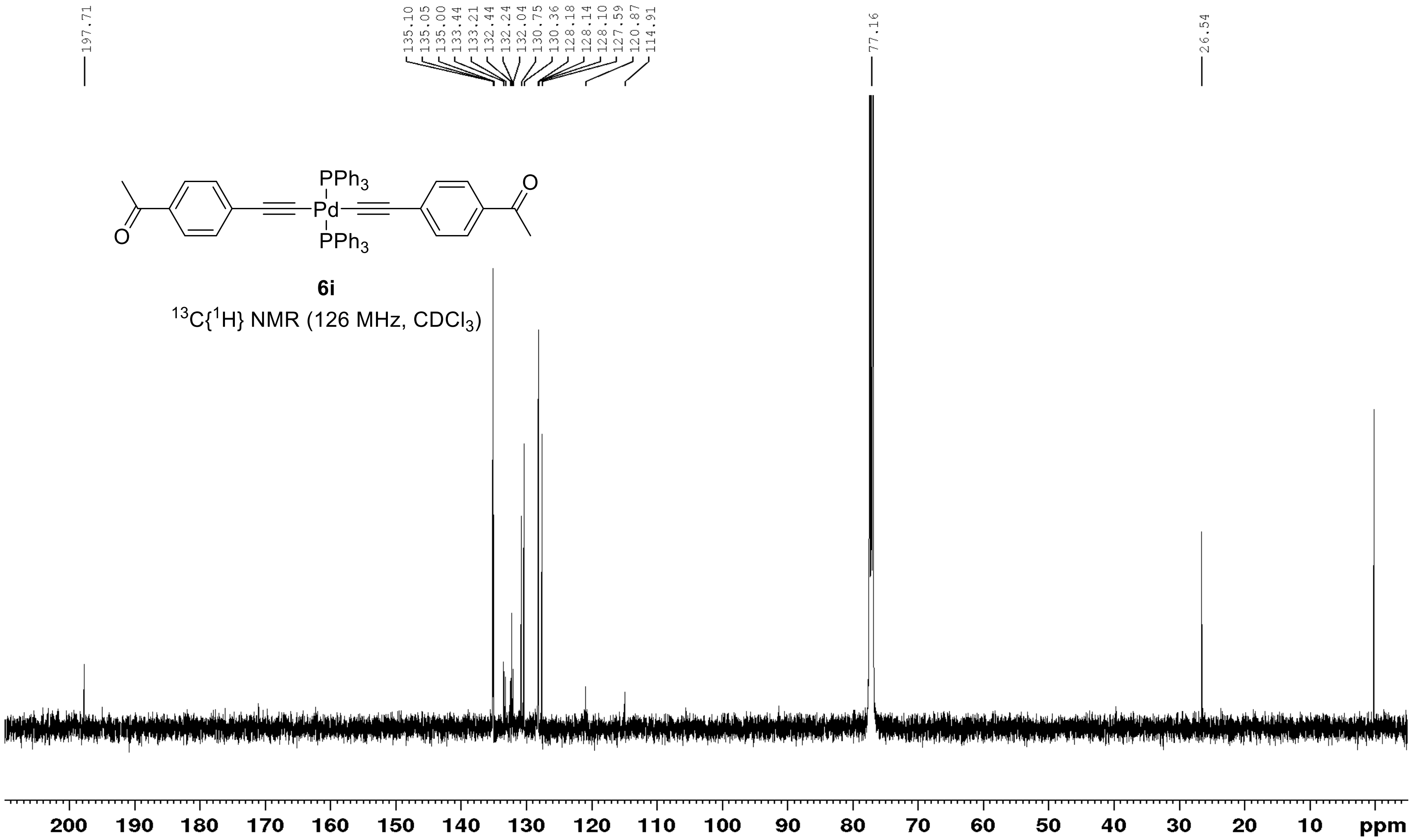

6i

$^{13}C\{^{1}H\}$ NMR (126 MHz, $CDCl_3$)

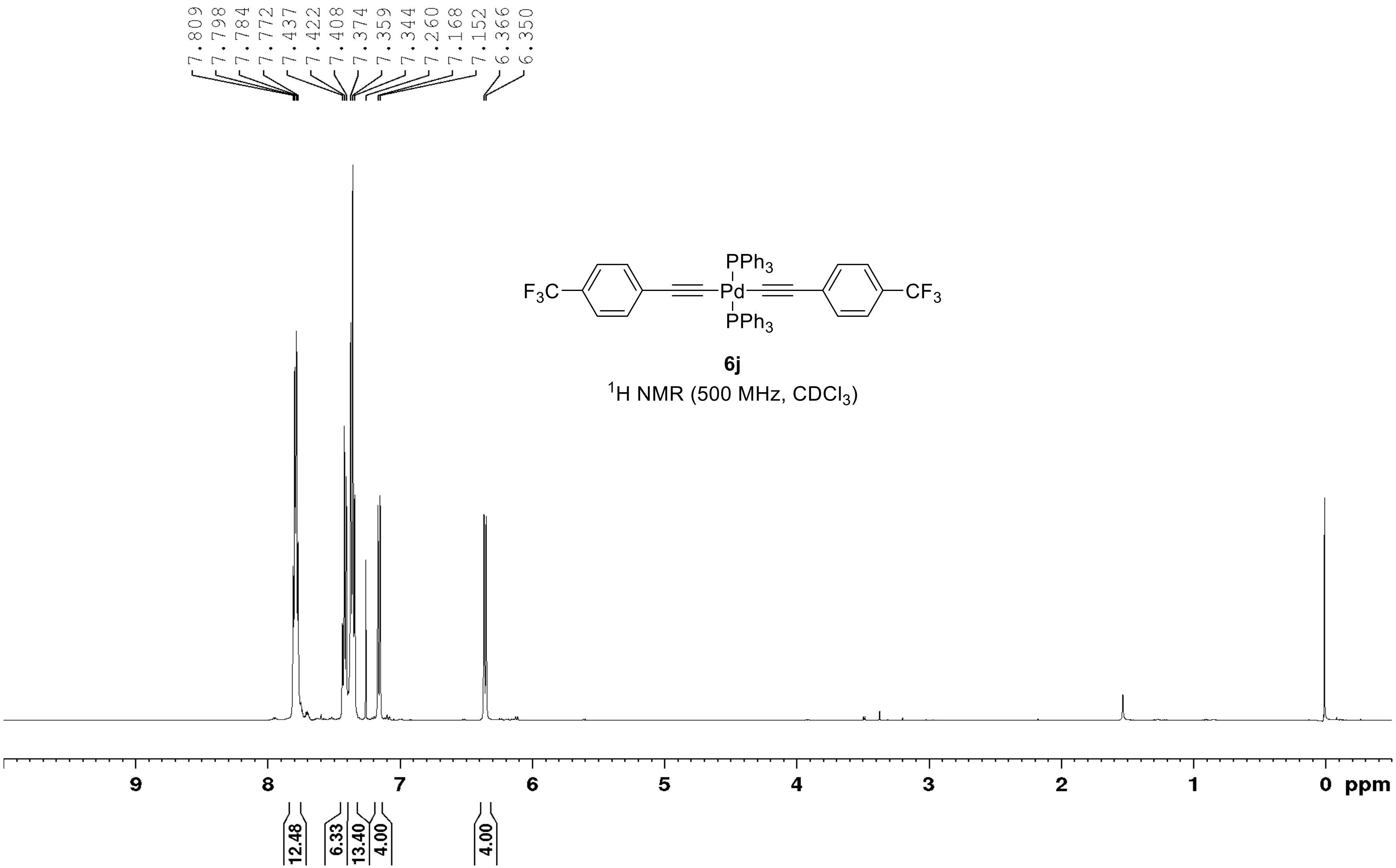
7.809
7.798
7.784
7.772
7.437
7.422
7.408
7.374
7.359
7.344
7.260
7.168
7.152
6.366
6.350
PPh3
F3C
Pd
CF3
PPh3
6j
1H NMR (500 MHz, CDCl3)
9
8
7
6
5
4
3
2
1
0 ppm
12.48
6.33
13.40
4.00
4.00

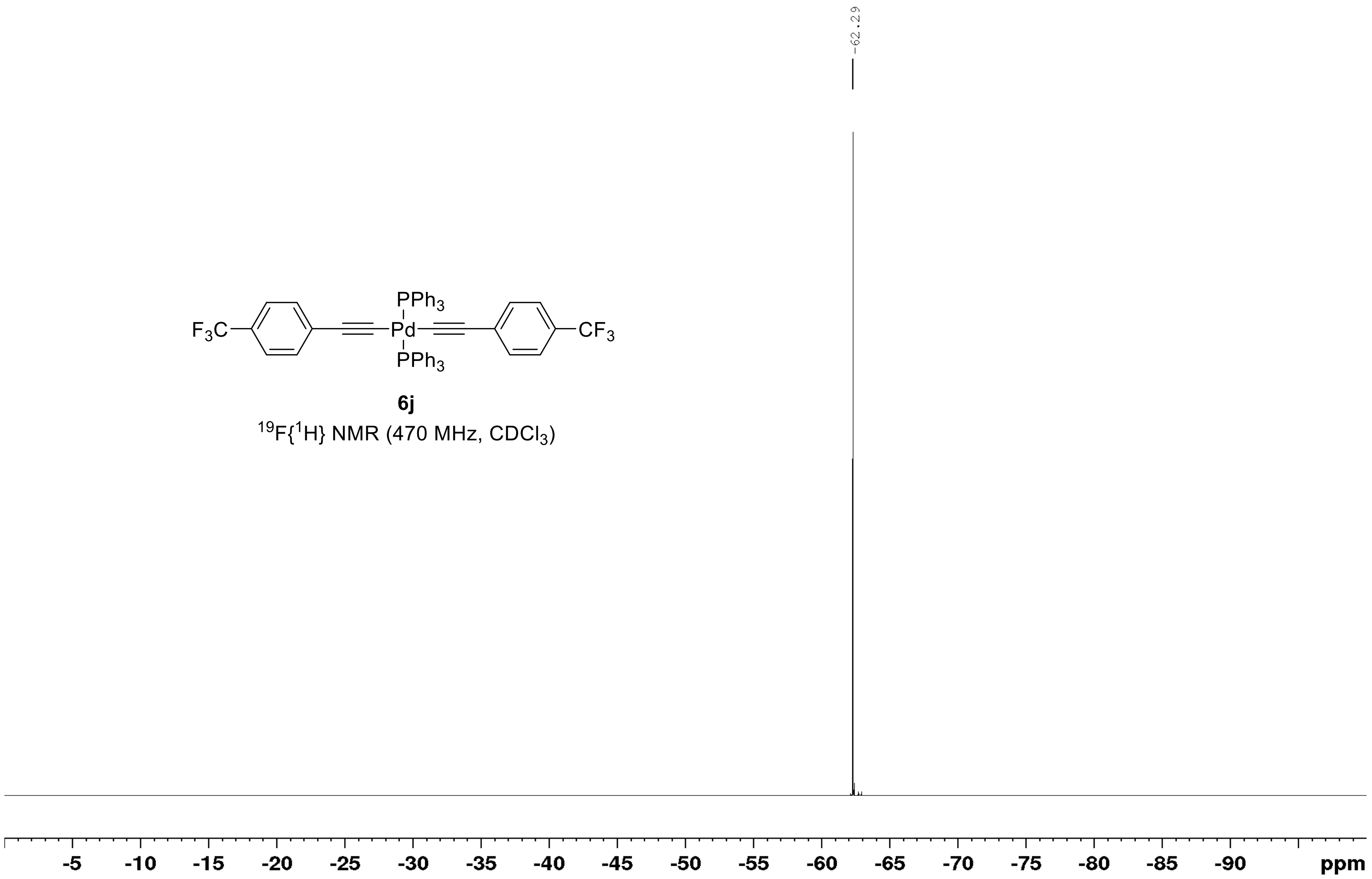
-62.29
F3C
PPh3
Pd
PPh3
CF3
6j
19F{1H} NMR (470 MHz, CDCl3)
-5
-10
-15
-20
-25
-30
-35
-40
-45
-50
-55
-60
-65
-70
-75
-80
-85
-90
ppm

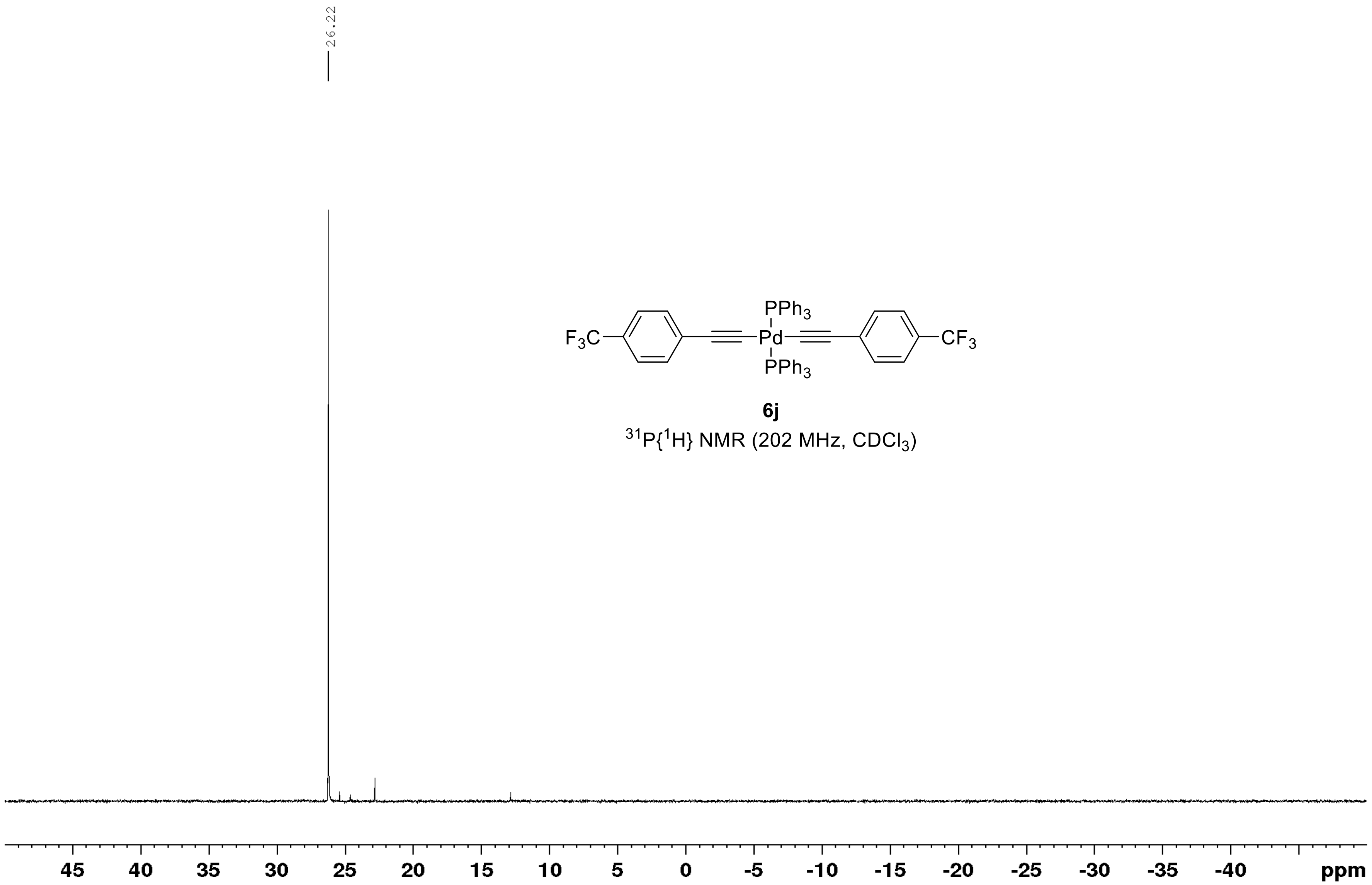

6j

$^{31}P\{^{1}H\}$ NMR (202 MHz, $CDCl_3$)

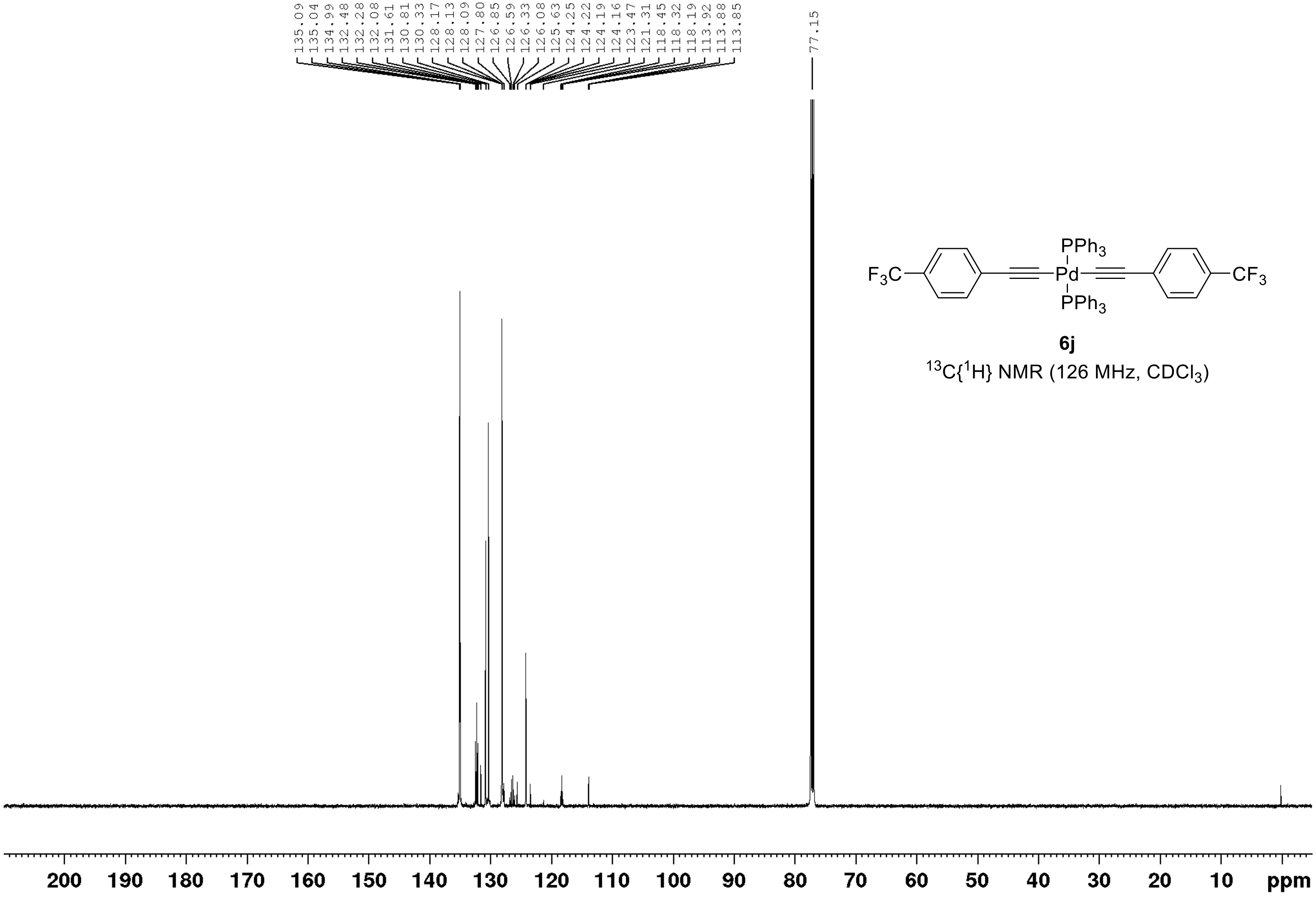

6j

$^{13}C\{^{1}H\}$ NMR (126 MHz, $CDCl_3$)

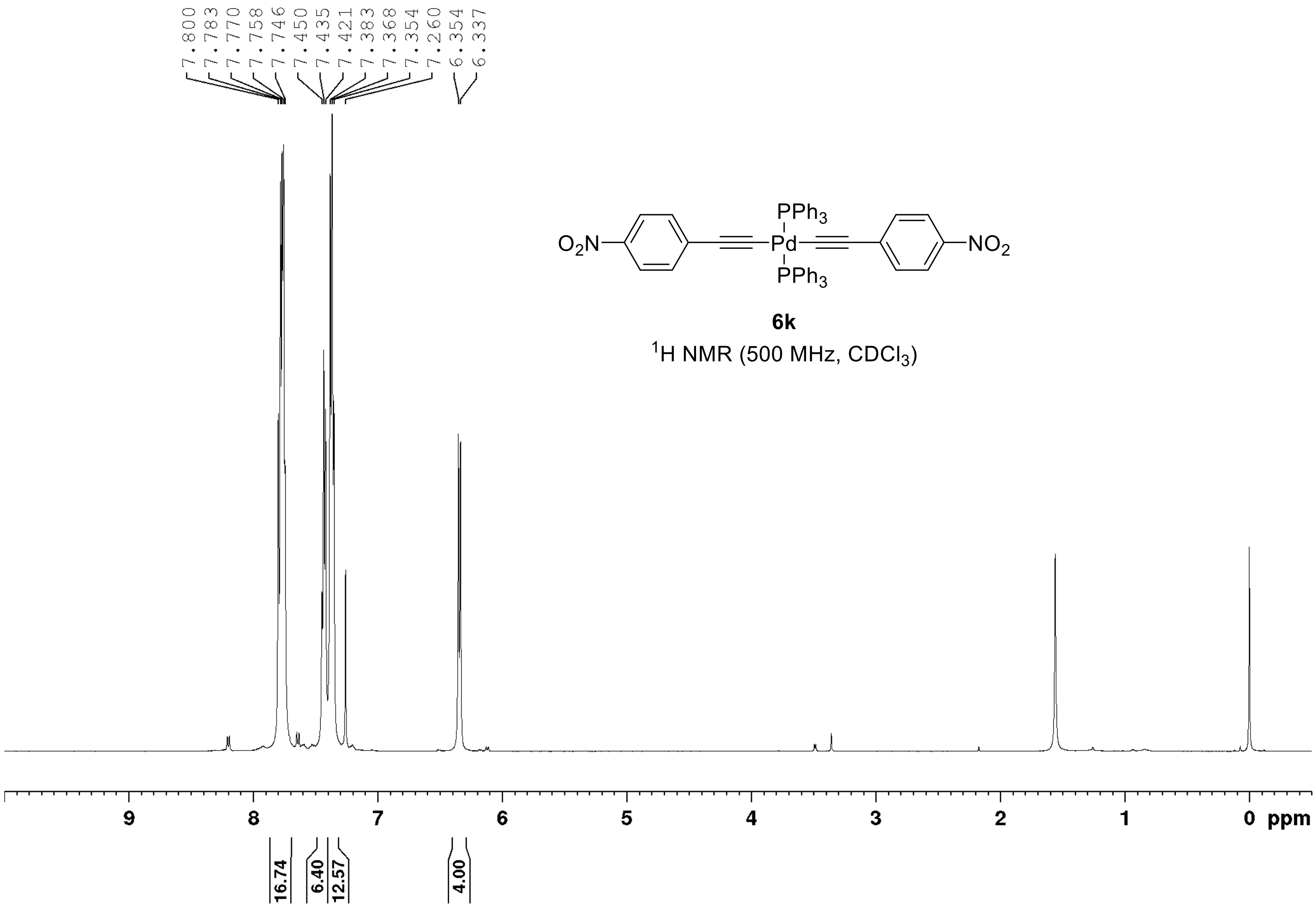
7.800
7.783
7.770
7.758
7.746
7.450
7.435
7.421
7.383
7.368
7.354
7.260
6.354
6.337
PPh3
O2N
Pd
NO2
PPh3
6k
1H NMR (500 MHz, CDCl3)
9
8
7
6
5
4
3
2
1
0 ppm
16.74
6.40
12.57
4.00

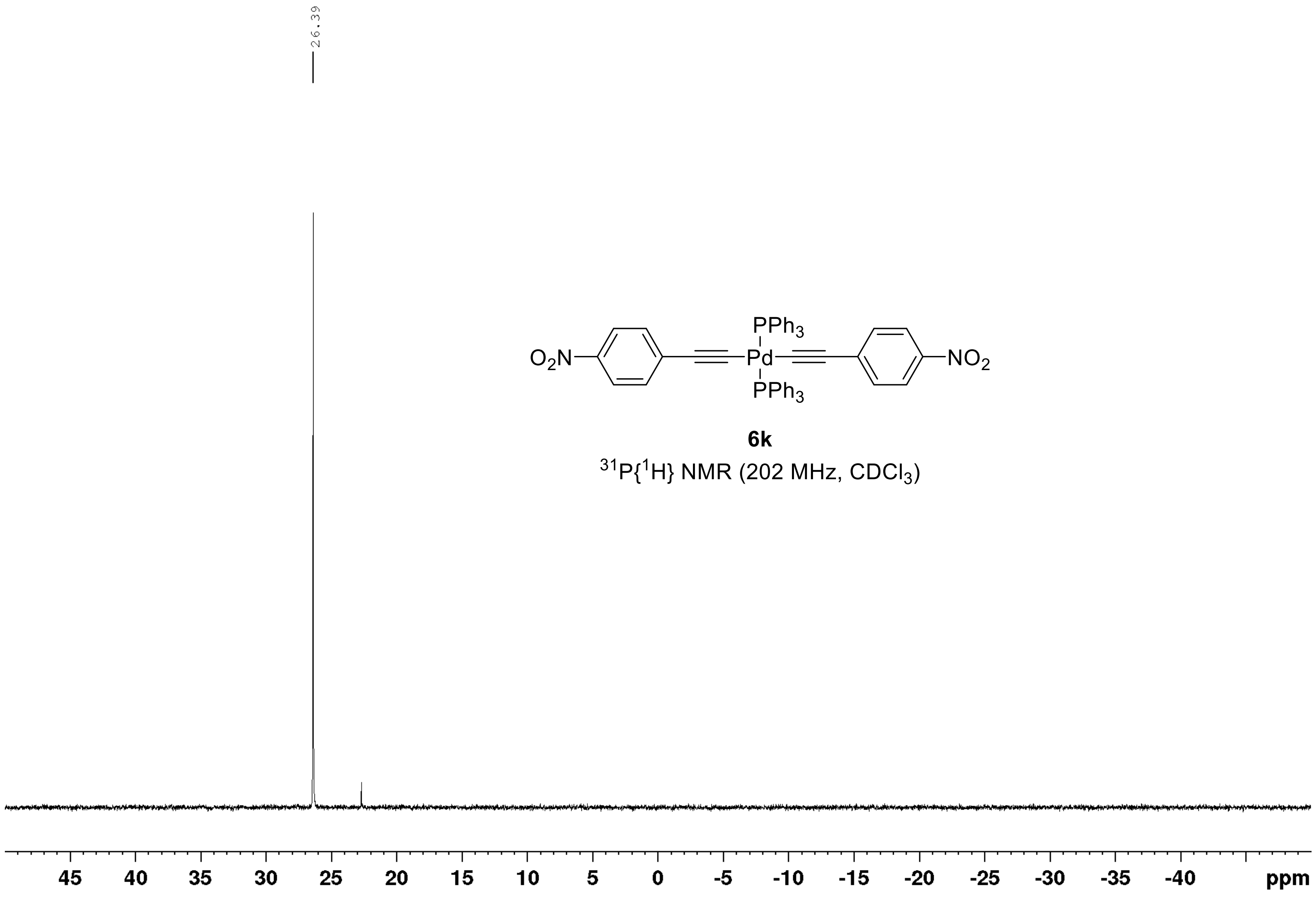

6k

$^{31}P\{^1H\}$ NMR (202 MHz, $CDCl_3$)

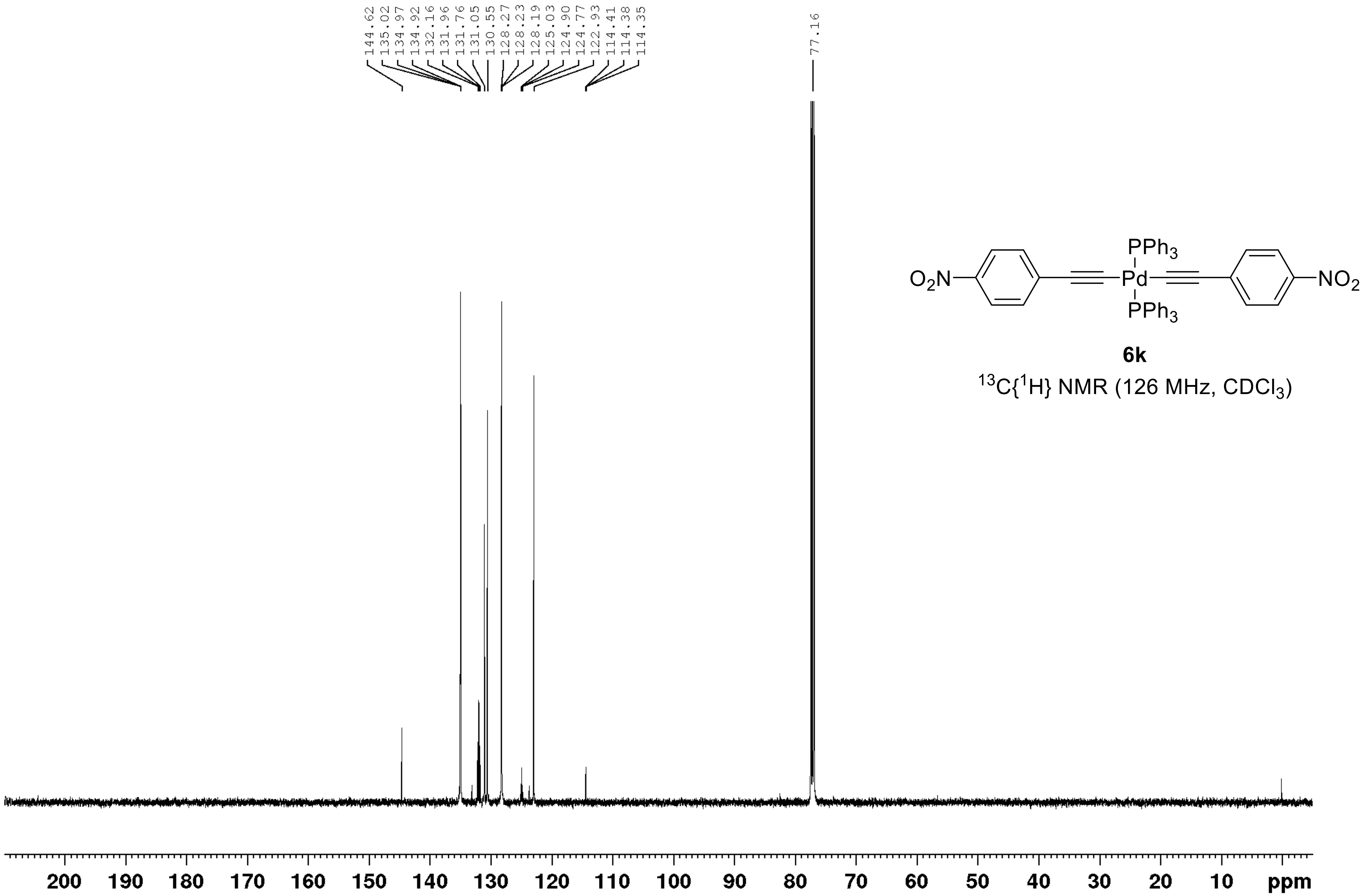

6k

$^{13}C\{^1H\}$ NMR (126 MHz, $CDCl_3$)

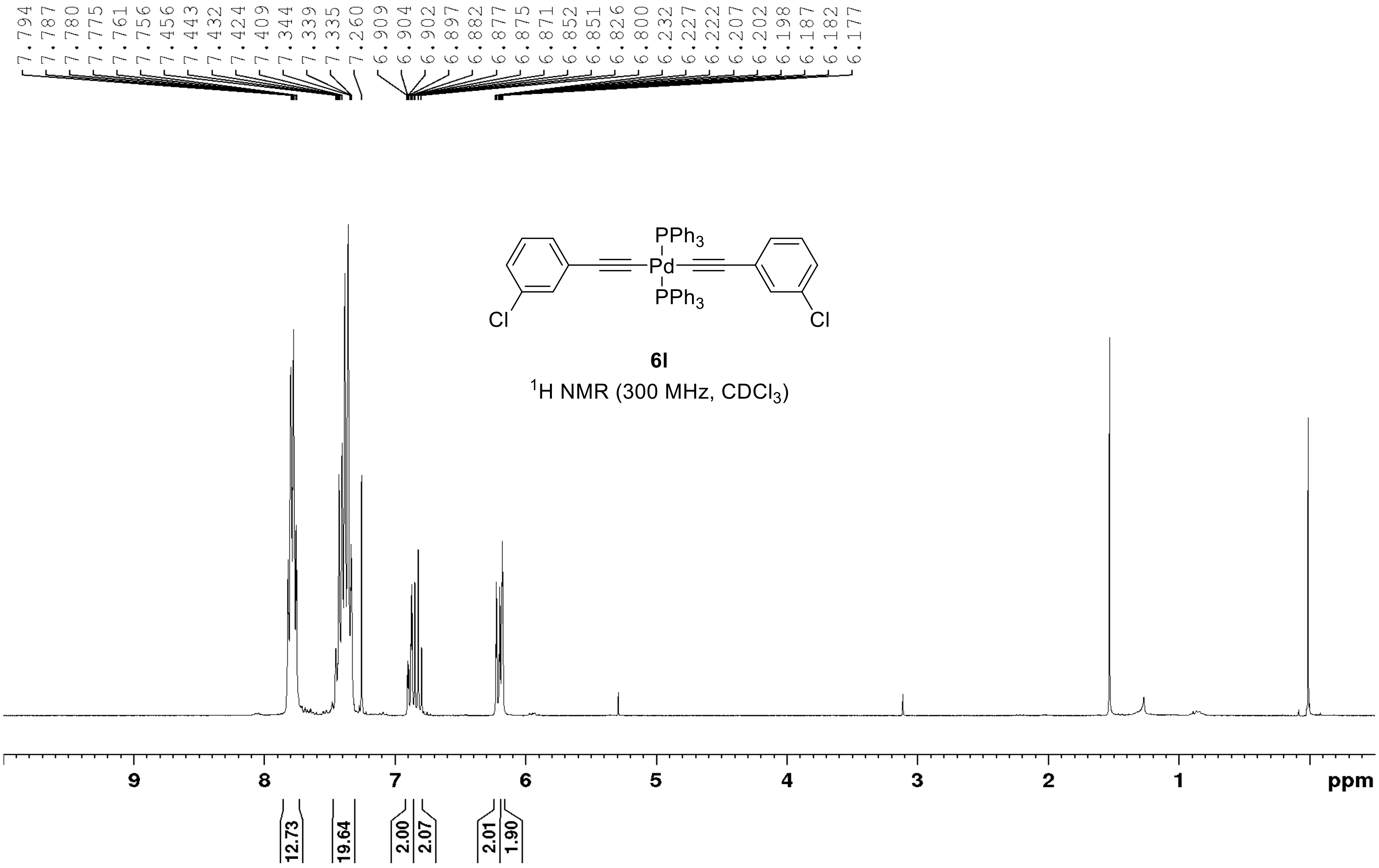
7.794 7.787 7.780 7.775 7.761 7.756 7.456 7.443 7.432 7.424 7.409 7.344 7.339 7.335 7.260 6.909 6.904 6.902 6.897 6.882 6.877 6.875 6.871 6.852 6.851 6.826 6.800 6.232 6.227 6.222 6.207 6.202 6.198 6.187 6.182 6.177
PPh3
Pd
PPh3
Cl
Cl
6l
1H NMR (300 MHz, CDCl3)
9
8
7
6
5
4
3
2
1
ppm
12.73
19.64
2.00
2.07
2.01
1.90

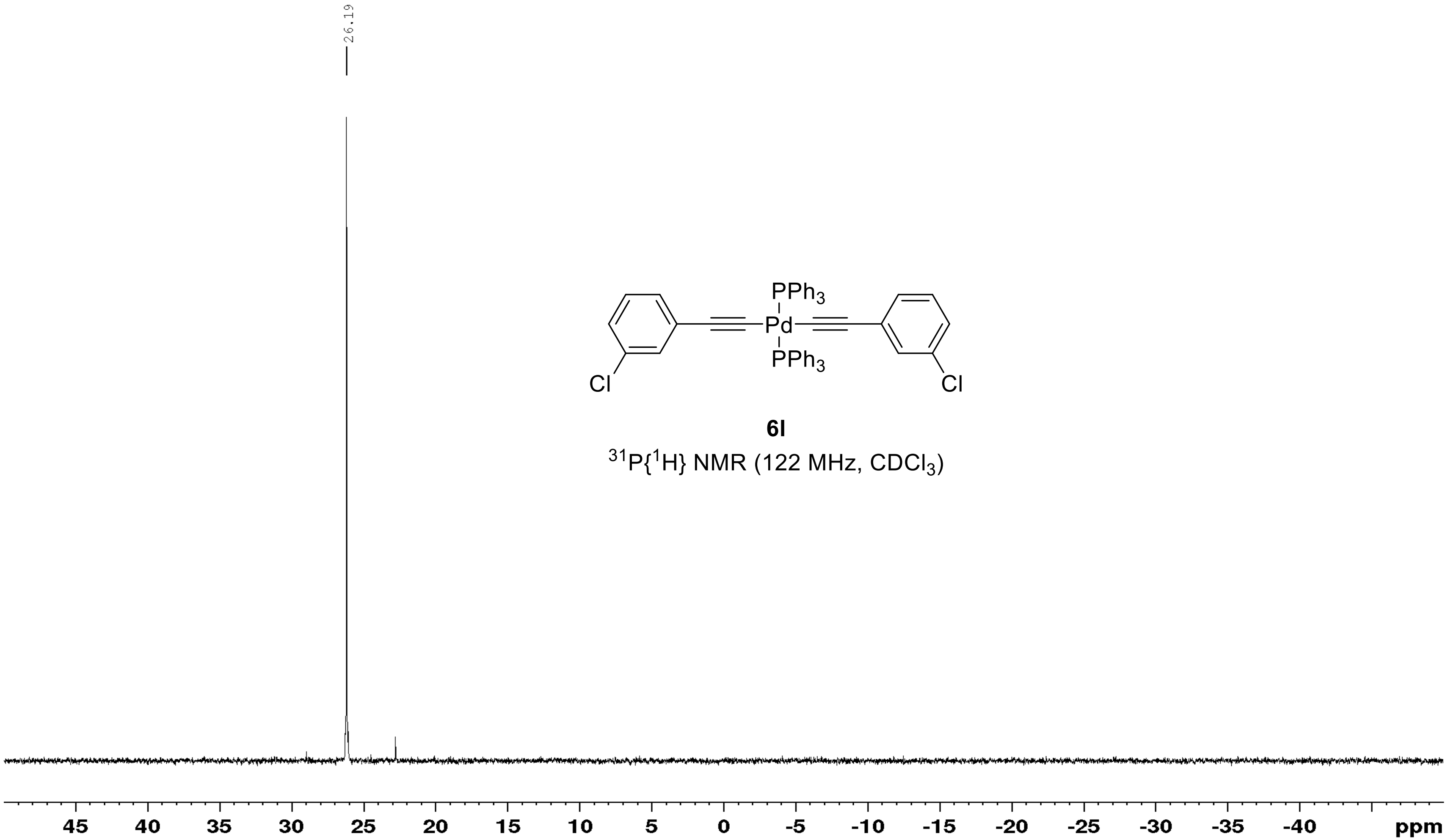

6l

$^{31}P\{^1H\}$ NMR (122 MHz, $CDCl_3$)

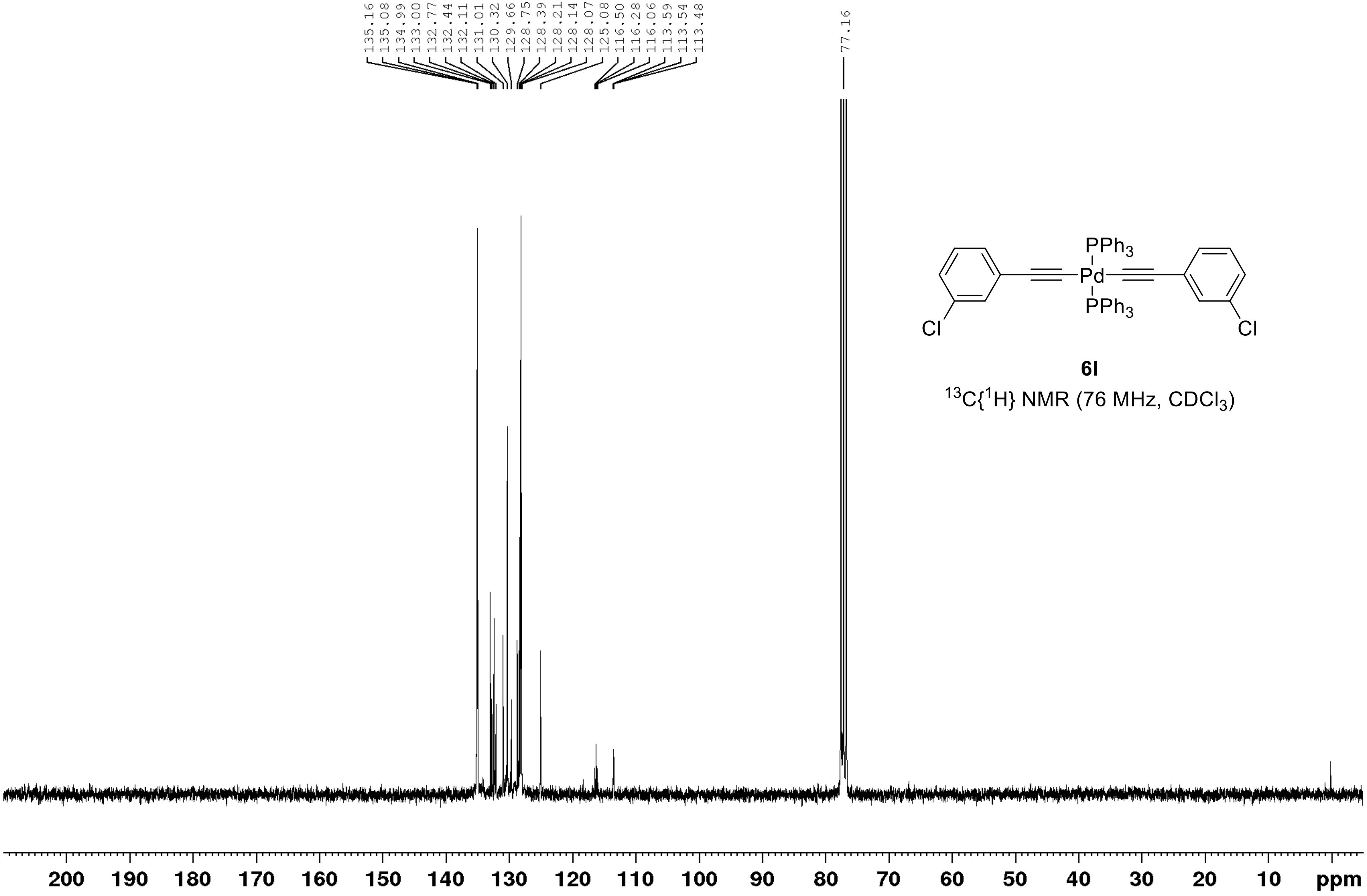
135.16
135.08
134.99
133.00
132.77
132.44
132.11
131.01
130.32
129.66
128.75
128.39
128.21
128.14
128.07
125.08
116.50
116.28
116.06
113.59
113.54
113.48
77.16
PPh3
Pd
PPh3
Cl
Cl
6l
13C{1H} NMR (76 MHz, CDCl3)
200
190
180
170
160
150
140
130
120
110
100
90
80
70
60
50
40
30
20
10
ppm

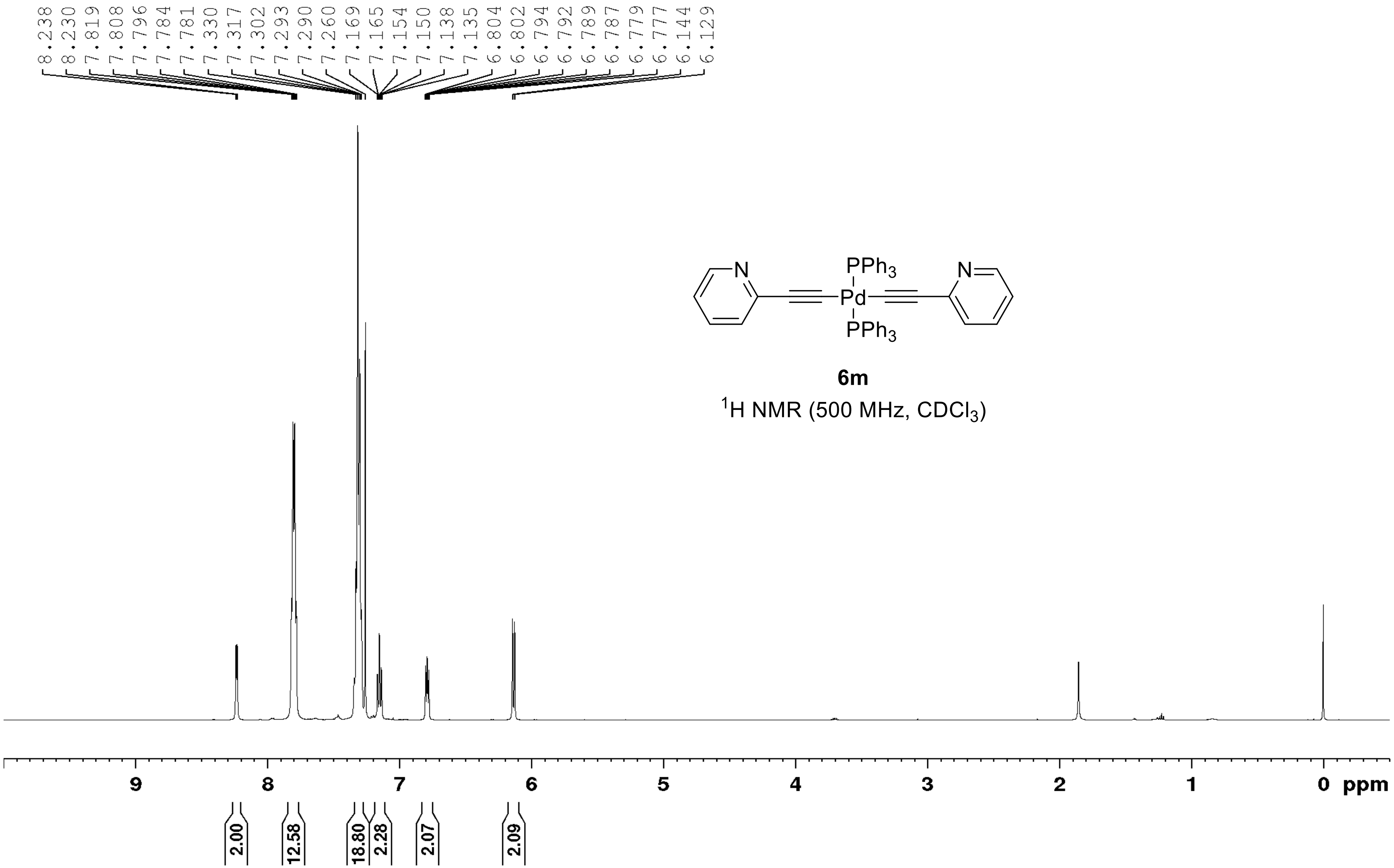

6m

^{1}H NMR (500 MHz, $CDCl_3$)

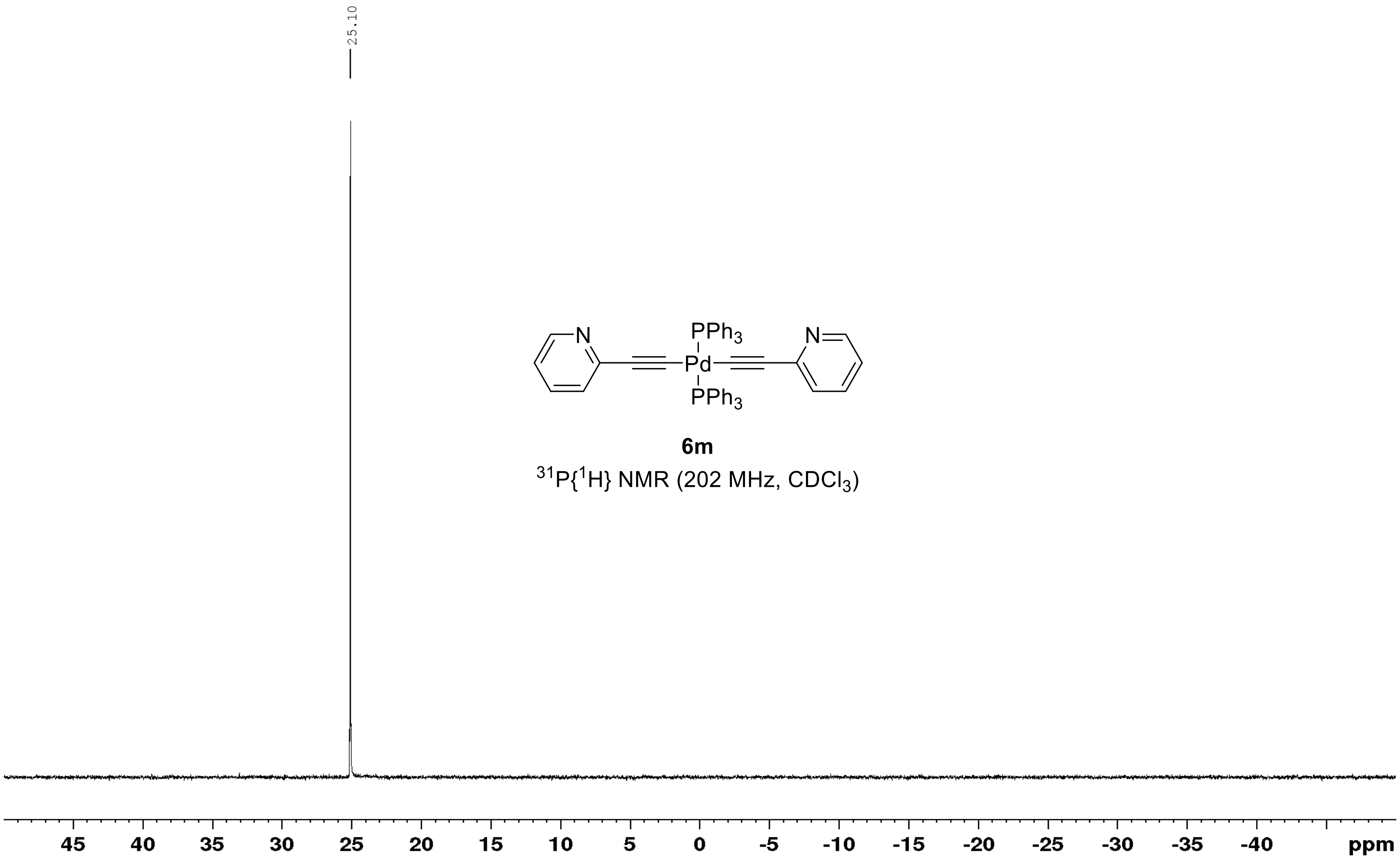

6m

$^{31}P\{^1H\}$ NMR (202 MHz, $CDCl_3$)

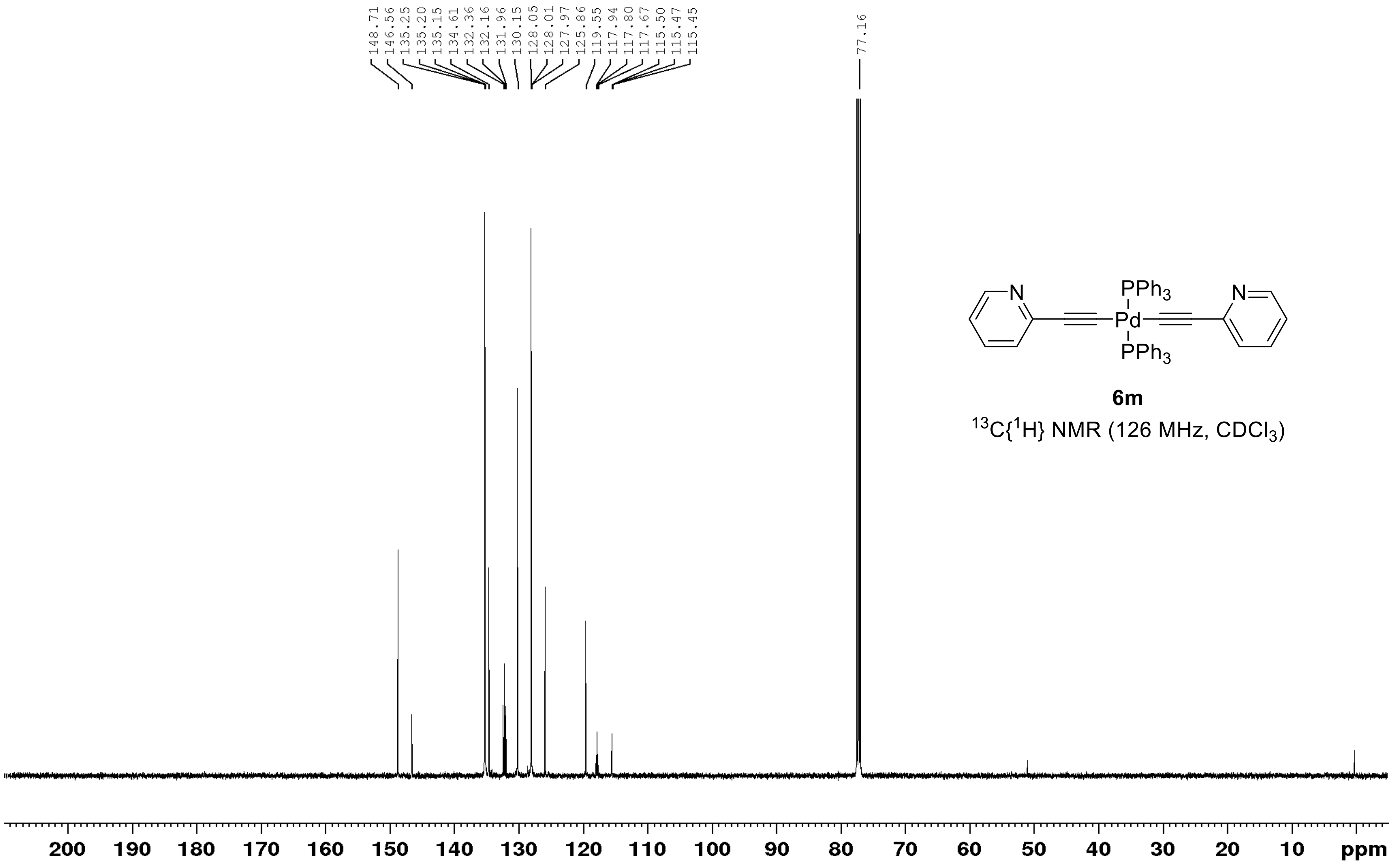

6m

$^{13}C\{^{1}H\}$ NMR (126 MHz, $CDCl_3$)

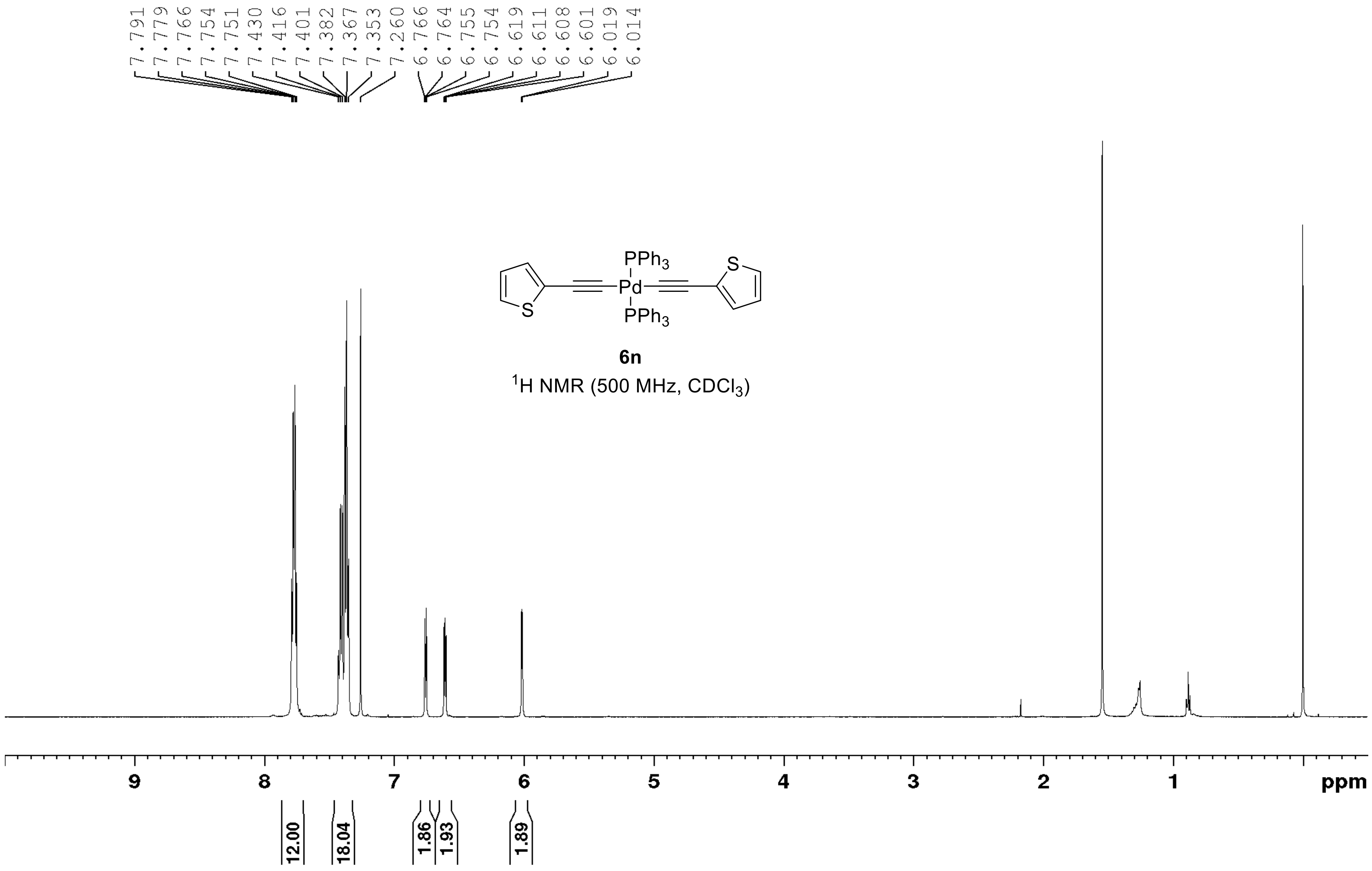
7.791
7.779
7.766
7.754
7.751
7.430
7.416
7.401
7.382
7.367
7.353
7.260
6.766
6.764
6.755
6.754
6.619
6.611
6.608
6.601
6.019
6.014
PPh3
Pd
PPh3
S
S
6n
1H NMR (500 MHz, CDCl3)
9
8
7
6
5
4
3
2
1
ppm
12.00
18.04
1.86
1.93
1.89

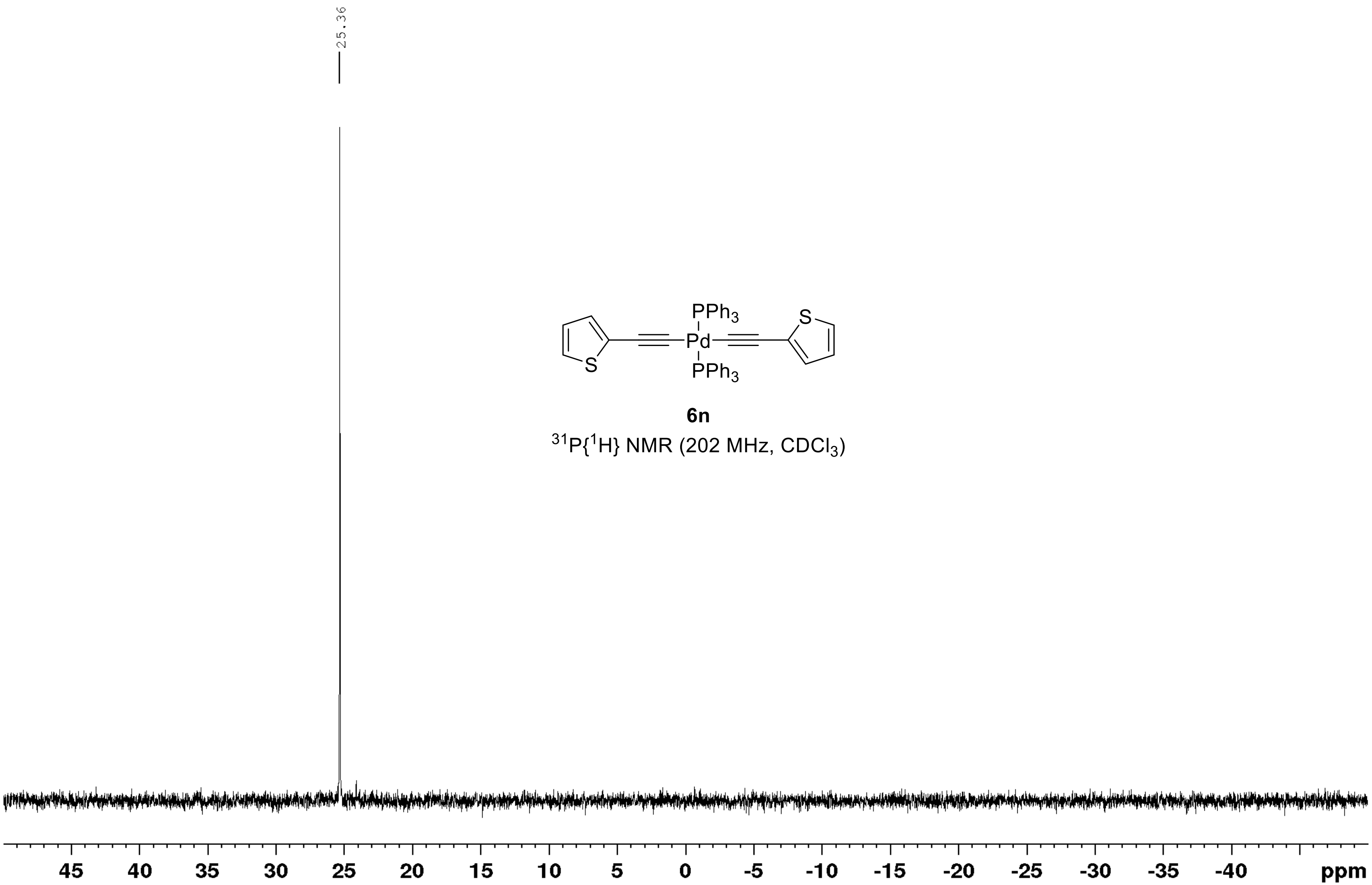

6n

$^{31}P\{^1H\}$ NMR (202 MHz, $CDCl_3$)

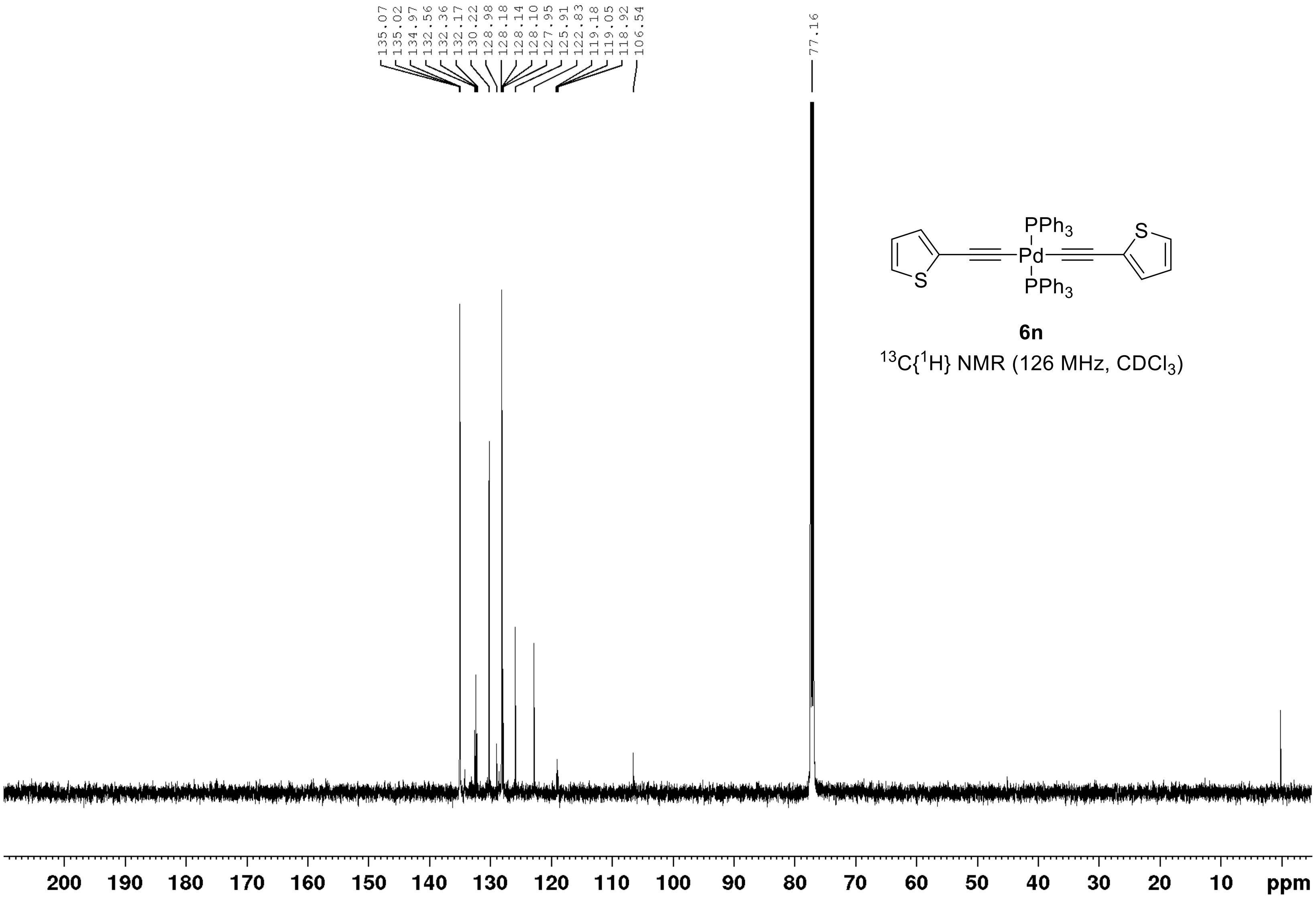
135.07
135.02
134.97
132.56
132.36
132.17
130.22
128.98
128.18
128.14
128.10
127.95
125.91
122.83
119.18
119.05
118.92
106.54
77.16
PPh3
Pd
PPh3
S
S
6n
13C{1H} NMR (126 MHz, CDCl3)
200
190
180
170
160
150
140
130
120
110
100
90
80
70
60
50
40
30
20
10
ppm

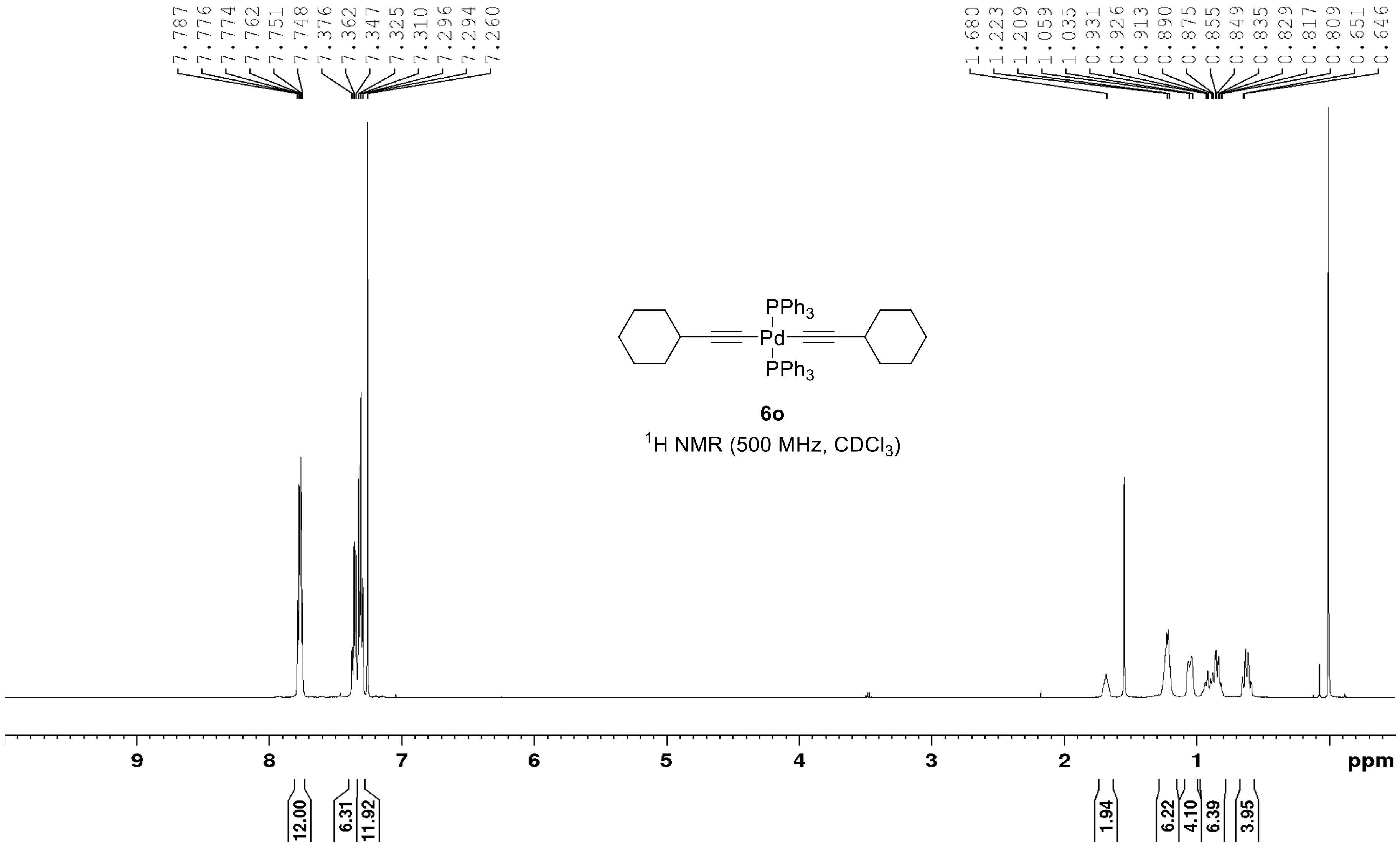
7.787
7.776
7.774
7.762
7.751
7.748
7.376
7.362
7.347
7.325
7.310
7.296
7.294
7.260
1.680
1.223
1.209
1.059
1.035
0.931
0.926
0.913
0.890
0.875
0.855
0.849
0.835
0.829
0.817
0.809
0.651
0.646
PPh3
Pd
PPh3
6o
1H NMR (500 MHz, CDCl3)
9
8
7
6
5
4
3
2
1
ppm
12.00
6.31
11.92
1.94
6.22
4.10
6.39
3.95

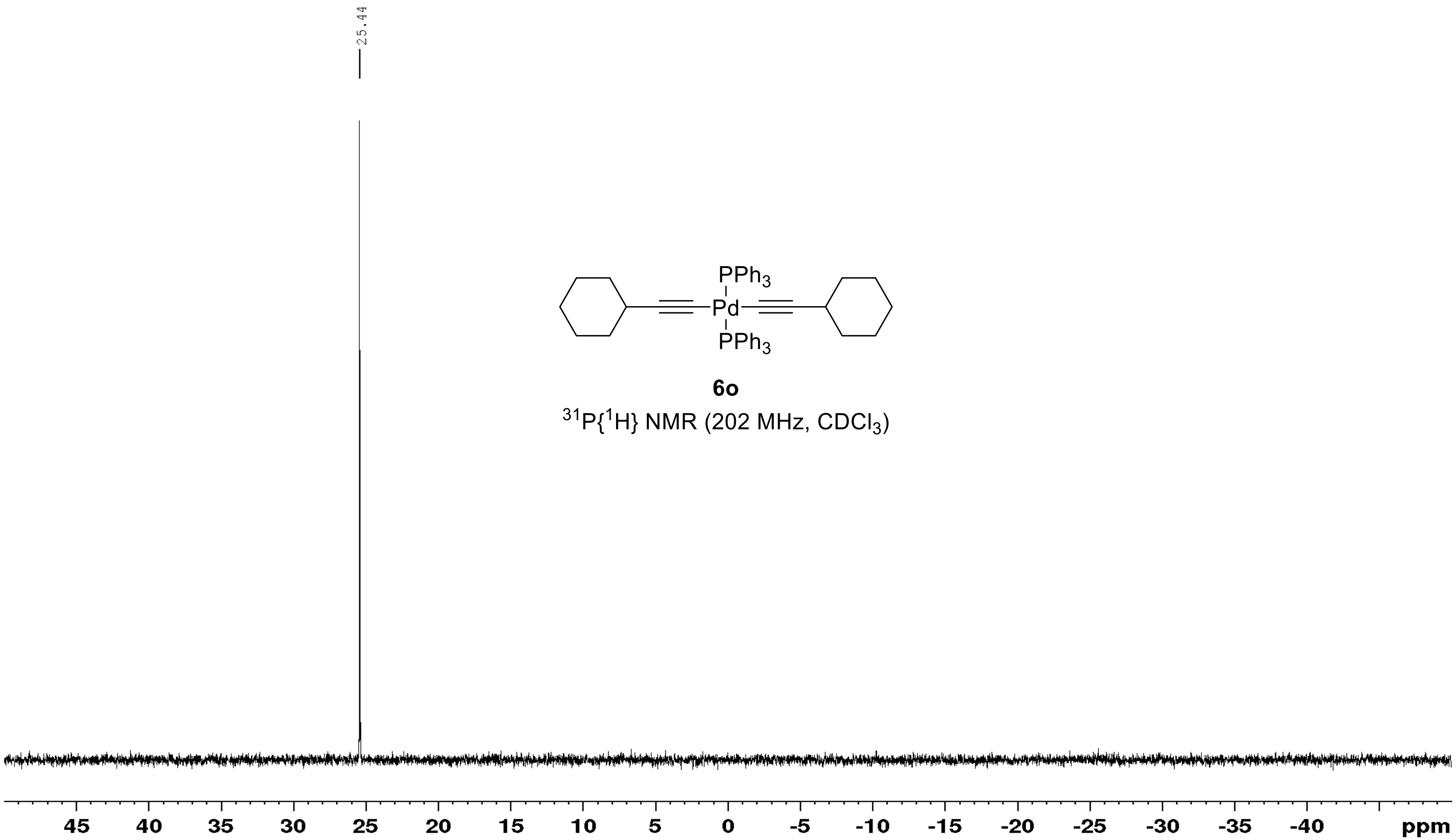
25.44
PPh3
Pd
PPh3
6o
$^{31}P\{^{1}H\}$ NMR (202 MHz, $CDCl_3$)
45 40 35 30 25 20 15 10 5 0 -5 -10 -15 -20 -25 -30 -35 -40 ppm

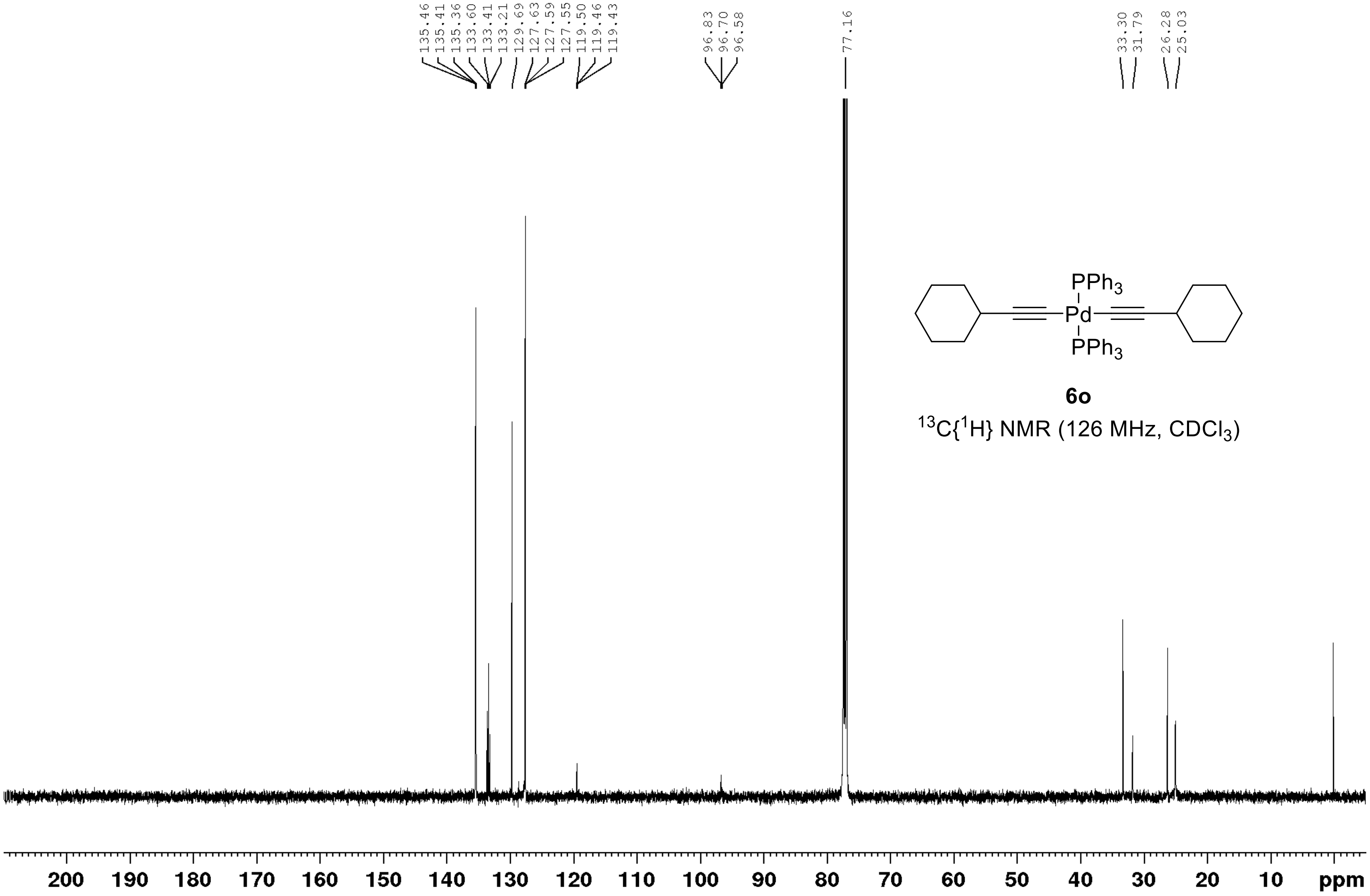
135.46
135.41
135.36
133.60
133.41
133.21
129.69
127.63
127.59
127.55
119.50
119.46
119.43
96.83
96.70
96.58
77.16
33.30
31.79
26.28
25.03
PPh3
Pd
PPh3
6o
13C{1H} NMR (126 MHz, CDCl3)
200
190
180
170
160
150
140
130
120
110
100
90
80
70
60
50
40
30
20
10
ppm

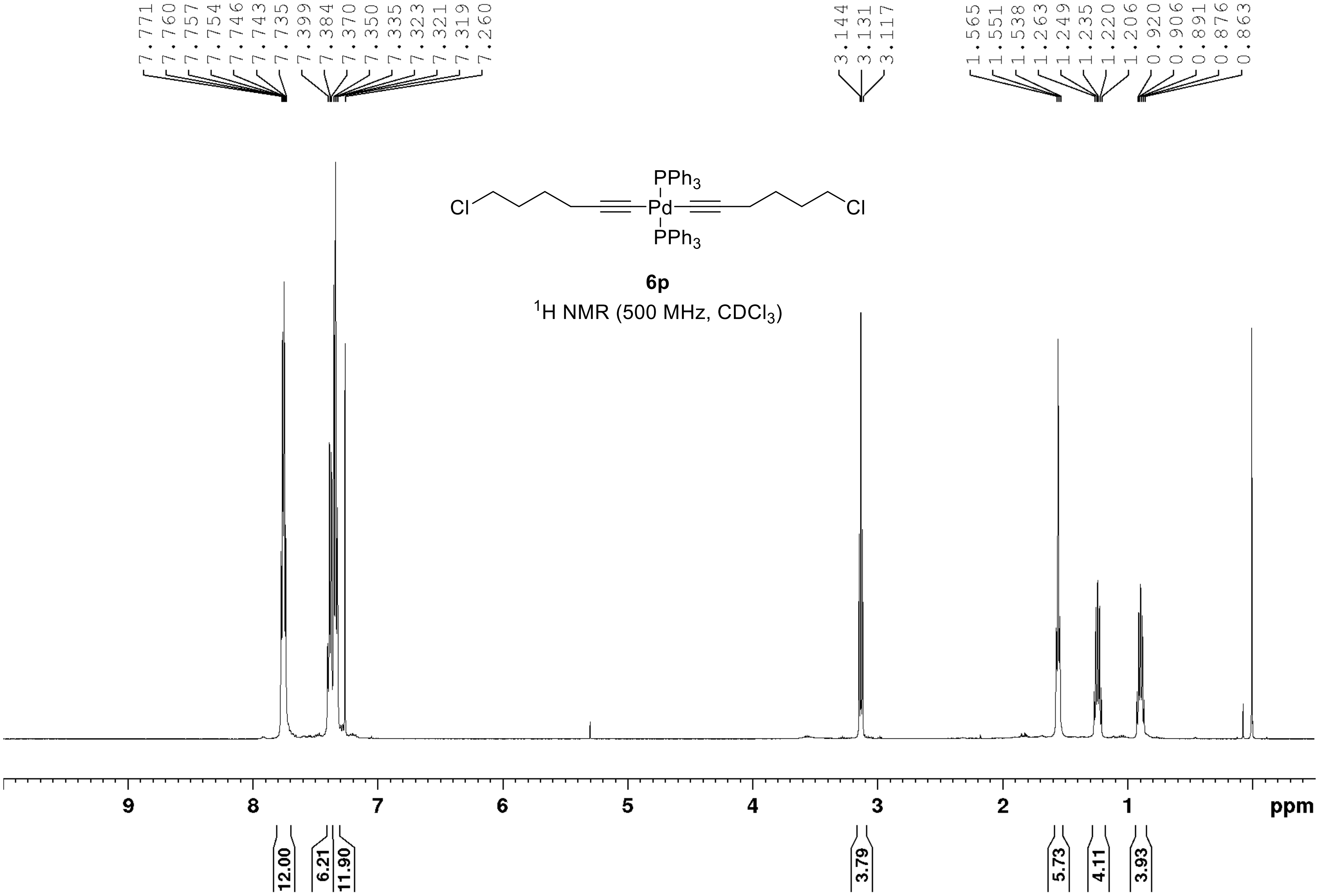
7.771
7.760
7.757
7.754
7.746
7.743
7.735
7.399
7.384
7.370
7.350
7.335
7.323
7.321
7.319
7.260
3.144
3.131
3.117
1.565
1.551
1.538
1.263
1.249
1.235
1.220
1.206
0.920
0.906
0.891
0.876
0.863
PPh3
Cl
Pd
Cl
PPh3
6p
1H NMR (500 MHz, CDCl3)
9
8
7
6
5
4
3
2
1
ppm
12.00
6.21
11.90
3.79
5.73
4.11
3.93

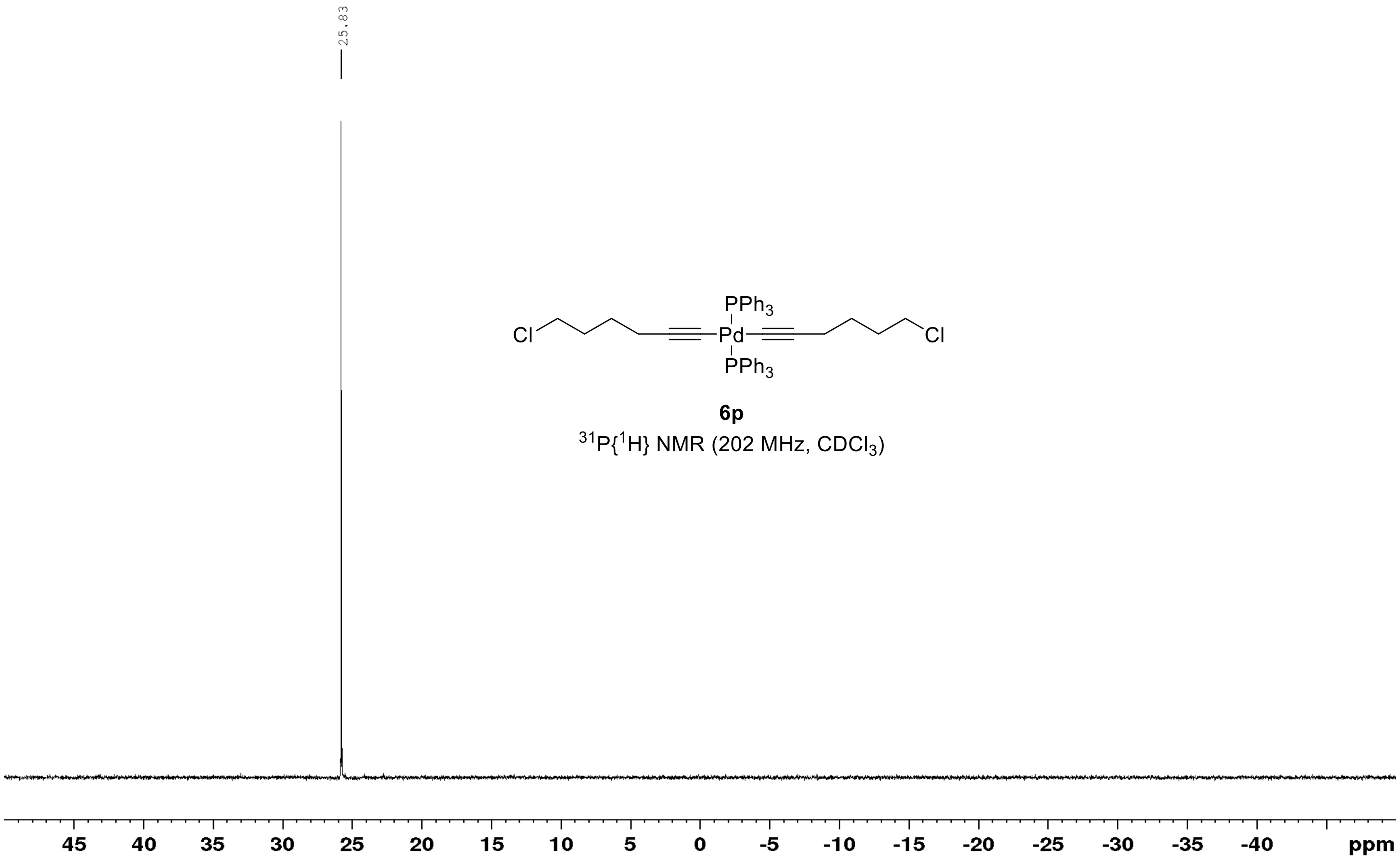

6p

$^{31}P\{^{1}H\}$ NMR (202 MHz, $CDCl_3$)

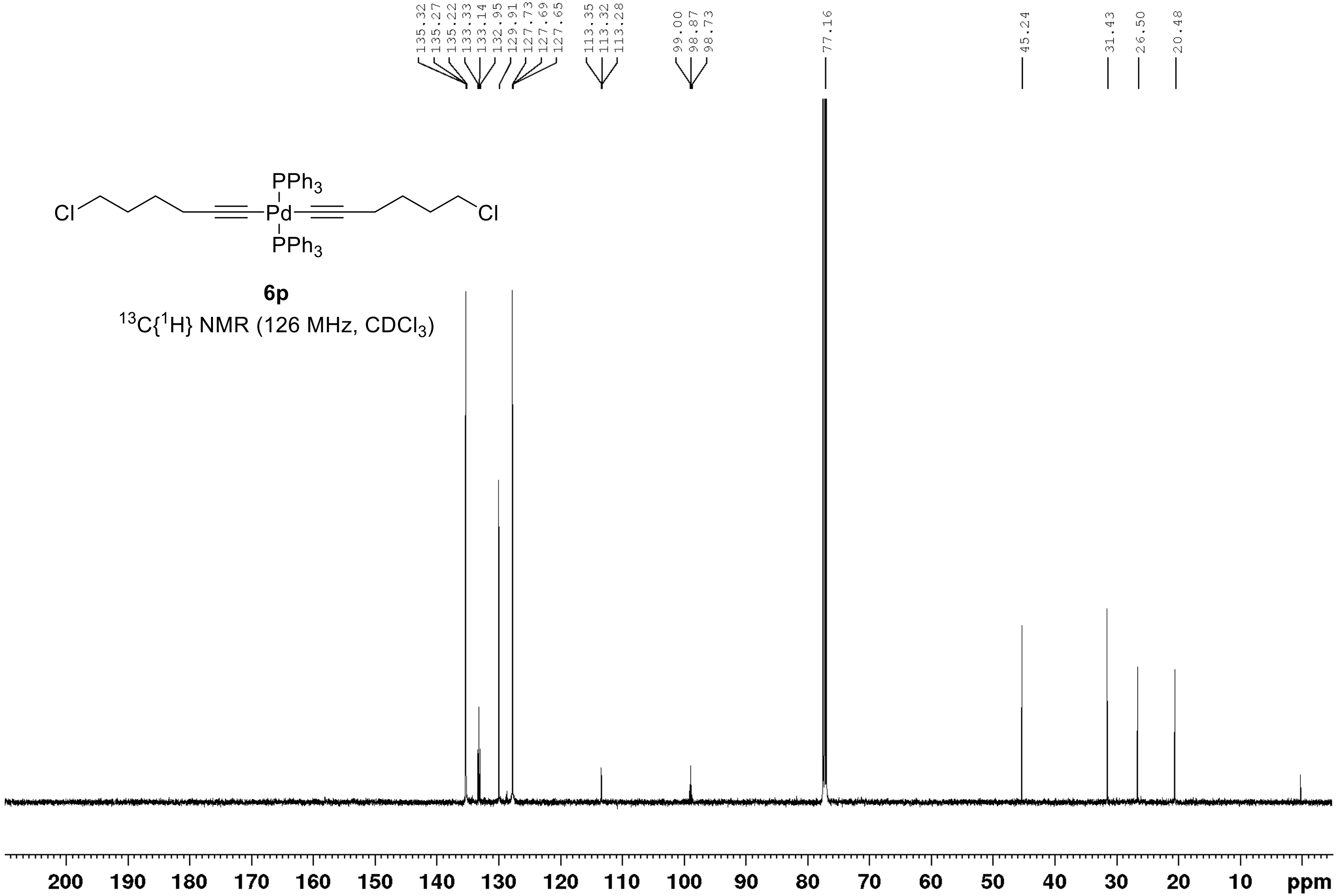

6p

$^{13}C\{^1H\}$ NMR (126 MHz, $CDCl_3$)

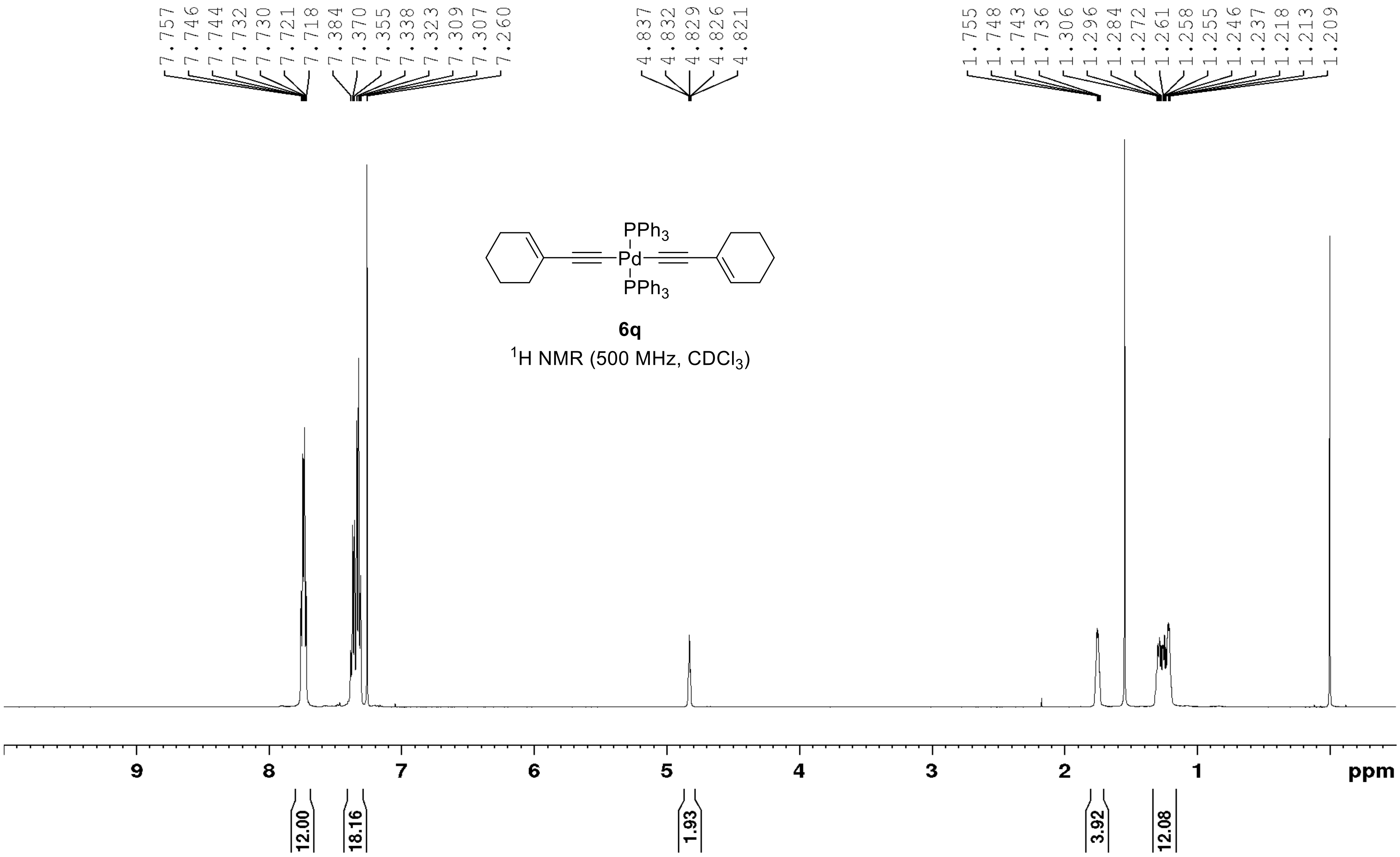

7.757
7.746
7.744
7.732
7.730
7.721
7.718
7.384
7.370
7.355
7.338
7.323
7.309
7.307
7.260
4.837
4.832
4.829
4.826
4.821
1.755
1.748
1.743
1.736
1.306
1.296
1.284
1.272
1.261
1.258
1.255
1.246
1.237
1.218
1.213
1.209
PPh3
Pd
PPh3
6q
1H NMR (500 MHz, CDCl3)
9
8
7
6
5
4
3
2
1
ppm
12.00
18.16
1.93
3.92
12.08

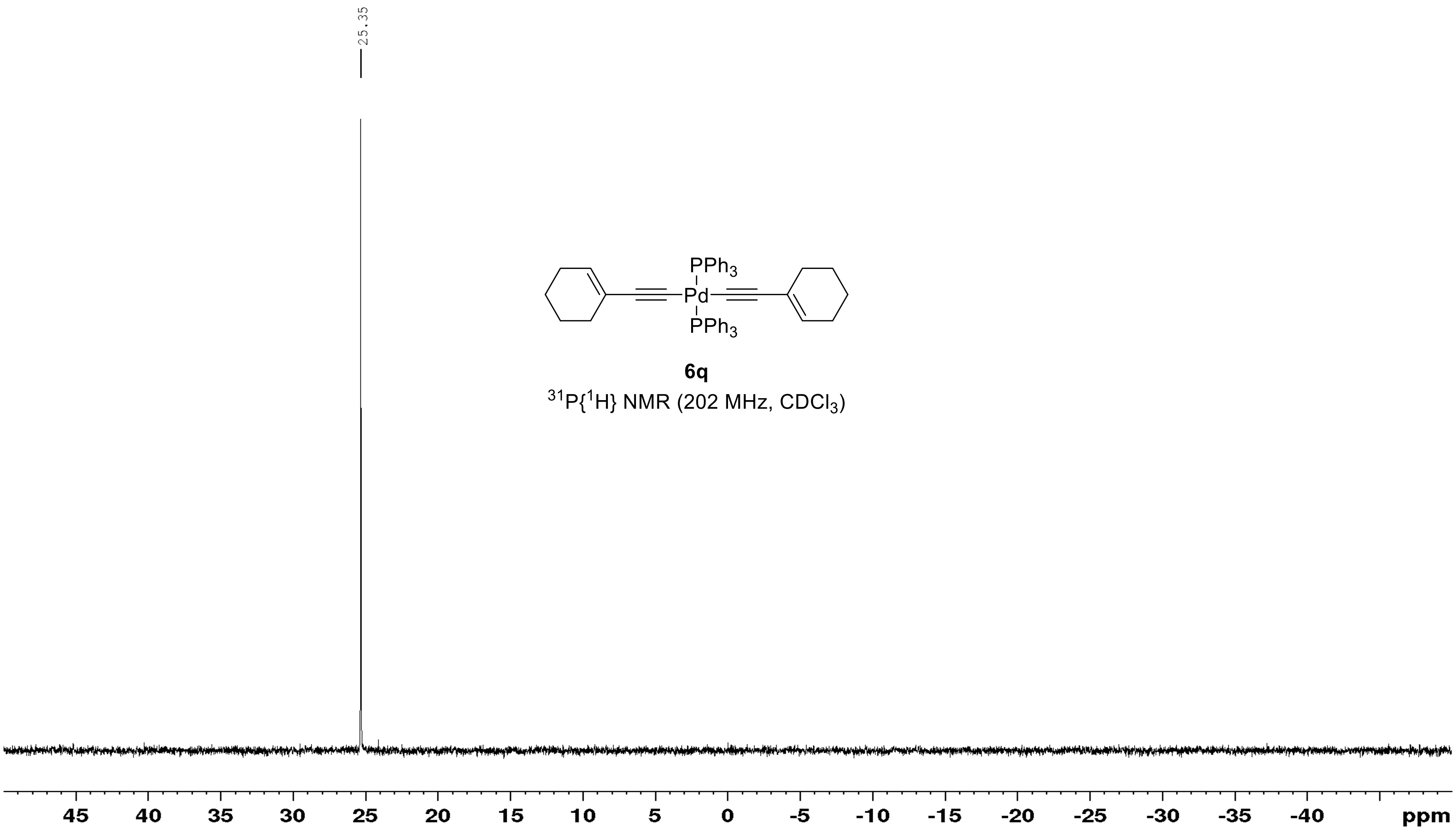

6q

$^{31}P\{^1H\}$ NMR (202 MHz, $CDCl_3$)

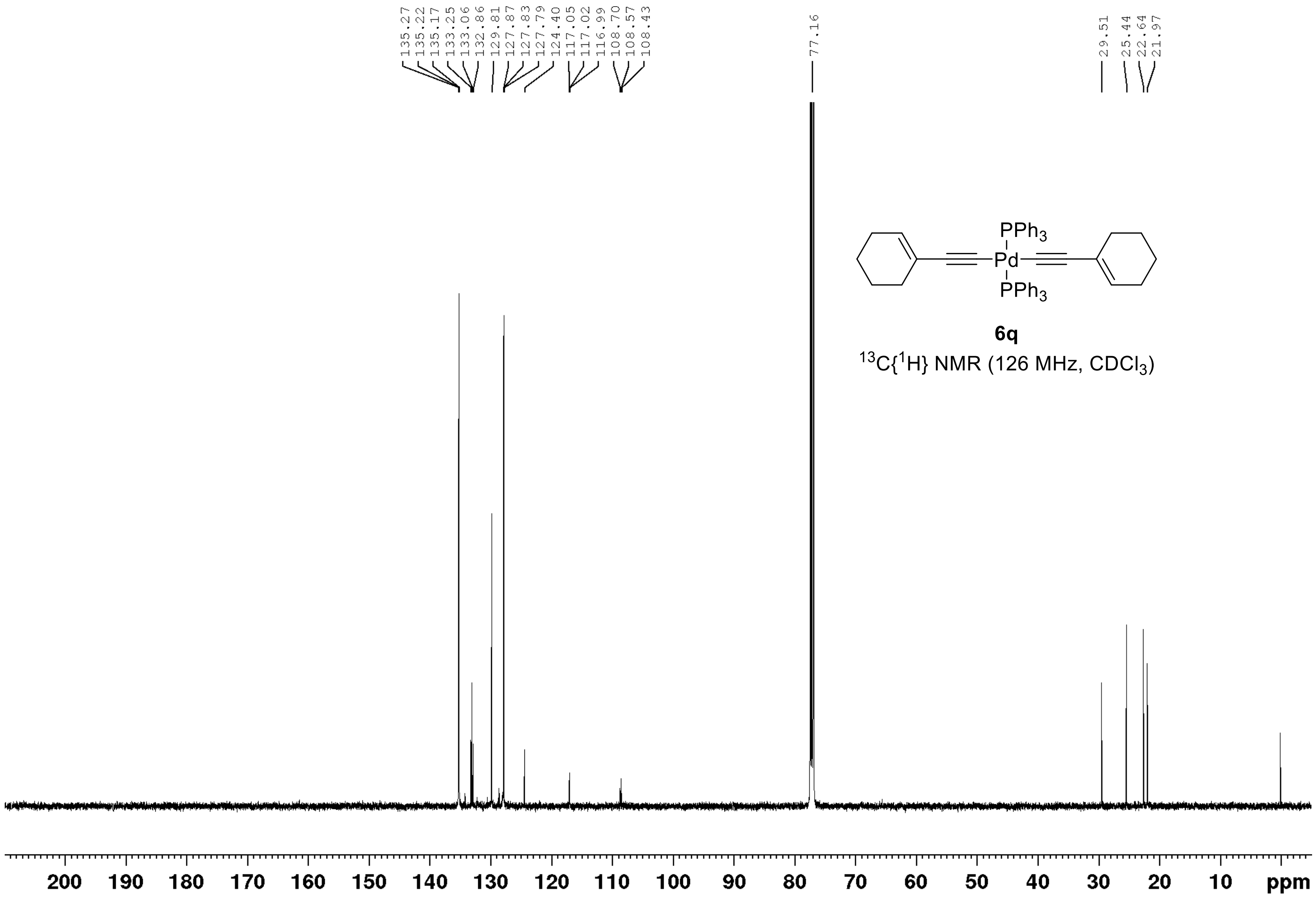
135.27
135.22
135.17
133.25
133.06
132.86
129.81
127.87
127.83
127.79
124.40
117.05
117.02
116.99
108.70
108.57
108.43
77.16
29.51
25.44
22.64
21.97
PPh3
Pd
PPh3
6q
13C{1H} NMR (126 MHz, CDCl3)
200
190
180
170
160
150
140
130
120
110
100
90
80
70
60
50
40
30
20
10
ppm

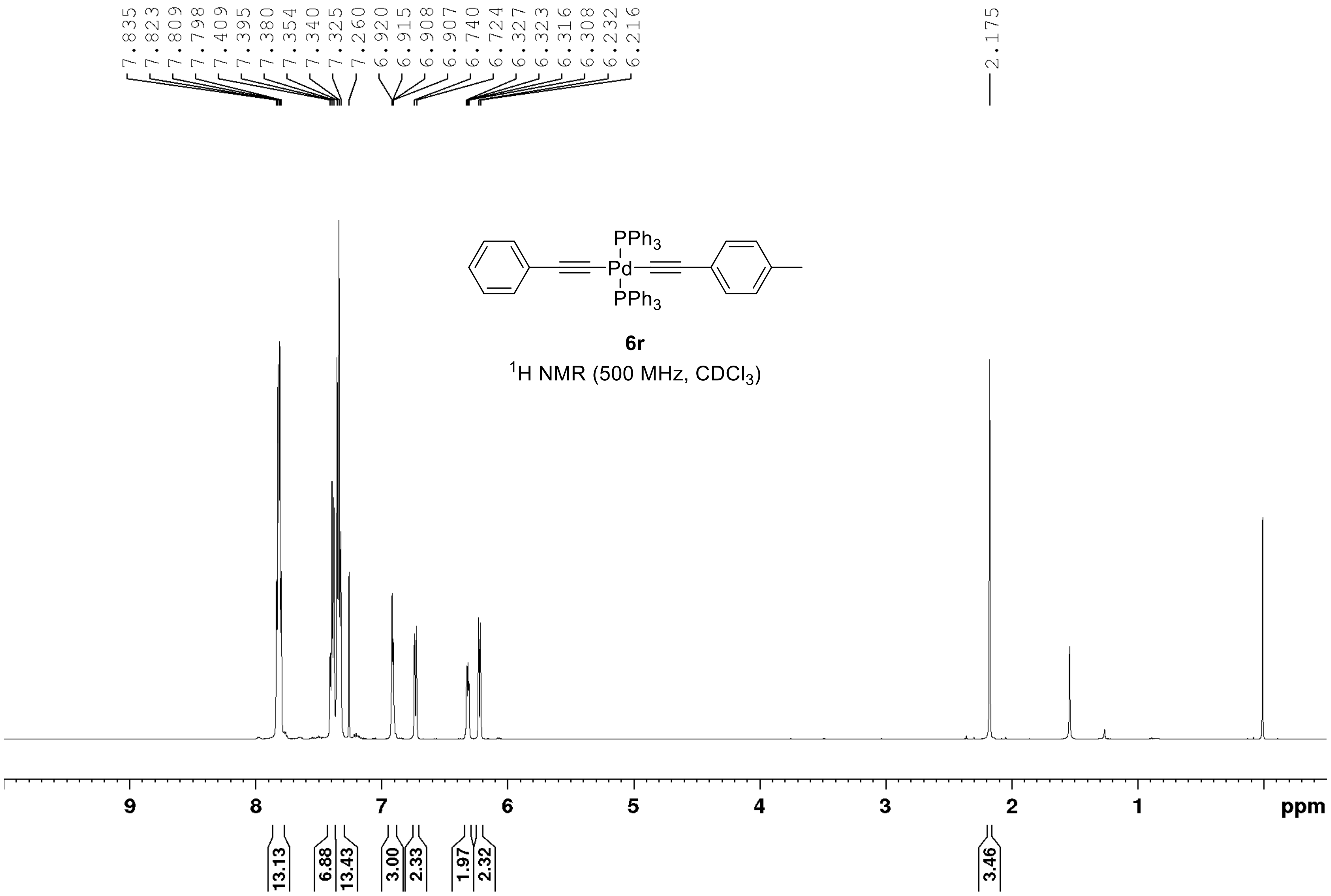
7.835
7.823
7.809
7.798
7.409
7.395
7.380
7.354
7.340
7.325
7.260
6.920
6.915
6.908
6.907
6.740
6.724
6.327
6.323
6.316
6.308
6.232
6.216
2.175
PPh3
Pd
PPh3
6r
1H NMR (500 MHz, CDCl3)
9
8
7
6
5
4
3
2
1
ppm
13.13
6.88
13.43
3.00
2.33
1.97
2.32
3.46

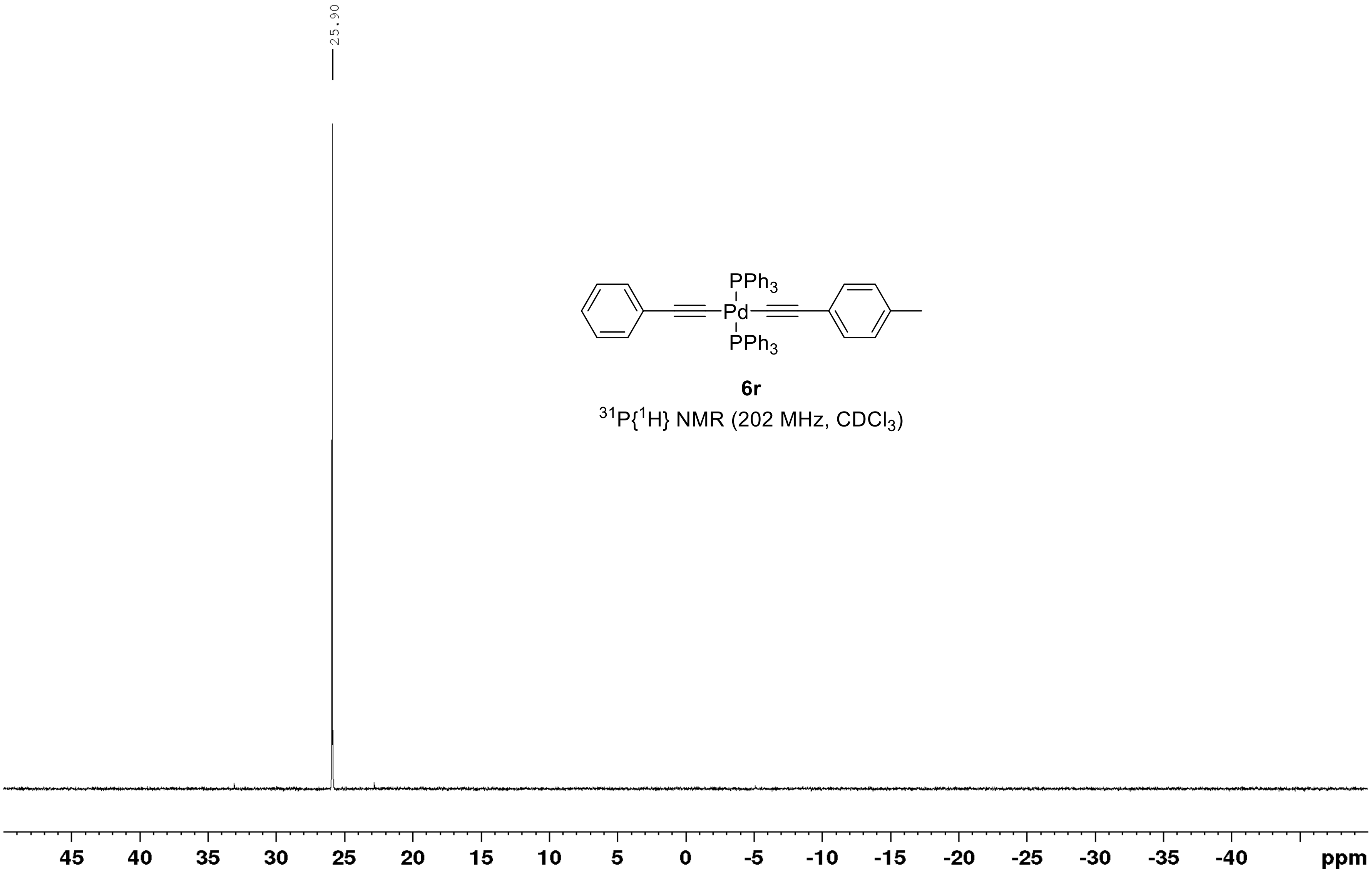
25.90
PPh3
Pd
PPh3
6r
31P{1H} NMR (202 MHz, CDCl3)
45 40 35 30 25 20 15 10 5 0 -5 -10 -15 -20 -25 -30 -35 -40 ppm

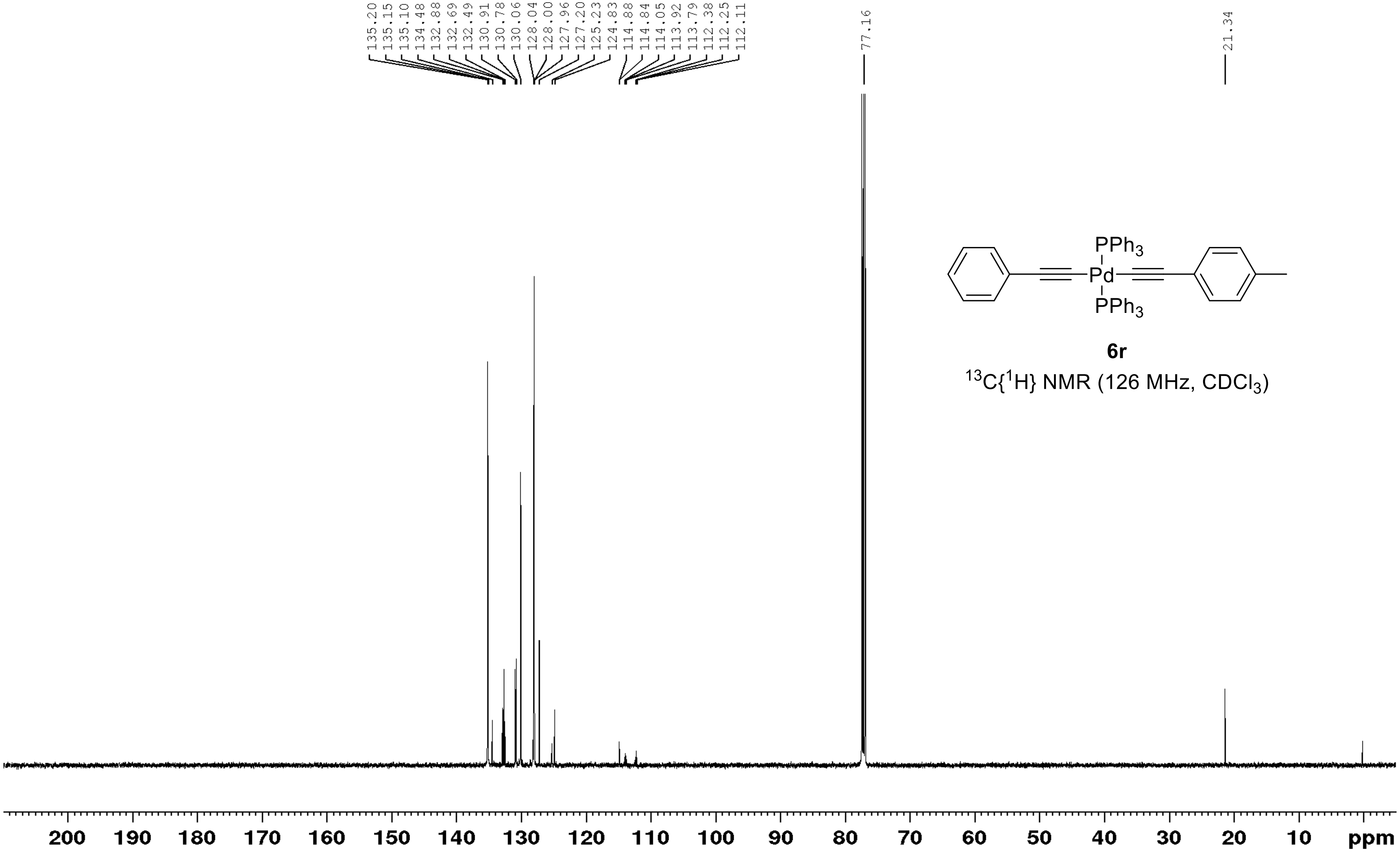

6r

$^{13}C\{^{1}H\}$ NMR (126 MHz, $CDCl_3$)

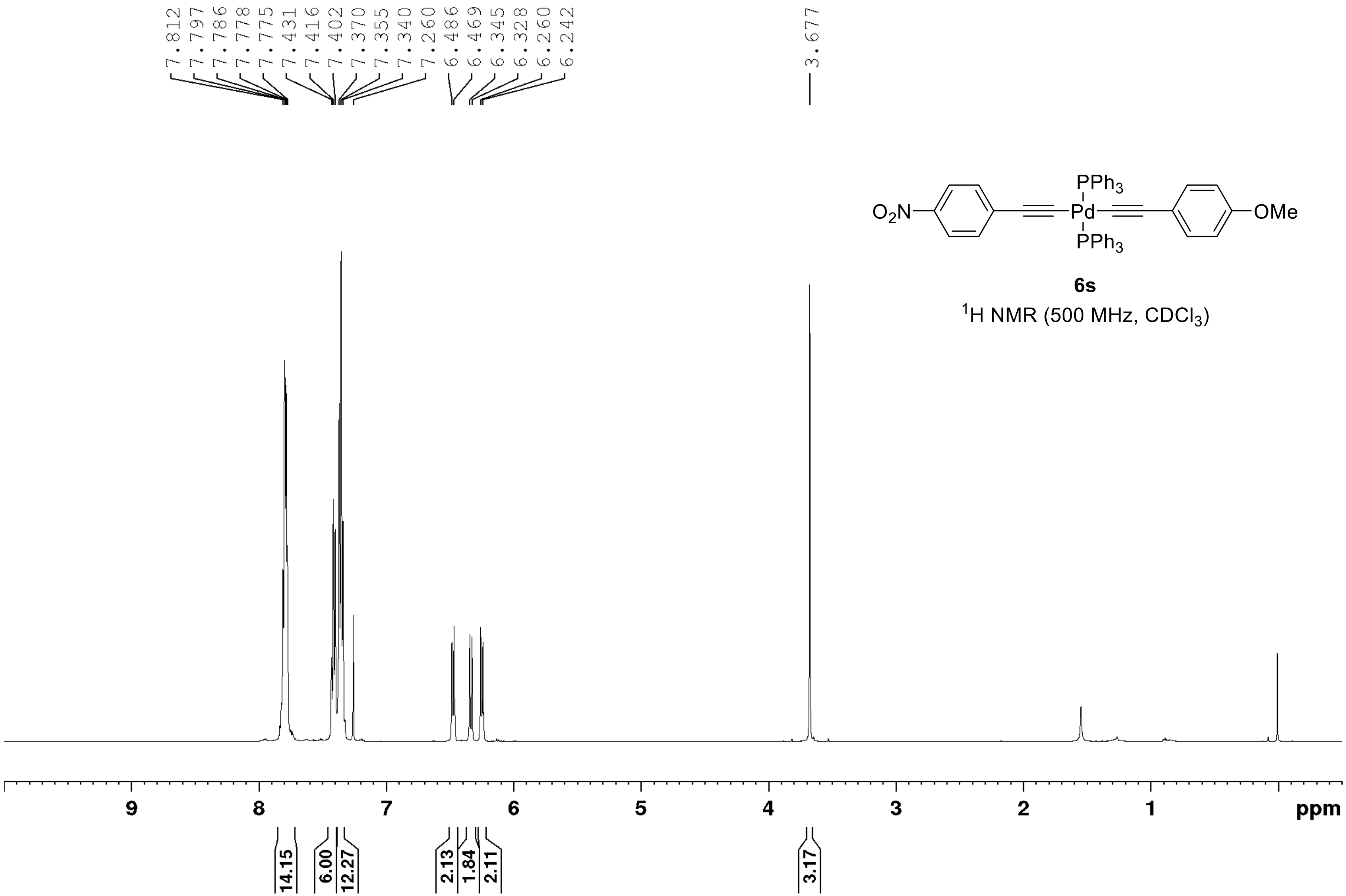
7.812
7.797
7.786
7.778
7.775
7.431
7.416
7.402
7.370
7.355
7.340
7.260
6.486
6.469
6.345
6.328
6.260
6.242
3.677
PPh3
O2N
Pd
OMe
PPh3
6s
1H NMR (500 MHz, CDCl3)
9
8
7
6
5
4
3
2
1
ppm
14.15
6.00
12.27
2.13
1.84
2.11
3.17

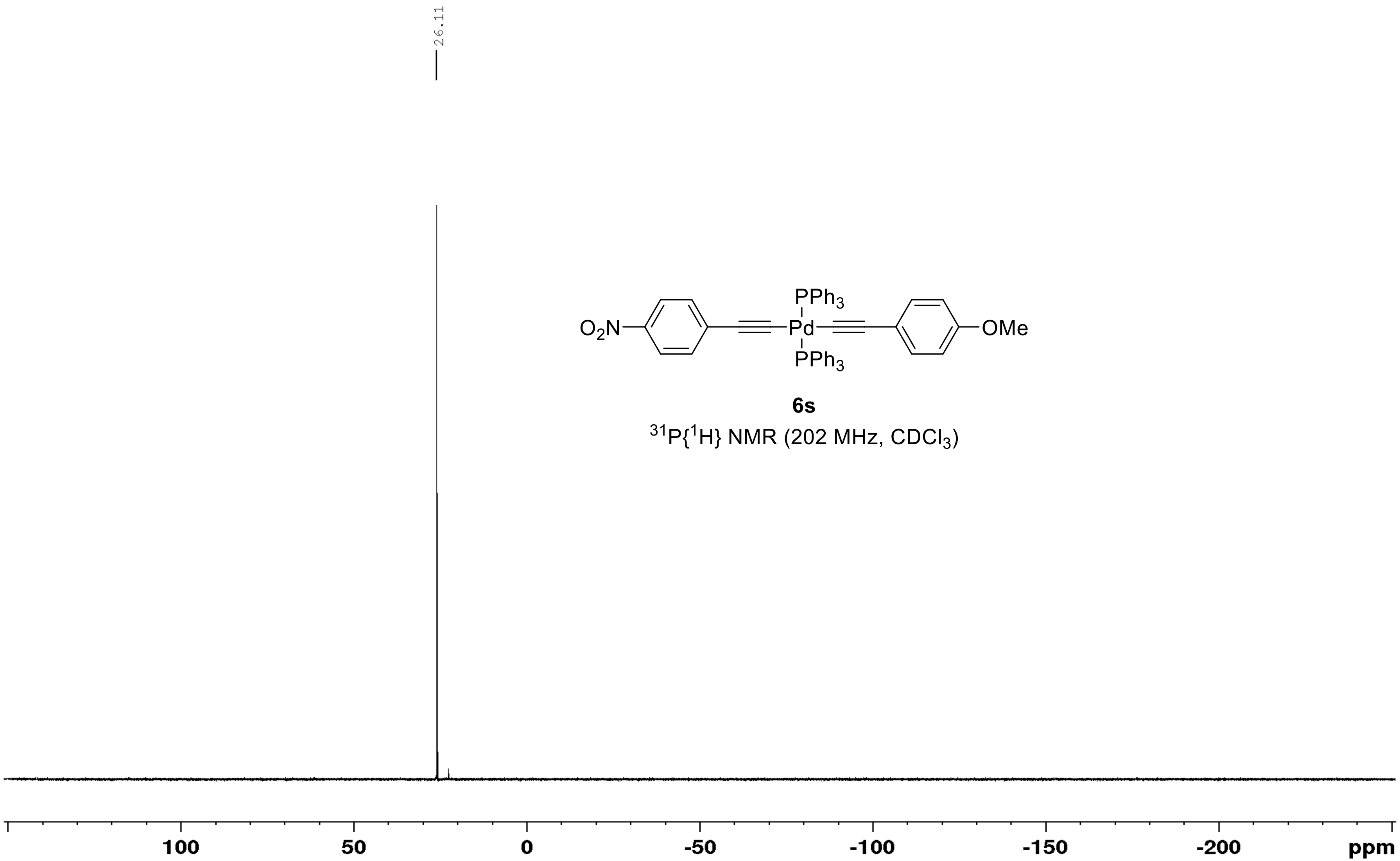

6s

$^{31}P\{^1H\}$ NMR (202 MHz, $CDCl_3$)

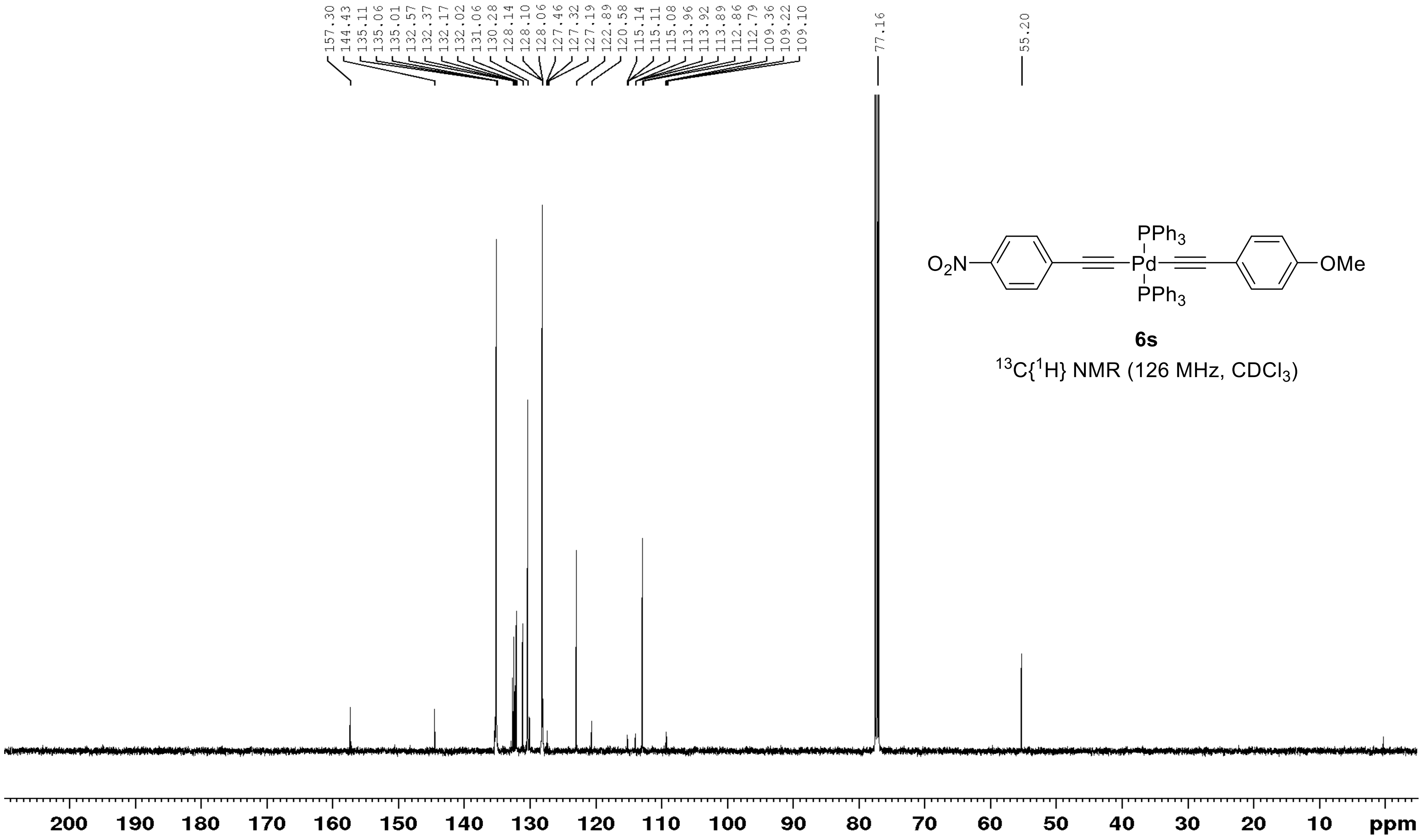

6s

$^{13}C\{^1H\}$ NMR (126 MHz, $CDCl_3$)

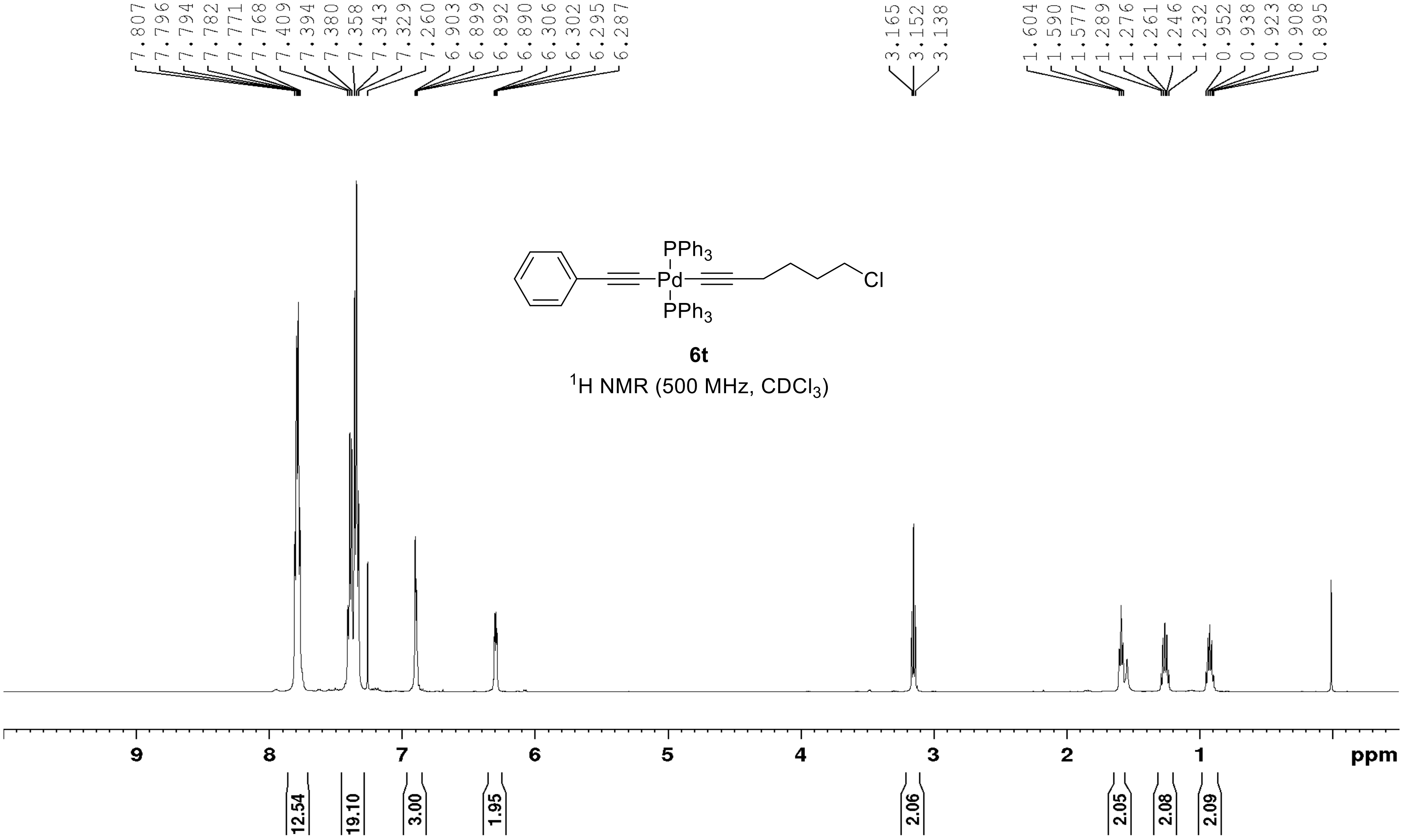

7.807
7.796
7.794
7.782
7.771
7.768
7.409
7.394
7.380
7.358
7.343
7.329
7.260
6.903
6.899
6.892
6.890
6.306
6.302
6.295
6.287
3.165
3.152
3.138
1.604
1.590
1.577
1.289
1.276
1.261
1.246
1.232
0.952
0.938
0.923
0.908
0.895
PPh3
Pd
Cl
PPh3
6t
1H NMR (500 MHz, CDCl3)
9
8
7
6
5
4
3
2
1
ppm
12.54
19.10
3.00
1.95
2.06
2.05
2.08
2.09

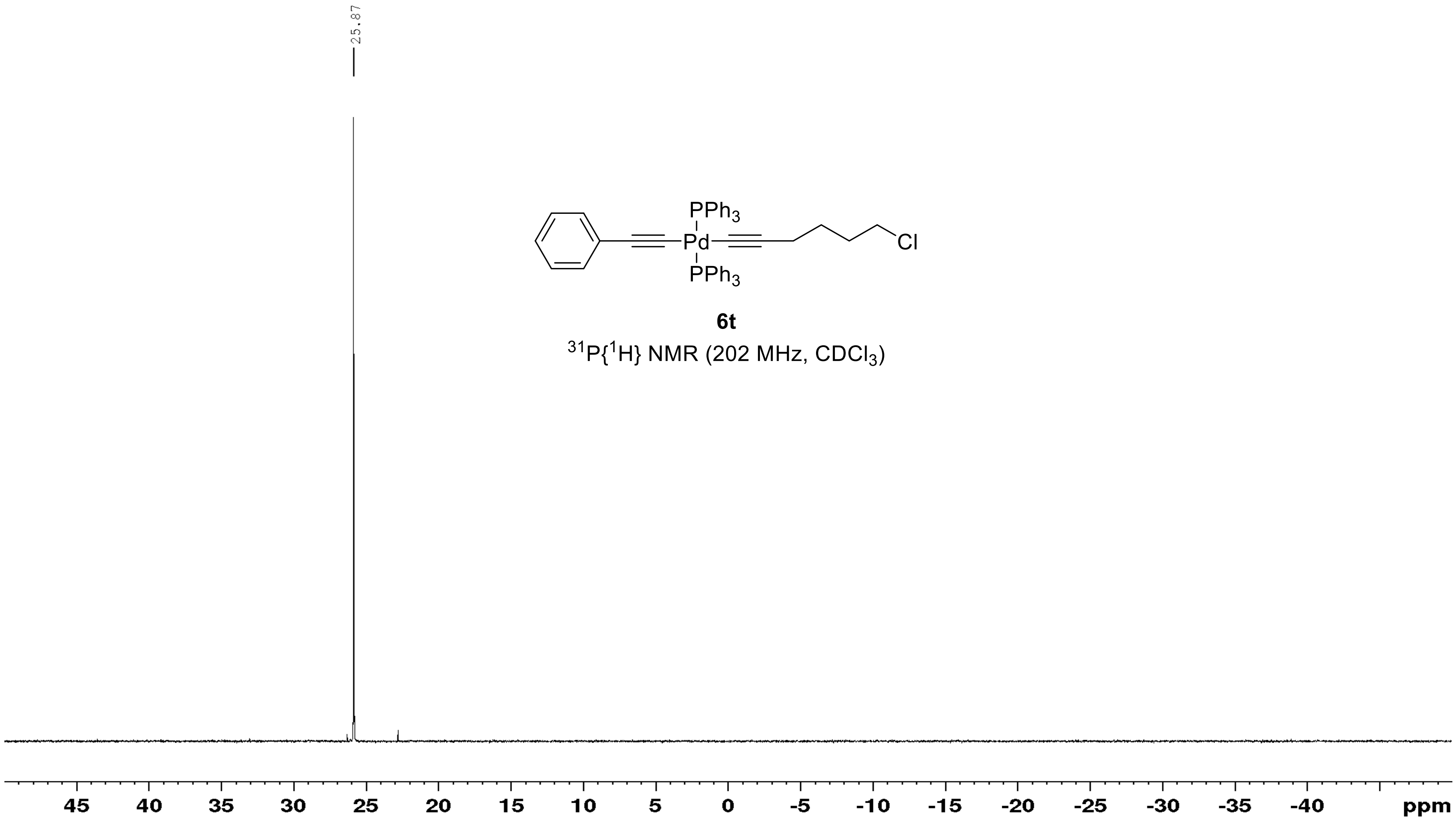

6t

$^{31}P\{^{1}H\}$ NMR (202 MHz, $CDCl_3$)

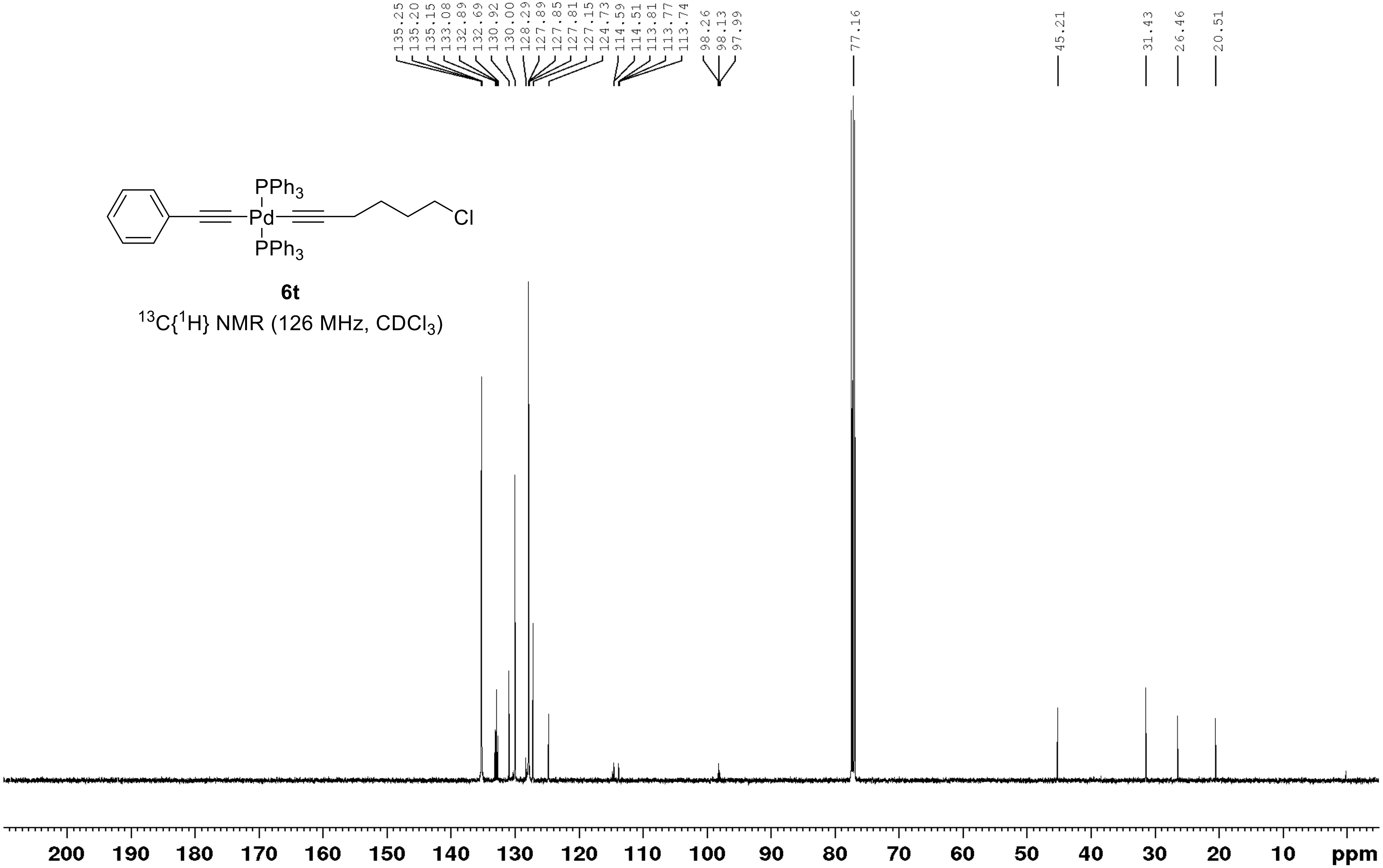

6t

$^{13}C\{^1H\}$ NMR (126 MHz, $CDCl_3$)

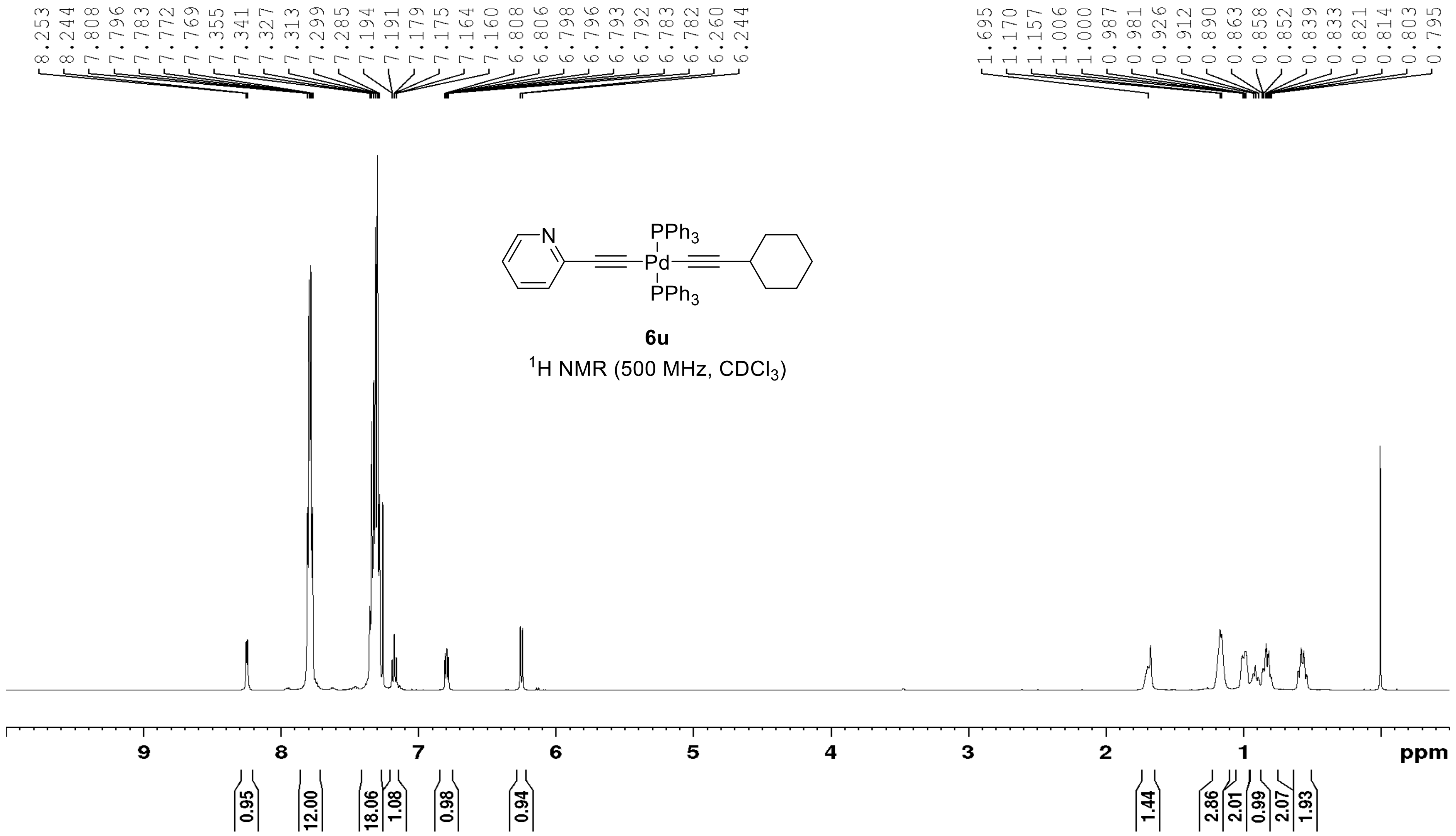
8.253 8.244 7.808 7.796 7.783 7.772 7.769 7.355 7.341 7.327 7.313 7.299 7.285 7.194 7.191 7.179 7.175 7.164 7.160 6.808 6.806 6.798 6.796 6.793 6.792 6.783 6.782 6.260 6.244
1.695 1.170 1.157 1.006 1.000 0.987 0.981 0.926 0.912 0.890 0.863 0.858 0.852 0.839 0.833 0.821 0.814 0.803 0.795
N
PPh3
Pd
PPh3
6u
1H NMR (500 MHz, CDCl3)
9 8 7 6 5 4 3 2 1 ppm
0.95 12.00 18.06 1.08 0.98 0.94 1.44 2.86 2.01 0.99 2.07 1.93

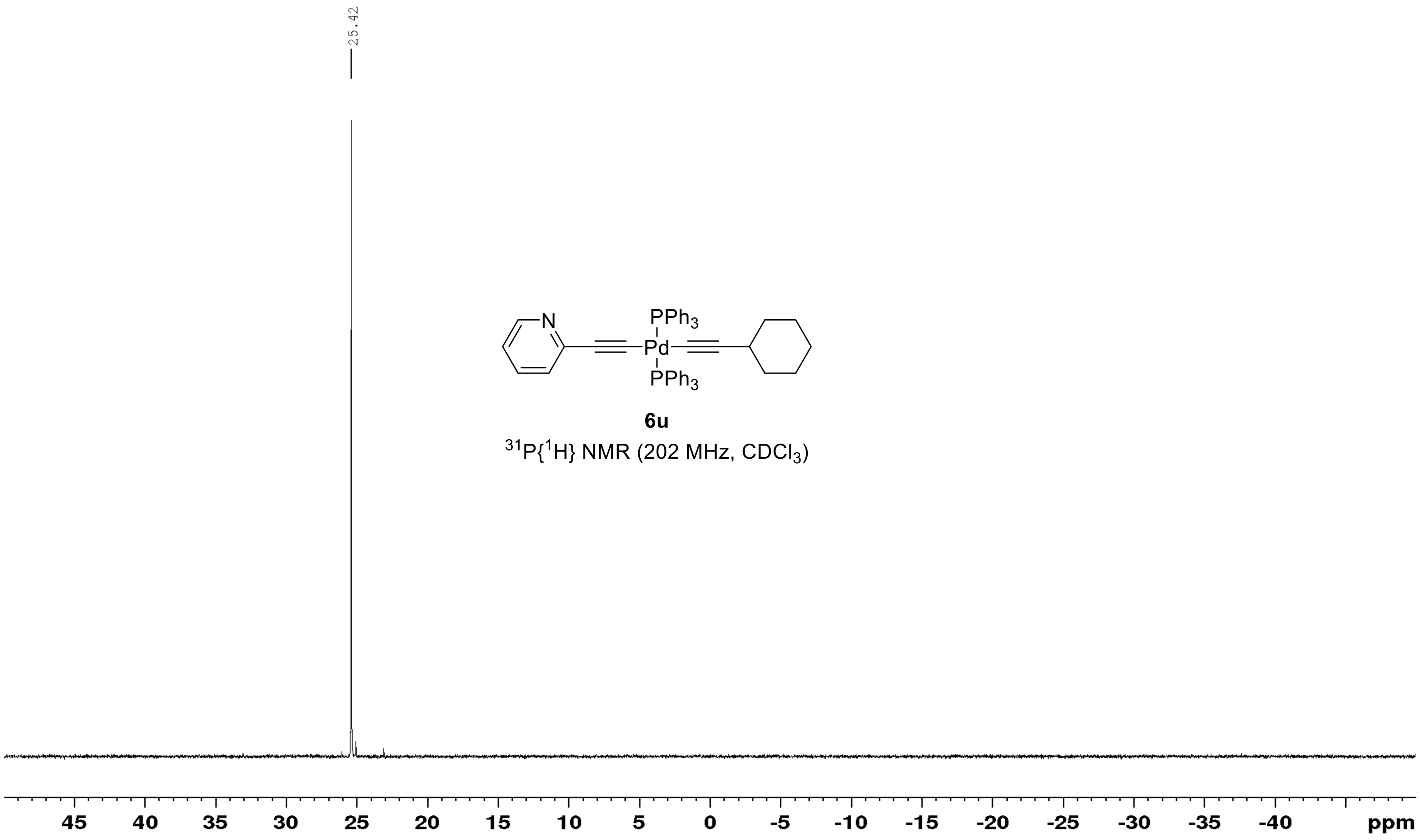
25.42
PPh3
N
Pd
PPh3
6u
31P{1H} NMR (202 MHz, CDCl3)
45 40 35 30 25 20 15 10 5 0 -5 -10 -15 -20 -25 -30 -35 -40 ppm

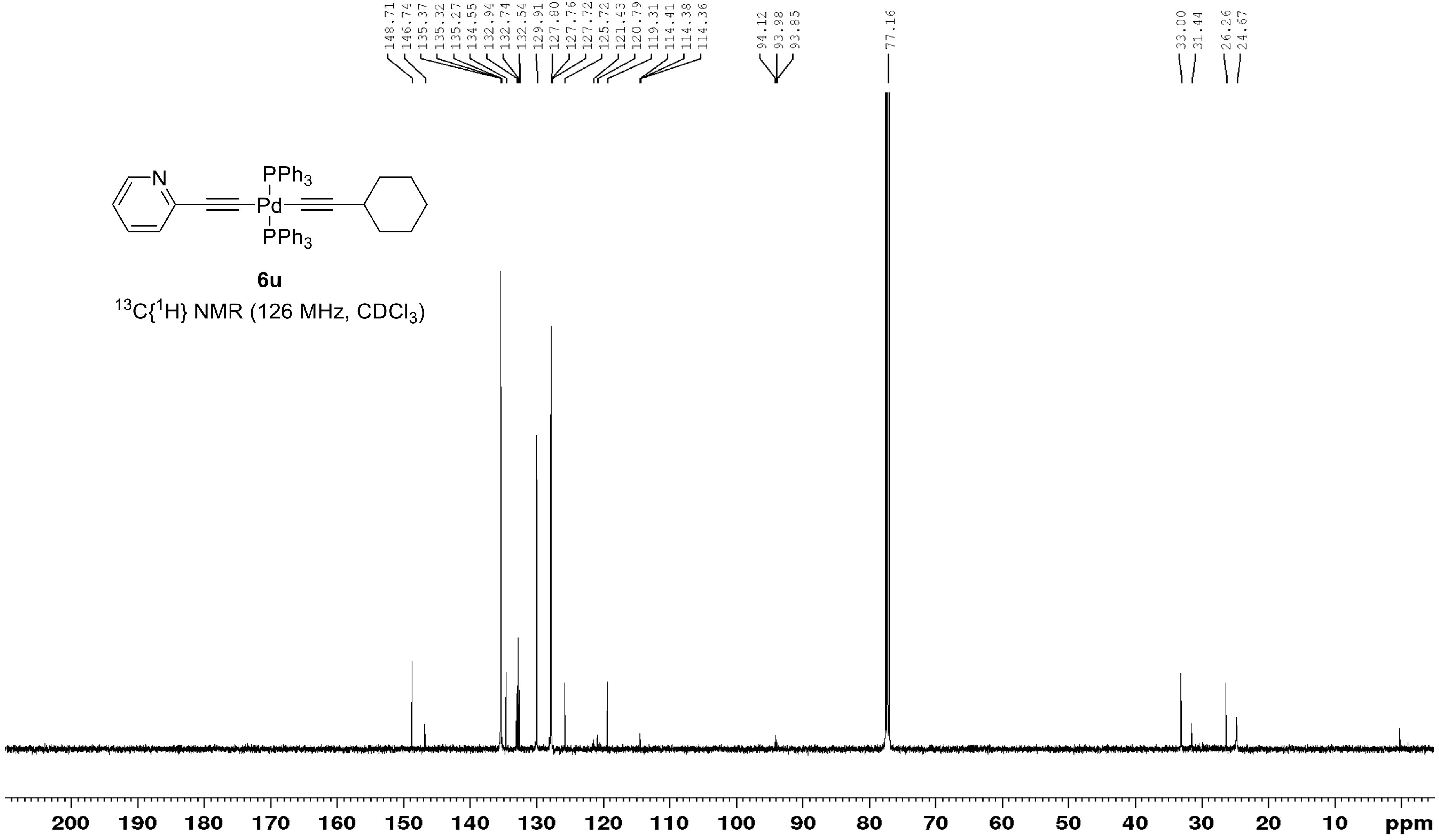

6u

$^{13}C\{^{1}H\}$ NMR (126 MHz, $CDCl_3$)

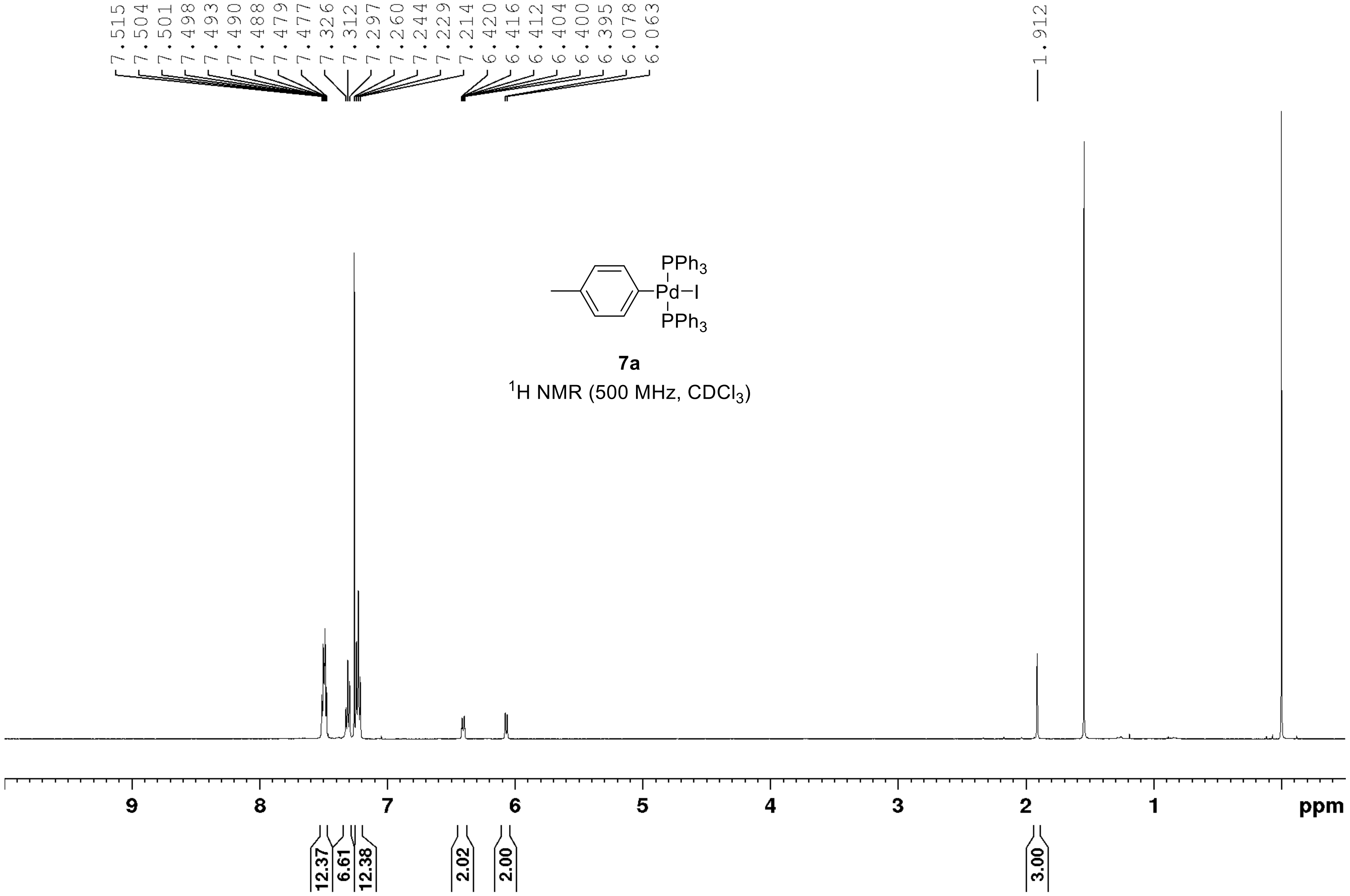
7.515
7.504
7.501
7.498
7.493
7.490
7.488
7.479
7.477
7.326
7.312
7.297
7.260
7.244
7.229
7.214
6.420
6.416
6.412
6.404
6.400
6.395
6.078
6.063
1.912
PPh3
Pd
I
PPh3
7a
1H NMR (500 MHz, CDCl3)
9
8
7
6
5
4
3
2
1
ppm
12.37
6.61
12.38
2.02
2.00
3.00

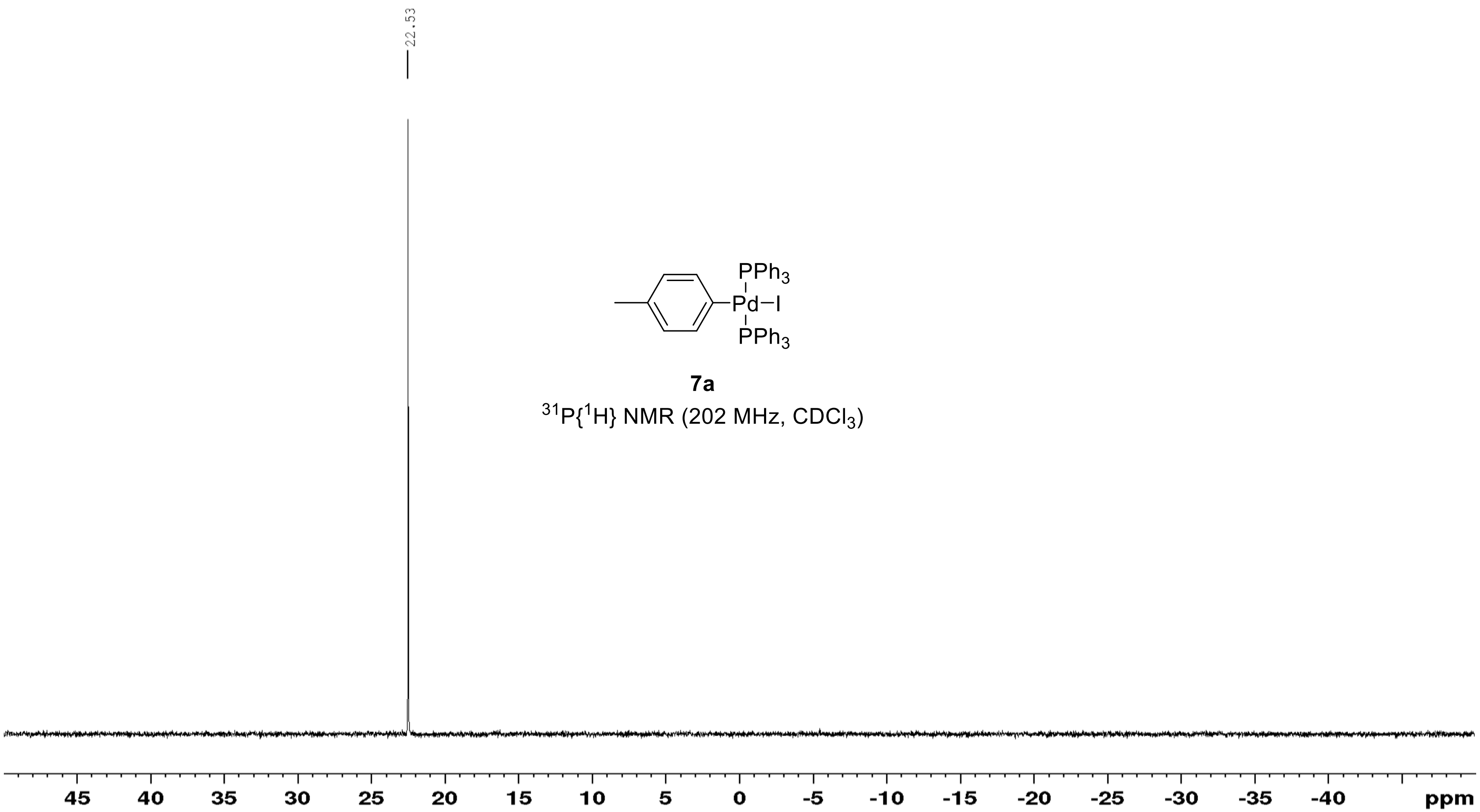

7a

$^{31}P\{^{1}H\}$ NMR (202 MHz, $CDCl_3$)

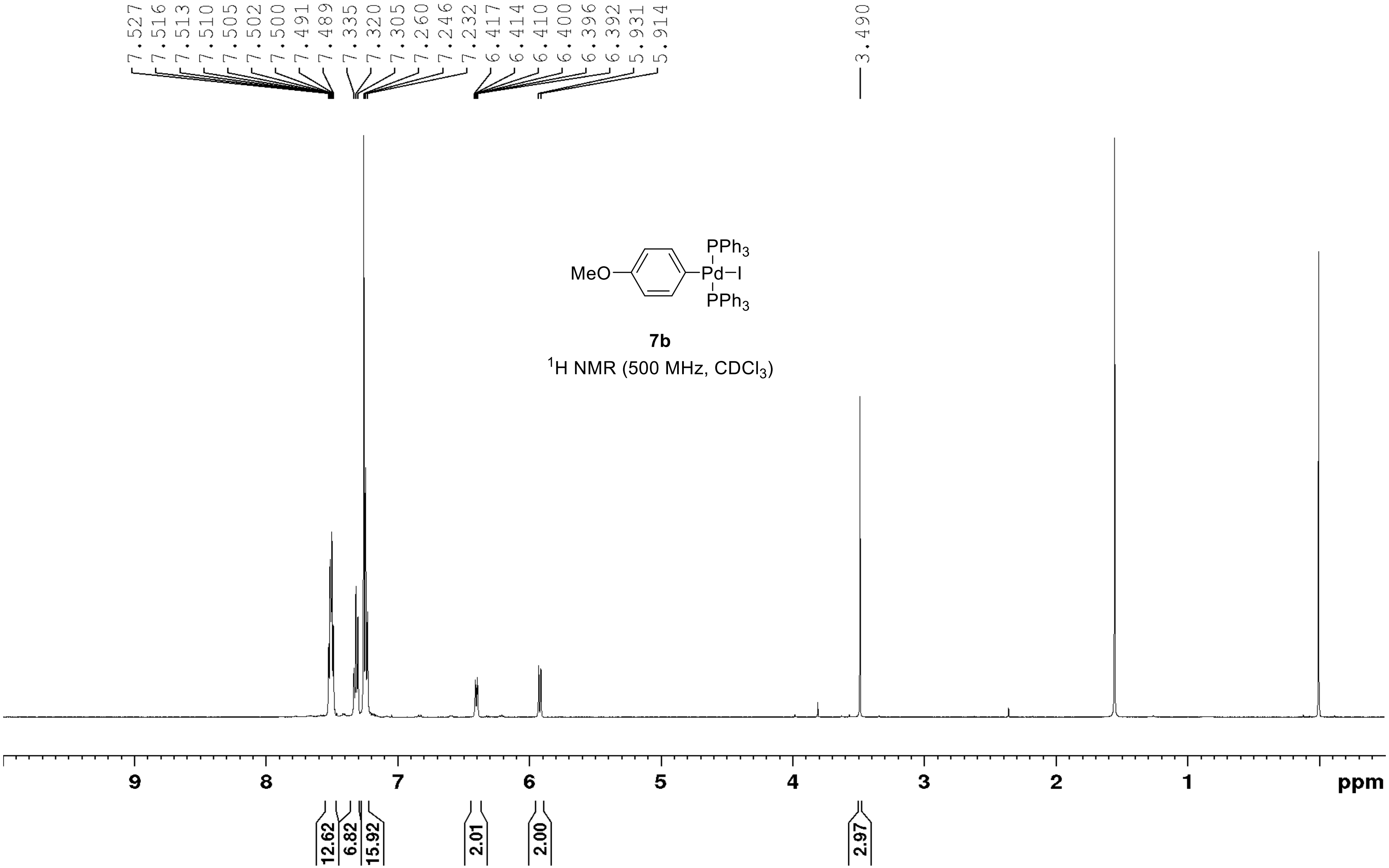
7.527
7.516
7.513
7.510
7.505
7.502
7.500
7.491
7.489
7.335
7.320
7.305
7.260
7.246
7.232
6.417
6.414
6.410
6.400
6.396
6.392
5.931
5.914
3.490
MeO
PPh3
Pd
I
PPh3
7b
1H NMR (500 MHz, CDCl3)
9
8
7
6
5
4
3
2
1
ppm
12.62
6.82
15.92
2.01
2.00
2.97

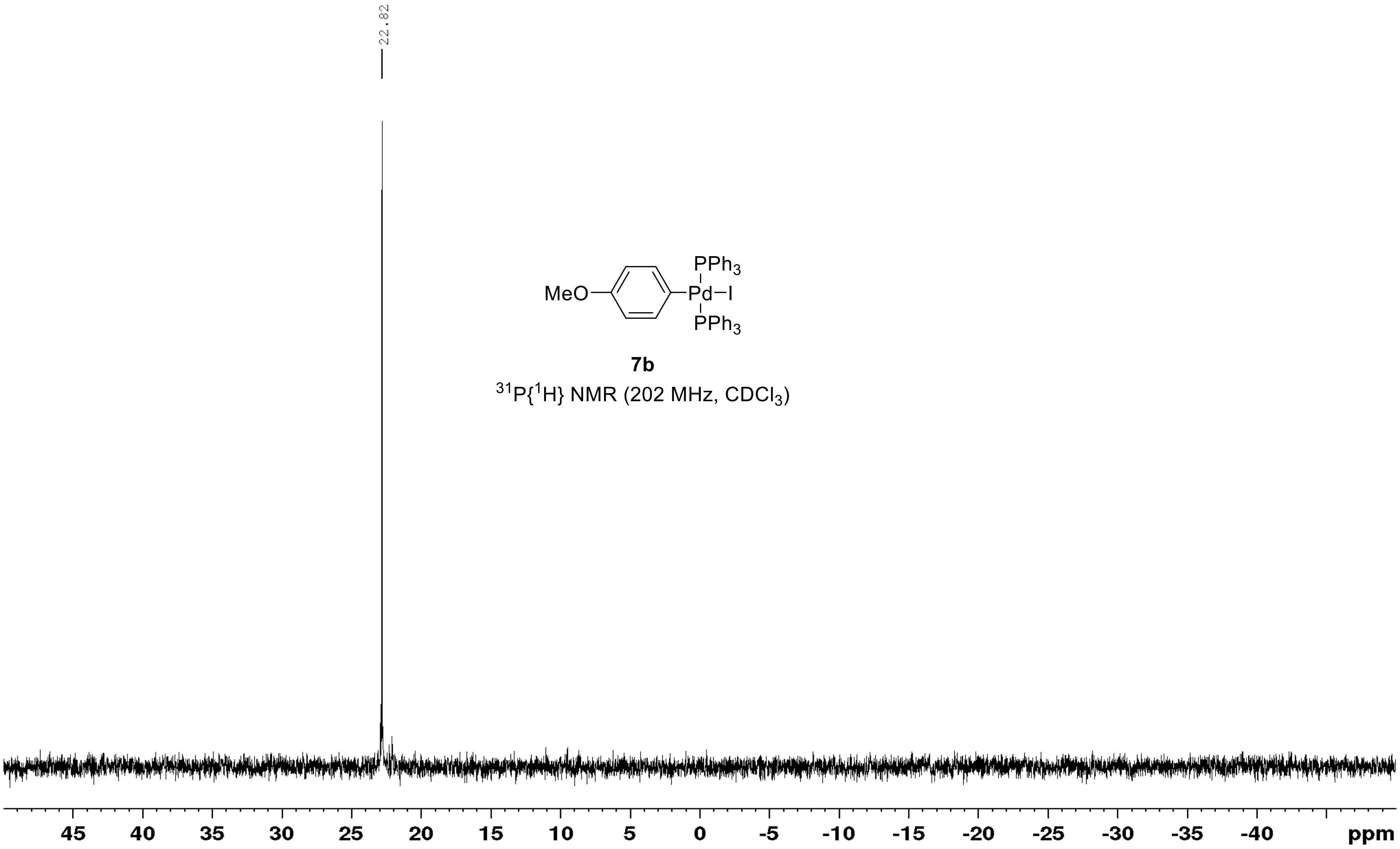

7b

$^{31}P\{^1H\}$ NMR (202 MHz, $CDCl_3$)

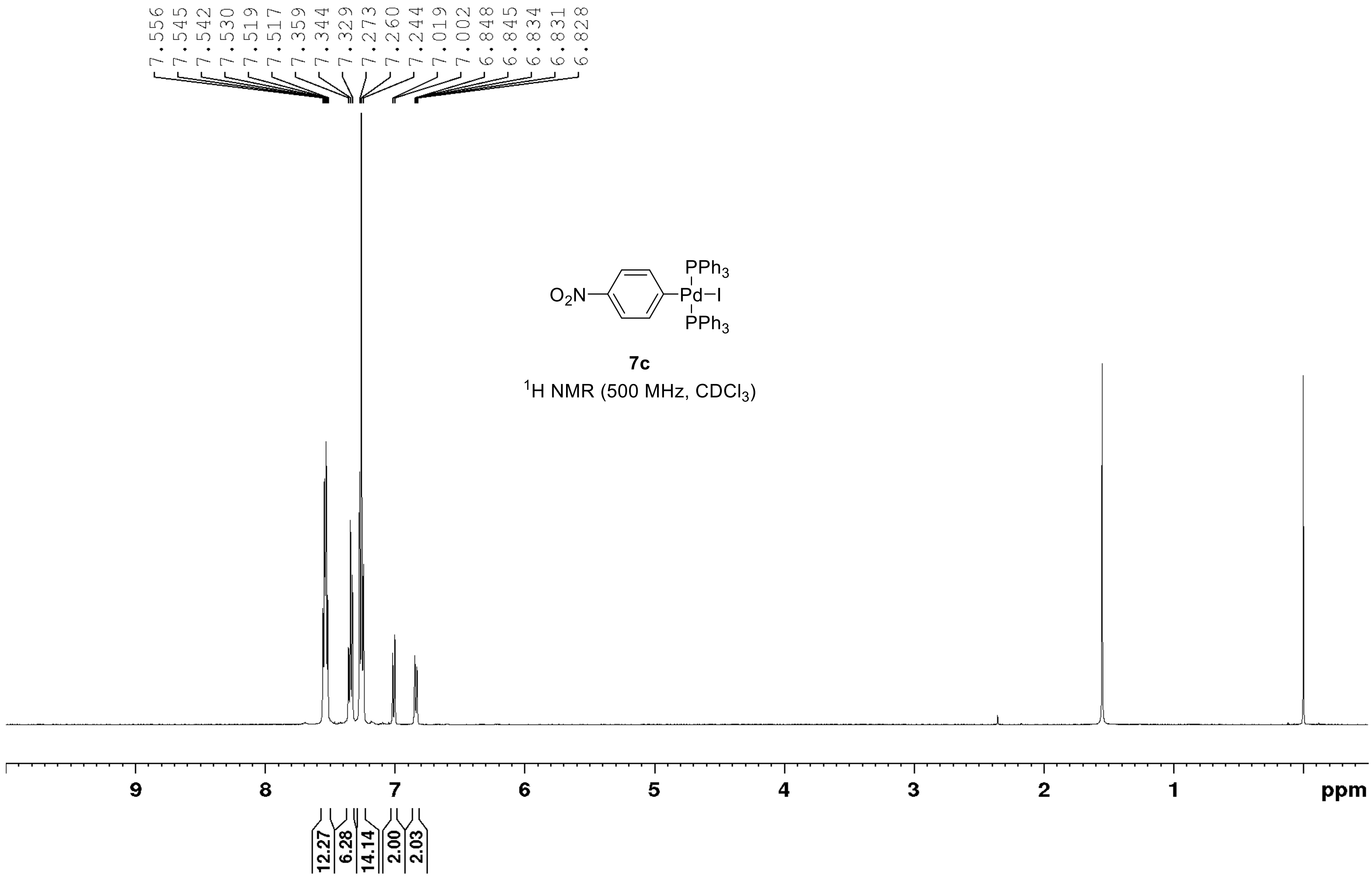
7.556
7.545
7.542
7.530
7.519
7.517
7.359
7.344
7.329
7.273
7.260
7.244
7.019
7.002
6.848
6.845
6.834
6.831
6.828
PPh3
O2N
Pd
I
PPh3
7c
1H NMR (500 MHz, CDCl3)
9
8
7
6
5
4
3
2
1
ppm
12.27
6.28
14.14
2.00
2.03

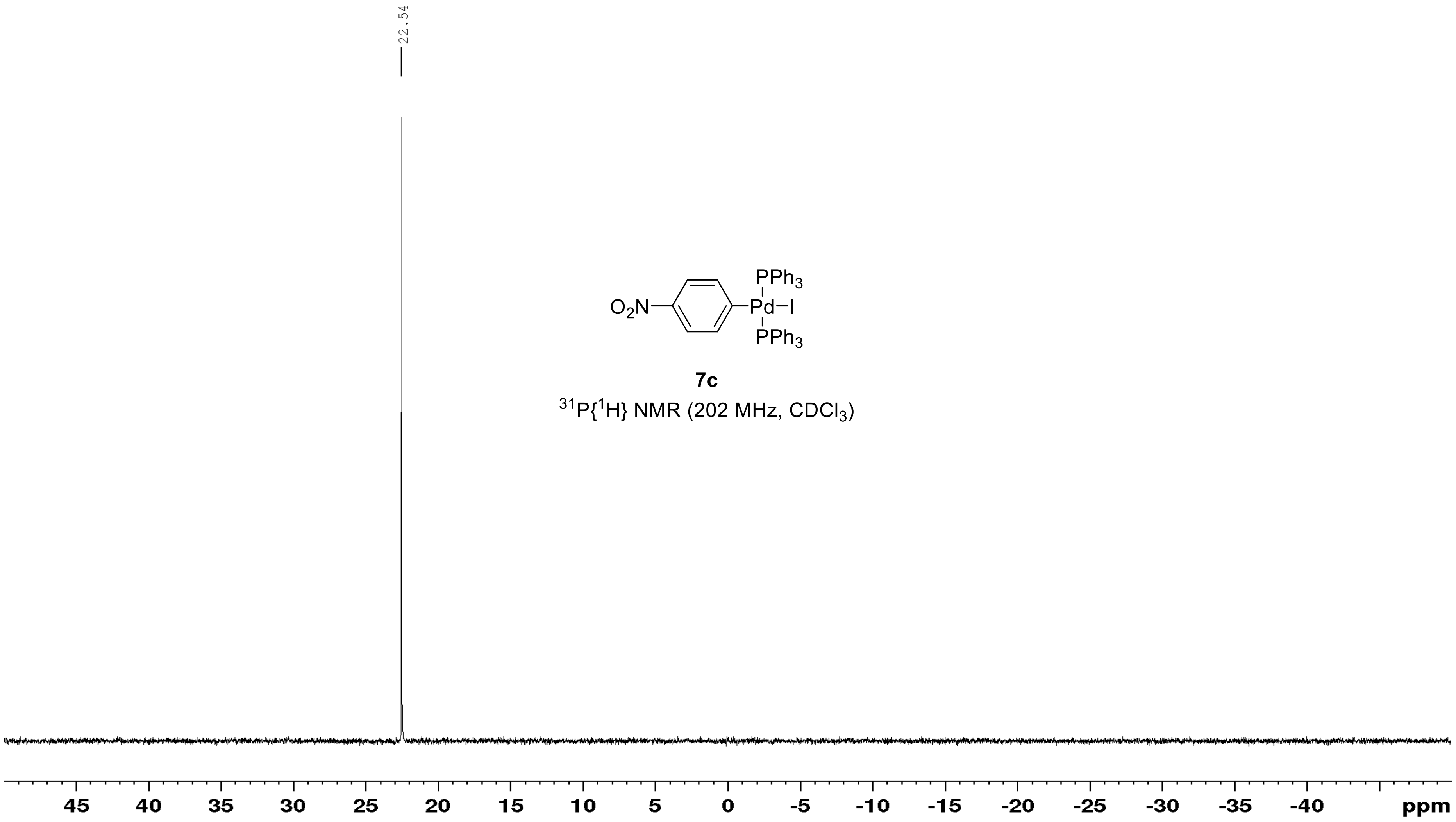
22.54
PPh3
O2N
Pd
I
PPh3
7c
31P{1H} NMR (202 MHz, CDCl3)
45 40 35 30 25 20 15 10 5 0 -5 -10 -15 -20 -25 -30 -35 -40 ppm

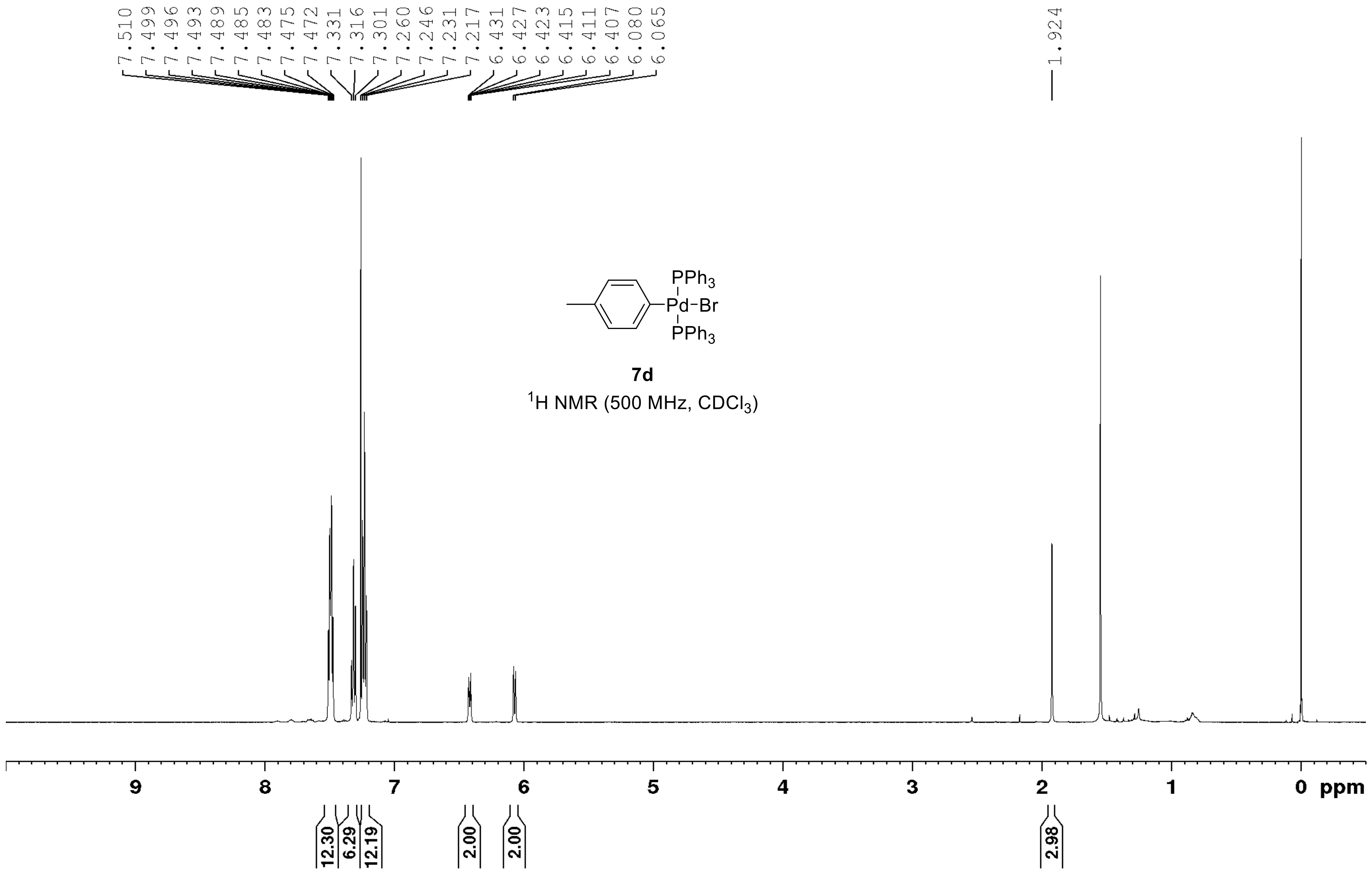
7.510
7.499
7.496
7.493
7.489
7.485
7.483
7.475
7.472
7.331
7.316
7.301
7.260
7.246
7.231
7.217
6.431
6.427
6.423
6.415
6.411
6.407
6.080
6.065
1.924
PPh3
Pd–Br
PPh3
7d
1H NMR (500 MHz, CDCl3)
9
8
7
6
5
4
3
2
1
0 ppm
12.30
6.29
12.19
2.00
2.00
2.98

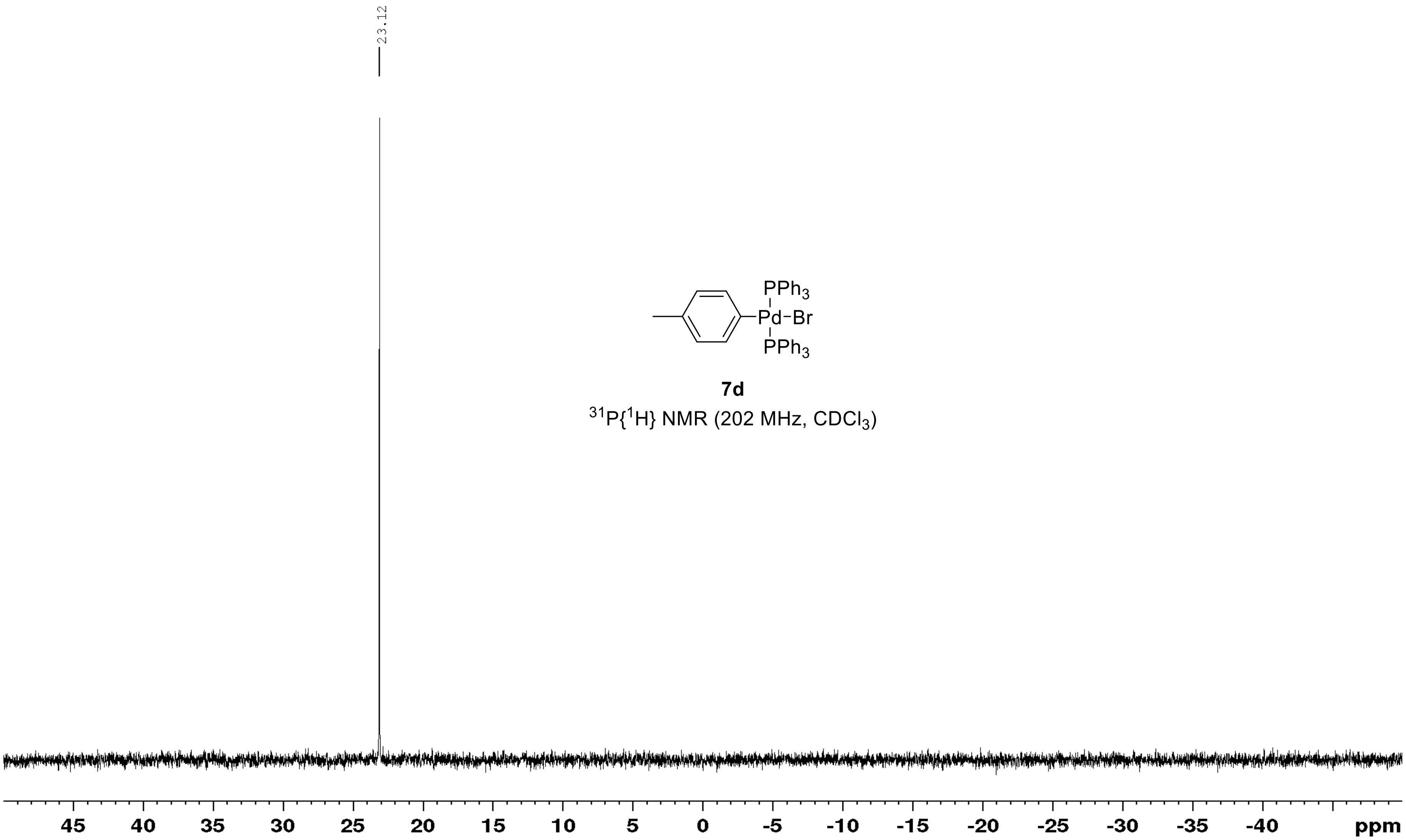

7d

$^{31}P\{^{1}H\}$ NMR (202 MHz, $CDCl_3$)

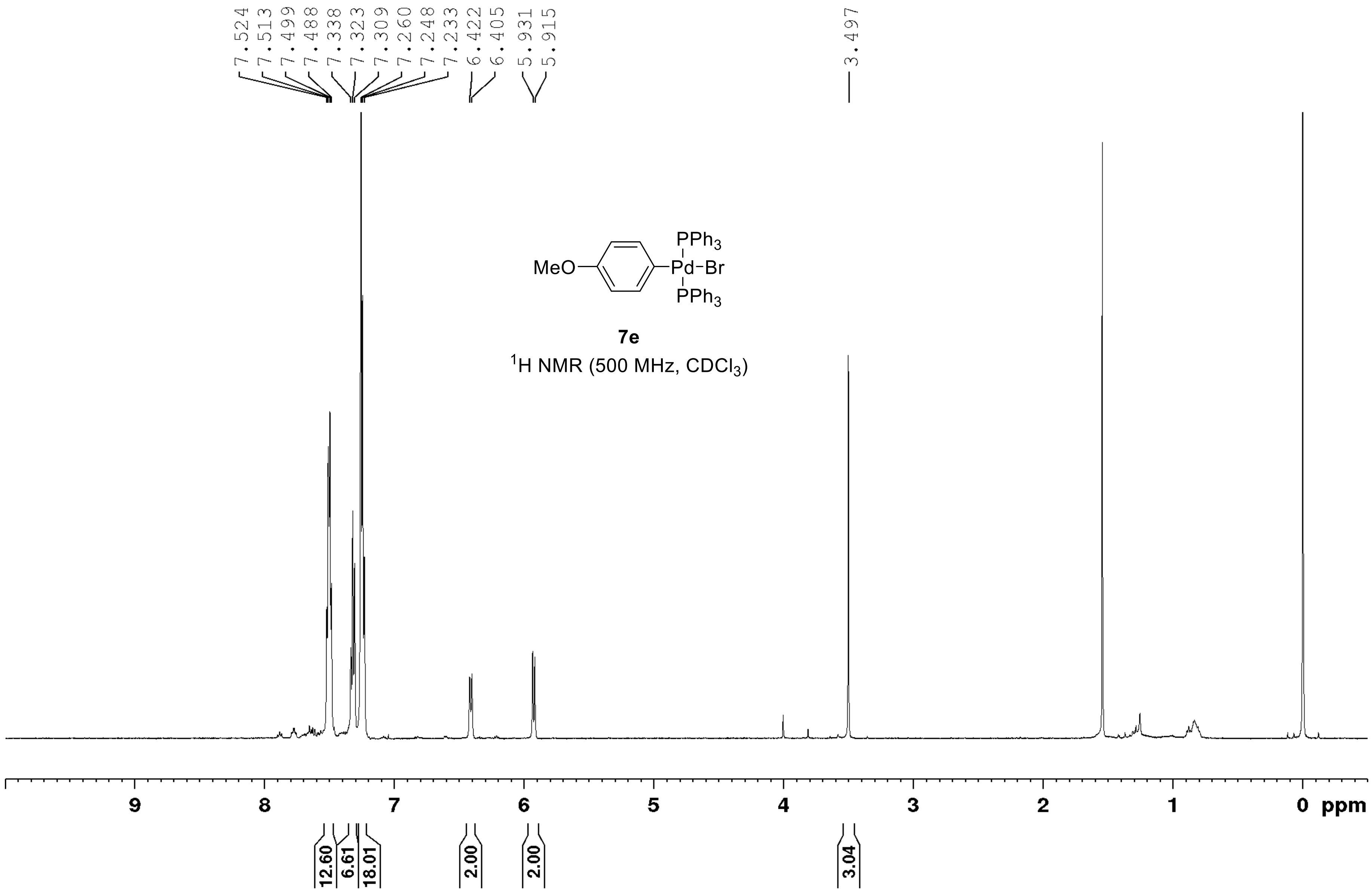
7.524
7.513
7.499
7.488
7.338
7.323
7.309
7.260
7.248
7.233
6.422
6.405
5.931
5.915
3.497
MeO
PPh3
Pd
Br
PPh3
7e
1H NMR (500 MHz, CDCl3)
9
8
7
6
5
4
3
2
1
0 ppm
12.60
6.61
18.01
2.00
2.00
3.04

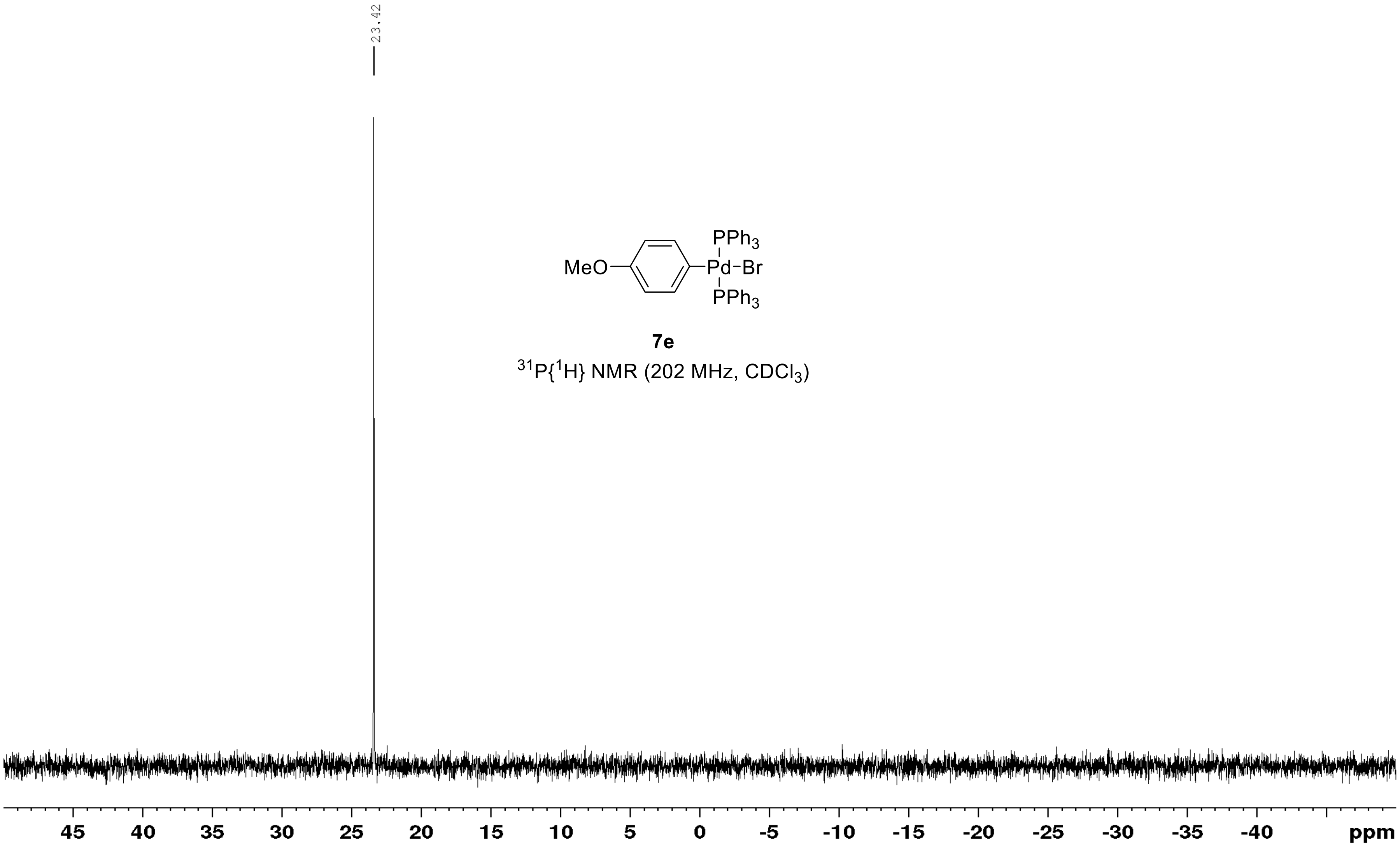

7e

$^{31}P\{^{1}H\}$ NMR (202 MHz, $CDCl_3$)

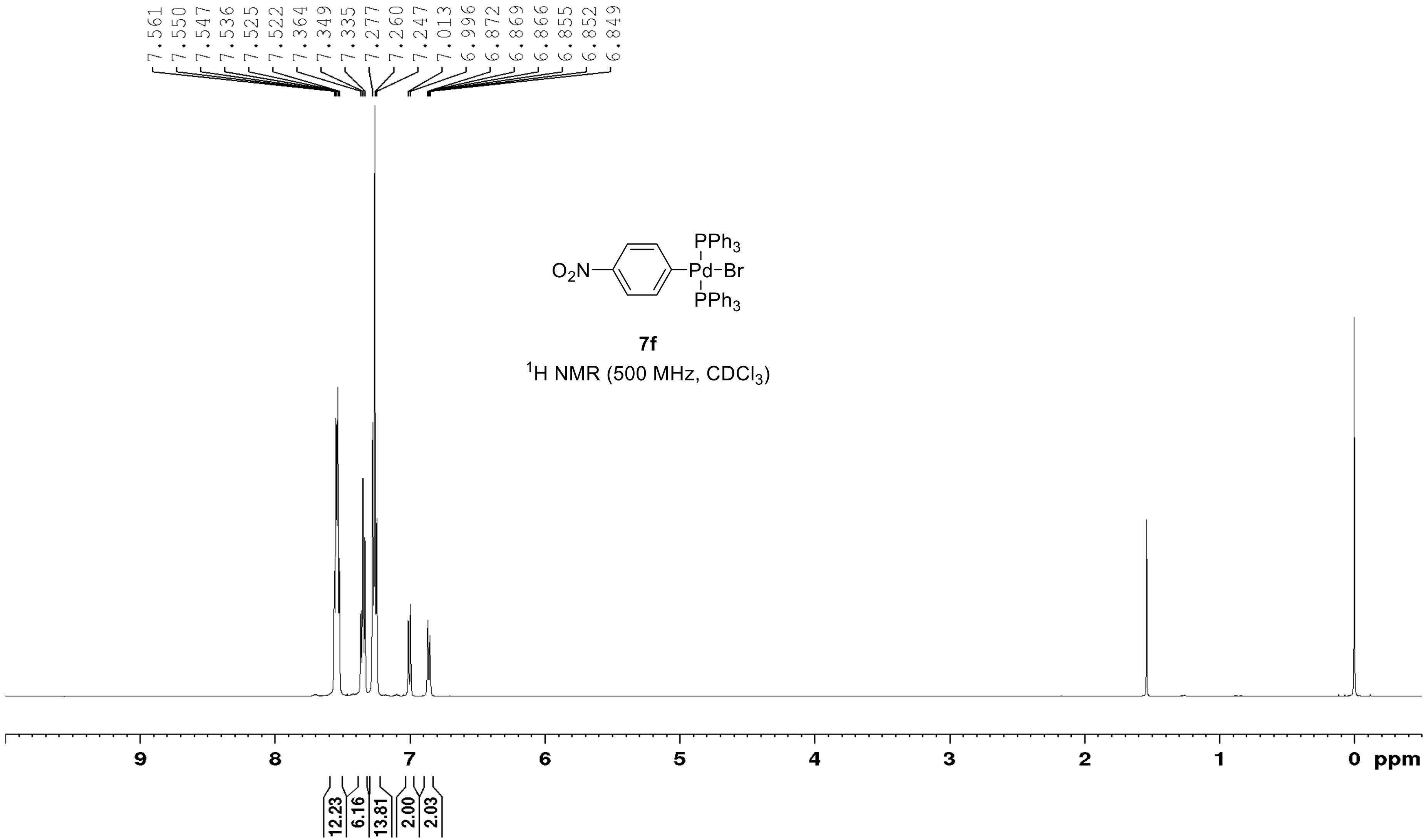

7f

^{1}H NMR (500 MHz, $CDCl_3$)

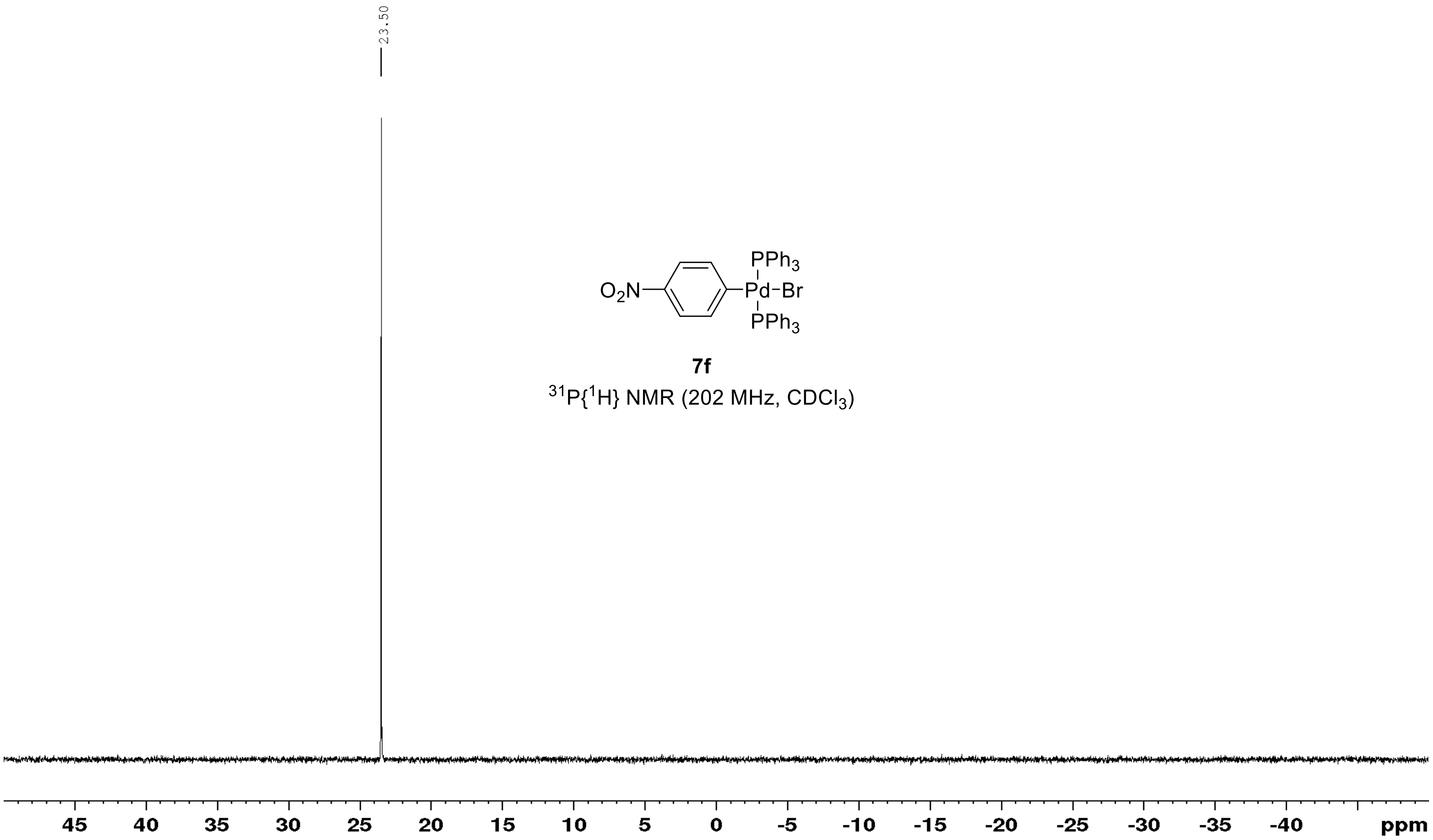
23.50
O_2N
PPh_3
Pd
Br
PPh_3
7f
$^{31}P\{^1H\}$ NMR (202 MHz, $CDCl_3$)
45
40
35
30
25
20
15
10
5
0
-5
-10
-15
-20
-25
-30
-35
-40
ppm

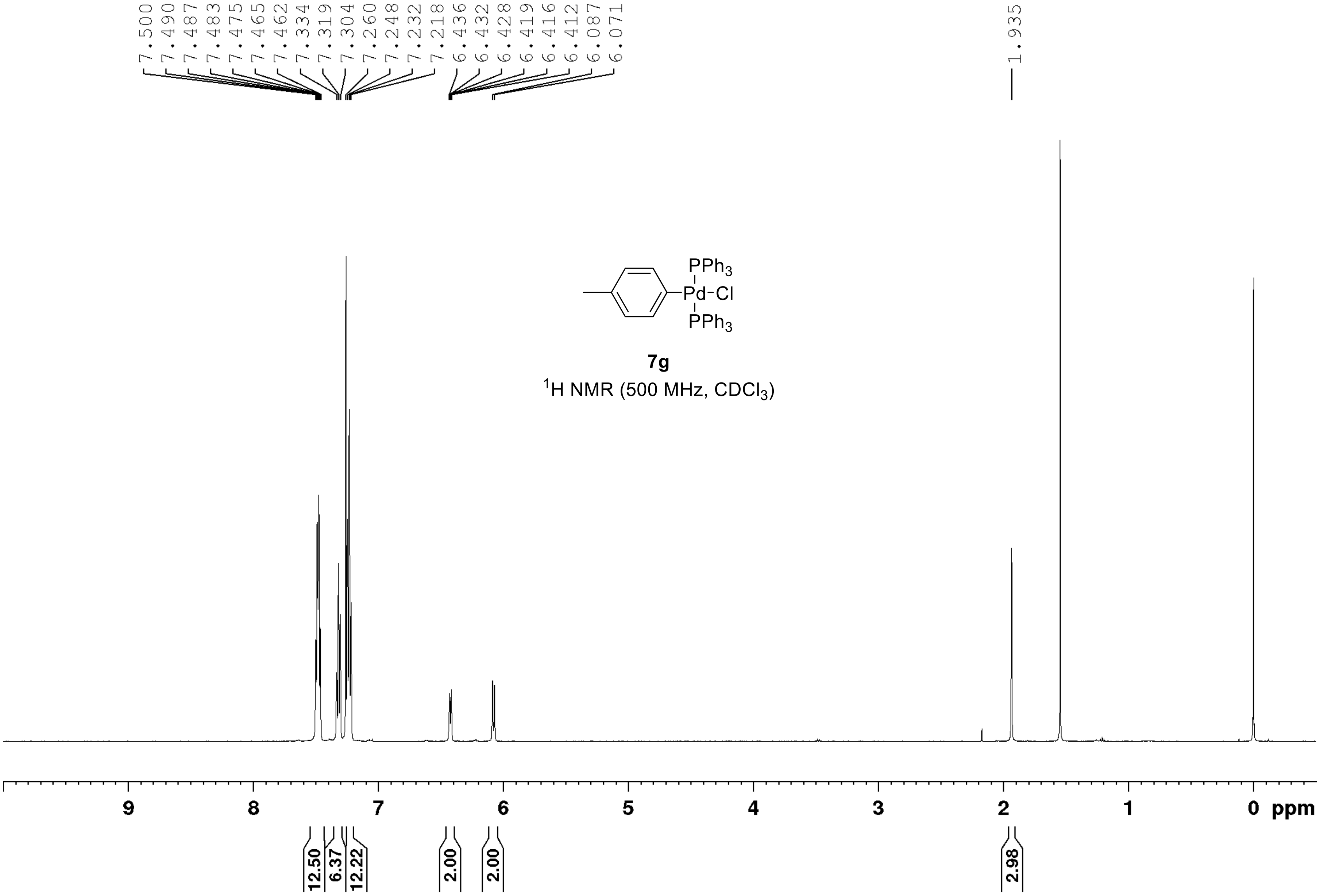

7g

^{1}H NMR (500 MHz, $CDCl_3$)

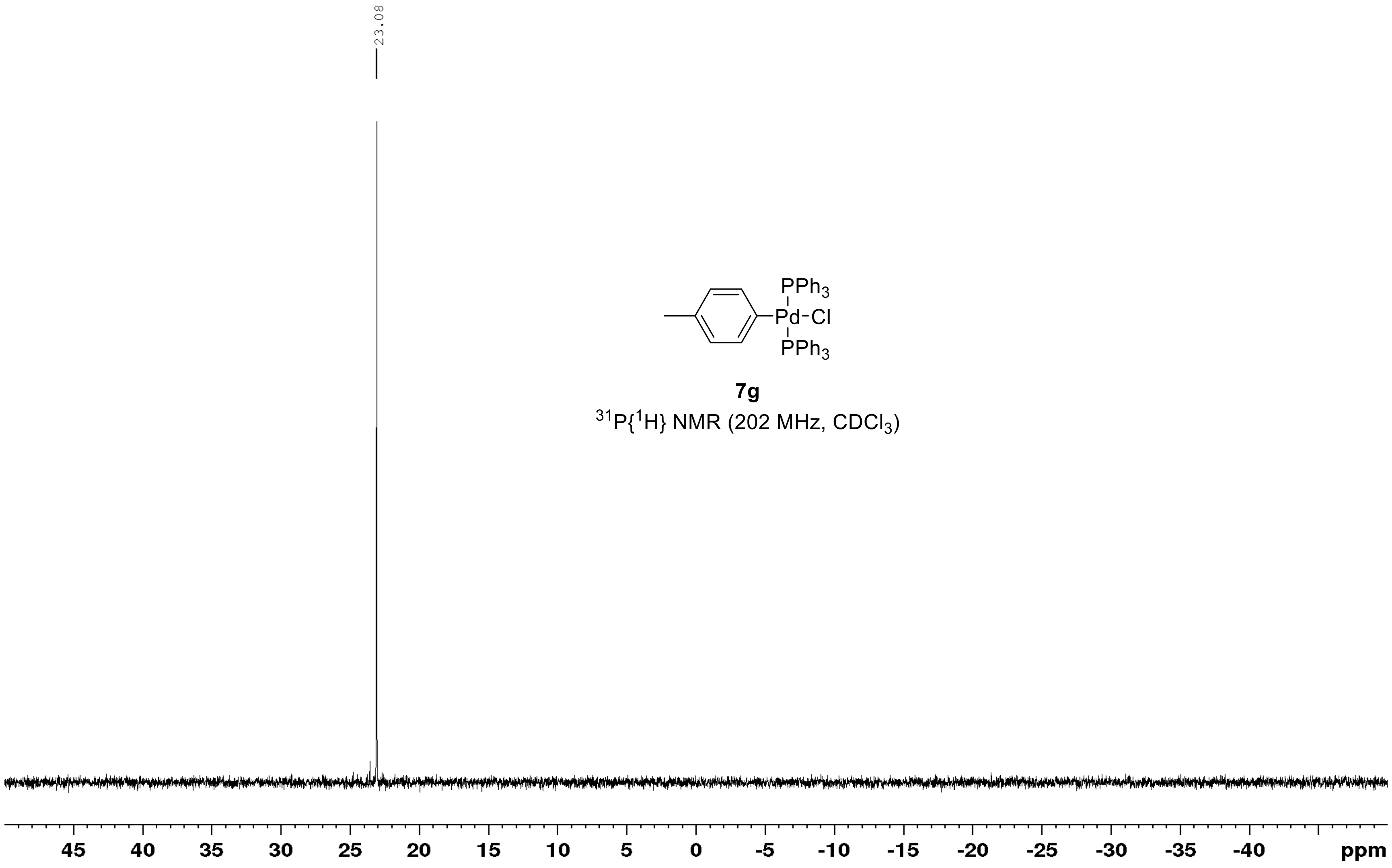

7g

$^{31}P\{^{1}H\}$ NMR (202 MHz, $CDCl_3$)

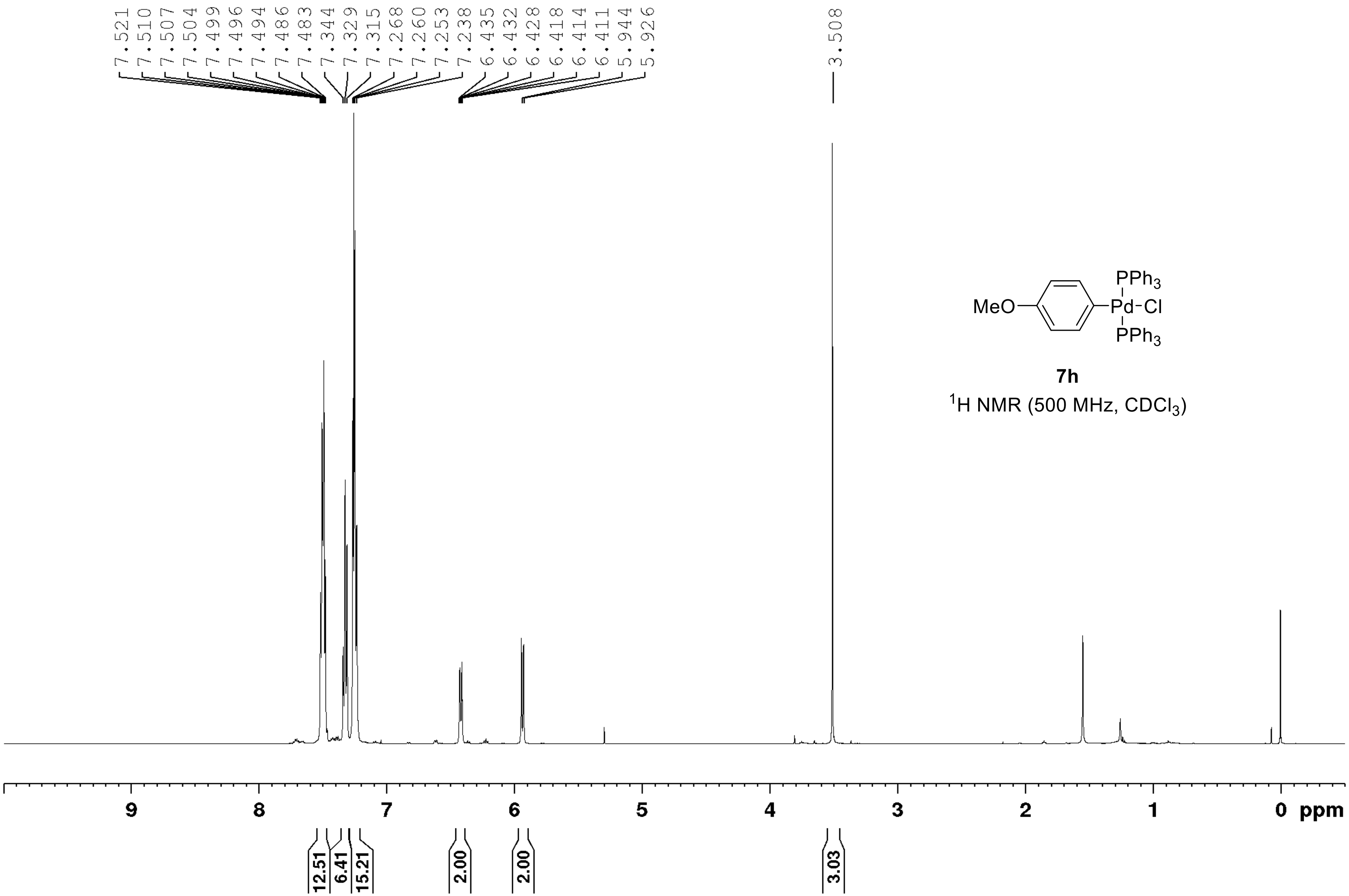
7.521
7.510
7.507
7.504
7.499
7.496
7.494
7.486
7.483
7.344
7.329
7.315
7.268
7.260
7.253
7.238
6.435
6.432
6.428
6.418
6.414
6.411
5.944
5.926
3.508
MeO
PPh3
Pd
Cl
PPh3
7h
1H NMR (500 MHz, CDCl3)
9
8
7
6
5
4
3
2
1
0
ppm
12.51
6.41
15.21
2.00
2.00
3.03

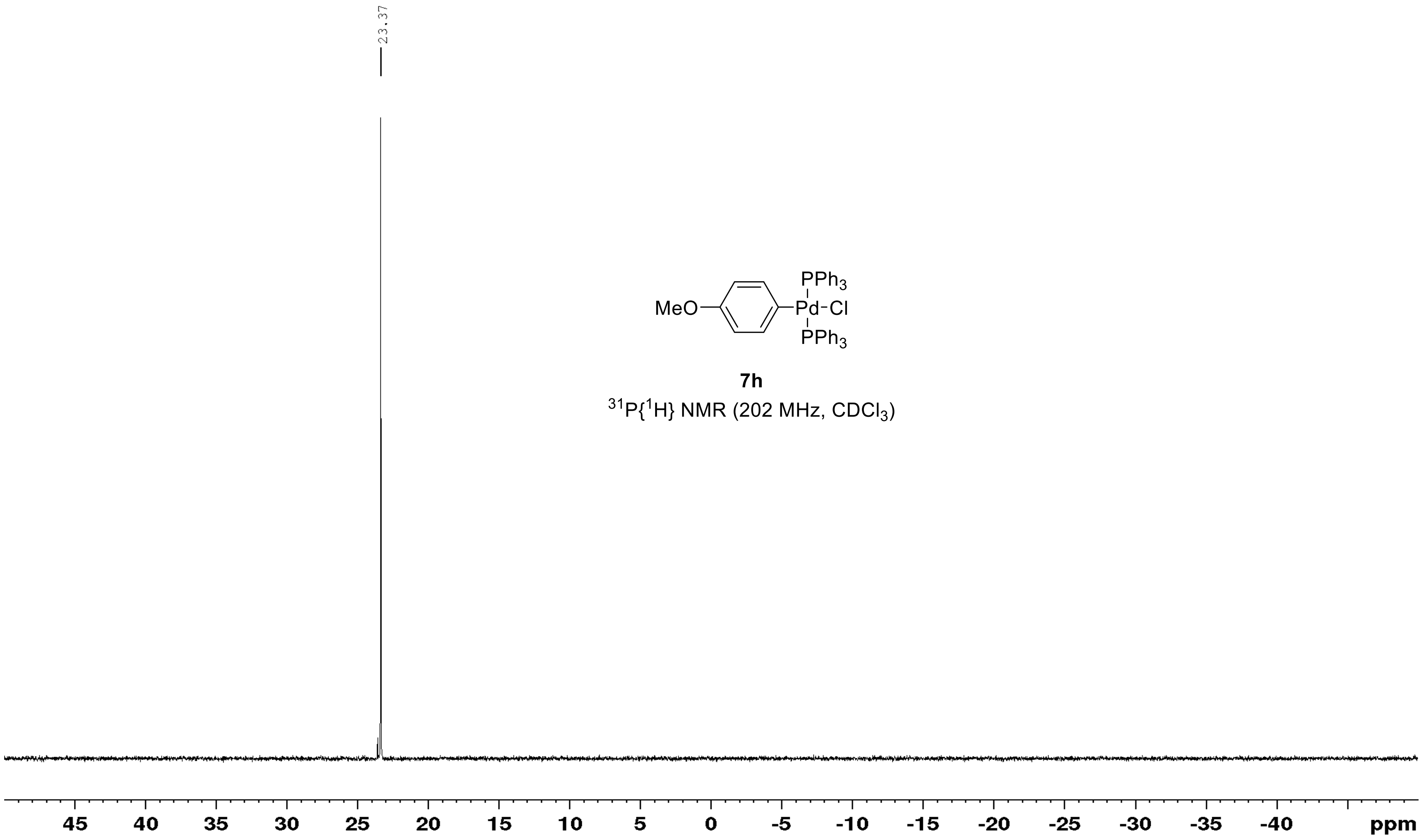

7h

$^{31}P\{^{1}H\}$ NMR (202 MHz, $CDCl_3$)

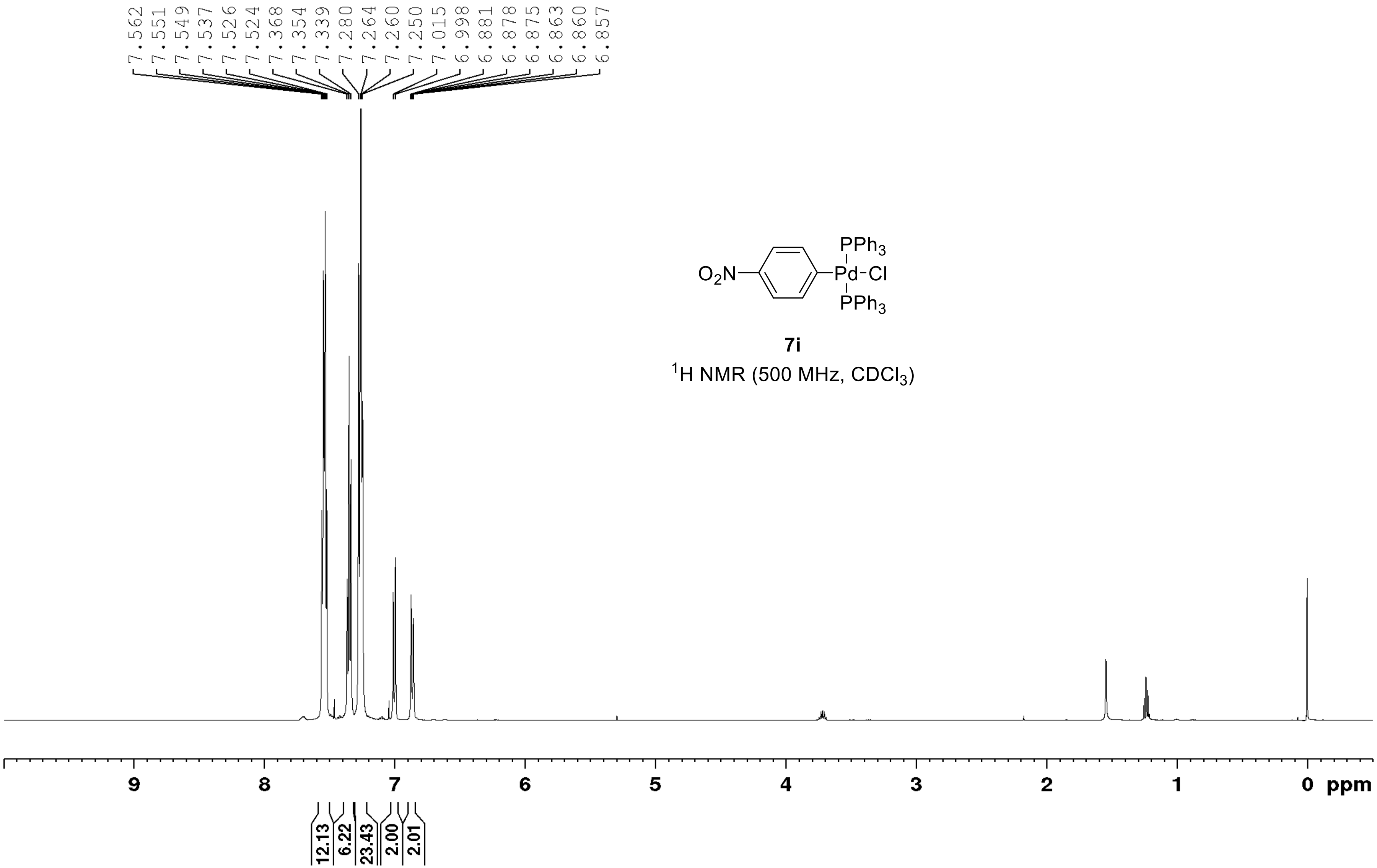
7.562
7.551
7.549
7.537
7.526
7.524
7.368
7.354
7.339
7.280
7.264
7.260
7.260
7.250
7.015
6.998
6.881
6.878
6.875
6.863
6.860
6.857
PPh3
O2N
Pd-Cl
PPh3
7i
1H NMR (500 MHz, CDCl3)
9
8
7
6
5
4
3
2
1
0 ppm
12.13
6.22
23.43
2.00
2.01

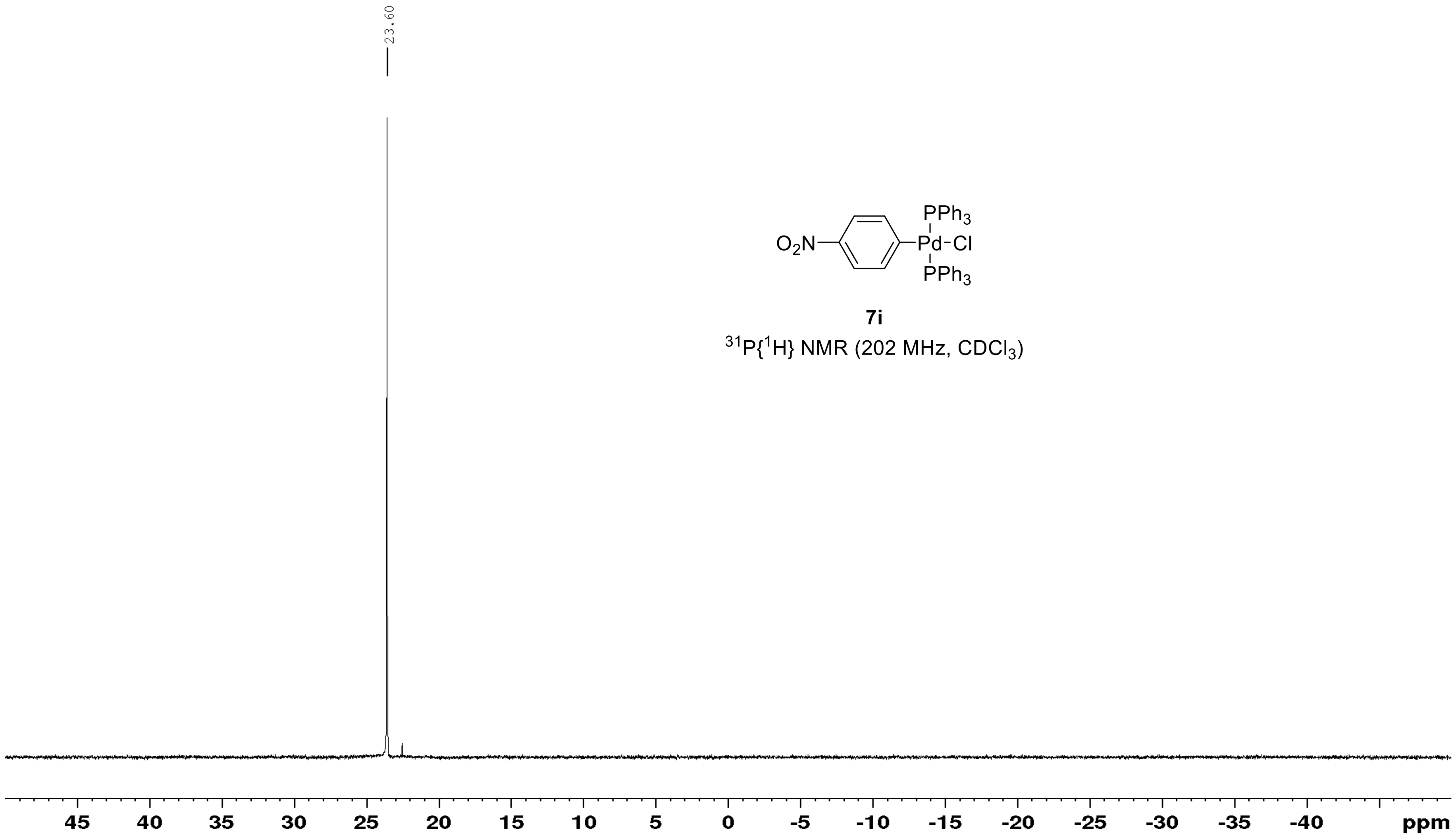
23.60
O_2N
PPh_3
Pd
Cl
PPh_3
7i
$^{31}P\{^1H\}$ NMR (202 MHz, $CDCl_3$)
45
40
35
30
25
20
15
10
5
0
-5
-10
-15
-20
-25
-30
-35
-40
ppm

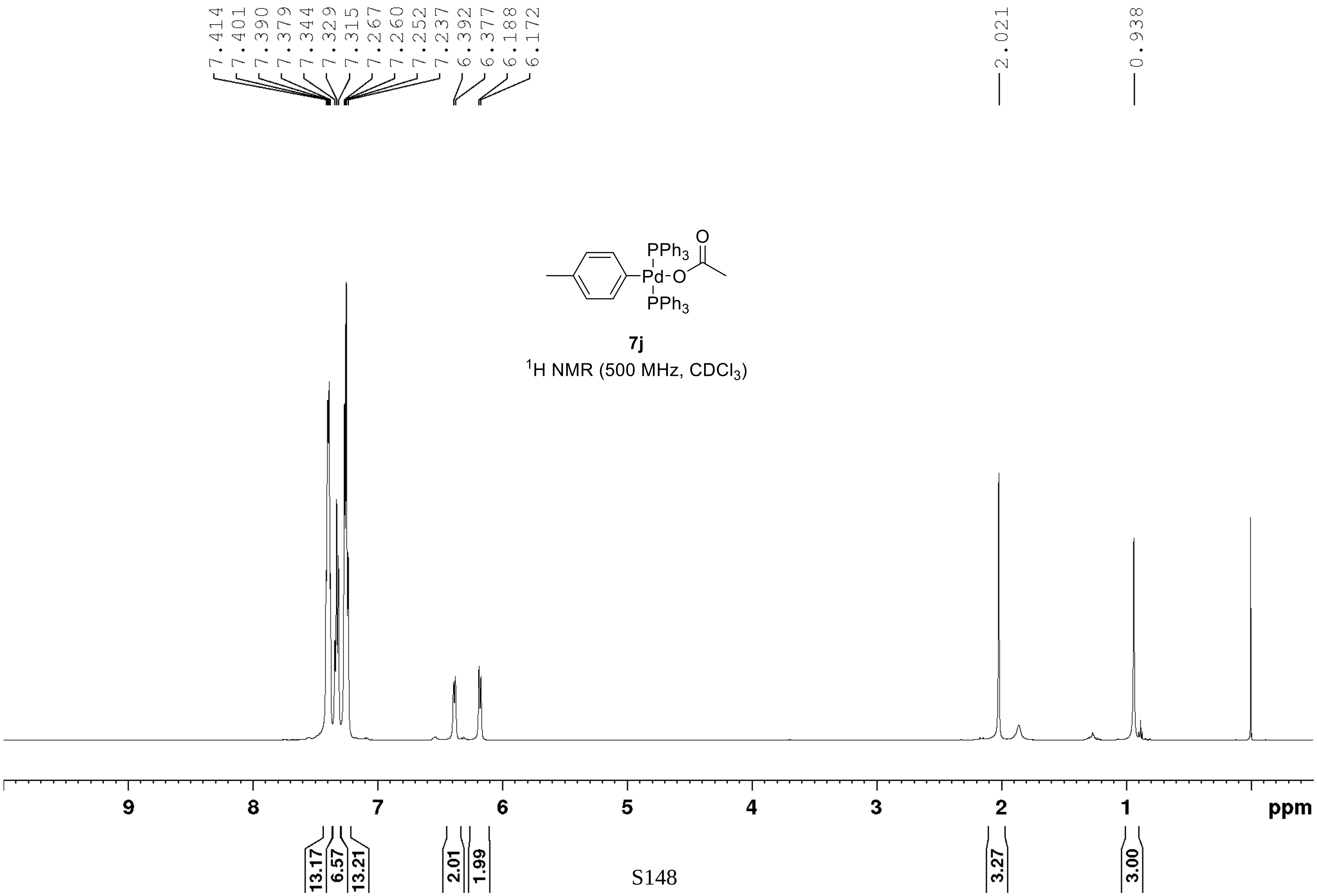

7j

^{1}H NMR (500 MHz, $CDCl_3$)

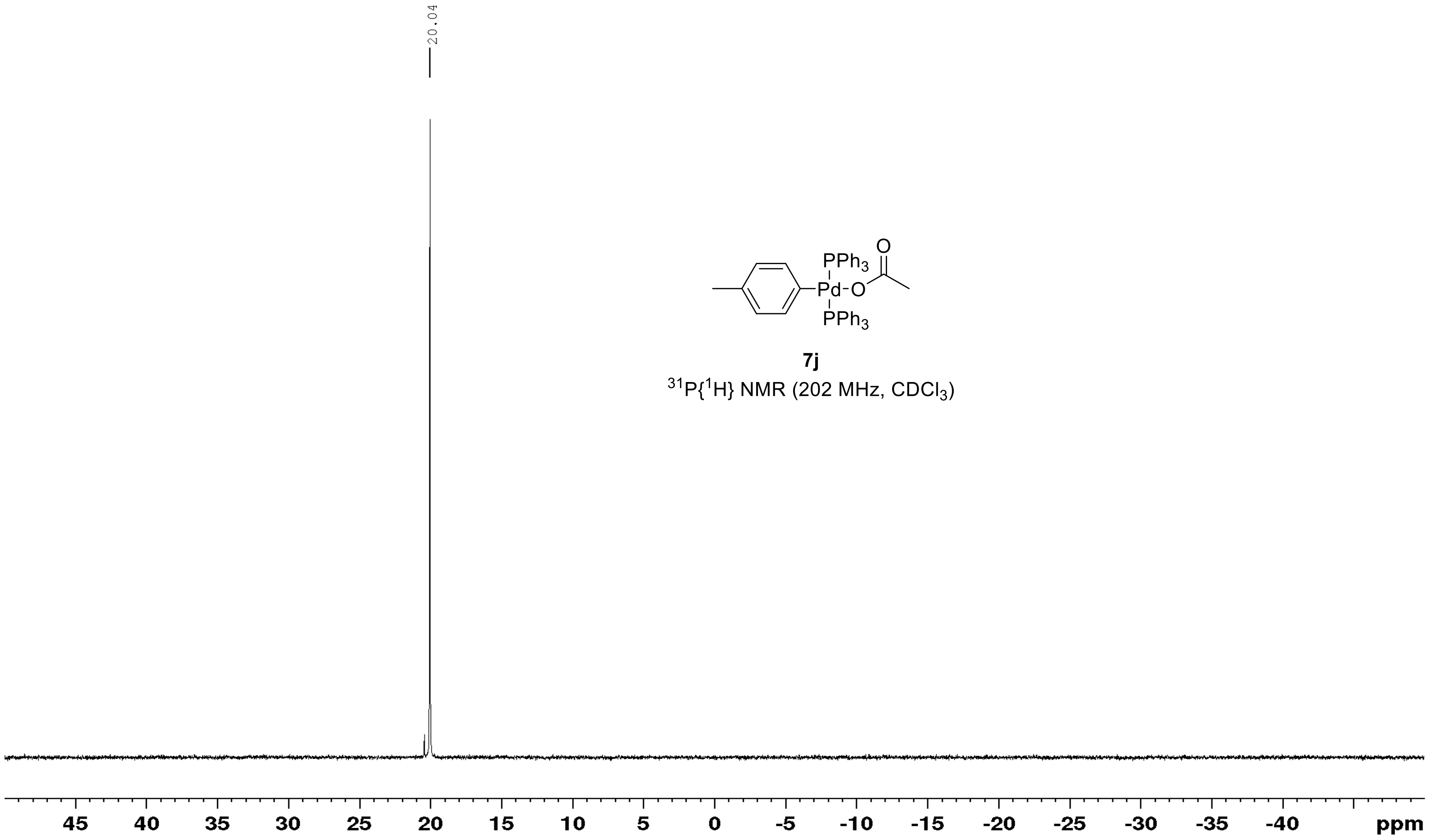
20.04
PPh3
Pd
O
PPh3
O
7j
31P{1H} NMR (202 MHz, CDCl3)
45
40
35
30
25
20
15
10
5
0
-5
-10
-15
-20
-25
-30
-35
-40
ppm

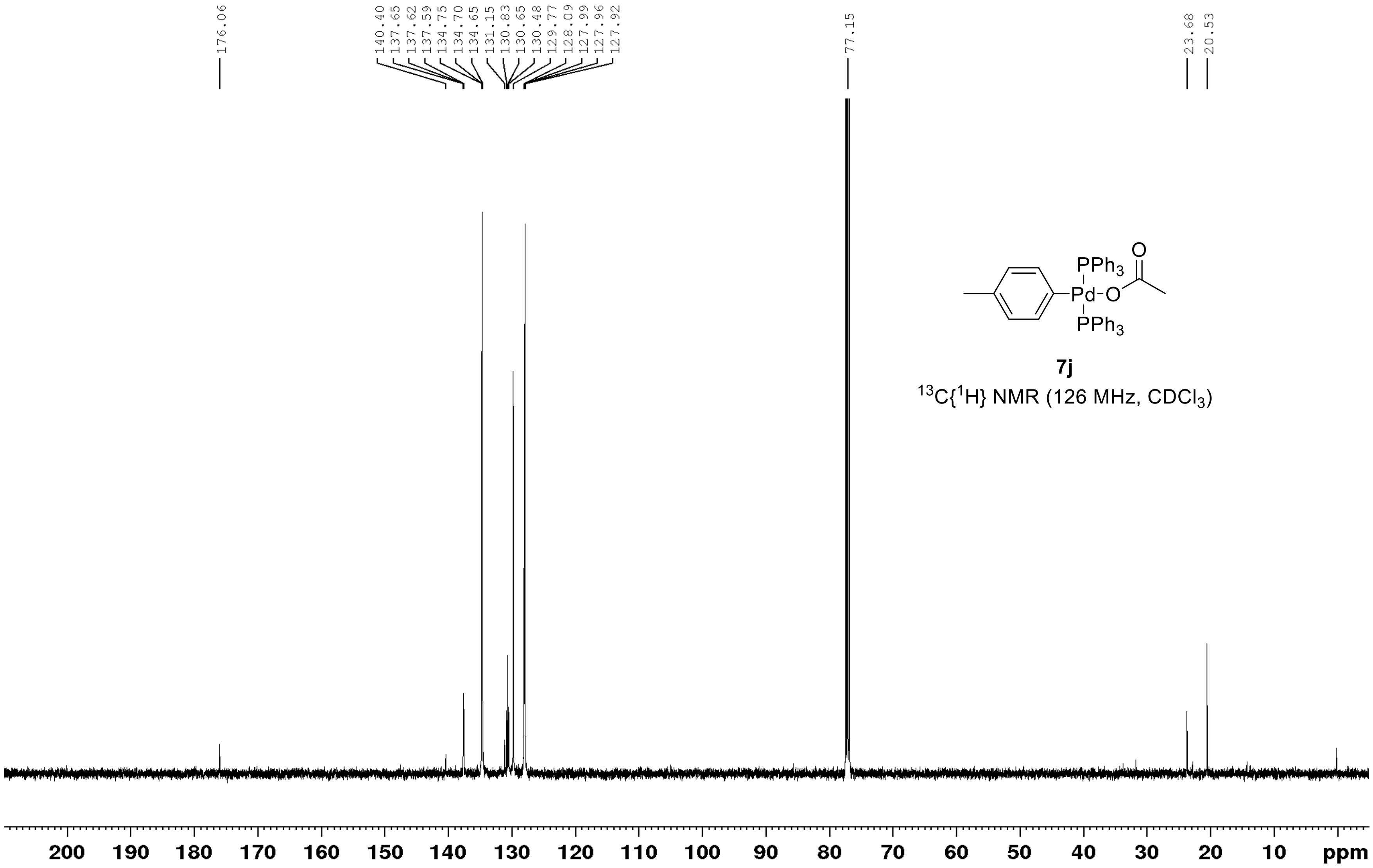

7j

$^{13}C\{^{1}H\}$ NMR (126 MHz, $CDCl_3$)

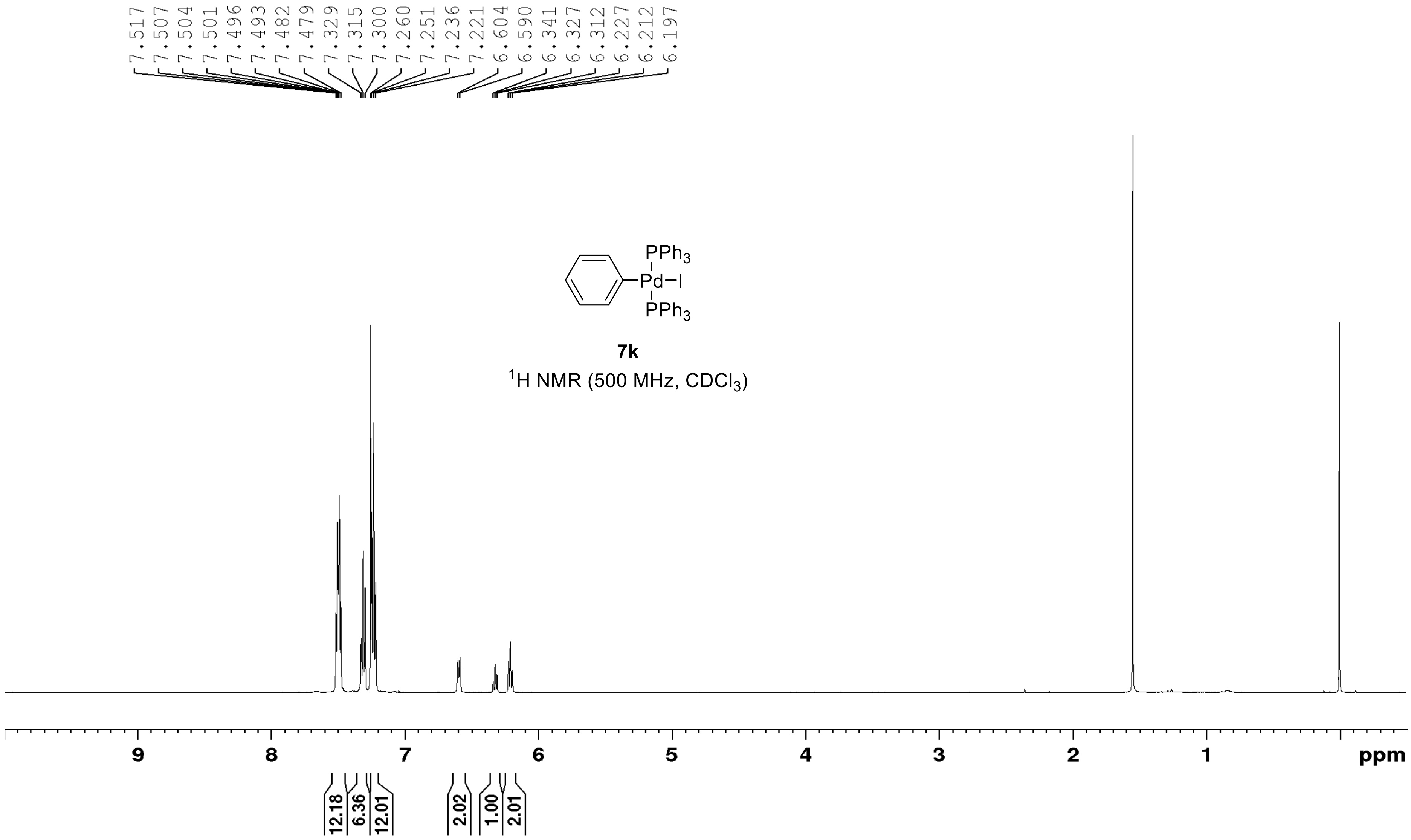
7.517
7.507
7.504
7.501
7.496
7.493
7.482
7.479
7.329
7.315
7.300
7.260
7.251
7.236
7.221
6.604
6.590
6.341
6.327
6.312
6.227
6.212
6.197
PPh3
Pd–I
PPh3
7k
1H NMR (500 MHz, CDCl3)
9
8
7
6
5
4
3
2
1
ppm
12.18
6.36
12.01
2.02
1.00
2.01

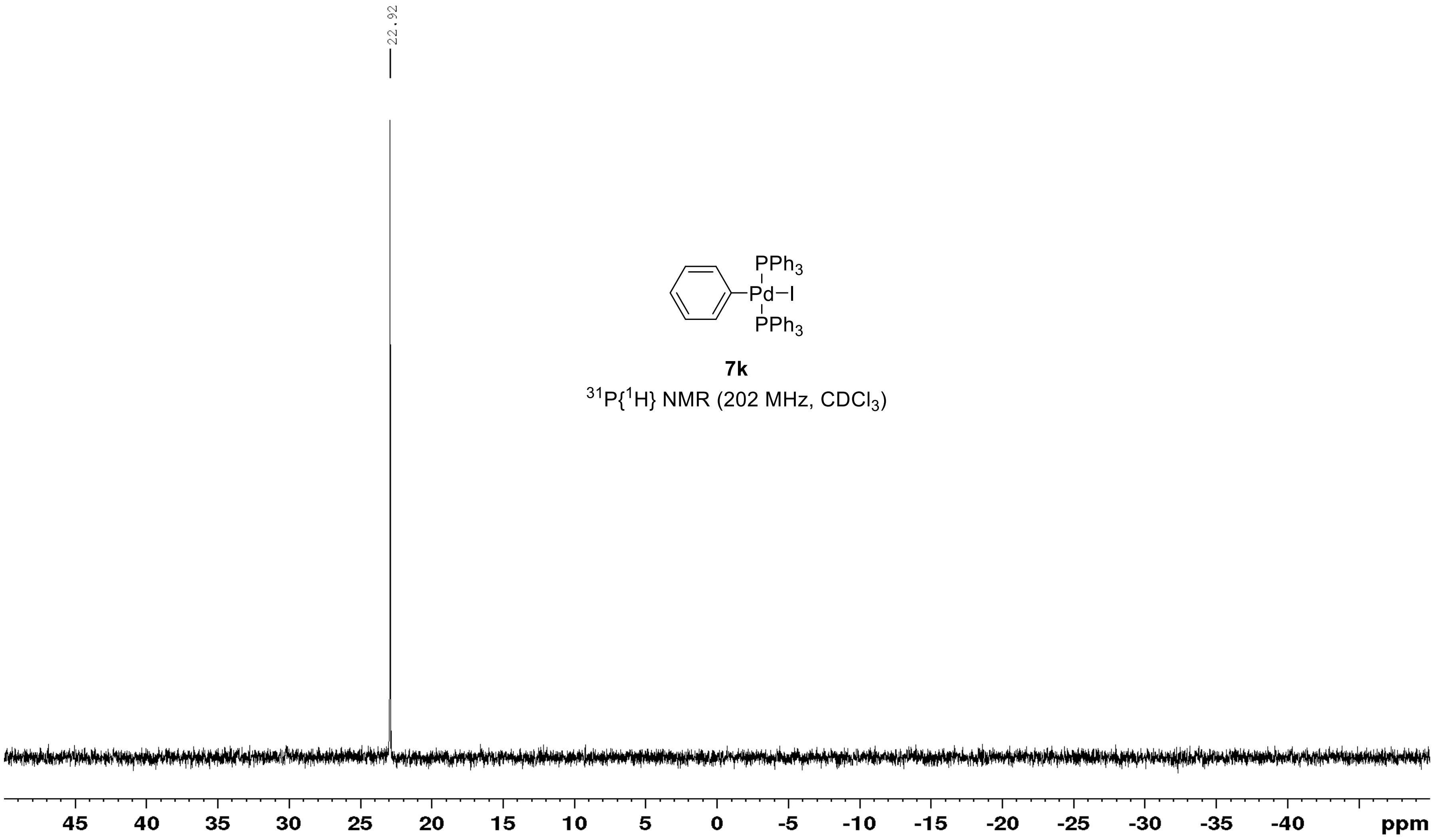

7k

$^{31}P\{^{1}H\}$ NMR (202 MHz, $CDCl_3$)

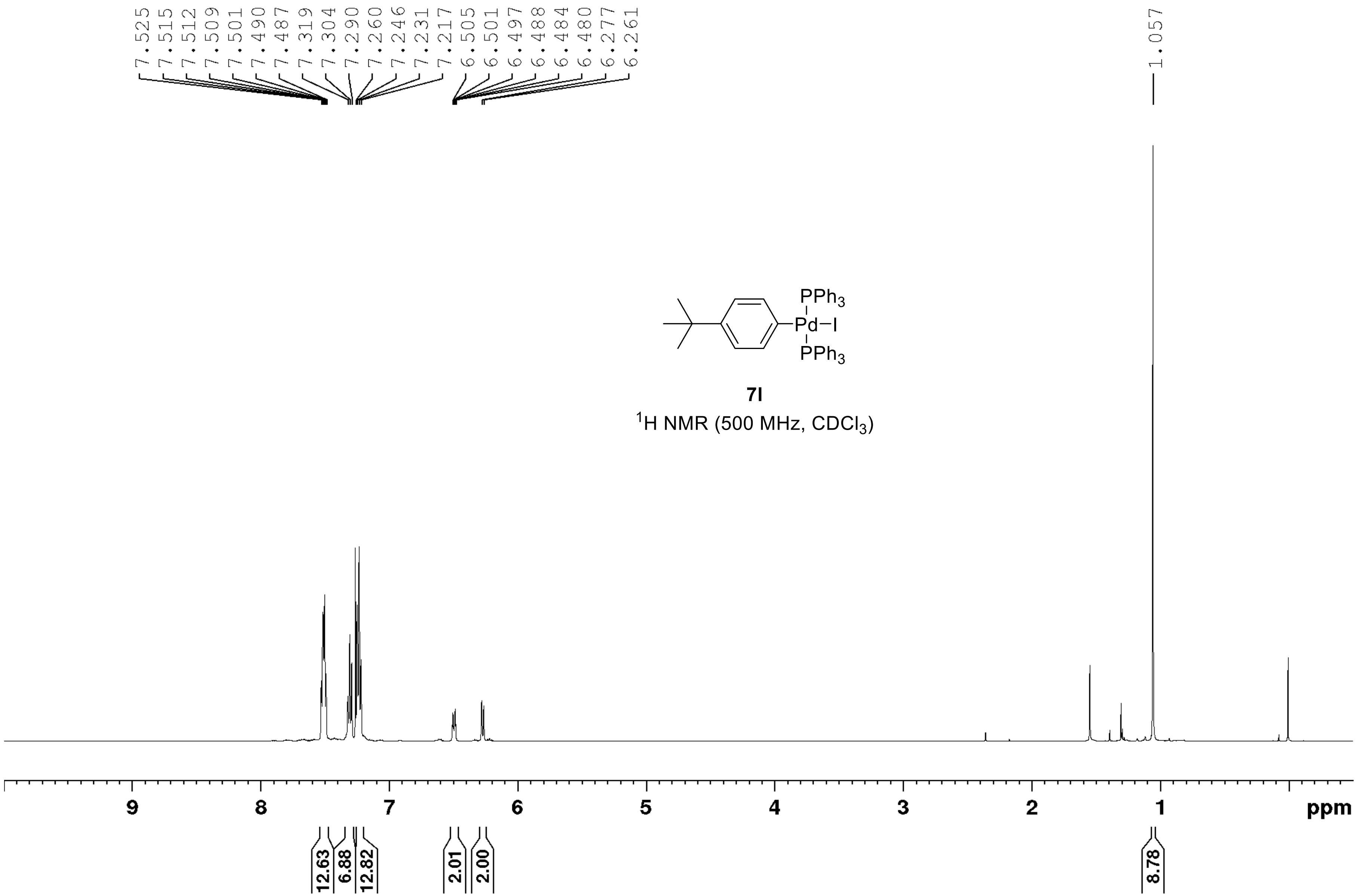

7l

^{1}H NMR (500 MHz, $CDCl_3$)

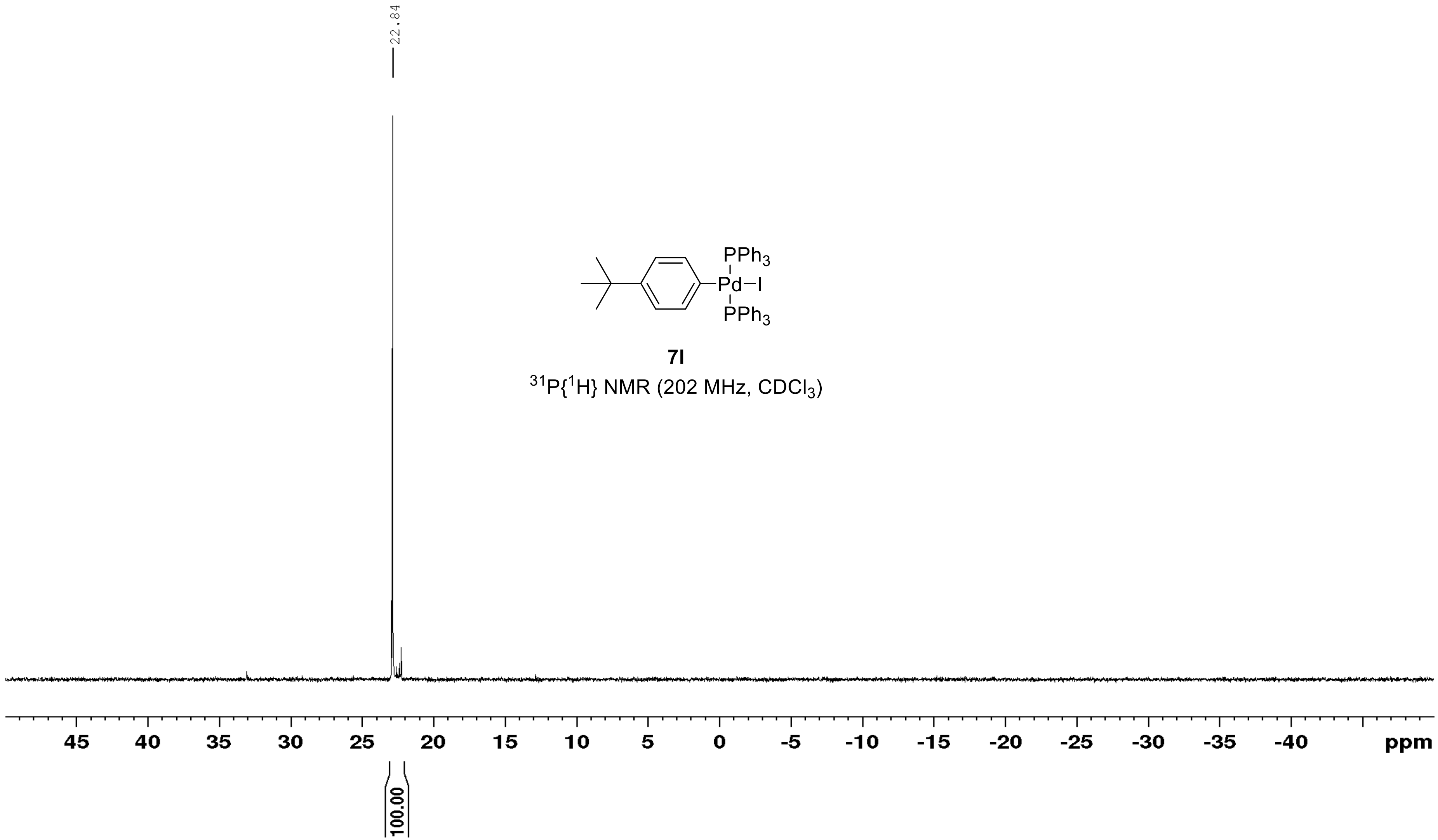

7l

$^{31}P\{^1H\}$ NMR (202 MHz, $CDCl_3$)

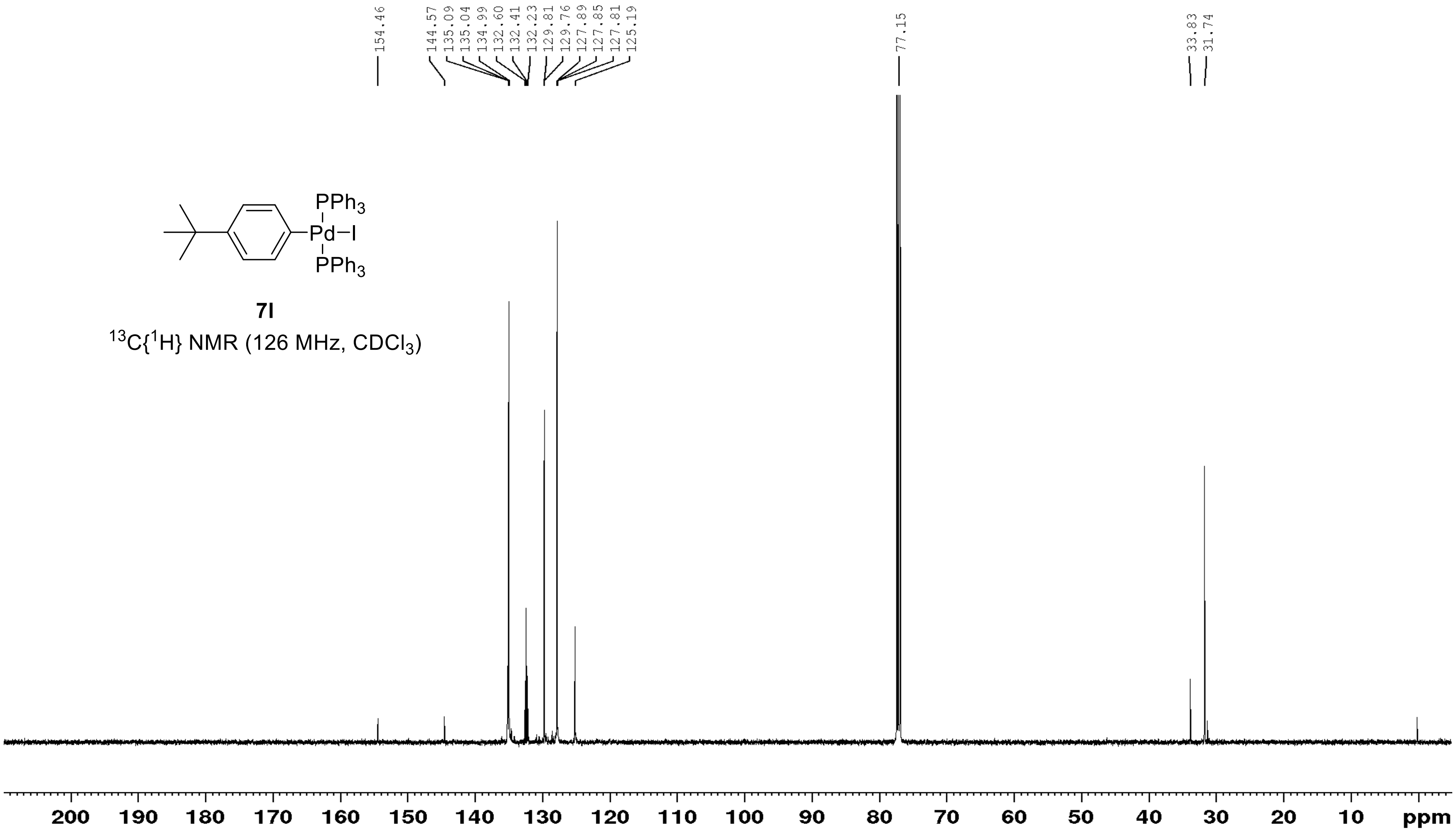

7l

$^{13}C\{^{1}H\}$ NMR (126 MHz, $CDCl_3$)

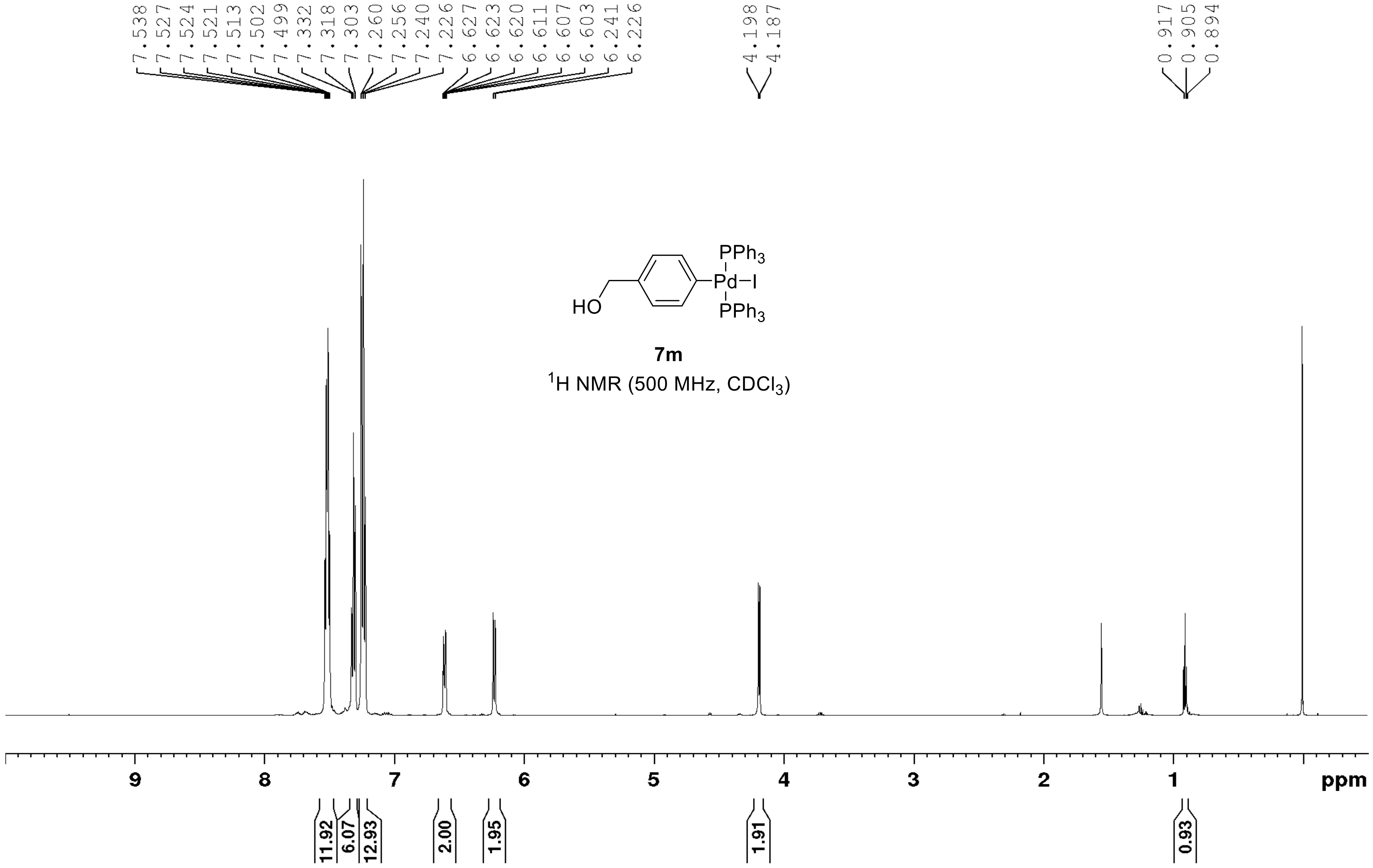
7.538
7.527
7.524
7.521
7.513
7.502
7.499
7.332
7.318
7.303
7.260
7.256
7.240
7.226
6.627
6.623
6.620
6.611
6.607
6.603
6.241
6.226
4.198
4.187
0.917
0.905
0.894
PPh3
Pd
I
PPh3
HO
7m
1H NMR (500 MHz, CDCl3)
9
8
7
6
5
4
3
2
1
ppm
11.92
6.07
12.93
2.00
1.95
1.91
0.93

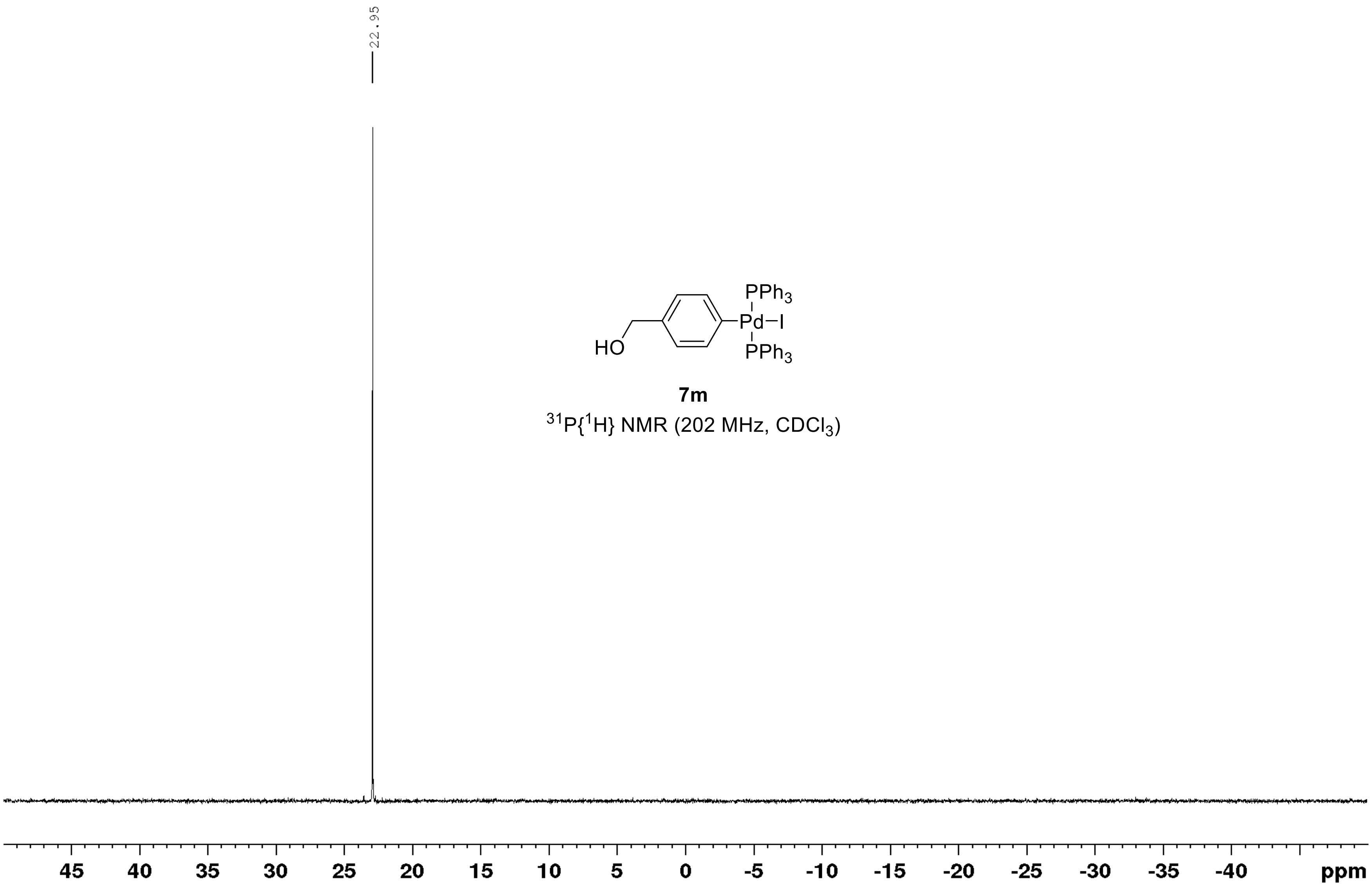

7m

$^{31}P\{^1H\}$ NMR (202 MHz, $CDCl_3$)

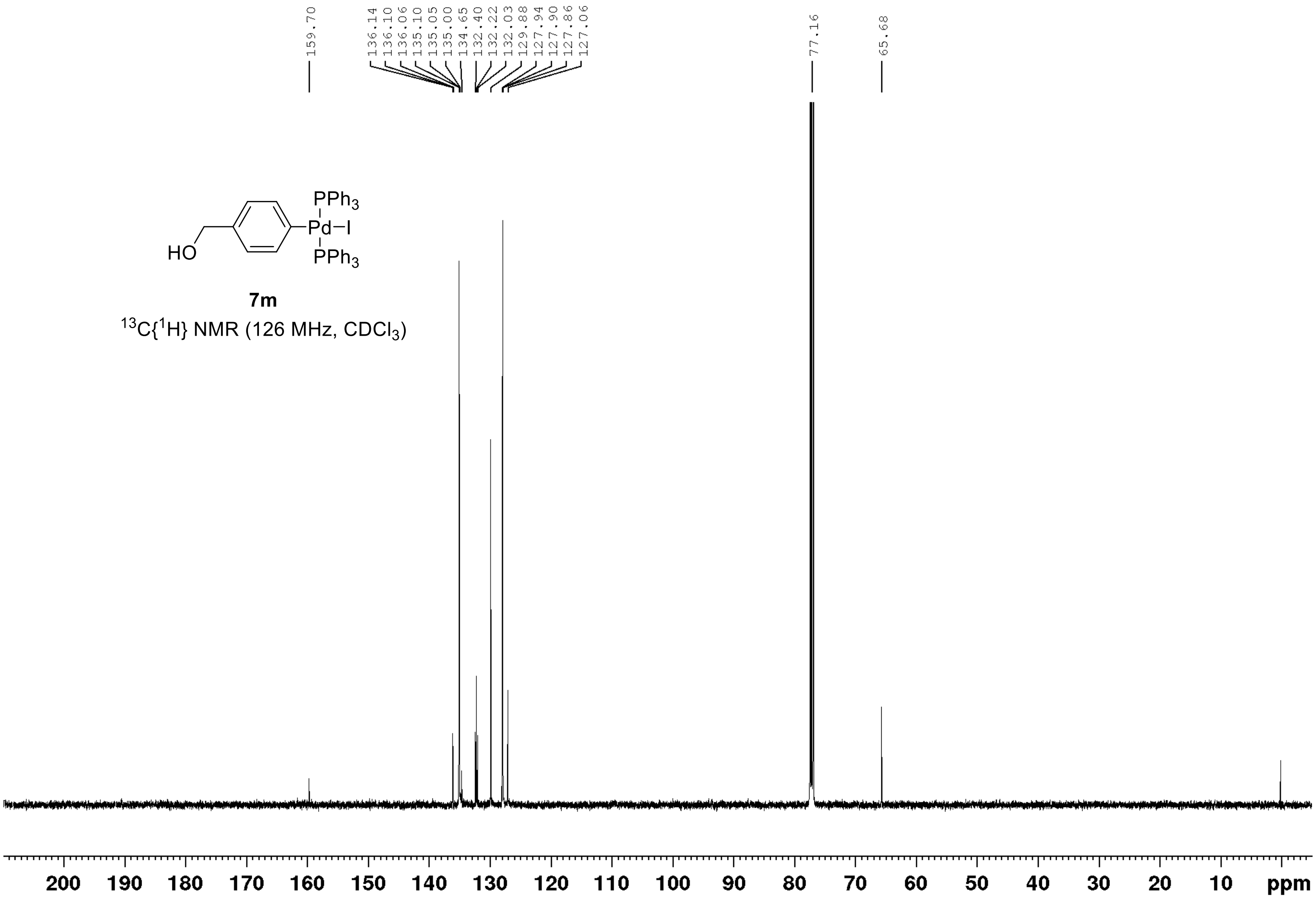
159.70
136.14
136.10
136.06
135.10
135.05
135.00
134.65
132.40
132.22
132.03
129.88
127.94
127.90
127.86
127.06
77.16
65.68
PPh3
Pd–I
PPh3
HO
7m
13C{1H} NMR (126 MHz, CDCl3)
200
190
180
170
160
150
140
130
120
110
100
90
80
70
60
50
40
30
20
10
ppm

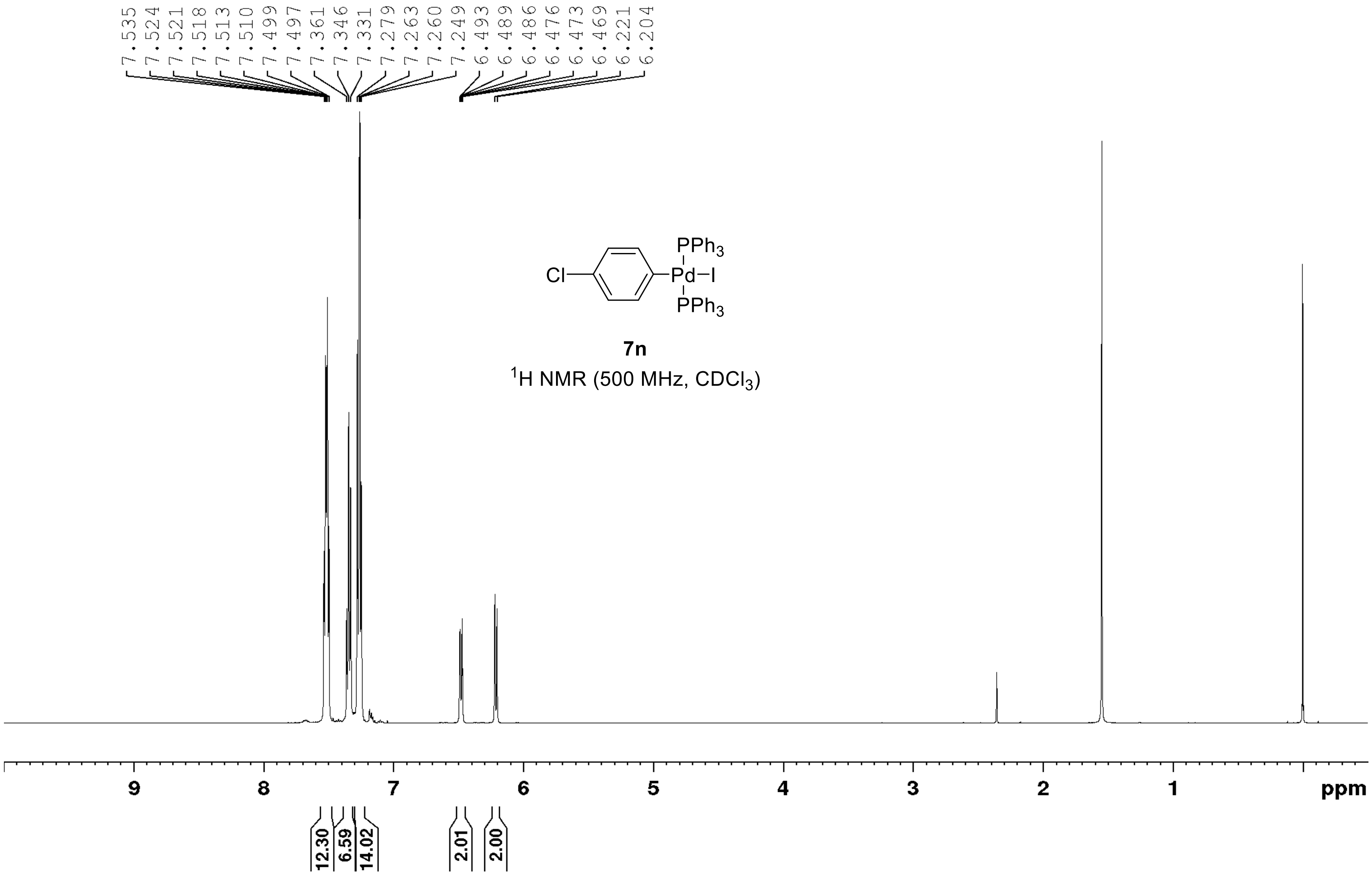
7.535
7.524
7.521
7.518
7.513
7.510
7.499
7.497
7.361
7.346
7.331
7.279
7.263
7.260
7.249
6.493
6.489
6.486
6.476
6.473
6.469
6.221
6.204
Cl
PPh3
Pd
I
PPh3
7n
1H NMR (500 MHz, CDCl3)
9
8
7
6
5
4
3
2
1
ppm
12.30
6.59
14.02
2.01
2.00

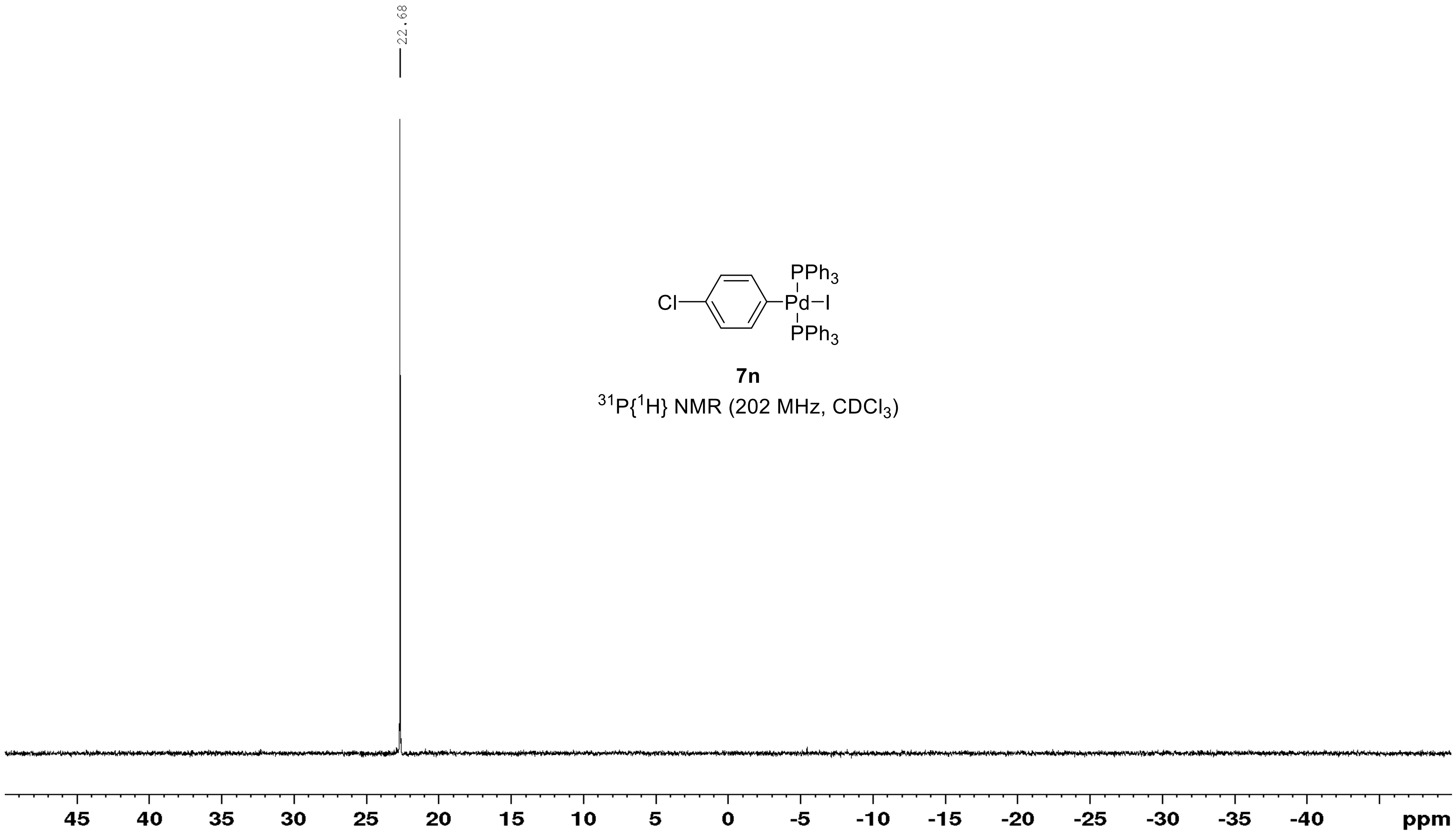

7n

$^{31}P\{^{1}H\}$ NMR (202 MHz, $CDCl_3$)

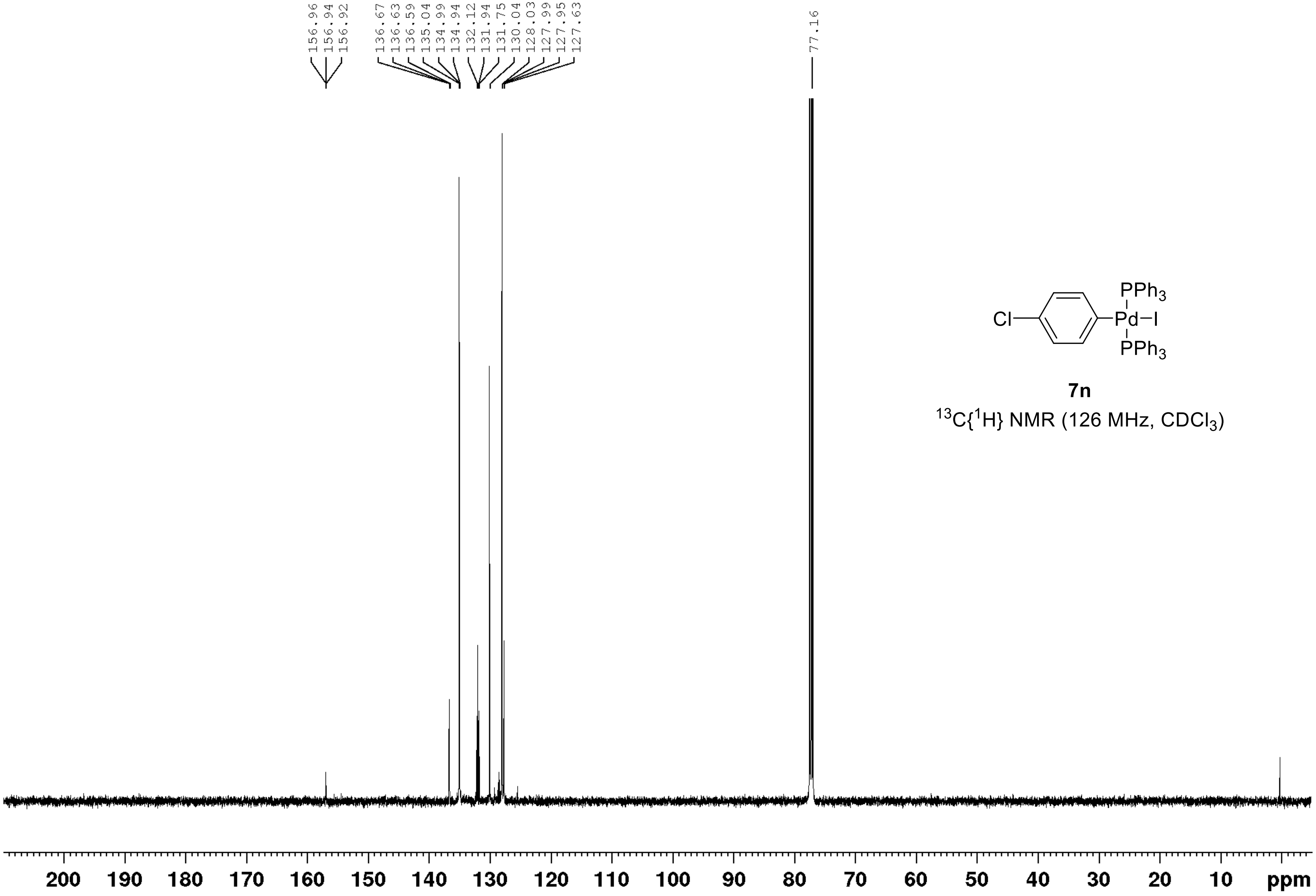

7n

$^{13}C\{^1H\}$ NMR (126 MHz, $CDCl_3$)

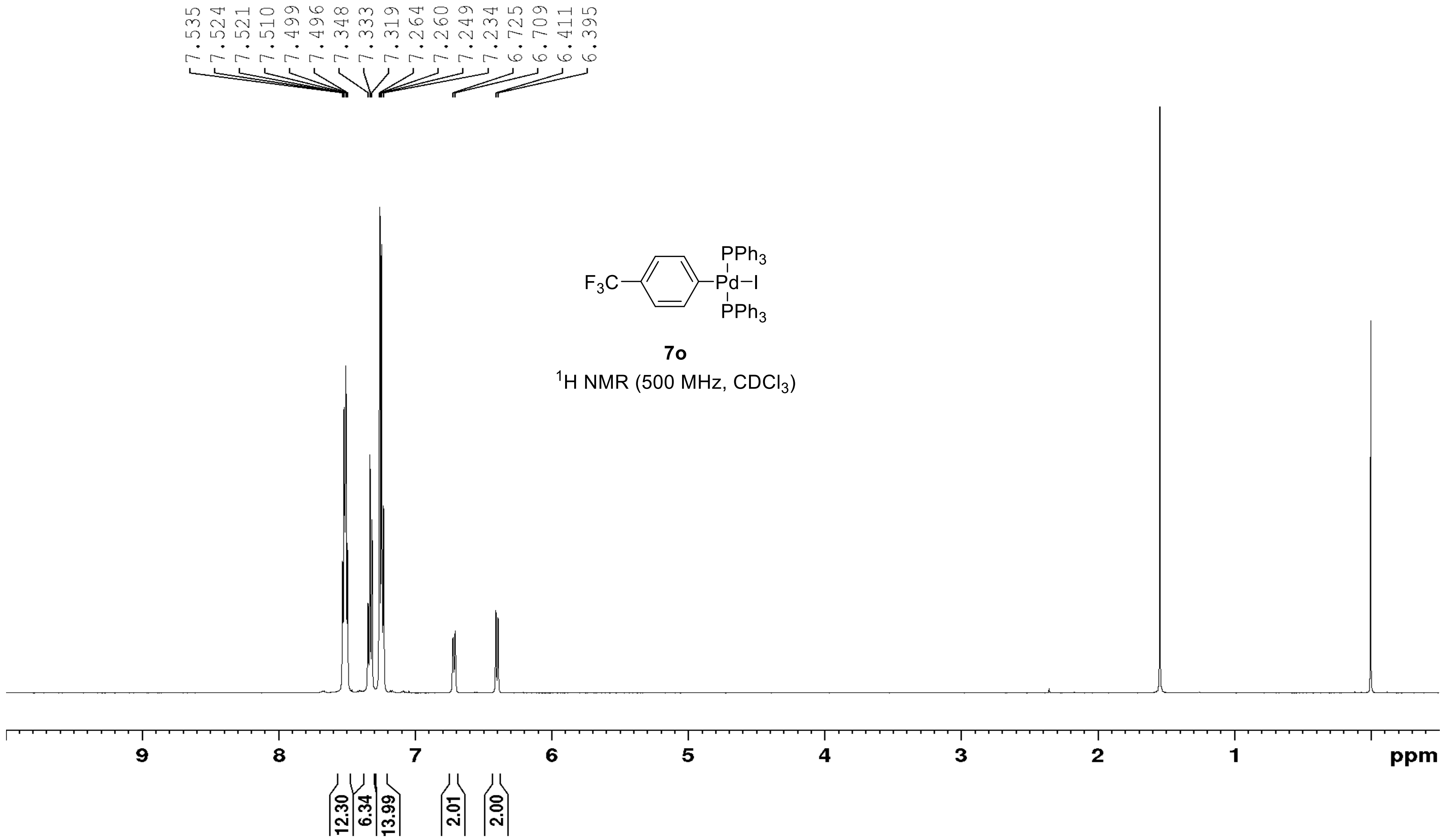
7.535
7.524
7.521
7.510
7.499
7.496
7.348
7.333
7.319
7.264
7.260
7.249
7.234
6.725
6.709
6.411
6.395
PPh3
F3C
Pd
I
PPh3
7o
1H NMR (500 MHz, CDCl3)
9
8
7
6
5
4
3
2
1
ppm
12.30
6.34
13.99
2.01
2.00

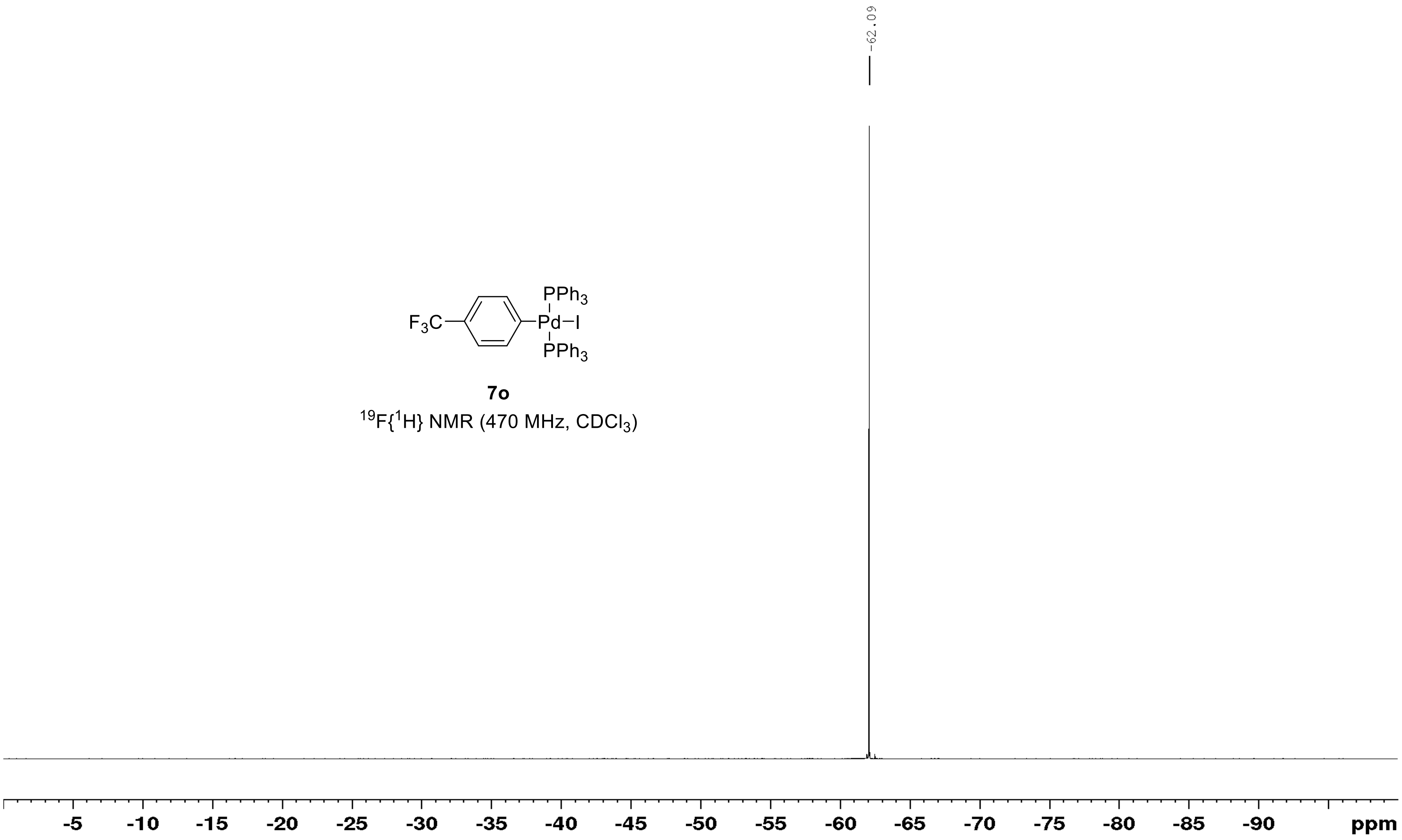
-62.09
PPh3
F3C
Pd
I
PPh3
7o
19F{1H} NMR (470 MHz, CDCl3)
-5
-10
-15
-20
-25
-30
-35
-40
-45
-50
-55
-60
-65
-70
-75
-80
-85
-90
ppm

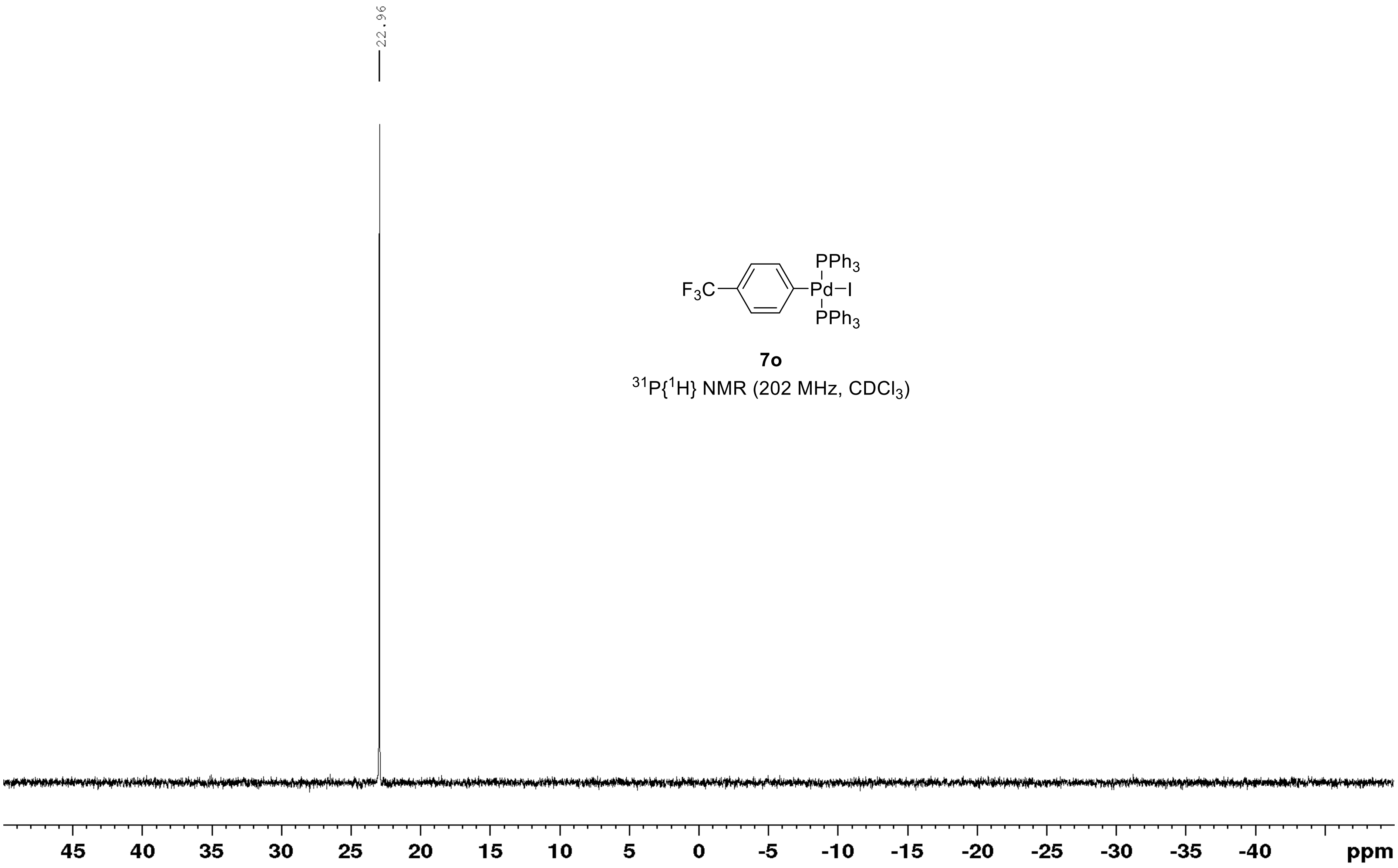

7o

$^{31}P\{^{1}H\}$ NMR (202 MHz, $CDCl_3$)

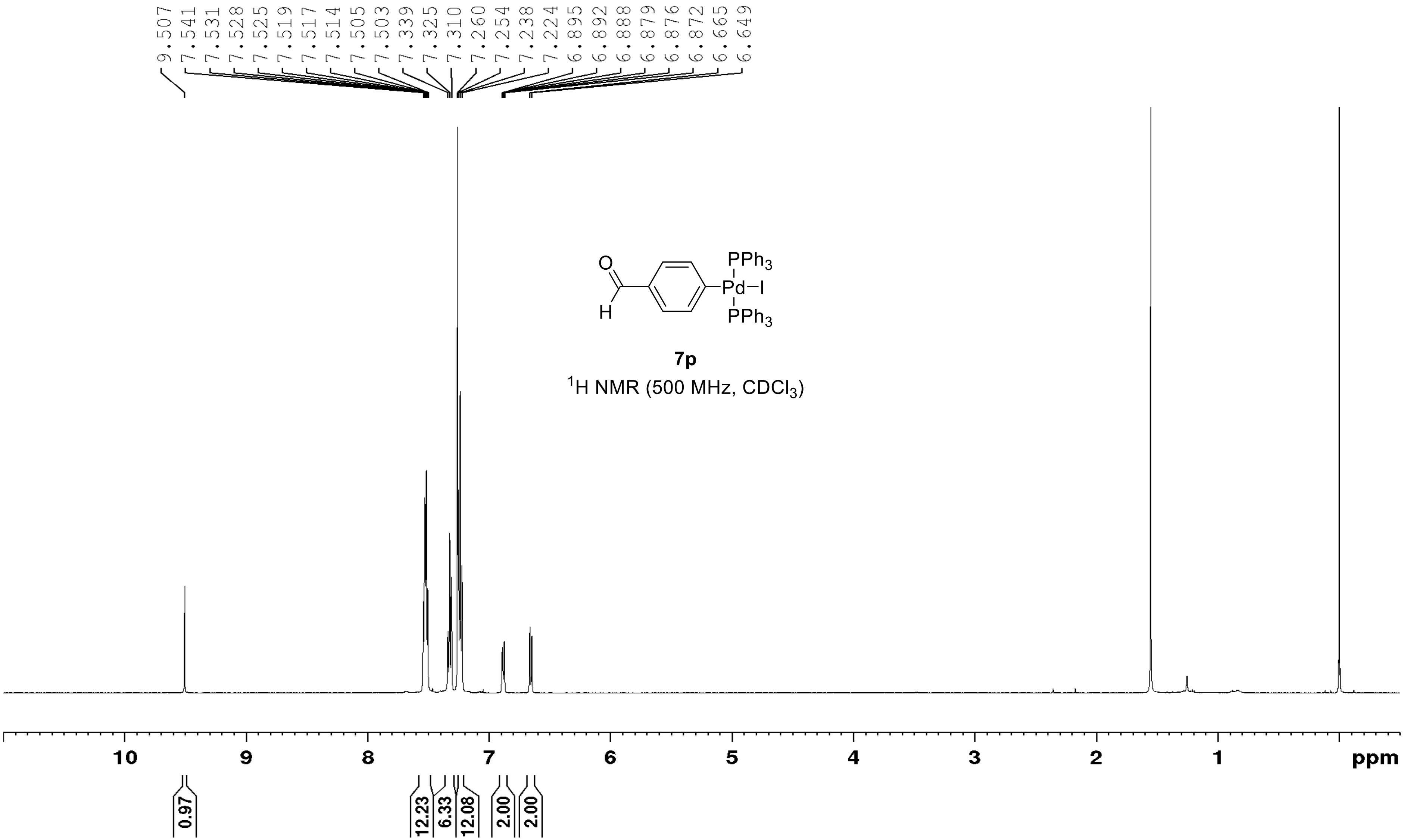
9.507 7.541 7.531 7.528 7.525 7.519 7.517 7.514 7.505 7.503 7.339 7.325 7.310 7.260 7.254 7.238 7.224 6.895 6.892 6.888 6.879 6.876 6.872 6.665 6.649
PPh3
Pd
I
PPh3
O
H
7p
1H NMR (500 MHz, CDCl3)
10 9 8 7 6 5 4 3 2 1 ppm
0.97
12.23
6.33
12.08
2.00
2.00

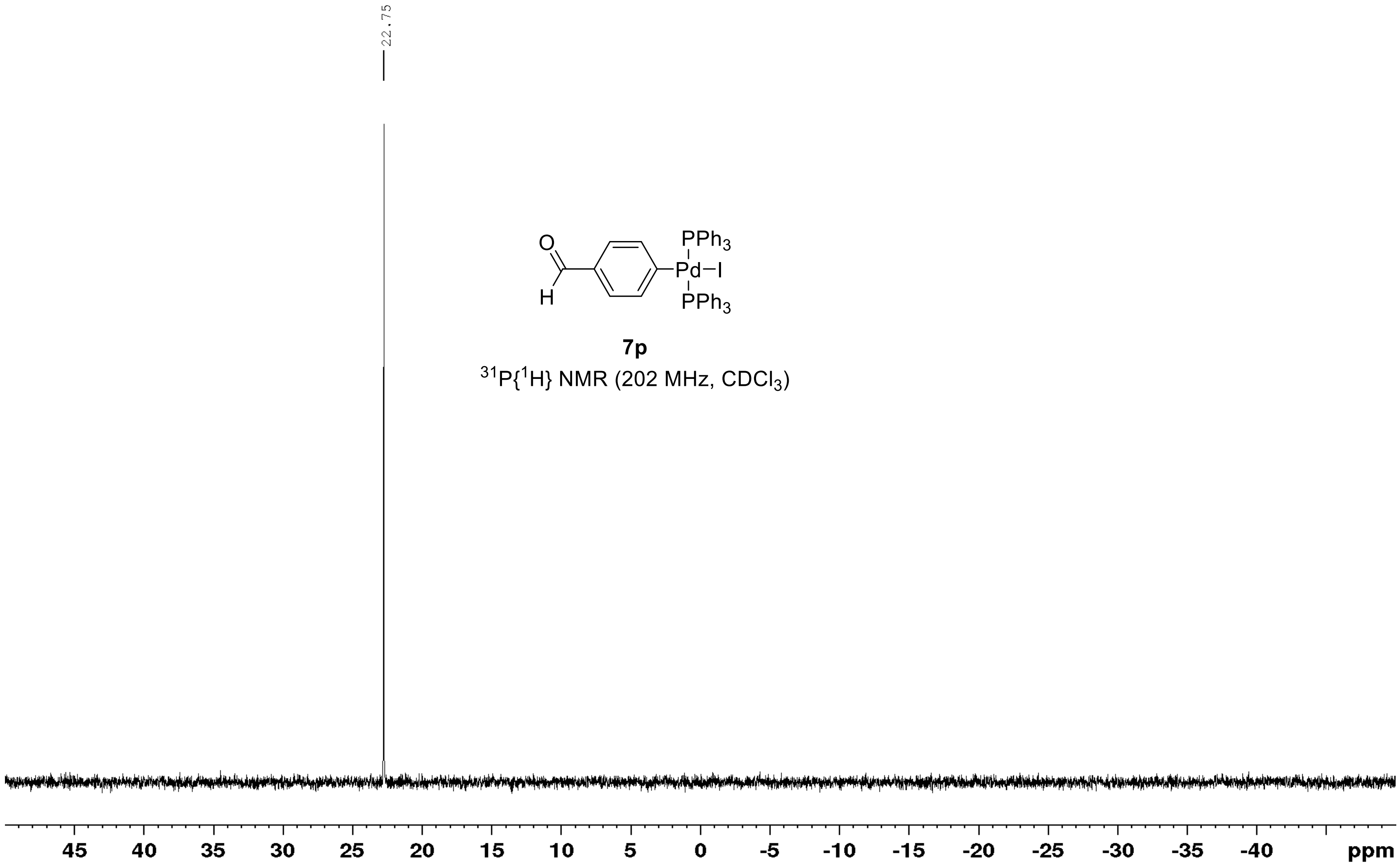

7p

$^{31}P\{^{1}H\}$ NMR (202 MHz, $CDCl_3$)

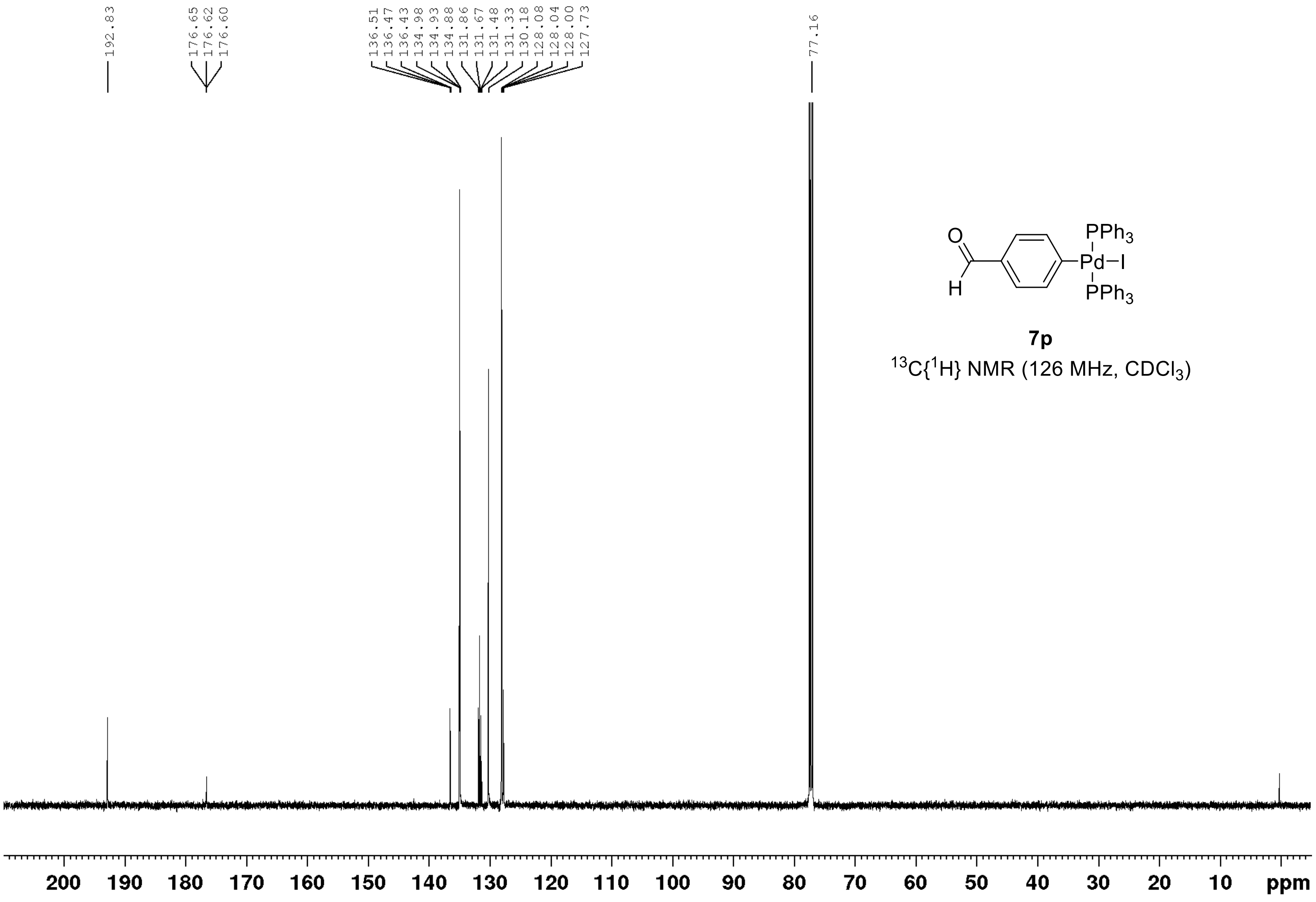
192.83
176.65
176.62
176.60
136.51
136.47
136.43
134.98
134.93
134.88
131.86
131.67
131.48
131.33
130.18
128.08
128.04
128.00
127.73
77.16
PPh3
Pd–I
PPh3
O
H
7p
13C{1H} NMR (126 MHz, CDCl3)
200 190 180 170 160 150 140 130 120 110 100 90 80 70 60 50 40 30 20 10 ppm

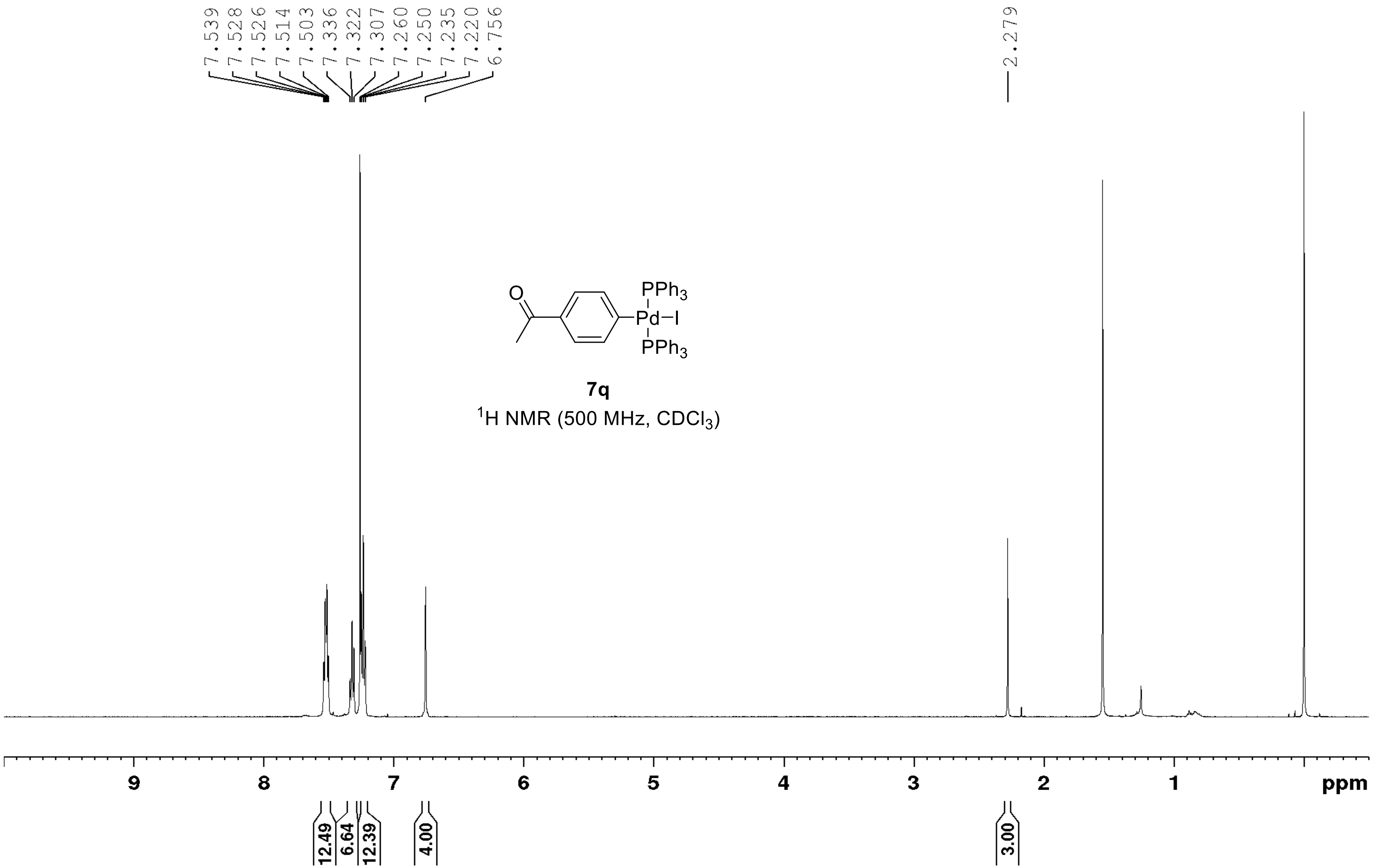
7.539
7.528
7.526
7.514
7.503
7.336
7.322
7.307
7.260
7.250
7.235
7.220
6.756
2.279
O
PPh3
Pd–I
PPh3
7q
1H NMR (500 MHz, CDCl3)
9
8
7
6
5
4
3
2
1
ppm
12.49
6.64
12.39
4.00
3.00

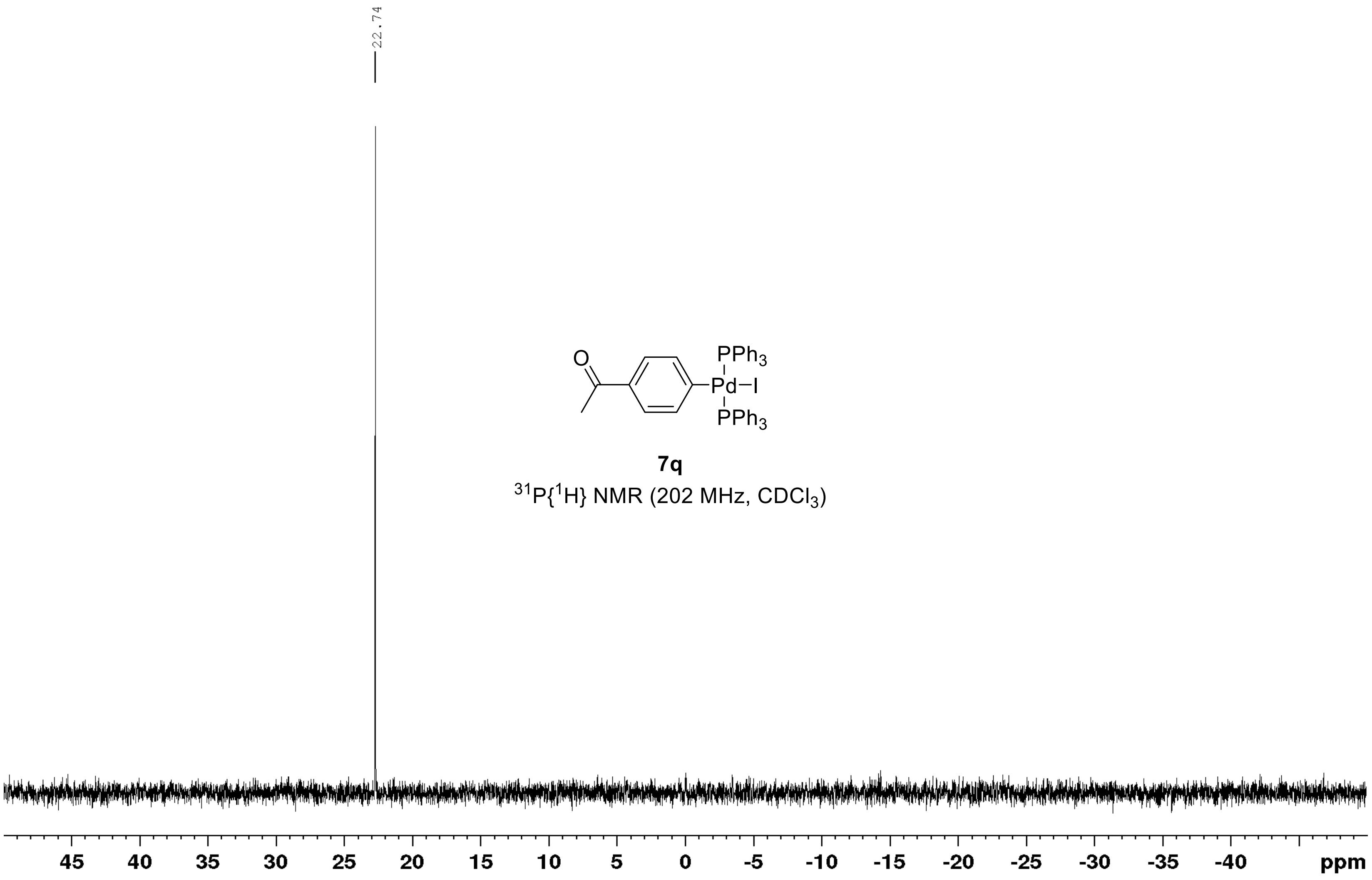
22.74
PPh3
Pd–I
PPh3
O
7q
31P{1H} NMR (202 MHz, CDCl3)
45 40 35 30 25 20 15 10 5 0 -5 -10 -15 -20 -25 -30 -35 -40 ppm

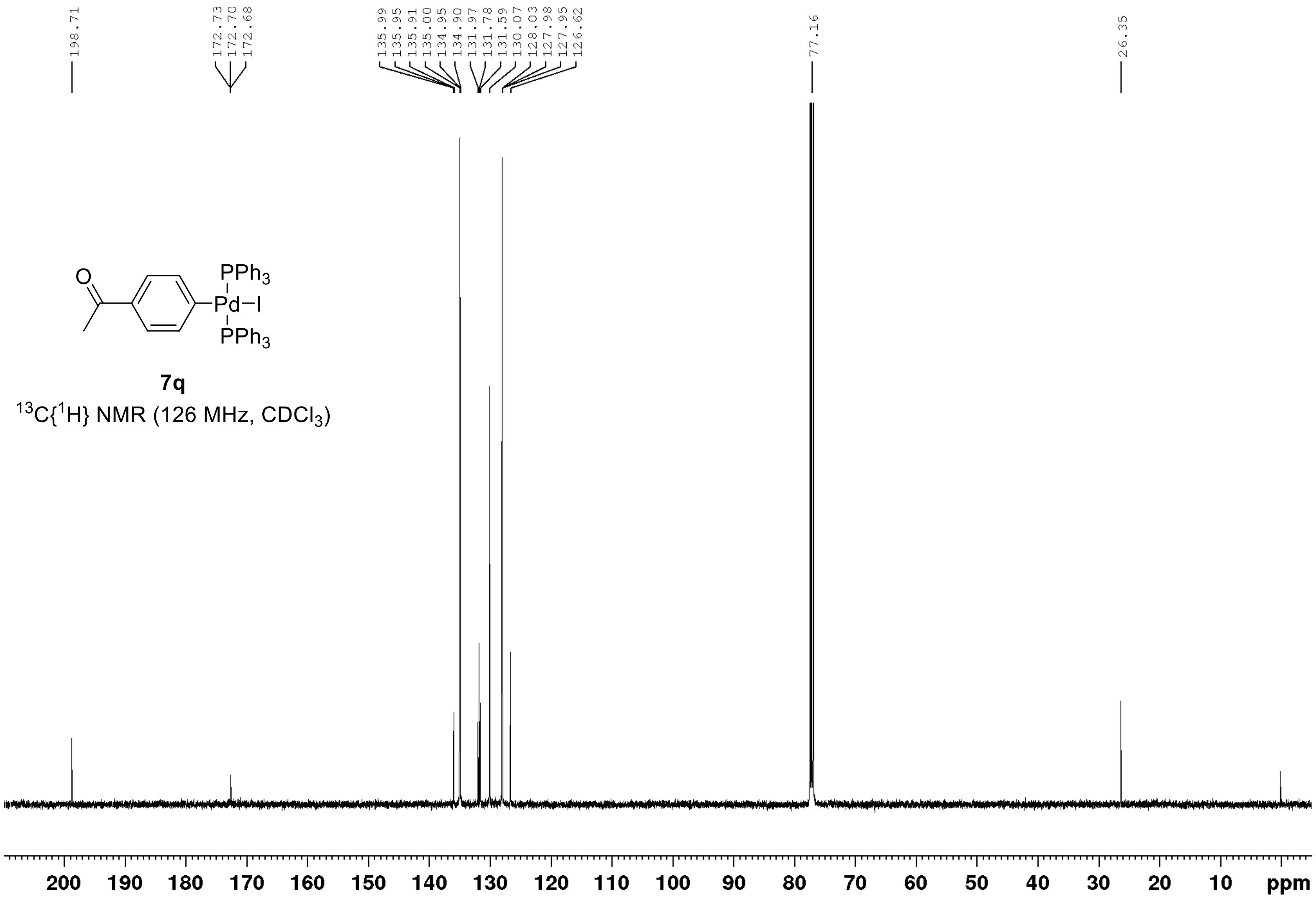

7q

$^{13}C\{^1H\}$ NMR (126 MHz, $CDCl_3$)

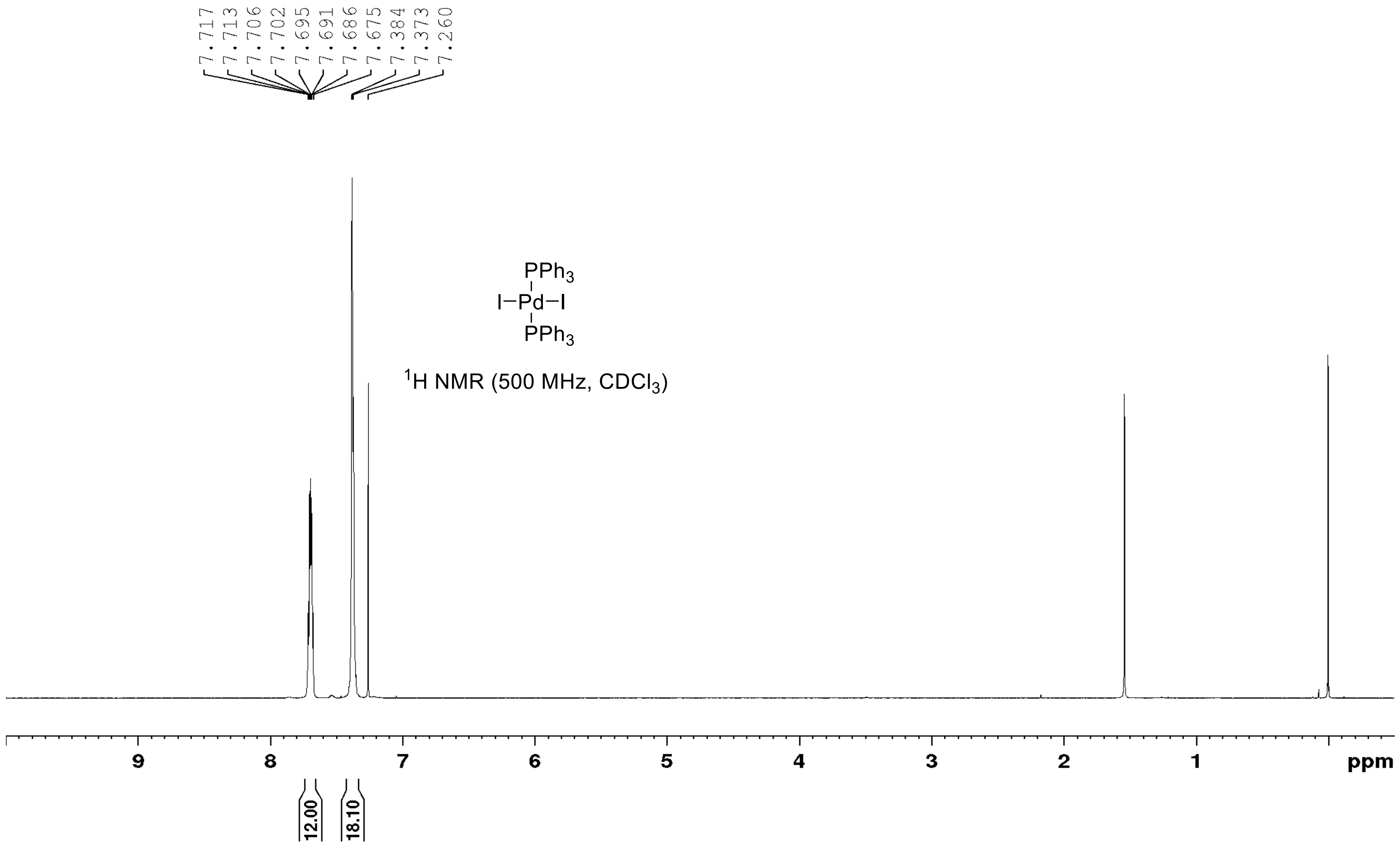
7.717
7.713
7.706
7.702
7.695
7.691
7.686
7.675
7.384
7.373
7.260
PPh3
I−Pd−I
PPh3
1H NMR (500 MHz, CDCl3)
9
8
7
6
5
4
3
2
1
ppm
12.00
18.10

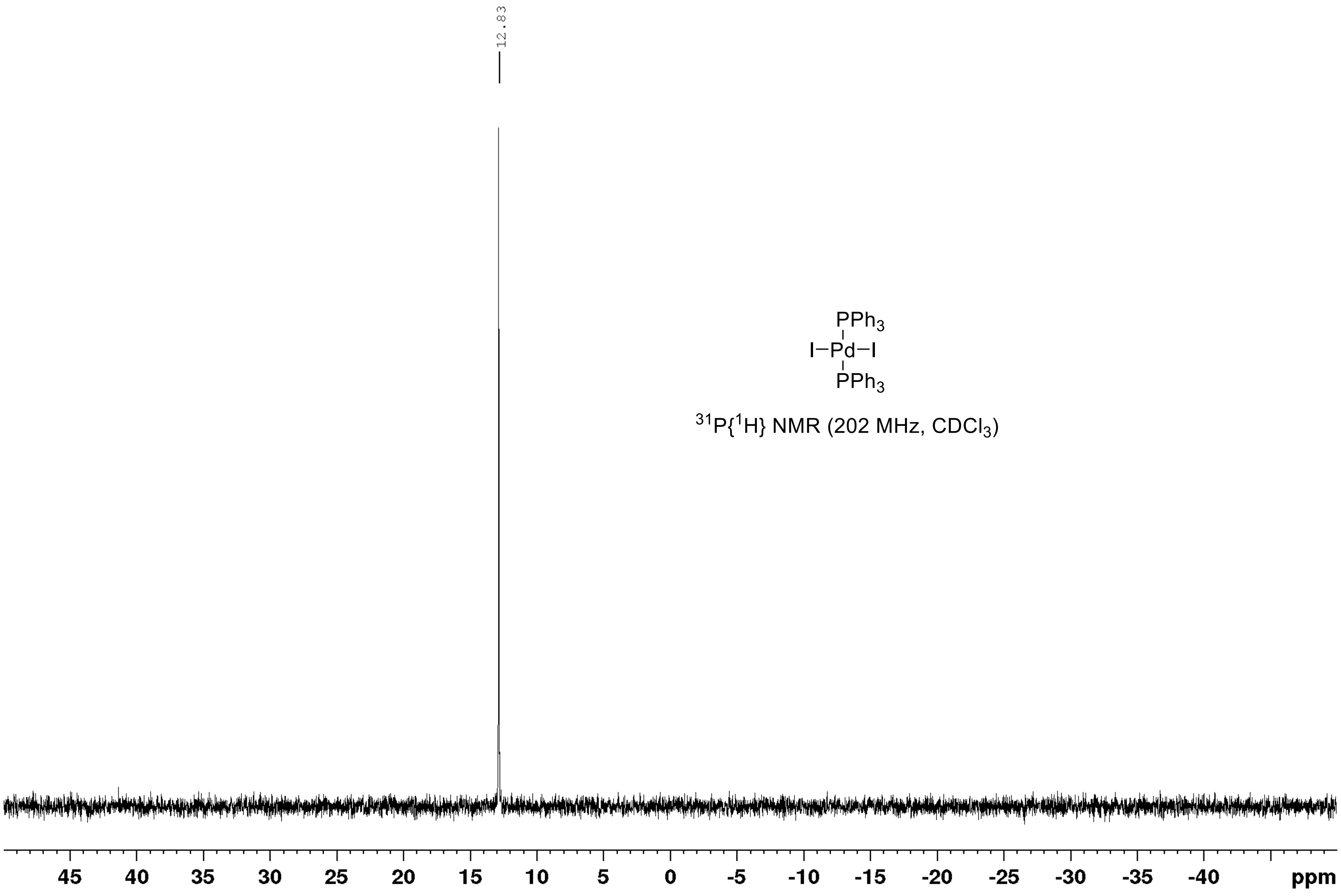

$^{31}P\{^{1}H\}$ NMR (202 MHz, $CDCl_3$)

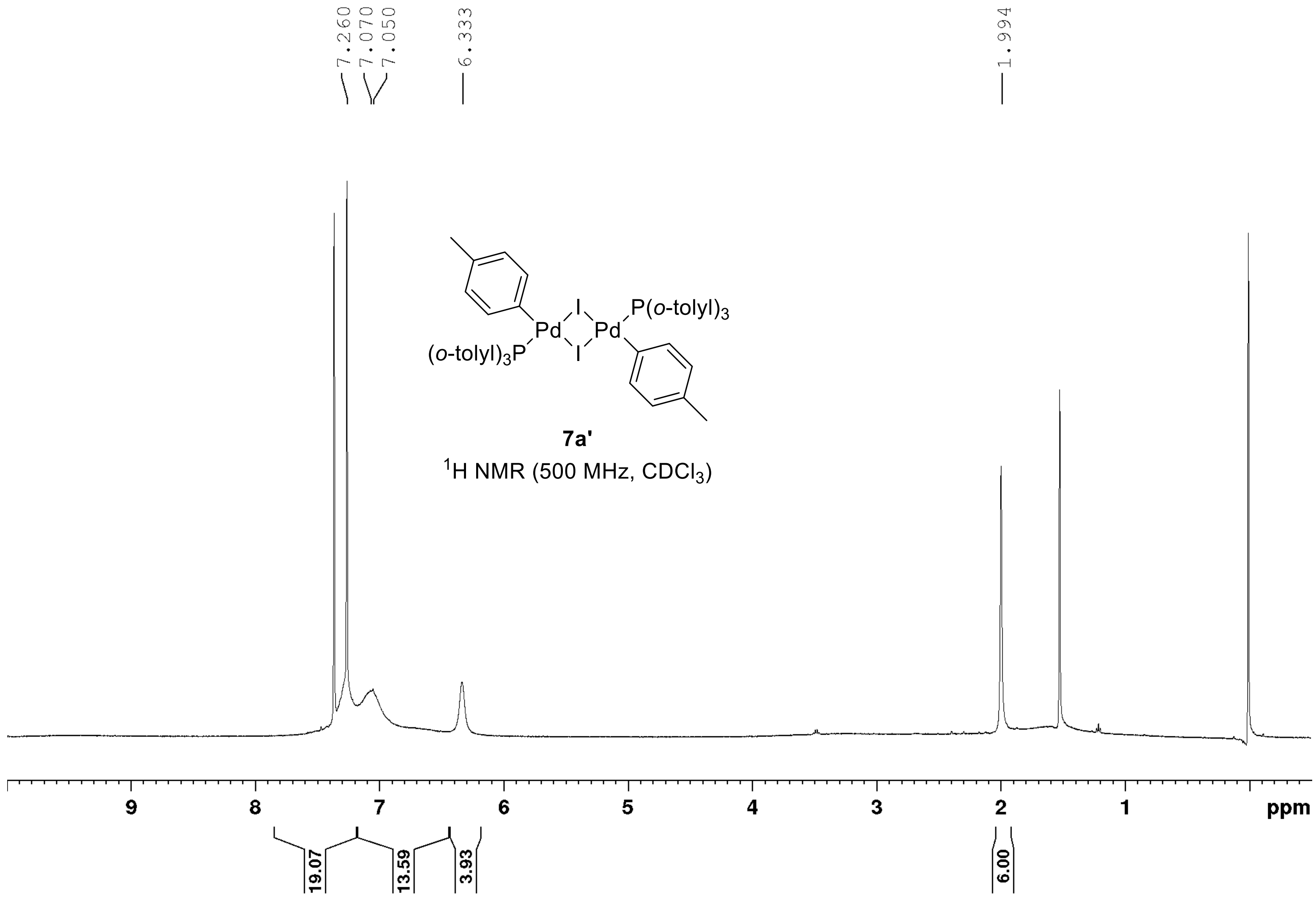
7.260
7.070
7.050
6.333
1.994
P(o-tolyl)3
Pd
I
Pd
I
(o-tolyl)3P
7a'
1H NMR (500 MHz, CDCl3)
9
8
7
6
5
4
3
2
1
ppm
19.07
13.59
3.93
6.00

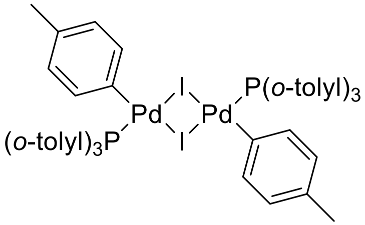

7a'

$^{31}P\{^{1}H\}$ NMR (202 MHz, $CDCl_3$)

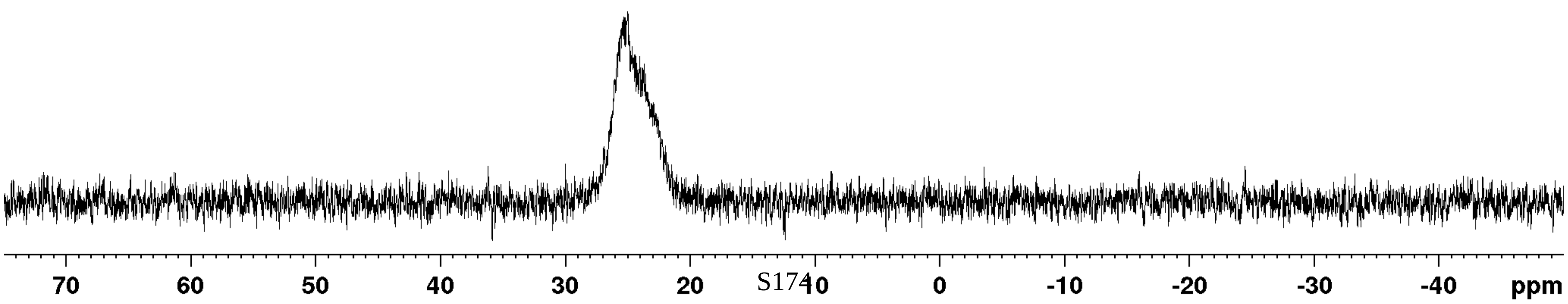

Supplement: Supplementary file 4 — Supplementary Data 1 [file 42004_2023_849_MOESM4_ESM.pdf]
